# The immunity of Meiwa kumquat against *Xanthomonas citri* is associated with a known susceptibility gene induced by a transcription activator-like effector

**DOI:** 10.1371/journal.ppat.1008886

**Published:** 2020-09-15

**Authors:** Doron Teper, Jin Xu, Jinyun Li, Nian Wang

**Affiliations:** Citrus Research and Education Center, Department of Microbiology and Cell Science, Institute of Food and Agricultural Sciences, University of Florida, Lake Alfred, United States of America; University of Missouri, UNITED STATES

## Abstract

Citrus canker caused by *Xanthomonas citri* subsp. *citri* (*Xcc*) is one of the most devastating diseases in citrus. Meiwa kumquat (*Fortunella crassifolia*) has shown a durable resistance against *Xcc*. Here, we aimed to characterize the mechanisms responsible for such a durable resistance by characterizing the transcriptional and physiological responses of Meiwa kumquat to *Xcc*. Inoculation of Meiwa kumquat with *Xcc* promoted immune responses such as upregulation of *PR* genes, accumulation of salicylic acid, hypersensitive response (HR)-like cell death and early leaf abscission. Hypertrophy and hyperplasia symptoms, which are known to be caused by *Xcc*-induction of the canker susceptibility gene *LOB1* through the transcription activator-like effector (TALE) PthA4, always appear prior to the development of cell death. Mutation of *pthA4* in *Xcc* abolished the induction of *LOB1*, canker symptoms, cell death, and leaf abscission and reduced the expression of *PR* genes in inoculated kumquat leaves without reducing *Xcc* titers *in planta*. Transcriptome analysis demonstrated that PthA4 promotes plant biotic and abiotic stress responses and the biosynthesis of abscisic acid. Transcriptional induction of *LOB1* homologs in Meiwa kumquat by *Xcc pthA4* mutant strains carrying a repertoire of designer TALEs promoted the elicitation of HR-like phenotype and leaf abscission, suggesting that kumquat response to *Xcc* is associated with upregulation of LOB1. Our study suggests a novel mechanism of plant resistance to *Xanthomonas* via elicitation of immune responses by upregulation of a host susceptibility gene.

## Author summary

Kumquats, members of the Citrus genus, are known for their durable resistance against citrus canker disease caused by the plant pathogenic bacterium *Xanthomonas citri* subsp. *citri* (*Xcc*). Here, we characterized the resistance mechanisms of Meiwa kumquat to citrus canker. We demonstrate that *Xcc* infection of Meiwa kumquat causes both immune responses and early leaf abscission, which together contribute to the resistance of kumquat to the pathogen. In addition, we showed that kumquat immune responses and early leaf abscission in the presence of *Xcc* are elicited by the transcription activator-like effector PthA4, one of the predominant pathogenicity determinants of *Xcc* in susceptible citrus varieties. PthA4-mediated elicitation of immune responses and early leaf abscission in kumquat occurs via transcriptional induction of the *LOB1* gene, which is associated with disease development in susceptible citrus varieties by promoting hypertrophy and hyperplasia symptoms. *LOB1* seems to play a dual role in the *Xcc*-kumquat interaction by acting as a susceptibility gene by promoting hypertrophy and hyperplasia symptoms in the early stages and as a potential activator of immune responses in the late stages; this poses an insurmountable challenge for the pathogen to overcome, contributing to the durable resistance of kumquat to *Xcc*.

## Introduction

*Xanthomonas* are ubiquitous pathogens that cause severe diseases on many crop plants [1]. Conversely, many crop varieties are resistant to *Xanthomonas* spp. via their innate immunity. Recognition of pathogen elicitors by the plant immune system is mediated through extracellular and intracellular receptor proteins that recognize pathogen-associated molecular patterns (PAMPs), secreted pathogen effectors or plant degradation products [damage-associated molecular patterns (DAMPs)] produced by the activity of hydrolytic enzymes secreted by invading pathogens [2–4]. Upon recognition of invading pathogens, plants initiate production of antimicrobial molecules, accumulation of pathogenesis-related (PR) proteins, stomata closure, modification of the cell wall and production of reactive oxygen species (ROS) [5]. Intracellular immune recognition of translocated pathogen effectors by plant resistance (R) proteins is usually more robust than recognition of PAMPs or DAMPs and in many cases causes localized programmed cell death known as the hypersensitive response (HR) [6].

Transcription activator-like effectors (TALEs) are bacterial effectors that are translocated into plant cells through the type 3 secretion system (T3SS) [7]. TALEs are important pathogenicity factors responsible for disease symptoms caused by many *Xanthomonas* pathogens by activating cognate susceptibility (S) genes. The specific activation occurs by binding to effector binding elements (EBEs) in the promoter regions of S genes through the central repeat region of the TALE [8,9].

Plants have evolved several independent resistance mechanisms against TALE-dependent pathogens [10]. Such resistance can be accomplished via DNA polymorphisms of the EBE region of the S gene. For example, EBE variants for the rice S genes *OsSWEET11* (also known as *Os8N3* or *Xa13*) and *OsSWEET13* (*Xa25* or *Os12N3*) function as recessive resistance alleles (*xa13* and *xa25*) that confer immunity against TALEs of *Xanthomonas oryzae* [11–14]. In addition, resistance to TALE-containing pathogens also occurs by inducing immune responses through either direct recognition of TALEs by an *R* gene (in the case of tomato, *Bs4* [15]; rice, *Xa1* [16]) or activating the expression of executor *R* genes by TALEs [17,18]. Consequently, researchers are attempting to engineer plant disease resistance against TALE-dependent *Xanthomonas* pathogens by exploiting the aforementioned mechanisms, with mutations of the EBE or the coding region of the S genes being one of the most commonly used approaches [19–23]. However, *Xanthomonas* pathogens are able to overcome engineered resistance by eliminating or altering specific effectors [24], posing a challenge for developing crop varieties with durable resistance against *Xanthomonas*.

Bacterial citrus canker caused by *Xanthomonas citri* subsp. *citri* (*Xcc*) is one of the most economically important citrus bacterial diseases worldwide [25]. The disease is characterized by the appearance of elevated pustules on the aerial surfaces of citrus plants. These symptoms are a result of tissue alterations characterized by hypertrophy and hyperplasia of infected cells [25]. PthA4, a TALE of *Xcc*, is responsible for these symptoms; PthA4 induces the expression of the canker susceptibility gene *CsLOB1* [26,27]; and disruption of *pthA4* completely abolishes the ability of *Xcc* to cause canker symptoms in grapefruit and sweet orange [27]. Lateral-organ boundary domain (LBD) proteins, such as CsLOB1, are a plant-specific protein family of transcriptional regulators that are usually associated with developmental processes [28]. Upon induction by PthA4, CsLOB1 facilitates cell hypertrophy and hyperplasia by reprogramming the cell transcriptional profile through upregulation of genes associated with DNA replication, cell cycle control, cytoskeleton remodeling and cell wall modifications [29].

Kumquats (*Fortunella* spp.), members of the *Citrus* genus, have been cultivated in many citrus-producing regions, including China, Japan, countries in Southeast Asia, and the USA, to be consumed as fruit or used in folk medicine for treating sore throats and coughing [30]. Kumquats have demonstrated durable resistance against *Xcc* infection [31,32], and much interest in understanding the resistance mechanisms of kumquats has been generated. Kumquat resistance to *Xcc* has been reported to be associated with inducing immune responses (e.g., HR) and causing early leaf abscission [33]. A long-term experimental evolution study of *Xcc* adaptation in kumquat identified multiple bacterial mutants that showed a reduced HR-like phenotype but also a decreased bacterial population in kumquat, demonstrating the limitation of *Xcc* in overcoming kumquat resistance [34]. The efficacy of kumquat resistance has been further demonstrated by its utilization in breeding programs with progeny hybrids showing *Xcc* resistance [35]. Kumquat hybrids, such as calamondin and limequat, are more resistant to *Xcc* compared to commercial citrus but slightly more susceptible to the bacterium compared to kumquats, suggesting that canker resistance is less likely to originate from a single dominant locus [36,35]. In this study, we aimed to further investigate the resistance mechanism of kumquats against *Xcc*. Indeed, understanding the mechanisms of durable resistance of kumquats against *Xcc* will have a significant impact on the generation of disease-resistant crop varieties.

## Materials and methods

### Bacterial strains, plasmids and citrus cultivars used in this study

The bacterial strains and plasmids used in this study are listed in S1 Table. *E*. *coli* cells were cultured in lysogeny broth (LB) medium at 37°C. All *Xcc* mutant and complementation strains used in this study were derivatives of *Xcc* 306 [37] (hereafter referred to as *Xcc*). *Xcc* strains were cultured in nutrient broth (NB) medium or on nutrient agar (NA) plates at 28°C. When required, media were supplemented with kanamycin (50 μg/mL), gentamicin (10 μg/mL), spectinomycin (100 μg/mL), ampicillin (100 μg/mL) and tetracycline (7.5 μg/mL). The citrus plants used in this study were Meiwa kumquat (*Fortunella crassifolia*) and Valencia sweet orange (*Citrus sinensis*). Plants were grown in a temperature-controlled (28°C) greenhouse under natural light conditions.

### Generation of *Xcc* Δ*xopE1* and *Xcc* Δ*xps* mutants

The sequences 807 bp upstream and 975 bp downstream of the *xopE1* (XAC0286) coding sequence were amplified from genomic DNA of *Xcc* 306 and joined using overlap PCR. The region 4178238–4182444 containing the *xpsM* (XAC3536), *xpsN* (XAC3535) and *xpsD* (XAC3534) coding regions of *Xcc* 306 was amplified and subcloned into pUC18 to produce pUC18*xps*. The 2886 *Eco*RV fragment (containing the 5’ end of *xpsM*, *xpsN* and *xpsD*) was digested out of pUC18*xps*, and the construct was self-ligated to produce pUC18del*xps*. Fragments containing the flanking regions of *xopE1* and *xpsM/N/D* were cloned into the pOK1 suicide vector, and plasmids were introduced into *Xcc* 306 by electroporation. Double crossover deletion mutants were generated using a two-step sucrose counter selection procedure [38].

### Genome sequencing and prediction of Meiwa kumquat promoter sequences

To obtain the promoter sequences of Meiwa kumquat, its genome was sequenced. Total DNA was extracted from Meiwa kumquat using a MoBio Powersoil DNA extraction kit (MoBio Laboratories Inc. Carlsbad, CA, USA) following the manufacturer’s instructions. Library preparation and sequencing were performed by BGI genomics (Shenzhen, China) using a DNBSEQ-T7 sequencer. High-quality 100 bp paired-end short reads (more than 160 X coverage) were mapped to reference genomes of sweet orange [39] and Hong Kong kumquat using Bowtie2 software with default parameters [40]. The promoter sequences (represented as sequences 1 kb upstream of the transcriptional start sites of all predicted coding genes) were predicated using the SAMtools/BCFtools pipeline with default settings [41]. The predicted promoter sequences are available in S1 File (for Meiwa kumquat) and S2 File (for sweet orange).

### Construction of the *Xcc pthA4*:Tn5 complementation vector and generation of *LOB*-targeting dTALEs

The broad host destination vector pBBRNPth was generated by cloning the -23 to +444 N-terminal coding fragment of *pthA4* (XACb0065) and an HA tag into pBBR1MCS-5. For construction of the *pthA4* complementation plasmid, the *Pst*I/*Eco*RI fragment of pET28-PthA4 [42] was cloned into pBBRNPth. For construction of the *pthAW2* complementation plasmid, *pthAW2* (XCAW_b00026) was amplified from the *Xcc* Aw12879 strain and cloned into pBBR1MCS-5.

Designer TALEs (dTALEs) targeting *CsLOB1*, *CsLOB2* and *CsLOB3* were designed based on RVD arrays that were previously reported to successfully complement the *pthA4* mutant [27,43]. The TAL Effector Nucleotide Targeter 2.0 target prediction tool [44](https://tale-nt.cac.cornell.edu/) [44] was used for prediction of potential targets in promoter sequences of Meiwa and sweet orange for each of the constructed TALEs to assess off-target gene induction (S8 and S9 Tables). dTALEs were assembled using “Golden Gate TALEN and TAL Effector Kit 2.0” as previously described [45] and cloned into pTAL2 as a final destination vector. The pTAL2 *Pst*I/*Eco*RI fragments containing the dTALEs were cloned into pBBRNPth, and dTALE sequences were validated by DNA sequencing. All constructs were introduced into *Xcc pthA4*:Tn5 by electroporation.

### Plant inoculations, chemical treatments and quantification of bacterial populations and leaf abscission

Bacterial suspensions (10^8^ CFU/ml for monitoring symptom development, RNA isolation and physiological measurements; 10^6^ CFU/ml for monitoring *in planta* bacterial growth) were syringe-infiltrated into young fully expanded leaves (two to three weeks after leaf emergence) of two- to four-year-old Meiwa kumquat or Valencia sweet orange plants. After inoculations, plants were kept in a temperature-controlled (28°C) greenhouse under natural light conditions.

For foliar treatments, plants were sprayed with (±)-abscisic acid (ABA), norflurazon (NF), 1-aminocyclopropane-1-carboxylic acid (ACC), α-aminoisobutyric acid (AIB) (Sigma-Aldrich, St. Louis, MO, USA) or water as a control at two, four and six days post bacterial inoculations. For each experiment, all inoculated leaves in each plant were sprayed with either 0.5 mM ABA, 1 mM NF, 0.1 mM ACC or 10 mM AIB diluted in distilled water or with distilled water as a control.

To measure bacterial growth, two 0.2-cm-diameter leaf disks from different inoculated leaves in a single plant were pooled. At least three samples from at least three different plants were homogenized in 1 mL of 10 mM MgCl_2_. Bacterial numbers were determined by plating 10 μL of 10-fold serial dilutions and counting the resulting colonies.

For quantification of leaf abscission, leaves of at least three plants were inoculated. One to three different leaves in a single plant were inoculated for each treatment. The total number of inoculated leaves was set as 100%, and plants were monitored daily for leaf drop for a period of two to four weeks. The percentage of leaf abscission for each treatment was documented daily as 100 × (number of abscissed leaves/number of inoculated leaves).

### Quantification of ion leakage, accumulation of H_2_O_2_ and leaf peroxidase activity

To quantify ion leakage, 1.5-cm-diameter leaf disks were sampled from inoculated areas and floated in 10-mL tubes containing 5 mL of double-distilled water (DDW) for 4 h at 25°C with shaking. Conductivity was measured using a CON 700 conductivity/°C/°F bench meter (OAKTON Instruments, Vernon Hills, IL, USA).

For quantification of H_2_O_2_ and leaf peroxidase activity, six 0.2-cm-diameter leaf disks were pooled from three leaves inoculated with an identical treatment from the same plant and kept in a 1.5 mL tube with 1 mL of DDW. Samples (three to four samples taken from different plants) were kept on a rotating platform at 20 rpm for 30 min, and DDW was replaced with fresh DDW. Samples were incubated for an additional 6 h on a rotating platform at 20 rpm. H_2_O_2_ was quantified in the supernatants using the Pierce Quantitative Peroxide Assay Kit (Thermo Fisher Scientific, Waltham, MA, USA) as instructed by the manufacturer.

Leaf peroxidase activity measurements were conducted as previously described with modifications [46]. Briefly, sample supernatants were diluted 10-fold, and 50 μL of diluted supernatants from each sample was incubated with 50 μL of peroxidase assay solution [1 mg/ml 5-amiosalycliclc acid (5-ASA), 0.01% H_2_O_2_, pH 6.0]. Samples were timed, and the reaction was stopped with 20 μL of 2 N NaOH once brown pigmentation started to appear. After the reaction was stopped, the optical density (OD) was measured at 600 nm. Peroxidase activity was determined in arbitrary units (AU) and calculated as OD = 600/[time (min) × leaf disk surface area (cm)].

H_2_O_2_ and peroxidase activity were quantified in 96-well plates using a Synergy HT Plate Reader (BioTek, Winooski, VT, USA).

### Isolation and quantification of leaf SA and ABA

Salicylic acid (SA) and abscisic acid (ABA) were isolated from *Xcc-* or mock-inoculated leaf tissues four days post inoculation. For each sample, ten to twenty 1.5-cm-diameter leaf disks subjected to the same treatment were pooled from different leaves in the same plant and frozen in liquid nitrogen. Samples were ground into fine powder using a mortar and pestle, and 100 mg samples were transferred into 2 ml tubes.

SA was extracted and quantified as described previously [47]. The samples were analyzed with an Agilent 1260 Infinity Quaternary Liquid Chromatography system. SA was separated in an Eclipse plus C18 column (Agilent, 50 mm × 4.6 mm) at 26°C and detected with a fluorescence detector at 305 nm excitation and 407 nm emission wavelengths. The mobile phase contained 0.2 M sodium acetate buffer (pH 5.5) and methanol (90%:10%). The flow rate was 0.8 mL/min, and the autosampler was configured to inject 20 μl aliquots of samples.

The ABA extraction protocol was modified from [48]. Briefly, 100 mg samples were mixed with extraction solution [80% MeOH, 250 mg/1 L 2- and 6-di-tert-butyl-4-methylphenol (BHT)] and incubated at -20°C for 12 h. Samples were centrifuged, and supernatants were collected and kept overnight in a fume hood to be evaporated. Samples were resuspended in 2% acetic acid, mixed with 1 volume of ethyl acetate and inverted vigorously. The ethyl acetate phase was collected, and the procedure was repeated twice. Ethyl acetate fractions were pooled and evaporated overnight in a fume hood. Pellets were resuspended in 50 μL of MeOH, and 950 μL of Tris-buffered saline was added once pellets were completely dissolved in MeOH. ABA was quantified by an Abscisic Acid Immunoassay Detection Kit (Sigma-Aldrich, St. Louis, MO, USA) as instructed by the manufacturer using a Synergy HT Plate Reader (BioTek, Winooski, VT, USA).

### RNA isolation and RT-qPCR

RNA was isolated from mock- or *Xcc-*inoculated leaf tissues at one, two, three and four days post infiltration. For each sample, three 1-cm-diameter leaf disks from inoculated areas were pooled from three leaves belonging to the same plant and frozen in liquid nitrogen. Samples were ground into fine powder using TissueLyser II (QIAGEN, Hilden, Germany). Total RNA was extracted using TRIzol Reagent (Thermo Fisher Scientific, Waltham, MA, USA) and incubated with RQ1 RNase-Free DNase (Promega, Madison, WI, USA). For RNA-seq analysis, a second RNA-isolation step was conducted using phenol-chloroform purification [49]. For RT-qPCR, 1 μg RNA samples were reverse-transcribed using a qScript cDNA Synthesis Kit (Quanta BioSciences, Inc. Gaithersburg MD, USA). cDNAs were amplified with gene-specific primers (S2 Table, gene symbols and the corresponding common gene names are indicated) using SYBR Premix Ex Taq II (Clontech Laboratories, Inc. Mountain View, CA, USA) by the QuantStudio 3 Real-Time PCR System (Applied Biosystems Inc., Foster City, CA). The *GAPDH* gene was used for normalization, and gene expression was calculated by the comparative *Ct* method [50].

### RNA-seq and analysis

Total RNA from three replicates of *Xcc* WT- or *Xcc pthA4*:Tn5-inoculated Meiwa kumquat leaves was isolated one day and four days after inoculation and sent for RNA sequencing (RNA-seq). Library preparation and mRNA sequencing were conducted by Novogene Co., Ltd., Beijing, China. Libraries were prepared using the NEB Next Ultra RNA Library Prep Kit (NEB, Ipswich, MA, USA) and sequenced using the Illumina NovaSeq 6000 platform with 2 × 150 bp paired-end reads (PE 150). The RNA raw reads were deposited in the NCBI SRA database under the bio-project accession no. PRJNA596239.

Raw paired-end reads were mapped to the sweet orange reference genome [39], and a gene expression profile was generated using the HTseq package with default settings [51]. Based on the gene expression profile, we used the R package DESeq2 (v1.20.0) [52] to determine the differentially expressed genes between groups (|fold change| ≥3 and adjusted P value ≤ 0.05).

For functional enrichment analysis, the KO and pathway information of genes from the sweet orange genome were generated using the KEGG KOALA pipeline [53]. KEGG pathway enrichment analysis of all DEGs was performed using Fisher’s exact test [54]. Gene ontology (GO) term enrichment of DEGs was analyzed using agriGO v2.0: a GO analysis toolkit for the agricultural community [55] using the singular enrichment analysis tool (http://systemsbiology.cau.edu.cn/agriGOv2/classification_analysis.php?category=Plant&&class1=Tree).

### Cloning and sequencing of the *LOB1*, *LOB2* and *LOB3* genomic regions of Meiwa kumquat

Genomic DNA was extracted from fully expanded leaves of Meiwa kumquat using NucleoSpin Plant II (TaKaRa Bio Inc. Kusatsu, Japan). Four fragments, including one fragment covering a 3,456 bp (NCBI accession num’ MT247386) containing the *LOB1* promoter, coding region and the PthA4 EBE sequences; one fragment covering 1,264 bp (NCBI accession num’ MT247387) containing the *LOB2* promoter and coding regions; and one fragment covering 1,515 bp (NCBI accession num’ MT655137) containing the *LOB3* promoter and coding regions, were amplified (primers are shown in S2 Table) from genomic DNA using Q5 High-Fidelity DNA Polymerase (NEB, Ipswich, MA). The *LOB1*, *LOB2* and *LOB3* fragments were cloned into pGEM-T (Promega, Madison, WI) or pHSG298 (TaKaRa Bio Inc. Kusatsu, Japan) vectors. Sequences were determined using Sanger sequencing (Eton Bioscience, Inc., San Diego, CA).

## Results

### *Xcc* induces immune responses and early leaf abscission in Meiwa kumquat

**Fig 1:**
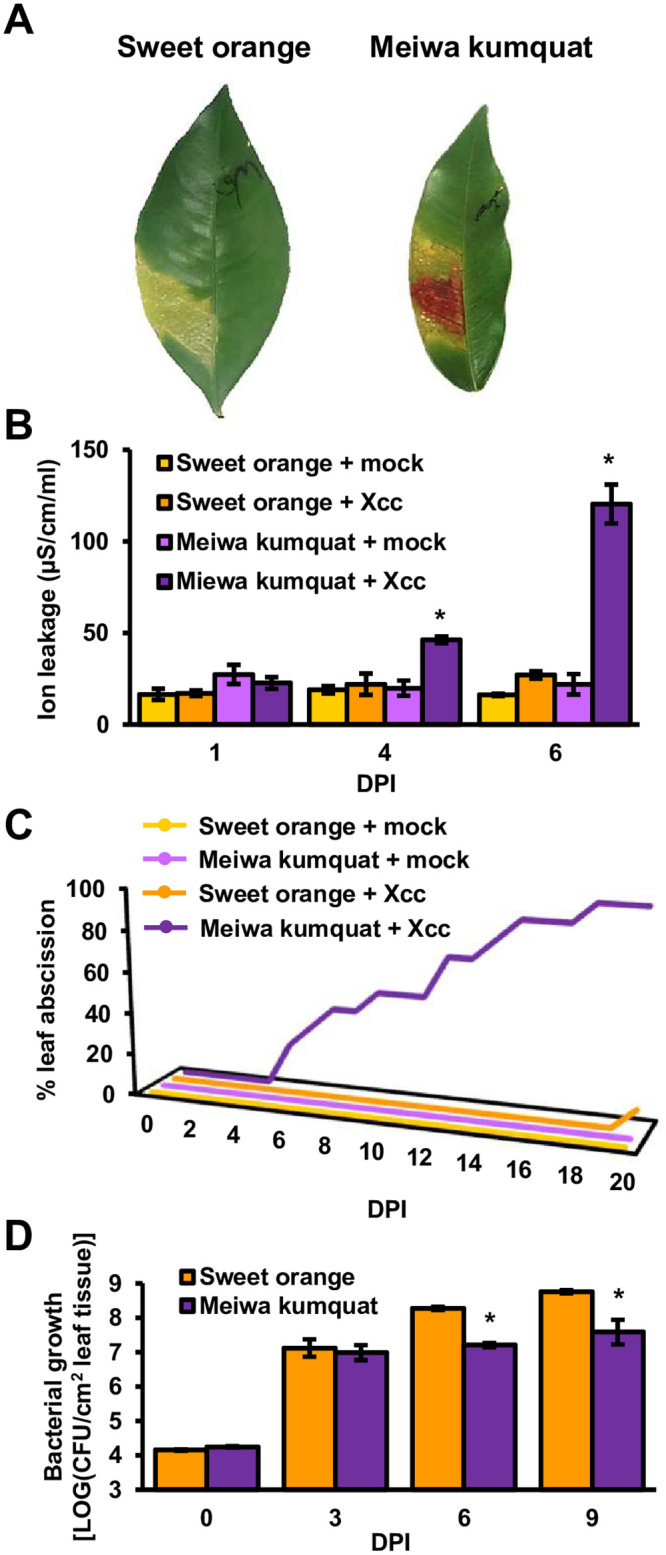
*Xcc* promotes the HR-like phenotype and early leaf abscission in Meiwa kumquat. *Xcc* cultures (10^8^ CFU/ml for A, B and C; 10^6^ CFU/ml for D) or 10 mM MgCl_2_ (mock) were syringe-infiltrated into sweet orange or Meiwa kumquat leaves. (**A**) Infected leaves were photographed eight days post inoculation (DPI). (**B**) Cell death was quantified by electrolyte leakage at the indicated DPI. Values represent the means ± SE of three (for mock) or four (for *Xcc* inoculated) independent leaves taken from different plants. Asterisks indicate samples that were significantly different (Student’s t-test, *p value* < 0.05) compared to sweet orange leaves inoculated with *Xcc* at the same DPI. (**C**) Graph represents the percentage of abscissed leaves at the indicated DPI. n = 11 for leaves inoculated with *Xcc* (maximum of two leaves per plant). n = 5 for mock-treated leaves (one leaf per plant). (**D**) Leaf *Xcc* bacterial populations were quantified at the indicated DPI. Values represent the means ± SE of three independent leaves taken from different plants. Asterisks indicate samples that were significantly different (Student’s t-test, *p value* < 0.05) compared to sweet orange leaves inoculated with *Xcc* at the same DPI. All depicted experiments were repeated at least three times with similar results.

The physiological responses of sweet orange and Meiwa kumquat to *Xcc* infection were assessed. Sweet orange and Meiwa kumquat were syringe-inoculated with *Xcc* (10^8^ CFU/ml); both plants initially exhibited classic early canker symptoms, such as hypertrophy and hyperplasia, between four to six days post inoculation (DPI). However, *Xcc*-infected kumquat leaves displayed an HR-like phenotype between 36–72 h after the appearance of canker symptoms, which inhibited further canker symptom development (Fig 1A and S1 Fig). Increased ion leakage, an indicator for cell death, was observed in *Xcc-*inoculated kumquat but was neglectable in susceptible sweet orange (Fig 1B). Additionally, we observed early abscission of kumquat leaves inoculated with *Xcc*. Leaf abscission occurred approximately at the same time as the HR-like phenotype was observed or slightly later. Almost all infected leaves were detached between 7–15 DPI (Fig 1C). We did not observe significant leaf abscission in *Xcc*-inoculated susceptible sweet orange for three weeks after inoculation (Fig 1C), and abscission usually occurred between four and eight weeks post inoculation in approximately half of the inoculated leaves. Since immunity is usually associated with halting or decreasing bacterial growth, bacterial populations were monitored. We observed an approximately 10-fold reduction in the *Xcc* bacterial population at the late infection stages in kumquat compared to sweet orange, and bacteria were able to colonize the kumquat leaf to approximately 10^7^–5 × 10^7^ CFU/cm^2^ leaf tissue (Fig 1D).

**Fig 2:**
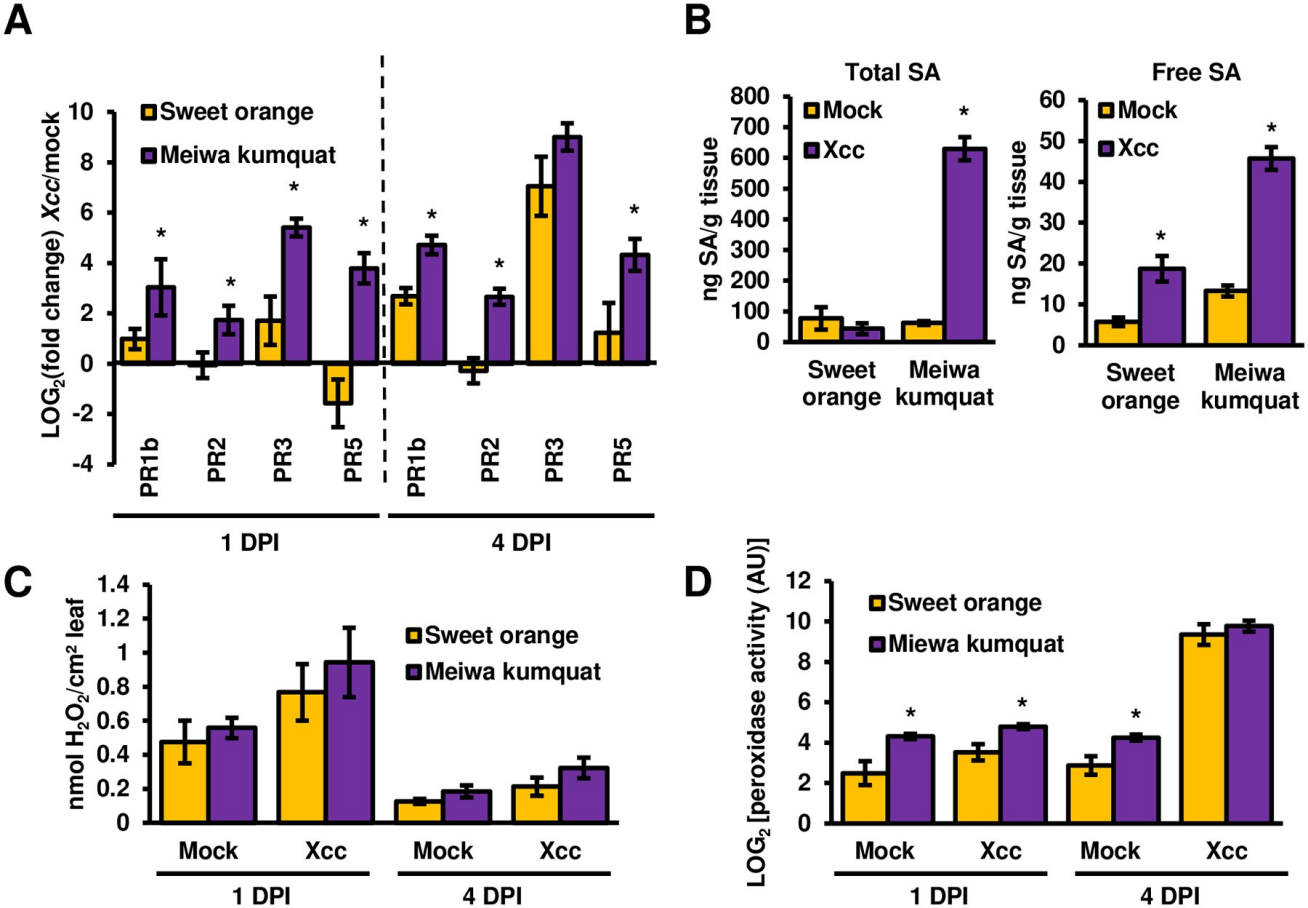
*Xcc* induces defense responses in Meiwa kumquat. *Xcc* cultures (10^8^ CFU/ml) or 10 mM MgCl_2_ (mock) was syringe-infiltrated into sweet orange or Meiwa kumquat leaves. (**A**) The mRNA abundance (*Xcc*-inoculated/mock-treated) of the indicated citrus genes was quantified at one and four days post inoculation (DPI). The mRNA abundance of *GAPDH* was used for normalization. Values represent the means ± SE of three independent experimental repeats. Asterisks indicate a significant difference (Student’s *t* test, *p* value < 0.05) relative to sweet orange. (**B**) Total and free salicylic acid (SA) were quantified at four DPI. Values represent the means ± SE of at least three independent leaves taken from different plants. Asterisks indicate a significant difference (Student’s *t* test, *p* value < 0.05) relative to mock-treated leaves. (**C**) H_2_O_2_ accumulation was quantified at one and four DPI. Samples were pulled from three independent experimental repeats (at least three independent leaves taken from different plants for each experiment). Values represent the means ± SE (n = 9 for day 1, n = 12 for day 4). (**D**) Secreted leaf peroxidase activity was quantified by using 5-*aminosalicylic acid* (5-ASA) as a substrate at one and four DPI. Values represent the means ± SE (n = 3) of arbitrary peroxidase activity units (AU). Asterisks indicate a significant difference (Student’s *t* test, *p* value < 0.05) relative to sweet orange. All depicted experiments were repeated three times with similar results.

To examine whether the response of kumquat to *Xcc* harbors hallmarks of immune responses, we investigated the expression of several plant defense-associated genes, SA accumulation, peroxidase activity and production of reactive oxygen species (ROS) at the early (one DPI) and mid-to-late stages of infection (four DPI). The expression of four tested *PR* genes (*PR1b*, *PR2*, *PR3* and *PR5*) was induced by *Xcc*, and the induction was significantly higher in kumquat at one and four DPI compared to sweet orange (Fig 2A). Both free and total SA levels were significantly elevated in kumquat in response to *Xcc* infection at four DPI compared to sweet orange (Fig 2B). Hydrogen peroxide levels were elevated in both plants compared to the mock treatment. We did not observe significant differences in hydrogen peroxide levels between kumquat and sweet orange leaves inoculated with *Xcc* (Fig 2C). At four DPI, peroxidase activity was induced between 100-200-fold in both kumquat and sweet orange compared to mock treatment, and no significant differences were observed between the two plants (Fig 2D).

Overall, the results demonstrated that *Xcc* induces immune responses in Meiwa kumquat and that kumquat immunity to *Xcc* is characterized by eliciting cell death and early leaf abscission.

### The immune responses of Meiwa kumquat to *Xcc* are mediated by PthA4

A previous experimental evolution study conducted by our group demonstrated that adapted *Xcc* bacteria that caused reduced HR-like phenotype in Meiwa kumquat harbor point mutations in a *xps* type 2 secretion system (T2SS) gene and the T3SS effector coding genes *xopE1/avrXacE1* and *pthA4* [34]. We hypothesized that these genes mediate kumquat immune responses to *Xcc*. Therefore, we examined whether kumquat leaves inoculated with *Xcc* mutant strains disrupted in *pthA4*, *xopE1* or *xps* T2SS display altered immune responses compared to the *Xcc* wild-type (WT) strain.

**Fig 3:**
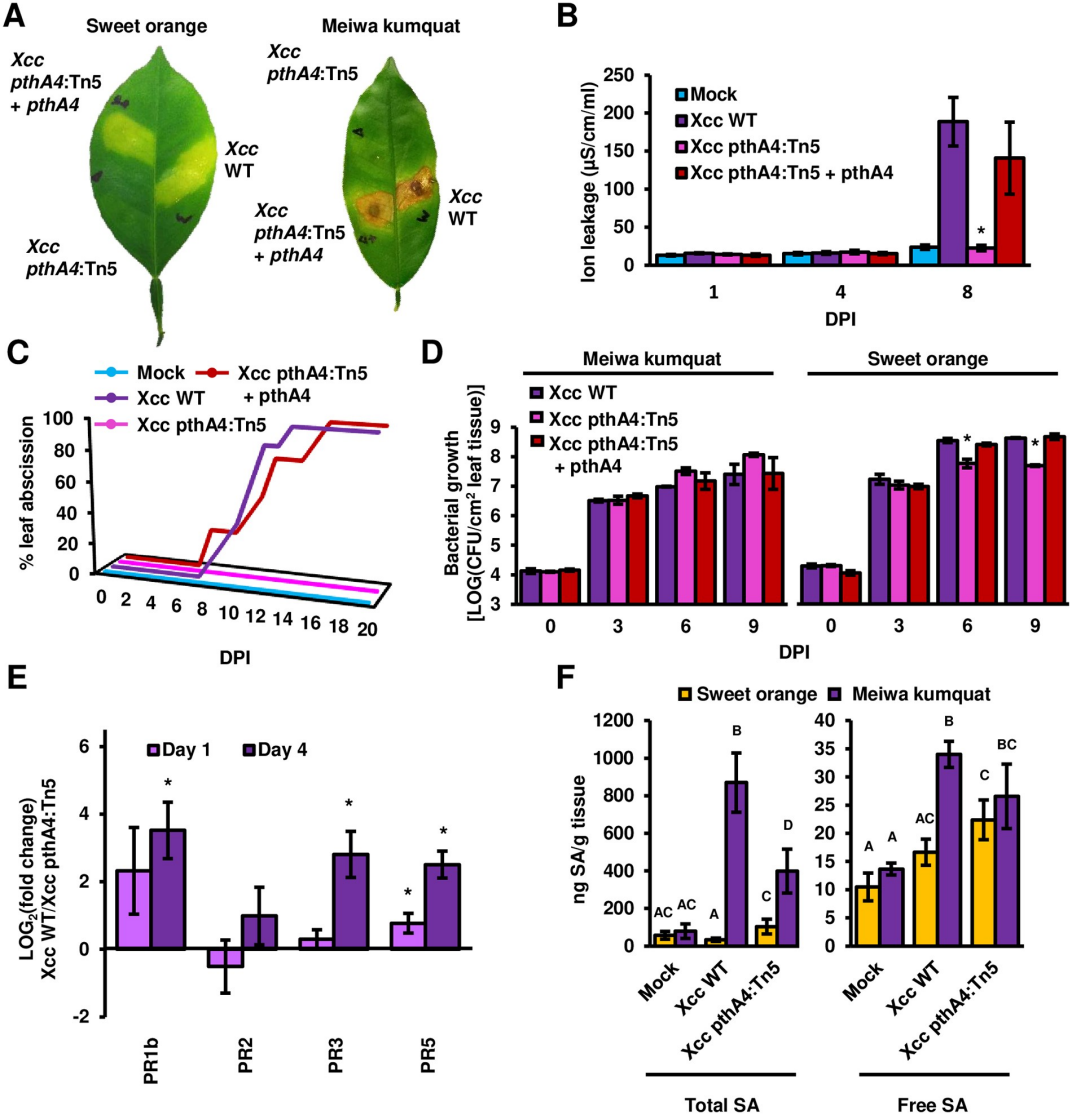
PthA4 contributes to *Xcc* responses in Meiwa kumquat. The indicated *Xcc* cultures (10^8^ CFU/ml for **A**, **B**, **C**, **E** and **F**; 10^6^ CFU/ml for **D**) or 10 mM MgCl_2_ (mock) were syringe-infiltrated into Meiwa kumquat or sweet orange (**A**, **D** and **F**) leaves. (**A**) Infected leaves were photographed eight days post inoculation (DPI). (**B**) Cell death was quantified by electrolyte leakage at the indicated DPI. Values represent the means ± SE of at least three independent leaves taken from different plants. Asterisks indicate samples that were significantly different (Student’s t-test, *p value* < 0.05) compared to leaves inoculated with *Xcc* WT at the same DPI. Experiments were repeated three times with similar results. (**C**) Graph represents the percentage of abscissed leaves at the indicated DPI. n = 8 for leaves inoculated with *Xcc* (two leaves per plant). n = 4 for mock-treated leaves (one leaf per plant). Experiments were repeated three times with similar results. (**D**) *Xcc* bacterial populations in leaves were quantified at the indicated DPI. Values represent the means ± SE of three independent leaves taken from different plants. Asterisks indicate samples that were significantly different (Student’s t-test, *p value* < 0.05) compared to leaves inoculated with *Xcc* WT at the same DPI. Experiments were repeated three times with similar results. (**E**) The mRNA abundance (*Xcc* WT-infected leaves/*Xcc pthA4*:Tn5-infected leaves) of the indicated citrus genes was quantified in Meiwa kumquat at one and four DPI. The mRNA abundance of *GAPDH* was used for normalization. Values represent the means ± SE of three independent experimental repeats. Asterisks indicate a significant difference (Student’s *t* test, *p* value < 0.05) relative to leaves inoculated with *Xcc pthA4*:Tn5 at the same DPI. (**F**) Total and free salicylic acid (SA) were quantified at four DPI. Values represent the means ± SE of pulled biological replicates (n = 4 for free SA of mock-treated kumquat, n ≥ 5 for the rest) taken from three experimental repeats. Letters denote significant similarities (Student’s *t* test, *p* value < 0.05) between samples.

When inoculated with the *Xcc pthA4* mutant (*Xcc pthA4*:Tn5), kumquat leaves did not display an HR-like phenotype or early leaf abscission (Fig 3A, 3B and 3C). Mutation of *pthA4* also caused a significant reduction in the expression of defense-related genes (*PR1*, *PR3* and *PR5*) and the accumulation of total SA in kumquat compared to *Xcc* WT (Fig 3E and 3F). While colonization of sweet orange by *Xcc pthA4*:Tn5 was significantly reduced compared to that by *Xcc* WT, kumquat colonization by *Xcc pthA4*:Tn5 was similar to or slightly higher than that by *Xcc* WT, reaching approximately 5 × 10^7^ CFU/cm^2^ leaf tissue (Fig 3D). It is worth noting that PthA4 is critical for both pathogenicity and inducing immune responses, probably explaining the subtle increase of *Xcc pthA4*:Tn5 population in kumquat than that of *Xcc* WT. A complementation plasmid expressing *pthA4* under the *lac* promoter was introduced into *Xcc pthA4*:Tn5, and the ability to induce an HR-like phenotype and leaf abscission in kumquat leaves was restored (Fig 3A, 3B, 3C and 3D).

*Xcc* Δ*xps* caused a reduction in immune responses and leaf abscission in kumquat compared to the *Xcc* WT (S2A and S2B Fig). However, *Xcc* Δ*xps* was not able to colonize the leaves to the same extent as *Xcc* WT (S2C Fig); thus, it was excluded from further analysis. *Xcc* Δ*xopE1* induced the HR-like phenotype and leaf abscission in a similar manner as *Xcc* WT (S2A and S2B Fig).

Taken together, we show that the TALE PthA4 is required to elicit immune responses and early leaf abscission in kumquat.

### Transcriptional response of Meiwa kumquat to PthA4

To examine how PthA4 induces immune responses and early leaf abscission in kumquat, transcriptome analysis was conducted in kumquat leaves inoculated (10^8^ CFU/ml) with *Xcc* WT compared to *Xcc pthA4*:Tn5. RNA-seq analyses identified 280 (134 upregulated and 146 downregulated) and 1500 (877 upregulated and 623 downregulated) differentially expressed genes (DEGs) (FDR *P value* < 0.05, cutoff was set at three-fold change) between leaves inoculated with *Xcc* WT and *Xcc pthA4*:Tn5 at one and four DPI, respectively (S3 Table, S3 Fig). Forty-three DEGs were selected for qRT-qPCR analysis; these genes showed similar expression trends as the RNA-seq analysis (S4 Table), demonstrating the reliability of the RNA-seq data.

We conducted KEGG and Gene Ontology (GO) enrichment analyses (Fisher’s exact test, *P value* < 0.05) of the PthA4-dependent DEGs in kumquat (Tables 1–3)(S5, S6 and S7 Tables). Similar to a previous analysis conducted in sweet orange [29], PthA4 induced the upregulation of genes associated with DNA replication and metabolism, cell wall organization, cytoskeleton and sugar metabolism (Table 1 and S6 Table) and the downregulation of genes associated with photosynthesis, flavonoid biosynthesis, amino acid metabolism and fatty acid metabolism (Table 2 and S7 Table). In kumquat, PthA4 was also able to promote the expression of stress-, defense- and immunity-associated genes (S6 Table). This indicates that both susceptibility-associated and defense-associated genes are induced in parallel by PthA4 in kumquat. To further investigate how PthA4 induces immune responses and early leaf abscission, we examined the differential regulation of defense and hormone biosynthesis genes. PthA4 promoted the upregulation of genes that are known to actively facilitate defense responses or antimicrobial activity, including the *CAP/PR1*, *THAUMATIN/PR5*, *BETV 1/PR10*, *POLYPHENOL-OXIDASE (PPO*), *PAPAIN-LIKE CYSTEINE PROTEASES* and *SNAKIN* genes [56–61] (S4A and S4B Fig). Among the hormone-related pathways induced by PthA4, we identified upregulated genes belonging to the ABA and ethylene biosynthesis pathways at either one or four DPI (S4C and S4D Fig).

**Table 1:** Significantly enriched upregulated KEGG pathways in *Xcc* WT inoculated Meiwa kumquat (4 DPI) plants compared to *Xcc pthA4*:Tn5.

KEGG pathway	*p-value*
DNA replication proteins	1.29E-30
Cytoskeleton proteins	2.91E-09
Cellular senescence	3.50E-06
DNA repair and recombination proteins	0.00067
Glycosyltransferases	0.0031
Protein kinases	0.004
Plant hormone signal transduction	0.0074
Homologous recombination	0.01
Pentose and glucuronate interconversions	0.0138
MAPK signaling pathway	0.014
AMPK signaling pathway	0.05

**Table 2:** Significantly enriched downregulated KEGG pathways in *Xcc* WT inoculated Meiwa kumquat (4 DPI) plants compared to *Xcc pthA4*:Tn5.

KEGG pathway	*p-value*
Phenylpropanoid biosynthesis	5.78E-14
Cytochrome P450	1.08E-11
Alpha-Linolenic acid metabolism	4.66E-08
Tropane, piperidine and pyridine alkaloid biosynthesis	1.50E-06
Glucosinolate biosynthesis	5.73E-06
Stilbenoid, diarylheptanoid and gingerol biosynthesis	3.14E-05
Phenylalanine metabolism	4.73E-05
Cysteine and methionine metabolism	0.0002
Phenylalanine, tyrosine and tryptophan biosynthesis	0.0008
Cyanoamino acid metabolism	0.0008
Isoquinoline alkaloid biosynthesis	0.0017
Flavonoid biosynthesis	0.0019
Brassinosteroid biosynthesis	0.0025
Diterpenoid biosynthesis	0.0051
Arginine and proline metabolism	0.01
Tyrosine metabolism	0.0133
Glycine, serine and threonine metabolism	0.016
Galactose metabolism	0.017
Photosynthesis	0.02
Amino sugar and nucleotide sugar metabolism	0.023
Beta-Alanine metabolism	0.025
Plant hormone signal transduction	0.028
Transcription factors	0.028
Sesquiterpenoid and triterpenoid biosynthesis	0.031

**Table 3:** Significantly enriched differentially regulated KEGG pathways in *Xcc* WT inoculated Meiwa kumquat (4 DPI) plants compared to *Xcc pthA4*:Tn5.

KEGG pathway	*p-value*
DNA replication proteins	2.27E-20
Alpha-Linolenic acid metabolism	1.44E-07
Phenylpropanoid biosynthesis	1.53E-07
Cytochrome P450	4.58E-07
Cytoskeleton proteins	1.47E-05
Tropane, piperidine and pyridine alkaloid biosynthesis	6.45E-05
Cysteine and methionine metabolism	0.00033
Plant hormone signal transduction	0.00063
Isoquinoline alkaloid biosynthesis	0.0025
Phenylalanine, tyrosine and tryptophan biosynthesis	0.0026
Cyanoamino acid metabolism	0.0026
Tyrosine metabolism	0.0033
Stilbenoid, diarylheptanoid and gingerol biosynthesis	0.0034
Phenylalanine metabolism	0.0037
Glycosyltransferases	0.0086
Plant-pathogen interaction	0.0158
Flavonoid biosynthesis	0.023
Pentose and glucuronate interconversions	0.031
Galactose metabolism	0.045

**Fig 4:**
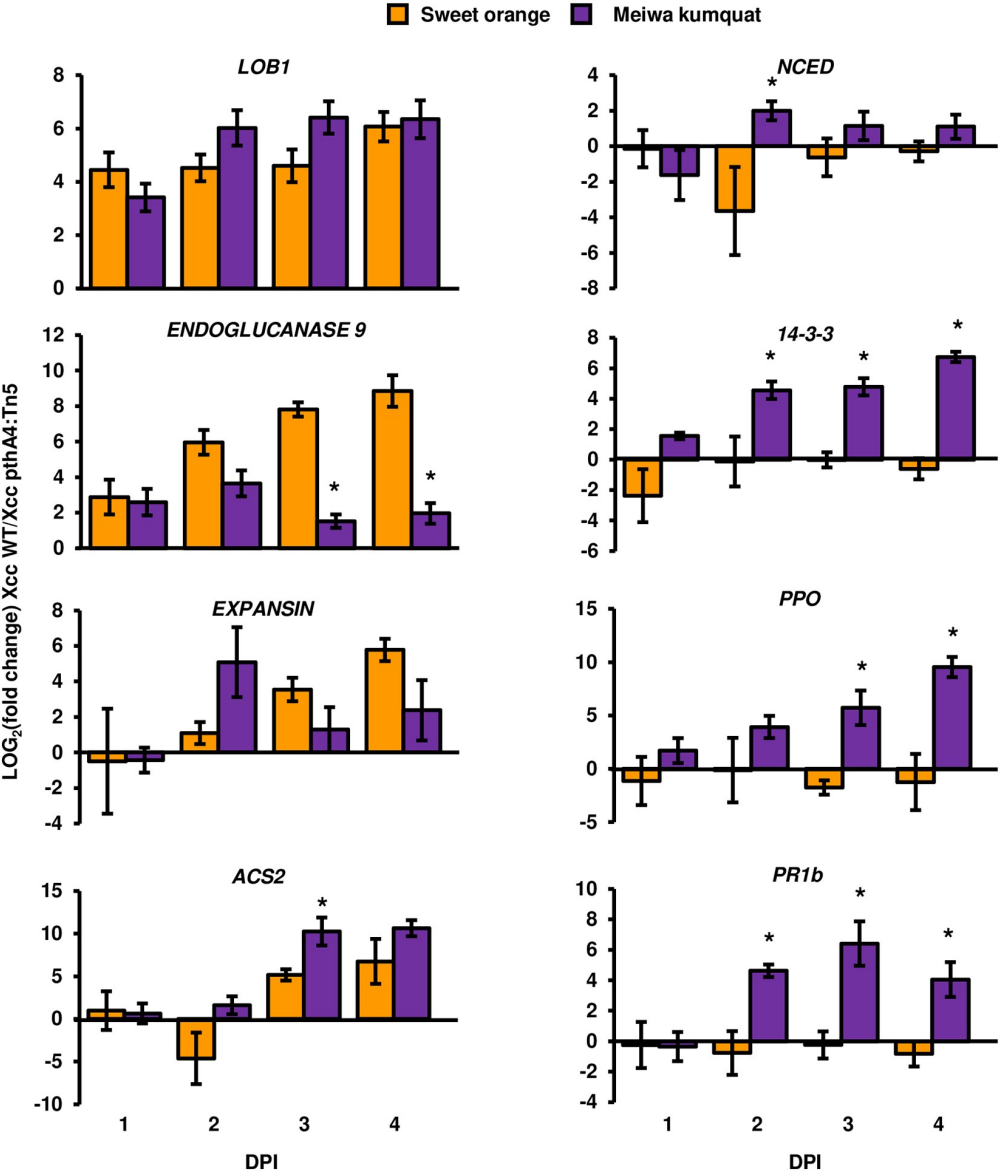
Temporal expression of PthA4-induced genes. *Xcc* cultures (10^8^ CFU/ml) were syringe-infiltrated into Meiwa kumquat or sweet orange leaves. The mRNA abundance (*Xcc* WT-inoculated leaves/*Xcc pthA4*:Tn5-inoculated leaves) of the indicated citrus genes was quantified at one, two, three and four days post inoculation (DPI). The mRNA abundance of *GAPDH* was used for normalization. Values represent the means ± SE of six biological repeats pulled from two independent experimental repeats. Asterisks indicate a significant difference (Student’s *t* test, *p* value < 0.05) in LOG_2_ (fold change) in kumquat compared to sweet orange at the same DPI.

To estimate the PthA4-mediated temporal expression of stress hormone biosynthesis- and immune response-associated genes, we monitored the relative expression (leaves inoculated with *Xcc* WT compared to leaves inoculated with *Xcc pthA4*:Tn5) of eight upregulated genes at one, two, three and four DPI in Meiwa kumquat and sweet orange after inoculation. Three of these genes (i.e., *LOB1*, *EXPANSIN* and *ENDOGLUCANASE 9*) were previously reported to be associated with canker disease susceptibility in sweet orange [29]. The rest of the examined genes encoding stress-associated proteins were shown to be significantly upregulated by PthA4 in Meiwa kumquat in our RNA-seq analysis. The canker susceptibility gene *LOB1* was highly induced by PthA4 in kumquat and sweet orange at one DPI, while the expression of *EXPANSIN* and *ENDOGLUCANASE 9*, which were hypothesized to function downstream of *LOB1*, were induced in the both plants at two DPI, but their induction was much higher in sweet orange than that in kumquat at three and four DPIs(Fig 4). Stress-associated genes were highly induced by PthA4 in kumquat at two or three DPI, while their expression was induced to a lesser extent or not induced at all in sweet orange (Fig 4). In particular, the *NCED* gene associated with the biosynthesis of ABA was induced four-fold in kumquat but not in sweet orange at two DPI, and its expression was slightly reduced in the following days (Fig 4). The expression of *ACS2*, which is associated with the biosynthesis of ethylene, was highly induced in both plants, with higher induction in kumquat than in sweet orange at three and four DPIs (Fig 4). Three immunity-associated genes (*PPO*, *PR1b* and *14-3-3*) [62,63] were uniquely induced in kumquat but not in sweet orange at two DPI, and their relative expression levels remained above 50-fold in the following days (Fig 4).

### Induction of the known susceptibility gene *LOB1* correlates to elicitation of immune responses and early leaf abscission in Meiwa kumquat

Recognition of TALEs can be facilitated through induction of an executor resistance gene by a specific TALE, which depends on the TALE unique repeat array [18]. We examined whether PthA4-induced HR-like phenotype in kumquat is facilitated by the induction of an executor resistance gene or through different physiological responses caused by the induction of *LOB1*, the susceptibility gene targeted by the effector. To identify potential executor resistance genes, we utilized the TAL Effector Nucleotide Targeter 2.0 target prediction tool [44](https://tale-nt.cac.cornell.edu/) and scanned the promoter sequences of all coding genes in Meiwa kumquat and sweet orange for promoters that are potentially targeted by PthA4 (S8 and S9 Tables). We cross-referenced the predicted gene with the PthA4-induced DEGs in kumquat (S3 Table) and determined that *LOB1* was the only induced DEG that was predicted to be targeted by PthA4 in kumquat (S8 Table). Our *in silico* analysis suggests that it is unlikely that the PthA4-mediated immune responses are facilitated by an executor gene in kumquat.

To experimentally test our hypothesis, we used designer TALEs (dTALEs) that contain different central repeat arrays targeting sequences in the promoter region of *LOB1* or its homologs *LOB2* and *LOB3*, which were shown to have a similar function as *LOB1* [43].

We first determined the genomic sequences of *LOB1*, *LOB2* and *LOB3* and their respective promoters in Meiwa kumquat [NCBI accession numbers MT247386 (*LOB1*), MT247387 (*LOB2*) and MT655137 (*LOB3*)]. We identified that the promoters and CDS sequences of *LOB1*, *LOB2* and *LOB3* in Meiwa kumquat and sweet orange share 98%-99% sequence identity (S5 Fig). Protein alignment analysis revealed that kumquat LOB homologs share 99% (for LOB1 and LOB3) or 98% (for LOB2) amino acid sequence identity with sweet orange (S6 Fig). Consistent with the induction of *LOB1* by PthA4 in both plants (Fig 4), the effector binding elements (EBEs) in the promoter regions of *LOB1* in kumquat and sweet orange were identical (S5A Fig).

**Fig 5:**
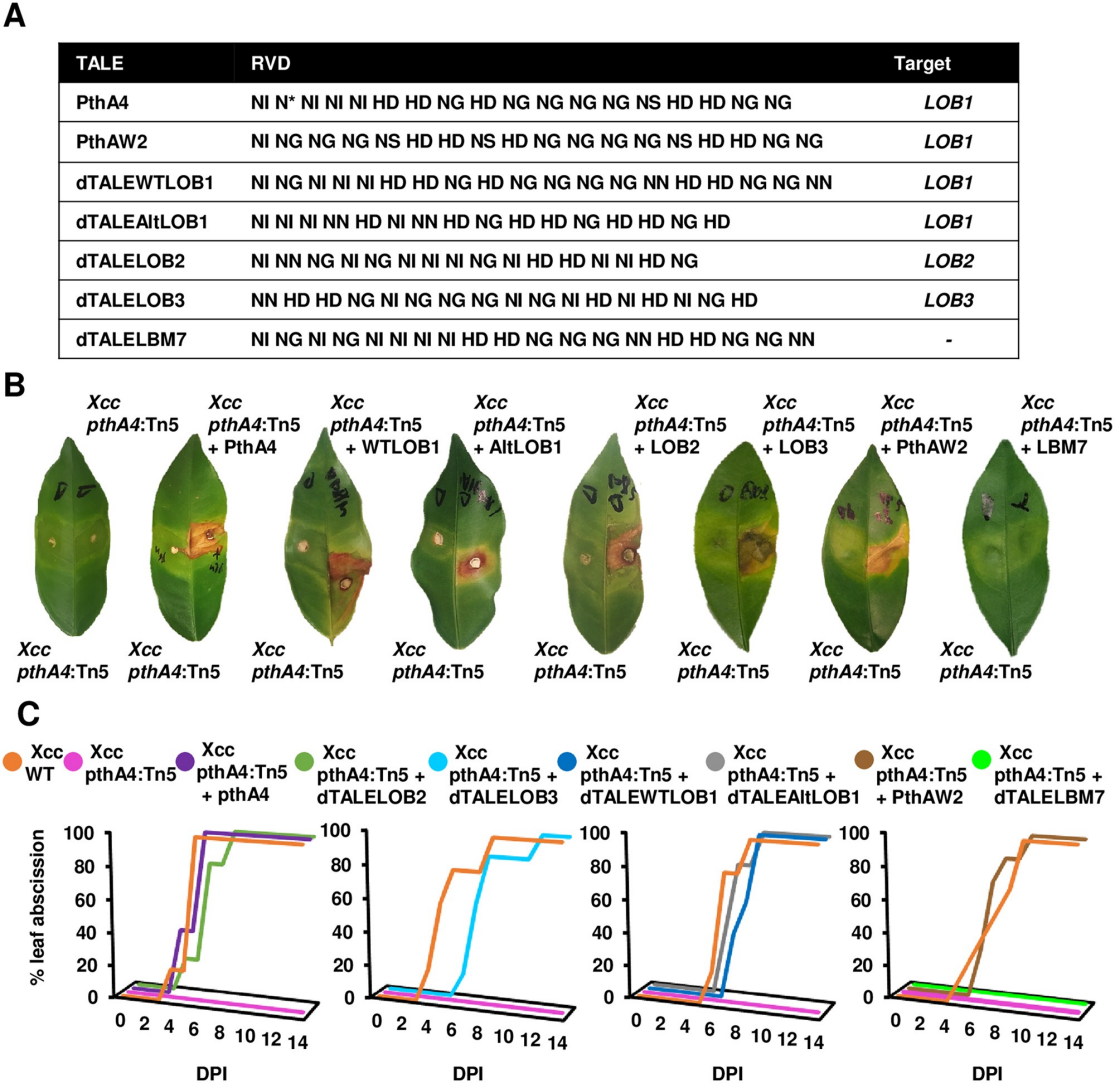
The HR-like phenotype and early leaf abscission are associated with the upregulation of *LOB1*. (**A**) Variable repeat residues (RVD) of the repeat arrays of PthA4 (XACb0065), PthAW2 (XCAW_b00026) and dTALEs used for complementation of *Xcc pthA4*:Tn5. (**B**, **C**) The indicated *Xcc* cultures (10^8^ CFU/ml) were syringe-infiltrated into Meiwa kumquat leaves. (**B**) Pictures were taken at eight days post inoculation (DPI). (**C**) Graphs represent the percentage of abscissed leaves at the indicated DPI (n ≥ 5, maximum of two leaves per plant). Experiments were repeated three times with similar results.

We constructed four dTALEs that target sequences in the promoter regions of the *LOB1* (i.e., dTALEWTLOB1 and dTALEAltLOB1), *LOB2* (i.e., dTALELOB2) or *LOB3* (i.e., dTALELOB3) genes (Fig 5A and S7 Fig). As a positive control, we utilized PthAW2, a TALE from *Xcc*^*AW*^ strain Aw12879 that targets a similar *LOB1* EBE as PthA4 through an alternative repeat array [27]. As a negative control, we manufactured dTALELBM7, a variant of dTALEWTLOB1 that harbors a substitution of five repeats in the TALE central repeat region and is unable to promote canker symptoms in sweet orange (Fig 5A, S8A and S8B Fig).

The TALEs were introduced into *Xcc pthA4*:Tn5, and bacteria were inoculated into kumquat and sweet orange. With the exception of dTALELBM7, the TALEs were able to induce the expression of their predicted target genes in Meiwa kumquat (S8B Fig) and, as expected, enabled the bacteria to induce canker symptoms in sweet orange (S8A Fig). Interestingly, we observed induction of *LOB1* by dTALELOB2 (S8B Fig). Binding prediction analysis did not predict any interaction between this TALE with the promoter region of *LOB1* (S8E and S9E Tables), indicating that *LOB1* is possibly regulated by LOB2 in kumquat.

TALEs targeting either *LOB1*, *LOB2* or *LOB3* restored the capacity of *Xcc pthA4*:Tn5 to induce an HR-like phenotype and early leaf abscission, similar to *pthA4* (Fig 5B and 5C). In addition, defense-associated genes were regulated in kumquat leaves inoculated with *Xcc pthA4*:Tn5 carrying PthA4, PthAW2, dTALEWTLOB1, dTALEAltLOB1, dTALELOB2 or dTALELOB3, but not dTALELBM7 (S8B Fig). We monitored bacterial growth of *Xcc pthA4*:Tn5 carrying dTALEs in kumquat and found that strains carrying PthA4, PthAW2 and dTALEWTLOB1 displayed reduced growth compared to *Xcc pthA4*:Tn5, while the bacterial populations of the rest of the strains were similar (S8C Fig).

Taken together, our data suggest that PthA4-mediated elicitation of immune responses and early leaf abscission in kumquat occurs via induction of *LOB1* and is less likely to be mediated through TALE recognition by a putative executor resistance gene. LOB1 seems to play a dual role in the *Xcc*-kumquat interaction by acting as an S protein resulting in hypertrophy and hyperplasia symptoms in the early stages but as a potential activator of immune responses in the late stages.

### Prediction of DNA binding motifs in the promoters of PthA4-induced genes in Meiwa kumquat

We hypothesize that the altered response of Meiwa kumquat to *LOB1* induction occurs partially because LOB1 regulates a different set of genes compared to sweet orange. We therefore screened the promoter regions of all PthA4-induced genes for potential DNA binding motifs unique to Meiwa kumquat promoters. We initially scanned the promoters for the occurrence of the LBD binding motif GCGGCG [64] but failed to identify significant enrichment of the motif compared to the promoters of the rest of the coding genes in Meiwa kumquat. Next, we performed motif enrichment analysis using MEME suite [65]. Our analysis identified two 6 bp motifs that were significantly enriched (Fisher’s exact test. p-value < 0.01) in the promoters of PthA4-induced genes in kumquat: the CACGTG G-box motif, which was reported to be bound by numerous plant basic helix-loop-helix (bHLH) and basic Leu zipper (bZIP) transcription factors [66], and the GGSCCC motif (S9 Fig). We next examined whether these motifs were also present in the promoter region of the gene homologs in sweet orange and found that these elements were enriched in sweet orange as well (S9 Fig). This suggests that the differential occurrence of these motifs in the promoter elements of genes whose upregulation is mediated by LOB1 cannot explain the differential response of Meiwa kumquat to *LOB1* induction.

### ABA and ethylene are involved in early leaf abscission in Meiwa kumquat in response to *Xcc*

Our RNA-seq analysis revealed that PthA4 promotes the expression of multiple stress-associated genes in kumquat. In particular, several ABA-responsive gene families, such as *HVA22*, *EARLY RESPONSIVE to DEHYDRATION* (*ERD*) and *HEAT SHOCK TRANSCRIPTION FACTOR A2* (*HSTFA2* [67–69]), were upregulated by PthA4 at four DPI (S4E Fig).

ABA and ethylene have been suggested to be involved in abscission in citrus, and ABA was reported to stimulate the transcription of *1-aminocyclopropane-1-carboxylic acid* synthase (ACS) [70–72], which catalyzes the synthesis of ACC, a precursor for ethylene. We investigated whether ABA and ethylene are involved in early leaf abscission in Meiwa kumquat in response to *Xcc*.

**Fig 6:**
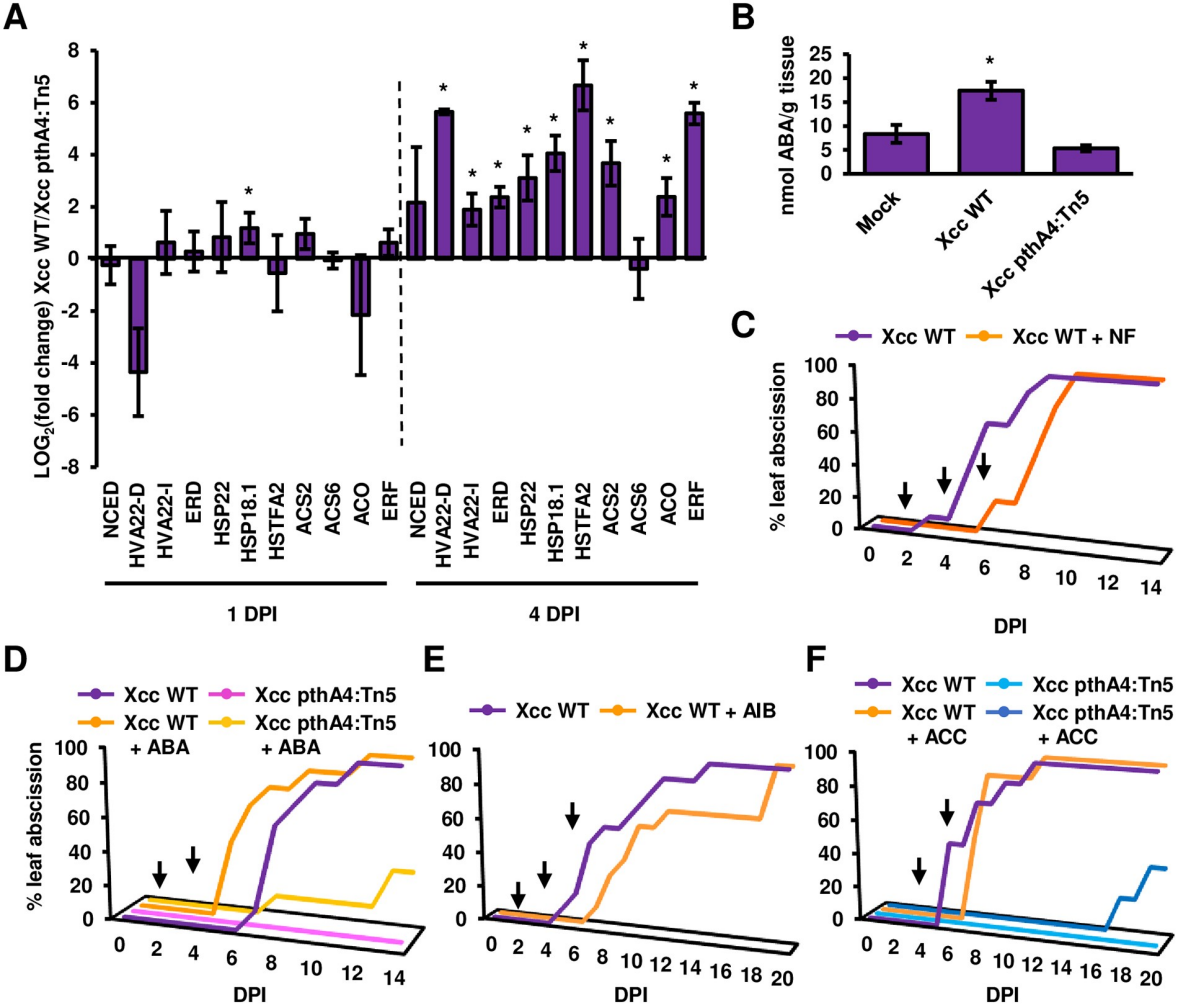
ABA and ethylene contribute to *Xcc*-mediated early leaf abscission. The indicated *Xcc* cultures (10^8^ CFU/ml) or 10 mM MgCl_2_ (mock) were syringe-infiltrated into Meiwa kumquat leaves. (**A**) The mRNA abundance (*Xcc* WT-inoculated leaves/*Xcc pthA4*:Tn5-inoculated leaves) of the indicated citrus genes was quantified at one and four days post inoculation (DPI). The mRNA abundance of *GAPDH* was used for normalization. Values represent the means ± SE of three independent biological repeats. Asterisks indicate a significant difference (Student’s *t* test, *p* value < 0.05) relative to leaves inoculated with *Xcc pthA4*:Tn5 at the same DPI. Experiments were repeated twice with similar results. (**B**) Total ABA was quantified in *Xcc*-infected or mock-treated kumquat leaves at four DPI. Values represent the means ± SE of twelve independent biological samples pulled from three separate experimental repeats. Asterisks indicate a significant difference (Student’s *t* test, *p* value < 0.05) compared to mock-treated leaves. (**C**-**F**) Plants were treated with either 1 mM norflurazon (+ NF) (**C**, n = 10), 0.5 mM abscisic acid (+ ABA) (**D**, n = 8), 10 mM α-aminoisobutyric acid (+ AIB) (**E**, n = 10), 0.1 mM 1-aminocyclopropane-1-carboxylic acid (+ ACC) (**F**, n = 8 for *Xcc* WT treatments, n = 5 for *Xcc pthA4*:Tn5 treatments) or water as a control (**C**-**F**). NF and AIB were applied at two, four and six DPI. ABA and ACC were applied at four and six DPI. Graphs represent the percentage of abscissed leaves at the indicated DPI. Arrows represent the time of foliar spray applications. Experiments were repeated three times with similar results.

The transcriptional expression of ABA- and ethylene-associated genes was monitored in kumquat leaves inoculated with *Xcc* WT or *Xcc* pthA4:Tn5 at one and four DPI. All the selected genes in the ABA and ethylene pathways, with the exception of *NCED* and *ACS6*, were significantly upregulated in kumquat leaves inoculated with *Xcc* WT at four DPI compared to leaves inoculated with *Xcc pthA4*:Tn5 (Fig 6A). In addition, we quantified the ABA levels in kumquat leaves inoculated with *Xcc* at four DPI and observed that ABA levels were higher in leaves inoculated with *Xcc* WT compared to those inoculated with *Xcc pthA4*:Tn5 and mock treatment (Fig 6B).

To further elucidate the role of ABA and ethylene in the induction of the HR-like phenotype and leaf abscission in kumquat, *Xcc*-inoculated kumquat plants were exogenously treated with ABA, ACC, the ABA biosynthesis inhibitor norflurazon (NF) [73] or the ethylene biosynthesis inhibitor α-aminoisobutyric acid (AIB) [74]. None of the exogenous treatments significantly affected the kinetics of cell death development in kumquat leaves inoculated with either *Xcc* WT or *Xcc pthA4*:Tn5 (S10 Fig).

The application of NF to kumquat plants inoculated with *Xcc* delayed *Xcc*-mediated leaf abscission, suggesting that this process is mediated by ABA accumulation (Fig 6C). Accordingly, the application of ABA resulted in earlier leaf abscission in kumquat leaves inoculated with *Xcc* WT (Fig 6D). ABA only partially restored early leaf abscission when added to kumquat leaves infected with *Xcc pthA4*:Tn5 (Fig 6D).

Exogenous application of AIB delayed *Xcc*-induced leaf abscission in kumquat (Fig 6E), indicating that ethylene emission plays a role in *Xcc*-mediated leaf abscission. However, application of ACC did not significantly affect leaf abscission induced by *Xcc* WT in kumquat (Fig 6F). We occasionally observed that ACC induced leaf abscission in kumquat leaves inoculated with *Xcc pthA4*:Tn5 (Fig 6F). This effect was not consistent between biological repeats.

The application of ACC or ABA did not induce any leaf abscission in mock-treated kumquat leaves at 20 DPI, suggesting that additional factors are required to induce early leaf abscission.

## Discussion

Resistance or tolerance to invading pathogens in closely related wild relatives of commercial susceptible crops has been reported on numerous occasions, but was lost during domestication owing to the loss of resistance loci [75]. Kumquats share high genome sequence homology to commercial mandarin hybrids [76,77] and were shown to have durable field resistance to *Xcc* [35]. Our study suggests that this durable resistance to *Xcc* is achieved by multiple mechanisms. First, *Xcc* induces immune responses in kumquats by promoting ion leakage and SA accumulation and inducing defense-related genes [5]. Second, *Xcc* infection of kumquats causes early leaf abscission. Leaf shedding is usually considered a disease effect occurring at the late stage of the infection cycle, and pathogens surviving in the fallen leaves act as the future inoculum. However, the early leaf shedding caused by *Xcc* in kumquats potentially inhibits or reduces further pathogen spread within the plant by removing the infected tissue. Early leaf shedding also prevents the semi-biotrophic *Xcc* from reaching high titers in leaves. Leaf abscission was reported to be induced in response to both biotic and abiotic stress and was hypothesized to restrict further pathogen spread when other immune responses fail to inhibit colonization [78,79]. It was recently shown that shedding of Arabidopsis cauline leaves in response to *Pseudomonas syringe* was associated with host defense signaling, and this response was shown to be dependent on the pathogen T3SS [79], indicating that such responses are actively induced by the plant to counter pathogen attacks. The early leaf shedding caused by *Xcc* in kumquat involves both ABA and ethylene, which is consistent with previous findings that both hormones are important for abscission in citrus [70].

We determined that kumquat immune responses are dependent on the TAL effector (TALE) PthA4. PthA4 induces the expression of *LOB1* in kumquat, which leads to the short-lived development of hypertrophy and hyperplasia by inducing similar genes (e.g., *EXPANSIN* and *ENDOGLUCANASE 9*) as in sweet orange, but at much lower levels. However, the HR-like phenotype in kumquat tissue developed a day or two days after the initial appearance of symptoms, and further disease development was inhibited. TALEs are pathogenicity determinants in numerous *Xanthomonas* species that activate host-specific susceptibility (S) genes to promote disease [7]. Conversely, TALEs induce immune responses leading to incompatible interactions [80–83]. TALE-mediated activation of immune responses in resistant plants can occur by direct induction of the executor resistance (*R*) gene [18] or by recognition of TALEs by NB-LRR-resistance proteins [15,16]. We initially hypothesized that PthA4 induces the expression of an executor *R* gene in kumquat and therefore utilized designer TALEs that target different EBEs in the promoters of *LOB1*, *LOB2* or *LOB3* in hope that these TALEs will bypass any potential executor *R* gene-mediated immune responses. Unexpectedly, the *LOB1*/*LOB2/LOB3*-targeting TALEs still induced immune responses in kumquat, leading us to conclude that PthA4-mediated immune responses occur downstream of the induction of the canker S gene. Our data are the first to provide evidence for such indirect immune recognition of TALEs, and the molecular mechanism of resistance requires further exploration. Such unique immune recognition likely supports the durability of kumquat resistance in the field since the *LOB1* regulon is required for disease symptoms [84].

In addition to *pthA4*, kumquat leaves inoculated with the *Xcc*Δ*xps* mutant strain displayed delayed HR-like phenotype symptoms and leaf abscission. The *xps* mutant was not able to colonize kumquat leaves to the same extent as the wild-type strain. Therefore, we cannot definitively conclude that the reduction in kumquat defense responses is indeed mediated by the *xps* T2SS or that pathogen recognition requires a bacterial population threshold that is not reached by *Xcc*Δ*xps*. Nevertheless, hydrolases secreted by *Xanthomonas xps* T2SS were shown to elicit host immune responses in multiple pathosystems [85,86]. It remains to be determined whether the hydrolases secreted by *xps* T2SS promote the release of DAMPs [3] that trigger immune responses in kumquat. Interestingly, the expression of many plant cell wall-degrading enzymes, such as endoglucanases, pectinases and expansions, is induced by *pthA4* in sweet orange and kumquat [29](S6 Table). We reasoned that cell wall damage induced by the combined effect of the secreted hydrolases derived from both the plant host and the bacteria promotes immune responses in kumquat through recognition by an unknown receptor [87]. Further study is needed to test this hypothesis.

The transcriptional responses of kumquat to PthA4 display high overlap with the transcriptional response to PthA4 in sweet orange [29] but also harbor some unique features. Many genes that are required for the expansion and proliferation of cells are induced in both plants. However, stress-associated genes are enriched in kumquat, and the expression of multiple biotic and abiotic stress markers was significantly elevated in kumquat by PthA4 compared to sweet orange. The temporal analysis showed that these responses initially appeared simultaneously during early infection, while stress responses became more dominant at the later stages. Such stress responses are accompanied by the accumulation of stress hormones, such as ABA, ethylene and SA, that were reported to contribute to leaf abscission [70,79], which is associated with kumquat immunity to *Xcc*. Whether cell proliferation and stress response are interconnected or independent of each other remains unknown. It is possible that kumquats harbor altered physiological responses to excess cell wall damage or to rapid excessive cell proliferation induced by PthA4 compared to susceptible varieties.

Alternatively, induction of the immune response could occur in parallel to canker development and is more directly linked to the LOB1 protein or its activity. One possibility is that LOB1 directly induces the expression of genes that promote localized cell death in a similar manner to immune responses promoted through an executor *R* gene. Another possibility is that the robust accumulation of LOB1 is directly sensed by an immune receptor. Further study is required to decipher the molecular mechanisms underlying this unique system. Overall, our data suggest that the most plausible scenario is that LOB1 acts as an activator of defense responses in the kumquat-*Xcc* interaction that corresponds to the dominant pathogenicity gene *pthA4* and homologues in *Xcc* populations, although we cannot totally rule out other possibilities. In this scenario, the requirement of *LOB1* induction for *Xcc* infection and downstream immune activation by LOB1 pose an insurmountable challenge for the pathogen to overcome, contributing to the durable resistance of kumquat to *Xcc*.

In conclusion, our findings unraveled a unique mechanism of resistance to bacteria that is activated downstream of S gene induction. We suggest that this unique feature can explain the durability of kumquat resistance to canker in the field since immune responses are induced downstream of *LOB1*, which is required for symptom development.

## Supporting information

S1 Fig Symptom development in *Xcc*-inoculated Meiwa kumquat. *Xcc* cultures (10^8^ CFU/ml) were syringe-infiltrated into Meiwa kumquat leaves. A representative infected leaf was photographed six days post inoculation. The enlarged area depicts symptoms and cell death in the abaxial leaf surface.
(PDF)

S2 Fig Contribution of *xopE1* and *xps* T2SS to *Xcc*-mediated symptoms in Meiwa kumquat. The indicated *Xcc* cultures (10^8^ CFU/ml for **A** and **B**; 10^6^ CFU/ml for **C**) or 10 mM MgCl_2_ (mock) were syringe-infiltrated into Meiwa kumquat leaves. (**A**) Infected leaves were photographed eight days post inoculation (DPI). (**B**) Graphs represent the percentage of abscissed leaves at the indicated DPI. n = 10 for leaves inoculated with *Xcc ΔxopE1* (maximum three leaves per plant); n = 11 for leaves inoculated with *Xcc Δxps* (maximum three leaves per plant). n = 3 for mock-treated leaves (one leaf per plant). (**C**) *Xcc* bacterial populations in leaves were quantified at the indicated DPI. Values represent the means ± SE of three independent leaves taken from different plants. Asterisks indicate samples that were significantly different (Student’s t-test, *p value* < 0.05) compared to leaves inoculated with *Xcc* WT at the same DPI. All depicted experiments were repeated at least three times with similar results.
(PDF)

S3 Fig Summary of RNA-seq analysis. Meiwa kumquat leaves were inoculated with *Xcc* WT and *Xcc pthA4*:Tn5 cultures (10^8^ CFU/ml), and total RNA was extracted one and four days after inoculation. Three independent kumquat samples inoculated with *Xcc* WT or *Xcc pthA4*:Tn5 were sent for RNA-seq analysis. (**A**) Distance analysis of independent samples at one and four days after inoculation. (**B**) Venn diagrams depict the number of overlapping DEGs (FDR *P value* < 0.05, cutoff was set at three-fold change) between day one and day four.
(PDF)

S4 Fig PthA4 induced differential expression of representative gene families in Meiwa kumquat. Heat map tables represent LOG_2_ fold expression (*Xcc* 306/*Xcc pthA4*:Tn5) at day one and day four; the tables are marked in a color gradient ranging from LOG_2_ = -4 (marked in yellow) to LOG_2_ = 4 (marked in purple). Color gradient scales are found on the bottoms of the heat map tables. (**A**) Defense response marker gene families: *POLYPHENOL OXIDASE* (*PPO*), *PATHOGENASIS-RELATED PROTEIN 1* (*PR1*), *PATHOGENASIS-RELATED PROTEIN 5* (*PR5*), *PATHOGENASIS-RELATED PROTEIN 10* (*PR10*) and *SNAKIN* (*SNK*). (**B**) Papain-like cysteine protease coding genes. (**C**) Abscisic acid (ABA) biosynthesis gene families: xanthine dehydrogenase (*ABA3*), short-chain alcohol dehydrogenase (*ABA2*) and *9-CIS-EPOXYCAROTENOID DIOXYGENASE* (*NCED*). (**D**) Ethylene biosynthesis gene families: *S-ADENOSYL-L-METHIONINE SYNTHETASE* (*SAMS*), *1*-*AMINOCYCLOPROPANE-1-CARBOXYLIC ACID SYNTHASE* (*ACS*) and *1-AMINOCYCLOPROPANE-1-CARBOXYLIC ACID OXIDASE* (*ACO*). (**E**) ABA/abiotic stress response marker gene families: *HVA22*, *EARLY RESPONSIVE to DEHYDRATION* (*ERD*) and *HEAT SHOCK TRANSCRIPTION FACTOR A2* (*HSTFA2*).
(PDF)

S5 Fig DNA sequence alignment of *LOB1*, *LOB2* and *LOB3* in Meiwa kumquat and sweet orange. Sequence alignment comparing the regions containing promoter, coding region and intron of *LOB1*, *LOB2* and *LOB3* in Meiwa kumquat and sweet orange. Analysis was conducted with the Clustal Omega Multiple Sequence Alignment tool under default settings (https://www.ebi.ec.uk/Tools/msa/clustalo/). All coding regions are labeled in green. (**A**) Alignment of the *LOB1* gene in Meiwa kumquat (Fc, NCBI accession num’ MT247386) and sweet orange (Cs, chromosome 7 28355982–28359437). The effector binding element PthA4 is labeled in purple. (**B**) Alignment of the *LOB2* gene in Meiwa kumquat (Fc, NCBI accession num’ MT247387) and sweet orange (Cs, chromosome 7 28339099–28340353). (**C**) Alignment of the *LOB3* gene in Meiwa kumquat (Fc, NCBI accession num’ MT655137) and sweet orange (Cs, chromosome 8 20045688–20047208).
(PDF)

S6 Fig Protein sequence alignment of *LOB1*, *LOB2* and *LOB3* in Meiwa kumquat and sweet orange. Protein sequence alignment comparing LOB1, LOB2 and LOB3 in Meiwa kumquat and sweet orange. Analysis was conducted with the Clustal Omega Multiple Sequence Alignment tool under default settings (https://www.ebi.ec.uk/Tools/msa/clustalo/). (**A**) Alignment of the LOB1 homologs from Meiwa kumquat (Fc, NCBI accession num’ MT247386) and sweet orange (Cs, Cs7g27640). (**B**) Alignment of the LOB2 homologs from Meiwa kumquat (Fc, NCBI accession num’ MT247387) and sweet orange (Cs, Cs7g27620). (**C**) Alignment of the LOB3 homologs from Meiwa kumquat (Fc, NCBI accession num’ MT655137) and sweet orange (Cs, Cs8g17160).
(PDF)

S7 Fig Effector binding elements of *LOB1*-, *LOB2*- and *LOB3*-targeting dTALEs in Meiwa kumquat. DNA sequences represent the promoter regions of *LOB1* (**A**, NCBI accession num’ MT247386), *LOB2* (**B**, NCBI accession num’ MT247387) and *LOB3* (**C**, NCBI accession num’ MT655137) in Meiwa kumquat. Sequences mark the start codons of *LOB1*, *LOB2* and *LOB3* (underlined ATG) and TAL effector binding elements (EBEs) of PthA4, PthAW2, dTALEWTLOB1, dTALEAltLOB1, dTALELOB2 and dTALELOB3.
(PDF)

S8 Fig Functional analysis of *LOB1*-, *LOB2*- and *LOB3*-targeting TALEs in Meiwa kumquat. (**A**) Sweet orange leaves were syringe-inoculated (10^8^ CFU/ml) with *Xcc pthA4*:Tn5 harboring plasmids encoding the indicated dTALEs. Pictures were taken eight days after inoculation (DPI). (**B**, **C**) Meiwa kumquat leaves were syringe-inoculated (10^8^ CFU/ml for **B**, 10^6^ CFU/ml for **C**) with *Xcc* WT and *Xcc pthA4*:Tn5 harboring plasmids encoding the indicated dTALEs. (**B**) The mRNA abundance of the indicated genes was quantified at four DPI. The mRNA abundance of *GAPDH* was used for normalization. Values represent the means ± SE of three independent experimental repeats. Asterisks indicate a significant difference (Student’s *t* test, *p* value < 0.05) relative to *Xcc pthA4*:Tn5. (**C**) Bacterial populations were determined at the indicated DPI. Values represent the means ± SE of three to five independent leaves taken from different plants. Asterisks indicate a significant difference (Student’s *t* test, *p* value < 0.05) relative to *Xcc pthA4*:Tn5. Experiments were repeated twice with similar results.
(PDF)

S9 Fig Enrichment of DNA motifs in the promoters of PthA4-upregulated genes in Meiwa kumquat and sweet orange. The regions 1 kb upstream of the transcriptional start site (predicted promoter) of Meiwa kumquat genes that were found to be upregulated by PthA4 were analyzed using the MEME motif discovery feature (http://meme-suite.org/tools/meme). Parameters were set to analyze strands with a 6–10 width cutoff. Bars represent the frequency of the CACGTC and GGSCCC motifs (represented on the top of each graph in sequence logo format) per promoter for all coding genes and genes that were found to be upregulated by PthA4 in Meiwa kumquat and sweet orange. Asterisks indicate a significant difference (Fisher’s exact test, *p* value < 0.01) relative to frequency in all coding genes.
(PDF)

S10 Fig Effects of ABA and ACC treatments on *Xcc*-inoculated Meiwa kumquat leaves. The indicated *Xcc* cultures (10^8^ CFU/ml)) were syringe-infiltrated into Meiwa kumquat leaves. Plants were treated with either 1 mM norflurazon (+ NF), 0.5 mM abscisic acid (+ ABA), 10 mM α-aminoisobutyric acid (+ AIB), 0.1 mM 1-aminocyclopropane-1-carboxylic acid (ACC) or water as a control. NF and AIB were applied at two, four and six days post inoculation (DPI). ABA and ACC were applied at four and six DPI. Leaves were photographed at eight DPI. Experiments were repeated three times with similar results.
(PDF)

S1 Table Bacterial strains and plasmids used in this study. (DOCX)

S2 Table Primers used during this study. (DOCX)

S3 Table Relative gene expression of DEGs in Meiwa kumquat inoculated with Xcc WT compared to Xcc pthA4:Tn5. (XLSX)

S4 Table qRT PCR validation of RNA-seq data at 4 DPI. (XLSX)

S5 Table KEGG classification of DEGs from Meiwa kumquat. (XLSX)

S6 Table GO enrichment of up-regulated DEGs. (XLSX)

S7 Table GO enrichment of down-regulated DEGs. (XLSX)

S8 Table Predicted Meiwa kumquat targets of TALEs used in this study. (XLSX)

S9 Table Predicted sweet orange targets of TALEs used in this study. (XLSX)

S1 File Promoter sequences of Meiwa kumquat. (DOCX)

S2 File Promoter sequences of sweet orange. (DOCX)
