# Supplementary material for: The immunity of Meiwa kumquat against Xanthomonas citri is associated with a known susceptibility gene induced by a transcription activator-like effector
Source: PLoS Pathog. 2020 Sep 15;16(9):e1008886. doi: 10.1371/journal.ppat.1008886 (PMC7518600; doi:10.1371/journal.ppat.1008886)
Supplement: S1 Fig — Xcc cultures (108 CFU/ml) were syringe-infiltrated into Meiwa kumquat leaves. A representative infected leaf was photographed six days post inoculation. The enlarged area depicts symptoms and cell death in the abaxial leaf surface. (PDF) [file ppat.1008886.s001.pdf]

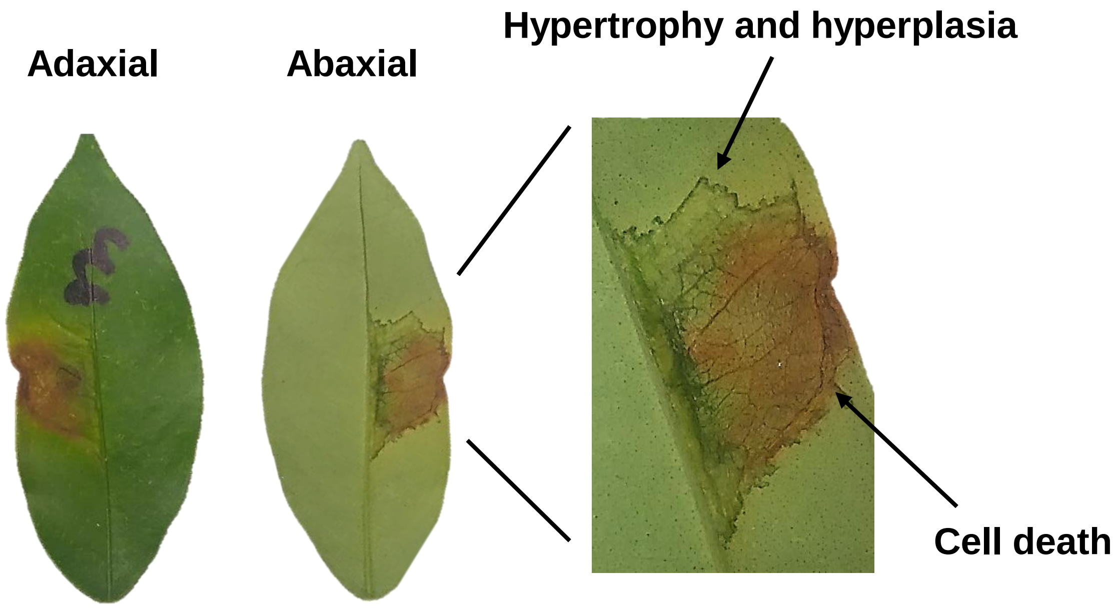

S1 Fig. Symptom development in *Xcc*-inoculated Meiwa kumquat. *Xcc* cultures (10^8 CFU/ml) were syringe-infiltrated into Meiwa kumquat leaves. A representative infected leaf was photographed six days post inoculation. The enlarged area depicts symptoms and cell death in the abaxial leaf surface.
