# Supplementary material for: The immunity of Meiwa kumquat against Xanthomonas citri is associated with a known susceptibility gene induced by a transcription activator-like effector
Source: PLoS Pathog. 2020 Sep 15;16(9):e1008886. doi: 10.1371/journal.ppat.1008886 (PMC7518600; doi:10.1371/journal.ppat.1008886)
Supplement: S2 Fig — The indicated Xcc cultures (108 CFU/ml for A and B; 106 CFU/ml for C) or 10 mM MgCl2 (mock) were syringe-infiltrated into Meiwa kumquat leaves. (A) Infected leaves were photographed eight days post inoculation (DPI). (B) Graphs represent the percentage of abscissed leaves at the indicated DPI. n = 10 for leaves inoculated with Xcc ΔxopE1 (maximum three leaves per plant); n = 11 for leaves inoculated with Xcc Δxps (maximum three leaves per plant). n = 3 for mock-treated leaves (one leaf per plant). (C) Xcc bacterial populations in leaves were quantified at the indicated DPI. Values represent the means ± SE of three independent leaves taken from different plants. Asterisks indicate samples that were significantly different (Student’s t-test, p value < 0.05) compared to leaves inoculated with Xcc WT at the same DPI. All depicted experiments were repeated at least three times with similar results. (PDF) [file ppat.1008886.s002.pdf]

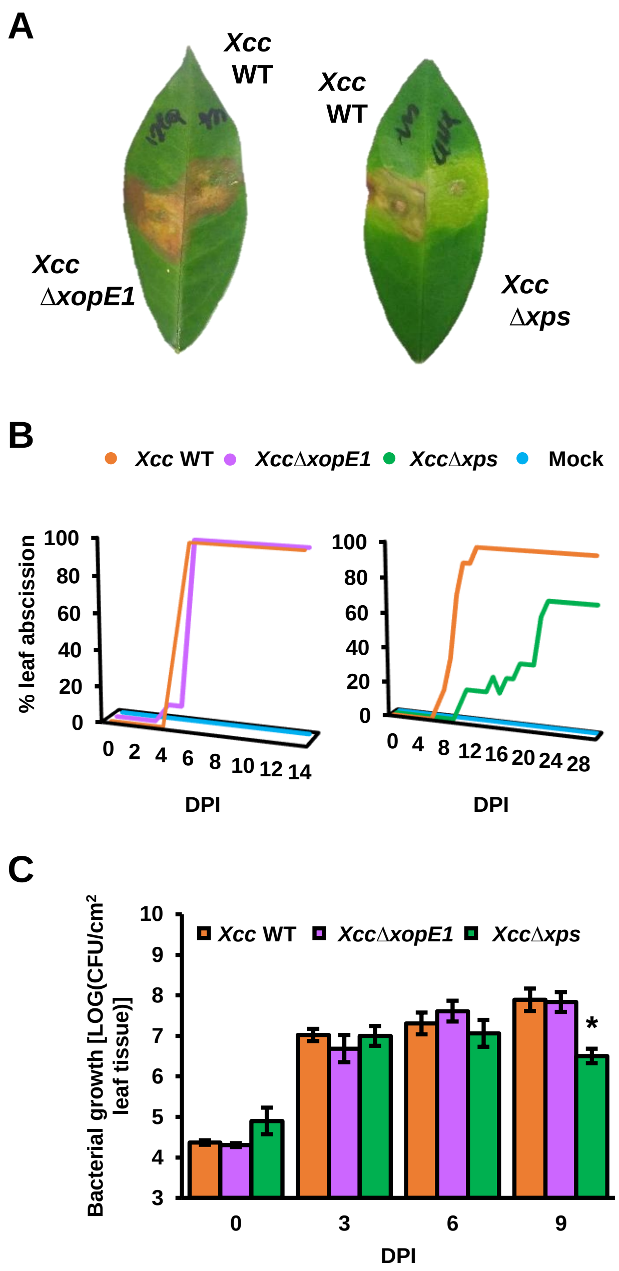


**S2 Fig. Contribution of *xopE1* and *xps* T2SS to *Xcc*-mediated symptoms in Meiwa kumquat**. The indicated *Xcc* cultures ($10^8$ CFU/ml for **A** and **B**; $10^6$ CFU/ml for **C**) or 10 mM $MgCl_2$ (mock) were syringe-infiltrated into Meiwa kumquat leaves. (**A**) Infected leaves were photographed eight days post inoculation (DPI). (**B**) Graphs represent the percentage of abscessed leaves at the indicated DPI. n = 10 for leaves inoculated with *Xcc ΔxopE1* (maximum three leaves per plant); n = 11 for leaves inoculated with *Xcc Δxps* (maximum three leaves per plant). n = 3 for mock-treated leaves (one leaf per plant). (**C**) *Xcc* bacterial populations in leaves were quantified at the indicated DPI. Values represent the means ± SE of three independent leaves taken from different plants. Asterisks indicate samples that were significantly different (Student's t-test, *p value* < 0.05) compared to leaves inoculated with *Xcc* WT at the same DPI. All depicted experiments were repeated at least three times with similar results.
