# Supplementary material for: The immunity of Meiwa kumquat against Xanthomonas citri is associated with a known susceptibility gene induced by a transcription activator-like effector
Source: PLoS Pathog. 2020 Sep 15;16(9):e1008886. doi: 10.1371/journal.ppat.1008886 (PMC7518600; doi:10.1371/journal.ppat.1008886)
Supplement: S3 Fig — Meiwa kumquat leaves were inoculated with Xcc WT and Xcc pthA4:Tn5 cultures (108 CFU/ml), and total RNA was extracted one and four days after inoculation. Three independent kumquat samples inoculated with Xcc WT or Xcc pthA4:Tn5 were sent for RNA-seq analysis. (A) Distance analysis of independent samples at one and four days after inoculation. (B) Venn diagrams depict the number of overlapping DEGs (FDR P value < 0.05, cutoff was set at three-fold change) between day one and day four. (PDF) [file ppat.1008886.s003.pdf]

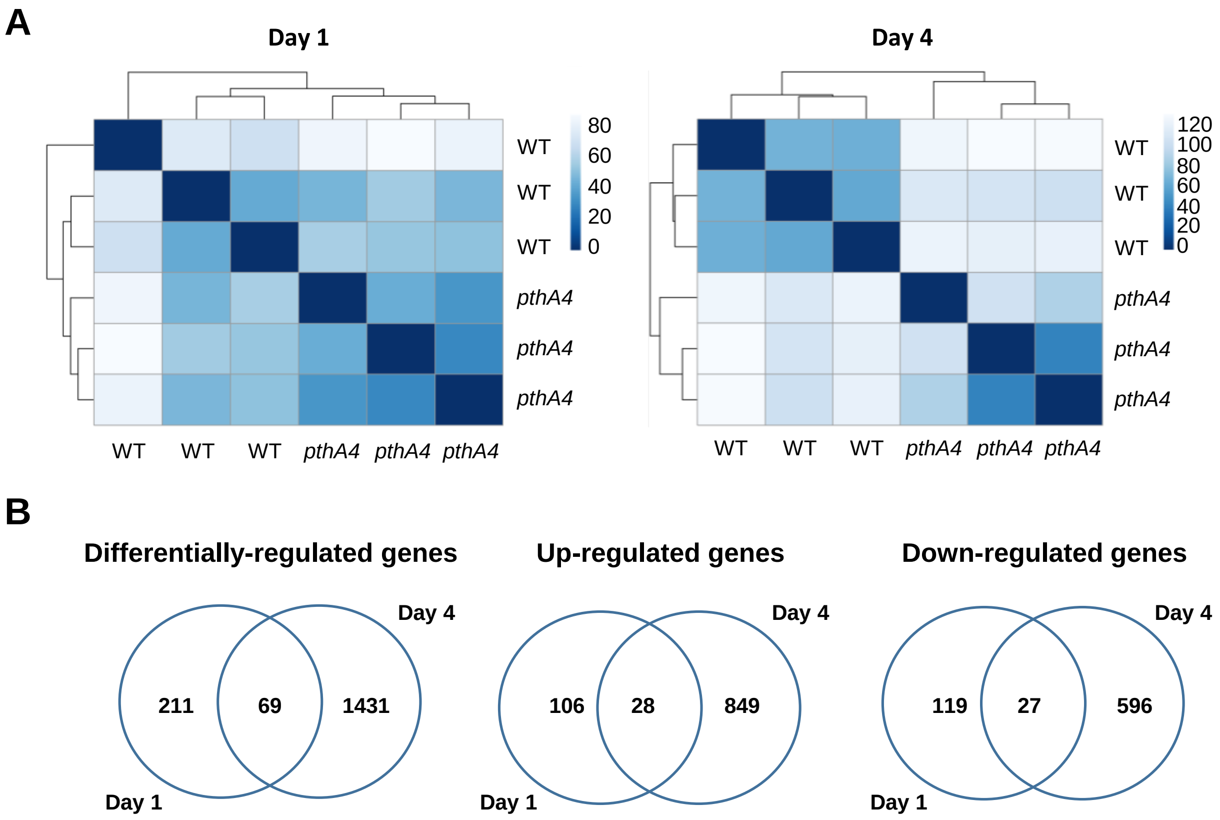


**S3 Fig. Summary of RNA-seq analysis.** Meiwa kumquat leaves were inoculated with *Xcc* WT and *Xcc pthA4*:Tn5 cultures ($10^8$ CFU/ml), and total RNA was extracted one and four days after inoculation. Three independent kumquat samples inoculated with *Xcc* WT or *Xcc pthA4*:Tn5 were sent for RNA-seq analysis. (**A**) Distance analysis of independent samples at one and four days after inoculation. (**B**) Venn diagrams depict the number of overlapping DEGs (FDR *P value* < 0.05, cutoff was set at three-fold change) between day one and day four.
