# Supplementary material for: The immunity of Meiwa kumquat against Xanthomonas citri is associated with a known susceptibility gene induced by a transcription activator-like effector
Source: PLoS Pathog. 2020 Sep 15;16(9):e1008886. doi: 10.1371/journal.ppat.1008886 (PMC7518600; doi:10.1371/journal.ppat.1008886)
Supplement: S4 Fig — Heat map tables represent LOG2 fold expression (Xcc 306/Xcc pthA4:Tn5) at day one and day four; the tables are marked in a color gradient ranging from LOG2 = -4 (marked in yellow) to LOG2 = 4 (marked in purple). Color gradient scales are found on the bottoms of the heat map tables. (A) Defense response marker gene families: POLYPHENOL OXIDASE (PPO), PATHOGENASIS-RELATED PROTEIN 1 (PR1), PATHOGENASIS-RELATED PROTEIN 5 (PR5), PATHOGENASIS-RELATED PROTEIN 10 (PR10) and SNAKIN (SNK). (B) Papain-like cysteine protease coding genes. (C) Abscisic acid (ABA) biosynthesis gene families: xanthine dehydrogenase (ABA3), short-chain alcohol dehydrogenase (ABA2) and 9-CIS-EPOXYCAROTENOID DIOXYGENASE (NCED). (D) Ethylene biosynthesis gene families: S-ADENOSYL-L-METHIONINE SYNTHETASE (SAMS), 1-AMINOCYCLOPROPANE-1-CARBOXYLIC ACID SYNTHASE (ACS) and 1-AMINOCYCLOPROPANE-1-CARBOXYLIC ACID OXIDASE (ACO). (E) ABA/abiotic stress response marker gene families: HVA22, EARLY RESPONSIVE to DEHYDRATION (ERD) and HEAT SHOCK TRANSCRIPTION FACTOR A2 (HSTFA2). (PDF) [file ppat.1008886.s004.pdf]

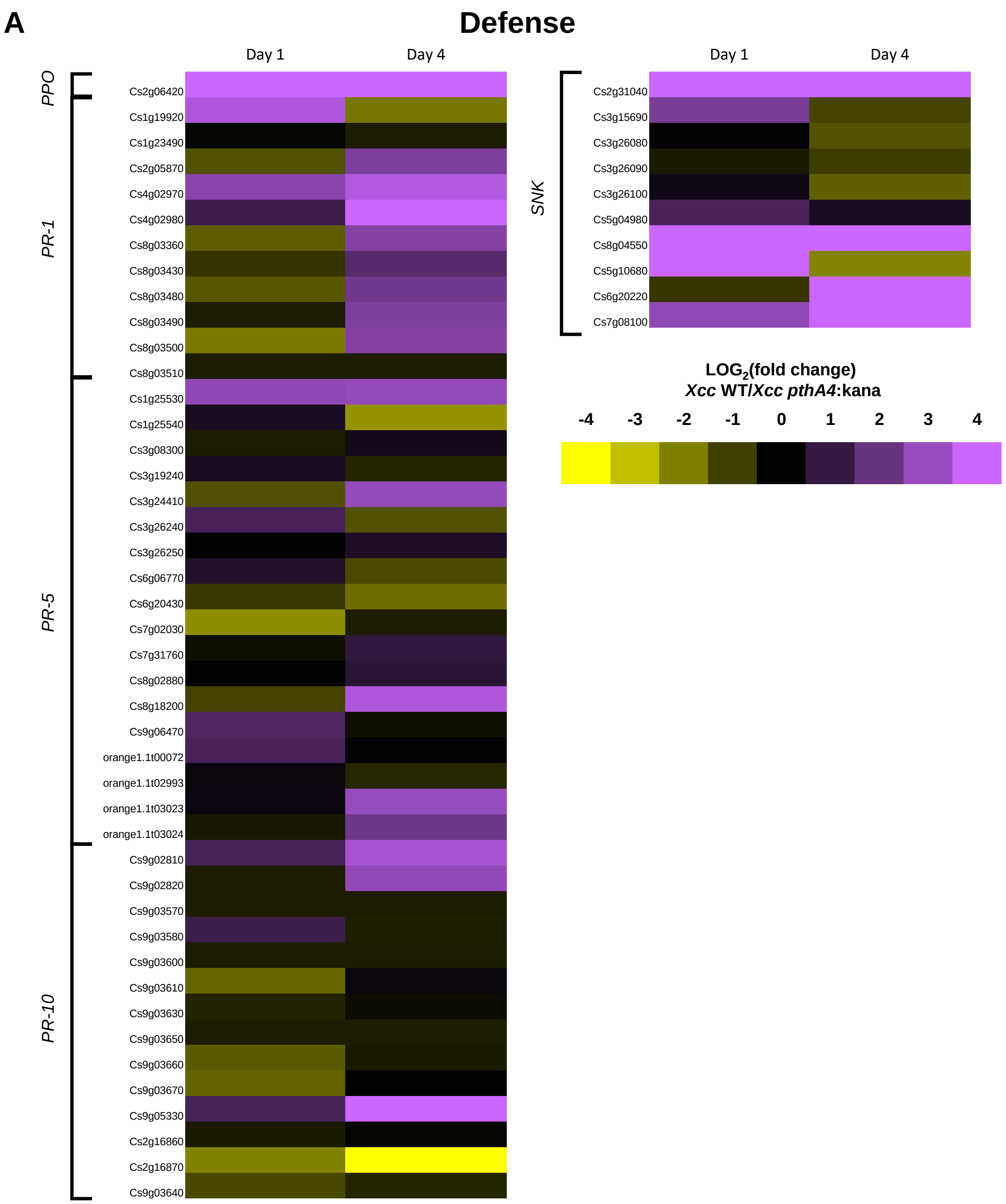
A
Defense
Day 1
Day 4
PPO
PR-1
PR-5
PR-10
Cs2g06420
Cs1g19920
Cs1g23490
Cs2g05870
Cs4g02970
Cs4g02980
Cs8g03360
Cs8g03430
Cs8g03480
Cs8g03490
Cs8g03500
Cs8g03510
Cs1g25530
Cs1g25540
Cs3g08300
Cs3g19240
Cs3g24410
Cs3g26240
Cs3g26250
Cs6g06770
Cs6g20430
Cs7g02030
Cs7g31760
Cs8g02880
Cs8g18200
Cs9g06470
orange1.1t00072
orange1.1t02993
orange1.1t03023
orange1.1t03024
Cs9g02810
Cs9g02820
Cs9g03570
Cs9g03580
Cs9g03600
Cs9g03610
Cs9g03630
Cs9g03650
Cs9g03660
Cs9g03670
Cs9g05330
Cs2g16860
Cs2g16870
Cs9g03640
Day 1
Day 4
SNK
Cs2g31040
Cs3g15690
Cs3g26080
Cs3g26090
Cs3g26100
Cs5g04980
Cs8g04550
Cs5g10680
Cs6g20220
Cs7g08100
LOG2(fold change)
Xcc WT/Xcc pthA4:kana
-4
-3
-2
-1
0
1
2
3
4

## B

# *PAPAIN-LIKE CYSTEINE PROTEASES*

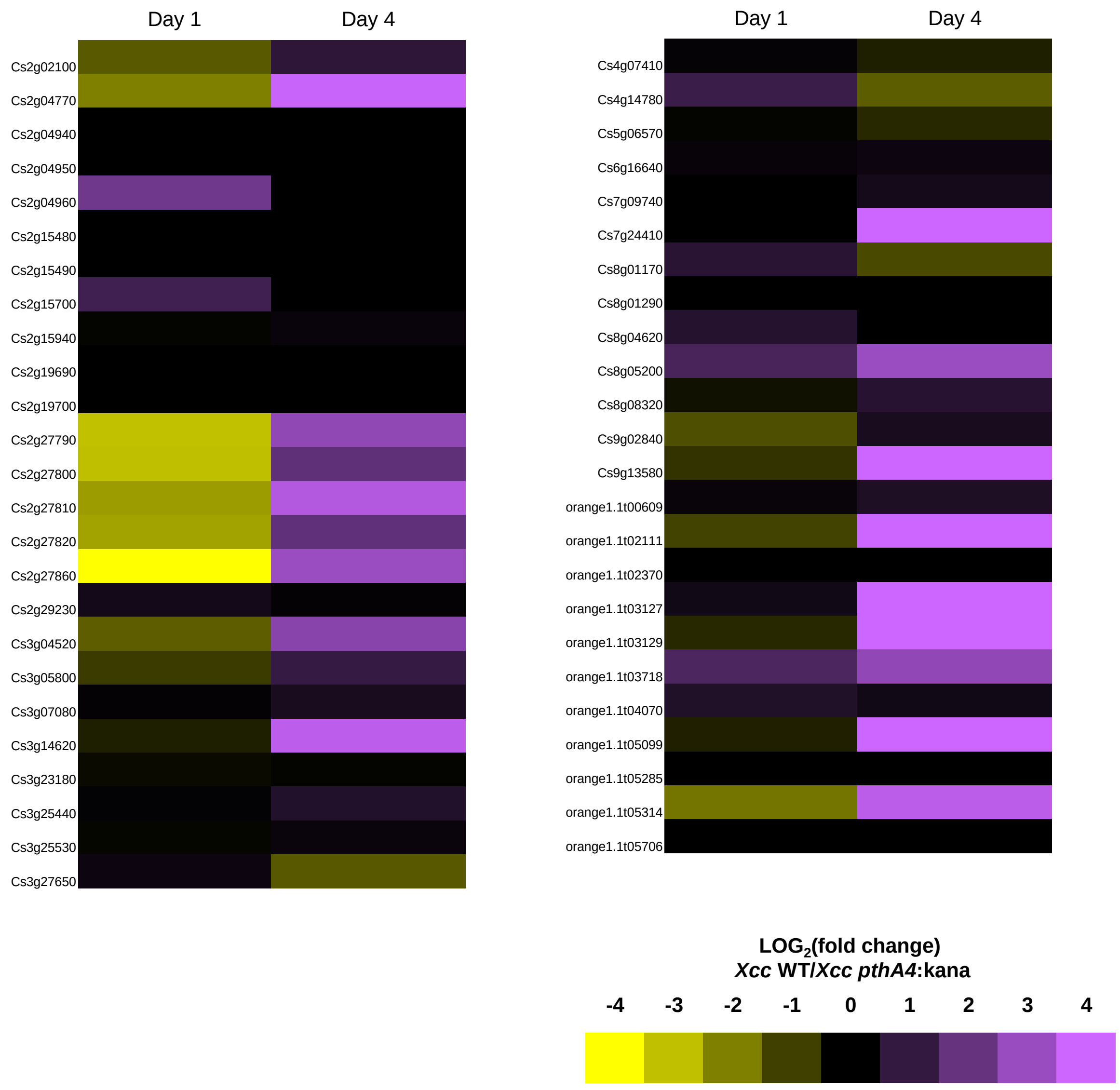

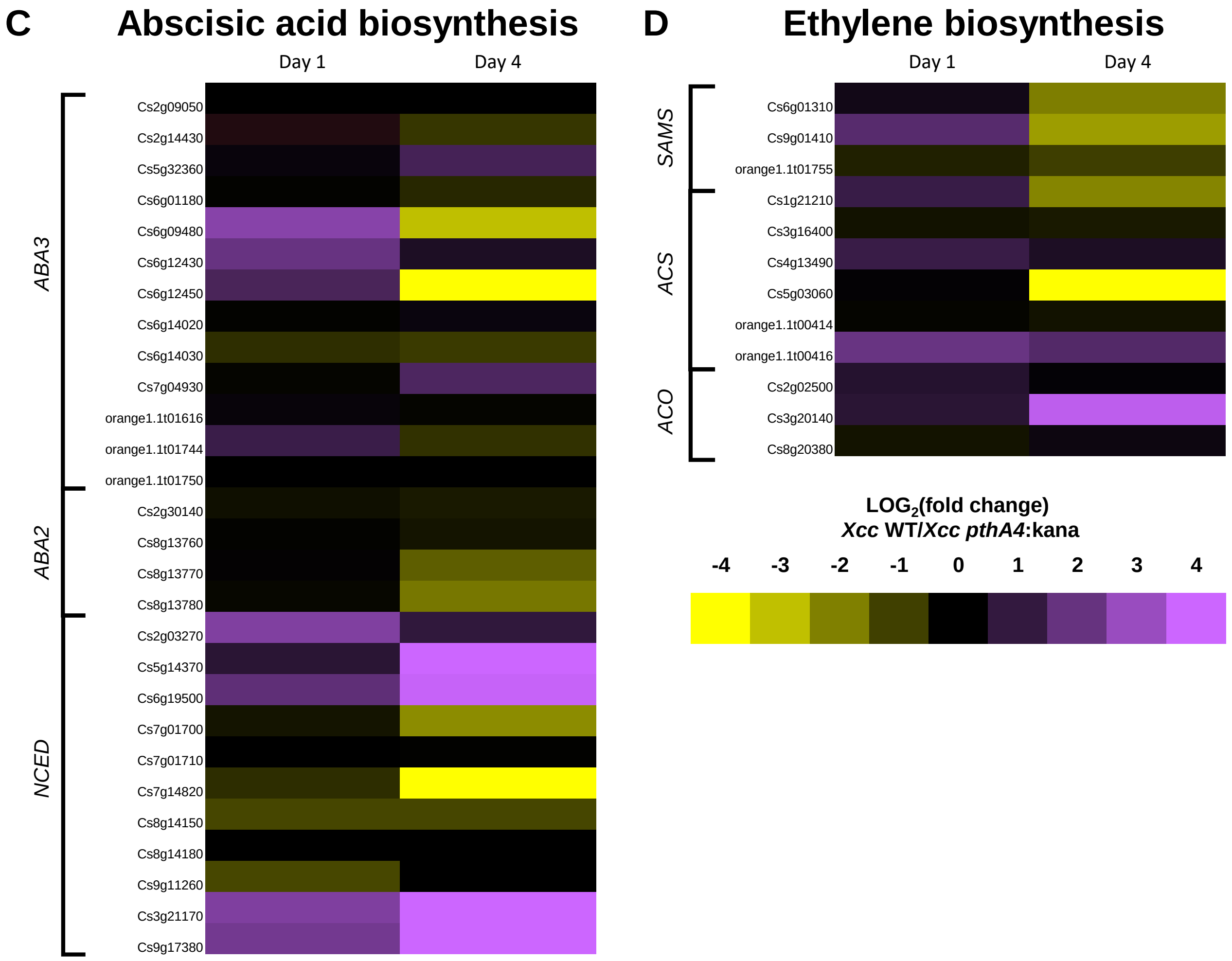
C
Abscisic acid biosynthesis
Day 1
Day 4
ABA3
ABA2
NCED
Cs2g09050
Cs2g14430
Cs5g32360
Cs6g01180
Cs6g09480
Cs6g12430
Cs6g12450
Cs6g14020
Cs6g14030
Cs7g04930
orange1.1t01616
orange1.1t01744
orange1.1t01750
Cs2g30140
Cs8g13760
Cs8g13770
Cs8g13780
Cs2g03270
Cs5g14370
Cs6g19500
Cs7g01700
Cs7g01710
Cs7g14820
Cs8g14150
Cs8g14180
Cs9g11260
Cs3g21170
Cs9g17380
D
Ethylene biosynthesis
Day 1
Day 4
SAMS
ACS
ACO
Cs6g01310
Cs9g01410
orange1.1t01755
Cs1g21210
Cs3g16400
Cs4g13490
Cs5g03060
orange1.1t00414
orange1.1t00416
Cs2g02500
Cs3g20140
Cs8g20380
LOG2(fold change)
Xcc WT/Xcc pthA4:kana
-4 -3 -2 -1 0 1 2 3 4

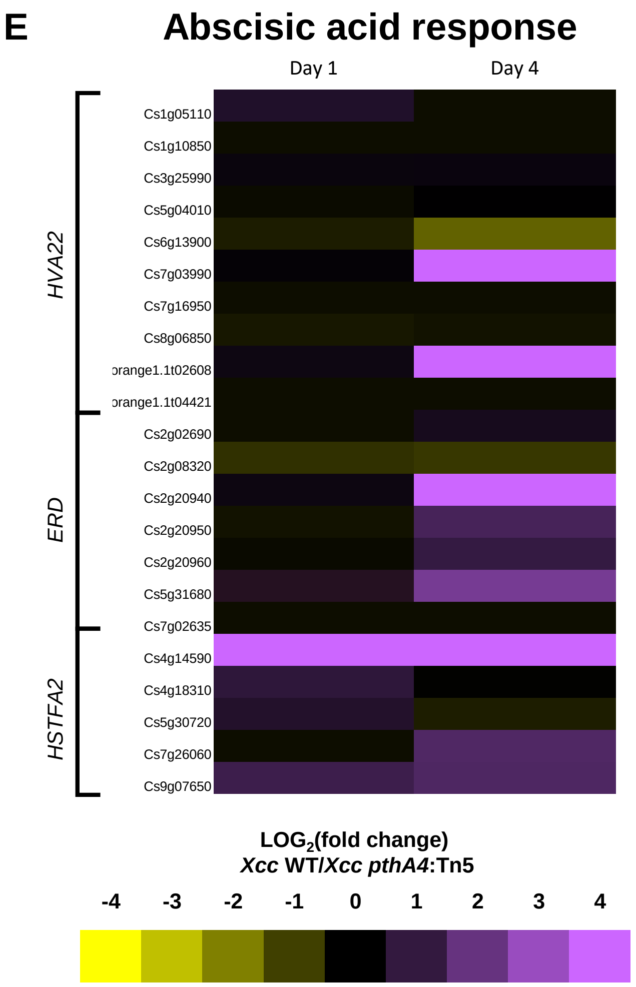


**S4 Fig. PthA4 induced differential expression of representative gene families in Meiwa kumquat**. Heat map tables represent $LOG_2$ fold expression (*Xcc* 306/*Xcc pthA4*:Tn5) at day one and day four; the tables are marked in a color gradient ranging from $LOG_2$ = -4 (marked in yellow) to $LOG_2$ = 4 (marked in purple). Color gradient scales are found on the bottoms of the heat map tables. (**A**) Defense response marker gene families: *POLYPHENOL OXIDASE* (*PPO*), *PATHOGENASIS-RELATED PROTEIN 1* (*PR1*), *PATHOGENASIS-RELATED PROTEIN 5* (*PR5*), *PATHOGENASIS-RELATED PROTEIN 10* (*PR10*) and *SNAKIN* (*SNK*). (**B**) Papain-like cysteine protease coding genes. (**C**) Abscisic acid (ABA) biosynthesis gene families: xanthine dehydrogenase (*ABA3*), short-chain alcohol dehydrogenase (*ABA2*) and *9-CIS-EPOXYCAROTENOID DIOXYGENASE* (*NCED*). (**D**) Ethylene biosynthesis gene families: *S-ADENOSYL-L-METHIONINE SYNTHETASE* (*SAMS*), *1-AMINOCYCLOPROPANE-1-CARBOXYLIC ACID SYNTHASE* (*ACS*) and *1-AMINOCYCLOPROPANE-1-CARBOXYLIC ACID OXIDASE* (*ACO*). (**E**) ABA/abiotic stress response marker gene families: *HVA22*, *EARLY RESPONSIVE to DEHYDRATION* (*ERD*) and *HEAT SHOCK TRANSCRIPTION FACTOR A2* (*HSTFA2*).
