# Supplementary material for: The immunity of Meiwa kumquat against Xanthomonas citri is associated with a known susceptibility gene induced by a transcription activator-like effector
Source: PLoS Pathog. 2020 Sep 15;16(9):e1008886. doi: 10.1371/journal.ppat.1008886 (PMC7518600; doi:10.1371/journal.ppat.1008886)
Supplement: S5 Fig — Sequence alignment comparing the regions containing promoter, coding region and intron of LOB1, LOB2 and LOB3 in Meiwa kumquat and sweet orange. Analysis was conducted with the Clustal Omega Multiple Sequence Alignment tool under default settings (https://www.ebi.ec.uk/Tools/msa/clustalo/). All coding regions are labeled in green. (A) Alignment of the LOB1 gene in Meiwa kumquat (Fc, NCBI accession num’ MT247386) and sweet orange (Cs, chromosome 7 28355982–28359437). The effector binding element PthA4 is labeled in purple. (B) Alignment of the LOB2 gene in Meiwa kumquat (Fc, NCBI accession num’ MT247387) and sweet orange (Cs, chromosome 7 28339099–28340353). (C) Alignment of the LOB3 gene in Meiwa kumquat (Fc, NCBI accession num’ MT655137) and sweet orange (Cs, chromosome 8 20045688–20047208). (PDF) [file ppat.1008886.s005.pdf]

A

```
Fc      AGTAATTCAACCATTTGATTTGCAATCACCGCGGAGGCTTGAAACTTGAACTCACAAATA 60
Cs      AGTAATTCAACCATTTGATTTGCAATCACCGCGGAGGCTTGAAACTTGAACTCACAAATA 60
        ************************************************************

Fc      AGATAAGCTAAAGCTGAAAATGAGCCTCTTATTTTCAAGGTGCAAAGCAGAACAACTAAA 120
Cs      AGATAAGCTAAAGCTGAAAATGAGCCTCTTATTTTCAAGGTGCAAAGCAGAACAACTAAA 120
        ************************************************************

Fc      ATAAAATGAATAAATACACAATCCGAAACGTGATTTTAATACAAACACTTT-----GATA 175
Cs      ATAGAATGAATAAATACACAATCCGAAACGTGATTTTAATACAAACACTTTCATTTCATA 180
        *** ***********************************************      ***

Fc      TGTATCATGCATGCACGAACAGCTAATAAGCAAGCGGTGGAAGTCTTTAGAATCACGTTC 235
Cs      TGTATCATGCATGCACGAACAGCTAATAAGCAAGAGGTGGAAGTCTTTAGAAGCACGTTC 240
        ********************************** ***************** *******

Fc      TGATGTTGTACAATCCAATGAACCTTGAAGTTCCCAGGTTATAAGTAAACAGCACGACAA 295
Cs      TGATGTTGTACAATGCAATGAACCTCGAAGTTCCCAGGTTATAAGTAAACAGAACGACAA 300
        ************** ********** ************************** *******

Fc      AAACTTCGTGGACTGACTTGGTCAAACTATC-TAAACAGTGAGCCAATTTTTGTGTGCGT 354
Cs      AAACTTCGTGGACTGACTTGGTCAAACTATCCTAAACAGTGAGCCAATTTTTGTGTGCGT 360
        ******************************* ****************************

Fc      TTCCATATTTGTAGTAACAGCACAATTGAGTGCCATGTTATGACAACCTTGACCTGGAAT 414
Cs      TTCCTCATTTGTAGTAACAGCACAATTGAGTGCCATGTTATGACAACCTTGACCTGGAAT 420
        ****  ******************************************************

Fc      GGTCCAGCATACTATTACACGTTGCAGCCAACGTTTATCACATATTTGAAAGTACATCCA 474
Cs      GGTCCAGCATACTATTACAGGTTGCAGCCAACGTTTATCACATATTTGAAAGTACATCCA 480
        ******************* ****************************************

Fc      TAACCCTGATCATTAAAAAATTTATATATATGAAAGGTGATTTTTT--TATTTTTTTTAC 532
Cs      TAACCCTGATCAT--CAAAATATATATATATGAAAGGTGGTTTTTTTTTTTTTTTTTTAC 538
        *************   ***** ***************** ******  * **********

Fc      CTTGAAAAAATTCATATTAACGTTATCAATGATTTTTTTTTAATAGTTTACCACTTATTT 592
Cs      CTTGAAAAAATTCATATTAACGTTATCAATGATTTTTTTTTAATAGTTTACCACTTATTT 598
        ************************************************************

Fc      TTTTATAACACCTTGGTAATTTTGACATTAGGTAGCAATATAATACGATAAAATTCACCT 652
Cs      TTTTATAACACCTTGGTAATTTTGACATTAGGTAGCAATATAATACGATAAAATTCACCT 658
        ************************************************************

Fc      CCATGTAATTTGAAGTTCTTTTCAATAATTTTTTTGACAAATTTTATAGAAGAATTTAAC 712
Cs      CCATGTAATTTGAAGTTCTTTTCAATAATTTTTTTGACAAATTTTATAGAAGAATTTAAC 718
        ************************************************************

Fc      CTTTTTTT--TTTTTTCAAACGAAGAAATGTTTTCGTCATTCAATTAAAATTAATGACAT 770
Cs      CTTTTTTTTTTTGGTTCAAACGAAGAAATGTTTCCGTCATTCAATTAAAATTAATGACAT 778
        ********  **  ******************* **************************
```

```
Fc      CATCTAGTGGCTTGGTGACGTACGCTTTAGATACAATTGTCATTCTTGCCTTTTCCTTTC 830
Cs      CATCTAGTGGCTCGGTGACATACGCTTTAGATACAATTGTCATTCTTGCCTTTTCCTTTC 838
        ************ ****** ****************************************

Fc      TCTATATAAACCCCTTTTGCCTTGAACTTTGTTTCAACTAAAGCAGCTCCTCCTCATCCC 890
Cs      TCTATATAAACCCCTTTTGCCTTGAACTTTGTTTCAACTAAAGCAGCTCCTCCTCATCCC 898
        ************************************************************

Fc      TTACTGTCTTTGCTTTCTCACTAACTACTACAACCCAACAGTTTTCTTCTCTCAAAAATG 950
Cs      TTACTGTCTTTGCTTTCTCACTAACTACTACAACCCAACAGTTTTCTTCTCTCAAAAATG 958
        ************************************************************

Fc      GAATGCAAACACAAAATTAATGTAGCAATCCCAATCACTAATATGAAGAACACTCAATTC 1010
Cs      GAATGCAAACACAAAATTAATGTAGCAATCCCAATCACTAATATGAAGAACACTCAATTC 1018
        ************************************************************

Fc      TCATCTCCATCTACTTTCTCTACTTCTCCTCCTTCTCAATCTTCTCCACGCTTCCCTTCT 1070
Cs      TCATCTCCATCTACTTTCTCTACTTCTCCTCCTTCTCAATCTTCTCCACGCTTCCCTTCT 1078
        ************************************************************

Fc      CCTAATCATCAACAATTGTCTTCTCCACAATCTTCTCCAAGCTTTAAAGCTTCTCCTTCA 1130
Cs      CCTAATCATCAACAATTGTCTTCTCCAGAATCTTCTCCAAGCTTTAAAGCTTCTCCTTCA 1138
        *************************** ********************************

Fc      CAATCCTCTCCAAATCTTGCAGATCCCCTCTCTCCGCCGCCTATAGTTCTTAGCCCTTGT 1190
Cs      CAATCCTCTCCAAATCTTGCAGCTCCCCTCTCTCCGCCGCCTATAGTTCTTAGCCCTTGT 1198
        ********************** *************************************

Fc      GCTGCTTGCAAAATCCTCCGCCGCAGATGCGTCGAGAAATGTGTTTTAGCTCCATATTTT 1250
Cs      GCTGCTTGCAAAATCCTCCGCCGCAGATGCGTCGAGAAATGTGTTTTAGCTCCATATTTT 1258
        ************************************************************

Fc      CCACCAACCGAACCATACAAGTTCACCATTGCTCATAGAGTCTTCGGTGCTAGCAATATC 1310
Cs      CCACCAACCGAACCATACAAGTTCACCATTGCTCATAGAGTCTTCGGTGCTAGCAATATC 1318
        ************************************************************

Fc      ATCAAGTTCTTGCAGGTATGCACTTCTTTCGTATGTGATAAATTCAAACTAATTAAATGT 1370
Cs      ATCAAGTTCTTGCAGGTATGCACTTCTTTTGTATGTGATAAATTCAAACTAATTAAATGT 1378
        ***************************** ******************************

Fc      CCAACCATTTTTTTTTTCTAATTGGGAGAAAAAAAAAAGAACTTGTCAATTGTTTTATTT 1430
Cs      CCAACCATTTTTTTT--CTAATTGGGAGAAAAAAAAAA---CTTGTTAATTGTTTTATTT 1433
        ***************  *********************   ***** *************

Fc      TCATCAATTAGTTGTGTGATTAGACTTTGGAGTGGTTGATTGTTCCACTCTTTTTTGGAA 1490
Cs      TCATCAATTAGTTGTGTGATTAGACTTTGGAGTGGTTGATTGTTCCACTCTTTTTTGGAA 1493
        ************************************************************

Fc      ACTTACGGACTTCTCTAATCAAAAGAAAAGAGAGTGTGACATTTCAACTGATTTCATGCA 1550
Cs      ACTTACGGACTTCTCTAATCAAAAGAAAAGAGAGTGTGACATTTCAACTGATTTCATGCA 1553
        ************************************************************

Fc      ACTTTAATGTTTGTTTTCAATTCACTTTCTTTAATTTAAATAGAGTAGATTATTCAAATG 1610
Cs      ACTTTAATGTTTGTTTTCAATTCACTTTCTTTAATTTAAATAGAGTAGATTATTCAAATG 1613
        ************************************************************
```

```
Fc      GATCCTTTTTATTTCTCCTTATAAAGGAATCATCATCACTCTTCCTTGACGGGGCACTGG 1670
Cs      GATCCTTTTTATTTCTCCTTATAAAGGAATCATCATCACTCTTCCTTGACGGGGCACTGG 1673
        ************************************************************

Fc      CGCAGGGTCAATCTGCCTCGTATACAAGTTTTACAAAGCTTAAACTTTGCCCCTTTCTAG 1730
Cs      CGCAGGGTCAATCTGCCTCGTATACAAGTTTTACAAAGCTTAAATTTTGCCCCTTTCTAG 1733
        ******************************************** ***************

Fc      AAAATATTGTGAATTTTACAAAGTCAAACAATAAGTATGAACTTAGCACATGGGTCCACA 1790
Cs      AAAATATTGTGAATTTTACAAAGTCAAACAATGAGTATGAACTTAGCACATGGGTCCACA 1793
        ******************************** ***************************

Fc      TTAGAAATCAATTCCATGCCCCAGCAGTTTAATATCATAAAGTTAGCACATGGCCCGTTA 1850
Cs      TTAGAAACCAATTCCATGCCCCAGCAGCTTAATATCATAAAGTTAGCACATGGCCCGTTA 1853
        ******* ******************* ********************************

Fc      AGACTTAAGCTTTGACAGAATCCAGCGCTCAGAGAACGT-TTAAAATGTTTCTCATTTAG 1909
Cs      AGACTTAAGCTTTGACAGAATCCAGCGCTCAGAGAACGGTTTAAAATGTTTCTCATTTAG 1913
        **************************************  ********************

Fc      ACTAGACTTGCAAATTAAAATTAAAATATTTCAGTCATTTTATTTTATTAAAATGTAAGG 1969
Cs      ACTAGACTTGCAAATTAAAATTAAAATATTTCAGTCATTTTATTTTATTAAAATGTAAGG 1973
        ************************************************************

Fc      GATGGTAAATCCCAAATTAATTTTTAGCCTCCCATCTTCTCAAGATTCAAATTCTGGTGG 2029
Cs      GAGGGTAAATCCCAAATTAATTTTTCGCCTCCCATCTTCTCAAGATTCAAATTCTGGTGG 2033
        ** ********************** **********************************

Fc      TCAGACAATAAAATTGTTTGAAAACGATTTGAAGCAGCCTTGGGGCTGAAAAATTCAGTC 2089
Cs      TCAGACAATAAAATTGTTTGAAAACGATTTGAAGCAGCCTTGGGGCTGAAAATTTCAGTC 2093
        **************************************************** *******

Fc      ATCCATATTTGCACACAAACAATGTCTCATGCCATTAAAAATTTTCCAGGAACTGCCAGA 2149
Cs      ATCCATATTTGCACACAAACAATGTCTCATGCCATTAAAAATTTTCCAGGAACTGCCAGA 2153
        ************************************************************

Fc      ATCTCAACGAGCAGATGCAGTGAGCAGCATGGTCTATGAAGCAAGTGCCAGAATCCGGGA 2209
Cs      ATCTCAACGAGCAGATGCAGTGAGCAGCATGGTCTATGAAGCAAGTGCCAGAATCCGGGA 2213
        ************************************************************

Fc      TCCTGTTTACGGCTGCGCCGGGGCTATTTGCCATCTCCAGAAACAAGTCAGTGAGCTTCA 2269
Cs      TCCTGTTTACGGCTGCGCCGGGGCTATTTGCCATCTCCAGAAACAAGTCAGTGAGCTTCA 2273
        ************************************************************

Fc      GGCTCAGTTAGCCAAGGCACAGGCTGAGCTTGTCACCATGGAAAGCCAGCAACGCAATTT 2329
Cs      GGCTCAGTTAGCCAAGGCACAGGCTGAGCTTGTCACCATGGAAAGCCAGCAACGCAATTT 2333
        ************************************************************

Fc      AATAACTCTAATTTGCATGGAAATGGCACAATCTCAAGAACAAGTCTTGCAGCAGCAGCA 2389
Cs      AATAACTCTAATTTGCATGGAAATGGCACAATCTCAAGAACAAGTCTTGCAGCAGCAGCA 2393
        ************************************************************

Fc      GCAGCAGCAGCAACAGTTCATGGATACTAGCTGTTTTTTGGATGACAATGGCATTGGATC 2449
Cs      GCAGCAGCAGCAACAGTTCATGGATACTAGCTGTTTTTTGGATGACAATGGTATTGGATC 2453
        *************************************************** ********
```

```
Fc      AGCTTGGGAGCCTCTGTGGACATGATCAAGAGAAATTAAAGCAAGATTGTTGAAATTTTA 2509
Cs      AGCTTGGGAGCCTCTGTGGACATGATCAAGAGAAATTAAAGCAAGATTGTTGAAATTTTA 2513
        ************************************************************

Fc      ACCTTTTAAGAGATTATTTACATAAAGCTAAACATACTTAATTATAAAAGTTTCTGATCA 2569
Cs      ACCTTTTAAGAGATTATTTACATAAAGCTAAACATACTTAATTATAAAAGTTTCTGATCA 2573
        ************************************************************

Fc      ATAATTAAAGTTATTTGCTGCGCGGTAGATGGGAGTGATTATTATGTGCTTTAATTTTCA 2629
Cs      ATAATTAAAGTTATTTGCTGCGCGGTAGATGGGAGTGATTATTATGTGCTTTAATTTTCA 2633
        ************************************************************

Fc      TTAGTCTTGTTGACAGAAAGGAATCTTTGAACCATCTGGAGAAGTCCTTTGTTAACGGTT 2689
Cs      TTAGTCTTGTTGACAAAAAGGAATCTTTGAACCATCTGGAGAAGTCCTTTGTTAACGGTT 2693
        *************** ********************************************

Fc      CGAGATTAATTATTAGTTTATCTTTATTTACATTTAGTGAAATTTTGTTTTTAACTAATT 2749
Cs      CGAGATTAATTATTAGTTTATCTTTATTTACATTTAGTGAAATTTTGTTTTTAACTAATT 2753
        ************************************************************

Fc      TTATAGACATAAATAACCCAACCAAGATGGGAATTCAGTGCTGATTTGTGTTTTGTGTTG 2809
Cs      TTATAGACATAAATAACCCAACCAAGATGGGAATTCAGTGCTGATTTGTGTTTCGTGTTG 2813
        ***************************************************** ******

Fc      ATATTTTTAAAAGATTGTGATCGCAAGTTGCATATATGAGATTAAAACTTGTCCATATAT 2869
Cs      ATATTTTTAAAAGACTGTGATCGCAAGTTGCATATATGAGATTAAAACTTGTCCATATAT 2873
        ************** *********************************************

Fc      TATTATAAATTCACATGTAATATTATATTTTAAAAAGATTAACACAACTTATATCAACAT 2929
Cs      TATTATAAATGCACATGTAATATTATATTTTAAAAAGATTAACA--ACTTATATCAACAT 2931
        ********** *********************************  **************

Fc      TGCATTTAAATTCTCTGAAAAAAAAAAGGCAAGAATACAACTTTTTTTTTTTTTACGAGT 2989
Cs      TGCATTTAAATTCTCTGAAAAAAAAA-GGCAAGGATATAACTTTTTTTTTTTTTACGAGT 2990
        ************************** ****** *** **********************

Fc      TTATGCTAATTCTTTGATCAAATTAAAGCGCTATATACAATATATGTAAATTGGTGACCT 3049
Cs      TTATGCTAATCTTTTGATCAAATTAAAGAGCTATATACAATAAATGTATATTGGTGACCT 3050
        **********  **************** ************* ***** ***********

Fc      GATTTTGTCATCTAAAATAGATTTTTTTAGTTCCTTGTCAAACAATCTCTTGACTTTGAC 3109
Cs      AATTTTGTCATCTAAAATAGATTTTTTTAGTTCCTTGTCAAACAATCTCTTGACTTTGAC 3110
         ***********************************************************

Fc      TTTGTCACCTGTATATGATAAAGAAAAATAACCTAAAAATTAAATGAAAATTTTTATGAG 3169
Cs      TTTGTCACCTGTATATGCTAAAGAAAAATAACCTAAAAATTAAATGAAAATTTTTATGAG 3170
        ***************** ******************************************

Fc      TTTTTTTATACATCGCCAACACCAAAAATGAGTGAGGATATTTTATATCTTAAAGATAGA 3229
Cs      TTTTTT-ATACATCGCCGACACCAAAAATGAATAAGGATATTTTATATCTTAAAGATAGA 3229
        ****** ********** ************* * **************************

Fc      ATCCAAAAATTTGCACGCAATATTACAATAATGAAACTACTCTACATAAAATATATTTTT 3289
Cs      ATCCAAAAATGTGCACGCAATATTACAATAATGAAACTACTATACATAAAATATATTTTT 3289
        ********** ****************************** *****************
```

```
Fc        TAGACAATTATTGTCGTTAAAATTCCATTGCGAAGATGTATTATGGAGTAGATTTTTGAA 3349
Cs        TAGACAATTATTGTCGTTAAAATTCCATTGCGAAGATGTATTATGGAGTAGATTTTTGAA 3349
          ************************************************************

Fc        ATTACATCATAATTTTATTTTTCTTAATAATTTTTAGCCGAAAAAGTTTGGGAAATGTCA 3409
Cs        ATTACATCATAATTTTATTTTTCTTAATAATTTTTAACCAAAAAAGTTTGGGAAATGTCA 3409
          ************************************ ** ********************

Fc        TCGAAGAGACTATTGAATTTCTATGCACCTCACACGGGTTGTTGATT      3456
Cs        TCGAAGAGACTATTTAATTTCTATGCACCTCACACGGGTTGTTGATT      3456
          ************** ********************************
```

B

```
Fc        ATATATATCCTAGATATACCAATGTAATATCAAAAGTATTATTTATTATGGGGCTAGCCA 60
Cs        ATATATATTCTAGATATACCAATGTAATATCAAAAGTAGTATTTATTATGGGGCTAGCCA 60
          ******** ***************************** *********************

Fc        ATCACGTTGCTTATCAGCTGGAAAAGACAAATTGATAACTTTTGTATTAACTACACGTGC 120
Cs        ATCACGTTGCTTATCAGCTGGAAAAGACAAATTGATAACTTTTTTATTAACTACACGTGC 120
          ******************************************* ****************

Fc        ACGAAGCTACGCACTAGCTTCCTTGTCTTTAGTTAGGCATGGAAGTCGCCAGACGACTCA 180
Cs        ACGAAGCTACGCACTAGCTTCCTTGTCTTTAGTTAGGCATGGAAGTCGCTAGACGACTCA 180
          ************************************************* **********

Fc        ACTATTGAAGTAGAAAAGTTGATTGTATTAGGTGTTGAAATATACTGTGTAAATCTTGAA 240
Cs        ACTATTGAAGTAGAAAAGTTGAATGTATTAGGTGTTGAAATATACTGTGTAAATCTTGAA 240
          ********************** *************************************

Fc        TGGACTTAAAAATTAGTATAAATACCAACTTGGTATAACTCATTCACTCAAGAGGAGTGG 300
Cs        TGGACTTAAAAATTAGTATAAATACCAACTTGGTATAACTCATTCACTCAAGAGGAGTGG 300
          ************************************************************

Fc        CGCAGCGACAAAGTTGCATTATTATATTATCAATTTCATGACCATGCACACTACATTTCC 360
Cs        CGCAGCGACAAAGTTGCATTATTATATTATCAATTTCATGACCATGCACACTACATTTCC 360
          ************************************************************

Fc        CCCTCTTTCTTCTTCTCCTTCTCCTTCTTTTCAATCTTCTCCAAGCATTAATGCTTCTCC 420
Cs        CCCTCTTTCTTCTTCTCCTTCTCCTTCTTTTCAATCTTCTCCAAGCATTAATGCCTCTCC 420
          ****************************************************** *****

Fc        TTCCAAATCTTCTCCAAATCTTGCTGCCCCACCGCCTATTGTTCTTAGTCCATGTGCAGC 480
Cs        TTCAAAATCTTCTCCAAATCTTGCTGCCCCACCGCCTATTGTTCTTAGTCCATGCGCAGC 480
          *** ************************************************** *****

Fc        TTGCAAAATCCTTCGCCGGAGATGCGACGAGAAGTGTGTTTTAGCTCCATATTTTCCACC 540
Cs        TTGCAAAAGCCTTCGCCGGAGATGCGACGAGAAGTGTGTTTTAGCTCCATATTTTCCACC 540
          ******** ***************************************************

Fc        AACTGAACCACAAAATTTCATCATTGTTCATAGAGTCTTCGGAGCTAGCAACATCATCAA 600
Cs        AACTGAACCACAAAATTTCATCATTGTTCATAGAGTCTTCGGAGCTAGCAACATCATCAA 600
          ************************************************************
```

```
Fc        GTGCTTGCAGGTATGTATGACAGGGACTATAATTAGCTCAAACTATAGGGACAGAAGGAG 660
Cs        GTGCTTGCAGGTATGTATGACAGGGACTATAATTAGCTCAAACTATAGGGACAGAAGGAG 660
          ************************************************************

Fc        CTGGCATCTACTCTTATTTGTGGCTAGCATTTACCCTTATATGTACTAACAAAACATTGT 720
Cs        CTGGCATCTACTCTTATTTGTGGCTAGCATTTACCCTTGTATGTACTAACAAAACATTGT 720
          ************************************** *********************

Fc        GTTCTTCAGGGACTGCCAGAGTGTCAACGATCAGATGCAGTGAGCAGCATGGTCTACGAA 780
Cs        GTTCTTCAGGGACTGCCAGAGTGTCAACGATCAGATGCAGTGAGCAGCATGGTCTACGAA 780
          ************************************************************

Fc        GCAAATGCTAGAATCCGAAATCCGGTGCATGGCTGCGTGGGTGCAATTAGTCAACTCCAC 840
Cs        GCAAACGCTAGAATCCGAAATCCGGTGCATGGCTGCGCGGGTGCAATTAGTCAACTCCAG 840
          ***** ******************************* ********************** 

Fc        AAACAAGTGATTAAGCTACAAGCAGAGTTGGCCAAGGCACAAGCCGAGACGGTCAGCATG 900
Cs        AAACAAGTGATTAAGCTTCAAGCAGAGTTGGCCAAGGCACAAGCCGAGACGGTCAGCATG 900
          ***************** ******************************************

Fc        CAGTGCCAACGAGACAATTTGGTTGCCCTAATTTGCAAGGAAATGACGACACAATATCCT 960
Cs        CAGTGCCAACGAGACAATTTGGTTGCCCTAATTTGCAAGGAAATGACGACACAATTTCCT 960
          ******************************************************* ****

Fc        CAAGAAACCATGAACAGGGTCTTGCCCCAACAGCAATTCAATGACGA---CGCCACCACA 1017
Cs        CAAGAAACCATGAATAGGGTCTTGCCCCAACAGCAATTCAATGACGACGACGCCACCACA 1020
          ************** ********************************   **********

Fc        TGTTATTTAGATGACAAGGATTTTGCCTCCACTTGGGATGCTCTTTGGACTTGAAATTAG 1077
Cs        TGTTATTTAGATGACAAGGATTTTGCCTCCACTTGGGATGCTCTTTGGACTTAAAATTAG 1080
          **************************************************** *******

Fc        AGTTAATTTACATGAAGTTGATGATTGATGAAGAAGAAGAAGAAGAGAGATATTAGTAAT 1137
Cs        AGTTAATTTACATGAAGTTGATGATTGATGAAGAAGAAGAAGAAGAGAGATATTAGTAAT 1140
          ************************************************************

Fc        AATGAATTAGACAGAAAAGTTTCATTTGAAGTATACTATGGGTTGTAAACTTAGTCCGGA 1197
Cs        AATGAATTAGACAGAAAAGTTTCATTTGAAGTATACTATGGGTTGTAAACTTAGTCCGGA 1200
          ************************************************************

Fc        AAGAAATATTGCGATAAATTTGTTGATTGTGCCCTTCATCCTGATTTTTCTGTTA      1252
Cs        AAGAAATATTGCGATAAATTTGTTGATTGTGCCCTTCATCCTGATTTTTCTGTTA      1255
          *******************************************************
```

C

```
Fc        GGTTTTTTTAGTTTCCATATATTATATATGTTAAACAATAATTTTCAAACCCGAAGAGCT 60
Cs        GGTTTTTTTAGTTTCCATATATTATATATGTTAAACAATAATTTTCAAACCCGAAGAGCT 60
          ************************************************************

Fc        TTCATACAATAAAGCCAAAAAAAATGTAAACCTAACGGGAACGAATCAACAAAAAAGAAA 120
Cs        TTCATACAATAAAGCCAAAAAAAATGTAAACCTAACGGGAACGAATCAACAAAAAAGAAA 120
          ************************************************************
```

```
Fc      ATGGCGAAAAACACAGCAGCCACGCAACCTAATTAATTTTCAACTCGAATAATCATGATG 180
Cs      ATGGCGAAAAACACAGCAGCCACGCAACCTAATTAATTTTCAACTCGAATAATCATGATG 180
        ************************************************************

Fc      ACGTCCAATTTCAATACGATGACGTTCTCTTTCCTCTCGCTTTTTGCCTATTTATACACA 240
Cs      ACGTCCAATTTCTTTACGATGACGTTCTCTTTCCTCTCGCTTTTTGCCTATTTATACACA 240
        ************  **********************************************

Fc      TCCCATTTTCACTTCTCAAACCCTGCCCCGCTCCCTCTCTCTCAGGTCTCACTCTCAGCT 300
Cs      TCCCATTTTCACTTCTCAAACCCTGCCCCGCTCCCTCTCTCTCAGGTCTCACTCTCAGCT 300
        ************************************************************

Fc      AATCCCTCTTAAATAGAAATATATCAATATTAATAATTACCAAACCATATGCTAAAAATG 360
Cs      AATCCCTCTTAAATAAAAATATATCAATATTAATAATTACCAAACCATATGCTAAAAATG 360
        *************** ********************************************

Fc      GAGAATTATGAGGCCGCTGCCACAAGAAATCCTAACAAAGTTACCAGTAGCCGTGCTGGT 420
Cs      GAGAACTATGAGGCCGCTGCCACAAGAAATCCTAGCAAAGTTACCAGTAGCCGTGCTGGT 420
        ***** **************************** *************************

Fc      TCGTCATCTCCTCCTCCAATATCAGCTAATTCTTCTGCTCCGCCGCCAGTTATCATGAGC 480
Cs      TCGTCATCTCCTCCTCCAATATCAGCTAATTCTTCTGCTCCGCCGCCAGTTATCATGAGC 480
        ************************************************************

Fc      CCTTGTGCTGCATGCAAGATTCTGAGACGTCGATGTGCTGACAAATGTGTTTTGGCTCCT 540
Cs      CCTTGTGCTGCATGCAAGATTCTGAGACGTCGATGTGCTGACAAATGTGTTTTGGCTCCT 540
        ************************************************************

Fc      TATTTTCCTCCAACCGAGCCTGCCAAGTTCACTATTGCTCATCGTGTTTTCGGTGCCAGT 600
Cs      TATTTTCCTCCAACCGAGCCTGCCAAGTTCACTATTGCTCATCGTGTTTTCGGTGCCAGC 600
        *********************************************************** 

Fc      AATATCATCAAGTTTCTTCAGGTACTCAATTACCTGGTTTATTTGTCAATTTTTCATACT 660
Cs      AATATCATCAAGTTTCTTCAGGTACTCAATTACCTGGTTTCTTTGTCAATTTTTCATACT 660
        **************************************** *******************

Fc      TCAGTGCGTATTTCGTACTTCCGTGAAACTTAAAAGGGTTTGTTAATTAGTCAACATGTT 720
Cs      TCAGTGCGTGTTTCGTACTTCCGTGAAACTTAAAAGGGTTTGTTAATTAGTCAACATGTT 720
        ********* **************************************************

Fc      CATACTGATCTAGCTGAGGAGACTAGGCAGCAGTAACATGTGTTATATGTTAGAGGTGGG 780
Cs      CATACTGATCTAGCTGAGGAGACTAGGCAGCAGTAACATGTGTTATATGTCAGAGGTGGG 780
        ************************************************** *********

Fc      GTGTCGTCGTGCTTTAATGTTTTTAGTGGACTAAGCAAACATAAAACAACTTTTAAAGGG 840
Cs      GTGTCGTCGTGCTTTAATGTTTTTAGTGGACTAAGCAAACATAAAGCAACTTTTAAAGGG 840
        ********************************************* **************

Fc      GGTTGAAGGTAATTTTCGCAGCTCCTGGTCTCAACGTCCCTGTTTATAGTAATGGGATTG 900
Cs      GGTTGAAGGTAATTTTCGCAGCTCCTGGTCTCAACGCCCCTGTTTATAGTAATGGGATTG 900
        ************************************ ***********************

Fc      TGAGTGAAATAATGAGAACACCGGTGCCGTGGGCTGCTGGCTAATTAATAAATAAAAGAA 960
Cs      TGAGTGAAATAATGAGAACACCGGTGCCGTGGGCTGCTGGCTAATTAATAAATAAAAGAA 960
        ************************************************************
```

```
Fc      AGTGACAACTCTAAAATGCACGCGCAGGCCATAAGCGCCTCACATCCGGCGGATAGATAA 1020
Cs      AGTGACAACTCTAAAATGCACGCGCAGGCCATAAGCGCCTCACATCCGGCGGATAGATAA 1020
        ************************************************************

Fc      AGCATATGCCGTTTCTTGATTTTGTCGTTCACAT------GAATATATGTAGTGGAAATG 1074
Cs      AGCATATGCCGTTTCTTGATTTTGTTGTTCACATGTATATGAATATATGTAGTGGAAATG 1080
        ************************* ********      ********************

Fc      GACATAAAGATTTAATTTATTGACTTGTGGATGCAGGAACTTCCAGAGTCTCAGAGAGCA 1134
Cs      GACATAAAGATTTAATTTATTGACTTGTGGATGCAGGAACTTCCAGAGTCTCAGAGAGCA 1140
        ************************************************************

Fc      GATGCTGTGAGCAGCATGGTTTACGAAGCAAGTGCAAGAATCCGGGACCCAGTTTACGGA 1194
Cs      GATGCTGTGAGCAGCATGGTTTACGAAGCAAGTGCAAGAATCCGGGACCCAGTTTACGGA 1200
        ************************************************************

Fc      TGTGCGGGAGCAATTTGTCAGCTACAGAAGCAAGTGAGTGAGCTGCAGGCACAGCTAGCT 1254
Cs      TGTGCGGGAGCAATTTGTCAGCTACAGAAGCAAGTGAGTGAGCTGCAGGCACAGCTAGCT 1260
        ************************************************************

Fc      AAAGCACAAGCCGAGGTTGTGAACATGCAATGCCAGCAAGCAAACCTTGTGGCCTTGCTC 1314
Cs      AAAGCACAAGCCGAGGTTGTGAACATGCAATGCCAGCAAGCAAACCTTGTGGCCTTGCTC 1320
        ************************************************************

Fc      TACAAGGAAATGGGAAAATCGCCGCAGCCCAATTCGCCACAATCTGTTGACCACTTTATT 1374
Cs      TACAAGGAAATGGGAAAATCGCCGCAGCCCAATTCGCCACAATCTGTTGACCACTTTATT 1380
        ************************************************************

Fc      ACAAGCCCAGAAAGCCCTGAAGCAAATCCCTGCAGCTTCTTTGAGGATAACAACTTATCT 1434
Cs      ACAAGCCCAGAAAGCCCTGAAGCAAATCCCTGCAGCTTCTTTGAGGATAACAACTTATCT 1440
        ************************************************************

Fc      GGGTCATTATGGGAACCAGCTCTTTGGACATGATCTTAATTAAAGTATTAGTTAGTTATT 1494
Cs      GGGTCATTATGGGAACCAGCTCTTTGGACATGATCTTAATTAAAGTATTAGTTAGTTATT 1500
        ************************************************************

Fc      TAATTATAATTAGATAATAAT  1515
Cs      TAATTATAATTAGATAATAAT  1521
        *********************
```

Figure S5. DNA sequence alignment of Meiwa kumquat and sweet orange *LOB1*, *LOB2* and *LOB3*. Sequence alignment comparing the Meiwa kumquat and sweet orange surrounding regions (promoter, coding region and intron) of *LOB1*, *LOB2* and *LOB3*. Analysis was conducted with Clustal Omega Multiple Sequence Alignment tool under default setting (https://www.ebi.ec.uk/Tools/msa/clustalo/). All Coding regions are labeled in green. (**A**) Alignment of the *LOB1* surrounding region of Meiwa kumquat (Fc, NCBI accession num' MT247386) and sweet orange (Cs, chromosome 7 28355982-28359437). The effector binding element of PthA4 is labeled in purple. (**B**) Alignment of the *LOB2* surrounding region of Meiwa kumquat (Fc, NCBI accession num' MT247387) and sweet orange (Cs, chromosome 7 28339099-28340353). (**C**) Alignment of the *LOB3* surrounding region of Meiwa kumquat (Fc, NCBI accession num' MT655137) and sweet orange (Cs, chromosome 8 20045688-20047208).
