# Supplementary material for: The immunity of Meiwa kumquat against Xanthomonas citri is associated with a known susceptibility gene induced by a transcription activator-like effector
Source: PLoS Pathog. 2020 Sep 15;16(9):e1008886. doi: 10.1371/journal.ppat.1008886 (PMC7518600; doi:10.1371/journal.ppat.1008886)
Supplement: S6 Fig — Protein sequence alignment comparing LOB1, LOB2 and LOB3 in Meiwa kumquat and sweet orange. Analysis was conducted with the Clustal Omega Multiple Sequence Alignment tool under default settings (https://www.ebi.ec.uk/Tools/msa/clustalo/). (A) Alignment of the LOB1 homologs from Meiwa kumquat (Fc, NCBI accession num’ MT247386) and sweet orange (Cs, Cs7g27640). (B) Alignment of the LOB2 homologs from Meiwa kumquat (Fc, NCBI accession num’ MT247387) and sweet orange (Cs, Cs7g27620). (C) Alignment of the LOB3 homologs from Meiwa kumquat (Fc, NCBI accession num’ MT655137) and sweet orange (Cs, Cs8g17160). (PDF) [file ppat.1008886.s006.pdf]

A

```
Fc      MECKHKINVAIPITNMKNTQFSSPSTFSTSPPSQSSPRFPSPNHQQLSSPQSSPSFKASP 60
Cs      MECKHKINVAIPITNMKNTQFSSPSTFSTSPPSQSSPRFPSPNHQQLSSPESSPSFKASP 60
        **************************************************:*********

Fc      SQSSPNLADPLSPPPIVLSPCAACKILRRRCVEKCVLAPYFPPTEPYKFTIAHRVFGASN 120
Cs      SQSSPNLAAPLSPPPIVLSPCAACKILRRRCVEKCVLAPYFPPTEPYKFTIAHRVFGASN 120
        ******** ***************************************************

Fc      IIKFLQELPESQRADAVSSMVYEASARIRDPVYGCAGAICHLQKQVSELQAQLAKAQAEL 180
Cs      IIKFLQELPESQRADAVSSMVYEASARIRDPVYGCAGAICHLQKQVSELQAQLAKAQAEL 180
        ************************************************************

Fc      VTMESQQRNLITLICMEMAQSQEQVLQQQQQQQQQFMDTSCFLDDNGIGSAWEPLWT    237
Cs      VTMESQQRNLITLICMEMAQSQEQVLQQQQQQQQQFMDTSCFLDDNGIGSAWEPLWT    237
        *********************************************************
```

B

```
Fc      MTMHTTFPPLSSSPSPSFQSSPSINASPSKSSPNLAAPPPIVLSPCAACKILRRRCDEKC 60
Cs      MTMHTTFPPLSSSPSPSFQSSPSINASPSKSSPNLAAPPPIVLSPCAACKSLRRRCDEKC 60
        ************************************************** *********

Fc      VLAPYFPPTEPQNFIIVHRVFGASNIIKCLQGLPECQRSDAVSSMVYEANARIRNPVHGC 120
Cs      VLAPYFPPTEPQNFIIVHRVFGASNIIKCLQGLPECQRSDAVSSMVYEANARIRNPVHGC 120
        ************************************************************

Fc      VGAISQLHKQVIKLQAELAKAQAETVSMQCQRDNLVALICKEMTTQYPQETMNRVLPQQQ 180
Cs      AGAISQLQKQVIKLQAELAKAQAETVSMQCQRDNLVALICKEMTTQFPQETMNRVLPQQQ 180
        .******:**************************************:*************

Fc      FNDD-ATTCYLDDKDFASTWDALWT     204
Cs      FNDDDATTCYLDDKDFASTWDALWT     205
        **** ********************
```

C

```
Fc      MLKMENYEAAATRNPNKVTSSRAGSSSPPPISANSSAPPPVIMSPCAACKILRRRCADKC 60
Cs      MLKMENYEAAATRNPSKVTSSRAGSSSPPPISANSSAPPPVIMSPCAACKILRRRCADKC 60
        ***************.********************************************

Fc      VLAPYFPPTEPAKFTIAHRVFGASNIIKFLQELPESQRADAVSSMVYEASARIRDPVYGC 120
Cs      VLAPYFPPTEPAKFTIAHRVFGASNIIKFLQELPESQRADAVSSMVYEASARIRDPVYGC 120
        ************************************************************

Fc      AGAICQLQKQVSELQAQLAKAQAEVVNMQCQQANLVALLYKEMGKSPQPNSPQSVDHFIT 180
Cs      AGAICQLQKQVSELQAQLAKAQAEVVNMQCQQANLVALLYKEMGKSPQPNSPQSVDHFIT 180
        ************************************************************

Fc      SPESPEANPCSFFEDNNLSGSLWEPALWT 209
Cs      SPESPEANPCSFFEDNNLSGSLWEPALWT 209
        *****************************
```

Figure S6. Protein sequence alignment of Meiwa kumquat and sweet orange *LOB1*, *LOB2* and *LOB3*. Protein sequence alignment comparing Meiwa kumquat and sweet orange LOB1, LOB2 and LOB3. Analysis was conducted with Clustal Omega Multiple Sequence Alignment tool under default setting (https://www.ebi.ec.uk/Tools/msa/clustalo/). (**A**) Alignment of the LOB1 homologs from Meiwa kumquat (Fc, NCBI accession num' MT247386) and sweet orange (Cs, Cs7g27640). (**B**) Alignment of the LOB2 homologs from Meiwa kumquat (Fc, NCBI accession num' MT247387) and sweet orange (Cs, Cs7g27620). (**C**) Alignment of the LOB3 homologs from Meiwa kumquat (Fc, NCBI accession num' MT655137) and sweet orange (Cs, Cs8g17160).
