# Supplementary material for: The immunity of Meiwa kumquat against Xanthomonas citri is associated with a known susceptibility gene induced by a transcription activator-like effector
Source: PLoS Pathog. 2020 Sep 15;16(9):e1008886. doi: 10.1371/journal.ppat.1008886 (PMC7518600; doi:10.1371/journal.ppat.1008886)
Supplement: S7 Fig — DNA sequences represent the promoter regions of LOB1 (A, NCBI accession num’ MT247386), LOB2 (B, NCBI accession num’ MT247387) and LOB3 (C, NCBI accession num’ MT655137) in Meiwa kumquat. Sequences mark the start codons of LOB1, LOB2 and LOB3 (underlined ATG) and TAL effector binding elements (EBEs) of PthA4, PthAW2, dTALEWTLOB1, dTALEAltLOB1, dTALELOB2 and dTALELOB3. (PDF) [file ppat.1008886.s007.pdf]

A

***LOB1* promoter**

dTALEWTLOB1 EBE

CAATTGTCATTCTTGCCTTTTCCTTTCTCTATATAAACCCCTTTTGCCTTGAACTTTGTTT

dTALEAltLOB1 EBE

PthA4/PthAW2 EBE

CAACTAAAGCAGCTCCTCCTCATCCCTTACTGTCTTTGCTTTCTCACTAACTACTACAAC

CCAACAGTTTTCTTCTCTCAAAAATGGAATGCAAACACAAAATTAATGTAGCAATCCCAA

TCACTAAT<u>**ATG**</u>AAG

B

***LOB2* promoter**

dTALELOB2 EBE

TTAAAAATTAGTATAAATACCAACTTGGTATAACTCATTCACTCAAGAGGAGTGGCGCAG

CGACAAAGTTGCATTATTATATTATCAATTTCATGACC<u>**ATG**</u>CAC

C

***LOB3* promoter**

dTALELOB3 EBE

CCTCTCGCTTTTTGCCTATTTATACACATCCCATTTTCACTTCTCAAACCCTGCCCCGCT

CCCTCTCTCTCAGGTCTCACTCTCAGCTAATCCCTCTTAAATAGAAATATATCAATATTA

ATAATTACCAAACCAT<u>**ATG**</u>CTA

S7 Fig. Effector binding elements of *LOB1*-, *LOB2*- and *LOB3*-targeting dTALEs in Meiwa kumquat. DNA sequences represent the promoter regions of *LOB1* (**A**, NCBI accession num' MT247386), *LOB2* (**B**, NCBI accession num' MT247387) and *LOB3* (**C**, NCBI accession num' MT655137) in Meiwa kumquat. Sequences mark the start codons of *LOB1*, *LOB2* and *LOB3* (underlined ATG) and TAL effector binding elements (EBEs) of PthA4, PthAW2, dTALEWTLOB1, dTALEAltLOB1, dTALELOB2 and dTALELOB3.
