# Supplementary material for: The immunity of Meiwa kumquat against Xanthomonas citri is associated with a known susceptibility gene induced by a transcription activator-like effector
Source: PLoS Pathog. 2020 Sep 15;16(9):e1008886. doi: 10.1371/journal.ppat.1008886 (PMC7518600; doi:10.1371/journal.ppat.1008886)
Supplement: S8 Fig — (A) Sweet orange leaves were syringe-inoculated (108 CFU/ml) with Xcc pthA4:Tn5 harboring plasmids encoding the indicated dTALEs. Pictures were taken eight days after inoculation (DPI). (B, C) Meiwa kumquat leaves were syringe-inoculated (108 CFU/ml for B, 106 CFU/ml for C) with Xcc WT and Xcc pthA4:Tn5 harboring plasmids encoding the indicated dTALEs. (B) The mRNA abundance of the indicated genes was quantified at four DPI. The mRNA abundance of GAPDH was used for normalization. Values represent the means ± SE of three independent experimental repeats. Asterisks indicate a significant difference (Student’s t test, p value < 0.05) relative to Xcc pthA4:Tn5. (C) Bacterial populations were determined at the indicated DPI. Values represent the means ± SE of three to five independent leaves taken from different plants. Asterisks indicate a significant difference (Student’s t test, p value < 0.05) relative to Xcc pthA4:Tn5. Experiments were repeated twice with similar results. (PDF) [file ppat.1008886.s008.pdf]

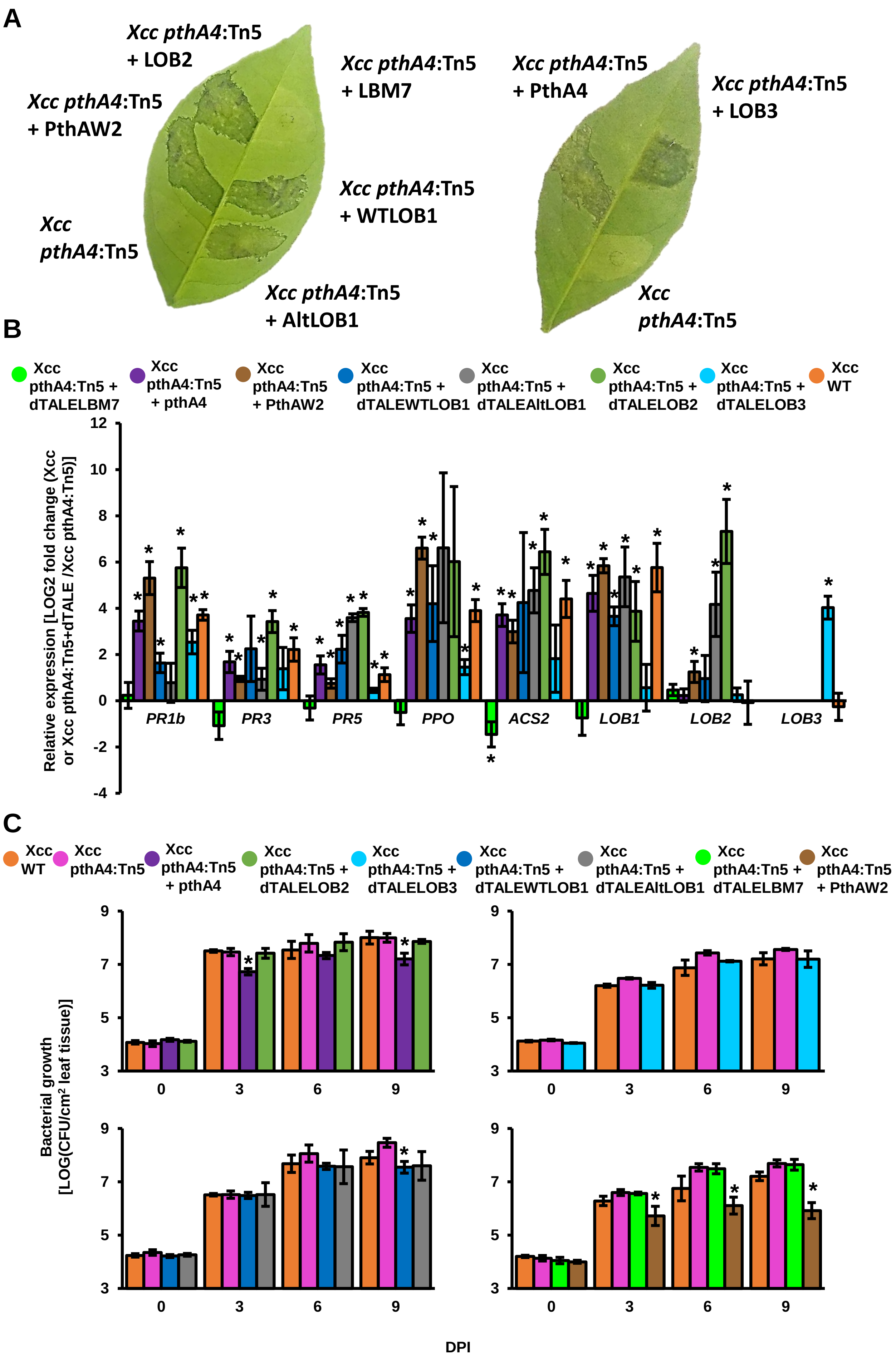

S8 Fig. Functional analysis of *LOB1*-, *LOB2*- and *LOB3*-targeting TALEs in Meiwa kumquat.

(**A**) Sweet orange leaves were syringe-inoculated (10^8 CFU/ml) with *Xcc pthA4*:Tn5 harboring plasmids encoding the indicated dTALEs. Pictures were taken eight days after inoculation (DPI). (**B**, **C**) Meiwa kumquat leaves were syringe-inoculated (10^8 CFU/ml for **B**, 10^6 CFU/ml for **C**) with *Xcc* WT and *Xcc pthA4*:Tn5 harboring plasmids encoding the indicated dTALEs. (**B**) The mRNA abundance of the indicated genes was quantified at four DPI. The mRNA abundance of *GAPDH* was used for normalization. Values represent the means ± SE of three independent experimental repeats. Asterisks indicate a significant difference (Student's *t* test, *p* value < 0.05) relative to *Xcc pthA4*:Tn5. (**C**) Bacterial populations were determined at the indicated DPI. Values represent the means ± SE of three to five independent leaves taken from different plants. Asterisks indicate a significant difference (Student's *t* test, *p* value < 0.05) relative to *Xcc pthA4*:Tn5. Experiments were repeated twice with similar results.
