# Supplementary material for: The immunity of Meiwa kumquat against Xanthomonas citri is associated with a known susceptibility gene induced by a transcription activator-like effector
Source: PLoS Pathog. 2020 Sep 15;16(9):e1008886. doi: 10.1371/journal.ppat.1008886 (PMC7518600; doi:10.1371/journal.ppat.1008886)
Supplement: S9 Fig — The regions 1 kb upstream of the transcriptional start site (predicted promoter) of Meiwa kumquat genes that were found to be upregulated by PthA4 were analyzed using the MEME motif discovery feature (http://meme-suite.org/tools/meme). Parameters were set to analyze strands with a 6–10 width cutoff. Bars represent the frequency of the CACGTC and GGSCCC motifs (represented on the top of each graph in sequence logo format) per promoter for all coding genes and genes that were found to be upregulated by PthA4 in Meiwa kumquat and sweet orange. Asterisks indicate a significant difference (Fisher’s exact test, p value < 0.01) relative to frequency in all coding genes. (PDF) [file ppat.1008886.s009.pdf]

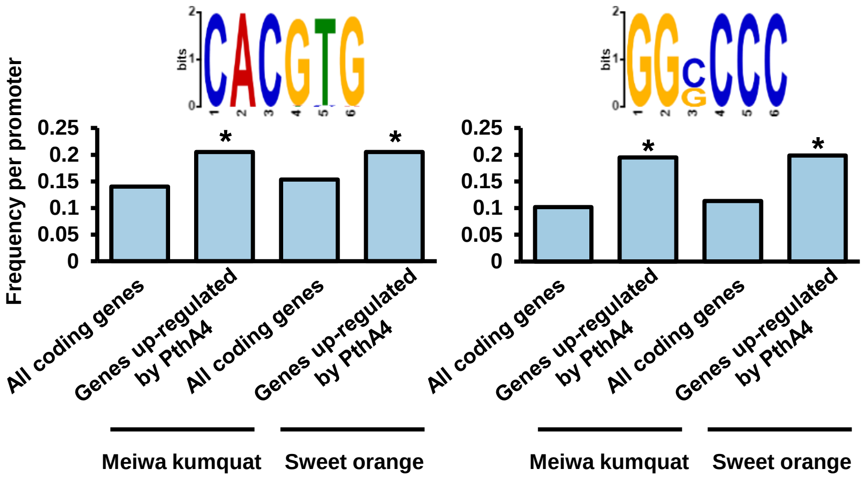

S9 Fig. Enrichment of DNA motifs in the promoters of PthA4-upregulated genes in Meiwa kumquat and sweet orange. The regions 1 kb upstream of the transcriptional start site (predicted promoter) of Meiwa kumquat genes that were found to be upregulated by PthA4 were analyzed using the MEME motif discovery feature (http://meme-suite.org/tools/meme). Parameters were set to analyze strands with a 6-10 width cutoff. Bars represent the frequency of the CACGTC and GGSCCC motifs (represented on the top of each graph in sequence logo format) per promoter for all coding genes and genes that were found to be upregulated by PthA4 in Meiwa kumquat and sweet orange. Asterisks indicate a significant difference (Fisher's exact test, p value < 0.01) relative to frequency in all coding genes.
