# Supplementary material for: The immunity of Meiwa kumquat against Xanthomonas citri is associated with a known susceptibility gene induced by a transcription activator-like effector
Source: PLoS Pathog. 2020 Sep 15;16(9):e1008886. doi: 10.1371/journal.ppat.1008886 (PMC7518600; doi:10.1371/journal.ppat.1008886)
Supplement: S10 Fig — The indicated Xcc cultures (108 CFU/ml)) were syringe-infiltrated into Meiwa kumquat leaves. Plants were treated with either 1 mM norflurazon (+ NF), 0.5 mM abscisic acid (+ ABA), 10 mM α-aminoisobutyric acid (+ AIB), 0.1 mM 1-aminocyclopropane-1-carboxylic acid (ACC) or water as a control. NF and AIB were applied at two, four and six days post inoculation (DPI). ABA and ACC were applied at four and six DPI. Leaves were photographed at eight DPI. Experiments were repeated three times with similar results. (PDF) [file ppat.1008886.s010.pdf]

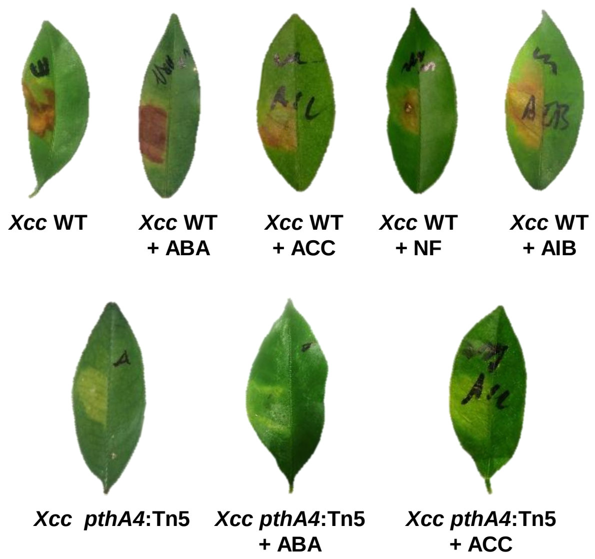

S10 Fig. Effects of ABA and ACC treatments on *Xcc*-inoculated Meiwa kumquat leaves. The indicated *Xcc* cultures (10^8 CFU/ml)) were syringe-infiltrated into Meiwa kumquat leaves. Plants were treated with either 1 mM norflurazon (+ NF), 0.5 mM abscisic acid (+ ABA), 10 mM α-aminoisobutyric acid (+ AIB), 0.1 mM 1-aminocyclopropane-1-carboxylic acid (ACC) or water as a control. NF and AIB were applied at two, four and six days post inoculation (DPI). ABA and ACC were applied at four and six DPI. Leaves were photographed at eight DPI. Experiments were repeated three times with similar results.
