# Supplementary material for: The immunity of Meiwa kumquat against Xanthomonas citri is associated with a known susceptibility gene induced by a transcription activator-like effector
Source: PLoS Pathog. 2020 Sep 15;16(9):e1008886. doi: 10.1371/journal.ppat.1008886 (PMC7518600; doi:10.1371/journal.ppat.1008886)
Supplement: S1 Table — (DOCX) [file ppat.1008886.s011.docx]

**S1 Table. Bacterial strains and plasmids used in this study**

| Strain or plasmid | Relevant characteristic | Reference |
| --- | --- | --- |
| **Strain** | | |
| ***Escherichia coli*** | | |
| HST08 | F–, endA1, supE44, thi-1, recA1, relA1, gyrA96, phoA, Φ80d lacZΔ M15, Δ (lacZYA - argF) U169, Δ (mrr - hsdRMS - mcrBC), ΔmcrA, λ– | Clontech Laboratories Inc, Mountain View, CA |
| S-17-1 λpir | RK2 tra regulon, pir, host for pir-dependent plasmid pOK1 | [1] |
| ***Xanthomonas citri* subsp *citri*** | | |
| *Xcc* 306 | Wild-type *Xcc*^A^ strain | [2] |
| *Xcc* *pthA4*:Tn5 | Tn5 insertion mutant in XACb0065 (strain 320E11) in the background of *Xcc* 306. Kn^R*^ | [3] |
| *Xcc* ∆*xpsM/N/D* | *Xcc* 306 carrying a deletion of the 4178852-4181737 genomic fragment [harboding *xpsM* (XAC3536), *xpsN* (XAC3535) and *xpsD* (XAC3534)] from the T2SS *xps* operon | This study |
| *Xcc* ∆*xopE1* | *Xcc* 306 carrying a deletion of *xopE1* (XAC0286) | This study |
| *Xcc*^AW^ Aw12879 | *Xcc*^AW^ strain | [4] |
| **Plasmid** | | |
| pBBR1MCS-5 | Broad host expression vector. Gn^R^ | [5] |
| pBBRNPth | pBBR1MCS-5 derivative for expression of -24 to +444 N-terminal coding fragment of PthA4 (XACb0065) and a HA tag. Used as backbone for construction of dTALEs. Gn^R^ | This study |
| pBBR1MCS-5:*pthA4* | pBBR1MCS-5 derivative for expression of PthA4 (XACb0065). Gn^R^ | This study |
| pBBR1MCS-5:*pthAW2* | pBBR1MCS-5 derivative for expression of PthA4 (XCAW_b00026). Gn^R^ | This study |
| pTAL2 | Intermediate destination vector for assembled dTALEs. Ap^R^ | [6] |
| dTALEWTLOB1 | pBBR1MCS-5 expressing deigned TALE (RVD repeat array: NI NG NI NI NI HD HD NG HD NG NG NG NG NN HD HD NG NG NN) fused to HA tag. Gn^R^ | This study |
| dTALEAltLOB1 | pBBR1MCS-5 expressing deigned TALE (RVD repeat array: NI NI NI NN HD NI NN HD NG HD HD NG HD HD NG HD) fused to HA tag. Gn^R^ | This study |
| dTALELOB2 | pBBR1MCS-5 expressing deigned TALE (RVD repeat array: NI NN NG NI NG NI NI NI NG NI HD HD NI NI HD NG) fused to HA tag. Gn^R^ | This study |
| dTALELOB3 | pBBR1MCS-5 expressing deigned TALE (RVD repeat array: NN HD HD NG NI NG NG NG NI NG NI HD NI HD NI NG HD) fused to HA tag. Gn^R^ | This study |
| dTALELBM7 | pBBR1MCS-5 expressing deigned TALE (RVD repeat array: NI NG NI NG NI NI NI NI HD HD NG NG NG NN HD HD NG NG NN) fused to HA tag. Gn^R^ | This study |
| pUC18 | Cloning vector. Ap^R^ | Thermo Fisher Scientific, Waltham, MA |
| pUC18*xps* | pUC18 cloned with the *Xcc* 306 4178238-4182444 T2SS coding genomic region. Ap^R^ | This study |
| pUC18del*xps* | pUC18*xps* digested and re-ligated with EcoRV (cutting out the 4178852-4181737 *Xcc* 306 region). Ap^R^ | This study |
| pOK1 | *sacB* *sacQ* *mobRK2* *oriR6K*, Suicide vector. Sp^R^ | [7] |
| pOK1*xps* | pOK1 derivative containing the 707 bp 5’ and 614 bp 3’ flanking regions of the *Xcc* 306 4178852-4181737 genomic area encoding for *xpsM* (XAC3536), *xpsN* (XAC3535) and *xpsD* (XAC3534). Sp^R^ | This study |
| pOK1 *xopE1* | pOK1 derivative containing the 807 bp 5’ and 975 bp 3’ flanking regions of the genomic area encoding for *xopE1* (XAC0286). Sp^R^ | This study |
| pGEM-T | T-vector cloning system. Ap^R^ | Promega, Madison, WI |
| pGEM5’LOB1FC | pGEM-T derivative containing the 1,097 bp of the 5’ area of Meiwa kumquat *LOB1.* Ap^R^ | This study |
| pGEM3’LOB1FC | pGEM-T derivative containing the 1,068 bp of the 3’ area of Meiwa kumquat *LOB1.* Ap^R^ | This study |
| pGEMEBELOB1FC | pGEM-T derivative containing the 182 bp harboring the PthA4 EBE area in the promoter region of of Meiwa kumquat *LOB1.* Ap^R^ | This study |
| pGEMCDSLOB1FC | pGEM-T derivative containing the 1,479 bp of the CDS + intron area of Meiwa kumquat *LOB1.* Ap^R^ | This study |
| pHSG298 | Cloning vector. Kn^R^ | TaKaRa Bio Inc. Kusatsu, Japan |
| pHSG298:LOB2 | pHSG298 derivative containing the 1,264 bp covering the 5’ promoter, CDS and intron areas of Meiwa kumquat *LOB2*. Kn^R^ | This study |
| pGEM:LOB3CDS | pGEM-T derivative containing 1,311 bp covering the 5’ UTR, CDS and intron areas of Meiwa kumquat *LOB3*. Ap^R^ | This study |
| pGEM:LOB3p | pGEM-T derivative containing 645 bp covering the 5’ promoter and part of the CDS Meiwa kumquat *LOB3*. Ap^R^ | This study |

*Kn^R^, Gn^R^ , Sp^R^ and Ap^R^ indicate resistance to kanamycin, gentamicin, spectinomycin and ampicillin, respectively.

**References:**

1. Simon R, Priefer U, Pühler A. A broad host range mobilization system for in vivo genetic engineering: Transposon mutagenesis in gram negative bacteria. Bio/Technology. 1983;1: 784–791. doi:10.1038/nbt1183-784

2. Da Silva ACR, Ferro JA, Reinach FC, Farah CS, Furlan LR, Quaggio RB, et al. Comparison of the genomes of two Xanthomonas pathogens with differing host specificities. Nature. 2002;417: 459–463. doi:10.1038/417459a

3. Yan Q, Wang N. High-throughput screening and analysis of genes of Xanthomonas citri subsp. citri involved in citrus canker symptom development. Mol Plant Microbe Interact. 2011;25: 1–72. doi:10.1094/MPMI-05-11-0121

4. Jalan N, Kumar D, Andrade MO, Yu F, Jones JB, Graham JH, et al. Comparative genomic and transcriptome analyses of pathotypes of Xanthomonas citri subsp. citri provide insights into mechanisms of bacterial virulence and host range. BMC Genomics. 2013;14: 551. doi:10.1186/1471-2164-14-551

5. Kovach ME, Elzer PH, Steven Hill D, Robertson GT, Farris MA, Roop RM, et al. Four new derivatives of the broad-host-range cloning vector pBBR1MCS, carrying different antibiotic-resistance cassettes. Gene. 1995;166: 175–176. doi:10.1016/0378-1119(95)00584-1

6. Cermak T, Doyle EL, Christian M, Wang L, Zhang Y, Schmidt C, et al. Efficient design and assembly of custom TALEN and other TAL effector-based constructs for DNA targeting. Nucleic Acids Res. 2011;39: e82. doi:10.1093/nar/gkr218

7. Huguet E, Hahn K, Wengelnik K, Bonas U. hpaA mutants of Xanthomonas campestris pv. vesicatoria are affected in pathogenicity but retain the ability to induce host-specific hypersensitive reaction. Mol Microbiol. 1998;29: 1379–1390. doi:10.1046/j.1365-2958.1998.01019.x
