# Supplementary material for: The immunity of Meiwa kumquat against Xanthomonas citri is associated with a known susceptibility gene induced by a transcription activator-like effector
Source: PLoS Pathog. 2020 Sep 15;16(9):e1008886. doi: 10.1371/journal.ppat.1008886 (PMC7518600; doi:10.1371/journal.ppat.1008886)
Supplement: S2 Table — (DOCX) [file ppat.1008886.s012.docx]

**S2 Table. Primers used during this study**

| Primer name | Sequence (5’ to 3’) (Underline represents restriction sites) | | | | Destination vector |
| --- | --- | --- | --- | --- | --- |
| *For construction of pBBRNPthF* | | | | | |
| NPthF | AAAGTCGACCGACCATGTAAAGAGGTATGCCTG | | | | pBBR1MCS-5 |
| NPthR | CCCGAATTCCGAAGCGTCGGAGGGTTGCG | | | | pBBR1MCS-5 |
| *For construction of pBBR5:pthAW2* | | | | | |
| pthAW2F | TCTAGAGGAGGCAGGGATTTGTATA | | | | pBBR1MCS-5 |
| pthAW2R | GAATTCTTCACCCGATCTATCCCTTG | | | | pBBR1MCS-5 |
| *For construction of pOK1 vectors* | | | | | |
| flaxopE15FXba | AAAATCTAGATGGCTTTACCGCGTATGCG | | | | pOK1 |
| flaxopE15R | GCTGTTCGCCTGATCGAAGAGTTCACGGCCTTGAAACGCATAGTCCCAT | | | | pOK1 |
| flaxopE13F | ATGGGACTATGCGTTTCAAGGCCGTGAACTCTTCGATCAGGCGAACAGC | | | | pOK1 |
| flaxopE1Rbam | CCCGGATCCACAACGGTGCGAGGAACACG | | | | pOK1 |
| xpsFBam | AAAGGATCCGATCGGATTGAGCAACGAAGCC | | | | pOK1/pUC18 |
| xpsRXba | CCAATCTAGAGACAAGTGTAGGCCGCAGTAACC | | | | pOK1/pUC18 |
| *For cloning the LOB1 region in Meiwa kumquat* | | | | | |
| LOBsu5F | AGTAATTCAACCATTTGATTTGCAATCACC | | | | pGEM-T |
| LOBsu5R | TCTGGAGAAGACAATTGTTGATGATTAGG | | | | pGEM-T |
| LOBsu3F | AGCAGCAGCAGCAACAGTTCATGG | | | | pGEM-T |
| LOBsu3R | AAATCAACAACCCGTGTGAGGTGC | | | | pGEM-T |
| LOB1CSDR | CCCCACTAGTATGAAGAACACTCAATTCTCATCTCCATC | | | | pGEM-T |
| LOB1CSDF | AAAGGGCCCTGTCCACAGAGGCTCCCAAG | | | | pGEM-T |
| LOB1EBEF | AATGACATCATCTAGTGGCTCGGTGAC | | | | pGEM-T |
| LOB1EBER | TGAGAGAAGAAAACTGTTGGGTTGTAG | | | | pGEM-T |
| *For cloning the LOB2 region in Meiwa kumquat* | | | | | |
| LOB2seqF | AAGGATCCTGTCATGCAAAATATTCTCTAGCTC | | | | pHSG298 |
| LOB2seqR | GGGAATTCATGTTAAAGTCTTTCACTGTGTC | | | | pHSG298 |
| *For cloning the LOB3 region in Meiwa kumquat* | | | | | |
| LOB3pseqF | ATAAATGCACGTGCGACAAA | | | | pGEM-T |
| LOB3pseqF | ACCAGCACGGCTACTGGTAA | | | | pGEM-T |
| LOB3ORFseqF | GCTCCCTCTCTCTCAGGTCTC | | | | pGEM-T |
| LOB3ORFseqR | CCCATTGGCTTTGAGTTAGG | | | | pGEM-T |
| *For sequencing of dTALEs* | | | | | |
| TAL_Seq_5-1 | CATCGCGCAATGCACTGAC | | | | |
| TAL_R3 | GGCTCAGCTGGGCCACAATG | | | | |
| *For RT-qPCR* | | | | | |
| Target gene | | Target gene locus | Forward primer sequence (5’ to 3’) | Reverse primer sequence (5’ to 3’) | |
| GAPDH | | Cs5g06870 | GAAAGGTCTTGCCTGCTTTG | TCCTTCTCCAGCCTCACTGT | |
| LOB1 | | Cs7g27640 | TCCACCAACCGAACCATACA | GGCACTTGCTTCATAGACCAT | |
| GEM-like protein 4 | | Cs7g13465 | ATGAGTAAGCTAGGGAAGAAAGCA | TCTCCACACCACCTACTTGAATAA | |
| MATE | | Cs2g15080 | TCTCTTGGTGGTTTCTTGTCATTA | GAAGCAACAGTGAGCTTGAAGTAA | |
| 14-3-3 | | Cs7g11330 | CCCTAAGTGAGGAATCATACAAGG | AAGTTATCTTCACCTCCATCTTCG | |
| HSTFA2 | | Cs4g14590 | CAGAAGACATTGAGATTGGAAATG | AATTTTGGCAAAGAAATTCAACAT | |
| ACS2 | | orange1.1t00416 | GTTTAGGGTTTGCTTTGCTAACAT | GTTTCTTGGTTGGTTTCTCACTTT | |
| ACS6 | | Cs5g03060 | GATTTGAGATGGAGAACAGGAGTT | ACACCCTTTATTCTGATGTTGGAT | |
| PR2 | | orange1.1t00643 | CCTTGTTCCCGCCATGAG | GCCAAGAGCTCCAGTTTCGA | |
| PR3 | | Cs1g26330 | GGCTCAAACTTCACATGAAACTAC | GTTGACAATAATCTCCAGGGTTTC | |
| PR5 | | Cs3g24410 | ATTGCCAATAACCCTAATGAAAAA | GACAGTTACCGTTAAGATCAGCAA | |
| ACO | | Cs3g20140 | CAGAGTACCGATCTGAGTTGAAGA | CATTGAATGCTTTCTTGATGTACC | |
| PR10 | | Cs9g05330 | TACAAGCAAGTGAAGCAAAGAATC | CTTAACATCGTACACCCCTTTCTC | |
| SPX | | Cs1g12640 | TGTCATCAGATTAAAGGAGCTGAA | ACTGTAGTGCAACAGACAAACCAT | |
| PR1b | | Cs8g03360 | GATGGGAAGCCATTATACGACTAC | ACAAAGTTGAGAGTGCCATTGTTA | |
| PR1c | | Cs4g02980 | ACAAACACACATCTCCGAAATGA | TTGAAATGAGCAGCAGCAAAA | |
| CML | | Cs7g07260 | TCTTCATCCTCTTCGAGAAAGTCT | CTTCAACTTCCTCAACCAAATCTT | |
| PP2C | | Cs5g02310 | GTTCGAGTCGTAGGAAAATCACTT | AGGTACCTTTAAACCCGTATCACA | |
| LOB40 | | Cs6g18700 | GAAAAGGAAGCTTGAAGATCGTAG | TGTCCACCTCCATTCACTACTCTA | |
| Cysteine protease | | Cs9g13580 | TTGAAGATGTAAAACCCAATGATG | AAAAATACCGCCTTTGTAACTCTG | |
| Cytochrome P450 | | orange1.1t01519 | TGGATAAATTAATCAACAGGCTCA | ATTTTCTCAATCGTCTTCTCATCC | |
| 3-ketoacyl-CoA synthase | | Cs4g06430 | TACAGAAGAACCTTGAGCTGAGTG | TCCAAGTACGCTAGTTCATACCAA | |
| Endoglucanase 8 | | Cs2g17090 | TGACGTCACTGTCTTTCCTACTTC | GTTTTGCCAATTGTCTTAGAAGGT | |
| Endoglucanase 11 | | Cs2g20750 | GTTCATGCCTTAACAAGAACAATG | AGTCAAGCAGCAAGTCAGAGTAGA | |
| Endoglucanase CX | | Cs5g01400 | GCTGATTCCAGGATCATCTAGTTT | AGGTACTTGGCATACGTTAAGAGG | |
| Endoglucanase 6 | | Cs5g02320 | TTCTGCTGGTAGAGACTTGAAATG | CTTTGTGGATAGTTGTTGCCATAG | |
| Endoglucanase 9 | | Cs5g20320 | AAATGTCATACATGGTTGGTTTTG | TTAGGGTTTGATGAGTGGAAGAAT | |
| Desiccation-related protein | | Cs9g17250 | TTTTGAAGTGTTGTATGCTTGGAT | TGAAACTAAAACCACAAGGCACTA | |
| ERD1 | | Cs6g07270 | AACTTCTTGCCTCTTTTCACTGTT | TTATTAACAGCCCATGATAGAGCA | |
| NCED | | Cs5g14370 | ATGAAGTTGTTGTAATTGGGTCCT | TTGAGTCTAATTTCGGACAAAACA | |
| HSP22 | | Cs9g14675 | TAAGGTAACCAAAGATGCACTGAA | GCTCTCTTCATCACCATCTTCTTT | |
| HSP18.1 | | Cs7g32260 | TGGCTTTGAGTCTTTTTGATAGTG | GAATATTAGCGACTGACGATGAGA | |
| HVA22-I | | Cs3g25990 | GTATATCGGCACTTCGTTAGACCT | GCAGCAGTAAGAATGTCATCTTGT | |
| HVA22-D | | orange1.1t02608 | ATATGAAAGGTTTGTCAGACAGCA | TAAAGTCCACAAACTTGTTCTTGC | |
| PAP | | Cs3g25470 | GCTTACGGGAAATATACTCCTCAA | GCTATTATACCATGGGGAGTGAAG | |
| SAM-1 | | Cs6g18060 | ACAACAAGGGGAGGATTTATTACA | ATGGTACAATAAGAGCCATTAGCC | |
| SAM-2 | | Cs7g31430 | AATTCCATGGGAATCTTTATCTGA | ACGATCCCTCTTTGCTCACTATAC | |
| SAM-3 | | Cs6g18050 | AGGCCTAGAATTCCAAGTTTTCTT | TGTAGAGACGACTCAGGAAACAAG | |
| SNK-1 | | Cs2g31040 | CAAAATGCGATTATAGATGCTCAG | TCTTTGTCTTCCAGTTGTTGTAGC | |
| SNK-2 | | Cs6g20220 | TTTTATCCAACAATGAGGAGGAAT | ATTGGCTTAACTGGAGCCTTAGTA | |
| PPO | | Cs2g06420 | TTTTACTCCGCTGGGAGAGA | AATCCGGCTCAGTGAAATTG | |
| LOB2 | | Cs7g27620 | CCACAAAATTTCATCATTGTTCAT | GCTCCTTCTGTCCCTATAGTTTGA | |
| Expansin | | Cs9g15150 | ACAAAGCCCTTTCTCTTCTTTTGT | ATCTGTTATCACAACTCGAACTGC | |
| ERF | | orange1.1t00851 | CAAACTCAGCAACAAATGATCTCT | ATGCATCACTCCAATACTGAAGAA | |
| LOB3 | | Cs8g17160 | TCCTCCTCCAATATCAGCTAATTC | GAAAATAAGGAGCCAAAACACATT | |
