# Supplementary material for: The immunity of Meiwa kumquat against Xanthomonas citri is associated with a known susceptibility gene induced by a transcription activator-like effector
Source: PLoS Pathog. 2020 Sep 15;16(9):e1008886. doi: 10.1371/journal.ppat.1008886 (PMC7518600; doi:10.1371/journal.ppat.1008886)
Supplement: S1 File — (DOCX) [file ppat.1008886.s020.docx]

>Cs1g09430

tgtatttttagggcaccgtcgatttttaccacatgACCACTCATTTAGATCAttaaagaaggtttcaataaTAAAGCAGAAtgGGATGAACCTcAAAAAACTTTGTCTACTCAAGAAATTTTTGAAACTGGTAGATGGTATTAACTATAAGAATAGTAAGCATAGAGGCAGTAAGCGAAAGCGTGATGATTGTGATAATGATATTGACAAATTATTCAAAAGAAAATCAATTTTTTTTATTTAGAATATTGGCAACATTAATTAGTTCGCCATGTGTTAGATGTGATGCACATTGAGAAAAATATTTATGAGACATCTATGACACATTACTTCATCAACCTAAAAAAATGAAAGGTGGGGTCAAGGCTAGAACAAATCTAATTGAATTATAGATTAGGGGCAAATTAATTCATAGTGAAAATGATAAAAATTCCATATCTGCAGCACCTTACACCTTGAGTAAaaaagaaaaaaatgttttGTCAAACTTTGTTTGAAACCAAAGTTCCAGAAGGTTATTCTTCCAACTTCAGCAGTCTTGTAAACTTAGATTATTATCGGCTTCAAGGTCTTAAATCTCATTACTGCCACACTTCAGGAGTATTTTACTTCAATGCATTGTGTAGTAAGGTGCTAGAAACAAAATCTCTTGACAAATTGGAAAAACAACATTACGTCACTATGTGCTTGTTTGAGCAATTTTTCCAAccttatttttttttatataaTGGTCCACTTGACGGTACATCTTGTTGACCAAGTCCGATTATGTGGACCTGTTCACCTTTGCTGGATGTATTCATTTGAGAGAAACATGAAATTGCTAAAGGGCTATGTTTTTAACCATTGTCACCCTGAGGGATGCATTGcCAAAtgtTATGTGGCAAAGGAAGCacttgaattTTGTGCTGAATAATTGTCCaATCATgatttcattggaCTGCCACCTAGTTGCATGGTAGATTATACAATTGAAAAGCCCTTAGGAGGAGGAAAGGCTA

>Cs1g01790

ttcagGCTTTTGAGACTGAAATAGAGGAAAAGGGTAGCAAAACTAAAATAATTGACTCCAACTTCAAATTATAGTAAAACTGAAATAAYTAACTCRAAATAAAGGAAATTTAAAGACTCTATATATYATTCTATATTTGTGCATATAATCGTAAACCTATAAACTAAAGTAATTAATTACTTGTTCAACAAGAATAAGTACAATCAAAGCCACCAAGGGCAAGCAATTCTTCTCTCCTCGAGTTATATATTATATAAAATGATGAAATTATATTGATGCACATCAGTGGagatattaagtataTTGAGGCAAATTCTTCTCATCTAGATTAGATTGATTCATAATTGAAAGAGATTACTGTAAGTTCTTTCAAAAttgataatttgtcCCATTATTAGAGGTGTTCCAAAAATGACTGCAATCGAGTAAATAGCTACGAACTAATCAATCAGATTGAATTAGAAAAGGAAAAAATTTGTACACCCATAAATTTGWAATTGGAGCTGTCTAAGAACACTAAAAAATTATCAATCAAGGAACATTAGTTGTACTATCTYATCTGTTTGACTGTTAATTTTTAATTAaacccaaaaaaatgtatcTTTCAGAGCGATATCTTATGAAATATGCAGTTAAGAAATATGCGGCTATATTTATCATAAACTAAATAGTTAAAATATTATAGCAAAATAAAATAACTTTTATAAACAATTTGATCCTTTTACTCTATTAAGATAAAAATTAATTTATCCCTTTGTTCATTACCTTAATAATTAAAATGATGTCAAATTAACCTCAATTTGtttttaaattttacaAAGGCTTTACTACAACCAATAAAATGAGTATAATATGAGGGGCCRGCGGCCCACCGCATGTATTATTTGGGCCTATTGTATAATTTTCTACCGAAACATTTATGGGCTTGTTTCAATATTCGACCCAAAWAATTCGGTTCGGATAAATGATTCGGGCCAGCATCACATCACGAAGCAGAGTCG

>Cs1g04290

nnnnnnnnnnnnnnnnnnnnnnnnnnnnnnnnnnnnnnnnnnnnnnnnnnnnnnnnnnnnnnnnnnnnnnnnnnnnnnnnnnnnnnnnnnnnnnnnnnnnnnnnnnnnnnnnnnnnnnnnnnnnnnnnnnnnnnnnnnnnnnnnnnnnnnnnnnnnnccctatttataaaggagattttaccaaaTTTTCAGAAAAATTCTTACTAATTAGATTATTATCTATTCAGttttGATGCAATTAGATATACTTGCCCTGAGGGTTTGGGTCGGGTCGTTTCACGCATGCACGCAagcgcgtgcgcacactcacaccggcccaaatacacaatcacactaatcacctagcacatcatatatatatttatgtctatgtatgtaactccaatttcaacaaaaagttaatttcatattccatcctatatacatgaaacatcaattaaataattttatttcccagggttggtgggaaagggaaatgatcatgtgacctcttgttttcactataaggggacaaacCaattcGACTTATTCCGTCAGAGATACAAAAAGTTACTTCTTGCTASTTAATATTCTGGTGACATTAAAATAAAATAGTAACWAGCATATTTATGRGCAAATCCAAAATATTTATGTTACTTTCAKAAAATATTTAAGTGTTGTACAAATTTTATTTTACTCATTAGTTTCCCTTCTTTCTATATATTAAATACATGTTCTTATAYAACAAGTAACAAGAATTAGTAGAATTTGATATATCTCAATCTAATCTATTGACCCAGAGAGTGAGAATTTTTTTAATATTGCCTATCGTTGAAATCACCTCCATTTGTTGACTTTGTATATTTTCTAAGGGATTCGCTTTGATTTTCTTTATTATTTTGGAGATCCAATCTTTCAAGAAGCAATTATGCAAGACACTTATAAATATGATGAATTTGGTTGCACACTATATGCCACTCAACACTCACTTTGGTGAATAATTTCTCAAAGCATGCAAAAKA

>Cs1g15920

AATAATAACATAAATAATTTTAAAGTTTATTTTATTTGGGTTTAATTGTCTTAGTAACACTACTTTTTGGCTGCAGGGGATCATCTTATTCAGGAGAAACAGCAGTGGTGTACAAATCAAGTGAAAGAAAAAGGCTTGAGAAGAAAGGATACAGAATCATTGGCAACATCGGCGATCAATGGAGCGATCTCTTGGGCACCAACGCTGGCAATCGGACGTTCAAGCTGCCTGATCCAATGTATTACATTAGCTGATTAATAAATAATGTTTACTTAGGATTTTGAATAAATAATGCAAGAGCCCCCATTTGTGCACCAAATTTGTACGCAGGTTATGTTATAATCTGCAACGTTAGAATAGCAATGGTGGAAAGAACTTCAAATATGGGCTTCCAAGTCCCAACTCGGCCAAAATTGTCAAATCCCAAAACCTGGCCTAAATGCCAGACSAAAACCATAATCCTGATTCTAATTATGTTTCCAGATGAGAGAGAACCCGAGAAYTCCtttgttgagagagatggtaTTCAAGTAATTCTTTGATCTTCTCTCGGAAATAAATGAGAATTTTATTAAGGATAAAAAAAGCTTAAAATTTCAAgccagatttttttgtaatTTCTTGCAAAAATAGATTCAGAATTGATTAAGGAAATCAAAGTCCACGCCATTTGAACCTAAAGGAAAGTGAGAAATTTCAGTtattaaaatatcAATCTTTACCTCTTATTAGTACTCAAATTTTAAGAATAGTAGAAGCAAAACACATTTACAATCAGTCAAACTGTATCCACCAGATGAAACTCGATTCAATCATCCAAAGTCCACTAAGTTAAGGCAGATGCCTTAACAAGAAAATTTATTCGAAATTTTTAGTACTCTTACAAGAGGCACTTTCTATTTCAATGTCATGATACTTAGTTTACATCATAATTGATGTGCCACCCACCCATCATTTATTAATAATTATCAATCAGAACTTTTGTATGAAAATGAAATAAA

>Cs1g04850

ATTAGTRAAATCCYTCCAATGRGCCTTTGGTCCAGTGGRARAACYCTTGCATTTACAATGTTGAGTGCAAGGGtTcgagcctccataaaaatatttatgggggttatctacaatcctctttcaattattaaataattgagtgaagtcaatctatccctcacgaaatgtgagtggagtggacagttctcaaattttttagagggtttaaagaatctgggcagcgcttcagaggagtacatgccatgaagaatgtcccaattgtagaatatgtttgtaaatattaattgtaatacatgatgtatgttagtggagtggacagttcttgaatatttgagagggtttaaagaatcttagcagcgcttcagaggagtacatgtcatgaagaaggtctcaattgtagaatctgtttataaatattaattgtaatacctgatgtaatatcagtaacaGTCCTGTATAACaatatataaaaaaaaaaAGAATTAGTGAAATCCCACTGAAAAATAAATCTTACATCTCTAAAAAGGAAAAAATTTATCATACTTGAAAAATAAATAATCGCATCTCACCAATATAATTATAATTAATAATTAGCAAGAAAATTAGAGTTATATATCAAATCTTAGGGTAAAATAAAACACAAAAGCTCATAATATACTTCACTTTTTCAGTTTATCACTATCATTTACTGTCTTAACATTTAGTattagctgcagactgcagttgatcAAAAGATAATAATCATTTAATTAAAARCYTGACAGCAAGAGACTAAAGTagtattattatttttttTTTTTGCCCCTTTTGAAACAACCAATAACTTCATTATAATCAAAGATTAAAGTGGGAATTAATAATTAGYAGTTMCGTGTTAATCAAGAAATAATTACCTGGGCCGCAGTCACCCATCATATATATGAATAAATAGACCCAACCCAAAGCTCAAACCCAACATGATAAATCACACGTGTGCTACACACGTGCATTAGGGTTTTAGG

>Cs1g24790

AATGCCAAATTGTTTTGAATTCACAAGAGGGGCCCTTTTCCCCTTAGCCCTTTCACTCCCTAAGAAATGAAAGACCACATCTAACTCGGCCAAAAAATTGGAATAGGAATGAGATTTACAATGCCATCATTGTATGACCGATAGTAGTACATGGTTGAtttggaaaaaaactctttTCTTTTTTAagtcgtttgttaagCTTCCTCTCCTTATATAGAGCTTCCCTTCAAAAGttctcaataaattCACTTCTCTCATCGGCAAGAAAAATATATGTATGTATAGAGTTGACAATTtaagggcttgaagtgtaagggttgggcttagacttttacttattttttcaaattttttaaaaattattgtaaacttgaaatgaattaataatagtcaagttgttataatcaacaatgaattAAAGAAAATAAATATTTCAAAAATAAAATATAACTAAAGAAAATGAAATAAATAAAAGTTTATAAAtttggtatttttgtattgaATTCTCTAATTCACGGACTCTCTTAATTAATTAATACTTAAAAAAGAGCTTAGCATTATAATTTTAAGTATTTTGATTCATtattgatatatatataaattaagGAttcaagCTttcttttttttttattatttttcaaatttgaatttattattctaatttataaatttattcttaaaaaaaaaAagtgtttggacttagctcaggttttttctcttaagcccttgtctcgaccctgtgcagaggcccggggcaaagcttctagaaatggataggATCAACCTGAGCTTTTTTTGCCATCCCTACACACCCATCCCAAATATGTAGGTGTTGATGACCATGATATTTTCAAATAAACAATTATTTTTTAAATTAAAACAAACAAAAATTTTAAAAAAAAAAATAAAATAGCATAAATATTAAATAGACAGGGTGTTTTTGGTATTTAGGGGGTGGATTTCTAAAATATATTCATTTTAAATTTTTKTATGGGGGTGGG

>Cs1g09740

attttgttaaaatcaagcaaaatattgtttgtacaaaaaaattatggtttgagtatatttatttacttaagagaacacttgcatccaatcatcgtatcaaattaatcattcttatttatttggttgtgtgtttgtttgtnnnnnnnnnnnnnnnnnnnnnnnnnnnnnnnnnnnnnnnnnnnnnnnnnnnnnnnnnnnnnnnnnnnnnnnnnnnnnnnnnnnnnnnnnnnnnnnnnnnnnnnnnnnnnnnnnnnnnnnnnnnnnnnnnnnnnnnnnnnnnnnnnnnnnnnnnnnnnnnnnnnnnnnnnnnnnnnnnnnnnnnnnnnnnnnnnnnnnnnnnnnnnnnnnnnnnnnnnnnnnnnnnnnnnnnnnnnnnnnnnnnnnnnnnnnnnnnnnnnnnnnnnnnnnnnnnnnnnnnnnnnnnnnnnnnnnnnnnnnnnnnnnnnnnnnnnnnnnnnnnnnnnnnnnnnnnnnnnnnnnnnnnnnnnnnnnnnnnnnnnnnnnnnnnnnnnnnnnnnnnnnnnnnnnnnnnnnnnnnnnnnnnnnnnnnnnnnnnnnnnnnnnnnnnnnnnnnnnnnnnnnnnnnnnnnnnnnnnnnnnnnnnnnnnnnnnnnnnnnnnnnnnnnnnnnnnnnnnnnnnnnnnnnnnnnnnnnnnnnnnnnnnnnnnnnnntctgctatttaccccgggtcaaaccccgaaagctaaaacaaaacgacgccatttcctcttcttcatgaaacgctaccgtttcaccctagcaaaagcaaacgcttctctcctctttgttttcctcttcctctgctcttcgtttcaccagggaacgacataacccaatcctcttccactcctcttcgtattgttcgttttgtttaaaatccaannnnnnnnnnnnnnnnnnnnnnnnnnnnnnnnnnnnnnnnnnnnnnntttgagggcaaattaagattttcaacacatccacagaaagagagtgagagtggtgagtgagttatcaaaaa

>Cs1g13180

ACAAATTTGGTGYCTAGGGYTGTCTTAATAATGTTAACAAAACCAAAAAATAAAATTTTGAAAATTTTGCAACACTTTGATAATTAGAATGCATGAAATTTTTAAACTTCATTAATAATGGTAAAATGAATTAATTATATGTAKATAGAATTTTACAAATATCTTTTTTTTTAATAAATAAYTTTTCATCACAATATAGTGTGAAAGACTAGGATAATGTATTGATRATCTTGCAACTCTCGTATATCACGACTTTCAATTTTTTCTTCACARTTTTCGAATGATCGATATAATTTTTCAAGTCACACGTCACAGGATAAAATTTCTTAAATATAACAACAATTTTAATGAACATAAGGAAAATATTAATGACAAATGCTTAAATTTCAAGAATGATTCTACATTTATAAAGTTTTGATACAATTGTAACATCTCAKATTATGTGATATTTAAYGTGTCATTCAATAATAAGCGTAAATAATTGTAAGATTAATTATAGTTTCASGTTTCATTCAATGACTAATGCAAATAATTGTCAGATTAATCACATAAAAGTCAAAWTTYATTTAATACATaagtcagtcattAGTCTGTGATATGTTAGTCAAGATAAGAACTTTATATTCCAGTTTTAATATTATCATCTTTATTTAGCTAAAATGTGAACTACAATTTACAAGATTTGCAAACATGTTGAAACCTTATTTGATATTGAGGCTAATAACTATAGCAAAAARTTACAATACTAAAACATTTAGTAAACATTAGTTATTTTAGTTTAAAAGTTATGTTGATTTGATTTTATATTTATATGAGAATATTTTTATTaTAATTTTTCATAATtttttaattcttcGGCAATTAAATCATAAATAAAAATAATTTGAATTGCCGTAAGTTACGGATGACGTAACTCTATTTAATTCAGAATTAAAYACTGAGACGGGATGGAGTGTTTGTTGCTTAACACMGTATTTAATAAATCAATAAATTACCGATGA

>Cs1g26100

GCCCaaAaGGCTAAAAAAaGGTTtcggtttgcannnnggCTTTCGCttTTCATTTTTATTTGGACAAATAGTGAAGTTTCGCAtaaagtttagttacTGATTGGTCGTGGATAAGATACCAAACCCCTGCGAATTGAGTTTTTAACTTTTGAGAGTCGGACATGGAATGTGTGATGGGCCTACCAACAAAACCTCGGTAGTTGGGATCTTTTTTGGGGAAAAAAAAATATTTGTTTTCTCGAAAGAATatttcttttaactgcCAATTACACTTTTTCTCGCCGAGGCTGATCAATTTTTTTCTTTTAGTATAATTCAAAGAAAAATTCACATATTCCCTATGTCTTgatacttttttttatttttTTAATAATTGTATCATTACCATTTGAAATTTCRAATATAACCACATCAAAAGATTTTATTAACTGAACCAGTAATTTTATATGAAAAAAAGGTCTATCTGATATCTAGAATTCCAATATTACTATAWCATACGTAAAATAAACTTATTTAAAACCACTTTAACCAAGTAATCACATAAATGCTGGTGTAATTAAAAAGGTAAGACAATGTGTAAACATGTTTATATAAAATAGTAACATATGTATTAGATTTTGTAGTATAATGGTAAAATTCACTCACTTCCTATGANAGAATGAATTTGTTaacttttttttttctatcCAATAAATTTATGATTGTAAAATACAATCGTAATTCAATAAAGAAAAAAAAAAAGAAAGAAAGGAGCGTCAATGGCTCGGATTCAAAAAGaaaaaaaaaaaaaagaacaagaacaaCTCATTTTAAACCATTGATCTCATTTCCTAGTAGTCATCGAGGGAAAGATGTGAAGCCTTGCACGCACTGCYGGGGACACTGGAACATGGAAGTGACGGTCAAACTCGAACACGccagccagcgtacctatgcggagagaaaaaaaaaacaaaAataataaaaaaaataagggACAAGGAGGGTGAGTTAAACTCATT

>Cs1g22100

TTTAGTGAATATGCAAGATATTTTTTTCTTTCTTTTTCCGAAGGTGCTATTATTATTATTTTTATGTTCGGTATCCTTTTGCTCAAAtgtcttaaaagataaGTGTATTGCAAGGTCGTGTCTTACTTCATTAAAGATACAAACTTTTGTAACTCAATTTTGTAAACATATCATTAgtcttttgttaaacgtgcaaggcannnnnnnnnnnnnnnnnnnnnnnnnnnnngcattgctaGTGGATTTGCAGCTCAATAGTGGGCTGGGCTGGTGGTCTTAacaatattatgacacGAAAAKTGTGAAATAGAGATTTGTGTTTAGTGTGATTAGCGGGAGTAAATTTTTGATCTACCGTtgttatattaatTACTTGATAAGAACATATGAAAGTAAAATGGGACGTGTTCGGCTTATATATGtaatcgctggatgtccatattcatttttttaaaatataaatgagaaaaaaattATTGATAAGAAAAAGCAGAGAAAAATTACAAGTTAAATAACAAAATGANACCCAGACAAATCTCAACCYTCTTTTaaaaggtttttttttttaattggAAATTGATGAATAATTCCAACAAGTGTAAGAATTACAAGATTCTAAGTTTGCCTAAGAGTCAACAACAGACTTGTCRCTTTCTTTAGWAAATAATGACAATTAATCCATCTATTGTTAAGAGGACAAGGAGGGTTAGAATATTAATTACGAGGCASYAAAGCTSTCTAACCATACTCTAAATCAGCCTcCTTTATTAGAAAGTGTAGTTATGATCGGCATAAAAACGACGASACGCGACAAAGACAGAGaagcggaaaagcccaTCCTGTTGGTCATGTAGCAGAGCTGAACTAAATGAATCTATGATCTGTTTGATGATATTATTTGAATAATATGATTATTTATATTTTAGAGAAGCCGTGAACCAACAGGAAACAGAAATATTATGAAAGTTGAAAGGTAGCAAGCGACTTGCCCAAAAGATTTT

>Cs1g22150

GGAAACTACTCAAATAAATCTTTCTAGAATAGAGTTTAACATTCACTAAATGAGCATAAATTCTTTGGTAATGATAACTCCTYGCAATCTAGAGAAAGTAATGGTGTTTGAGAATCAGTGAATGTAAAGCCTACCCTATTGTACTGCCAAAATGGTCCACATCTGATATTATTTGTACTACACCATATCTTTCATAGACCTTTTGAGGCGAGAACAGAGCATCAACTGTACTGTTTTATACTGGTATAAATTAGGAAACCCAACCTTTGAGAGATCAACACTGTGAAGCATATAGATGAGTGGTCCATAATTTTGCAAAATCCTGCATGggaagatttgagtaCTGTGGTATGTGATAAATAGAATAGAGCACATGATGCTTGGGACCATTTATTCTAGTGATACTAGGTGTTTTATTGATGCAAGTTTTACGCCTTTAAATGGTTAACCAACGTGATTCTTTTGTAGGTAATTATGTTTTCTTGGGCCTGCTCTAAATCCTTGATATGTACAATTGTTTACGAGTTACATGTTGAATTTAGATCATCAGAGTTTATAGCTCACCTTCACCATTAATCATTCCTTCAATTTACTTCGTGTTCACTAATGTTGTTACGTAGTCTAAAGCGTGGCCTCTGAGCAAATTGATTGCAATTTATATAGTTGGACTATTAATTGCTCTACTGTTAGTTAAGCTAAAGCTTCTGTTAAGAAATCATgtatgatgccatagaAGGTTCTTTATAAAGGTGAAAGTATGGAATCCATGTGTTGATATAGACCAATACTAAAAGTTGACATCAGCAATTCCCCTCAAATGATCAAGCATCACTTTGTGGGAACACCAGATAGAATTGAGAGTTGACGTTAATAATATCCAAGGCCACTTGCATTTCTTTTTAATGCTTTTCTTTCTGTCCATTAAACTCCTTAGTGATCTATCCCTATTTAAGAGCAGCACAATAAAAAACTKAACTTACATATACCCTTCTGATTGGAGA

>Cs1g21320

GAGAGCCTGCTGGAGAGTATGGTAAAGCTAGTTAAAGAGAAGTAATgaggttaaagagttTTTTGCAGAGAAACTGGCATGGCATGGCATGGGTTAATGGTCACTTTATTGGCGGCAAGTTTTTTACAGTCTCTCTTTCTCTCAAAAAAGGAAAAGAAGCCAATTAGCTTTCAAGTCTTATGCATACGGTGTAGCACAAACACTGCCTCTGTGGGCTATGGTCGCTCTCAGATGAGTTGCTATCCACAATGTCCTTTTTCTGATTCTATAATCTTATTTAGATTTTCTGTTCCCCATTTGAAATACCAAACAAACCCCATTCTTTGAAAAATTTATGTGCATTACTCACCTCTCAGGTTATTTAATATTATTTattacatgtgtgtgttttttttttttaaatttCTTTCTCTTTTCCTTATTGTAAAGAAAAGAAAAGGGGAAAGTTTTCTTACTTATTTGGGAAAACTGTTAAAAGAAMACACGCCGAAGAACTCCTTTTCCAttaggcttcagcaAAATGAGTCAAATTGATATGCGTCTTAATTTTGTTTACAATCCTTTATTCCCATATCATAGCCMGATGATCACACGCTTGATCGTCATTTATACACAACCACAATTATAAATGCGGTGCTCTGTTTGGTCGGTCGTTTTCATTAATTTATTATTGAAGGCATCGCCCCATGTTTCCCATTGGGAGAATAGAAACAGCATGCTTTAAACCTTTTATAGCCGGTTCCTACTTGCAGTAAGCAGRGTCAGTTGGSATTTTGTGACTAACCTAATTGCAGAGTCCAAATACAGAGATGGATTCYCACTTGACATCTTTGCGACATCAATGGACTGGCCTGGCCGGAGCCGGCCAATTTTGATTGAGACAATAAAGCCCTTGAGCATATGCTAAAGCATATACTTATTGGCGGATGTAACACGGTAACTCTAATAGCTTATAAGGATGAAGAATCTTGCTGAACAAAATTTAACGAACAAGAAGAAAT

>Cs1g25820

AATTTGTCWACATACGATCATAAATAATTCAACGGTTGATTGCAACATAGCTATATTCATGCACTTAAGAAGCAGCTTATGCTTATTTGTTtatttattttgttttaattacaatacACCCCACCTTTTCTCAATCCTGAACTAAATGTATCTTATTGGCCTTCGGTCTCTTACTACAATAGTTCAATGTAGGAGATAAACAGTTTGCGGTGTCCTTCGGAAATTAAGCAATTRACTTGTAATGGACTAATGGATCATTATTCTTTGTGCATACGTGTGGCTTCCCCAATTAtcacaaccccccaaaaaACATGAATCTTCCCATCAGCTTTCACAAGTGAAATCGTGTCAACAAGTGAATGGAATTGAATTGTAAaaggtgttttttttttcttcaTGACGAAATCTGATGAGTCMTGTTCTAAGTCGTTTTCTAACACATAATTTATATTTGATAATATTAAAAGcatgaaccccccaactgCTTTTTTCTTTTCTTAGTTATTATATCTATAataattATTAATTATTAAATTTATTGCAAGGGCCGAGGGACCATCCCATCCTCATTTATAGAAGAACATGCTAGGTTATCAAACTATATATTTTGGATTATTTTATTYTGATTGAAGGGCCCATGTAGATTGTTATTAAATCCTCCATCTGTTTTTATGGATTCTGGAACTATATTTGGGTCCAAATGAGTTCTGTTTTTTTTTTTTTTATATCCAAATCCAGCCCATTTACTTTCATTccttagtccccaccttatcaaagtgttgcatttgcttttgaaatagttaaatAGTTaaTGCGTGCTGCTCAAAGCAAGGGTCCACTGGGGTTCCCCCACGAGGGACGACGTGTGCCTTCCAGATTCTTCCACGTACTCCTTCCGAGTCACAAGTGTCAGTATACGACACAACTTACACTCTCATCTTTCATCTTTTAAGTCATTAATATATATRTCCTTCAAATGATTAATGATGTCCTAGcattag

>Cs1g08780

nnnnnnnnnnnnnnnnnnnnnnnnnnnnnnnnnnnnnnnnnnnnnnnnnnnnnnnnnnnnnnnnnnnnnnnnnnnnnnnnnnnnnnnnnnnnnnnnnnnnnnnnnnnnnnnnnnnnnnnnnnnnnnnnnnnnnnnnnnnnnnnnnnnnnnnnnnnnnnnnnnnnnnnnnnnnnnnnnnnnnnnnnnnnnnnnnnnnnnnnnnnnnnnnnnnnnnnnnnnnnnnnnnnnnnnnnnnnnnnnnnnnnnnnnnnnnnnnnnnnnnnnnnnnnnnnnnnnnnnnnnnnnnnnnnnnnnnnnnnnnnnnnnnnnnnnnnnnnnnnnnnnnnnnnnnnnnnnnnnnnnnnnnnnnnnnnnnnnnnnnnnnnnnnnnnnnnnnnnnnnnnnnnnnnnnnnnnnnnnnnnnnnnnnnnnnnnnnnnnnnnnnnnnnnnnnnnnnnnnnnnnnnnnnnnnnnnnnnnnnnnnnnnnnnnnnnnnnnnnnnnnnnnnnnnnnnnnnnnnnnnnnnnnnnnnnnnnnnnnnnnnnnnnnnnnnnnnnnnnnnnnnnnnnnnnnnnnnnnnnnnnnnnnnnnnnnnnnnnnnnnnnnnnnnnnnnnnnnnnnnnnnnnnnnnnnnnnnnnnnnnnnnnnnnnnnnattgtaatagtGATTTGGCTTCTTAATTTTCTTTGTCGGTACTGCATTATTTCCCATTCAAAAAAGCTTTCAAGTGTTTTAcaacatcatcaatctttttctaacaatatgtctgctatatttctcatttannnnnnnnnnnnnnnnnnnnnnnnnnnnnnnnnnnnnnnnnnnnnnnnnnnnnnnnnnnnnnnnnnnnnnnnnnnnnnnnnnnnnnnnnnnnnnnnnnnnnctagctagttctgaaagtttacttttaattgtcaaattattttcttagcacagtctactaaaaaaagaaacaaaagaaataaaaaagaagtagcttgttaatgactgctctgtaatgtctatttttcatgttaatt

>Cs1g16760

ctctccccatgggagtcccactcccactccccactccaaaaatgagtggggcccacgaagtaggattctcaatccttccctaattagttgaagtaaacactggagtgggaggaatccacactcctcactcccactccagaaagtaaacacaacattagtgcagtcaagtcttccattctnnnnnnnnnnnnnnnnnnnnnnnnnnnnnnnnnnnnnnnnnnnnnnnnnnnnnnnnnnnnnnnnnnnnnnnnnnnnnnnnnnnnnnnnnnnnnnnnnnnnnnnnnnnnnnnnnnnnnnnnnnnnnnnnnnnnnnnnnnnnnnnnnnnnnnnnnnnnnnnnnnnnnnnnnnnnnnnnnnnnnnnnnnnnnnnnnnnnnnnnnnnnnnnnnnnnnnnnnnnnnnnnnnnnnnnnactaacagTAACCAAAAGAATGATGCACTTTTTTATAAGTTCACATTTTCCCATGGTTTAAAAATATGCGTGGTATTTCAATTAAAATGAAAAAAAAAAGATATATTAAAAGAAAAACAACTAAtcaatccatataaacnnnnnnnnnnnnnnnnnnnnnnnnnnnnnnnnnnnnnnnnnnnnnnnnnnnnnnnnnnnnnnnaagtaataaagccattatatatattACAAACTATATAATAAATAAGAGTRACTATAATCCTCTTAGTAAAACTAATAATTCAGCTCTTACATTCtgacagttttgaaacAAATTAATCCATCAGAGAATTTGATTTTGGGGTTATAACAATCAAATTATTGCACTGTATTAAAAAATTTTGACGAAATTCAATTGCAAGACGGTGTAAAAAATTTGCAAACTGTGgaatwcttataATTTTTAACAAAAAGAGGGATTCAAATGTTAATTAGTCCTTTAAGACTTGGGTTGGGCTGTGGGGTTGAACAAACCCACGAGCCCAAATAATGGGCTGGGTGGGTTTTGTTTACCCGCCCGTTTCCAGAAAATGGGACCAAAAGAAACTAGG

>Cs1g18590

GGTGGATTTATCAGGAGGTATTCGCAATCGAAGAACAGCTCGGAGCAAAGAGATGCAAGCAGGATCCGTCTCCTATCAAACAAATTCAAGTGCTGagatagtcctctagtcacaacacaactcaactaggtctcagcatcaacagctacaatttttttttttnnnnnnnnnnnnnnnnnnnnnnnnnnnnnnnnnnnnnnnnnnnnnnnnnnnnnnnnnnnnnnnnnnnnnnnnnnnnnnnnnnnnnnnnnnnnnnnnnnnnnnnnnnnnnnnnnnnnnnnnnnnnnnnnnnnnnnnnnnnnnnnnnnnnnnnnnnnnnnnnnnnnnnnnnnnnnnnnnnnnnnnnnnnnnnnnnnnnnnnnnnnnnnnnnnnnnnnnnnnnnnnnnnnnnnnnnnnnnnnnnnnnnnnnnnnnnnnnnnnnnnnnnnnnnnnnnnnnnnnnnnnnnnnnnnnnnnnnnnnnnnnnnnnnnnnnnnnnnnnnnnnnnnnnnnnnnnnnnnnnnnnnnnnnnnnnnnnnnnnnnnnnnnnnnnnnnnnnnnnnnnnnnnnnnnnnnnnnnnnnnnnnnnnnnnnnnnnnnnnnnnnnnnnnnnnnnnnnnnnnnnnnnnnnnnnnnnnnnnnnnnnnnnnnnnnnnnnnnnnnnnnnnnnnnnnnnnnnnnnnnnnnnnnnnnnnnnnnnnnnnnnnnnnnnnnnnnnnnnnnnnnnnnnnnnnnnnnnnnnnnnnnnnnnnnnnnnnnnnnnnnnnnnnnnnnnnnnnnnnnnnnnnnnnnnnnnnnnnnnnnnnnnnnnnnnnnnnnnnnnnnnnnnnnnnnnnnnnnnnnnnnnnnnnnnnnnnnnnnnnnnnnnnnnnnnnnnnnnnnnnnnnnnnnnnnnnnnnnnnnnnnnnnnnnnnnnnnnnnnnnnnnnnnnnnnnnnnnnnnnnnnnnnnnnnnnnnnnnnnnnnnnnnnnnnnnnnnnnnnnnnnnnnnnnnnnnnnnnnnnnnnnnnnnnnnnnnnnnnn

>Cs1g04640

aataaaaataacacaaatttatcacaataatgttgtcaattttggactttaatctacatgattgaaaagtgtacaagaaaaatatagctaatttattaagatgatattgtcaatttcggacttctaatctatgctattcaaaagaaaattaagtaacaaagaaaaagatattagttaaagtcaaaaatattttctttttcccattttttttaataatatgaatagaatgtgagaattaggatttatattagtaaacgatgagttcttataaataaaaattgaaagagaacaaannnnnnnnnnnnnnnnnatgaaaaaaaaaatctatcaatgatttttaaatttaaataatataattaaacataaatttttttttaagaaaAATTAATCCCaAgcgAGACAGTGGTCCCCACGACAACTTGGACAGACAGATTTGGGCCTACCCAATTTACCGCCAATAAACTATCGCGAAGTCRCAAACAAAAGGCACATTGACGCTCTCTTGTCCTTTTGGCGGGAAaattgaaaaaaataaaaaAACCAAAAATTCAAAACCCTAATTGCTTCCAAATTTGAAATGCAACATTTCATCTATTTAACCGGCCGCCCCTTCTCATTTCTTCTCGCGCACACTACATGTTCGATAAAATTCCTCAGTGAGCAAAAAAAGTCGAtaaccaatcaagaatcaagatgtacgTCGTGAAGAGAGATGGAAAGCAAGAGGCGGTCCATTTTGACAAGATTACGGCGAGACTGAAGAAATTGAGCTACGGGCTAAGCATCGAGCACTGCGACCCGGTGCTTGTGTCCCAGAAGGTGTGTACCGGTGTCTACAAAGGTGTCACCACCAGCCAACTCGATGAATTGGCCGCTGAAACTGCCGCTGCCATGACCACTAGCCATCCCGATTATGCCTCTGTGAGATTCATTAACTTTACCTACAAATTGGGTTTGGTTTTACAAGATTTTGTTTGTTAGTCAATTTAGTGAAAATGGCA

>Cs1g25430

nnnnnnnnnnnnnnnnnnnnnnnnnnnnnnnnnnnnnnnnnnnnnnnnnnnnnnnnnnnnnnnnnnnnnnnnnnnnnnnnnnnnnnnnnnnnnnnnnnnnnnnnnnnnnnnnnnnnnnnnnnnnnnnnnnnnnnnnnnnnnnnnnnnnnnnnnnnnnnnnnnnnnnnnnnnnnnnnnnnnnnnncgtcacagttttggtcacagCCATGACCGTTGGCCTTTGACATGCGGAATGGTTGTCACGAGAACAACCTAAGCAAAGKTATCATGAGCTAATAGCTATGGRTCCAAGTCGTTCCCTATRTTTTGTAAAAGCTCTCTTGGGAGACGGAGAGCGCATTTACATTGRGCTCTTCAGCGGAAATTGCACATATGTAAGAATGTCAAATCAGTATGTCCGGTGGTGAAATTGAGAAACAACACTAATTTGAGCATGATCACTGTACTCGGGACTAAATATTAGCTCATTTGTTCTTGCGAAAGTGAGCGTATAAGATGGGGTAGAAAGAGTCCCGACAACTAGAGCATCAAAGTCGTGTCCGCTCATGCTTTTTTACAACTATAAGAGAATCCCACCCAAATTTTATTATTTTACTTTTATCCTCAATTAAAAKATTATTACTATTTATCAAAAAAAATATTATTACTAAAAATGAGGACAACAACTTGTGCTTACCACAGCCACACCATTCTCTGCTGAGTCCCTTTTCTGTGTGGAGCTACTAACTTAACTAGTTGATTAATGAGTTTTTTAAAAATARATTATAAAATTAATGAAATAARATTATCAAATAAATAAATAATATTTTAAATAttttaatatttaaaaacAAATTTTAACTACTATTCTCAAAATTCAAAATTTAAATTTTTATTAAtaaacacaaaataaTTTATTTAATCTTAAAAGTTCTACTTAACAAACAATCAATAATAATAACACTTATTATTATTGTAAGTCATTCCAAACAGTCGTAAGTTTAATTTGATAATTTTAATAA

>Cs1g23110

TTAGCTTAGCAGTAAAATGACCCAATGTGTAGCACACGAAGGGCAATCATTTTGGCTCTTTAGTTAACAAAATTCAAGCCAAAAGTATCGGGCAGTGGCTTTCTAGTTGGGTGTAGGGACATCAACTTTACGCGTGATTCCTTGTGATTGGCCCTCTCCTTGAACTRGTAATCATGGTTTTMAACAGCATCTAATCTTCATCAAATAAACTCAAGCCCGGCCCCCAACAAAGATTATTCTTTTGGGAAACTTGATTACTAGTATCATTATATATGATGCTCACTACCGATCTACACTCTACGCTCTACAGTCTCCACCACTTTCAGTGCTTCATTTTATAAAATGATTTAARAAATTCCATTATAATACAGTGCATCACCCACTGGAAGTCATCTTGTTTCAAACAAAGACACCCACAGGATACACTGGTATTGCAAACGAAAAATGATCTGATGTTTCGATCAACTAGCTCGTTCACAACARACACAATCACGAATAACGACATAGTTTTTACTTTAGTATAGATTAAATAGTAATTTACAGTTAATAAAAATGAAAAATTTAGAATCGACAGCACATATACAATTAAATCTGAAAAACAAATATGCATGGTAAGTTTTAATCACCAATATAYTTGTTTAATATTCATTTTTTTWAAATGTAGATATTATGTAGATTTAAACTAAATTCTCTCAAATTTTAGTGCAATTAAGATTCAAACTCTAATGGGAATTTGCCAATTTGGTATTGTTTAATGCGAttattcaattttttttttttggaagAATATTACCAGGTCTTTTCGAATTYGACTTCTCACAAACAAGGAAAAGAACAGTATGATTATGATCAGAAGGGTAGAATGGGAAATGGAGAAAAGGCCAATTACTTCCCTCCAAAAACCACTAACAGCAAAATTCCAAATCGAAGACCACTACAATTTTTTATATGGATTAATAAAGTGGCTTGCTGAGATTCTTTCACATAATTGTATTCCTGTA

>Cs1g23410

GAAAATGAAGCATTTATTTTGGTCAACAGTACAAATTTAATTTCCAATAGTCAAAATCATAAKATCCGGGTGAAAACCCTAACCTGATCTCCAAAGCACGGCTAAAGCACTCAGCAGCCTCGCCGTAATCACTTTCTTTCAAAGCATTGGTTCCTTTCTCCATCAACTCGWCTGCAAACTCTACCGTCTTCTCCCGTTCTCCGTCGGCGATGGCACCGGAAGTTTCACAGTTGTTGTTGCATGTCGACTCGGTACCGCTCACAGTCACAGATTCCATAGTGGCTTCGACCGAGGCTTGGGTGGTTCCTACTGTTTCTGTGGGTTGCGCAGTTTGCTCCGCCACCGTCTGTGAACCTTCTTCTTCCGCCATTTGCTTCGTTTCGAAATTTGTTCAAAACGGAATGGCCGAATAGGAATAAATATTTTTATTAGACGCCGGATTTAGCGAAACGACATCGTCTCMTAAGGCCGTATATGTTAAAAAGTGGTTATAATTTTATTTATATTaccccGAGAGCGACAAAACATTAAGTTCTACTATTCCTAAGATCCTATTAAAAAATAATTGTTTTACGCAAATCTTTCTATCTCTCCTTTATAGAAGTACTTCTTTGCACTATTTAGaacaacagattgGGAATGAATTTGTAGTTTGAGAATTTAAAGATTTATTAGATTGATCAAACTGAAAAATGAAAATTAGGGTTTATAATTATATAATTATATGTTGAATGAGTAATATATTTTTTGGAGTTATTGAATATAGTCCTCAATTGTAAACTAATATTGAGATATTTTAATAATATAAAATTTTATTAAAATATGAGTGTTCAAACTACTTTTTTTTTGTACGAATAGTGCCCCTCctatttaaatAATTGATTGATTTcTTTAAAAGATTACtcatattaaataaaaaaaaaAAGGTggggGGTGCAGGGATATTTGAAGGTTAAAATAGTCTCTTTGTTTGAAATACAAACATACAATCCTTTTTCCTT

>Cs1g25800

GGCTGGTAAAGCTGCACTGCATTGTTTATTCCAGTGCCAGAAAGTGAGAAAAGTTTGTTTCGTTTCCAGCTTGGGGTAACTTCTTGTGCGGGAAGATAGTTGTAGTGAAAGTTGTTAGAAATTGATGATATAACTTCTTTCATGGAGGGAACAAAAAATCGTGTGCCACAGCTTCATTTTTTCTtatttgaaaaaaaaaaaaaataaaaTAAGAATCTTATAGCATTTCAGAAATTTAGGTAATGTGTAAGGTTTCGATTTTTGCAGAAGGCTTGGACGATCTTGTAAGACTGCCTCTGAACCAGGGCTAAGCAAAGAAGGTGCCTGGCTCCTAAAATGAAGAGAtaggaatygtctggtctggtctggtagtagGAGAGGAgtaaccgtgtgtgtagaagAATCATGCGTGTGATGCTCCAGTTTTTGTTTTYATAAATTGGTGAAACGGTGCTTATGCATGTTTGTTCAATGGAGAAGGAGATTTGTATTTAAATATTAATTTTGAATAGAGTAAAtaaatattgtcaaTATATTTTCCTTAACGTAGACAATTGAATGTCCAAAACCCCGAAGGCATTCAATGGCCTGCTATTTTCCTTCATTTTTACCACCGTGTTAAGAGAATATTGATATATTGCCACAAATTTCTCAGGCTTGTGCATTATTTAATTAGCTTTTGAATGATTTTGTTGCCCACAMTAATAGAGAAGATACAAATTTACTTTTATATAAATAAATTGGGAGGAAAAATGAGTCCCAATATTCCATGCTCTCAATTTACCGAATACCTTGAAAAGGTYGAATTTAACAACTTAATAAGAAATTAGTATAAAGTACAATTTGGCGTAAAGTACACGACCCAGAATCAACGGTCTAAGATTTATTCAGAGCTTACACGTCAAGCGCACAAGATCACGCCGATCCAGagcgtctaaaggcAATTGAGGATCTTACTACGTATCAAGTCAAAACGCAACCTCgttaacgagagagagaga

>Cs1g19770

ggccaagaaagtcaaagccgtgggtatcaaaatcttcttcaaaaccgtgttcatcaaagcttggtgggtgaaaaccgtgttcaattttcaaagccatgtgccaaccattcttgtcaaagcttctagatgcagccatgccaannnnnnnnnnnnnnnnnnnnnnnnnnnnnnnnntcaattttgacAAGAAGAACATATCACTCGGGATTTGTCGAAAGAGATATGAAAAACTGGAAATCGAATATATGAACGCTGAATGAAAGGGTGTTCTATTAAATAATCTATTGATATTTACATCAGATTTTTTTGAGGATCACTTTAAAATCAAATCTATTACTACACAATTTGTATCACACATCAATAGAAAGATGCCTATTGCTAACCAATTTTTAGTTGAAAGAGGGAACTATAAGAGTCCCTAAGATTAGACTCAAACACATACAGTGTTAAACGTAGTATTTTATTTAGAGTGGGACTATTAGCACTTTGTAATTTTATTTTCAAACTCCTTTTCTATCAAAACCTACTTTAATATCTTtttttatttttttcagttTTTTTAATTCAAAAAGAAAAACAATTCAATTTCTTCCAAACAACTCTAATTACAAACATTGTTTTTATAAATTTTTGAATTATTAAATCATACATATTttactcaaaaaaaaatcaaaTACATGTATGAATTAAGTAGGATTAAAGTGGACTCAAATTATTATTATTTATTTTTTAAATTAAAAATAAGTTTAGAAAATtTTGTAAATTAAaAATAAAtttACAaAActtatttttagaaagtttttaattgccaacatagtaacccggccgaatgcatacaccgattttgttaattattcgtcaggttctgtccagtcttttcataattattgtccagtctcttcaattattacttttcatgtgaaccacacaaatttcgggtgtcacaattttatatggtttacatgagaagtgtaagttACAATGATTATaacttctgagac

>Cs1g15500

nnnnnnnnnnnnnnnnnnnnnnnnnnnnnnnnnnnnnnnnnnnnnnnnnnnnnnnnnnnnnnnnnnnnnnnnnnnnnnnnnnnnnnnnnnnnnnnnnnnnnnnnnnnaaaaAAAAAAAAAAAAAAAAAAACTCCTCACAATGGTGTGTGTGTGGATTCCACGTTGTGCCCCGAATGTGGTTGGCGTTTAAACAACTAATATTTCCATGAAACGCAGGCCAAATGAAATTACAATAAACAAATACTCGTAGCAKATTCAACGAGCTTTTGATGATTTGAGTTAATCCACCCGATTTGAGGCCTAATTATGAGGACCTTGAAAAGTGGTTTTGTTGTGTGACAGSGTCATGCATTTTWATCCACTAACTTCATTTCCTCCAAACATGGCCCATCTTCTTTACATTCATTTTTCATTTTCTGATCCAAAAATATCATAAATATTAWATASGCAGCAATAGTTAGGAAAWWWTTTCATTATATTCGTAATACTTTCAATATTTAAARGAACATATTAAATCSATTTTTTTAGTATTCATCTCATGGTATTAACATTTTAATAATCTTTACCCGTGTGTAttttATTATAACGGTATAGTATCACaTTTTTTTTTAAAAATAAATAATGTTGGTGTAGCattcatctaatgaatgatataatagaatgaTAAATAATTTTATATTMATCATKTAKTAAATAAGTGATAAGRAATATTGAAATTAAAGTTGYCACTCAAATGTTAGAATTTRAAACATTATTTYTAGAATACACATAAAAATAAGGTAAGCAATaatccttacccttATAATAATTCCTGATAACTTCATTGTAATTTTTTTTAAAAAAATATGAAACTCATTGACCAAATATACATTTATTAATTATTCGAACAGTTATTGAATAAattaacaaaaaaaaatttcaATTTTTGAAAAAAATTATTAAAATAAAAATAAAGAGAAACCCCACGCTCAGAgcttcactcgtATATAAAGAACAGAAAC

>Cs1g16270

TGGATGtttttCTTTATTTAGCTTGCTACTGAGTTAAACTCATGAGGGATTGTATGAGTTAAAAAGTTATATTTGGCTTCTCCCAACCATATCCCTAAAATAATAATATTAGATTAACAATGTTACTAGTTAaaaaaaaataatgtcacAAATGAATTTAGTTTAATCAAATTAAtattagccaagtgCTAGCCATGATAGTTATTAACAACATtaaaagaaaaaaaaaaggcttAGTTTATGAATTATGATATCATCAATCACAATAACACTAATAGAAATAGATTAAAAATTATCATTACGTTTATAATTGTAAATTGCAAGTATTTATATAAATTATTGTGACTTAAAAATAGTaaataaatgtaatcatcaaaaannnnnnnnnnttatgaataaagtaattaatctcacataaATTTATATAAATTAACTTACAcaagagatttgcgAGTATTATTTAAAAAATATCAAACATTAAATATTTTAGACTTGTCTAATATACAAATATTATAGGTCTAAATATTTATGACGTATTGTACTATCAAaaaattgataaaatattttgataaaatttttatgtaaaacatcatnnnnnnnnnnnnnnnnnnnnnnnnnnnnnnnnnatTAATTTATTATTATTCTAATTAMTATAGAATTTTCATATAGTTTACATATATTTTAATATTATTCAggaaggaaaaaaaagtagtAGTATAGTTTCAAGTTAAATTGAGGGGGTCTTTCGTAGTATTTTAGTTTGAATTGATTAgtttgattttttttttaaatgAGGATGATTGTTTGATAATTACCTTTGATATTCTCTGTTTCACAATTGATAAGTGTGGCCGGTTACACCCGCAATATGAAAAAAATAACATAAGGCAGCCGCGTGACATGCCATGCTGCgctgtattactattGTTCGGCTACCCTTCTATCGCTATATCTGAATATAATAATAAAAaaatatcaaaaaagacaaAAAAGTTCTCTCC

>Cs1g21260

gtctttgcaacatttgaattaatcatctctggggaggtccctttttttgtagtgtgctattgttttatacgcgtgcgtgtgatgggtactgtctttttccatttttttcgtaaataaattttgtttataaaaataaaagcttttggcaaattaaatactacttccaaacctttcaccaaaaataataataataataataaataaataaataaaactacctcgattcgctatcccctcggttgattccatcacccaaggccaaggtgaagctagcaaacgccaaactcgagtatatatgaatcaatcaaataaatgatcatggaatcatcgaaacaaattttgtcttctctattttgtatttgtcgtctacatcgcccatcaatctcatttagtataaaatctaaatgtcatgattggcagcacaataactctacttctagagaactgcatacgtctgaatcatgagaacttttctgcttaacacggcaccaacgctaagtcattcattttatttctcatattcgctttgtttttttgcacaaaccattaattattctcttgacttccttgcctgctcgataagaatgtacgtaatgcaaatggcaatgccagactacgtcatttaccggcttcaactttcaatataaacgacaagccgaaccactgccgttctgtgagatagtcgtcttatgacttgaaaaacgtcgtccctttctatcagccgctatcaatcagtccccggccagggcgcaatcgctgctatccatttcaacactggtcccactaaatctaacggcagaccgttcactcgaaggccgttaccattttccacatcaaaatgagtatctttgccactctataaaaagaggggacgcccctccagattcttggctccaattttccaaattcagttaaaaccacagtgaacatcaattttataagtctgttaactctttggggcgaaaaatatcacgagaatgatggcatgttgcggcgattca

>Cs1g14950

ttctgatttcgagatttttattttcagtttgctgttttaaaattcaaacctatgcattcaaagtgtgaagggcaagctcattgactcgtgnnnnnnnnnnnnnnnnnnnnnnnnnnnnnaaacAATAattggaaaaaaaaaaaattgaacCCAAATAGTCAAACAAAGACAATACAAATTTGAATTCAGAATTCTATATTAGATGACTCATTTCAAACTtttgataagataTATAAGACCGTGATTAAAACATGTGACTAcaatcttttttattttcTTTTCTTTGATATAATATTATACTGCACATATGACATAGAAAATTTATTCAATTTCTTAGATCAACTCCGGAATAATCAATCCTGATAAAAAATATCAAGTGCGAATGTCTAAATATAAATATGGTATTACCCTATTCATTGTAATAATTAATTGAACATAAACACTATAATAATtgactcgaattgttgccatggacattggttnnnnnnnnnnnnnnnnnnnnnnnnnnntaaaatatactAGCACTATCTATCCCCATTATTTAACACGATAGGCACACATCACTTTACTTTAACTACAAATGATCGCAWGAGGCTTCACTTTACTTGCACATCCAACATGTCACACGTCCYATTGGCACAAMAAGTGGCACATCCAGTGGGTGATGCGTCCCAWTGTCSCATGGCCACACCAAATCAATAACTGTCCTTCTGCCGATGAAGCTGATAATATGACRTCACTACGCCTACCAATAATGTAAAAATTTCGATCAAAATTCGGACCCACCACCATGCAAAATTCCAAAACCTTTGGTTCGTGAATCAGCGGTGGCCTTTGCGTCATCCTCTTGCACYGATAGCAAAATCAATTGGYAAAACCAAAAAGGTGGTGAAACACTAGGCTAAAAGCACCAAGAAGACACAGTGCCGAAACGTGGCAACATGCTACTGGTGGTTTGGARTAGAAGAAGTTGGCCGTTTCCTCATTCACGCAACCCCCCA

>Cs1g26410

TTATGAGATGATTCAGAATGGCATGATTATTTTGGAGGTTTCCACTGTGGCCGTCCTAACCAGGTCGTTTCCCTGTTTGTGATTGAGCTTGTAATGTTGTTAAATGAATTTATTTTCTTTGCTACAGGAACAAGGCATAGAGTCAGATACAAAAACTTGTAATTATCCAAGGCTTATAATATTCGTATTCTGACATTTTGCCTGCTTGCTGCTTCTATCAGTCATTTTGGCTTaaaaaaggaaaaaaaaaactcgtAAATGCACAAAATTCTTGTTCGTTCTCAATATCAGCTCCTAGTGACATGGATGAGTAGCCTAAAAACCTTTATTTGACTAGTTACTTAAGTTGGAATCCTGAGAGAAATTTCTGTTGGATCACCTGCTATCCGGGATCAATTATTCGGATAAAGAAAAAGGATTGATTTGTTTATTCGATGGAAATACGCGTTTAAGAAAGAAAAGAAAAGAAAAAGGAAAAAGAAAAAAGAGCTGAATCGGCAGGAAATAAGTAATAAAaGTATTTGATGAACCGGCACATAGTAGTTGACCGTAGACCCTACCCTAGAGTACCCTGCCCTACAGAGAGCGTAGACAATGCAAGTCGTATTAGTGCCGCACCTGACGCGCCGTAAAAATACAAACCGCAGGAAAGCGAAACAAAACCAGCAATGAAATGATTGATATTTGATAGCAGCATCTCACACACACACACaCacACACACACACTCAATATAATTTGAAATCAGATGCGGCACGCCACATGCATTGCAAACCAAAGTTACCGTTGTACTTACACGAAGAGTCAAAGACAAAGTCATCGCCTCCCCACTCTAATCCAACGGTCACCTATCACTAGCCGTTGGTTTTGAAAATAATAATAATAAATCCAGACAAAGCATCCATTGTTttattttctctcctccaTTTTGTCTAAACACTTTAAATGTTCTTTTGAAGGAGGGGAGGGGCTGTTGTTTTAGAGGATTGTTTCTTctgaaaat

>Cs1g01380

TTAATGTCCATTAAGATTAATGTAATATGATGTTTACTAATAAAAAGAGATCCAGATGTACATTTTTAAGGTAGTGGGGTTTAGCTATGTATGACTAAAACTATGGTAGTCTATGAGACAAATTTTTGGTCTCAGATTTCGTTTCCTCTCGTATTTAAGCTTTGACATATCATTATAGAAGTTATAACCTATCTTGATGCTCAAATCTTCACATATGATTTGATATTTTGGACTAAATTTCTCAAGTTGAAAATCAACGCCCAATAGCTGGCTTGGCCACAACGTGCATGCCAATCACTCTTTATAAATATATTCACATTCTTTATCTTTAAGGGCATGTCCATTGTATTATTGGCTGTAAGCAAATTAAATTTTTTTAATCTAACAGAACACTCTCGCTAAGACCAAATAACAATTAGCAAATCATTTCCTCTCCTTTAAATTTTCTTTCTAAAAATTTACTGATTCTCATTTATCCAAAGACCGGGTCTCTTGGTTCCCTCTGTGCACCACGCAAAGCAAACACAAAAAGTAAGAGGAAAAAAATTCAAAAAATTTCAAAATTCCATTACCTAACTAATCACAAATTTAATTGAAAAATTAGCACTCTTCCCtttttctttttctttttttcttttTACCTTCAAAATAATTATGACATTTCTCATTGTCTTTTTAACCAAAAGAGAGAGAATAATTATTAAACAAAGAATCAATCTTTTTCTTCTTTAACAGCCACACTCCAGGAGGAAAGTTTTAACTAGCTTCCTTGCCTATCATCTCCTGGGGCTAAGGCCTGTTTCAAATTGATTTCCAATCCAAATCCAATCCCTTTTCTTAATTCTCAATCTTTGTGCTAATCATTTTGGGAATGCACCAATGTCTCGTCTATTTGTTTTGTTAAAAATACATTGCAATTTTTCATAACAAGAGCTGCATTTTTCTCCAATTTTCTTGGGAAGCTACTTGTGCAACAAGCCTGAAGATTCAACATCAAAGCA

>Cs1g21200

TGATCATAAAATTCCCAACATTTGACCTATATAGCACAATGCAAGAAATGGATTAGAATACCCTGTTGAGAAGATATTCCCAAAGTGGGGGTGGGGAGTCGGAAAAAAGGGAaaaaatgaaaaaaaactctatGGCAGTACTTTAATCTAATTGGGATAAGTCTCCCACAATAAGTCATGAggagattttttttgtcgttAATTTTCATAAAAATTTTTAGTCAAATATCTCTATTAACGGATAATTCATAAAGAATACAAAAATTACAAAATAATATCCATAATCTATGTAATAGCACATTACAAATGTCAACTGACATGCTAATAATAATACTAAGGAGACAGTAACGGATATACATATATAACACCAACAAAAATAACTGAACGACAAAATCATATTAAAAAGGATGACTTAGATGTCTATGCGAGAAAATaaaggatgacttatatatttatatatatatatatatataaGCAGCCATGAATATACGTAGTGAGTAGAAGAACCATTAATTTCAGCAGCTGGTGGTGAATTGATATATGTATAAACAATATATGGGTCCATCAAATGAACGATCATGATCATCATCCATTCCCATCACGTAACCTAGCAGATGTTTGATGATCCTAATGATAAAAGCTAACGGAGCAGATTGGAGAGAGAGACGGAGTCAATGGCAACGGCGGATTATTAGCACACatgtatgtrtrtgtgTGTGTGTGTGTGTATTAGGGTCAGTGTAGATTCTACACAAAGAAAGAAAGACAATGAGAAAAAAACTTGGTCTCCTTCTCTGGCATTTCTAATTTCCCCAATTATTTATttatatttataaaaTTAAACATAAAGTTATCCTCTTTTTCTCAATCGACTTTTTGAAATCCTGTGACCGTTCCTTGTCTCTCCTTCTCCAGCTActtattctctctctctctctctctctctctttttgaTTTCTTATATATATATATAtatatAAACACACATACACAATAGAGCA

>Cs1g23080

AACACCACTTGAGATAGGAACAATAATAGATGGAGGCACATATGCACACATAGTAAATAAAGAACACAGTAAAATAGGATTAGTAAAATGGTRCGGCGATCTCATACCGATATTTAAGTCAGCTAAAGTTTCATATCATATACTCATTTTTCTTAAAATCTcctagatgtaattttAcgtgttttaaaaaaatacATCTAAATAATTTTATTTATCTTACTTTAAAAAAATTTAAACTTATATTTATTTCAATCATTTCATAATAAAACGATACTATTACGATAAAATTTYCTTAATACATGTAAGTTTTTAATTTttttttaaactGATTAAGCATCAGGTTACTGTATTACACAGAGATATTTACAAATGACAACATAGCYTTGCACTGATATTTACAATCAGATATATACCTGAGACTTacactattacattttttattctataaagtacatgctcaaccaaatacccctcatgagagagaaatgtctctactccactcgcatttcgcaagggagaaagatattataagcaatcttcacacaattataattaattgaaagagtttgagtccgtagagactcgaactattactttcatagtcatacttaactttaaagataatgattcaccactggaccacaagcccaagtggctaTGTAAGTTTTTAATTATCAGCTTGTTTCACTGCAATCACAGTTTGGCCCTGAACAAATATTTACCCGACATATATAAAGGGGTTGTCAAAGGGATTGTCGTATGCGTtgAATATCTAAAGCAGTGGCATAATACGGGATTGTCCAGGGGACTACGGCtacttcaaaagattgGACTCGTGTCAGAAAAGGAAATGTGCCATTGCGTGATCATCTAACAAAACCAAAACGAGGTGCCACAACAGTGAGTGCATGGCGTCCACGTTACCTTCATACTTCCACACGTGTAACACTAGAAGAAAAGATTGAAAGCGTTCTCTCTAAATGAATTGGAAAATGGCGTTG

>Cs1g20500

atagATAtaattataaTACTAAATTATTTTAATAAATATTATTTAGAGTAGAGCTATTGGCACCGCTCTTAcaTCTTATTAAAACACCGTTATTACGCGTATCCCATTAAATAATATTAAAaaatttatcataactaTTAACACCCTCTTTAATTCCAAAAATAACATTATTtcagttaTTCTAAATAACTCTCTCTCCTAAAATCCAAAATACAGGTATCTTGTTTCTCGTTCTcttttcatcagtcttctcatctttaatTtcAatcaaatgaacattttaatttagagtttaaaaccaatccttaaggaaataattgacttataagaaaactattaaaaaagagagattgttcttgatgtttatgcactgaCAATTGGGTAATCATGATTCATGAGGTGTTGCAAATAAAAGATTTTTACTTCTGTGGCAGTTATTGTCGTGTAGACTAGGATAACCTACAAGTTTACAATTTTCAACTTTAATTTATGTGTTCCTTTCCtTcttgattcctttctttaataaaattaaattcatcaggattacaacaaataattatgaataatagaacaaatacatctcaaaattcttaaagccaaactcttatttttggatatctatgaaccaaatttgcaagagtgaattgtagatatccttacaaaaatttgtgtgtatttgacttcaaaaagttttttgttggttgctaattcttaagctatttgtattcatccagtcttgagattAGCAAGAGATTATATTAaTACAATAGCAGaACATGATGGAtgatgaatacaaAAGTTTtggCCgAaaaagaaaaggtaaatttcgcaggagtggtgtaaagaattggtggaaagagaaataataataggtttaattttttttaaaaatgtcctttaatgtaattttatctcaaaggtgttttattagaacaataaaGGGgTGCtaAtagctcaactcTATTATTTATTATAATTTTTAAAAttATATTCACctAAaT

>Cs1g19860

ACAGCAGCTGTAGCTTAAAAGCTAcaacacttaatTCCAAACAGGATCATAGAGTCTCTTCATATCACGTTGAGCTAGAATTTATCAAAACATTTGTCTACTTATAATTTTAATAATCTCKTTTTAGCATCAAAGAAAGTCCAGATTAATTCATGTTTGCTTTTCTTTGTTTTCAATGTTTGTATAGTATTTTACCCTAGCTTCATTTTTCATCAGCTAATCACTATAATTCCTCATTTGATAAGAAAATACCATGCGAATGACTACAAGAAATAAAATTAAAGCCCTCATTATTCTAAGATAGATGTTAAATCCCCTTCTATTAAGGAAGTCCATATTTTATTAAAATTAGAACGAAGTTAAATCATAAAAGCACGAGTTCTAAGTTGCTAAAGGACGGTAAACATTGTATTGAAATGTGCCTTTGCTATCTTTTAGTTTGATCAATAAAATGATACATCTCTTATTGGAAGATtattattattattattatTATTATTATTATTATTATTGCTGCTAAAtatagtgagaggttgcATACCTTAATCTAATACGTATATTATTGTGTTAAAAAGAGTGGCTCTTGAACTGATCCTCCCTAAAAACGTGTGATGATAAATARCATTTTATGaccaagttaatatTGTACCAAAATTTATTTTTTAAAAAATCAAATTTCTTTCTATGCTTACTTGAACCTCAGATaggctttaaaaaaaaAAATCTTACATTATTTTATTCAAAAAAATTTTTCCTAAGGGGGTTTGTGAAAGATGTATTTAGAACATGCAACCTTTGACTAGAAGATAGAAATAATGTCACTGAAAATTAAGACaaatcataaccaaaCTCCCGAGTCAAAAAGGAACAAAGTTATAAAATAGATATACATATAATTCAAATTTGACTTTTGAAGTGACATGTGAAATCTCCAAAATTTCATAAAACTTTTAAACTATAAATTGAACAAAGTTTCTTTACTTAAGCGTCAGAGGCATCAA

>Cs1g12910

atatgaggattcataataagaacatatattcaagaattctccactattaaacaagtctaaagggctaggatttaataaacctggaaaatgttttggaaacattccaacaccaaacacaactaccaaaaatcaacataaaaaaacatatcacttaaaattaaaatgatatgtttacctatacaacataaccatataagtatgtttcattatattttatctaaaatctactgaatcaaatacaagcaacacttttatcctattaagtaaacataatagaaacctggaatcaattattaagtaacataacggatgaatcaagtcaaaggctggcttctggtgtaaccaaagctgttgatctgcaaaatgtaagagatcaagaacttgatagactctgtcttggaggcttctgttccaaagacttggcttcaggctacgacttcggaaaaaaggtggccatgctgacagaacaagtgatcctattgaagaatgaatgaggagaaatcaaagaagttgctgagatgacaccacaagatgcagcggttgaattagttgttgagcaaatannnnnnnnnnnnnnnnnnnnnnnnnnnnnnnnnnnnnnnnnnnnnnnnnnnnnnnnnnnnnnnnnnnnnnnnnnnnnnnnnnnnnnnnnnnnnnnnnnnnnnnccaacgcgtctgctctcaccatcaatgctgtcagtgaataagcacatcacaccttaataatgacagctgggtgttatcatttcggtcaaaaaggtagacccccaaaaacaatgataataattaacagtcataaataaaaaataatattgggtatgttaccattttggtcaaaaagatgatttcgccaaaaagataattttggatctgtaatacgattttcgccaaaaagataattttgccgcnnnnnnnnnnnnnnnnnnnnnnnnnnnnnnnnnnnnnnnnnnnnnnnncctataacttgaggagataataagagtttttcattatactctact

>Cs1g11100

ACTCGTCTCARTTMACTTTCAATGCCCATATCTCTTTCTCTATTAAATTCCTTCCATGTCAATTTTGCTGCATGGTTTTGGTCCAGTAACGTGTTTGCAACTTGTTCTATTTTTCCAGGATAAGTAGCCATGGCAAAGCTGGTTCAAGAATAAATCTCTCAATTGATTTGAGCACCGAAGTAGATGAAAATAAATTAAATAATTAAATAAYGTATTGTGTCAAAGTGACTTACTATTGTTGATTTGTATGTAAATATATTGCTCTACCAATTTCTTCTCATGTGTAATTAATTCATTTGATAAAAACCTCGTGAATTTCCAAATGTTACCTTCAAACAATTAGTGATTTGTCTTCATATTGGGTTTGCTTTTGCCTTAATAGTTGCCACTAAATTTGTCAACCTGTCCTTATAATTGTTGCTGAATATTCACACACCGCATTACGTGTTTTGTGAGTGATTTTTATTTCTTTCATAAATATCTATTTATGTAATTATTCTTTTTTGAAAATGACAATTTTATTTAATATCATTTATTCTAGATAAGTANGGTTATTGTTGTCTATTTGATGACATAAAaaTTAtatctgACATAAAAATTATATCACAAAAATAATTTTATATTAACATGCTAATTATAACTGAGCAAATAGGAGAAACATTAGGAACTTTTTAAAATAGTACTGTTAAAAAAGTTTATGATCTGGGAACGACTTTTTAAGAAATATAAGTTAATCCAAGAGGTTATATTTTTTAATACAATTATAAATYTTTTATTTACGTGcatataaaattACAACAATGTCCTTGTCCAATGATCTAAACAAAAAAaagacATAATttTAAAaaTTGTGGATAAatTCAACAGTcaatatcacttttgaACTCTTATGTAAAGGAAGaAAATGATTATTCTTGAATTTTTTAAATAAATTTTTATTTTTAAAAGAAAATAAAAAATCTTTTAAAAAATTCGCATCTCCAAATTGTCTTGTCATCCTC

>Cs1g04470

TGTGTACTATTTCTCAAATTAAATTACACATATATTCTCTCTATTATGTCCTTTGTTCGTTTAATTtgttcataatctttttatGCTTTTATTTAGATTTCTTCACCGTTAAAGTTAATGAATTCATGAGTAAATGTTAAATTTATTCCGCTTTTTTTTGCATTTTTTTAATATGCAAACTAAATCAGATTGCTTTTCTATTATATGGTAATTGTCAATATATTTATAGTTTATTGTCGTAAATTTCATTGAATCTTCTCTTCTTTTATTTATTTATTTGTATATTTAAGGATAGATAGAGAAACTAGTGTCAAAACTCTTCCAGAAATGTACACAAGAGGATTATAGGTTCGTTTGTAAAGAGATATATTCAATATAATTATAAGATTACATACAGATATTATCTTACATAGAATTGGATCTGATTCTTAAATTGATTTTCCGCCGTTGGATTTGCATCGTCTGTTGCTCTCCTTTCAGCTTGCAAACCTAATATAAAAGGTTCTAATTGATTTGAATAATGCTAATGGGTACCTGAACATGCCTTTTtcaaagaacttTATTTGTTATATGGGTCACTCTATGGTGTTATTATAAAAGTTATAGGTTTATTGCAGGAGTTTAAACGAGTTATTTTAACTAATTAAATAAAAAAAGAATTGTAATAAGAACATATCTTATTTAAAAGAATTAGTAATCTTTTATGATATCTTATTTATTTAAACAAAAGTTGTTACCGTGATTGGATAGGATTATATAGATCTCGGAAAATTCTTAAAAATTTCGTTTCTTTATGTAAGTTAGAAAAATAGTTTCCGTACTGTATAAAGAGATACCTAATTAAAGTTGCGTGTATGAACTAATATAAAAAGAGTCGTCTCGTTGAAGGCTTGAAGCCTCTCTCACATAGGTGGGATTCAGCCATCAAGTACGATTAATTTTCCTCCCTCTATTGTTTTCGGGGTTGGACAGTGCTACGCACATACAGATACAGGAAAATA

>Cs1g02000

TAATTGCTCATGTAAAGTAACAATTAAATGCAATCACCATTACACATGCAAAGTTACTATGTAYAGCTATGTAAATTTATTTGGTGTAAATTTAACCATTTGCTACACATCCATGGTTCATATGACTAAACCGGCTTCCTTTTCCCTCTTATGTGCAGTTCTTTGACAGGTACCGAAGCAGACGGGTGCCAGTGTAATTCTRCTATTAAAGGAAGTACARTGTAGCGATGAGTCCACTTGTAAAAATACTGCAGGATGAAAAGTAAAAGAWTKAAAAAAAAAATCTTTAATTTTTGTTAGAGTGTTGATTGTAGAAATTGAATCTCACTTGACAGCAAACTAAACCTTCAACCAAATAGAGAAAAYGGAAATGATGGAAATGATTTCAGAYTTATTTTGTATTACTCTATGGTGGTTTTTCTTTTGACAGCCATTGCTGAATGGAAATAATTTTGTGTTTCAGTTACGRTTTTTTKATATCATTATCAGTAATCCATTRAGACAAAATAGATAATTTCATTTTCTTTTSCTTTTGTTTTTGACAATTATTATCAATAWGATTTATAAAATTCTAATCTAAATTGTTGTTTGTTTGTTTGTTTGTTTAGCAYGTAGTAAMRGGWATGTCTACAAGAAGATGGATATCTAGGTGGGAAAWYWWWWTTTWATCAAAAATATAAAAAATTTAGAACTATGAATCACWATTTTATTTATCACATTTTCTGGTTTTAAAAAACCTTTATTAtataaatatatatatatatatttttcaTTTTCTTATTTTTACCATCATTCCYTCACGTTCTCGCatcacacgatgctctarTAMCCAKTGTTMTCAATTCTTTAATTCCGTTAATACGACATCGTTTTCAYCTTTCAGCTTGYGTTTCCATTTCGTTATTARGCGGTGCCAACTGTTTACAGTGCGCCGCCGTTTAGACAAATGCCTCATGTCTCTCATTGTTTGCGCCAAATGCCCAAATCTTAACAGTCTTTCA

>Cs1g04080

AGGAAATTGCTATCTTCGATAGCTCATATAGGGATTTGTGATCGCATTGAAATGAAATAATATTTTGTTTCTGAAGTCTTTACTGAATAACCAGTCGTTGAAATTTGTGGCAGGGAATAATCTACAGTTTGTTGGATAAAAAGAAGGAGGCTACAGAACAGTTTGAGATTTATTGGAGCCTCGTACCTGATGAATTTCCACAACGAGGATTTCTCGATGACGTGGTTTTGGCAGCAAAGACCAAATCTAGAGAACRGTCTCAGAAGAATTTTGATGCTGCATTCTCACACCGGAAATAAAGCACCAGCTCCTTTCTTTTCTTKCTCTTATTATTGATTTAGCTGCTTGCTTGTCATATTTTTAGGTTAGTAACTTTAGAAACAGAGARAAAAGTTGCTAAATTATGTGATAGTAATGTACAGTGtaatttttgggcAATATRYAAGCTCTTTTGATACGATTGGTCTCAAAGAAATATCACAATTCACAACATTTCAGGCGCTGCTAGTTTTAGCCTTTCCCCCACTCTTTCCCCGGCGAACCCCAAATAATCAATTAAAATTCAAACAAAAATGAGCATTGGACATTAAATGGACATCAAAATCTCATGGTAgagtaatttaatttgatgttatagtnnnnnnnnnnnnnnnnnnnnnnnnnnnnnnnnnnnnnnnnnnnnnnnnnnnttggtaagtgataatattatgtagCACTATTTGATTATTTTATAGTTTTAATATTATTCACAGGAAGATATAGAACTACACCACGTGTTTAAATGTTCATTGTATGATAAGTAGATATAGAACTACACCACGTGTTTAAATGTTCATCGTATGATAGGTATTGGTGCTCGCTTGATTATCATCATATGACAACTATATTTTAGAGAATTTTATAaaaaaaaaaaattgagaaCCACGTATGTAATTAGCTATATCTAAAAGGACTAAAGAGGCAATTACGTTTTTATCTTAAAATAGAAAAAATAAAGTAAC

>Cs1g02450

aaactttattgtcgaatgtatgaatgtatggtaaaaaattgataaattttttttacaaTCCaaaacttctaaaaactttcccCGTGATTTTTGTATTTTCATTTTTACTATCAATAATACGCCTCTACAACGAGGCTCGTTCATACAAAAATAAAAATAAAAATCATAAATTATTAGCAAAAAAAACATCAATTATTCTATCCTTAAGATGTTAAGCTAAATGATTTTCCAGCTGATGTATCACCAATCAATGACCGACATGTATTGAAAATAAATYTTATTYCAATAATGCTATGCTTACTCAGTTGGAATATAAAAACTAWTACGATAATGTTATACTTACTCAGTTGGAATACTTAATTTGTAtcctaatttttgCAGCAAATAATGTAATACTATTTAATAATTGGCTCAAGTGATAGTTAGTTATTAAATCTTATRATTTTTCATTCAAATGATAATARTCTATTYTTAKCACCTRAATTTGGGCACTTAAAGTAAGCATAGCATTTCTCYAACTATTATTCTATCACTYATGTGATGATTATCTTCCATGCTGAAGCTGCTGGAGGGTAGCCACGCCAAATTTCAGTAAGATCCCTACTTTTTTGGTTTATGCATTTATAGATAGTAtaacttggtaagcATTTTTGTTTGGTCGGTATAATCTCTTGCATTGTAATAAAAGGTTCRAGATTCAAATCAGTAATGTATTTCACTTTYCATAAACGTAKTATACATWAaaaaaaaaaaaaGAAACTCAACTTGATTAAWATAWCAAGRCTAGTGAATCCAGAAGGGTTCTCCTAATCCTTTGAAGTCAAACATACATAAAAatatcattttttcttttTTCAACTTTATTAAGCACCGCCAGCTTTAGAAGAAATTTGAAAACGCGGGAACCAGTATACGACAAAATGGAAGTTGTTGAGCCTCCCCGAAAATTATTTAGGAAAKATGCAAACAATTTCGGAACCTAGTTGCAATTCCGAATAATT

>Cs1g10390

tcccatttgggatctgcaaccaccagcgttagtatctcacgtgaaagatctagaaagacaactcaaggaaatagaggatggtatatatcatctgcaattggaactggattcaataaaaggacgaaggtgagttnnnnnnnnnnnnnnnnnnnnnnnntattgttctttttcacttttgatttgattttgcagatttgatttattttgttttgattcactctcccatataaatggcggataacggtactccgtgacttttttaagtcggtattctcagtttcccacaataactgaaacctcaatgtcaagtatggaagaaatacaacaaaatttgcatcaacattttccttctctccctcaaaagcnnnnnnnnnnnnnnnnnnnttgttaatgatcaatgggattcctgttgacattcgctggttaattgaagcaaaggtctgattggcaggtgagttttctgatccattcatttcctacatgcctggttttggtaaaagtacatttgctaagaaaannnnnnnnnnnnnnnnnnnnnnnnnnnnnaacgtgaaattcaaaatttatggaagttgttccaaaaggaaagtgcacggtttagtcttgggaatctgactctaaaagaccttgtttgccagtttataagaaaattggatgagaagcctatcctcgacccatagagggcgttcaagccgttactggacgtaaaatcagaggaagatgtgagccacaatcacaactaccagtaaatatctttttattttactgttcttttatcttttgcattattattattATTGTGTGtcTTTTTTTatctaatcactgtttactttttcttttcactgtttgcctttgaattattaagctaaaagcttcacgcgtcatttgacaaacacaccaccccatttacagatgtatatcattatgtccgccaccaagatccttaaataggaacttcttttcaagcactcacaaattgtttctttttagaattgttctaa

>Cs1g03090

ACGAGAATATGTGATGAATAATGTGCTCCGGAAGATTGCTGAGTCTATTAATAGTAATTCCATtttatccctcctccTCCTCCTCCTCCTCTATTAATAGTAACAATTTATTATTattaggaataataataataatgattaTAAAAATGATAATTATTATTTCAAAATGagaattaaatgattataaataatcataataataatGATTATTAAAGATAAAATGAGAATTTCAAAATTTAGGCGGAGGTAATATGGTCACTTTAAAAAAANAGATTAATAATTATAAAAATGATAATTATTATTTATAATCATTTAAGGTTAATTTAGTAACTTGTAATAATTCATTGCGATTTCTAGATATAGTAAATACATCAATGACAATACAATTCATTCTAATTCTGATTCTTACAGTTGAAATAAARAATTTATTTTGATTRTCATTCTCTATTCCGATTKAAAATAAACAGTTTATTTTAATTCTTATTTCCTATTCCGATTCTAGCTCATTCTCATTCCCTATTTCGATTCAAAATAAATAATTTATTTTGATTCTCATTTCTRATTCTYATTCTACTCCATTATGATTTCAGCTCATTTCGACTACTYATTAATAAACGAACCATTAATGTTAAAGATACATTTCGGTGGGTCTCTATGAGTCTATTGTCTagccccttttttttccttcAATTAAAGTAAGGGATTGGGAAATTCCGGCGACAGAAATRAGTATGTTGGTTGACCAAATTAATTAATTTCCATTCAAAacaagaatttaatttatttattttcgggtATTTAAGTAATTTTAATTAAAAAGAATTTTACGTGGYATTTAAAGAGTAGTCAAAAGGACCCAGACSTATTTYCATTCACCACTGGCTTTGAACAATGGAACTTACCGGCCCAGTTATTTCTTCTTCTAATTTCTAAAAAGTGAAATATCCAATCAAATGAAATAAAATAAACGTGCTAGCTTCTCCGATCGCACAAACWTCGCG

>Cs1g08030

AATATATTGACTTAGGACATGTCATTGATAACATGTATAATGAAATCAACAACTTTTTATTTAAAACTTAAAATTGAGTTGTAATTACCTTTTACCCAAATTATTCACTCTAAATTGTTAATATACCCTTCCTAATGATATGATGATAAAAAATGATGAATAATTGGTAAAATTAAAATAAAATTTATCATTGGAAAGGGTGTGTTTGTAACTTATAGTGAATAATTtgcgccaaaaaTAATTACATCTTAATTTAAAGTTTTaaaTAAAAAGTTGTTAATTTCATTATACCCATGACATGTCTTAAGTTAATATATTGAATTATTTATTATAGGAAAATAATAGTAATTAAAAATTAAAAATAAATTTACTATTAAAATAAATAAAATTTAaaaataaaaataagagTTATATGTCTTTTTTAAAATGTTAAGAAATTTTATGTCATTTTTTTCAAACGTGAAGGCGTCATTGTCATTTTCCCTTATTTATCTATATATGAGAAATTAATAACGTGGGGGCCAACTTCACGTGCATAGACCACACTCATGCATGACTATCATTCAACTATCTCAGCGTCATTACTATTGTAACAGTGTTATCCTRCAATTTTATTCGAACTCAGCTGATTAGTTAAATAAAAATCATTTTTAGAGMAGTATTGTCTTACACTTTTATTCGAATTCAGCTAATCAGTTAAATAAAAGTTATTTGAAAACCCTTGACGATGCCTTTGGAAACTTATCAACCATTTGATGAACATCGCACCACATACCACATTATTTGTAATAAAATTTTGAGATAAAATTTAGAATATTTATTGTTGTTACACTTTCTATTTAGCCATCTCAATCTCTCTCTCTGTCATGGAAAACGACAAAGTAAGATTGACTATTTCTCTCAAAAATTTTCTCCCATTGGTAGGGCACTAGGGCCCTTCGTCTTAACTCTACATGACTTAATCCCTTGAAAATTTTTCAGAAAAGGAAAGTCGAAAAGAG

>Cs1g17020

nnnnnnnnnnnnnnnnnnnnnnnnnnnnnnnnnnnnnnnnnnnnnnnnnnnnnnnnnnnnnnnnnnnnnnnnnnnnnnnnnnnnnnnnnnnnnnnnnnnnnnnnnnnnnnnnnnnnnnnnnnnnnnnnnnnnnnnnnnnnnnnnnnnnnnnnnnnnnnnnnnnnnnnnnnnnnnnnnnnnnnnnnnnnnnnnnnnnnnnnnnnnnnnnnnnnnnnnnnnnnnnnnnnnnnnnnnnnnnnnnnnnnnnnnnnnnnnnnnnnnnnnnnnnnnnnnnnnnnnnnnnnnnnnnnnnnnnnnnnnnnnnnnnnnnnnnnnnnnnnnnnnnnnnnnnnnnnnnnnnnnnnnnnnnnnnnnnnnnnnnnnnnnnnnnnnnnnnnnnnnnnnnnnnnnnnnnnnnnnnnnnnnnnnnnnnnnnnnnnnnnnnnnnnnnnnnnnnnnnnnnnnnnnnnnnnnnnnnnnnnnnnnnnnnnnnnnnnnnnnnnnnnnnnnnnnnnnnnnnnnnnnnnnnnnnnnnnnnnnnnnnnnnnnnnnnnnnnnnnnnnnnnnnnnnnnnnnnnnnnnnnnnnnnnnnnnnnnnnnnnnnnnnnnnnnnnnnnnnnnnnnnnnnnnnnnnnnnnnnnnnnnnnnnnnnnnnnnnnnnnnnnnnnnnnnnnnnnnnnnnnnnnnnnnnnnnnnnnnnnnnnnnnnnnnnnnnnnnnnnnnnnnnnnnnnnnnnnnnnnnnnnnnnnnnnnnnnnnnnnnnnnnnnnnnnnnnnnnnnnnnnnnnnnnnnnnnnnnnnnnnnnnnnnnnnnnnnnnnnnnnnnnnnnnnnnnnnnnnnnnnnnnnnnnnnnnnnnnnnnnnnnnnnnnnnnnnnnnnnnnnnnnnnnnnnnnnnnnnnnnnnnnnnnnnnnnnnnnnnnnnnnnnnnnnnnnnnnnnnnnnnnnnnnnnnnnnnnnnnnnnnnnnnnnnnnnnnnnnnnnnnnnnnnnnnnnnnnnnnnnnnnnnnnnnnnnnnnnnnnn

>Cs1g10040

nnnnnnnnnnnnnnnnnnnnnnnnnnnnnnnnnnnnnnnnnnnnnnnnnnnnnnnnnnnnnnnnnnnnnnnnnnnnnnnnnnnnnnnnnnnnnnnnnnnnnnnnnnnnnnnnnnnnnnnnnnnnnnnnnaactaaatattcggatttgcatatttttttgaaataaattctctgcactatttattttttgtcatatgtatatttaaccatcacaaaattttaacaagtgatttgcatcactttatttctaaacttgtatcgattaactactttgacaccaacaaaatttagaattcattaaataattcaaataaaatgatcattttacccttaaataaattcatagactattttatcttataaaattttaaaaaataaAAttataaTTACAAGAATACCCCTAAATTTATTAAAACAAATAGATCTCAAAACCCAAAGTGTTAATTTTTGTAATTGATAAAATAAGCATATATTAACGCACGTATGACATATGTAATACAACTAATTAATAcataaatgacatgtgtagtttagtnnnnnnnnnnnnnnnnnnnnnnnnnnnnnnnnnnnnnnnnnnnnnnnnnnnnnnnnnnnnnnnnnnnnnnnnnnnnnnnnnnnnnnnnncttatgtATTTTAAAATTTCTCTTATTAAAAagttgggaaacccCACTATTCCTTTTAGCCTCCCCCGTCCTCACCAGAGCTAGTAGCAACAAAAACAAGCATACTCAATTGCAGTAGAATTTAGAGCAATAGAACTCAAGGAAATATCTAAAATGTATCATATRGTAAGACTAAATACCTCGAGAATTTGGTGAAGACGGGATTTGTCATCTGGGCCCATTCCYTGAGTGCAACTRATGTAGAAATAAGGCTCGAGCCCATGAACCTTTCAACCAACACTAGGCCGTAACACATACCAAAAGCAAGAACTYGACCAAACATTATTACTATTATAATGACAGCGAAGTGACTCACTGATGGTCCGAGATTGGGACAa

>Cs1g06170

ccgcagacatatggattggatatggattgagtcaaattcgcatccgatctgcagatgtatgaattggatttggattgagtttaatcaatccgtggattggattggattgaaaaattataatctgtaaaattatggatcggatatggattgatgtctaatccgtaaaatccgatccgcgaacactcctacattttaggatgagaagaagacagcaaacttggcagggccaatattgatcctttcgtgcaagttctttttgaacttaggttataatctgtggttggttaaatgttagatttaacttcgcatatatgaactatatagacatctttgaacagttttttattagcctcctatagagcatttgatagatgatattagcctaaaatgatacttcaaggtaagacttatttacatgctttggtaagttgtgtttatatgaaaaatatccgaaaacatacatcttttggaaattttaaggaaattgcattaagatttttaagaaaatattttctttattattattcaaaaaatatattatttttggtcccaaatggttttcttttataagaccaagatcaaagataatttcacatttacatgaaaatactttcggcattagcattagaattcagaaacgtttttatttagataaaaatatattaaaacgcttgaagtgatattataaaattattttatatctacttttttttttccaacaagggtctttaaaacaaatactttaccattataggtcacagataaccacattgaaataaataacctgagggaaattactactaagaccccagaaatttttcccgcttaatcttaacgaacAAAGGAATATAACTGTCTAGAGAGAAAAATAAAGAAAGCTGCACAAAGagagagaatcaaaGCAAGTCATAAAACCGAGCCTTAACTGAATCTTTCATTCAATTCCTGTAATTTCCTTtcttttttttcTTTTCTCTTAAGTTTCTTTTTTTCTCTTGAAA

>Cs1g12400

TTTAACTGRGCTTGCATTTGATTTATATAGAGGTGACGGTGGAGTTAATGAAATGGGTCAGTGTATTAATGAGATGCAGCGACAAAAAGAGAGGCTGTTGCATGAAATGAAGTGAAACTAGTTATGGCTTCACGAgaattggggggctaggaATTAATGCATTAGTGACGCCATCAGCATTAATGCGTTTCACGGGATTTTTGCCCACGTCGGGTTGGAGCTATCGGGKCGGTGTTGCTCSGGTGGGTTATAGTCGTGGATCAATCTTGTTTCTTATTGGGCMTTGTAAYACCATTGTATTGGGCYAATGTCTCNAATGGGTACRGNTANAAATTTTGGTTCGACTATTGAGACCCTTCGTTAATCTCGTTAATCTCTTATTAAATTATAATTTTTTCCTCTCTTGTCGATCTATTTTACTTGTACATGTATATGAGCTTTTCTTTTCTCTCTCCTATCTATGCACAAACCTGAAACattaaatttttttttttgttggtACAGCAATGATCAATTATGTACTTAAATTTAAAGGACATRTTAAAAGCCACCAAATATATCTTCCACAAAGCTCATGAGCTAACAATTAAACAATTAAACAATTTAATAAATTATAAATTTGTTTTCTCTAAACTTCATAAATATTTGAAGCTCCTTCTATGAAGATGCTCAAGGAGTAACTattagagaaagtatacatatacacagtaagttaggtttaaatcaagtgagactagtatggagtttcatctctttcttTTACTTTCttttttttttCATAttaattatttttattttagggaagtTActattttttttttGTTTTGCAGAaagagaaagtctaaAGAGAAGAATAGAGATATCAAAATTGTAAACAAGATTTAATAATAAAAAAATTTCTGGAAGTCGAAAGATTTAAACATAAAAAGATTGGTTATTTTTTAAAATTTGTATTTTTAGTATTAAGATGAGTACAATAATTTTTATGAGAATTGTGGGAG

>Cs1g18920

GACTAGTGAATTGTCTTATAGCTTCTTTTATAGTATATTTCtttataaaaaaaattaattTTTGGTCTAGAATTCAATATGAATATCAAAATATTTGAAAAAAATTTATGTCTACGTGTTCTTCATAATTTTACCGGAGTTAAAAGTTTTGTAtagtatttttttttaaaaaaaagaatcaATAATGATATAACCACGAATAAATGGACATGCCGAGTTGTAGCTTATGAGTCATGAAGAAATTAGTTAGACGAAAGCATCATAAGGCCCAAACATAAGATCGAAATTCTCTCCTGATATCTTTTMTTTRAACAAATACACTTACATAATTGGTAGTTAATGAATGAGAATATAATAATTAAGTAAATATTTATTATACATAAATTaaagagtatatgtatgaaaagatttttcagATCTTTTATCCTCAAACGTAATAGTATAACGCGACACCTTAATGATATAAGCAGTTGAGATTACAAAaaaaatgtaataGTTGGAAGTAGGGGCGGAGCAAGCAAGTTTATTAGAGAGGGGCAACTCCAAATAACAGAATATTAAAAAAAAAAatatattttttcatggaATGTCGTCCATGTGGAGCATACTTCGCATAATTCACATACAAACATAAGTAGTTAAATACAATTCTTGYAGATACATTAAGCTTATCGTTAAAAGAATTTTTGAAATGATGAAACTTATTTGATAAATTTTAAGTAGTTCTTTTCTCTGAAGGCGGTACAGCTAAATATTTAAAAATGAAAAATGAGTGTACTATGGAGGCCGCTTAACTAACTTTTGATTGGAGGTATCTACAAAGAAAAATAGGAATTCTTGGAGGCCTCCGCCCTTAGCCCGATGATGCGAATAACTCCTCAATGCATGAGACAATATGGTGTCAAATTAAYCAAAACTCATCCCYGTGTTTGAATTTCTAAATATTAGCTTGAGTAAAGGAAAGAAAAACCCTGCTGGGTTTTTGTTGGCTTCAGCTGTGA

>Cs1g06310

nnnnnnnnnnnnnnnnnnnnnnnnnnnnnnnnnnnnnnnnnnnnnnnnnnnnnnnnnnnnnnnnnnnnnnnnnnnnnnnnnnnnnnnnnnnnnnnnnnnnnnnnnnnnnnnnnnnnnnnnngtcattaattgagtggttgataattctttacaatatccaagtgaattaattgtattatctcaccaactactcaattaatgacacatcatttgggatattttttaggataaaaaatttgagatgtgtagcattactannnnnnnnnnnnnnnnnnnnnnttaattgtattattctcaccaatcaattgatgaataccacggtgtttgagatggcgtttgaagataaaaaatttaggatatttaaaagtgaagttcaatctaaaaacctataaaaaataaaaagaataagaaaaatatatcttataaataaaaaagactcgacctctcaacTTAAGAAATCGACGAAATTAACGCGCTTGTTACTTAAAAGTAATTTCCTGTGCTTTAGAACTAATTAACAGGTCAGCAATTCAACATACTTTTCATATTCCTAATAAACAACAATGAGATTTCTRGCACTTAGGCGTTTCAACCTCAACCTCTGTCATTTTCCCTCACATTTGCATGGACCATTACAAACTGGACAAAAATTTACTCGTCTGATTGCATTTGGCCCGCCAACACATGGCAAGATCTCATTGGAGTACCACAAAGGGCGCTCCACTTGTACATCCCTACAGATAAAATTGGACCATATATACATAAAACTCTTTGTAATTTTCCTCGTGCATGTAGCTCTATAATAATTCATGTACTAATGCAGCCATTGATCAATGCAATTTGATCAATCAAAAGTGTCCATAATTGTAATCAGAATCAACTTGCCCATAGGCATGACGCCACAGCATCTGTTGTTGCTGGCCACATACACAGAATCCAATACGTGTTTAATTGATCCACTAYCACCACCAAGCTTTTATTCTCCAAGCAAACTCAATATA

>Cs1g22540

GTTTAAAGGCTGAACTAGTCAATTTAGTTCAACTAYTTTTAGATGAACCTTTCCACGAAAAAWGAAATTCGTAACAACCTCAAAATTTGTGCTAGAGAGAGAAAGAGCAACAATCATCGAAWGTTGACAAAAGAACACAAATCAAATTATTTCTCCTCATTATAAGATTAAGAGGAAATGTAATTTTTTTTTTAATTCTTATTTCATGCTCATTTGTAATTTTATTATYCYTTGTATATTTAAATGTATAATTATTTGTTAATACTTACACACAAGAGGAATATTATTAAAAGAGTAAAAGAAATCTTAGACACCTTAGAAGACCGAGTAATAGAAGTTTGAAACAACRACAACTGCAACGACCTTATCCATATAAATACGAAATGTCAGCCwgtgccaaaagagaaATTGACCAAAACACGTCAAATTTTGCAATTTGACCACAAATTGCATTAGCCGTAAAGACAAATATAATTATCTTTTACCCCCACATCGGAGAAAAAATTggcttcatttaataataataatctcattttaCCCCacatcggagaaaaattggcttCCTCAATTTTAATGACCTTCATTAAAAAaaaaaaaaaagaGAGAAAATATCGTAATACAATCTTAGCATCATAATAATAATAATAATCTCATTTTACTCATATCCGAATATTCTCAAAATAATAAACAAAAGGCATGGTTTATTTAAaaaaagaaaaaaaaaatatctGCAGGGATCCACTCCACCTAACAATATCAAAGGAAGGAAGGAAGGAATGAACCACGTTTCATATAACTGGGATCGCCRRAGTTTCCGTCCAAAGTCTGAAAACGAGTCCTAAGCAGGAAAATATCCTCGCCTTTCACGTGCATTAACTGTAGAGAGCTAGCAAATATTGAATCAAATACCaaaattaaaaactactaATAAGTAGTAARTTTATTAAAAAAATATTAAAtattnnnnnnnnnnnnaaaaaaaaaaaaaaagAAGGAATCTC

>Cs1g02830

CAAAAAcgaagaaaaaaattcgatttcatcccgtatttagtacggcggcgaagaatcaaattataactatatttatacctttttctacttcgtcttccaagtgcaggataaccccaatgggttgtgggtcttttttctaccaaacagagtccttccttcaccacacccatggggatggtctacagggttcataactatTCttactactagacgcttccctagccaacatttagatctgggtttacccaaACTTTTCTGGTTTAATCTGCTTTCTATGCATTTGGAGGTMTGTGAATCAAATTGTAACCTGTAGTGCCTGCTTTGAATTTTTATTTTTGTTATTATTATTATTTTATAGATTTTTTGCATGAATGGATACCAGACCTTTCTGCATGGACCCCACCCATGTTAAACCCTAGACTCTTGTACCACCCAGGAGATTTTAACTAACTGAACCAACTAGCACCCACAGGTGGTTGCCTTGTGCTGACTATATATTTATGCATGAAATAGACTTCCTAATGCTGAGRTTTTAattttcttcagacCTCCCTCTCATGTTTTTTCTTGTTGTCTCTTATATGGTTTGTAGAAGATGTAATTTGTTATCTAGACAGGAGTATGAACAATTTATCACTTATCAATGTTATTTTTTGTGTTTTTAATTAACAATGGCTAATTTCATTCTTGCCATAACTTATGGTTTTCATATAAAGATTCACTTCTGTATTCGCTGTTGTATTGCCATTTATCCCTTTTTAGGAAATACAACTTCTGGTTACTTTTTAAATCAAGATGATGGATGAATTGTGTATTTCTTTGTTTTTTAACCTGAAATGGTAAGAGTAAACATTGTACCAGTCGAGTTTATGAACTTACAGTGTTTAAAGTACTTTTACGATCCTTTTAGTAGGATACATAGACTTTTCTTTAACGGTATTTTGTTGTGTTTTTGTTATAGACTTTCTTTAATGGTACTTTTTTTGTGTTTTTATTatatc

>Cs1g16890

ATGATTTTTTCCTCTCTTCTCAATCTATATAATTGGTTTTCGCACAAACTTAAAATATTAATCTTTTttnnnnnnnntttcatattgattatttTTATTTTTGGGAAGTTTGTATTTTTTTAAGTTTTATTAAGAGAGAAAGTATAAAGAGATTAACAAATATATCAAGATTGTAAAAAATTAAGTGTGAGAAAGTTACAAAAAAATAAAAGCTTTACGAATGGAAAAAGTGTTTACTAGGAAAaaaaaattttccacaTTACTGAAGTTTAATTGGAAAAAAGGTTTTATAAGAATTTTGCAGAGGTCGCCATAGACGTTCAATCCTTAATTCTCTTTTAAAATGAATTTCTCTTGACTAAAATTTAAGTGAGAAATTGACAACTAAGAATATAAAAATTATAAATAAATTAGTCTTAAGATTATCACTATTTAAACACGTGGCATAATATTTTATTCgggatgtctnnnnnnnnnnnnnnnnnnnnnnnnnnnnnttttatttttgggcgctttattagcatggATTTGTTGATTTACGTGTGAACTAATTAACTCAAAGTCGAAAGAACCAGACGTCTCAGCCTCTAAAACTTGTCTGCACATGCCAACTTCGTTTCTCCGATATTTTGTGATTGCCCGTTAGAGTCATCAAGTCATTATCTATTTGGTTAACAAGAACCTCCGGAGGTTCTGTTGCTTTTATTTCAATTCTTGTTATCAATTTGTTCAAGACAGGTTTTGTTTAATTTCTTTCAATGGCAGCTTATTGTCTTCCTTGTACATGGCTTCCTGCTTTCGAGCCAGACCATGCTTTAGAGCCCCTTCTGTTGCTGTCTTCGAACAGACGTATGAAGCTCATATCGCCATCAAGTTGCAACAACAGCAACATTACTAGATTTGGTATTTGTAATGGCTTTTTAAGAGAAAGCAATATTAATATAATGAATGGCCGCCGTCTTTGGCCTTCTTTAATTATATTAATGCTTTCTCACTCTTTC

>Cs1g09810

AAGGCATGCCACTTTTCCCTTTTCAATTCCAGCACTACTAAACCTTAGCTCAACAACTTAACAAACTTCATCACTTAACAAAACGAAATCACTCTCAATAAATTAACAAATTCCATCAAACAAGCATTGGGTAATTGAAGATATAATTatcaatttggagagcttcttaaggttagtttatactcttaccataacctaaactTTTAATCTATTGATTTTATCATTTGAGTTCGAAATTGAAGTGGGTTTTTATTGCAAACGTTGTTATTTTTCATTAACAAAACAATGTGACTTGTTAAAGTAGTTAGTTTGAGTTGAGAAATGAAGATCAATCATTGAAACCCCTgaaaaatcgagcaaaacagaagcccacaaaaagtgagacaggcgcctgtctcaccaaaatatgtgcgacaggcaagtgggacattcgcctgtcacacatataaacccactggttttatgtgagaCAGGCACCTGTCTCACTTGTTGCCACTGGTTTTATGTGAGACAGGCGCCTGTCTCACTTGTTTCCACTGGTTTTATCTCTCACTTGTTCTAATTGATTTTATGTGAGACAGGCACCTGTTCCACTGCCTGTCTCACTAAATTTCAGctatTTTTctaaagtcataacttgggctacatgtgtccgttttaggcgtataaaatatcgtttcgaagcttacaatgagacgaatcaaatccaaaattgtatatttcatttcatccacataaatgtactgcatattcactttactaggttttgtagcgtgtaatcttatctcttttgttttttttataatttttaggtttaatggattccattgtacttcaattatgttacaacggttggtgggagacgttagcagatggtcgtacggagtacgtgaATGCGAAGAATGCAACATTTTTAGTTTGGAAAGATTGTACGTTTGAGCAATTTTTGGCAAGGGTGTATGAAGTTTTACAGATAAATCCTAATTAATATAGTTTGACGA

>Cs1g13036

AATATAAAAAGRTATAATTGTCCAATCTCAATCAMAACCTYAGCATTTTAGCAAAAATATACTTTTATTCCATATTTTACCAATATCCTCACCATTATTCAGAGCACCAACCTTTAACTTTCTTCGATTCTGTTTGGTATAACTTTTTAAYTAGAGCTTTTACTAAAAAATAATGGTTTGGTTATCAATGAGAATTTTTATTAAAATTTATAAAATTACTTTAATAGGTAAATTTCTTAAAGTTATGTTATGAAATGAATAAAATWAAATTGtgaaatatataaagcatattttagacaatannnnntttaaaaaaacttaacctgtactTCAAAAAACTTAAAATTTAAACTTTTACTAGTATAGATAAAATAACTTATTTaatttttaayaATTTTACTCAAATTATATAAAAAATATTAAGTTTAAAAAAAGGTTATAAGATTAAATAAATAAATTTGACCGAATYTACACTAACTCTTAYAAAATAAATATTTTGCGCCACAGAGYtaaggtcaatwaTGTTGCAACTGAGGTATTgtaccatatatatatatatatattggtATTTAACAAAGTTCTTGGGTGCTTCTCTGTTCAATCCAGCGGCTGAAATTAATTATTGGATAATTGATGCAGCGACTGACAACCGTTGCAGCCGGTTGTATATTAAtttaagagtaatgatacagccacaaactcttgtacaaacttattttgtacaaactgacgtggcattaattcattggttgaatgaaaatataaattaataaaaacaaatcatgtgggccaagtgatatttaattcaatcaatcttatcatgccacatcaatttatacaaaataaatttgtacaaaaatttgtggctatatcatcactcttaaTttaatagcagttcagcaagcgtaggttccgaaaatttcttagtcctgattagggtttcactaattccacctgaaaattgatctcgcgacagcaattaagacataaaattagaatta

>Cs1g13090

AATTTCAGATTGCTAATTGCTATGCTTTCACCCTTTTAACTGTGGACTTGTCTTTGGTGATTAATTATCAGTAAAATAGCGAAATAAAACAtgtctgattctcAGCTTCCTTAGTGCYTATAACATATGcgttttttttaaaaaaaaaaaAAAAACACGAAAGGAACCTTGTAATTAAAGTCTGGGTCTATTAGCTGGTAAAATATTTTGAGTTGATACAACAAGTGTTTGATATATGATTTTATTTTTTATTTAATGATAGATCGTCATTGTAGATAGCTTGTTGTGTAACGTTTTTGAAATGCAATCGTAGAATGTATTATGTTATTTTATTGCTTCACTACTCTTTATCCCGTGCAATGCAtCAAACGTGCTCTATGAATACTATatttagatcttgagtatcacatatttggtcaatggaacaagtgagcataaacaattttaggatgctaatattttacaataagatatataaggtttgtttaagtgtcactatcaatctaagttgacgtaaacttatttattttatcaccatcaacttgatctgaatcgaatcatgctgttacttcaacttggcaaataaaaattctatcttagttgtttcaaaatatctaacttgttttacccttgttatcttcttagagagaatctgaagggagtctaaattaacttgatgtgaatcattggaggtgttagttgatattattatccttcatttgaggccaaggtctaactcctatgataaggaacgactttcctagaactcgggttctgggttcgagcctcactaatgggcttgtttaatttaaataaaaaaattacccgccattttattgcactaataatatcataatattttattgtactaataatatcataatattctttcaacttattgcctcttcttaaattagctataattcatttcttccaaaacctcacaagctcaacttttcattgttaagttattccttcttctttgatatctcgcagaaatt

>Cs1g18380

nntttgagtaatgattttgtatttttagttgaattttgaacaataaatttGTATTTTTAGTTGAGTTTTTATCCTTAAAATAAAAAAATAAATTTAATTACACACTTAAATAAATTGATAtttgttattccaccccaatagtttaaatagCCATTAGTATACATCCATACTAATAAGATATTTCATTAACTTAATTAAGAGAAGAGTAAAATTTTTWTAAATAATTACTCTATTTTAAATATTTATGTAATTATCTTTTAATTAAAATAAATTTAGCCAAATTTATGTACTAGCTAAATTTARCCACATGCATGCACATGCTCAACAAGTACGGATTGATATATCTACRAAatattaaacagctgATTCTTGTCGGCCACTARCTAAATTTAGCCACATGCATGCACATGCTCAACAAGTACTGATtatattatctaCGAAATAGGAACAGCTCATTCTTGAAGTATTGTGACTTGCAATATTTGGTTCATGCATTCTACTTTTTGGGTATTTCTTAATTTTAAACAAATCATAATATATAATGAAGGTTTTAAACAAATCATAATATAGCCACATTCATCTTTCTGAAATCATGATCTCGTGAGATTTTGGAGGTTTTAGTATTTGATTTYGTAAGCACTTACGCAAGAAGTMGKGTTTGGTTAATTTAATTCTACACTTTAAATTAATAATAGTTCTTRCATTAATTACTGATTAACCAtatgattttttttttttttttgttgcaATGAGATAATCACTTTCTAGAAGAAGAGAAGAGACTTTGTCATTATAATTTTGTTCTATATGATTTGGTGATTACTTTTCCAGTTTCCACTAATTATAAATAGGAAAAATAATCAAAACWGTCGATTTCAATGGACTTGGAAAAATTTGAAAGACATGACAAAAGAGGGGAAAAAGAATTAATATAAATTCTTACTGATCCAAAACTTGGTTTTATCAAAAGATTAAGTGCTAATATACTGCCATTTTGCAAT

>Cs1g06915

TAGGTTTATTGGGCCTTTTGAGATTRTGGAGAAAATTGGCACAGTGGCTTTGCCACCGAGCCTTTCAAGGCTTGACGATGTGTTTCATGTGTCGGTGTTGAGGAAGTACATGGCTGATCCTTCTCATATCTTGGATTACCAACCTATTCGGATCTCGAAAGATATGTCATACAAGGAGCAATCGATGGAGATTTTGGACAAGAAGGAACAAGCTTTAAGAAAAAGAGTTATACCTTTAGCAAAAGTTTATTGGGCAAATCATTCTGTGGAAGAAGCTACTTGGGAACCAYGCAGAGATTAAAGAGAAGTACCCTCAGCTTTTTCAAGAATAAGATAAGGTTTTAAATCTCGAGTACGAAATTTCTCTTAaggaggggagaagttGTAAAACCTAAAATTTGTTTCATTTTTTTTTATATATACATATTTTATAGAATAGAGTATTTTATACTTATaatatgtaagtgaatgattaaagtttcatttcgattATTTTTCTTTTATATGTATTTTATAGAATATATTATTTTATATTAGTAAATATGTTGAATGATTAAAGTTTTGTTAATTGGAGGAAGRGGTGGGGTGTGTGAGCTCCATAAACAAAACAGTGCGGGCCTTGTTAAGGCCTAAAGCYAATAATATCTTGTCAGGTTTGTGTTGGGTCATGacagtatttttttaacctGCATATGGTTGATGTCTTGTAAACTCGAGCAAGTCAAAATTGGCATGCAAATGTTGTTAATGAGCCTRAAGGCAATGCGGTTGCATGAAACCCAAGTTTGCTTTGTCAAGTTTCCTAGGTTTCCTTCCGCTATATAAAAATATAGCCAGCTAATTAAAAATAAAGTTAATATTATTAGCTGGGAACACAAACTGTCATGCAATGCATTGGCAGGCTCAATGCTCTTACTGACTGAGGGCAGGCTTTAGATATTTCTCTCAATGCTCAATMTTGAGAATGCTTATCACTGACTTTTCTCCTTTYCATCTTGTAG

>Cs1g20620

GAACTAATATCTAAACGTAAGGAGTATCCTCAATACTAAAACATCGACTTTAGGGAGCTAATAACGGTTTTGTGAATAATAGGTTTTGCATTAATCAATAATATTCACATTTTCAATGTTAAAATTGTTAATCGATATATTTGACCAATTGACTGCCCCCAAAAAGTTCGGCACCCGCaattcgttttttttttttttttttttttctctcTGTCTTTGTTCTGGTAATTAAACATTAAGCAATAAGCACCCGCCAGTTAGGATGTTTCTACCGTACTATCGAATCTCGTAGGACTAAAATTTAATTTTTCTTTTATTTCAATGTGCCACTCGAGTGAAAATTATCCAAATATTTGAAGAAATTAAAARTTTAATCACTTGTCTTGAACGTCACGTGATACTTTTCCCATGACAAAATTACCCAAATAACTTGGTCCAGAAGACTGACGTATCTCTCTGCTCTCGCAAAGAAAATTAAACCGCGAAACAAGAAAAGCAAAGGCAGTACCTTTGACATTATATTCTGCAATGAAGAAAAGAAATTTCCATATTTAAGAAATAGTCAAAAACGTTTAAAaawttgaaaaaaaattgaaAGAAGTTGGCAAGGAGCCGTTCTCTTCTTAGATGAAAGTTATATAAAGGCCATTCATTAGCCATTACAATATGCACGTTTTTGAAGCAATTAAGGTTCCTAGCAAGCACCATTATTTGGTAACGTTCACCAAGTAGAGCTAGCTAGACAACATCTTCTCTGTTACCTTCTTCATTTGCATTTACAAGGGTATGTCCATTTTCTCAATTACCATCATACTAAGAATGTTTTTATTTTTTTCCTTTTATGGGGTTCTATAATTAATTACTTGCCACCTTGGCTTACCATTTTCTGCTTCTGAAAGTATATTATAAACGACTCCAAATTGCTATGTTTAAAGAGTGTTCTGTTCACGTTMGGTATGCATGTTGTTGTTTTCAGGCCTTCGTTCATTAGAAAATAGTTA

>Cs1g20760

atcatgttcaatgtagttgtactacatcaataaattcatacatgattaagacaaatcattcaatgaatttattacagtctatacctaaataaagtgcccaactttatttatcaactgcgaactaaatttatttaatcataagataacttgtatttatgtcttctgtgaatccacatggtgatcacataaatacatataatatgattaaatggactttaataaaaatattaatgtaattaagatatttgaataaaatacctcattaattttattaatcagaaaaaaaatttattacagttaaataaacnnnnnnnnnnnnnnnnnnnnnnnnnncaacactaagAAGTAATTATTAATTATATTTACCTATGCGATCTATGAGTATGCAACTGATGAAGGTGATTAGTGATTGCTTCTGATTAATCCCTTTTCAAATTCTTGCAAAATTAACATAAATAACTGCAGAAAAGTGGGCTGCCGGTCGCTGGGAACAAACGTTAAATTCGAGTTATATTTATAGAAATTTTTACTTTCTATCAAATCATGTTTCACTGTATTCATTAAACTTAATTAACCTGTAATTTATATGATCCATGRAACTTTGGAGTCTTGtttattaaacgtgaCCAAACATAGATAAATTTGAATAAAACGTATGAATTGGAGTATTACTAAGAGAAGCTAAAAGCCATATTCATGATGTTGCATGCATGGTAACTGACAAGAAGAAAGACGAAAGTGGTCGAGGATGAAGGATTGCTCCGCCTTAATATTTTAAGTTTTtttataacgaggatgaTGACTACTTTCCACTACCGAAACAAAAATTAATAACACTATTAATWGCAATATTTTAATTAACATGTAAATTGATTATCAACTTTTTTGTATTATTCTAATTCCAACGTCGCTCTCTCATCTTCCCACACTATACGCTCCGGTAATTTCCTAGCTTTTGCAAGTCAAACTCAAGTTTTCAAACAAAACCTTTCTGTTTTCTT

>Cs1g17470

AACTAATTTGTGCACTCAAAtaagaaacacaGTGATCAAATWTTTTTAAGTGCGATAAAAAAATTAAATTTAGAGTAAAAGACATCCAGCTCTTTGAAGGTAAACATACGAATAACTAAATCCCGTCGTTTTAAAAATAGACAGTTAGATGTGTTTCATATAATCGACGTTCAAAACTCTYATAAATTATATATGTTTAGACAYGGTTAACGTATGGGCATTAATATGCTATTGTCCTTCAAGTTTAATACACTAATCTTTATCTCTATCCATGTAGTTGAAATTTATGAACGAAAGGAGAGGTGGTTGCACATTTTTAATATTGGGAATTTAGCTCACCATTTGGGCAAAGCCCTAACGGGTCCGACCGTCCGAGAGTCATTTTCTCTTGACTTTTCGTCGGCAGCCAGCACATGATGACAACAAARGCTACATCAAAATTCGGTTCGGGCATCCTACCGGCYGAGTAGTTTGTTTTTGTTGAATTGGGCTTCGGCTTGTGGTCCATCGTCTCTTTCATTCGATTTTATTTGGACTTGGCACTCATTAGACTAGTTTACCCATTCCTCATTTGGGCTTGGGCTTAGGCTCAATCCTAATGGACTTCTAARGAGATATTAATCCCATAATACCTTAATAAAATGTTGTAATTCAACAACGATTGGTAAAGGCGTCCTGGTTATTGACGGACACGATGATTTGTTTACTGTCTACTGAACATAttttcaaaaggaaaaaaaaAAAAAAAAACATATTTTCCCTCAATGATTCAGTGATAAACGTAGGCCCACTCAATCACACATCACAATCTTTGATGTTTTCTTCTACTCAACAGATAAGATCTTGCCACGTGGSTGAATCKYCCTTTRAATTTATGGAATCAAGAGCGTCCAATCACAGCCAATCGGTGCCTCAAAGTCTGTTTTCAACATTGAAWGGGTATTTTAAGCAATTAACTCCTCGTTTAAAAARCCCCTCTTGAACTCACGTCTTCCATTTTC

>Cs1g23900

TATCACTTTCTCCTGCAACTTCAGTTTGGAAAACAAGTTCATAAACAACATTCTTCCGAAATCATTCTTCCCATAATCTATTCGAATAATAATCAATTTTCTACTGGAATCTCAATTCGGTGAAAATTTTCATAAATAAATTCCTTTCGAAATcattaatgctcgtaGATTTGAACAATGATGATAGCGTTTTGCTTGAGTGAGGCTTTGAGCTTTCACATTTTAGGTTTCTGTCGATTGAGAGTGCATGAGGCCATGAGTAACGATGACTAGTGTGTTATGTGCTGAGAGTAAACACTTTGtaaaattttaagtatTTTAGAGAAACTAACCCAAAACGCTGTgcttttttttttTTTCATTTTTTTTATCTGTTAAAATTTTAAAATTCGGTCAAGCATAGTTAAAAATTAATTTCTATCACAAAGTTTATGATAAAKAAAATTTCTATCACCAcaatattatacatATTTTATAGACAATAATTCTATCATAATTTCAATGTTAATTTTTTGATAGACCATAATTTTATCACGAGTCCATCATATTTTTATGATATAAGTGAATTTKTCATAAAATATATGTCATTTACTATTAGTTTTCTTGTAATAACATATGTCATAATATCATTTGTTTGTTATATGGCTAACATCGTATCTCTTATGTATTTTAAAAATTTTCAAATAGTATATAAAATATTAGAAATTTGAGATATAAACTATTCATATCAAGGAAAGTGTATTATATTATTCATCCTATGAAAAATCATACTAAGAAATATTAACTGGTAGCTTAAAATAGAAActtataaaaaaaaaatcaaatCAAATCTTAGGTGTATATAAATCATGCCCACAAGACACCTAAAAAGAAGGAGAGCCCTTAGGCAACCATCAATCATTCTCTAACAAATTTGCATTTTTGTTTTTAACTCTTCATCGTCGATCTTTAACTTCTTGAATCTTTCTCTTCTTCTGCAAATCTTTTAGAATTTACGTGCA

>Cs1g21550

TTCTTCKGCAAGAGCCTGTTGCTGGGACGGTGWGAGTKGTGCTTGGGACTGGATTTTGGCTTGCTTCCGATTGTGTTTCTGGATTTCCTTTTTGCGGATGCTCCTGAGGATTTTGGGCGTCGGAAATACGCCRTGTTTGAGGAGTTTCTGTTTGATCTTTTCCATGTTGGGTTCGAATTGTGCTTGCGCCGGAGCYGGCAGTTGAAGAGGASCTGGCTGTGCTTCCATGTCCACCAAYCAACTACTGAAATCGTTGTTATTAGTTAGTRTGTTTGGGTTTGGCAAGCAAARCTGAGTAcactatccgcttagaGGTTTTGGCTAATATTTTACGTGTGCCGCTGAGATTTTGGCTCAATGCCGAAGATGCACACACTATTTCAGAATTAGTCTCAAGTACCCTCCCGTGATGTCATCATCAAATATTGCATTAGATatttgtcnnnnnnnaaaatatacattgCATAAGATAATGTTATTTTAGTTGTCTTTGCATGGGCGAAGGTGTCTCACCCTAGTGGCACAACTCATTTATTCAAAGCTTTCTAATCATTGTTGGGATTTTTTTTTCTAAATTAAATTGaatatgagtagatttttatcataataatataggtagatattatgtnggatagaaatttgaaatgtactaaactgaatttattaaagaaaataatcttaatactttttattatgaacATTAATAATATTTTAACTGTAATATATTAATGTATGTGTgtgtgtatataaAAGACTCATAATCTTCACGCTTACAATTTCACAATATGGGTTTGTTCGTAAGATTATATATATATATATATTCARGAAATCAGATTTGAATTTCAAATAAAGAACTCCTGAAGAGTAATTTACAGCCCTTTCAAAAGAGTTCACTtttcatttttagttacTTTTTTaaaggrtctactcaattatAATAAGAGTAACTTATGGCCCATAAAAAAGAAAAAACCCTGCGGGCCGTAACTTAAAATTCTCAC

>Cs1g18420

ATTTGAGGAACTTTATTGCAAAAAATTTATTGTGAATGTAATAAATATTAYTTTTTATTTTWTTTAGATTATGTATaagtatnnnnnnnnnnnnnnnnnnnaggtaataGATTATGAAGTTCTTTAGttttttTAATTGAGTTTTTTTAATGTAATTTCATATATTTAAGAAAAAAAGATATGTCTAAACAATTTTTTAAACATACTATGAAAATAAYAAGTAATTAAAAATTTATATCTATTTTGRTCATTATATAATAAAACAAYACTTTCGTAATAARATTTATYAAACRCATGTAYTTTACTTTTYMAACTCACARCAACYGTAACAACATAGTTTACCAARTACYAAGTTACTTTTTTTAATCAATAGCTTATTTCATCTATACAGCATAATCAACTTATTTCATCGGCCTCCACAATCTCAAACTGGCCCTAAGTCTAACAAtaattaaaattttttTTTTAACAAAAACATTCAGGGATTTTTAATMAATTTGCTACTTTTATAGTTACAACAAGTCAGGGTTATTCACAATTCATACTTAAAACTATTATTTAACAATTTAAGGACATAACAATAATATTTTTATCTACCATTTGATATCTTTATTTGTGTATTTTTACTATAAAAGATAAAATTAATATTTTACTAATCAACTAAttaatcaactaatagttttaattggaaagttataaaataaaagattttataaggttatggtagtaaaatatactttttacagtcatacaatattttacaattctttaagtagtgaaacaatgagaatcttttaataatcatacgtttatcaaatttataaactaaaaaaaaaaaaaaacttatatgaaaacttatctttatcaaattttccatttgataaagaaaatttacaagaacAAAGAAAACTTACAAGAATAAATTTTCCGAACTAAACAACAAATTATCTTTTTCTCAAAGTAATATCCTCATTCCAGCTAATTTTAgTTATATACATAAC

>Cs1g15680

TTAATTAAACTCTATTGTAACCTTTTATCATTTTCTGGAATTTTCATTAAAAAAGAGCTATGCATAGTTAGGAAGTACGGGTTTCATAAGAATTTTAAAAGGGTGTTTAAAGCTTTTCTGTGGATTAAGGAATCGGCCCAAAACAAAAAGGCCATTACGTAAAACGACGAAAAAGCTGCTGAAGTAAAATATAAAATCGCCCACCGTGGGGCTCGAACCCACGACCACAAGGTTAAGAGCCTTGCGCTCTACCGACTGAGCTAGACGGGCACCAGTTAAAATGCTTACACTGAGAAAAATMTTATAGTTATTATTTCTTTAAATTTCTCTATTGTTTTTACATTGAGTGGTCAAAGTAAACTCAACCCACTGAGTCCGATCCAAGGGTTCAGATAAACTCAACCGCACCGCAAAGGCACAAACTCGCATGTACCATGAAACCACTCACTGCATTGCAACGCTTGGACCTTTTTACACTCATCCGTGGCTAGGCTTGTCAGAGCAAGAACTCCAACCCCCTTTTCGTCATTTCACAACTTTCCTATTTCATGCAGTTTAAACAAAATACCAAAAATTTCARCACCGTGCGTTTCCTCCGCATCGACGACCCACATCCCTCCACGCAATATTTTAAATTCATAAAAAATTCGCAATTAATTACAATGCCTCCTTAGTTtcttctttttctctttTGTAAAGTAAGTACTCCTGTCTCTCTCTGTTTCTCTCTCCAGATTCTCTTCTAAACAGATCCCTAAATCACTAACCCTAATTCCTCCGACTGTGATCACTGATTCTTGAATTCTAAATCAAGATTCACGATTAATACCCTTTTGAGTTTTGCAGTGTGAAGGATTTCAATCTGTCGTTGGAAATTTAAAAAATTTGTTACCTTTTTCTATCGTGTGTTACAGTCCCGATTGATTGCTTGAGAATTAAGGTTTTTGTTTTGCAAATTTTTTTACGGCAAATAGTGATTATGTACATTGAATGATGAACGA

>Cs1g19450

ATACTAATGCAAGTTTCATAATCATACAACAACAATTATGAATACATTRATAGTCAAAAAATTAATTTTCAACATAATCGATTCAATTCTTATCTTGATTAAAGAATCGTTGAATTAATTTTTAATTTAATTACATTGGCAGTCAATAAATAcgtaataattaatttttaaaaatatTATAAAATAATaAcaaataaataaTATATATTAATTTTTCAGTATGAGAAATTTGACAACCCCgtttTGTAACTTAAAAAATAACAAAACCTTTTTCCCATAATGGGCAATATAATAAAAAAAGAAAACAAAAGAACATTGAATAATTGCAAAAATCCAAGCAGTACAaAATACAGACCATAGAAAAATGTCAGACTCAGACAAAGCAAGCTGTAATCGCTTATTGGAGAGAGTGAAAGAACTGGGGGAGGAGCGGGGGGCGCAAAAaatgttaaaaaaaaagggatttttgatttgaaatagaaaatctgaaagtgattctagagggaagagaaataaatacactcttttttaatctctaaacgaaaatagcgacttttttctttttcacgttttctcaaataataacataaattatccatatatatcacaaagacctaactccacataattgaccaaaaataactgttaattatgttcgaaaaacttatatgagaaggatacactattattttttagcagtcatttaattaaattaaaactaaaggatgagatctcttattgaaacgtaattgtatatatagtaatattttaataagaaattcttcttttaaannnnnnnnnnnnnnnnnnnnnnnnnnnnnnnnnnnnnnnnnnnnnnnnnnnnnnnnnnnnnnnnnnnnnnnnnnnnnnnnnnnnnnnnnnnnnnnnnnnnnnnnnnnnnnnnnnnnnnnncattatataatatgattgtatatataatagtttgcatataaatgagaaacttcttcagcaaaatacaaactttctcctcttaatattat

>Cs1g02890

AAATCATTTGTTTAATTTAAATAAGTTCCACAGAGATTCCTCAATCCCATCACCTTTCTGGACATGCCAAGAGTGTGCGGAAAGCAATGTAGTGTTTCCGCACACGCGCGCAAGAATGAGATCATGCGACTATTATTAAARTATTAAAGAAGTTAaaatataagaagattattAGGCTATTAATGATAatctttttttcttttttgtcCCCTTTCTTTTCGATATGAAGGTATAATTATTCCTTAACTCAGGTTGTGAGCAAGTCATGCCTTTATTTATATATATGTATATATGATTATAGAATAATGTATCGCCTTGCTTCTTGCATAATCTGCTCATTCCTCcctattagcagcaacCTTGCCTGTCCTTTCTAGGAACTGGCGCTGGCTgcattattcttataatttaataagtaacgagtattctatccnnnnnnnnnnnnnnnnnnnnnnnnnnnnnnnnnnnnnnnnnnnnnnnnnnnnnnnnnnnnnnnnnnnnnnnnnnnnnnnnnnnnnnnnnnnnnnnnnnnnnnnnnnnnnnnnnnnnnnnnnnnnnnnnnnnnnnnnnnnnnnnnnnnnnnnnnnnnnnnnnnnnnnnnnnnnnnnnnnnnnnnnnnnnnnnnnnnnnnnnnnnnnnnnnnnnnnnnnnnnnnnnnnnnnnnnnnnnnnnnnnnnnnnnnnnnnnnnnnnnnnnnnnnnnnnnnnnnnnnnnnnnnnnnnnnnnnnnnnnnnnnnnnnnnnnnnnnnnnnnnnnnnnnnnnnnnnnnnnnnnnnnnnnnnnnnnnnnnnnnnnnnnnnnnnnnnnnnnnnnnnnnnnnnnnnnnnnnnnnnnnnnnnnnnnnnnnnnnnnnnnnnnnnnnnnnnnnnnnnnnnnnnnnnnnnnnnnnnnnnnnnnnnnnnnnnnnnnnnnnnnnnnnnnnnnnnnnnnnnnnnnnnnnnnnnnnnnnnnntttcccccccCCCCCAAAAAAAAAAAAAATAACGATATTAAGTCAACCA

>Cs1g03270

GTAATCTATACCTGCATCTATTATTTTCGTATTTTTGTATGTCAACTACCATTTKTAAATGTCCTGGTGAAAATAATTTTAATCTCGTTTATTTATCTTTCAATGAAGACTTTTGACGTATTTCTCTCAAGCACATCGGAAATGAGACAAATAGCCCTTCAAGTTTGAAAGAGTTACAAAAGAAAAGAATGATAGTGATGAAGTACATTTCAAATTGTAAATTTATTACACTACATACGTACATACATAcatatatatatgnnnnnnnnnnnnnnnnnagtgcaATAAATTCAGTTAAAACCTGATAGAACAAATCATAGAAAATTTTTGAGTGCTCTTAAAGCATTATTTTTTTTTCCCCATCCGATCCTTATTAATTACCACTTTTACGCTTCTCATAATTAATCACACGATATCGTCATTAATTATTTAAGGGTCGCCATTAATTATTAATTTAagggggaaaaaaatataaCGCTAAGGTAATGAAAAAATCTTATTAATATCTGTGAACTCATCATACGGAAACATGCATGTTAGATTACAGATGGCCGCAAATATTCTGAATAAGCAAGGACAGCAATGTATGAATCATGCGATTTAGTGATCACTGATCAATCAAATTAATTAGAACTCACCCAAGATTAAAGAAAAATTTGAAAGAAACAAACAAACAAAAAGAGTGAAAATAAATAAAAGTCTATCCATACAAAACATGCAATWGATTTGAACCAGTAGCCCTAAACGCACCTGCATGGatgccagtgcaGATACAAWAGACAGCAGCGAATTTTCACAGAAACTTGCGTTAAGAGAAATAATTAAATAACATATTGGGGTGTCTGTAAATAAATTAGCCAAGGAGCTCTCGATTAAATTGTCCACGGCCATTCAATTCGTTGAATTATATATAATACTTACAAAACACTCACGAATCCAGCAAGGAACCATTCTCAAAACTTAACTTTCACACACGAACACTCacatacaaaaaaaaaaaa

>Cs1g12700

ACtttttaaatttgtgTGTAGATTAATAATAAAATTGAAAATAATTaaaaaaaTAGCACAaTAATactttGAGTTTtaAcaataaaacaaatcaaATAAAAATTAAAAACTTAGAAAAATGTATATAAAAAAggggcATTTTCGGAATTAAAATGAATATAACTAAAAAAGGGGTTTTATGAGGAGATGAGAGGGGTGCCAATAKTCCAACCCTTAATATTATTCTTAAGTTGTTACCTAAAAGATATAAAAGATAACCAATAATTCGAGGAGTGTTACCACAAAAATTTTAGGAGACAATGGCTAGCATAAAGTTTTCAAAATACATGTTTTTCCTGAATCTGATTATTTGTTTTACAGTGTGAGAACATTAAAAAATAACTAAGTAAACAAATATTGAACTATTTCAATTACCGACATAAATATANCAACTTcttgttattattattattattatttttaatannnnnnnnnnnnnnnnnnnnnnnnnnnnnnnnnnnnnnnnnnnnnnnnnnnnnnnnnnnnnnnnnnnnnnnnnnnnnnntatcccCTTCTGGAACTTATTTAAATTTCTATGCTAAAYTCATATTACAAACAACTAAAATTTTGGAACACTAATCCTAGCAAATTTGTCTGAGGGTTAAAATACGAAAACCAACTAAAAAAAATCAATATCCACATCTAGGACAATGATGTTGCATTATCGTATATGAATTACATTTTATTGAAAAAAGAAAATTGATAACCGAAAACAATGATATGAACGTTGCAGAAAAGTTATTATTATTTTCAAAGATTCAACAATATAACCCGGCGGACTGCATATGGAACAATCTTTAGAAAGAGCGGTTTCATGAGCAATAAATCAAARGCACCAAAAATAGCTTACCAGCCTAGCAAGACGTGCTCGTTGCCACCCAAATtctatcccccccctcccgtCTCCTTCTCTCTCTTCTGGTATTGTTCATCCTGCAATCTTTCTTGCCGGCAGGCCGCCA

>Cs1g20690

caccctagcacaaaccctcagtccacattttgcttaacaaacaaacaatatgccctagtgcttttaaaattaacctgaaacacaagaatttggtccatatgttaggtgctccttgcaagttggcaaatctcgaagcccaacgaagataaatgagtgattttatccaggccgtgtgtgtaattccaggcccatatcagttgagacttttcgtgaataataagacatggatctaaaattcacgcccagaagacagactttgagtgaaaataaaatgtctgagaaagtgacacaatgcaggagtttgagactctgtgtttggtggtagaagaaccgttcaaatcaaagtttatgtggtcacatttgcctcacaagaacaagataggatatcccacattcactttgtggctaagaaacggtggaatttggatagggatttcacggctggtttttataggccttttatcaatttagttttttcaatccgaccttagcttttcgaattttagtttcagtattttcaatcaacattgtaatctttattgcctagcattttcctaataaataaagaattgatgcatcggacacccattcatgtacacacgatgatgataaatttagaacgcatcttaatcatcgtaaaccatacacatttacttatttaatttttgtcatcttcgatattcgcGGaacctaaaattaagaaatttatgaactagaaattatagaatttcgaaagaacaattttatcgcctttacttgatgtttagcttaaaatttataaagaatttactcgttgttgactagtcatttatttttgggtaaaattaaaattataaatnnnnnnnnnnnnnnctcgattgattgaattaagtatctctaaattcatatgatttgtttttattactctttatttttgttatagatcggtgatttacaataattgagaattatataatccttaatttatagttgctaaatttggtgagtccgttgtaggtgag

>Cs1g09440

gattggggtatcgagcgagtgtttactgtaagtgttgacaatgcaagtccaaatgagggtgctcttaggtacttgattgatagagtgaagacttggaggggtgatggcttagtgttgaatggagattatttgcatgttcgttgctgtgcacacatattgaatttgatcattactgaagggttgaagaaattagagcagagtatagttagtgttcagaatgtggcaaagtatgtgagatcttccactgctagaatgcaagcatttcaaattcgtgtgcaacaagagaagattaattgccgaggaagtgtgattttggattgtcccactaggtggaattctacatattccatgttgaatacggcattaaaatttaagccggcatttgatcgaatggcacttgaagataagctttatgatgcttatttcaatgaaaaagagggtgggaagaagaaaagggaggggccgccattatacagtgattgggaaaatactcaacgcatagtcaagtttttaaagacgtttcatgatgcgactttgcagttttcttcatctttgaaggtaacatcaaatctttattaatttcttttaatttttnnnnnnnnnnnnnnnnnnnnnnnnnnnnnnagctttattaacattgtttttatttatataatagaatacaaaagacaattaaaaaaaaaaaagaaattctataagctatattttctatgagctatattttctatgagctatattgtttttaatttttgatatataataatatgatgaagtttattttagctttattaacattatttttttttaggtgacatcaaatatttgttacaatttgataagtcaaactgaacaatctttgggatcattgtccactagtaacgatagtnnnnnnnnnnnnnnnnnnnnnnaaatgaaggaaaattttaataagtattgggatggttgtttcaagataaataagttgttgattgttgcttccatacttgatccaaggtgaaaaa

>Cs1g16520

CCATTTCCATAATGTTTTAAAGGAAGACTGATGAAAATTTTAAGTGCTTCCAGGGTTCAGATGATGatctaaaagaaannnatataaaggaAGAGAGAGAGATTGATGCAAAGTTTTGTGTGACTATACGGGTATATTTGTAAGGCTAGATAATTTTGTCTTGGGCTTGAAATCGGAATATTAGATCATCRAGGATTATTTTTTAAAAATAAAAAATTGTTGCTATTAAACAAATCATTTTGGAAGAAGATCATGCAGGAAAGAGTGAAAATATGATAAAAGGAATTAAMAATATTTAAAATTTAAAAGAAATTCATATAATAAATTAATTTTAACCATTATATTGATTATGGTTTGAAGACTAAGGGCCTGTTTGGAATTGCTTTTAAAAAGTTCYAAAGTGATTTTGAAAAGCCAAAAGTTAATTTTGGTATTTAATAATttttaatctctcttatatTTAACACAATTCTATATATATCATAATATAAAAATATTCCAATCTAAGATCTTGAATTTGTTCAAGTCTGATAAATCTATTAAGTCATATAGTTAATAGTATYATGATATTATATTATTAAACCATATTACTTGATAGCATTRSCAGATTTCATAAATGTATAAGTTCTTGAACCAAATTAGTACTCTACCATCGTATATATTATAGAAATCCAACCTTATCCTCGTTAACTTTatgttgnnnnnnnnnnnnnnnnnnnnnnnnnnnnnnnnnnnnnnnnnnnnnnnnnnnnnnnnnnnnnnnnnnnnnnnnnnnnnnnnnnnnnnnnnnnnnnnnnnnnnnnnnnnnnnnnnnnnnnnnnnnnnnnnnnnnnnnnnnnnnnnnnnnnnnnnnnnnnnnnnnnnnnnnnnnnnnnnnnnnnnnnnnnnnnnnnnnnnnnnnccgggcccaggcccaggcccaggccaacaccgcctaggccgaatccgaaatggttgattcttccctttCCTCAGCCTTCAACTTTTATAATTGTATTAAA

>Cs1g08620

acttttttagtttnnnnnnnnnnnnnnnnnnttaattTTATTGGACTTTCAAATAATATAAAAATGTATTCACATAAATGGTTGTTgcactctttttttttttttcaactTTTCTTGCTCATCATTTATAGTTAAAAATCTAAACTTGTGCTACATTTCTATCCTTGCTGAACTAAAATAATGGTACTCGATAATATGAGATTTTCCCATTTTAATGAAGTGCTGACTAAAATAAAAATATAAAATAAATACATACTCTaatatgaactcttaTAACTTTACGTAGATTGTTTGCATCTTGTCATTCAGATGAGTTAAAATACAaaaaaatatgaaCTCTAATAACTAAAACACAAAAATATATATACTAAAACGCGTACTTTTGTTGAATCTTAATTATATCCACCTRTTTTTTWAAATATAAATGTCTACGATAGGTAATTTATCTATATAtactatttacatcaaaattcaagattagatttggaggaaattataaatgtatacgaatgaggagaaaaatgaaaagaAAATATATAACACAATAAAAAAATTGAACTTAAGGGAAAAAGGCAACAACATCATCTCATAGTTTAGCAAAATAGTTGCAGAAACTCCTTGTCTAAAAAAATAATCACACTATATTGATTTAAGTGTATTTCATTTGGACATAATTATTTAAAAAGAAGTATTTGTAAATATTTATAAAAATAAGAAGTTATTCGGAACCATTTCATTTCATCAAATTATAAAATGGAGCTGTCTTTTTTTCCATTTTAACATAGAGCACGTGCGATTAACGTTAATTTAATTTGTATTGGTAGATGGCTTTGTCATGTTTTGTTTTTATATATATTTTTTCTGTAATTTTTCTCCCAATTTATATACCTGRATAGGTAATATTGGAGAAATTTTACAACTTGTCAAATCTATTGAATAATTAGTGTTAATTTCAACTCCATCAATCTCAGTATCTCACCAAAATTTTAACTTTTTCTCAC

>Cs1g14390

ATCTTAGCCAAAAGGCCGAGAAAGGTATGCTTTGTTAAGTGTCCCGATTCTGTTTTTATAGCTTCCGTCTGCAACTTGCTTAGGTTGCCCTGTCGATGTGGGAAAGAATTTGCTGCATCTGATATATAAATTAAGGAGATCATGAGAATGGATTGTGTTACCTTTCGGCATTGAGCTGCAAGGTATTATCATGAACTGATTCAGTTATTTTAGCATTTTTAGCCCATGAGtaaaaagcctttcactcaaccttcaacctttccttgttgttgtaactcacattaagacgaagcatagatggatcgtttaaannnnnnnnnnnnnnnnnnnnnnnnnnnnnnnnnnnnnnnnnnnnnnnnnnnnnnnnnnnnnnnnnnnnnnnnnnnnnnnnnnnnnnnnnnnnnnnnnnnnnnnnnnnnnnnnnnnnnnnnnnnnnnnnnngccaaccttttgcttcatcaccaagaagccgaaatggcactggataagggaacttaaacggtaaaaatttgaaagaaaaagctgctttatcaaactggaaaaggattcttttcccgtcnnnnnnnnnnnnnnnnnnnnnnnnnnnnnnnnnnnnnnnnnnnnnnnnnnnnnnnnnnnnnnnnnnnnnnnnnnnnnnnnnnnnnnnnnnnnnnnnnnnnnnnnnnnnnnnnnnnnnnnnnnnnnnnnnnnnnnnnnnnnnnnnnnnnnnnnnnnnnnnnnnnnnnnnnnnnnnngacaccagaaattgtgttttcaaaactgcaattgatttatcactagagtttaagatcatcagcctattatagaaattaggggtgggcatttggttcggttcagtttgaaccgaattgaactgaactgtattataataatttagttcggtttgattcgcattagaaaatcaaatagtttaaaatttctgaaccatttggtttttaatttggttcggtttaaAtccgaaccaaacggttcagtgaaannnnnnnnnnnnnaaaaAAAGT

>Cs1g25660

tnnnnnnnnnnnnnnnnnnnnnnnnnngggcacgaattttcggtcaagtacaaacaagtaattccggcgaaccagaacagtggcaaactcgattagaaaaaaacagatgaaaggaggaaagggaAAAATTGCAAGTGCTCTGTGTGCTGGTTTTTGTGTTCGTTAAAAAAaaaaagagggaamGTGCCTTTTTATACTCATGTGGGTAGGTAACAGCAGCAAAAGAATTSAATTTCGGAATTCAATATGTGCAATTTAATTGCTGTAACAGCCAAAAATAATTGTAAAGTTGTTACAAATCTTTTGAATTCAAAGAGGTGTGAAACTATTACAAAAAAAATTCAAAARGTGCCATGTAAGCTTGGATGATTTTGGGCTGACATTCGTGTACGCACTCGAGttcaaccaAATCggttttgaatttnnnnnnnnnnnnnnnnnnnnnnnnnnnnnnnnnnnnnnnnnnnnnnnntgcatggttaagtgtttatatataaacactaatcccttggcttctttttcccatgtgagataagtctcatttcctttcaaatttccaacttcaaaggtaaactttgagagacaatttctcattcactcaagcaccattttgagaaaataaaattactcttttgaagtattaacggaatagaatccgaacctcacaagatttCtcActtTCGCtAAGtgagacccactcaAAATGTGCCttAAAATAGCATATAGGCATGCTATTTTTTCAACAGTTTAGTCACGGGTAAATATAGTCTATGCGAATATAATGGACTCTAGCTCTGAAAASTTACAAAAGTGAAGAAGCACAGAATTTAGCCGAGAATGTTGATAATGGCTTGCCGTTTTCTTTTTCACTGTCCATGCAAAAATTGGAAAAAAAAAaaaaaaaaagctcaaACATGGCACGAACTCCGAGCCACTGCGGACTTATTCACATCCTAAACATAAAATTTCAATTTGATTTATTTTTCTTGTAATCTAATCTC

>Cs1g22550

tattttgcgaaattattagaggttctaataatttattcattgtaccaTCTTtctagtaatttattagagtcataatatatttatgacatataatatctcttatctaaatttagcatcttgaattatataaaagaaaaaaaaatctaatttatgtcttaagnnnnnnnnnnnnnnnnnnnnnnnnnnnnnnnnnnnnnnnnnnnnnnnnnnnnnnnnnnnnnnnnnnnnnnnnnnnnnnnnnnnnnnnnnnnnnntataaaacaaaatcatttagttataaaaaattaatgagaacttatttaaaaaataaataaatatatgattcaaaataaaattttctgtaaatagtcattacacccatactttatttttcgttaaaattattttcatccattaaattaaaaaatatcaaaatctaactgcccatatgtactgactgtttgttacagctgatgtaacatnnnnnnnnnnnnnnnnnnnnnnnncagaaagttatcaataaaaaaagacttttttaatgtgaagagtaagcagcaaggttctgaagaaaattaataggagactgtacagtggatttcaagagagtgaaaaattgttataaaaaaaagacataatcataaatcaggagttctgattttatttttaatataattattttgagcaataggagagtttattaaaaagggtattattttaatcnnnnnaaatatAAGTGAAAAGGGAGTTCTGTAGAGGACATatgagaggcgtaaATAGTYGGTTCACTCATAAAGAATAGAGGACTGTGTAGTGTGCAGTGCGGCCCAATTCAATAATAACACGCCCGACCAGGCTCGTCCTTTAGGACCCACCGTCCTGATTCTGACATGCAACCATGTGCTGTCTTACTCCCCAACTGCCTCTTGTAACAAATTAACAACAGTGTCCAGGACTCATAAACAAAAAGGCCTTGCCTATTTAATTACATCAACTATCACATTCAAACCTGTATCGCGCTCACT

>Cs1g21440

TATATCTTTCGATGCGTATCATATGAATTAAGTTTCTTTATTTTGTTCCTATATATGATATTTTAGGGCTTTTAAAAAAAAATTAAATAGATTTAAATTTAGTTGAAGGCTGAATAGGCTTAAATGCAGGTATGTGAATGATATTAATTTTTGTGAATTAAAAATATTTGAAAGGTTGTGTTTTGATATTTATACTCTtataattatatatatatatagagtaATTTGATAATCAAAATTTAATAAAAGATTTTTGACATGCCAAACTCTATTTTAAATTAAAAATTATTTGGTAAAATTAAYTAAATTAAACTCTTAGTTATACTTCTTAAGCTCTTTCTCTCTCAGGCTCAATCAGAGTCACTATTTAATATTTCAATCTAACAGTTTCAATAAGAAATTGAAAATTTGTGAGGAATTCTTTTGGTTTTCACATTAACTAATTATTATCTCTAAATATCGTATTTAGTTCGTAAAAAAAATCAGTGTTAAATATAAAAAAAATGGAATGATAATTATAAATACAAAAATTAGATTGTGATTATTTCTCCTTAAATCGTAACTTGACTCTTAGTTTTGCAGTCACCCAAATTCGAATAATAATACAAAATAAAYGTTGGTGCTTATGAGGTTTCTCACAAKAATGACTACTTAAAAATAATAGTAttttttwaaaactaaaATTAGCATTAGTTGAAGTAATAGTATCTCACAAGTGAtaaattaaaaaaaaaaaagatctAACAAMMGATTTRGAATATACGTTAAAACAATAAATAAATCAAAATATGTATTAAGTTAGACGTTCACAATTTTGTTTTTAAGTTGGAAATTCAAATTATATAGTAGGTGTGAATCGTGTGATTCATCCAAGCCCTTGTTCTCCAWGGAATCMGAAAGCAAAGCTGAAACGACCCTTCGTGGCAGTTAGACATTGTGAGCTGTGACcaaamcttggtttAGCCCAGTCTCCGGGAGTTTGCGAGTTGKGACTACGACCC

>Cs1g25980

CCTAACCATTTGTCATTCAATAAACTGATAAATAGGGTGTTCAGATTTTTTTGATTGGACATGGAGCATCATCTGAAATTGCATAACTGATTATGTCAGTGTTATGTTCATATTCCTGTCATTTTTTTCCCTGTTTTTTTTTtgnnnnnnnnnnnnnnnnnnnnnnnnnnnnnnnnnnnnnnnnnnnnnnnnnnnnnnnnnnnnnnnnnnnnnnnnnnnnnnnnnnnnnnnnnnnnnnnnnnnnnnnnnnnnnnnnnnnnnnnnnnnnnnnnnnnnnnnnnnnnnnnnnnnnnnnnnnnnnnnnnnnnnnnnnnnnnnnnnnnnnnnnnnnnnnnnnnnnnnnnnnnnnnnnnngggtgttgcggtttatattcctttatattaTTTTGTATTTTTATTGTTTACAGTTGTGTAAAGTTGGTGAATTCTTGTGATAAATGTATGTTTGGTATATTTTGATGTCATCCTAGTAGTTGGATGGAATCTAGTCACATTAAGTGTAACTGTTCTTATGTTCTATATTTCAAAAGCTTGACTGGGTAGCGTTTTTTACTTTCTTTCCTACAATTACACTAGGGATGTGCATGTGCTGAACTCTGATGATATTTTTGGGCATGCCATGTATATAATGTGATCACATCTTGTTCCACGGATAGTTCTCCATGATTTCAGGTTTTGAGCATTCACCCTCATTTGACCGTCTGTTCAAGAAGCACTTTTTTATTGCTTGTACAGTGCCAGCTCCTAAAAGCTGTATTTTGTGTATGATTCATTATTGCATATTGCCAGCTTTATATAGCATATACATATATGGACGTTCAAAAGCAGTTATGCTGGTCTATATTATTGGGTGATATTCGAAGTTTCCAACACTTGCTGCGACAATACATACATATGCCTTATTAGAAATGGGAAGTAGTAGTGCTTGCCAAAAATGTGAGCTGGGGCATTCTTTCTCTTGTAAAAGAAGAAAGGCATAAAAAATCAACAGTACATATTGT

>Cs1g04970

atcaaatgaaaagactaaattacccctctagcgttatcctatttaaacgacaagtaacagtttggtggacggcagatacattttgtcaacttagggtggaCGATTGaTACaCCATCAAAGTGTTGTAGTATTTTTGATAACAGAGCATCTATAGGGTATATGAATGATAAtttcccatatatatatatatatatatatatatatattatttAACCATTGACTACTTTGATCATATAGAATTTAATTATACCCTTATAAAAATTAAGGTAGAAAAATATTATTCACCATCACAGTTGGTTCCAAAAATGTATTTCTCATTTTGGTTCTAATCATTGtaagttaaaaaaaaaaaagcataCAATCACRGCTAATTTTATTGTCATGATTACATATGAATTGCTCAAACTTTCTATTTCAACTATCGAATKTTWTAGCCYCTTCTAAACATGAAGAATTTAAAGCTATAATTTGTAGGTGCAATTGTATTTGCACATGCCATGAGACAATACTCATTGACATTATTTTAATTTTTAATAGTTACTCTTTTCTTTTCTTTTTTTCTCTCTCCTCtctctcccTCATTTCCCCCSTTCTTTCCTTCATTTTCTCAAATCTCAAACGCCCACtataaaatattttttTTTAYATTTTATTWTCTTAGAGAATTATATTTTGTACATGATGAAATAACTTTAATGGGTAAAAACTGAAAATTAAAAGATAAGATTATTTAAACCCATAAATGTATTTGTTATACAACGATGGTTTAGAAATAAAAAAATATTAAACGAAAATGCATTTGAACTTTAAGGAGTGATTTTAGGGGTTAGAGTAATYAATTAACTGTAATTTTTTTTTTATCACAATAGAGTCAAAATATAATAAAATAGAGAGTAAGAAGTTAAGTGCAATTTGCCAATAACAAAAGACAAAAAGTGGGATATGCTGTGATAGCCGGACGGGCGGACCACTGAATTTAGTTGTTAATTACTGGAGAAATT

>Cs1g23640

AATCACTTTCAAATTGAATTCAACAAACACTTTTTAAGTTTTAGATTGTCAAATAATTGATTATTTAACAATCTAAAGTAATCCCAAAACATACATGTAGTTGATTTTTGATATTTGCTAGCACAAACAAAGTGAAAATTGTAATTTTAATGAATATTTTATACAACTATTAGGATGATATATGACATTTTCTATTATTAATTTTGCCCATAATTAAAATGTGTTATTAATTTYATTCAATGACTAAGGATGTATACATAATTTTACTTGATTCATATATCCAACATATTTAAGTATTACGGCTGATATTCTAATCAAACAATTATTTCCCCTAATATAATCTCCATCCCCATTGACGAATATAGAATATAAATGATATTTGTAATTTGTCCTCCTCCATACGTAAAAAGAGATGGYATTTTTAAAAAACATATTTCAATTAAGCAAACCCTAGAGATTAACTAAATTTAAAAACAATAATTGCAATTAATTTCTTTCTATAAAATAAGGTCTAAAATTGTTCTATTGATGGCAGTTCTATCCTAATTGCTGAACGTTCCAGACATTTAAACCACCTGAACTGCAAAGTTAACTAACCGGGGAAGGTCATATCTATCTTGTCATTTATAACTAACGCCCACGCGGCAACACTACTGTGTGTAATTAACTAGCCTSTACGTAAAATAAAATCTCTCTCCGTCGTGAATGGTAATCAAACTTTGATAGATATGAACAATATGTGGCGACACGTTTGCGCAAACGTAAGACCAATCAATGYCCAGTTCTCGCATAGCGGCCGTATGAGCAGCATTACATTacatgtaaacaaacaggcctaTTATATtatatattttactttTGACCCTTCAAAATAAATAACCATCGCTCTCCAATCTCTCCCCCTCACACGTGTTAAACGTGTGATTTCCAACATCACCGGTTAGAGTCAACGTCAGTTGCCTACTCTGATCAATAAATTTTCATATGATCGGACGGTTGTG

>Cs1g24730

GCAGCTAGTGGGTTGGACAAAAATCAAAGAGATACGAGATCTAATGAGAGACCAAAAAAAAAAAAAAAAAAGKAGATGGAGMACCTTCATTTGGGAAGATGAATTGAGACAAATATAGTCACTATGCAGYGCAAGATTTTTTTTTTATTTTTTTAATatgtgtgtatgttTTGTAgaaggaaaaaaaaaggttacTCAAAATCTGAAAGATTTAACTRAAGTACTTGGAAAAAATACAACTGATTTTTTTGATTGAGAGATAAAGCTGCTAGTGTATTCTAGCTTTATTGATAATTTTTTCcccttaaaaaaatatataAAAAGTAGAAGAAAGGAGATGGatttgagggaaggatggttGCGTAGGAGGACTCCAGCGTCCGTGTTCGGCTGGAAGTGTTAATTGTGGACCATTGAAAGACCCGAGCCAAGACAACAGCAGCAATTGAAGCRTTCCATCTAAYATTTTTCTAGTTGTTAMTATAATGCCCCTGGTCAGCGATCRTATTGACGARTCAAACMCSCTTCAAAATgaatyatataatATATGAAGACCACTGTCGGCTGCGGAGGCTTTCTGGAATAGTATTAGGTAAAATTTGCCCCTTATTGTTGATGCACATGTCCYGCTTTGGTGCTGAGCATCCGTGGCATGAAAaagagtatatatatatatatatatatatatgacactTCGAATATTTAAAAAAAAAAGGGGTTAAAATTTGGATTACGGAAAAYTTTAAAAATATAATTGTATTTAATGTTTTATAGCATTATTTACASTGYAGATGAATCTTGTAGCACGTACTTTTTTATAAACACTAATTCATGCAAAGCTTGATTAATTTCTSAGTTAATAAGAGAGAACCCAGATRCCAAGATCCTGAAAAACAGCCTCCCAAAGCATCCTTTAATTAGCTTTACTGATTTCTGTTAACGCGTGCCGGACACTCACCAATAAGATCACAGGTTTCCTCTTAATCTGACTAAGAACAAGC

>Cs1g05560

GGACTAAAATTTGGTCCCGTAGATGTAACCTTTTTGCCTCATCTGTTCTGTAATCAAAGTTTTGGTAGTGAAGTGGATTCTTTCTCACACGAYTGTTCAAAAAGTAAGACAAATGATTAGGTAGGAATGCTTTATCCTGCTCATGATTTATGTAAATTTTTCTGTTCCTCATAGTAAGGATATACCATTGTGGTTAAATGACACTGAGTTGCTCAGTAATTTAGTGAAATGATGAAAAATCTTCCCCGGTTTTCTCAAGTGTCATGTAGCTCTCTAATGGATGGATTGTTTRCCAGTCTTGAGTTCATGGATTTTGCCAAGTAAATGAAGATGCATCAGAAACGAGATGCCAATATTTTAGAGCAATCCCATATGAAAACCAGCTGATTGGCTAGTACTTGACCCCAAAATCATTTAAGTCTGCAAAAGCAAAGAACACTAAAGCTGTTCAATTTTATTGAATGATCAAGAAATATGGAATCTGATGAAATAAATTGAATTTTCTTCACATATAATGATGATTTAAAAAGAATAATAATAATAATAATaatawtmaagaactTGTAAGAGACAAGAAAGGTAGTAGTTGATTTGGGGGAATCTTCTAGCCCTTCAACCTACCTCAGCTATCCTATGTTGAGGAGGTAAATGACTGCATGAACTTGAGAGGTGAGAGAGGCACGTTTTAAGGAAGAATGTATGGCCACATCAATTTGGTCAATTTTRTAGAAAACAAACTCTCACATCAATCTGTAGCATCTTTTATTTTCAGGATAAATAAACAAATGCAGAGAAACTCTTTCGGGGGAAAACAGTACAAAAGAAGAAGTGTTTTTCAATGCAATCAATGACAACTCTTCAAAAACTTAGCCTGAGAGTCAAGTTACTTAAACCTTAGAAGATTTCAAAACATAAAACAATTGATACTCTTAGATCACGCGGWAAGTCAGCATTACAAATACGCATTAACAAATGCCATATTCACCATCAGAAGTTCAGCA

>Cs1g14520

gaaccgaaatgcattggaactttctaagaatttagagaaggctaattctcagatctctaacctcgaagatgagaaggcagtgctttacaagagcctaacggaacagaagagcatagccaatgaatcccgagaaaacatggaagatgctcataatctagtcatgaggcttggccaggaaaggaagagtttggacaagagatcaaagaaacttgaagaggaattggcatccgcaaaaggtgaaatactgcggctaagaagtcaaataaattcatcaaaaactcttgtaaatgatgagaatcctcggaaagttgaagatgacaacaaggtcccagtcactgcaaagaaaactactaggaggagaaagagtaactcacaatgagactgcaaactaattttttgttctccatattcctaatgttcatttattatccaatgccatttggcactgagactttgcattctcagattaatttttcgcgacgcatatgtatgattattgtataaatgccgaggcatgagattagggaaatggctctttgtatgtaaggatatagtcttttcatttcattgtcttcacttttcatgaatgtataatgtcaagttgaggtgcctactcagtgtatttactcttttattcttccttttttctggggtgaagtatagattgctggtttgctgcaaacattgttggcatagtttattttcaacattgtgaagtgtcatgctttcacatacatataaaatacatatgctaattgtccaacacaaaaggggaattacactcaagcattttatgttgcgctattaccaaaattgatataaaaagttttcgacaaatcctttaatttgtaagattaataagtttatcatctataaatgtaattttttaatactgccattaaaaaaaaaaaacaaaaaaaaaaacgataaaccctttggctgtactcgaacccgcaatcttgagaaaagaaaatcaatcatgctaaaccccttggccgcga

>Cs1g22370

aacgcgcaaaactcatccatgacaactacccctttgtcatctcgtaaatctaggagcgatccagtacccatgccagttaacggtatggaaatttctcaccattcgatgaggatttcagttccccgtcattattcttccgaaggtgatgtctttcttcctttttcttctgtacagtgttTTTTgatagtatttgtttTCTTGCTTTGCGGAGAAACCTTCTTTTTGATTCAGAGCCGCCTCCCCCTGGTGTAACACTTGAAACTAATGGGTATGAGACACATCTCAATGTTACCAATTATGATTACAGTCGAAGGGTTTTTGGGGCAACATTGGatagtattagtcgctctagatctggccactataacagtagtaatcGACATTACTGATGCTTATCATGTCACTTAGTCAAAAAGAACTCTTTTTCATGTAAAGTAAAAGTGTGGTACATTGTTATGGTTAAGGTAAGATAAGCTTNTTTCAGTTCCTAACTAATTTCCGTAGGTGTGTCAACCCATGGCTGGACTTGAGAAAGCTGTACTTTATTTTTTATCATAGAAAATTGTTTGTTGTTTTAATTGTAAAAGTAGCATCTGTCATTATTCTGGACTGTGAAACCTGATAGGAGGCAGTTATTTATTTTTAACACTTGTCCTGTAATTAATTGAAATGTCTTTTTGACTGTTGATATAGTTAAATTAATTTTATCATGTTGCTGCggtattggtttgtagacttattattattattatttgggaatgaacaggaaaactgatgatttatgtatttgtgtttattatatatatgtttatttgtgtgctttcatacatgaataaccccaatcttaatttgtaaacttctcctaacttcttctttataaatagtaatatatCtATAGAAGTTTCTTCGTTSCTAAAGTAGTTGATAtctggttgagattgagattgagatctgactTACWTGATAATGTGGAAGATTATAAACATatagtaaacatgcaA

>Cs1g06460

aaaatataacatattaagattaattgttcaaataaagttacaataatatattaatataacaccgaaattaattgtacaaataaagccacaattaattgaaatggtgaagtttctaaaaccaaacacagtatgatccttcaccagggatattgcacttgtcgaaaagtggtgattacgaagtcgtcaacgatgagaactaagagaagtacgagtggcgattttaaaggttgtgatagtgcaattgtttggttggaaccaatgattgtgaagtggctcgatggacttgggaaagtagatggcttgcacttnnnnnnnnnnnnnnnnnnnnnnnnnnnnnnnnnnnnnnnnnnnnnnnnnnnnnnnnnnnnnnnnnnnnnnnnnnnnnnnnnnnnnnnnnnnnnnnnnnnnnnnnnnnnnnnnnnnnnnnnnnnnnnnnnnnnnnnnnnnnnnnnnnnnnnnnnnnnnnnnnnnnnnnnnnnnnnnnnnnnnnnnnnnnnnnnnnnnnnnnnnnnnnnnnnnnnnnnnnnnnnnnnnnnnnnnnnnnnnnnnnnnnnnnnnnnnnnnnnnnnnnnnnnnnnnnnnnnnnnnnnnnnnnnnnnnnnnnttgggtaatggctaagatatgttataatattttatgtaactatgcaatgtcacatttataatattctttaattaaatttataataattttttaactaaataaataaatatatatatatatatataatttttttttttgcggattcacgaatttttaaggatttacagaagtaaatccatatccaatccatcaactgcggatttttaaattttcaatgcatatccaatccacagatttnnnnnnnnnnnnnnnnnnnnnnnnnnnnnnnnnnnnnnnnnnnnnnnnnnnnnnnnnnnnnnnnnnnnnnnnnnnnnnnnnnnnnnnnnnnnnnnnnnnnnnnnnnnnnnnnaactaggtGATTTTACAATAATTTTATGTagtttttctttctAGATTC

>Cs1g14710

TCAATCCAATAGTTTATTCAAAGTTTTTCTTTAAaaaaaaaaaataagggcaTCCAAGACATCATTTAATAATAAATCTATGGATGAAAAATTAAATTGTCATTTGAAGCTGAAAATTTAAGGAACGCACTCAACTTATACGCGTAATTTTAGTAATAAATACATCTTGATTAATAAATTTYATTTAAATTKTTTAAATGCTACATATTGCACTTAATTTATCAGTTTTAATTCTTTAAATAGTATAATAGATATSTTTTTYTCCCAAATTTCATCTCAAAATGATGTGTTATTRATTAAGTGGTTYATAATTCTWTACAAAATTRWAYTAAATTAATTGTATTATCRCACCAACTAATTGATGAAGACCACATTATTTAATATATTTTTTTGAAAAAAAATGYGAATGTGTAASACTARTTATTAATAAAATACSTTCAAGTGGTACTTAACACGAGGTTACAAACATTTTAGAAACCTAAAATCAATAGAGATTCCGGTATTAGTAATTAAACAGCCATAAACTGGTAAAATCACTTAGATTGTTTTCTTAATCAAATAAATYYTTGAGATTTGAATGAACGAGTATCTGCTAATCAGAGAAGGTAAAGTATGAGAATAACACAAGGCCAAAGTAGAACCAATATATAATTTCCATGAAAACGAGATGCCTMCTTTGCTTTTTGTAGAAACAAATTACATGCTGTTGTGCCAGGCCAATTGAATAAAAAARTYAAAGGCCCGACARGATGCAGTAGGCCTGGCCCATTTACAAACTCAAGGGGCCACAAACATTAACACCATGACTCCATATTTAGAACATCATAAATTGAAATtaaatttacttATRGCAATTGTATTTTTAGCCTCAAAATAGAGTCCACCAATAAATAATCACCGACTCACACTCAAGccctatttgatAATATGTTCTTGTTCTATAAATTGAACCTCTCCCAAAATRAAAAATCACWAGAAATTTATCTAATTAGACAGCAGCA

>Cs1g09270

TATTTGATGACAAGGGTATTTTGGAAATTTTAGCAGTCCACTAACAGTCAACGTTACAAAATACTAACGCCAAGGGAGGTTTGTGTAATTACGRTCATACATTCTCTCAGACTGTTAATACCCCAAACCTCARRGGAGGTCCCTAAAAATTATCCCAATAAAAATTGAACATCGCAAAATCCATCAAGATTCAAGATAATTTTTGTGGATGGGAAAGCCGAAAGCCCGTTTGTGATAAACGCTTTTATCTAATCTTTGTCAAcacaagaaaaatTGAAAACTATTGATTAGTCGAGCCGCTTTTATCTATCTACCTCAGAGCATCTCTAAAAAGTTCTTTAAATTTTACTCTTCAAATTTTAATTTGCTTACTGATRTGGCAAATAAGTGTATAGAAAAATATAATYCCCTCCAAAACACTCACCAAATAAGAATAAATTAATATTATTTTAATCAAATCTTTCCAACTATCAAATTGAATTGTTATTATATTTTTTAAAAAACATAAATAATAATTATTGTAGTCTTATCTTTTCAATAAATAATAATAAAATATTAATARAAGAGGGAGAAACTCACTTGTCAATATTGAAGASTGGGAAACAAATCTTATTTGAAGAATCTAAATTAACGTATCTTTTGGAGTGTTTAATATTGAAATGGCTCTTCAAATAAGATTTTACTTCTTATTTGAATAGCGTTTTGGAGATACTCTCGGTCATCATTAGGAAAAAGAAAAAGGCCAAAAAGaagagttcaatgtTCCATTACATAAAGGTTCTGTACATCGTTTACTCWGTTGTGATGTAGTCAAAATTCTTGGCCATTTCTGACCAAGCCGCGTAATTATTGCCCATACACCAATCGCCAATCCTTTCTTACGGGGGGCAACTTGTATAAAATTATTGACTCCCATTGTCCTACTCACCATCCGCATTCACATCATTTTCCCTTTACATATTATATGTAAACAACTCATTATTCAATACTTCGTTGTTTCC

>Cs1g25950

ACAGCCAATAATGGACTTGGATATGTCAGGATGTGCGCSGTTTACTTTCCAAGCCTACAACCTCCCAACGCCTTCTCTTACAAATTCATTTATGTCCCCCGAGCTCTTGTTTTGTTACATTTTGCTTAATACTTTTGTTGTTTGTTATTCCGGAttttttCCCCCtatttaagaatcaaagannnnnnnnnnnnnnnnntcctagcttttaaaagactaGTAGAAGTCTAGAACTTAGGATATGCTATTACCATTTAAGTTAGTCTTCGCCTCAATATTATTCCGAAATATATttagtgtatatatatatctattCTATCATAAATAAAATAAAAAATTCAAGTTTATTTATTGTATAGTCATTAATCTCATCTCTTTACATAGTATTATTTAAACATATATATGACTTGATCGATTTAGTAATAAACTAATTTTATATAAGATATTATTTTTTTATTAAAGTAACTAGATAAATTTGTTAATCATAAAACTAATTATGTATTAAAAGATTACTTTTTATATTAAAGTATTGAAATAAATTTGTTAATTAAAATAAGATATTTCTTTAAGATTCTAAAGCTACAAACTAAGACATTACGCTAACTTGCAGgctaaacaaaaaaaaagttgtTAATTAAATTCATTGACTTTTCTAGTATCAACAACTAAAATTATTATATTATTACTACAATCACAAAGACTTTTGAAAAAATGAATAACGAAGATCATATGATCATTTTATTCAACTAATCATGTAGTGCTCAAATGAATAAAGTCATTGACAAATTCACACTATTAATTAGAGATtgaatttttttTTTACTATTTATAAAAAAAAAAGTATTGAGGATGGAAAGAAGTGTTGGTAAAATTATTTTAGTACTCTTTAAAAGTATTTATTTGTTATTTTATATATGAAAATATTCACATTACGAAGAGAAAAACTACAATTTAAAAAAAgAAGGAAAAaTgggtaaaattatannnnnagaagggccaAC

>Cs1g17890

aaataactAAGATGTTAAATTTTTAATTAAAAATTTTATCTTTGAGCTACTAAAAATCTTCAATAGAATTGTTATCGAGAGGGTTAAAAGTTATTTTAACAAGTTAAACTTGATAATTTTTTTAAATTCACTTTTAAAAAACCATCTTATCCTAAAATAGATTATGACGATATTTTTATATAtTTAAATAAACTAAaaaattatatttatttaattaaaAAATAAAATCATTAAATTTAAAGAGACTTAGGATACATGCTTCTGGGATTTTTTAaaacttaccatttttaacattaaaattttggatcannnnnnnnnnnnnnnnnnnnnnnnnnnnnnnnnnnnnnnnnnnnnnnnnnnnnnnnnnnnnnnnnnnnnnnnnnnnnnnnnnnnnnnnnnnnnnnnnnnnnnnnnnnnnnnnnnnnnnnnnnnnnnnnttcaccctaaaatttaatacttataacaacaattatttctaaaacTATAACATTACTAAATTACTCTTGAAACCCTATTTCCATAAACGTATAAGAATTAATGAATGAAATCAATAAATGAAATGACTTTCACTTTTTAWTCAATCAAAACGCACTGCACAAGTACACAGCCATTTATATAGTACTTGTCTGATTTATGTCACCTGGTGATTATTCTCACAGCCTGTCATTCACGGCTGTTTCACCCATTTAGGGTAAGAAAGATCCCTCTTTATAACTTTTTTTTAACCGTTCTGTATGACTTTGAGAAAGAyagagattgtgatttgtgatttgtgacgcacACAAAACACAACATCAGTTGTAAGAATTGTACAGTGGCTAGCTGTATGTTTGTACAGCAGCAAATGCCATTATTGGACTGCCTGTGACTTGTGACATACCAGACAAGAAGACACGTGTTCCTCTTGTCTCATGAAAGACCAATGAAGTAGTTGTCTTCATGTAAGCAGCTATACTTGAACTGGCCCCGACCTATAGCAATTTTACTTCTTTATTtattta

>Cs1g26620

taCTGAACGGGTAGTGGTGGCAAGAgctgcacatcgatgaaagGGCTATGCTSTCTACAGTTGTGCACTTGGCTGAAATGGAAAAATTTGGGGATCAGGCTAAATTGAACTGAATCCGAGACTCTTGAAACTCATTTTATTTATATTTTTTCATTTTGAGTTTGGTTCAGTTCGGTTTAATCTTAAAATTCGAACGGATTCAAAATTAGAAATAGACCAGTTtttttttttttttttttttcagttTGAGTAAACTCGACTGGGTTTGCCCGTTAAAACAAAGGgaaATAATAATAATAATAATAATAAGAACCCAAGCaatccttaatacacccggtcctaaccttaagctgtcnnnnnnnnnnnnnnnnnnnnnnnnnnnnnnnnnnnnnnnnnnnntaatttgctTCTTCGTTCCATGCTTCCAACTGCTGTTGCTTCCTCTCTGCCTCCTTCTTCTTTCGCCGTTGTCAACTGCACAAGGTCAGGTTATACGTASGTGGTGCATGAATGGGACANTTGTATTGTAATTTGTATAAAGTTTGTTTGTATTTTGTAATTCaattgaggcaataaaCAATAGCTGATAtTTATATTGTAATTTGTATGATGGTTGtttgtatttttaaaaaAGACTTGTTCAGGAGGATGATGCTTGATAGTTGATTAATAGTATTTGTTTTTGACACAGTTAGTTGATTGGGATGTGATTTGAGATTTTAGAATTTtaggttactacctagtgatTTTTGGAATTGAGTTAGTTGTAATTCGAATTAAAATTAAACTAGAATTTAGACCCGAATTTTRAAATCGAACTAATTCAAAATACGAACATAAACCACACCgaaccgaataattgattGAAACTTAAAAAGGAAGAGTAAAATTTCCTTTTAAAATGTGGTTTGYACTTTCAATTGAAGAGCTAGCAAAGCGGGAAAGAGCCTCCTCATTCGTCAACCTTTCCAGTTACCAAATCGAGCAGTCGCAGTCGCTCA

>Cs1g11580

actttgtgggaagttggtgtccaaatttatgatgcatataaacaagaattatttactctacgagctgtcttgttgtggacaattagtgattttcctgcttatgggaacttgtctggatgctcagttaaaggatattttgcatgtcctatatgtggggaagatacacagtcctgtagacttaagaatgggaagaaannnnnnnnnnnnnnnnnnnnnnnnnnnnnnnnnnnnnnnnnnnnnnnnnnnnnnnnaacaaaggcttttgatggtaaaccagaaagagactttccttctaagccattaagtggtgaagaaacattaaaaaaggttgaagggattcaaaactcatgggggaagaaaactagnnnnnnnnnnnnnnnnnnnnnnnnnnnnnnnatttgctggaaaaagaaatcaatatttttttctctcgagtattggaaacatttgcatgttcgccatatgttagatgtaatgcacattgaaaaaaatgtgtgcgaaagtatatatggtacattattgaacattccaggaaaagcaaaagatggacttaattctcggttggaccttgttgatttcaatattaggaaagaattagaacctgttgttgaagtgaatcacacttatttacctgctacatgttattccttgactagagtggaaaaagtaatgttttgcgaaacattatttaatctgaaggttcctgaaggatactgttcaaactttaaaaatcttgtatcaatgagtgatttaaagcttattgggttgaaatctcatgattgtcatgctttgatgcaacaactcctaccattggctattcgaggaattttgccagaacttgttagatatgccatcactagactttgctttttcttcaatgatttttgtagcaaagttgtggatgtagagaagttaaaccaaatacaaaaggatcttgtggtcacaattaatttatttgaaatgtatttccttcctgaattttttgatatcatggttcattt

>Cs1g23720

TTAAATTTTTTTGTGTGCTCATGAAATTTGTATTTCACTAAAAAGCTAATAAATTGTARGAGYCATTTAACAATAGMTGAAAAATGAGAGAATAAATCATATTAATTATAGAGAGAAAATAAGAGGAAAGAGAAGAAGAAATACATAATAACATGTCATGGAGCTATTAATTTTAATTTTTTATCAATAAAAATAAAAATCAGTATTTAATTTTGTCTACTGATTTGAGTGGTATTAAAAGGTTAAGATTTTATATATATTATTGTCTTCAAAATACGGCTCCCAATATTGATATATAATGTAAATGTTGTGTTATGTTACTATAGGAAACATGAGAGATACAATACCCATTAATATATGTATGACATATGCAATTAAATTTTAAATAATCATAATTAAATGTTGATTTTTCAAAGTAAACGCTGGCAAGTCATAAAATTATATTATTTGATTATTCTAGAATGTCTCGCAAAGTGGATTCTATGCAGTGCGAATCGTGTTGTGTGGGGTGGAAAATATGAATGTCAAGTCAACATCCCTTGAATCTTCCCatgagcagatgAGATAGCACAATTACGTCAGTATTCTTGACTAACGGTTCGACTTTGATCTTTCGGTCGTCTGCGAACTTCATTTTTTTTTTGTAGCAACTCTGCATCAATTATCAAACTGTCTACTWGACTGATTCAGactagttttttttttcctgaaCTGGGAGATGGTCTTGAATGGACCTCAAtTGTGAAAGATATCTTtaagcctataccgcaattcagactaatttttggtgatatcgggtcggtccgtaaggggtaaagtactaattaaagtggtgactcaatacgnnnnnnnnnnnnnnnnnnnnnnnnnnnnnnnnnnaatgagagtgccgaatcactcataccaacaaacTTTGGTTATCCAAATTAGTTATTAACTATTGGAACTTAGTTATGGTCTCCATCCCAACATTAAATTGAGTCAATATTGGTTTACAGAAA

>Cs1g21000

CTAATTTGGCTAGACTGTAATTTACTGTAACATGACCCTTAAAAAGGTAATAAGATACAAATACGATGTCATGATACAAATAAAATGTCATACTTCATTTTTCTGTCCAAAGTAATGATAAGATTAATTAATAAAACTTTGTCGTGCGTATACACACGCATATATACAACTAATGTGTTATAAAATTTTATTTTCCTAGACAATAATTGAGGACGGAACTCAGCATATATATGTCGAGAGAAACATATATATCTATGTATTCACATCTAAGAAAAATATTCTGTACAAGAATTTTTTAGTCACAAAAATGGGATTGCATTATTCGTAGACTTCTTTTGGGTCCAAGGAGAATGAAGGccctttaccaacaaAGGACAGCATTTTCACACGTGTGATAACAACATTAATTAAgtattaaacgcggaCGGTACCCACTATATAAAACATTGAAAATCATTCAATCCTGTCCAATTGGAATTGCACAGCTTCATCACTTCCTTTGCACAGAGGTAAAGCTTCCACTGACTTTTTACTCTTTCTCTTCTTTGATGAAATTCTGAATCCTGTGTATGATTGATTTGGGTACAGGTGTTGTACTCAAGTTAATTAGCATTAGAGCATATATATATGCTGCTTTTGTCCTTTAAATTTGAAGYTTCTTTCTTCGAGTTATAGTGAGAGATATCTTGGMATTAGTCTGAGAGAAAATTTGGTAATAACTATTTTAATTTCCCGGTGCCTCTGTCTAATATTTCAGGTTTAGTTCTAAAGTCCACCTCTTGCTTCAAATACACAACCAAAAGGGGGAAAAAAAAAGGCTCAGAACTATGGAATTGTCATCAGCTAAATGGGTTTCTGAAATGGTAAAATAAGactattatatatatgaaagtTATTTACGCACTATATGAATTAAATGCGACGAAGAGTATGGGTTATTTATTATCATCAAAATTACTAATTTCTCTGATAAATTCTTTTAATTTTTGATTTATAGGAAA

>Cs1g17810

AGTATCTATAATGCATCAGATTACTGGGAAATCTGTCTAACATATTACAACTGATTTCACTTCTATGCAAATCTGCCTttctatctccttcttttcttTTWTACTTGATCCAATTGTTATCTGTCCTGTAATCACCCTTAGATTTCTTTATTCCCCTTCTGATTGTATTGTTGTCCCACGTACGGTATCATATCAGTTATGTATGTAAATTGATCCTTGCAGCTGTACTTGTCTCTACCATTGATTTAATATATTCATTAGCATGTACTGTTGCCAATCCTTTTCRAAAATAAGATATAGAAATTATATTTATCTATTTATTTATTTTTCGGAAAACAGTTATGTTGCCTTTTAGCTGTAATATTGTATTTCTTTAGGGTGCACACTTTGTATAATCTTTTGGATATTTTGGCGCATTTTTGCTGCTTTATTTAATTTTTTGTTTATCTTTTTAATGATACCCTTTTGCTGCGACAGGAGGAAGATAACAGTGTGCTTTATGCAAAGCTTTCAAGTATGGAAAATTATGGTTGGGGTGATATTGGAACAAATGCTGACAGTCCATGCAGTGATGCAATGCAGTGTGACAATATCTTTGGCATTGATCCATTTTACATAGAAAAAGGTGATCTGACAATTTTTTTAATAAGCATTAATATTTTTAAGTGTTAGCTTGTTATGGAGGCACAAGGTCTAGAAACTTGGAAATAATTTGACCAGATGGACCTCTGTTCCATCTCTCTATTAtgtgtatgtgtgtgtgtgtgtacacaAAATATATTCAATATTACTATTTTTATTCCCAAAATAAAAAATGAGATGGTGGCAATCAGTACTTTGGACAAAAGTGAAAATATGATAGTCTTGTATAGTTGCAATCTTTAACTTGTTTATTTCAACTGGATGTGATGGATGTATCATACTAATACTTATCAGGCAAGGGCTTCCASTCTTGTAATCATGWTTTCTAGTAACATATGGATTATGTCTATAGGTGATGA

>Cs1g26730

nnnnnnnnnnnnnnnnnnnnnnnnnnnnnnnnnnnnnnnnnnnnnnnnnnnnnnnnnnnnnnnnnnnnnnnnnnnnnnnnnnnnnnnnnnnnnnnnnnnnnnnnnnnnnnnnnnnnnnnnnnnnnnnnnnnnnaaacaaaaaataaATTATTTAAATAATTTcaattaaaaaagagttcTATGACAAATTTTAAGAAGTGTCCAAAAACCTACCCTATATAATAATGAAGATAGAGAAATCGGCTTTGGTGACTTCTTGATCCATACTCTCCACAATGTGAACTATTGGATGTTCAAACACTTTCTAAGACGTTTTTACCAATTGAGCAACTAACAATGAATGAAGTTGCACTTTTCAAGTAATACAATATGTAATAATAAATAATTTTAATTGAGTGACATTTTATTGAATGGTGAGATAAAATAATAAAATAGTTTAATAAATTTTGAATTGAATCGTCAAATGATTAATTTTCGGTGTATTTACGATTATTTTTCTAAAATCAGAATGGATAAGAATTTGAATGGACTGAAATCAAAATAGATTAAAATCAGAATCAAAATAAGTAGTTTACTTTAGGTATatgaAtcaaaatcaaaatgagctgtattctcattgATATATTTACTTTATCTTGTAATCAAAATAAATTATTACAAGTTAGTAAAATATCCTTAGTTATGATTATATATTTGTTTAGTGAGAAAATAAAGAGTAAATATTTTTCTGTGATGATGACGATGTTTATtattattattataaaattaattattacatgtnnnntacagcagctttaTGGGAATATAATTTTTACTATTATTTTTATATTTTTAAAACATAAATATTATTAATAAAAAATAAAAATTATTCACTAATTGAAGAAGTAAACACACCCCCAAATGTTAAATGAGCGCGTAAATTATTATTTTGGTAGCCTACACCTTCTGAAAAAGGAAATAACaataaaaaaaaaTATTATATACAGAGCCAGaggtgtggg

>Cs1g05430

TTACATCATTCTTTTCTTTCCCTTTtctgtacaaacaaacmcttctgGGATTAAATCTTGTTTACATGATTACAAAAGCTGTCAATCTCGCAGCAGTAGTACTCACCTTGTATTGGAGGCCTGAGCCTAAATAAGTAAACAGCCTGATTTTGATGGCGATGAAATGCAGGCATTCTTATATGACCTTAGTTAGACATAATAAGCTCCAATGCATTTCGTATTACTTCATTTTGACAGGAGCATTAGGTATTGTTACCTCKGTTTTACAGATGCATTTGTTTTCTTTTCAGATCATTCTTGGTCTCCAAGTAGCGCATGCATGGAGACCACCAATACAGGGCTAATTCATGGCTTCTGATTCCCATATCAGTTGTAAAACAATGCTTGTTTGYGCATCACTCCAATCAGTGCTATCAATTCTAAATGTTGGATGCTATAACTGATATGTAGCATAAAAACTtgcaaatttttTTWTTTTAATTTTATATCTTAAATATCACGTACTTATAAAataaatttaaaaaaTATTTTTTTTTTCTAAGAAAATTGTCTCTGCACTACTTCAATTCTGTTGATGTAACTATTAAAGAATTATAACAATGTCTATGCAGCATCTAACTTTTTTTTTTCTATTATTTAGCCACTTCGTCATTACAGAAAATTTAATTTTAAAGAATTTATGGTGAAAGGCTAATTTGCCACTGGAATTTTATGGATATGATTTTACCAATGAAATGTAAAAAAATTATGAAGACAGTAAATTACCAATTACCCTTGCATTAAAAAATATAATGACAAACATGCCCGCAAAAGTAACTGTTGGAATTAAATAAATAAATAAAAAGCCcgactannnnnnagttacttaaaatgtcttgttaaaaaaaaaCCGAAAGTTAAGTGCGGTGAAGATATTTTTAGAACTCTTGAGTGAGTGTATAAAATTTCAATGAAATTGAGGTGGTCCATAgataattttttttctattaaTACaaaataaa

>Cs1g26470

TAGGATCAAGGGAGAAGACTTGAAAATTGCCTTTGACAGAGCTATTTGAGCTGTCTTTCGGTTAAACAAATTAGCTATGCTTACYTCACTATTTGATCTTCATGATTGTACAACAGGTCATTTATATGTGAAGAGTGATGTGTATGGATTTGGTGTTGTGCTGCTTGAACTGTTGACGGGTTTAAGGGCACTTGACACTAAACGCCCAACTGGACAGCAAAATTTGGTTGAGTGGTTGAAGCCAATGCTGTCTCAGAAAAAGAAGCTTAAAACTATAATGGATGCACGGATAGAGGGCCAGTATTCGTCTAACGCAGCGTTGCAGGCAGCACAGCTCACCCTAAAGTGCCTAGAATCAGATCCTAAAAGGCGCCCCTCCATGAGAGAAGTTCTGGAAGTGTTGGAACAAATAGAAGCAGTGCCAATAGAAAAACCAAAGAACTCCATGTTTACCAGTTTGCATTCTGCACCTCATCGCCGTGCACAACGACCCATTCATCATCGCTCCCCACTTCATACAGGTCACCGTGGCAGCAGCGTCTGAGACGGAAACCGAATTTATGCTGACACAAAAGAGTATTGTTCCTATTTCTTAGAATGGAATTTGTTCTACCTACCATTATTCTTTCTTTAGTTGAATTAATCATGACAAGTTTTGAATTTTAGTCCTGGTTTGTGCTGAATGATGAGATGATGAAGTTGTAAAACTAGAGATTTCCCATTGTCTTGTCTGATGTAAATGTGACTGGACACTGGAGTGAAGTTGAATGGTCACGGCCAAGAAAATCTAATTAATCTAGAGTTAAAYGCAGAAGAACAAGGCAGCGCATTGTTGCTAATTtgctgcaaaattgwgAATATGAATCACATACATATTAACATACGTACGTAGACTACTGTATCGYATATGAGATGTCTGGTCAATAATGACGTGAAGAAAGTCTTGAAATGGGCCTGCTTAGAAGAGTTGGACTTTGGAGCCCATACCATCATSAAGCGCT

>Cs1g04370

CTACATATATCTGTTAATGGTTTAATTTCTTCYAGTTTTAGAGTTCTCACAATCAAGTGCTTGGAAACAACGAGAGGTTGCATGAGTGGGGTCTGTGGGGATTGGGGAGATGGAAGTGGATTATCGCCAGGATCTTCTTTAAAGGGTACTTTAATCACCTTTTAACATTCACATCAAATGTTTGTCATGTACGCTATCTATTAGTAAGTTATTTGTAAAATATTATTGGTACTTGTCTGTTAGTTTATTCGAGCCATCGTAAAGGGCGTTGAAAAGGACAAGGCAATTTACAAATCACATATTAGACGGTTCTGCTTGAATTGCACGTCCATAAGAGGAAGAAAAATGGAAAACGAAACCAATATTATATTCACTAAATGGGAGTAATATTTATTGGAATTTATTTTGTTGCGWGGATGGCAACAAATGTAATCATCGTTGATCTATGTTGCACAAAAATTTTCTATAATACATAATCTTATAtGGagaagagtttatgaaaatttgtattagaattTGATTAGTTAGTTAAATAAAGTTATTAGATAATCATTTGGTCGGGCTTTTAGGAAAATTTGTTGTTGATAAAAGATTATTAAAAAATTACTATGTGAGAGACGTTTTAAGTCAAAGCATTTTTCATAATTATTAGATTTAAAGTTGAAATGATTATTGGTTCTTTCAACTTTAGATCTAATGATTGTAATAGATGCTTTAATTTAGAGCAGAACTAACGTAGCTAGACCCAAAATTGTAYGTGTGAGGTTCCAATTTTACACACGGAAGGACTTTATGTTTAYGAAGATATATTACAAAAAGGTGCACTTTTTCCAAACTAAATGTGGATGTATGTAATTTAACTAAACGTTAAGTAGCTAMATGAAAATTTACCCACAAAGGCCCAAAACAACACTCACAAAGCCCGATTAAATTGTCCGGCCCATGATTCTCCATTCAGTTATAAAATGGCGCACGCAAACGCGCCCTTATCCGTTTCGTAT

>Cs1g06130

TAAGAAGATGAAAAAGAAAAAGAAAAATAAAGGGGGRAGCAGTAGTYCRCGTCCGAGGCTTTTGGGTATTTTGAAGGCAGTTTTATTCGAAACTTCTTTGGTACGTATATAATATATAATAATAATAATTAGTTAATTTCCCTTTTGTTTATTTATCTTGCTCTGAATCAATAACAATAATCAGGGTTAAATTAAATTTGTAATCATTGCAGGCGAAGAGAATCAGAAAGAGGAAAATTAAACAGAAGASGTGCCAAACAAGCAGTGGATCATCAAATGAGGAATCGTCGATGCTCAAAGAATTTGATCAAGATGGAAGCATTACTTCATCTGCTTCGGCGTGTACTTCCTCGTCTGATCATACGAACAAATCCCTTCGATTGGAGTGTAAACAAAAACAACAACGGGACGTTGTTGTTGCTGAAGCTCAACATCATCATCATCTTAATCAGCATGGGAGAGGGTGTTACGATTCTTACGTTGGCTTCTGTTTGATTTTAATTAGCCTATTGATATTGATTGTATGGGGCAAGCTCTGCGCCATATTTTGTACTTCCACGTGGCTTTTCTTGGGGGCYCGTTGGAACTCCGACAACCAATCATCGGAAAATATGCCTGTCGATTCCAAATCGTCATGGGAGGGCAGGTAGAAAGGGACCCATCTCGCGGTCTATATAGACCCATTTCGACATTTTGCTTTAATTTGCCagtttggtacctttttttcagatttttggttcatgatatgtatagctttatatgttatacttatatgtaagTAAATTAAAAAGGATTTAGGGTTCCTGTTTTGACTTTATCTTTttgcaattttttaagtaCGTATGTTTATATATGATATTGAGGAAATGAAAATTGTACAAACATTTTGGTGGTGGCACGAAAATTATTTGAATATTCAAAGAAGCAAAATGATGCTGCTGGTTAACAAACGCTGGCACTTGTTGTTTTCAGAACTTCGACTGTTTTGGTGGGATGATCAT

>Cs1g06230

GGCCTAAGATGTGGAATACAAGTACCGTAGTCCATGGATACAAATTTCATTATTATTGATTTATTAAACAATAATTGAGGGTCAAAGCCCTCCAATTGTGAATTGTGATCACATGAGAATTTGCCATTCTCACTTTGTAAAGCAGCCTCCTCTTAACTTCCACCARCCGTCGAGTGAACATTTGCCGCTCGCATCACTAAATTCAAAATACGATTGTGACGCGTCACGTTATCATTGGTCCTTTCCCATTTATTCCCGCGAGCTGCTTTAACGCTCATCAGCCCACAAATTCCCCCTTTTACATCCGGAATCAAATTATTAATAATAAACTATACTGCAAATTTATCACTCATTCCCCTTGAAATTTATGATATTCTCACAGAGATAGCATATTTCATTTAATAGTCGCTTAGCTCCCATTTAACAAAATTGAGGACMCTTCAATAACTTACAATGTGCTCAAGATAAACACGCTTCTGACATCCAAAGTTCGcttcttttttttTTTTTTTGAAAAATCAAAAGATTACGAACAAGAAATTGAATTTGACACTTCTATAATTTTCTTTGGTCTCAATTTATAATAGAAAAAGATTTatgagtgtaagattagtgttTTTTTttatcaatccaatccgtacaatcagatgCCTATTGATATtTACATATGATAGTTATTGGAACCACTTAAATTACTTTTATTATGGGATAATTCAAATTTATATCTCatatactttgaatgttttatttcacttatattttttattagaagaggaaacagtctcttactggactcaaactgctactctnnnnnttaatcccactcggacacttgacaattcgacatgcatgagaagaaaaaaaaaattattttgataattaattaaaaaaaggaagtgaataaatcccggccatagaagtttaaatctgacacgtctcatgaaaaagattagaaaacattcttcctcgctggtttattttaatacagatctaggnnnnnn

>Cs1g12760

AAAAATaaaaaATAAAAAAATKATAAAAATACAAAGAATTGAYGATTtaaaataaaaaaacaaatTGAARTGGACCAATTTAGTTTGYTTTTGTTTGATTTGTWGGGCAAAAATTGATTGGTTCAGTTTGATTCATAACCTAATTAGGAAAAGGTTTAATAGCTTttgaaattggtctAAGTGATTATGAAATTGYAGAAAACAAARCATGATTTTAATTTAGAATAATTTTGTTTGTTTATTTTTTGGAATATTATATAATTAAAAAAGAAAAGWGGAAAAATYAAACTAATACCTTTATCTGAGAATGAGGTTAAAATATTGAAAGAGGTTTATTTCATAAGTTTAAacaaaaataatgttTGGTAAAATTTTAAAAAACTGCCTTTTTCATAAATTTATCTATTTTGATCATATTTCTTAAAAGCAGCTAATTAAATAAGATACCACATTTTTTTAAAAAATTCTTATTATAGTCTTAATATTCTTTTTTTTTAAAGGACAATTATATCTTACTTTTTCAGAACCCTAATACACAAATAAAAAAGTGATTTAAACTTATTTTCATTATGGTAATTATATAATAAAATTTATCAAATATATATACTACACATTTCAAACTTATAACAATCGCAATAACATATTTWKTCAAAWACTAAATTARTTTTTAAATAAGTAATTTCTTTTATCCACACAACAAAAATACCTTATTTCAATTGCTCATCAGAATCCCAAACTGACCTTGAAACTATTTATGCTGGAGTTATTTATAGTTGGATAAAGAGGMTCYTGAAAAGTGGTGCTGGAAATCGWGGGATGGTAACGTATTCATATAACCATTCGTTACAGCTTACATGACACATTTTTTAATATTAAAAATAGACTTATGATGTCATTTTATTCTTTATCTTMTTTATSTTTGYGGCATTATTTCACGTTAAGGGTATYACTAATGAAAtgtaacaaaaaAAAAAAATTGTAAAAAAACTATTATATTTCGA

>Cs1g11550

tagccattgtttctctaatggttgggaattggcagccaattttttcccccttttccatgtattcatatgtaaatttgatcaatgtgtttgaaatgtttcttcctcaacttaacttgtatcctaacccatctactctattgaatgaagaagctcctggtgtgataccgcagttgcactacctatgtttaatttttatgtacttaatttagattattcagtaatgagagtaggatatctcgcatctaatttattaaagtatttagaatttgtaatatatactaataatatttaaatatgaaatgaaagattttgtaatataaattgattttttggttattttgtggtgccttttaattttgcgaataaatcaatacgcaatgatccaattcaatatatttttttaaataaattaaacagtgataccatggtaacagttatcatagacactaacaaaatatcagcaactactaacactatGGTGTCATAATATTGGTGATATTGTAGTGATAGTAATCACTGACACCGTATTCGCGTCACCATAAACAAGTGTCACTATTTTTCTGACTGTCGGATTACTGACACAATTAACAAGTGTCRCTAAAAGTAATTACTGACACTATTCTAATGTCAATAAAGACCGTTTTTTTAGTAGTGCTTTCCTCATGGATATGGATTTTCTTTTTATAATTTATGGGAAAGTGAGTGAAACCCATGTTTGTCTAAACTAAGCATTAACCCTCTTGTGTTTCTCTTGAAGACAAACCTTTGCGCTTTGATTTTTCAAGAGAGAGAGGGGGAAAAAAATTAMGCAAAGTTTGGGAGTATTTTGGACTtgaataaaaattgtttTCAAATaaccaaattattttTTTTctttttgTAACTTTCATTGTTATGCTTGATTGAAGaTTCTtttttaaaaataTCATTACATCTATAAAATCTATCACAAATTGATCAAAACGTGCAAACAAAAATATGTTGGGATTTATATATCTTGA

>Cs1g04270

GAAAGTTTATAGGTTCTAAGATGTTTTTCCGTTGGAAATGAAGATTTGACTCTTTCGGTGGAAATGATTTGAACATTAGaagcgacacttttgatacTTtttttttttttatgatttggacattattggtgaccaatttgggagcagttggtcggaaaatgtttttgagaaccannnnnnnnnnnnnnnnnnnnnnnnnnnnnnnnnnnnnnnnnnnnnnnnnnnnnnnnnnnnnnnnnnnnnnnnnnnnnnnnnnnnnnnnnnnnnnnnnnnnnnnnnnnnnnnnnnnnnnnnnnnnnnnnnnnnnnnnnnnnnnnnnnnnnnnnnnnnnnnnnnnnnnnnnnnnnnnnnnnnnnnnnnnnnnnnnnnnnnnnnnnnnnnnnnnnnnnnnnnnnnnnnnnnnnnnnnnnnnnnnnnnnnnnnnnnnnnnnnnnnnnnnnnnnnnnnnnnnnnnnnnnnnnnnnnnnnnnnnnnnnnnnnnnnnnnnnnnnnnnnnnnnnnnnnnnnnnnnnnnnnnnnnnnnnnnnnnnnnnnnnnnnnnnnnnnnnnnnnnnnnnnnnnnnnnnnnnnnnnnnnnnnnnnnnnnnnnnnnnnnnnnnnnnnnnnnnnnnnnnnnnnnnnnnnnnnnnnnnnnnnnnnngggaatttgtacatgggtcccgtagccagaatgaggttttttctttaatttggcaacgacatattccttcgcaggtttcttttttcttgtggcgtttattgaatggtttcttggccacggatnnnnnnnnnnnnnnnnnnnnnnnnnnnnnnnnnnnnnnnnnnnnnnnnnnnnnnnnnnnnnnnnnnnnnngacacttatttttgaattgcccagaggttcaacaggtttggcggcgattttactcaatgattggtcgtccgtatctcccttttttgtctccccatgctttgcttagtcactggcaacggtgttgctgttcttctaaacatattcgagtcattcttccatgtttcattct

>Cs1g14970

TAAAGCTTCCAATTTTATATAATTGCGATGTGTTCACACACACATGCGTTGGTCCATACCTCTTTATTATTTATGCTCTTGTATTACCTAAGTCTCACTACTGTGTTACTGTTCATatgagagtagtaagttacagctagccgtaacttaattggatccTTTAAACTAAGGTTAGTGTACcctaaaaaaataaaaatcatAAAATGAATGTGTTTTTGTGATGTAATCTTATGAGAGAGAATGGTAAGACTCTTCCCATCACGTGGAGGGAATAAATTTGAGTTTCTAACAACATAAAGTTGATAMTTTATTRGAATCAATTCGGTTTAGAGAAGTTTTAGTTGGATTCATAAAATTRTGAATcatggattttttttctaatTAAGTTWTAAAAagtaactakaataaaataaaataaattgactAATTAYASTAATCAGCGAACCTAATTTGTATTGGTTCATTTTAGTTTTTTTATTTAAATTATTTCAAAYAATTCTAAACTGAACCTAAGGTTGTTTACCCTTGTACACGCTCTTTTGTAAATATTTATTGGTTGGCATGGCATAGATCGAAAATTACTGTGAAGTAAGATGGTAATAGGAAAGGTAGGTGATTAATTTTGAACTTCAACTATTAAAAATATTAACAAATAATATAAGGGGGYTTAATGAAATTAACCAACAGTAAAAACCACACGTATTTTGTMATTTTGTCTTTAAGTTTCGGTGTTCTGGCACTTATACCCTGGAACACTCAATTCCAAGACTTCATGCTCTTKAACGCTTGGAAAGGTGTCGAAATTTCTACACTAATCAATCAATTCCCCACCGACTAACCCCACCTTCGAAGCTCTTCAGAACCTTCTCGAACTCGCCCCAATCCTCACCCTCCCTTCAATTTTATAAACCCTTTCTACAAAAAGAAASCCAGTACTCGATCAAACGATTCAATAACTACGCTTCTCGCATCAGTGAGACATTTTTACAAACGCCTTTGCA

>Cs1g20130

TAAATTTCAAATTGGAAAAGAAAAGTTAAAAAAACCCCTCAAATCTCGTGATCCGTGGGTAGGGATGCCAACGGCTTAAGTACTGTGCGAGAATGTATATGACTTGACTGTACCCGTATTTATTTAGTAGAAAATACCTTCACCATCAGTCTTTATCTGTCGCGGACTTAATGGATAAGTCCGAAATTTTTCGATAAAAATTATAAGTACACAGTTATTTTAGAGTTGTTATGAGATAGAACACGaattttaattatgtatataaagtggtaaatagtaaaAATATagaatAGTTTTAACATAGTCCGACCCAAATTATAATTACCGGTCTCGTCCCCTATCCCTAtaatccttttttattttTTAACTTCAGCATATGTTTGAAAAAAAAAAGAATAATATCTTAACTATATAGCTATAGATGCATACAACACTTTATTTTAAAATATTTAAAAAAAATTATTTTCAATCTCAGATAAATATtaattataatttaataattgttaaatttaattTTAATcaATGTATTATTAAATATTTAGATCGAAAGTTAAAGTCCCCACTTTAACATTTCTTTATTCAATTTCAACTAATCTTAATTTGATTAGAATAAATTTTTAGATGGTTTAAAAKATATTTAATCCATTAAATAACTAAAAAATCTTTAGGCTGTTGTCGTGGACATTATTATATTGGCTTTTRCCCTTCAATGATTAATGAATGCTTCCCGTGCATGCCAGATggtctgtgagtgGCACCTCGCTATTTTATTATATTTTTTATATGACAAAAGAGATACAACTCTAATTTGTTATGACCACAAAGGTTATAACTCTATTTCAAAATTACATGTGGCTCGTTATAGTTTCTTCAATAATTTTAAAGGGATCTCCTAAGTTAAATTTTATGTATTTTATATTCATTTATATAAATCGTGCATAGTGTGAAACTCACTTATTATTTATATGAATGGGTATAAGATAGGTGAGATGTAGGCTAGA

>Cs1g05910

AACTCAATTGGTTCTTGTTCATCGTGCTCTTGATTATTTTCCACATATACCGGAACAGTCTCTGACTTTTCCTTTATAGTGCTATCATTTTCATCTTTCCTTTGCAATTGATATTCTACAAAAATAACATCTCTGCTGATGACGATCTTGTGGGCAGTGGGGTCCCACaaacgatactcctttattccatcagcatATCCCAAAAAAATACATCTTCTAGATTTTGGATCCAGCTTTATTCTTTCTTGGGCATACATAGGAGAACTATACATAGGAGAACTAATTaaaaagaACTAATTAAAACTRTTTGTCCAAGGCCCCAAATATRATTRARCCGGCCCTGGTCTACTCCACTTTCCTTGTACGTATTTYCMTTTAAGGGGRCGGTCTTTGAATTTTAAGTTATATCAAATAGAAATACTATTTCAcccttaattaagaTCCAACTTYTACAAAAGTAATGAYATGGGTGCTTTGGGTAAATCTTAAAATATATGAAGTAATTAAMTCGAATTGGAGAGTCCAAAGGAAAATTTATCCACATTTCACATTCCATGCACATTATGAGTTAAACGTTTAATAGAGWATTTTTTTCTTGAGTTCTTGTAGTTTGTGAAAAAAAAGAAAAAAGCCCGAAGWTAAAAAATTCYTTAAATGCATCACTTAAACCTTTCGAAATTAAAATTCATTTCATTCGTCCTTTACTRAACTTTTRTCGTRGTCCATTTCAAACTTGTTTACTGATTATTTTACAATTGTAAAAATCTAATCGCCTTCGCAGTaattatcatcgCAATATCGTACGGTCGCAGATTGTCAATTAATTTACTgttgattttttttgtctttATAGAGGAATCGCACCATCTTTTGACTTCACttttgtccttctttcggcaCATTYATCAGTTTCCTTAAAAAATTTGTCRATGTTTGAAAYATCGCCACCCAGTTCACACTGGCGGACCCTCAAAMTCGCTCCAGAATTAATCAAATRCAAC

>Cs1g03250

ATGAGTATATAGATCGTCTCTATAGGAATTGATGTTATAGGATTTGGTACAACAATCTTAATAGTGCATTTCAATCTAAATTAAAATAAAATAATTAATGAAACTAATGAGCTAGCTAAATTAAAAAAagcaaataaaaAAaTGCAACAAGAAAAATCTCTTACTTTAATAAAGTATGAGATTAGAAAATATGAAAAATACGTACTCCAATGTAGTTTATTTTCTCTTATAGTGTATTGAAAtttgtgttttctaccATTTAACTTACTGGTTACTTTATAAAGTAAGGAATTCCCTTCCTAGAGGGACTACGTCACTAGTATTAGAAAGTCACACAATGATATAATGATAATTAAAAATATTTTCATAATTTCACTGTTTATTTATTTCACAATCATACTTATTCTTTTTCTTTCATTAAATCAAATTGTTCAAATGTATCACATTCTCTATCACTAAAATAAAGAATAATTTCATATTGTACAATTTTAATAATTTATATTATAAACTTATTTATTCTCATTCTTTTATTAAATACATYTATTTTTACTAAGTGATTTAATGCATCGTTGCTCRGTGTGTGTGRGTTTGCACGCGtgtgtgttttttactctTTCATATGAACTGTCATCCACACAAATATTTTATATTTATCATAAATTTATAAACTAATctgagataacattaacaacctgATTTATGTTTAAATTTTAATTGCTTATATTCTTTTTAAATCaattatctttttTAGATTATTCGATTAATGTCCCCGTGCTTCGATGTATAAGACATTAAAATTAGTTGCGATCACTTGGAGTAAAGGCAGAGGCGGCACATGGTCCAGGTCATCACTCACATAAAATAACACAATCCGATAGAAGTAAATGTTAACGTTATGCTtagcttgatagatagataaacaaATAGTAAACGAGATCGACAAACACGTGAATAATAAATAACACCGTTATTAATTAATTAGTGAACTATTTTGATACCTA

>Cs1g16860

CAATATGATTGCTGTGGTAAGAGACGGCGCAGTGGTGGAGTATGGCAGCCATGAGACGCTCTTAGCTTCCCATCTTAATGGTGTTTATGCTAGCTTGGTCAGAGCTGAAACTGAAGCCAATGCATTTTCATGAGTAGTTGATTTTTGGTCTAACCGTGTAATGGATCATATGACCTAAATATAAGCCTTATGAATATATAACACCTCCATAATTTTGCTCATGCTAATAATTATATGTAGCTAAAGAATAAGCAATTGTATAAGCTAGCATATAACTGAATGTAGATACTATTGACAAGGACTTTAAAAAATGGGATATTAAATGGCATGATCCAACTTGTGAAATTTGGCATGTTGTGAAATTGGTATGCTTCGAATCAAATCCTGTCTACTGATGTAAAACTGGATTGGGTATACCAAAAggtataagataagCTTTCTATTTTCTGTTGAGTGTGTCCACAGGAAAAATTCCAAGCTTCTACTTTTTGAATGTATCTCCACTTATTAATCGTAAACTAAACAGCCAAAATTAAGTCCTTCTACCCGTAAGAACAATCCTTTCTATYATCTCTTTTAAATGAGGCACATGCAAAAATTTTGGTCAAGTGAGRCATTGCGATTTGAATGCAGGAATGGAACATTTCGACATTCTCACCTTTCCTTAAAACTCCACTCCCTCTAACTTAAAAGGGAAATGCGACCAGGAAGTGACCCACATCACAATGACTTAAACGCCCCTTTGAATAGAAGAATAATGCTCTAACTTCAATAATCTTACACATGGGCATAtaTGGGCATATTCGTCTCTTCATGTCCCGTAAAGGTGGGCACAAAATTCTCTCAAGTAAACATAGTCTCTTCGCTTTCAATCTAAGAGAGCTTGATGGTTACGTGCCTGGCATGCAAAGCTATAGAGGATAATTCATTGCTTTACCAACCAGCCTGTGCATTTTGTTTGCTGAAGCTAGCTGTTATTTTTCCATTCTTCTCTTCGCAGA

>Cs1g18520

tatgtctgtcatatgctttcgaagaaatcaatcactgtggccattaattcttaccattgtaggctattaagtgaatcctactatgctttttgttaactggaaattcaattgcatcttttcatattgccatattcattcagttaagatcattttcaggaaaacactttcaggagtgttaattaatcttgtggataatttatgagagaagctgacaaattctgaatacagcaaaagtaaaagagcactaaatctatgaatggcctttcaattttcaagataattcaaaagctaatttcaatggagtggtctatttctatttgatatataccttttgcagacaagtttctgtgtattacttactcatcaaaattaaaattgaaattttttgggcataatatgcgtagattacaaataattatgtgttaatcatgtaagcaatctgggttataattaagttcctcatgtatatgtaaaacataagaaataaaatgcaaaaagttatataaatttccaccgtacgtctattcaatatacataattgggtaagtctcagattactccacccctgtgaattgattatctnnnnnnnnnnnnnnnnnnnnnnnnnnnnnnnnnnnnngctctgacttatttctgtgtacgcatgaattttttaaaaagaagtcatacgaggagagatcctgatcgtggtcgagttgattgagcagagatcctaatcgtggtcgagagggtaaccagaatctcgcatcaataaagaacatatctttaattaaaatttaaagaataatacaaattatgaaataagatagtgttaaattttttttcaaggaataataatatttaaatatttacttgaatttctttgggataaaatttttaaaatatttatcattattcataaattacctcgattatttaagcagttcggaaatttaaagggggtgtcaagagtcgagactcaaaagcctatttgatgacaaaagattgcgtcatccgaagtt

>Cs1g10280

TATATTTGCTTATCGCATTAGTCATTGCTTCAAATtctttaaaaaaaaattgtaaTCCTCGTCATTTATCACATTGGTTCAAATTCTTAATTTTTTTTTATTAAAAGTTCAAATTCTTAAAAATTTGTGTGAAGTaaaataaaaaaattgtaaCCCTSGTTAATTAAACAATTAAAATCCAAAATTTATATTATACATAAGTTAGTAGATGACAtgatatcatcacatagtttcattatgataactacttattaaatcaatttagattagtttaattgggtcaaacgacaatattactcttacatggttgaatacatatnnnnnnnnnnnnnnnnnnnnnnnnnnnnnnnnnnnnnnnnnnnnnnnnnnnnnnnnnnnnnnnnnnnnnnnnnnnnnnnnnnnnnngtatatgtagcatcgcttgcagccaaggatcattttgctaccaactatggtgattctagaagatttggcaaattaaatggatcattctttcatctctctttaatgaaataagctatttcttaataattacttcccgtatgattctttgcctagccatcacttgcaagagtcgtttattgttgccgtcacatagtttgaattctgaatattttataaagtctattgaaataatcatAttgtttcaagaTTTAACTATTTACCGCCCACCGTATCAATTTCTGAGTCGGCCTTGGAATGAAGAGTCATAATTTGACTAGTATGGGTCACACACGTCTTACGTTTCTTCTAATAATCAAACCAATTCTTGGAAATGCTtagaatgccagATCAATTTTGCCTCAACGGACCKTGCTCAAGAAGGAAAGTAGTTGTCAATGATTCCAAGTCATGGCCAGAAATTATGTGCCACTATAAATTCCGTGAGGCATCTCACACAAAATGCAAGAGGAggctagagtcttGACAAACACTCCATAAAGCACTGTTTGTTATTTGCATCCGTAGTTCCACTTTTCAATTGGCCAAAAATCAGAAAA

>Cs1g01170

tcactagtaaaatatttccttctgtcttcgcccgtggatgtaggctaaaagccgaaccacgtaatttctggtgtcctctattgtgcttgttctttattttcttccaattttattttagctgcatgtctgcttcacccaccaatttcctaacagtggtatcagagctattggttgtatttttggagtcgggaactgttcacgtaaggggtactattcacgtatacggcactgtacacgtataagacactattcatgtatactgtattgttcacatatacggtactgttcacgtatacggtactattcacgtgaaacggtggaagcgatccaagaattttccggtgcaaagcagggaatagtagtgtgagaaaagcaactgtggttttgtacatgtctaggaaagttctgtcacaaaggcgataagagcttaaggagtctgggttttaagtgggaccattgtgacccctccagtctttcctgggaactttcctggtgtgcattctcacacatactcacaactattcaaggtggtatactagcttgtgtgcagtatttatcaacaaaatggcggcaaagtatgaaattgagaagtttaacgaaaataatttttcgttgtggaaaatgaagatgaaagttgtattgaggaaaaataattgtttggcagcaattggagaaaggcccatggagataactgatgacaagtggaacgaggtagacggtaacgccatttctgatatacacttggcacttgcggatggagtattatccagtgtggcagagaaaaatacggcgaaggaaatatgggatactctcacaaaattgtatgaggccaagtcactacacaacaaaattaaatgaggagagcaggcggaaaaataaggaaaacagacaagtaagttcgcagcaagcggaggcgctatcggtgacgagagggagatcaacggaacgtggccccagtgGGAGTaaaaatcagggtagatcaaaattcagaagtaagaagn

>Cs1g19130

tctacatgatGAGCGATCACCTGATTATCYRGGAGGTACTCTTCTAAACTRAAAAAGTACAAAATTAATAAATTATACTTAAATTTGAGCACGTGTAGGTAATGTAATAAGTTTACTCCACAACRACCAGTCAAACGCAATCTTTGATTCCAATAGTACCATGCAAATATCACCCCCGTGCCACGCGCCTCCAAAGACGGTAATTTTACCCRCCGCTCAAACtccaccgtttgtCCGTACATAAGCTATTCATAACWGGGGATAAGAATATCAAGGTCCCCAACTTCTTATTTTTTCTTAAATAAGATATAATTTGTTCTTTTATCTGTGATATGTATTTGTTTTATAAGTTTTGGTACGAAAAAAaaaaagaatttatacttacaacataaataaaaaaaattttaaatTTTATTTTAATTATAAGAMGAGAAGTAGAATGTCWCTAAACAAATGAATGATTCTCTCTTAAACTCTAATACTATAATGTGTCGTGGTTCGATATAATAAGGATACAAAATCGAACAGGCGAACACAGGGACTTATTTGGTATAGATGYATAAACTATATTCTAATTCGAGAAAATGAGAAAGTGTTCGGAAAGCCAAATGTTACAGCGTCCTAATTAGACAATTKTCACAGCGTACTTCAATATGCGCGTGGGGATTGGTTTCATCGTACTTGGAAYTAAAGAAAAGTAAAAACACTTCGGGTTTAAACAAATCTTATGCGGATTTTGCGAGAGACGATTGGCCACGTTAAACATTAGTAACATGTGGTTATATATAAATCCAGATTACACTCAGACTCAGTCTTGCCTGTCCATCCTTCCCCAAATAGCCGCTAAGATTTGGCCACGTCAGtaattatccgcAAGTTTCTTGGGGGATCTCTCTGTTGTTGGCACTGTAAAAGAAAGTACTGTAGAAGATAATTGAAGGGAGTCTCAGTTCTATTCTATCCACTTGGGTCCTACGTTGTCCATATCTCATTTGTTTAGC

>Cs1g24040

TRAAATTTTTATAAAATCTCTTTTCTATAATTTTAACCTCAATTAAACAAGGTTTTAAGTTGAGAGAAACAAAATCTAATTAAACCCCAATATAATCTTCCATCGATTAAAATAGGACTCTTAGTTGTAGGTGCCCAAATTTAAAAAATTCTCAAAAGAGCAAGAAGCTAATAATTTTTGTATttttttaaagytctaATCCGATTTTGCTGATGAAATTGAGAGGCATATCACTtagtgataatgcCTCACTTTCTTCTTCTTCTTCTTTGGTYAGAACCTTGCATGATTTTTATCAAGAGTTTCTTGTCTTCTGTAAGTTATTCTTTATTCAAAATTGAATTTGTTGGAGCTACAAACAAAGAATAAAGAGAATGAAATTTATGTAATAAGAGATTATGGAGCGATCTTATCTAGTTAATTATTCTTTTGAGAAAACTAAGCTAGTGAAAATGTTGYAAACTCCAATAAGAGATTATAGGGTTTTGTTGACAAATAAGAAGGTAAAGGTGTTTGTGAAAKTGAAATGGGTGTACTTAAAGCAGCGTAATTTTCAGATATGTTTTWAAAAAGGTGYTTGTACAAAAKGGGTTCTACAAGTACTTTAAACTGGTGCTAATAGCATCACCCGATGACAGTAGTAGTTGGGCCACCATGAAGACTGAAAAGTAAAAGAGAAAAGCTACACAGAATTACAGATCTCTCTATACATATCKTTGAGTCACGTGGCTACATTAAGTACATGCCATTTGTCTCGAAATGAGAAGACATGCAAATTTAACGCTACATCTTTTCACTCCATTGTCTTGACACATGGCATGGGWGTTGCTACGTGTAGAACCAAGGAGAAGCGAATTTTTGGTAGTATTGTCGTAAGAAGAGGATCAGAAACCAATTGATTTGCTAAATGTAGCCCTTGCagtggcatattttaactggtttATCTCACAGTTaaatttatatatataaatacaCGATCTCTAGAAACCRCAAGAAKAAAA

>Cs1g08550

TGCTTATGKYCTTAAGCTTCAGATCAATCAATTTCACAGACACTYACATTAATGATTATAATTTGATCACTGCTCATTMATGTTTCAATATTAAATTATGGTCTCCCTAAGTTATATCTAATCAAACTTASAGTAAGATAAAAATTCACTATAaattagcccacttagcccttttgcTCCCTTAATTCTTTTTGTATTGCTTTACTTGATTGTAAGTTACAATCATTTTAACWCAGGCAAACCCTAAAGTTAATTGCAATTGGATGCTTTTTATTAATATATAtaTAtatggcacgaaaaaatagttgGATTGTGTGCTGTAAYCTGAATTAAAACTCGTTTGTTCGAAACTTTTTATAGCTACTGTTGGTGAAGAAAATTCATtgtgcggggttagaaTAAAGATTTTTAGATATTCAATCTAAAACACTAATTTATTAGRCGGAAGAATTTATTCTAATTATAAACAGTTYWTTTGAWKRTAATTTTGTTTTTTAATTGATTTTGTCGGTAAGCTACACTACAGACTAAGTTGCCAGAAACTGCAAGTTACGTAAATGATTGGWTAGTGAATAACAAAGAAGAGTTAATTAATTAATTAACATTTATGAATTAATTAGACGtgaagttttayctgtAAATATTGAWATGAAAGAACGATAAGCATGAAAAGGACTCCTTCGTTAATTTTRTAAACTTTTTATGGGAGAAATCAATACATTACCAGAAAGCAAGAAAACAGTTTGATGTTAGCTTTGAATTTATAACAATAACAATAAATACAGTACAAGCCCCATTTTGTTTTTCTTGCCATATCCGCCCACCACTCtgtratctctctctctctctctctctctctctctctctctctctctatctatctatttgtttATATATATATATATGTGCACAGATGACATGTATATAAATAGATATAGGTAGATAGAGTAGAGATGATATGAAGAGTAGTGGTGTGTGGCCAAAATtcagataaaaaaaaa

>Cs1g03450

nnnnnnnnnnnnnnnnnnnnnnnnnnnnnnnnnnnnnnnnnnnnnnnnnnnnnnnnnnnnnnnnnnnnnnnnnnnnnnnnnnnnnnnnnnnnnnnnnnnnnnnnnnnnnnnnnnnnnnnnntgttgaaaataatctcatattccaaaccaaaattcatctttatcgtcaaataattataaccaaactttacaaaattaacccaagaatgcaaagaaaacaaaaagttttcattttctttaatcactgaccatnnnnnnnnnnnnnnnnnnnnnnnnnnnnnnnnnnnnnnnnnnnnnnnnnnnnnnnnnnnnnnnnnnnnnnnnnnnnnnnnnnnnnnnnnnnnnnnnnnnnnnnnnnnnnnnnnnnnnnnnnnnnnnnnnnnnnnnnnnnnnnnnnnnnnnnnnnnnnnnnnnnnnnnnnnnnnnnnnnnnnnnnnnnnnnnnnnnnnnnnnnnnnnnnnnnnnggaagataaattcgagacagatagagagtgatgcatgtgaatagatttaactttttgttattttccactttttattttttttaataataaaaaatatctacaatgctgagagagggagagaggtgaaacaacaacacaagttgtcgTTTCTCTCGGCCTTCTCTCAGCATTATAAATATTTAGTACTACTCAATCATTAATACCTAATCTGGGTTCTCTTGGAACTTTTATCAAACACGTCAAACAAATAAAATTCACCCTCCAAGGACCAAGACCTTGTTCACAAGGGAATACGAAGATGAATGAATCAAGCAAACAACACTTAATAAACTTGGCTCCCTAGTTCCTACgaatatgagcgagcgagcgagcGTACCATTTCATTTCGATTTTGGGCCTGGTCGAGCACGGGCTCATGCTTCATGTAACAAATTTGGCCCAATATAGCCAGCCCGGTCCAGGTTTCCCGTTCGACCTCTGCAAACCGGTCCTCATAAAcccgatccacgatACGTGTGGTTTCAAGTGGCACGTGT

>Cs1g03510

ACGATTTAGTAGATTTTATTATTCTTGACGAATGAAAATATATACGTTTATATTTGCTGTATATAAGATAATGACATGTACAGTACACTGTATGAAATATATAGAAAATCGAAATTAGTTAATATTTTGTTTCATGTGTTTGTGTGTTGCATCAATTGTCTTCTTTCTCTAGTTTCAGAACCCACATGGTTCATTTTTAATGGCTGCCCGCTTGAGATCAGTTTATTAATTTTGAAGCTCTATTAATATGACTATTGTTATGTCTGGTGATCCCATTAGTCAGATTTTAATTGGTACATTTGCAATTTAtatatataacttttTATTAAACCCTAATGTGTAACGTGTGGCGCGGTTCAATGAACTCATCGCAGAACTCAGCGCATGGAAAATGAATTKTATAAAatttgaaaaaaatttATATCAACATAAGAGACAAAATCGATCCCGAAGCAGAGGCCAGATCAGTATTGATTCAAATATAATTTGTTCAAGTTGAAGATGACACATCAACAAGCAAAAGATACTTTGATTTTCGGATAATAATTGAATTGATAAATAAAATTAAATGGAAGAGATCAAAGTTAAAAATAATAATAAAGCATTMATGAATGMAAAAATTCAACAAGGTTGCCCTTGAGAAGCAACCATGTGAAATCAGTGGTGTTTTGATGGGATGGCCTAACCATCATCGGTTAGTTATTTCAACCAAAGAAGAAATTAGGAATATTGATTATATACATGTAATTTAACACAAGGCAACAGTGCATATTGACAGAAGAAGAAACCAAATCCATATACAAATTAGTCAATACAAGTAACAAACGAATGAGTGAAGGATGGATGGCTCTCAAAGAAAATTAATTAAGCAATWTTTTATTTTTCCACATGGGTAACAAATTAGCTGACCTGTCTAGAAATAGTACCCTCTTGGGTCATATATATGTATGGACGGAACCCCAACCTCCCCAAGCTTCTTTAATATAATCAATCATATTGCT

>Cs1g25790

TTGTTGAAATGCATTRCATTGTTGCACACCTTTTGTTGACTCCCGGATTCAATATGCATTATGTATTAAAAAGATTGGAATTTCCCAGTTCCCACTAAGAAGACCAACAACGTTTCCTTGCATGGACGTACCAAATATAACTKAGTATTGTTTTAAAGGCTTGGCCGCGTCTTCTGCGTAAAAGGTGAAAGAGAAAACACGAAaataaaaaataaATAAATACATAATCaagtctttaattaaGATGCTTGTCAACCGTGAGAGTTCAATGAAAATCCTAATTATGTAATTAACAATGTGACCCCAATCGAAATGCTCCATCTAAGTTTTGAACTTATWATTATTAAATTAAGTGTCCCGTTGATTTTGAAATGTCTCAACTTACCCCGTGCGGTCTAAAAGCTCGATTCTTCAGAATTTGATTAGTCAGTTTGATTTTACAAAAGTTCATTACAAAGGTAAAAATATTTTAGCTTGTTGAACTTTAGTTGTTATTTCGAGNTGATTTTAGAAGAACACGATAATGGAAWTGTCATTATMATATTAGCTKAKGCTACCAGTCTGMCAAGCAGCCCRTGARGATAATTCTATCMATAAGGAACTGTCGAGAATGGAAAATACATTTTTATTTAATTGTAGACAAGTCTCCGTCTCATTTCAGAATATAATATTGGCCTACGACAAGCTTTCCAAATTCCCAGGCTCCCCGACTGGGACATTGGGTCAAGCAGGCttggattttttgtcaaaCACTCGATTAAACTTCGATACGTTCCATGGAGGGGCCACTTCTATTTTATTTTTATTTTTGAATAATTGCAGAAAGCAGATTCAACAGGCGGAATCAATGCGGACGCTGAGAAAAATGACTGTCTCCACCGTGAGTCCCGGGCCAATTGCGAATAAAACACCCCTCTCGAAGCCATCACCGGTTGTGGGTTTGGCCTCCTGCATGCTGTTCTTCCTTATCTCATTTAARGTCCATTCATCTTCATGATCTT

>Cs1g03040

CAAAAACGATTTACGTGTGCATATCAGAAAATATGCCCCCAACCAAAAACTTTGctctcaaagtaattTGCAGCTGGAgtagtattttttTTCCTCTCTCTTTTTTGGTCTGTGCACACTGCTTGCTGCCGATTAGCGTTACCTAACACAAGAAGCTTCGGCGCGAAaggttaaaatgcatgGATCTGACTGTGATTGCGGTTGAAACAGGAAACTGGTGGCTACGAAAGAACATATTATTTCCATCAACTCAACATGTGCTAGTTGATTGCATATTTGTTTAATATAGTGGGGAAaaaaaggggtaaaatTACTTAAAACGTTTAGTGAACATCCAgaaaaaaaggggTAAAATTACTTAAAACGTTTAGTGAACATCCAATTCTGTTGTTTGATAAGCACTAATTTGAAACTTTTATCTTATAAAAATTAATTTTTTATTCTTTATACAATAAGTGTTGAAATGCCGAATTTTTAGAGAGCGAACTTCAATTTCACACTCACCTGAGTAGTTAAAATATGAGTAATGATATAGTTATAAATTTTTATACAAACTTATTTTGTATAAATTGATGGGACatgAtaAaattagttgaattaaatatcacttgccccatatgatttatttatattattttatattttcattcaaccaatgaattattgtcacatcaatttgtacaagataagtttatacaagaatttgTAACTAGATCATCACTCTTAAAATATTTAGTAGATACATTTAAAATTTAAATTAATGATAAAGATGCCCTATAATTTTGTATTTATCAAGTTCATTGGAGCCTCAAGTGTATATAATTTTCCtTTAAATCTACTGCCCATATGCAGGAAATGTGACACgtgtcaaaaagtacgtAGAATACATTAAACAAATCGAAGGATGTCAGTTGAGTTGACGTCAGCCTATGACAGGTCCATGCACTAAAAACAGAGTCCCTTTCGTTTTAAAAGCTCGCAGAATGAGAAGTGAGAATCTG

>Cs1g12820

AATTTTTAACTGATTTTGCGCTAACGGTTTAACAATTTTGCATTTCCGACRAAACACGAGCAATGRAACAATTTGATACCATTTATGGTATAGAAATATAGAAAAATTTGTATACAATGTATCCYTAAATCAAGAACTAACACAATAAATAGTTTGGTATGAGATGCTTCGTGCCATCCCTTTTCTTAATGGCTcaaaacaaaaaacattaTAAAAACATATCCCTTCTCTTAAGGTGCCCATATTGCATTGCAATGAAGGAGGGGGTTTGGTGCTTGACTCGGCGACAATGGCGTCTTTTATGGGCACCTTTCGGTCCCAACCCCAGCTGCCATTTCTTATGCCMCACGTTTGCATTGAATTGTCTAATCTCACATGCGTGTGGGTATGAATTATTAGTAATTAGGTCTTCATTTTGGTAAAAAAAGTCAGcaaaaatattaTTTTTGGGCACTCCTCCACTTCTTTGAGTCAAGCCACGTGCCACAAGTCATTTTTTGCCGGCAATTTTATTGCTGCTTCTTTCAGTCTCTACACATTACAATAAGTCTTACATATTTTCTATTGCATGCTTTTTCATCTGTTGGATTCTTACACACGCCaatttttttttttttttagnnnnnnnnnnnnnnnnnnnnnnnnnnnnnnnnnnnnnnnnnnnnntagtatgataaataatagacaaaattatTTTaaaAATATAAATAATAGTCgTTTTTCTGTCAATCGAACCTAATTTTATTGCTTCATACCGAATTAGAATGAATAATATAAAATTATTTTAATTTTGACCGTTAATTTCAAAACAAAGGTGATCTAAATGGGGTGCGAATTAAAGCAAATGCACTTTTGACCATACAAGCATATTCTTTTGAGATCAAATGGTTTCTTTATTCAAATAAATGCTCTTTTGTCAAGGAATCATCATTRACACGCCCATTTAAAAAATAAGAATTATTTGATGATKTCCCACACTATAAATATAGAAGCATGTTACC

>Cs1g08430

TCTCGTGCTTTATYGTGAAGCCTTYTTATGGATTTGAGTGAACCCTTATTATGGTTGCCCTATTTTACRAACTAATAGCAAGGTGAACATAATGAGATTTTYAGTAAAGCCTTATTATGCTCACCCTATTTTATTACCCAAACATCTTTCTTTGTCCTTTGCCTTATTTCTTTCATTAAATATTTACATTGTAKTARTTTACGCTATGATGGATAATTAWATCATCATTTTGTATACAATTATGCTAAGATAAGTTTATTAATGTCTTAATTTAATTTWATTTATTTTTCAATTGGACCTGCATAGAAYCATAATCACTTAAAATTAATACTCTTTATAAAATTATAATGTAGTAATTAATACYATACAAACAACAAATCAYATATTAAGAGAGTGCARTTTAGAAGCTTGACRGAGTGGTTTTCAATCATTTTTATTTCGRAAGCTACTTYAYTTCAAATTTCCAATGATAAAACAGGATAAAAAATGATTTGAACTTTYCRTACCCATAAATTGTGGCTCCAAAAAAGCTAACCTAACCATcggtttgcccgattTGGGTCCAATTTGCACACCAagAAaAAgACAATCAATTTAATTAATTAAATAAAAAATCGACTTGAAAATTCGataaccaaaaaaataaatATTTTAAAAGTAAAAAAAAATCGAACCCAATTAGAACCAAACCAATTTTGGTTTGGTTCATTTCATAATTTTAATTATGTTTATTCCGTTTAGCTTAGttcaataaaaatcgaaCCGACCGGACCGAACCATATCACCTACTACCCCCTTTAACTTctaattaaaaaaaaaaaggaaaaKMTACATTGAGAAYGCCCTATGTGTAAAATGCTTCTTGGAATTTGTTCGGGTTCCAaaaaagaaaaaaaaaagttaatAGCAATTCAATTAAAACAATCCATCTTTGAAATTCCTAATATTCTTGAYCAGGCTTTTTTAAGGGCCTCAAACCATCTCTGAAAACAAGGGCTTGG

>Cs1g04910

ATGTTAAAAAAATTATTTTTTGAGATTTTGTTGAGTTTATTAATTAACTATTTGATGATATTCAGACCGTAAATATGATATCTTTTGGTACTAAGGACCCATTTGTGATAATTTTTAGAAGGTTTAAAAGTGATTTTGAAAATTTAAAAGTTAATTTTACTATTTAGTTAAAAAAAATCAAAACTACTTTTGTCAAAATCAACCCCTCTTACAgctaattttaAAAAgtaggaaataaAAtagtttttagattctaAtTTTGAAaATTAATttTATTCTTTTATATAATTTCATAAATATCCTTAAAATCATTACCTAAACCCAAAAttatcattattcaaattagaaataaaattaatattttaataaatttttattataattcatcaatatcaatttattggtcggattattattaaatttgtttcactataatataaaattcattannnnnnnnnnnnnnnnnnnnnnnnnnnnnnnnnnnnnnnnnnnnnnnnnnnnnnnnnnnaataaaattattacaatacataattaaaaatcatgtttttatatatataaataaATTTTACCCTACATTATATACATTTTATTCAtttttttaatagtTTAAAAATAAAATTTATCAAATGTTCATAATTGTTTTTAAAACTCACAATGTTTCTGAAAACAATAATTATTTTAAAAGTTACAAYATTTCCAAACTGACCCTAAATTATTAGACATCTTAGACCGCCAAGTTATATATTTTTTTTCTACAAATTATTGTCAAAAATTTACAAATAAACTTATAAAAATAATACTTGATAAAAAAATTAAGACTTTTTCGGAAAATGGTTGGTTACAGATTTCCGTACGGCACTCAAAAACCGTTATATTTAGACGGATTCCAACTTTGGCAACACATTGCCTCAGAAATCACAAGCCAGCTTCTTCTATCATTATTACGCACATTTCTTTGTTTAAGTGAGAGTGAAGGATAAaggccagagagaGAGAAAGCA

>Cs1g07890

TCAGTACACAATAATGTAGCAAAGCGTGGGTGGCTAGAGTTTTGTAACCATCCTCGAGACCCTATGCTGCCAGTTGTAAAAGAATTTTACGCCAATTTGGTAAGCCCTGGTCAGCACAATGCTTGGGTAAGAAACTCACTTGTTCCATTAGACTCTCGAGTTATAAATGCTTTCTATAATttacctgctgaaattaattgtgagtatgccaagttgcttgacaaattgaccccgcaaagatggaacacaattttcacgacacttacagtagagggtacatcgtgggctaatgaggaagggcatgtaattaacaagatagatttaaaacccattgctaaggtgtaggtgaaatttctgaAATCTAGGCTTATGCCAACCACCCACACCACTAATGTCTCATAAGAAAGGTTGGTATTGTTGTATGTTATTGTTAGAGGGCTTCCCATAGATGTGGGAAGCATTATCGAAAAATAAAATTTGGGACTGTGCCATGAAGAACCACAAAGGTGCTGCCTTGCTATTTCCTTCACTCATCACCAGCATCTGTGTAGTATCAGGAGTTTGTCTTGATGCAAAAGATGAACATGTCAAGAATGATGGTGCCCTTACTGCACGCACTATTGAGAGAATTGCtggtgaaactgctggAgCCACaTCTAAaCCAGCTGCTGCAACACGGGCGagacGAGCtaTtGGGctagaaaatgaagctgtnnnnnnnnnnnnnnnnnnnnnnnnnnnnnnnnnnnntgaacaatctgaccaacatgaggaagagggtgacaagtctgttggtgaactagatttagaggaggtcctgactagggtttctcacgttctttctcagacttttcctctgcctctgttggtgaactatcagagaatacaagcactgagcaccagcatcactcaatgtgctgaggctcagcagagggaaaacgatcggttttggagctaCTTACAGCACCTGGAAAGCCACTTACACTAATTTTCTCTATACA

>Cs1g19320

AGGAAAAAGAATTATAATTTTAACAAAAGGTATTTTAGTAACTTGTAATAATATATTTTGATtatatatataGGAATATTGATAATTTCGGtttcaattctaattcttaaaacttaaataaccgacctattataatattctccattctaatttgataccatcccaattttattttaattatagtgcnnnnnnnnnnnnnnaaatattgagttccatctttcgaatgatcctacattgtagaaaagagcatagtagactagggacaaagctttacctaacgattttcgcactccgtggctccaattaaaaatgaatgaaaggctgactaggattggcacaggcgtcgtggattcatgaattttagctcaaagcgtgtcctatcagcgcacaactttaacaaataaaataggaaagcggaattaatgaagacaaaattgctttgctttgctccgagtcatagaagcgatggcaactggcaagttggcaacattggcaacttgtttattttcgctagaagcatcatctcacagttaaaTAAATATTTAAGAGaaaGGACATTTTACGCTTGAAGGGTTGTTCTCCCtcgctgtttttacacaTAATGACATCACCATTCAYTAATAATAAAACGTATGATGAATGACATCAGTTAGTCTGCAAGTGATTGTATCATCACCAAACCAGTCCACGCAAAGCGCAAAAGATAAGAGTTTATTTATTTTAATCGAAGTAGTGGCCGACTAAAAAGGATAGATGCACCGTCCGCCGCTAGTAAAAAACGCACCAGAACAAAACCTTCGATCAAGACATTCGAATCTCTATCATTTATATGAAGaagacaagtgctctttcccTTTCTCCCCAGGCGTGGAAAGTTGGATTTAGAaattacaagtccaAAAGGAGGACACTTTCTTTCAACTTCTGTGTCCCCACCACATTTTATAGATCACATAAATTCATGCACTGGGACTCCTCCCTTGACCCTTGGGATAATTTAC

>Cs1g15840

TGAagctgtgagctcttGTGATTCCAAGATCTGAAAGTTTGGCAGACWGTGATAGTTCTATAGGTTTATATTTTTTCTTTTTCTGGTTATGATGATCTATCAATTTATCCTATTGCGCTTTGCTGTCTGTTGATAGATGGAGGTTGGCTGTGTTGTGTGGCAAGTTTAGGGGTTCTGGGATGATGACTTTTAATCATtgtgctgacttgTGTATACTTTTAGAAGGTTTCTGGTATGAGGGCTAGTGTGCGCTTAAAATCTACACAGCTTGTGTATACTTWTGTGATGTAATGTAATAAATTTATACTGGAACTCTCTCCCTCSCTCTCGATTGTTATGCCTTATTTTGTTCTTGTGTGACTTGGAGCTGGTTTTCGAATTATGATAGAATCGCTAACCATCAACTAAAAGTGGTAATTGCACAACTGACCAAAATCAAACCGTTTTCGAAGAAATAAGTTTTGTGAAGTTAGTTCACCGTCTCCCTCAAATGGTTTGAACTTCCCAAACCGGTTTATGTTTGATGGGGGTTAATTTCATTACAATTTTCTCATTGTCTTTAAAAGAGATAATGTAATGTCTTTTGTATGATTCGGTTCTGAACAGTAATTGTATTGTGACCATCCAAGCAGCATCTTTTAAAGATAaagattaaaaaaaaaaaagtaaaGATAGAAAGAAATATTCCTAGGTGTCCAATATTTTTTATTCCTTATCCTACCCAATTTTATCTAATCTTCACTGTCTAATTAAAATAGGATTGTTRAATTTTATGCGGATKAAACAGCTATTTCTTATTCCTRGACATTCAAGAAACTGTTTTGTARCCAATTTAAATCATCTAGATTGGATTGATTATTATTATTATTGTAGATAAATTAAGATTATRCCGTTACAGATGGAGAACGGTAGAAAAGAAKAGAAAGCACTTTAAGGACACGTGTAGTATAAAAGCGTCGTGCACTtcagtaacactgacacacagcaTTGAGTARAGAAGC

>Cs1g12040

gttatacaattcctatgtgtgatagaatcctgtaaatttCAacgaagagaatgaagaaaactgatgagtagaatacaaagaatttgcttctgggcttagcaaaagacaagatgtttcgatttctgtactacaacgttcataagcaaatgtacatagttgaatcaaccaaatcagggggaaaatacaatacttaatttgaattgcaagcacgactaactactttgcaattcagctctgacttgaattttttAATAGGGAATCATTTGCATCAAAATGCTTAATTGATTTACGACAAACGATTAAGGCAATTGATAGTGACATATAATCAAACCTTCATAATCTTCATAGCATAATATTAAAACAAGCGTAGAATAATCATTTCATGTTTCTAGAATTTCTCTTTAAataagaaacaagaagtaatgataacaattttaggcaaaagtttcaaaatcatagctgtcaacatttctacatatttcctacatttttctacaaacataaataattgaataaaaaaatgatcggaaaactaaccactggggcaacttcaatgccaccttggtttctctggtatcctagaactgtacatggctctgaagaatgaaacgaaggtaacagtacaaaatccgccatctggacaactaataaatcgtgcaaatcagcgacaaagctggcacggtggctcccatcatcaagaatcgtttagtaatggcgtttcaattatacagagatagctcatcatacctttgctttgctcctgctcaagtgaaaggggtggtatttcgtactgttgtcaatggaaataacatttgtgccacaaaannnnnnnnnnnatataaactgaggtcttgtccTCTGGCAAGTTTTGTTTTGTCTTGTCTTACTGACAAGTTTGCTTGATATATTTTAAGGATTGRTCGTCARCTTYTTCTTTTACTTGTTTTGGGGTGGATTCAYTCTGTTAATCACAAGTGGTATGGCATCTAATAGCAATTCAA

>Cs1g24560

TGACTTATCATGGAGTTTTTACCTTTGTAATTGTACGTACAATGACACAATCTTCCTAGTGGCGGTTTCCTCTCCTCTTTGGCCTTTGCTTATTTGCTATGCATGCTCGCAGTGCGCTCTAGAGTCCCATAGCATTAACTTTGTCAGTCTTATCTGATCATTAAAGTTTCTCAGGGTTGCTTTCTTTCACATGTAAATTAATCTCGAGCTGTTGTGATGAATTCTTGGTATTACTTTTAGTCGTKATTACTCTTCACTGAAGAATAGAAACATGGCGTGGTTAGACTTCTAATAGAAGCCTTCATAGAGCAGCGGCTTAATTTAACTATAAGCCAGAACTGAAGAGTGATTAATGCATTACATAGTATGGAATCAACAATTGACTAGGCATGTTTATAATATATATAGTGAAATCATTACTCATCATGAAAAATAAGTGCCAACTGGYTAAAATATTCTCGTTCAWTTTTATCTTTTGGTGGGCAATTGTTGGGAAAATAATTCAATAGACTGATCAATGCAGTTGTTAATAAACGCATAAACTCACCATGTGTTTACAGTATTCGAATTTYGAACAAGCCTCGCRAGYTGTTAAAGCTATGCACATGCCTCCCTTTCGATAAAGATAWGAATTAAGATGGGGCTTACTTCTGCAGCTAGACGGAGCTTTGTTTCAGTTCTGACATAAGGGTTATATAGGCTTTTAACTTTTTATTTTGTTTTTTCGTTTACTAATAGAGGRTCTAAGTAAGAGTCCAGCAACGTAGTTTTGGCCTCTAACTAATAGGTGAAATAAATAACCAGATAACATCCGGTAGTACGTAGTTACATATAGACATGCAGACCAAGTCCARGACACTTCCATCTCCCATCTTATCTAAGAGCATCCATTAATTGTAAAATACTAYAAATTAGAACACATGGGTATAAAATAAAAATGGCATTAGCGGGAGGGATAGTGCGTTGATTGGGGAAACTGGGGGTTCCCAAGGCAGTGTTCA

>Cs1g02990

caaaaaagaaaaaaatcaattgattagaaaaagatgccactgcacaaatacactgtacacaaacatattcctgtgtcgtttgttattcttgatatggagtttatttgctttcctcccgttaatggggtaattcaaatttgccgaaagtggacctaagtgtttgctggtccataattgaggcttccaaattcaacctttcgggtgcgnnnnnnnnnnnnnnnnnnnnnnnnnnnnnnnnnnnnnnnnnnnnnnnnnnnnnnnnnnnnnnnnnnnnnnnnnnnnnnntctaaaatttaAGACCTCTATATTTAGTTTTTTTAAGTACTAATAAATATTATTTTATAAATATATAATATAAATTAaTTATAAAaaattaatacagtacagtgcaacaccaaacataatataactaaaaattaatacagtacagtgcagcaccaaacattgtacaacattattataatacagtattagtgcaatacaacataatacaatacaccagcataaaaataatgcaatacaatataatacaatacagtgtaCCAAAcacACCCTTAATACCATTGCACGACCTTGGCAGCCCATGTTAATTTTCTCACTTTGATTAGTGTGGTTCGTAGAAGGAAGTCGCAAAGTTCAATCATTGCCCGGTTAGTTGAACGTCGCAGTCGCATTTTCAGCTGTGTGAGAGAGTGTaccttacgttaAACgtaaggtaGGCAAATCCCTTATTATATAGCTAGATTAAAGNTAGATTAAAGTAACTTTGTCTCCATTTRATTTTAAGTATTAATTAAAGATAMAAAATTGAAAACCCAGTAAAAGAAAACATAGTGAAGTCATATTTGATTCTTTAACGGGTCCATTATAAAAGAAAACCATAATTATGAACCCGAAATTAAGTATTTTGTCATTAGAATGTCAACGTCGCCGCACTAGCCACTAGACAAATTGGTATAAAATGCTTCSGAAGACAAGCAaartgaaaagggcaaGCAAGT

>Cs1g04240

AATATGAAACTCTTGGAKTTTTSAWAAYTTAATCAGAAGGGAAAATGAAGGACAGATCGATCATTTCTGGTAAAAAAGTCTTGTGCYTGTCRTTAMATGATCCCKACAGTGTATARTCATTTTCCTTTATCATTAACCATCAATTATTWCTGRATATTTGTTATCATAAAATTCGGATAAAATAATCGTTGATTCKTTTCAATRAAACGGAAGCTTCCTTTGTATGTTTCTGRGGAAGTCAAATGATCAWGTTGTTMTCACATGCGTATGCATTGAACCATTGTAATTAACAAAGTGTATCAATTTTCAATSAGTCAAGGTGGGACTGAAAAACTCTATTAAACTTGTGGGAGTGGGACAGATAATCTATTTTTGAAATTCATWACACTCTCAGTAATTTAGTTTAACAAATATTTAACTTCTTTTTATCTWATTTSTTATCTCATCAGCACTGCAGAAGTggattatttcataaGCAGCAYCAATccaaaagtatccCTAAATAAATAATTTTATTCAATATTAGGATTTGTTCGCCAAWGACTGYCTTTAGATTTTTARAGTAAATAAAATTACTATAAAATTTAATTATATGTACGTTAAATTTTTATGAAATTTTAAAATCAYTTTCYGGGGTTTGTATAATAAAAGCYTATaatagtaaaaaaaaatamttMTCTTTAAAATAGTATTTCTTATTTGTATAWTMGAGTAAATTTTATAGGGAGtttagatttttttttcgcggTGCATCAACTTTCATTTTATGTMTCAAGTACAATTTATATATTTTATTTYTATATTTGAGTAAATCTTATAGGGAGTTTAGATGTCTTTTTCGCYGTGCATRAACTTTCATTTTTARAAtttatatttttttatttaATTAACTCACACACATTAGTGAGGCTCGAACCTYAAACATGAGTCTTTGAGTGACCACTTTTTACCATTGAAACTTGACGTCGCCGTCTAATGTCAAATACAATACGAGATCAATA

>Cs1g11050

nnnnnnnnnnnnnnnnnnnnnnnnnnnnnnnnnnnnnnnnnnnnnnnnnnnnnnnnnnnnnnnnnnnnnnnnnnnnnnnnnnnnnnnnnnnnnnnnnnnnnnnnnnnnnnnnnnnnnnnnnnnttattgtttaaaaaaattaagcgaaatctctcttatatttgaaattaagtttaaatgcttctatagttatattaactgtgttaattttatatgttaattttttattttacttctagtcttgatgtagattataaaatctaaaaattttaaaaaatagnnnnnnnnnnnnnnnnnnnnnnnnnnnnnnnnnnttaaattaatttaataataaattgtaaattaagaaaaaaaaattaactaacataatatttatgtagctactagcttgcattcattatgattcttgannnnnnnnnnnnnnnnnnnnnntttttttcccgacaaatcacgaagagtagtgccattcagcaaaaggtcatattgttttggcttcttcagccttgatgctttcttccccttgacaaacatttgaagaagctacagcaagccggtttattcaggtaattaagctattttttcttttagattatattgcactattgattnnnnnnnnnnnnnnnnnnnnnnnnnnnnnnnnnnnnnnnnnnnnnnnnnnnnnnnnnnnnnnnnnnnnnnnnnnnnnnnnnnnnnnnnnnnnnnnnnnnnnnnnnnnnnnnnnnnnnnnnnnnnnnnnnntccgtaactcaaaatatgtgttcagatcatgtctttcacggatttggctcagcatattttgtaaatcttatgtaagcatatttgttccacataagtnnnnnnnnnnnnnnnnnnnnnnnnnnnnnnnnnnnnnnnnnttatctttattattagtagttataatatttaatgtaaaaaattgatgcaattttgaacaaaaattgcatttggcagaaaccttatgctaatgctatactgattaattctcttctaggaccagccaa

>Cs1g26680

AAAACAGAAGATGAATGAAGTTGAGGGAGGGAATtggaaattttttactaaAAGCGCCACTGCGCCAGGCCTACATATTTACCCATCACGTCGTTCAATAAAGATCCAGTAGTCAAAAGCGGGTGTACTGAGAAATATACAACCTACGTTCCATGGGTCTCTAAACAGGCCCAGATCTGGCTCAAAGAACccataagattctcgccagattacgattttttttttaATTTTTTAATTTTTTAATTTTTTTTCGAAAATCGATTTTATTTTATTTTCTAAGATTCACCCACCCCCACCATGCTCAACAGAACAACAGCATGACTGAGCTCTCCTTGATTTGAATCCTAATCTAACCTGAAGTCAGAATACAACAATATTTTAccagtgtctaatTGTGAGATGAGCAAATATTTTCTTTCTTTCGATAWATTAAGAAAAGATTTAGAACACATTTTTCAAAATAAAAATTAATTTGAACTACTCGCCCCTCTACtttttcaaaaaaaaaaaaaaaattaacCAATTTATAGTTAGTACAAAAACATTTTATTTTGTATGCATTGAGCCATAAAATATGTAAAatttatttccttcgtannnnnnnnnnnnnnnnnnnnnnnnnnnnnnnnnnnnnnnnctcggGACAcGTCTAAACTTTGGTTaattacttttgatttTTCTCCACCACTGGGTTGTACCCTGTTGAGCTTTATGGGTAAATTTACCTGTACATTTAAAATATTAAAGAAAGGAAATTAGCTGGGGAAAATGCAAATTGCAGACACAAGACAAGCTYATGCTGATCCTAAAATTGGAAGATTAGAAGAAGCCAGGAATCTTCGAATCGGAACAGTCTTCATAAGAATGTTTTTGTCWTTTGACTTGACTTAAAGTTAAACCCCTKCAATCGATCAGGCCAAGCAAACCAATATctcctcttctgtcTTCTTCTCTTCTCTYCATATAYCAACGGTTTCAGTTTTGAATCCCCGCCATTTGCA

>Cs1g02090

tcttattatcaatttatcactaattggttgtcttgttcattgtgtttatgatttacatattttttgtagatggaattccagaggaagcaatactttctactgagcagttgattgatgaaagttggatgagtgagcaacaatcaaatgaagtggatgattgagagtttagtctttagcactagtggtttgaaatttgaaatttaaattgtagaattatgttatggatttgtatttgtggatgtttattactttaaaattttgaattttagattctatattgtgttgtatttgttttagaacctggataagttatatgttttgtatttgaaattttgttgagctaaactaaagtaatctggattctggnnnnnnnnnnnnnnnnnnnnnnnnnnnnnnnnnnnnnnnnnnnnnnnnnnnnnnnnnnnnnnnnnnnnnnnnnnnnnnnnnnnnnnnnnnnnnnnnnnnnnnnnnnnnnnnnnnnnnnnnnnnnnnnnnnnnnnnnnnnnnnnnnnnnnnnnnnnnnnnnnnnnnnnnnnnnnnnnnnnnnnnnnnnnnnnnnnnnnnnnnnnnnnnnnnnnnnnnnnnnnnnnnnnnnnnnnnnacccggaaatggttatacgtgtgtatgccaacctcgtgcaatcgacttgcctgtgttgtcaaaatagaagaagaagaaatatagaaacttcatataatgagccaagtttaaaacaaatacactcaaataacccaaaaaaaaggccaaaattcaaagtctatggactagcgatattgcttgcttccacttgctctcataattgagctacgtaattatgttatccacttcctgcatgggcctctgaaatagaaccgcttattttgactaacaacagtccacaaggccgtccaggcatatatgtatgtataaacaatactgcctatatgtatatatacacgctcccgttctttttttgatttatctgttatggtgccctcgtttgctcatgagcttataaaaa

>Cs1g13900

GCTCAAAGCGGTCCGATTACGCAACTTTTTGCTTCCTGGAAGTTCGAAAAATCATCTGCGCACTATTGAAAGCAAATTAAGCATGATATAAACGACAAAAGGAAGAGGGTTGGTCATAGTTTAAATAAATTTTTTAAGTTCTTATATCTTTGAGATTTCGCACTCGAATGGACAACCAAATAAAGGTGCCACAAAAAATTGAGTGTTTATAAAATCATTTCAGTATTATCTGGCAAGTATGAAAGAAGAAATTCAACATTTTCCACATTATATTCGCATTATTTCAATATTCATGATTTTTTTTGAAATATTCATGATAATTAAATATTAAATTTCCCATATCTAATTCGAATATatgccaaaaaagaaaaaaagaAAAGaAAGTATCagagTCATAAAGAAAGTCGAATattattaccccaaccccaCCCAGAAGTCACGCCAAAATAATGATGAGAAAAAAATACGATTCGACAGATTACTTGAAAAGctcttttgtttttgtttttaaTTGAAATATATGAAAATAGCCAAATGGCTCACAAACGATGGGAGTGTCACAGCCCGCCTTTGTTCACTTATGGGGTCCATTAATCGCCGTGCATCCCATGGTGCTTCTGTGTGCAACTACACATGGACCACCATGCaacgtcataatTCTTGTTCAGCCACGAAAACAGATGTTCAAATTGACTGCCTTTGACTTTTGTTATTTCTCAAATAAGTGACATTAGCAGAGTCTCTTTCGACCTTTATTGAATAATgtgacaaaaaataaataTAATGGAATAAATAATAATTGGAGAAAAAACttcacaaataannnnnnnnnnnnnnnnnnnnnnnnnnnnnnnnnnnnnnnnnnnnnnnnnnnnnnnnnnnnnnnnnnnnnnagtcaaatgtaTCAACGGAGGGTGAAGAAAATATGGGAAAAGAGTTAACACGCCttacattcgcaaaATAAAAGAAAGAGGAACTAATAAAGTTTGAGTGATTATATA

>Cs1g19110

aatatatgcaggtgatcatgggtttaataggtaaaatctctagaacctcccactatttcatagtaccccgaaatgcacctatcatcccaaaaattttgttaaccattcttgttcttcttattattatttgcttattttgCAGTTTCACCCCTCTAAttAGTGTGATGTCAGAGGTtTcTGGCCCCACTTTAAATGCCTAGGAATTTTTGTTTCAGAAAAAAATAAAAACAAAATAaaaaaatttaaGACATTATGTATAAAAATATTTCAAAAAactcaatagaaaaagtccatcttgtaacaaatatatgcgctaagatcaatatcacaaatcattgattggttgtgtggtgatctatgaacacctccataccttatcaaggatttgagTTTTACGTGGAAGTATATTTTATTTTTTTCATAATTTTTCGTGATTAGTATATGTCAAGTCAAAATGATAAGATATGTCTTAAGATGAAAAGTCTTAATtggtcgtcttcctacaaatacctaaagaatatgtcattaatgtggttgaaatgtatgtggcacatgaaactttcaaattaagtggtctaatcaaattaaatcatggaattggtgataaatcaagctatcagcatgaattaagagactaaagaaaggagattttatcaacggacggaataaattaagtagataaagaggtgactcattgacactatatatctaattctgttttaattaattacatcagtttattagtttccaataaaagaaaagtcgtgaatagagacatattaattggatgaacaaaattatagaatttttttcttttttttttcttgttatataattaaacatagagttagatatccaaacatgctacGTAATTGTATGATTCCTAACTTTACCTAGCATACAAACAAGATCAGTAGGAGAATAAATACTTGGCCAAAATGGTGAAAATTTTCATCAGAAAGTTAAatcagaaaaaaaataaaaaTATTGAAGTTCAACAA

>Cs1g25010

GTTGTTTAAAATTAACCCAATTCAGGTATCCTTGTTCGACCACTGCTAGATCATTGGACTCGTTTGAAGCAACCCAAGAACTCGTTCTTTCTAACAATAAAGCCAACAAGAATAGCTTCAGCAACATTGTGAAAGAATGAGAGGAAAACGCGAGGCTGAAGATTTGAATTAGTAGTAGAAGATAAAGAGTGTAATATTAACAATGAGGACCAAAAGSCAGAGGAATCTATCAGGCTATGGCTGTATCTACGTTGGCCTGTACAATCACATCCTTTAAACCCMGCATTGATTTTTCAGTAGAAKCACTGTTTCTTCTTGTCTCTTTTGGTGAACCGAGAATTATATCCATTTGTTATGCAATAAAATAGTGTTTTGGKGCAGCTTTTTCACAACTCTATAGACTCTGGTCATCAGATAATTATAAGGTGTTTCCGTGAATTTACTTAGCTTTTTCCAGCTTCCATTGACTATCAGTGGCAACTTGTCTTCTACAAAACAACACaaactaataaacaAAATTGAACATAAATGCATGATGGGTCCACGTAGATGTAATAAAGATTAACTTGCTCTTCCWACCATTTTGCTTTTTTATCTTTYTGAGTTTTTCATATGTTAAAAGAAAAATTGCAATAGGTAGGCTACAWTAGAGTATGGTCCCTTATAAATTMCAATTTCAGTTTGTAGTRTGAGGAAGATTGCTTACSGATGTGACAGGAGAAACGACAAAGTGAAATAGTGGTTCATGATggagatactacccaaAAACGaTGACGCATTGATCTCGGCTTCGTCATCCATTTTTATTTTTGATCACTTGCTTTAAAAATAAATCTTAAACTTGGTCACKYGTTMAGCAAAGAATCCGTTGTAGTCTAGGTGGTTAGGATACTCGGCTCTCACCCGAGAGACCCGGGTTCAAGTCCCGGCAACGGAATTTTTTTATAATGTAGCTCTTTACTTTTATTAGTATTTatttattttttttttaactttCTCTG

>Cs1g21880

AAAAGTGATAAGTAAACATATTAGTGATTTTATTTCATGTTGATTAGAAGCCACAACACCTTAACATTACAGTAGCCAATTTCTAATAATTACTTATTTTGGTTGGTGTTTGGATTGGAATTttttttaaaaaattactTTCGAGTACCATGCCCTGCGCAAAAGAACATCTAGGGTGGACATAATTGCTTGAAATGCAAGCCACTGTTCGCTGTTGTAGGTAGCCAAATCCTCCATAGACCGGGTGGTGTGATTAATGGGCCATAAAAGACTTTTGCCATGCATTATTGGATTGTGGACCCGATGGAACAATGAGATGTGCAATCATAATGTGGATTATTGATCAARTCAGAAATTTCGCGTAGTCAATTTTGTCGCAAGGGACTCATAAATTGCGCGATGTGAAGGGACATCCAAGGGAGGCCTGTGATGTTTACGAAAGCTATACATACATATATACATATGAAAGGGTTGATGGATAGTTAACATCTAAATTAATTTTTTAACCTTTATTTTTCTTAGGATTCGAATTCACATGGCCAGTTTTTATTCTTTTCATTAACTTTGTATAAACATCATTTATTGAAGAAGAAATCCAAATATTGCACTATTTCTATATCTTTTATAATAATAGTAAAAGGGTAACCCAATTATCTATTGCCCTAATAGAAAGGCATGTAATAATAATTGTTTCGCATAAGAATAGAGACCCACATTCCATTCAGATAGCATCTGAAAGTTACCTGCTTAGATGGCCTTTTCTTTCTAATTTTGCTAATCAATAAATATGCGACAACTTTCATTTTAATGCAGTGAAACAGATACGTACAAGGAGGATGGACAAACTGATGAGACTCTTCAAAATAAATCAAGTAAGCACGGGTTAGAGATATTGGATATTCAAGCCTCTTAAGAAGTACGTTATGATGAAGATCTTTAATTTTGTGGATGGGAATATGCGCCACGTGGAAACTCTTACCTTTGGCCGCCAATAAACATGC

>Cs1g09370

CATCTGTAATAWATTCATCACAAGAAAATGGCTGTGGTTCCCATCAATCTCAGTTaatttcttttctttcTTCTTTTTTCTTATTTGTATGTAGAAAATATATTGCATCTAATTCTGTATCACATATATTATTTGGTCAAGTATATGCAACACTTTTTAATCTTTTTTAATTTCACTTTTGACTTACCTATCTCTTTGACCATATTTTAATCTTTAATCAAAGCAACTACTTGACCTTAAATTATTTAAGTTTTACTGCAAATACTAAGATAAAACATCGTCAGCACTCAAAGTAGAATATTTAGAGGCAAGTTTGTCTAGATTCAAAATTCATATAATTTTTCTATAAAACAAGAAAGGTTAGGCCAAAGAGGATACAGCAAGCCAGCAATTACCTTTAATTAGTTACACTCTGTGCTATTGGGAATACTACATACCTGAGACAGGATTGAATGATATGGGAGGAGTTCTATTCAAGTTTCTATATTATCCATATTCTGAATTATTAGTCAACCTTATTGGCTCGAAAGTTAGAMTTYGGAATGTTAAAGCATGTTCTGATTGTCYTCTGAAATAAAMTRGGTAGGATAMTGTTGAAGAMGATTCAAATGATTCCACAAATCTCYRGGTTCTGTAMATTGTTCATCTTAAATTGAATTGGTTTGTACTGCTCTGTAGATTCTTATTCTTTTCATTTTTTAACTTTGGTSGGTCAATTTGGAAATCAGAATTTCrtcggattttttttttTGGCTTACTTTTAGAGTATGCCTATGGGGTGGCGTGGGAACAGTGAActctcactgttggcttcatagcaatcaagagtttcttattaaattttttgattaaatcttaatcaagtgcgagtcactttattgatttaatttttgttctcagatgaatctcctgtgccacaaaggacaaagtgcaacaagccaattttttggctcactaaagaatcattaaatagagttggatcttatgatggagaaggtaca

>Cs1g17370

nnnnnnnnnnnnnnnnnnnnnnnnnnnnnnnnnnnnnnnnnnnnnnnnnnnnnnnnnnnnnnnnnnnnnnnnnnnnnnnnnnnnnnnnnnnnnnnnnnnnnnnnnnnnnnnnnnnnnnnnnnnnnnnnnnnnnnnnnnnnnnnnnnnnnnnnnnnnnnnnnnnnnnnnnnnnnnnnnnnnnnnnnnnnnnnnnnnnnaaccttagttttcgctgaggttctttatttaacaaagaaaaaaactaaataccagagttaatttttaattttttaaaatcatttttaagcctactaaaaataattttaaatgagtcctaagtcaaagatacgagctcagcggaaggcCAAAGTCGTAATAAACTTAATTACGGTATGACGTGTAATTATACTCACCATTTTATATTTTAAATATTAAATTATAAGTATCCTATCTGTATGAATTAATACCTGCACTAAAACAAATTTCATTTCACAAAAAATCTAAGTAATTCGTGTATAAAGCACCTCACATGGCTAGCTAGCATCGCAAAGGTCGCTTAGTGGTCAAGTACAATAATCTCTTCTAATAATTAATTAAAAATTTAAATTTATTAATTGAACAATTAATAATATATTTTAGTGCCACGTTTCATGCCTTAAAATTGATGTCACTGTATACGTCATRCACATTATCCTGAAACTAATAAATTGTAAATATAGGCSACCTTTCTTGGAAAATTGTCTcttatctctttgttatatattttctgatcaacaaaaaaaataaaattgaagttttttttttnnnnnnnnnnnnnnnnnnnnnnnnnnnnnnnnnnnnnnnnnnnnnnnnnnnnnnnnnnnnnnnnnnnnnnnnnnnnnnnnnngatgctgtttatgcattaaccgaggggccatgggttatctttgggcattatttaacagtgcagctgtggacacctcagtttgacagcaccactactgatcttgactctgccattgtttggattaggttacccggca

>Cs1g07190

atattcaaaaaaatgaggcattcccaaaatatcagaccttaggcgagtaccttgtttgcttatacttaaagtcgccaccagagatttctatctctctcatttatctataaaggatgcacattagttttctcagttccctccaatattttatatagaagtgatgagaattaaagtttgggggaaactataaaatactggtgggaaataattatattttccttataaattaaataattacattagacattaataaataaaaactaatttgtaatcaaattgaaaatttaatgctgtatggtattatatgtttataattgtaacattagtagtttannnnnnnnnnnnnnnnnnnnnnntattattattttatttataattataacattataatattatattattcgataaaagtatattattaaattattctaatgttataaaaattaatatatagaaaatttagttannnnnnnnnnnnnnnnnnnnnnnnnnnnnnnnnnnnnnnnnnnnnnnnnnnnnnaaataatattaagttaattttaatattaatatacaattatttataatgcagtctatagtaaaccaaacattgtgttgcattacaatttaatacaatnnnnnnnnnnnnnnnnnnnnnnnnnnnnnnnnnnnnnnnnnagtgcatcaaactcaatcttaaaaaaatctaaaataagaattatcaaaaatattttagttatcaaataatttatttaaatatcacccaaatattgaggagttaaatttcaaaactcgtccagataaaatctaccgtgcagccaacaagaagaacgatttctttaaaatagccgaatttctaaaaatccaaaattgaaaaaccctaaaccattgggagtcgggagccctttaattagtttttaatttttgtgaaactaaACAAGTAAACATCACAAAAACGACAACCAAAATGAAGATTGACCGTCGTTTCATCTCCGTCTCAGTCTCAAATTAATTCAACTCCG

>Cs1g10920

nnnnnnnnnnnnnnnnnnnnnnnnnnnnnnnnnnnnnnnnnnnnnagattatgtaaatgtgtgtttggtaagtaagaaaatcaacgagaaaatgaagaagaatcaatctggaatttattagaattaaaactgtattatacaaaaatgttttaagctcggtatttatacattgtaaagaagctgactagagtttgttataacgcctatacacgcttaatgagtttgttaacaataatgctgactcagctaactaactgttacacgtcagctagaagataatcaagtgtgctcatcctcacagctctgatcatgctctgtaacagtttcggtccagtaatgtttctgcaatttgttctatttttgcaggacgagtagccatggccaaaagttggttccagaacggatctctcaattgatttgagcaccaaagtagatggaaacaaattaaataattaaataatgcattgcgtcaaagagacttattaaagtttatttgtatttaaatatattgtttttccaagttcttctcatttgtaattactacacatcctttgataaaaaaaaaaaaaaaaaaactcgtgaatttgtaaatgttacctttaaataattatttatttgtcttcatattggatttacttttgccttaatagttgccgccaaatattcacacacaaataacaaaggtttcaccgcaatatgtatcctgtgagattttttttcctgtaaatattggttagagtaatataattattcttttttatcatatatatatnnnnnnnnnnnnnnnnnnnnnnnnnnnnnnnnnnnnnnnnnnnnnnnnnnnnnnnnnnnnnnnnnnnnnnnnnnnnnnnnnnnnnnnnnnnnnnnnnnnnnnnnnnnnnnnnnnnnnnnnnnnnnnnnnnnnnnnnnnnnnnnnnnnnnnnnnnnnnnnnnnnnnnnnnnnnnnnnnnnnnnnnnnnnnnnnnnnnnnnnnnnnnnnnnnnnnnnnnnnnnnnnnnnnn

>Cs1g09910

gccttcaaaaaatatagggtcttaggcaccttcatatgttcTTTTTAATATATAAATTATTTACACAAATATTGATCATAAATTATAAATAAGTTCGAGTGATAAAATGTCTTAGGTCTGCCTCTGTCCTCATGGGTTGGCTTAagactttaaacaatgaaattaaacaaaccacgTGGAGGTAGCAAAAATTTAGGCAGAGTTGGCGCATTATATTCTGGAAGCAACCTTATATAGTTGTTTATTTATTTGTAAGATTGTATCCATGGCCCAGTCACATGCCTTTAGATAGCATAtcaacttgaatcaAGTTAATTCAATAAACTTTCTTAATAATATATTAATAGAAGGTGACGGTATATTATTTTAGACTTTAATAAAACTCAGGTGATGAGTTATCTTCTTTAATTCTCACATAATTAACAAGTATTTGGGTAAATCTGCAGTATCGCATGAATTATCTGTAAAAGAATAATAGAAAACATTTGATTAATGATCAGTCTCATTCTCTTTTGAAAAGTCTTAAATCTCAGTTCTTACTTAAATTAAAAAAATCACTCATGATATTAGATTATCTTCTTGTTTATGCATCATACTTTACAGCTTTTTCATCATGTATGAAGGGTTCATAATCACATCTACTTTTTTTGTATTGATAATAAATTTTTTTATCCCACATGTCAATCTTTTGAGGTTTTTGAAGATATAATTATTATAGTTTTcattttaaaaaaaaAATTAGATAGTTCACCCCAAAATGAAATTCTTAAATTTCCTAATTAATATTATTTCAAAATTAATTTTCTCATAAAAAaacagaaaaaaaaagaaaaaCTCTACTCCCTCAATTAATTTTCTCATAAATTTTCCCTCGCACAAGACCCTCTATTTTAGTGGAAAGCTTGCCATGCAAAATAAAAAAATTATATGCATATTGGTTAATGCTTTTAAATCACGTTGAGTTAAGTGGTGAACAAAAGAAAGATGGTTTTGAAGTTTCC

>Cs1g15460

ttttcgattcggataaggattctgcgacaacaaaagacaagtgagtacactttgtgtaatgaaaaaggtgacgtgttaattcccgctcacaagtttatttaattaattaaatatttgtagtaccaattattgggcctggccataggaaatagaggcaaAGCCCATTTCGAATACCGGTGAAGGATTAACGTTTGTAATTATATGTTGTWTGTGAATTAATCTTTCAATGTATAAATGAAATTGGATAATTATTGAAACTCTATAATAATTTTGATTCTTTTTGGGAATAATTTTAAGAAGTCTTAAKAATGGAAAWTTCCTTGTTACGAAAACAATAAAGGGGTTCGTTACTATTTTTGTTTGAATTTCGCAAAATCATAATATATTTTGTAAATATTTTGGTAATAAACGAAAATAATAAGGYGGTTCATTWCTATTTTGTTTGAAGTTTGCAAAATTATAATATATTATATACATGTGGCTAAGTTACAAAATAYTGTATCTTACATCAACCTAAAAATTATGGTTAAAAAYYTAARAGTATTTGACAACTAAACTAAGAGTTTGTTTAGGCAACTGCACTGCCGTATTTGTTTTGTTTACAACAATTCTTATATTGATGTATGTTACATTATTATGTAACTTAGTAAAACCTATTTTATAAATATTTTAGTATTAATAATTTtaccgatctgaacatttgtgttcaacttaaatcctaatattataaataatttatttattatataATAAACATAAATTACTATTCTACCATTAAATAAATAAACACCACATCATTTGATAATTTTCCTAACCGTATCCTTTTTACTAAATCACTTCATTTTTACACATACCATGCTTAAATTACGTAATATAtatatacacacacacaccttgagTGACTATAAACTATGAAGCTTTTTtaaATTTAAATGATTATTAAAATAATAAAGCAAAATGATGActaatataatttTCTATAACAGAAAAGAAAAATGCTA

>Cs1g11240

GACTGAAAATCCacctttagagaaaaaaaAAATGATGGTCATTAAAATGAAYGAGAATTTTTATTGAAGAAAAAACTCTCAAACAAATCGAAGGKSGTACAAAATGCGCCAAACCTCCTATCAAAGCAAAAGCACTAAAGTGGTGAAAACTGAAAATAAAATAGCAAACAATAAATGTAAAccagtannnnnnnnnnnnnnnnnnnnnnnnnnnnnnnnnnnnnnnnnnnnnacatatatataGTGACTGCTGTGTGGAAAGACTTCACGAGTCAAGCAACTCGATAACATAATACTCGCTAGCAGCTCTAACCTTAGGCAATTGGATGATTGCCCAGGCCCCACGTAGAAAATATATTATCTACTRATTTTTAAAGAAGAAGAMTACAACTACTTTTTATCTTAAAAATTGAATAATATCTCACTAAACTGTATCTTTAAAGACCTCATCTTAGTCAAAGTCRCGTTAAAACTGTGCAATAATTACTTCGTAATTTAAAAATCAAACTTTTACCTTGATAGCTATTTTTCCAAAATGAACTTCAATGTAACTATCACACCAATCTTTTCAACTCAACCAAAACCACTTCAAGATAAGTATTTTTTTAAAATTAAAATTAAAATTTTTTACTTAAAaatatgaaaaaaaaaattatgCCTAGACCARAGCGAGCTTATTATTTTTTCATTCAAACATTGTGTGTGTATATTACAATTATAAATTTATAAYAARTATTTTAAAATTAATTTTATTATAATACAATCTAAATTTACATCCTCACAAATATTCGAAAAATATTTATTTTACCTACATTAATGAAAGGAAAATTTATTCCTAACCAGTTATATAATTAAAAGAGGAGTAGAGTCTCAACATCTAGCcactTAATTTGAGCgcatGGTCCAACCTTGTCGTTCGGCTGCCCTTGAAAGCTAARATATAAATAATATATTTAATCATGTGCGTCYCAGTTCACACGGCCATAAACTTTGGAAAAGTC

>Cs1g26550

TGGGTTATTATTATATGATATATATCATAATTTGATTCACATACAAAAATGCAAGAATGCCGCAtactttggtggaagatataaaaattactcaaaatttaatatttgagtaTcAAtaaggtaacggctatgttctctaaaacacctaggggcctcggccaccttttgcattgcatttaatcttgctgctgcttttgccccgttgcgcgttagggaatgtttgggattgccgtcagaagcactcaccactgcttcattagctgatcacgtaaccgcagcactgtttcccacctttaaaatcccagtcattttcatttttttttttttttggcttagttggtgttgctttaagaaaaatatcgttacTTCCAAGTAATTGATTACTTTCGTTTTGAAGGTTGGGTTATTATTATATGATATATATCATAATTTGATTCACATACAAAAATGCAAGAATGCCGCatactttggtggaagatataaaaattactcaaaatttaatatttgagtatcaataattttttttttttttttttttttttttttttttgggggggggggggggggggggtttggtatggtttggTTTTctatataaaaaaggaaATATAATTGAAACgaaacaattaaattaAATATGGTAATCAAGAAAATTCCCGGTAGAAAAATAAGGATTAAAATATTGATATTTCCAAAAACGGTTAATGGCTTCAATGTTCACGGTTTATGACCGTTAAGTTAAACGAGACTATTTATTACATTTTGAAGAACGAGAGGCCcccaaccacgagagagaaaggaAGATAAATTGAAGAAGAAGAAGAAGAGGAGAAGACAAAAAACAGagcaggagagctaatgCTAAAATACGCAGGAGGGGACACACAACTCCACAACCCCGGCCCATaataataataatcgatcgannnnnnnnnnnnnnnnnnnnnnnnnnnnnnnnnnnnnnnnnnnnnnnnnnnnnnnnnnnnnnnnnnnnnnnnnnn

>Cs1g16360

tnnnnnnnnnnnnnnnnnnnnnnnnnnnnnnnnnnnnnnnnnnnnnnnnnnnnnnnnnnnnnnnnnnnnnnnnnnnnnnnnnnnnnnnnnnnnnnnnnnnnnnnnnnnnnnnnnnnnnnnnnnnnnnnnnnnnnnnnnnnnnnnnnnnnnnnnnnnnnnnnnnnnnnnnnnnnnnnnnnnnnnnnnnnnnnnnnnnnngagtcatgttgatcctatagatgctagttgtgagaaagccatgttgtcttgtatatggaacatttttaagttgcaaaaaaagttctcatatttgttgcttctatattgcgtgatcatctcgacaattgataataaattatagcaatgataataaatttgaagtattatttttattacaaactaaaattgatgatctagatacacattagaagtttagactatgggctagctcttggaacattttagaaagatatggtttttcattatggtgacaatttaactagaattcattactttgcattttctcccaagataaagaatcatttactttgtgtatattgggttatggttgtgtaatgaattctagcctccaatgcagtcatgtaaagaattttttacccgacttctaccggactgtagccgtgtaaagaattctttactcgacaacagttgtgtaaagaattctttacacaactgcagttgtgtgaagaatactttgctcaatagtagtcgggtatgaacacctcactctactatattcgtgtattagttctttactcgactgcagtgtgtaaagaattattcacttgacatgcgtcgggtatgaacactttattttcaatgttacaaagaatctatggtttatatagtggattgtagttcaatgcatgctttactcgactgtagtcgtgcaatgaatgatttacttgactggtatgtcgtgtaatttttatgattggtttaaaatatattattatgtaacttttcaatcattttatatattgattgataaattttttttt

>Cs1g15170

gttatggggtttataggaaaaaccaaatatacccatcctcatatggcttaataaacattgctttaaaacccaaagctttttgaataatttgcagaaaagtaaagtcgagtatccagaaagaatttctccgtgagatcaagagaaaaaagagacccccttggcgtggttttctcggggtttttataaacaccccacacccacgaccttagatcgtgccaataaccattaattgatcaaattcgtgccctgatttttcggaggagctttgtggtggtttattttaattgagtctttattcctggttatcacctggtcgaggtgatctgctcaagtttagaccacctggtcgaggtgatctggtcaagctcatagcacatggtcgaggtgatctgctcgagctcatagcacctgatcgaggtgatctgctccacctcatagcacctggtcgaggtgatctgctccacctcannnnnnnnnnnnnaggtgattaTTGTGATTAGCCACTTCGGTATTTCTCTTcACTATTTTTCTCACATTTCACATTATTTATATAAATATTCGTATTTTAAATAATGAATCCACATCTTAATTAATTAATGATCAATATAATTGAAAATYGATWAACCTTGAATACTAAAGATatactctgacttATTGTCATTTTGTWTTGAATGTAAGTATTAGCYYTATCACTTTAATCCAAWATCWTAAGTCCAGCTCAATACATMTAACCAGACAAGTTCAAAAGATCGCCTAAAACTCTAAAAGGTTTCTTTTATATTTATTGTTATTTTACTATTCTAGCTAGTGATTTTACTTTTCTCCTGAGCCATTTTWTATATCCAAGWGGGACATTTCAYAAAACGTCACTGAACCACAACAATTTGTTASAATATCAAAGAKGGATAGGCTTATCCAGCTCTCGTCCACATGTCACCGCCTAAATGGGATTCCATATCTCTCAAAACAAAACCCAGTTCGCCACGTGTCCCGCTGACATC

>Cs1g10440

nnnnnnnnnnnnnnnnnnnnnnnnnnnnnnnnnnnnnnnnnnnnnnnnnnnnnnnnnnnnnnnnnnnnnnnnnnnnnnnnnnnnnnnnnnnnnnnnnnnnnnnnnnnnnnnnnnnnnnnnnnnnnnnnnnnnnnnnnnnnnnnnnnnnnnnnnnnnnnnnnnnnnnnnnnnnnnnnnnnnnnnnnnnnnnnnnnnnnnnnnnnnnnnnnnnnnnnnnnnnnnnnnnnnnnnnnnnnnnnnnnnnnnnnnnnnnnnnnnnnnnnnnnnnnnnnnnnnnnnnnnnnnnnnnnnnnnnnnnnnnnnnnnnnnnnnnnnnnnnnnnnnnnnnnnnnnnnnnnnnnnnnnnnnnnnnnnnnnnnnnnnnnnnnnnnnnnnnnnnnnnnnnnnnnnnnnnnnnnnnnnnnnnnnnnnnnnnnnnnnnnnnnnnnnnnnnnnnnnnnnnnnnnnnnnnnnnnnnnnnnnnnnnnnnnnnnnnnnnnnnnnnnnnnnnnnnnnnnnnnnnnnnnnnnnnnnnnnnnnnnnnnnnnnnnnnnnnnnnnnnnnnnnnnnnnnnnnnnnnnnnnnnnnnnnnnnnnnnnnnnnnnnnnnnnnnnnnnnnnnnnnnnnnnnnnnnnnnnnnnnnnnnnnnnnnnnnnnnnnnnnnnnnnnnnnnnnnnnnnnnnnnnnnnnnnnnnnnnnnnnnnnnnnnnnnnnnnnnnnnnnnnnnnnnnnnnnnnnnnnnnnnnnnnnnnnnnnnnnnnnnnnnnnnnnnnnnnnnnnnnnnnnnnnnnnnnnnnnnnnnnnnnnnnnnnnnnnnnnnnnnnnnnnnnnnnnnnnnnnnnnnnnnnnnnnnnnnnnnnnnnnnnnnnnnnnnnnnnnnnnnnnnnnnnnnnnnnnnnnnnnnnnnnnnnnnnnnnnnnnnnnnnnnnnnnnnnnnnnnnnnnnnnnnnnnnnnnnnnnnnnnnnnnnnnnnnnnnnnnnnnnnnnnnnnnnnnnnnnnnnnnnnnnnnnnnnnnnnnnnn

>Cs1g02650

GATTCTGTTAAAGAAAagggacccctacCGATTGTTGTTAGTATATTCTCTTTACAATAAGGAGGATTTTGATTGTTTTGTCTTTTTCCACATTTTGTTGTTAGTAGAACTTTTATCACTACAGTTTTTATTTAAAAATTTTAATTGTTTGATTGTGAATTTTTAAAACTTATATTATAAAAAAtaaaataaannnnnnnnnnnnnnnnnnnnnnnnnnnnnnnnnnnnnnnntttattagtagaggtaaaataatttatttaatttttaaaagttctattaaaaaaataattaaataataataaaaattttattaagagtttttaaaannnnnnnnnnnnttaggctctatttggtatatcttttcaagtagagtatatttaagtaaagtttttattagtagagtttttacaagtaaaatttttatttaaaaatttttagttgtttgattgttaataacaacttttattaaaaattataannnnnnnnnnnnnnnnnnnnnnnnnnnnnnnnnnnnnnnnnnnnnnnnnnnnnnnnnnnnnnnnnnnnnnnnnnnnnnnnnnnnnnnnnnnnnnnnnnnnnnnnnnnnnnnnnnnnnnnnnnnnnnnnnnnnnnnnnnnnnnnnnnnnnnnnnnnnnnnnnnnnnnnnnnnnnnnnnnnnnnnnnnnnnnnnnnnnnnnnnnnnnnnnnnnnnnnnntttcataatatcttatagaattaaataaacacttatgactattaaataaatacttataattATTAGATAAGACATACTAAATAGGTAATTAACGTTAAATATTAAAATTACTTAATAATGAATGGTATTCTTAGAATATAATGGGTTGGTTGATGAGTGGTCGACAGGTTAAAAGTTTGATTAAATAATAACAAAAATTTTGATAAGCATTTATAAAATTAAATAAGTACTTATTAGTATTAAATAAATGACGTCAAAAACTATTAAATAAGCCATAAAAAAGGGTAGGTGATTACGTCAAAT

>Cs1g09256

GCTATAGTCAAGACGAAATTGAAAGTGGTTTGAAATGGGAGAATGGTCAGAGAGAGAACCTAAgagaagaatttttttttttccaaaAGTATTAACAAATGAAGATTATTAAGAAAGTACAACAAATGAATGTTAGTTTAAGAATTGATAAGAATATATACGAAAAGGGGTTTGAAAAGAGGCCAGTGCTATGTTACGTTAAACGTAACATACACGCACTCACAACAACATTTCTAATGGTGGGGCCCGCTACTGTTGCTGTGAGTGTGTCTACGTTACATTTAACgtaacatagggtgatcttagaaaagaatgttagaggtgtgctaataattcaactcttttttatttttatttttggccacgtaaatgcgttattaaactatatagtttaatagaGTTTAGAGTTGGAAAAAATGCTTGGGCCtaccctccacatgagGTCTTGGATAAGGGTTTAGGAAAAAAAGTATGGGTTAGATTTGATCCTCTATTTAAAATTTAAAATAATAAATTAATTTCTAAAAATAATAGAAAAATAAAACTTAAATTCTCAATTTATAAATGAATGATGATTCAACCTAAAGTATAATACTAAACTCTTTCTTAAGCCTTAATTAATTAAAAGGGTGCTTGAATTAGAACATTCAGTYCCAAGAAATTAAATATGTAAGCTTTTATTTACTTCATTTTTTATTTATTATTTTATTTTAAAATAATTATTTTCATTAATTCATTATTAATTATAACAATTTGATGATTATTAACTTATATAAATTTACAAACCCCAGCTTAAGCCGCAATTTGTTATCTTTAATAYAGTTAGTCCAGACACACTACACTAACTCTATCAAAtcataCTAATAATGTATTCGCATGaaaaaaaaaAATTGAGMGAGCTCgcacttaagaattTTTATATTATTATAAACGACTATTTAAGAAATCTACTAATGATATTTGTAGCCGTGTACACGAGCCACTCTCATAATTGCTTGAAT

>Cs1g21960

cccaacccaatccgtaaaaaactcatacgggtcgggtcggtttcacagattgagcgagttgattcccaccctgtTATTTTTATGAATAAAGAGATTCCGTCGCAAATTTCATTTCTTTATCAGAAGCAGGCCGTAGACAAGAGAAATGAAAAGGCAAGGTGCTCTGGTTCATTCTTATTGCAGTATCCATCCCCTTTGTGAATTTTCCATATACATTATGGCCACATACCAGCCATTCCACGCACTCTTTTCCCTTGGTCGTATGTTCCCCCTTGTACAGTGAACAGGTAACAAAAGAYTGGGTCTTTTGACAGCCATCAAAACTCTTTATAGAGCCCCTTTCTAATTGAAATTAACTAAATAACTAAATATAATTTaattcatttttttgttttTTGAATGCAAATTTAATTAAAAAACCTAACTAAATTATTATTTTAATTAAAATTTTACACCCACCCTATTTCATAATTTATAATTATATTTTTATAATATTTTAAAAAATAATGAGATTTTagannnnnnnnnnnnnnnnaactacataataagcataaaaattTTAACAATATTGGGATAAAAATTTTATGGAAAAATGACTTATTTTAATATATGGAATTTTATATTATAAAATTAAAATGGGATATTTTGAGAAATAAAATGTAAAAGGGGGTATTAGGTAGATTTTAGAGGGGTGTTAAAAGCTCCTCTTTAACAGGAAGCCTTTCTCTTCTATAAAGGCTGGCCCATGAACCATGATACAGTGACATGGGCCACAAGCCTCACCTCCCAATCTCGCATGCCATCAGCACAATCTCCTCGTCTCCTGGCACTAGGATGTgtcgcgCTAGGatgttgacatttcaAAAGTGACGTCCTCGTTCGTCATCGCAACACCAGTCCAGCTTGAAATSGTTTCAGTGAGGATCATCACATCATCATCAGCCTCAAGAGTTGCTTTTTACTGCCAAAATTCGAAATTAAACAAAAGAACAACAAATAAA

>Cs1g23970

ATATACTTCCACAACATATTGGTGCTAAAAGTAGGCTCTGCTGTTGAGCTATATATATTTTAGTTTCGAAAATGAGCTGGGTTGTAAGACTGTTGTATTTTTTCCACGAGTTTTCGGTTTTGTACGTTGGAGATAGATCAGTAAGATCCTATAGAATAAGCTTTTAATCGGTTTGTTTGAGGTTGTCTTTGTTACAAGCGTAAAGCCCAGCGTTCACAGCGGAGAGAACATCTATTTTTGAAGGTATTGTTTGCCCACTCCTCTTTGAAAAAGCTAAACTGATAGTAACAATAATTAAAGGTGGTGATTGGACGCAATGAGATAGGAAATGGTAAAAAGAATAACCAACTTAAACTAAACTAATGTTGGCAAAGACAGAAGTttgacacagagagagagaGAGAGAAAGAGGGAGAAAGAAGCCCTGCTTTCTCGCGAAGCCAGAGGACAATGACAGGTTTCCATTAGCTAATCTAGATTAGGAATTTCACCAAGAAAAGCCCTTTCTCTCTCTTCCCCTTTTGAGAACCCTTCAATCTTCTCCATTCAAGGTATTKTTTTTCCCTTtttttcccttttttTTGAATTTTCTTCTCGGATGTCGGAATTCTTTAGCCATGCATATGCTGTAACAAAATAATCTTGTAATTTTTGACMGTGTTTTAATTTGGGACATCATTATTAGTTTAACAGATATAATTTATTAAGGCAAGAGGAAAACGGTTTGGCATTTGTTGATGATGGTGTTGCAGCAGGGAGAACAAAAAACAATAATAATTTATGTTATCTAGCTTTGTCATACTGGAAAAAATTTTATATGTCTACAGGCTAGTTAATTTCATTCTACATTACATGAATTGATTGATTATTCATTCAATGGTTCACAGTCTTTGAATCTAATCAATCTCTTCAGGGTCCTCGAAAAGACTTTTGAAACTGGCAGTTAATTAGCTAGGGAAGAGGTGGAGTTTCTTGGGGTGGAAGTTCTTTAGCTTAAACAA

>Cs1g09860

tacttcctaaaaaagtgcaacataggtcacattctgacatnnnnnnnnnnnnnnnnnnntgtgcctacttctttagatcaagttccatcacaagactcatcaccaaatgatccagaagagagtggcccgccagcttacattcccaaggctccttttcctcaaaggttaacaaaggtaaagaaagggacttcaacaggtgnnnnnnnnnnnnnnnnnnnnnnnnnnnnnnnnnnnnnnnnnnnnnnnnnnnnnnnnnnnnnnnnnnnnnnnnnnnnnnnnnnnnnnnnnnnnnnnnnnnnnnnnnnnnnnnnnnnnnnnnnnnnnnnnnnnnnnnnnnnnnnnnnnnnnnnnnnnnnnnnnnnnnnnnnnnnnnnnnnnnnnnnnnnnnnnnnnnnnnnnnnnnnnnnnnnnnnnnnnnnnnnnnnnnnnnnnnnnnnnnnnnnnnnnnnnnnnnnnnnnnnnnnnnnnnnnnnnnnnnnnnnnnnnnnnnnnnnnnnnnnnnnnnnnnnnnnnnnnnnnnnnnnnnnnnnnnnnnnnnnnnnnnnnnnnnnnnnnnnnnnnnnnnnnnnnnnnnnnnnnnnnnnnnnnnnnnnnnnnnnnnnnnnnnnnnnnnnnnnnnnnnnnnnnnnnnnnnnnnnnnnnnnnnnnnnnnnnnnnnnnnnnnnnnnnnnnnnnnnnnnnnnnnnnnnnnnnnnnnnnnnnnnnnnnnnnnnnnnnnnnnnnnnnnnnnnnnnnnnnnnnnnnnnnnnnnnnnnnnnnnnnnnnnnnnnnnnnnnnnnnnnnnnnnnnnnnnnnnnnnnnnnnnnnnnnnnnnnnnnnnnnnnnnnnnnnnnnnnnnnnnnnnnnnnnnnnnnnnnnnnnnnnnnnnnnnnatactcagcatgtacaggatcctaagaaacacaccccagttattctaggtcgtcctttcttagctacagctgatgctcatattagttgcaggactggaaacatgcaattgtcttttggtaacatgacca

>Cs1g19010

agatgtgttgtagatattctatattgttaatctataactccaatactcataattgctcccaccaagataccgggtaatcttgactacaagtgtgtgtcatgcccattggtaactcaaatggaatatcaattacaatcatgaaatcataactaactcaagattaagattacagtaaaatcaatgcctatgagatttaataagtctgacagttattacaaagttaattaaatatcatatgtaatcatgttcaatgtagtcatactacattaataaattcatacatgattaaGaCAAaTCATTCAATGAATTTATTACAGACTAAaCATAaATAGAGCGCCCAACTCTATTTATCAACTGCGACCTTAATTTATTTAATCATAaGaTaactTGTAtAaCTTATATTTATGTCTTCTGTGAATCCATATGATGATCACATAAATACATATAATATGATTAAACGGACCTTATTCAAAATATTTATGCAATTGAAAATATTTCAAATATTTTATTAATCAGAAAAtaaataaattaataTAMTTTAAAGAGCATATAATCCCAACWGTATGAAATTGTATGAAATATAAAATTAATTTTAAAAATGAAAATTTTGAAAAARTACTTTTTCCTTTTCAAAATTTCTTGTAAGATAAGATAATTTTACCAAAAGTGATATATGAAAAAATTTATCAAATATTAAAATTAACTTTAAACTTTTTAAAATTATTTTTGAGTCTCTTCAAAGTAATTCTGAATAGGCCCTTAATTGATTGACTTGATAAGATAATTATTATTAATTTTTTTAGTATTTATTCTATATTTTATTTTGCTAGTRAACAACTTCTTATACCCAGTACATATTTGASAGTGGTTATCTATTTATCATATATATTTCAAAACAACAATAAAAATTATTAAAAACATGACAACCACACTCTTGATATAGAACTATTTTGACTTATATTTTTCAACTTACAGTTCAATTCCTACAGTACCGATATTCA

>Cs1g21340

tcttAGTAAAATATTTTATATATTTTAAACCAGGCTCATTCGAAAAATAAAGTGATCAGAAATTATTCGAGAATATTATATTATTTTATGAAAATGGGCTCCTAGAGTGTCGCTTGCACATGCCTACTTATAGTGGGCAAATCTTCTTTGTTCCTGcctagagatggcaattgtacccgcaaacccgcaaacccgcccaaacccacccgccaaaacccgcccgttgcgggtaattttacccgttgcgggcgggtttagtattataaattaaaacccgcacatggatgcgggtgggtttggtattaaccttgtnnnnatgtggcccatctcttattctaaagtcataacctaaagaaaactaaaggcatttcccttcgaattagtatttgaaaatattaatcttaattattattataggcattgtgttggatgggtgcttatggtggtgaatgaaatgaatatnnnnnnnnnnnnnnnnnnnnnnnnnnnnnnnnnnnnnnnnnnnnnnnnnnnnnnnnnnnnnnnnnnnnnnnnnnnnnnnnnnnnnnnnnnnnnnnnnnnnnnnnnnnnnnnnnnnnnnnnnnnnnnnnnnnnnnnnnnnnnnnnnnnnnnnnnnnnnnnnnnnnnnnnnnnnnnngctatatggaatttgaggttattgttataaatttatatattaaatatttgtgattttaaatacagaaacccgcaaaaaatctgtcggataccattgcgggtttggtaattgttaaaacccgctgcgggtgggtttttttaaaaaaattaaaacccactgcaggttgcgggtgaatttaataatttaaattttgtgtgggtttgagtttagtaatgacaaacccactacgggcggtgcccattgccatccctaTTCCTGCCTGGACTCGAAGGCAAATCCAATTTGGTCGGGCCACACGTTTCCAACATGTCACAAAACGTTGTCGTCTCCTaagtccgcacaatccgcTGTCAGACACACACGA

>Cs1g16500

ATGCCCAACGGCAGTAATCCTTCAAATATTATCRACGACATGATTTGGGTTCTTGAAATTTTGATTTGAGTATATTTTTATctctatgtttttttgtttgATCTGTGCATTGGGGGTTCCAACAATGCACCAGTAGTTGGTGGTTGTTTTTGCAAGGGGAATCCGTCGTGGTTTTYGTGTGTTTAGTTCAAATGACAATTTTAACCCTCTAATGTCAGCAATATTTTAACAAGGTTATGATGGAAGTGGAACAATGATAATTTTATTAACTTCAAGTAGATTGTTGTAAtatatatatATaTATATATTTTTTATAAAATGTCAAAATCaannnnnnnnnnnnnnnnnnnnnnnnnnnnnnnnnnnnnnnnnnnnnnnnnnnnnnnntaaaattttatttcatcacttttttttttattacatgagactattgctcagattacaactcatattacatgagaatataactgatatgtacaaatcagactataactgggaccaacatacatcaaatctaatggagcactgcccagattttaaccctcttaaatattcgagggatgtctactcctcgcactggggcaaggaagcagactgccttcactcaattttatttgaggaaggaaacacccccacatgcattgtgggggttcnnnnnnnnnnnnnnnnnnnnnnnnnnnnnncttcttaaagtatattttatgcaaacataatttatttatttatatcgaaaaccctgttcaaatgactaaaatgtcctttcGGTTGCCTTATTAGTTAATAGTTATTATACANCGNATTCTCTTGGATTGTGATGGTCAAAATATAATATTAAAAGGATGTTATGGTAAATTTAGAGTTTTAGTGAGGTTGGGTGGGGATCTATTTGATACAAGCAAAAAAACTATATCTTTTTTCAGGAAACAATAAAAAATRAGGGATTTCGAAGTTATTATCCAATAGTATTTTAATTGATAAAAAATATTTTCTCTGAATTAACG

>Cs1g25310

ggaggtatttatatttattacaacttaatcacaatgataaattaacctaactaataaacaaataataaccacacatcaaaactaaacaggcagatgtaggaaaggttgggccaccttcctggagcattttagcgggggcaggggctcatgcaaccaaacattaatgaattcattagcattcctcttcgggttcattggagtctcaggtaaggcctagagaagggagaggaagcagcgctctgaactcattgattcagagacaatatttggtaaattgcagtgcttgccgaaggcgaggtatgcttttaggtacttaccacATTACGACAGTTAGCACCACAAATTTTAACTACTTAAAGAAGCTTCCATGAGCTAGATTAAAGACCATTTAGGCTTTAAAGTTGTATGAATCAAGTTTATGTTCCAACAAAAATCCAGTTTGGGTCTAAGATCGATGCCTAATCCTACCGTGAAGAAAGTAAACCGTCTATATGGTAAATCTTTATTTCAATTATATCATAGTTATAGAAGTCCACGAGCTTATTTCATATTAAAAAACTAGATAAAAAATTGAGAGTTaGTCTCAAATTTacattattagtctaaaatcaattctgagctcgtGTggttattccttatCAATAGTGAATTTACCATTGTTGTGTGACTTCTAACTTCACCACCGTTGGTGTCTATGGATAATTTTTTATTTTTCAAATTTTATAATATAATTCTAATGCAATCACTtaaataaTTCTAATGCAAtcaagatcaccGTTACGTTTTTAAATTCTATTTTACTAGAATTTTTGCATCCATGGAGTTTTCTAACAATTTTTCAAATACCAAATACATAAGTCACTTAAAGAGCAATAATTCTAAGGTAATAATGCAKTTACTTTCCCTTGTAGCGGAAAAAAAAAATGGTCGAGTATCCTTTcccttttacaacnnnnnnnnnnnnnnnnnnnttgagaaaacatttggggttGGTGAAATAC

>Cs1g09210

atctcgcctcccacctgaagtgtctcctgaaatagtctccgtagatctttccccaattctggaagtttcaccttctaccattttctcaacgaactatttccacttgaaaaactgtctctatgatgttaatttcacgtgaataaatacaccaggaaagttccccggagactggacgggaactcggtcccaattaaaaaccagaatccttagactcttatcgtatacgtgaacagtaccatatcccccaagtacgaaccgtcggctctgataccacttgttgtgtgcggaatgcggaatcaagcgcaccaaataattatagaaaaataaaggacacaagaattatgtagttcggtaattaaacctacatccacagaggcaagaaagaataattgtttatttaacaattaatagagtacaatatttgagtaacgaatctcacttccaaaaacccaaatatacccagtactctcacactctacaagaaagataaatttcccagacacacttctctcacttctctcaaaagcttannnnnnnnnnnnnnnnnnnnnnnnnnnnnnnnnnnnnnnnnnnnnnnnnnnnnnnnnnnnnnnnnnnnnnnnnnnnnnnnnnnnnnnnnnnnnnnnnnnnnnnnnnnnnnnnnnnnnnnnnnnnnnnnnnnnnnnnnnnnnnnnnnnnnnnnnnnnnnnnnnnnnnnnnnnnnnnnnnnnnnnnnnnnnnnnnnnnnnnnnnnnnnnnnnnnnnnnnnnnnnnnnnnnnnnnnnnnnnnnnnnnnnnnnnnnnnnnnnnnnnnnnnnnnnnnnnnnnnnnnttcaacacttagagtgttgattccagtggtagtgtcgtgtttgatattatcctcatgccttactattatttatgctcgaagaatgagatttgctcataactctatggatacatcaccgatggagaaataatttcttatggtttcttatgcaaagctaagtaaggcaactagtgaatttgcatcgtcaaac

>Cs1g15310

CACTCCTTTCATTTAAAATTTGTTTATAAGTCTTAAAAGGACAAAAGTGCCCttcttgacgaatgtctgacgaaaaaaaaaataaaaaaaaGTTACAGAAAATTCATGAATTGTCTTGTATGTTCAGTATCTAAGGTATGAACATCCCTTGCACCTTGACCTCTGGTCGGGTTATCGCTTTAGCTTTGATGCATAGAGTGCAAATGGGTGTGATATTTGTTCATGTGTTTTACTTGCAATTGGCTGGAAAGAATAAATCACTGGAAGATATACGGGATGCAGATCCTAggCTTgtatagtagttgcaggcagatcttggagacggatgctgagttcattgattcacaaatggcttggatgtgnnnnnnnnnnnnnnnnnnnnnnnnnnnnnnnnnnnnnnnnnnnnnnnnnnnnnnnnnnnnnnnnnnnnnnnnnnnnnnnnnnnnnnnnnnnnnnnnnnnnnnnnnnnnnnnnnnnnnnnnnnnnnnnnnnnnnnnnnnnnnnnnnnnnnnnnnnnnnnnnnnnnnnnnnnnnnnnnnnnnnnnnnnnnnnnnnnnnnnnnnnnnnnnnnnnnnnnnnnnnnnnnnnnnnnnnnnnnnnnnnnnnnnnnnnnnnnnnnnnnnnnnnnnnnnnnnnnnnnnnnnnnnnnnnnnnnnnnnnnnnnnnnnnnnnnnnnnnnnnnnnnnnnnnnnnnnnnnnnnnnnnnnnnnnnnnnnnnnnnnnnnnnnnnnnnnnnnnnnnnnnnnnnnnnnnnnnnnnnnnnnnnnnnnnnnnnnnnnnnnnnnnnnnnnnnnnnnnnnnnnnnnnnnnnnnnnnnnnnnnnnnnnnnnnnnnnnnnnnnnnnnnnatcattttaaaaactttaaattgggagctggctaagttacagattctgtaacatacagatccaacttagccagctcctgattggttagctagctaccaaacaaaaataattgcatactttttcttcatatgcaggacaggtgtggtgtggtgca

>Cs1g16390

TTTTGTTTGAATATTAATAAAAAGAAAATAATTAAATTACCAATTtaacctaaaaaaaaTAAtattaatatttaattgtcaaaaattgtatgcaaactaccaactgtgcaaatggtgcttgtaaaaaaattaatataatttttttattttctagaattaaattagtaatttaattattttctttttattaatgtctaagcaaaaaaaattattataaaaggttgatattgtaaaaataagctaaaagaaataaaaaataacgatatgggtatcaatactttaatgacagannnnnnnnnnnnnnnnnnnnnnnnnnnnnnnnnnnnnnnnnnnnnnnnnnnnnnnnnnnnnnnnnnnnnnnnnnnnnnnnnnnnnnnnnnnnnnnnnnnnnnnnnnnnnnnnnnnnnnnnnnnnnnnnnnnnnnnnnnnnnnnnnnnnnnnnnnnnnnnnnnnnnnnnnnnnnnnnnnnnnnnnnnnnnnnnnnnnnnnnnnnnnnnnnnnnnnnnnnnnnnnnnnnnnnnnnnnnnnnnnnnnnnnnnnnnnnnnnnnnnnnnnnactcctgcttcttttccgtatTTCCACTGTTGCCCTTTGTTCTAGTAATTTTATTCCCTACGTGTTACGGATCAGATCTCCACGTGGCATTCGTTCCCATGGTTCTTATGCCACGTGTTGstcccarcaaaatCTATGTTTGCTCATTGATACGTGAACCTTTCGGATTCCTAAACGTGTCGRCTCCAAAAGACSAAATCGGACACGTATATACTTGAACTGGARCTTGATTTGTGGATAAACAAGGGATATTATTGTAAATCAAARTTTCCTCNGTCGCCAAATAGGTACCATGATCTTGTTATACACACCTGTGGGACCAGATATAACCACGTGGACCAATGACAGAGCCGTTTTAGATGGAGAGCTCACAGCCACTAAACTGATTAACAATTTtcagccacaAGCGACAGCTTTGACTGCATTTGATCCTATAAAAGATTGA

>Cs1g26450

AGTGCTTGTTTGTTAATTCGCGCTTTCAAACAGCAAGAGATTCCATGCATGTCTTTTGACGAGGTTTAGTTGTTCAGTTTCTTGCATGGCCCGTCTCTTTTCATTACATGGATTCAAAAAACTtactcttacgagttacgtcattaTCAATCATCTCAGGACTCGATGATGATGGTGCGTCATCATCAAGCAGATGCAGTCACATGTAACTCCAGAAAGGCCAACGGCTTGTTACCTTAAAAAGTGGCAAAAGTTTCAGGTCCACGGTGGCCGAAGCGGCCGGGGCATCTACGTTGAGSGTGAGCCGCCCCAACTTCACGCTCTTATAKATGAATCTTGCTCCTGACCTGACCGCATCTGCTATATCTGGCCRTCTGGGGTTGAAYTAAGGAACCGATTGTATGCTAACTAATGGACGACGTTTTTATTTGAACTCCCCTRCTCATGAGTATTGCCTGATTAATTTATTAACAAATTAAATATTATTTTGTTTTTATGATCAATCACTTCTTACAAAATTTACTTACTTGTTCGATCATCATACATATTATTAGATCAAATTACTAAAAAAATTTATACATTTGTTGTTTGCacatatwtgymactwctttgtcaCTTCTAACTATAATTAATGTTGGTTTsgtggccactaatAtatgtaatattatTTTATTATTCTTGATGTTTGTCATCAGTtgtactgtgtactgctACCACGTACCAGCAGTGAAGGTTAATTTACTGTCTGTTTTGTGTAGGCCACGTCTCTCgtTCGTCAGATAAAAYACGCTTCATTTTTGCTCTATATACGATTAATCATAATATATGYTTTCGATTAATATAATAAAATTACTATCACRTTTTTATATAGACGCACACTAAAAATAACAAGCAGTAAATGGGTGCCACGTATAAATTTCTGAACCCTTGACGCGGGCAAAGTCGTCCTGAAAGGTTTCAGTAAATCGGTTGAAAAATAATATTCCGCCCAAAGTTCTCGT

>Cs1g11990

gaatcgaattttgcggattaaacatcaatccatatctgatccacgattttacagattgtaaatttttaattcaatccatgcatttgtggatttgatgcggatttgacccaatccatattcaatccaccattttgcggattggtttgcaggctaaaaatatttaatttcgtcctatccacgaacaaactaactaaaagaaaattaatataaaaattatttttctactgatcaaattcaagataaannnnnnnnnnnnnnnnnnnnnnnnnnnnnnnnnnnnnnnnnnnnnattcagaaatttcaaaatatagcacaccaaaaagaaaaaaatctcatttttgtagatcagtacatgaaaaataactgtttaggaaattatatttgtttatttaattatttttattttagattAtTATCAGATAAGATATAATATGATGTTAATGCAACTATATTCTAGCttatttatttattagtatgtactaatgtcatattcacaagtttaaTTTTTTattaaataaattattTTTTTtgcggattcgcggatttacaaaagtaaatatccagtccaccgactgcagatttctaaatttttaatctaatctaatccatcaatccatgGATCGAATTTGTACGGATCAAATATATTGAGCAGATCGGATTGTATTCTGAACACCTCTAATATTTTGGTCATTAGTTaaaaccaaaaaaaaaaagtaatTTTAACGGTGATTTTAtcactgtttttttttttttcacccTCTTGTTGAAAGCACGGGTTCTAATTTGTATTGTAGCTGCTTCTAACTCATTCCCTTGAAAAATTAAAATGTCTCTTAGTCCAACAACATGTTACGAATCTTCCCTGTGCTTTCAAAGTCCATGTGAATAATCAGCACATGTTTACGACTCTTCCCTGCTGACCTGAAGTCATCCTTGGCTTTTTCCTAAATGCATGAATTatacacgtcgcaACATTTGGAATAATCATTTGATCAATATCA

>Cs1g06440

nnnnnnnnnnnnnnnnnnnnnnnnnnnnnnnnnnnnnnnnnnnnnnnnnnnnnnnnnnnnnnnttacttcagatgccttttaatcgtaatttattattataactttgtacctttattcattaatcattcccatgaatttttttacctacaaaaaattcatgttatgtggaatattattccaattttagatgggtcaaactccccaccctatcattctggtgcacttgannnnnnnnnnnnnnnnnnnnnnnnnnnnnnnnnnnnnnnnnnnnnnnnnnnnnnnnnnnnnnnnnnnnnnnnnnnnnnnnnnnnnnnnnnnnnnnnnnnnnnnnnnnnnnnnnnnnnnnnnnnnnnnnnnnnnnnnnnnnnnnnnnnnnnnnnnnnnnnnnnnnnnnnnnnnnnnnnnnnnnnnnnnnnnnnnnnnnnnnnnnnnnnnnnnnnnnnnnnnnnnnnnnnnnnnnnnnnnnnnnnnnnnnnnnnnnnnnnnnnnnnnnnnnnnnnnnnnnnnnnnnnnnnnnnnnnnnnnnnnnnnnnnnnnnnnnnnnnnnnnnnnnnnnnnnnnnnnnnnnnnnnnnnnnnnnnnnnnnnnnnnnnnnnnnnnnnnnttatctaaatttttacttttattaatatctaaagaatatccaccaTCACTAATTATACAATATAATTAAAAAAAATTACCCCACAATacttttatttgtggggcagtagccctgattagtaattaattatacacaaaattggaccacatgcgctcatgcgcacacattttatcatggtaaggcagagctcgtataatataaatttacaagtacaaaaaattcatgtgacgttgaaaaaattgattaaaaaaaaaaATTcatttcatttcgtgccctcagtttgcttCGCGTTGCGTACAGAAAGTTTGTACAAaatattttgtgtgtcagaCCGTCCAACCCTGCTCTTTTATTGTTGTAAATGGCAGTGATGCAATTTTCATATATTGTTTGTCT

>Cs1g08450

cattaacatctaaatagaaaaaataaactttttttacttttatctgaatgaaaaaaagtcgaaatgacaagtaaatattatattccaaaaatatctctgtaaaataatttattttctaaactataaaatctaatcctttttttgttttaatttacaaggacataaatgtcaaataactaaatttaagatattttttattatgaaaacaaataaaataccatnnnnnnnnnnnnnnnnnnnnnnnnnnnnnnnnnnnnnnnnnnnnnnnnnnnnnnnnnnnnnnnnnnnnnnnnnnnnnnnnnnnnnnnnnnnnnnnnnnnnnnnnnnnnnnnnnnnnnnnnnnnnnnnnnnnnnnnnnnnnnnnnnnnnnnnnnnnnnnnnnnnnnnnnnnnnnnnnnnnnnnnnnnnnnnnnnnnnnnnnnnnnnnnnnnnnnnnnnnnnnnnnnnnnnnnnnnnnnnnnnnnnnnnnnnnnnnnnnnnnnnnnnnnnnnnnnnnnnnnnnnnnnnnnnnnnnnnnnnnnnnnnnnnnnnnnnnnnnnnnnnnnnnnnnnnnnnnnnnnnnnnnnnnnnnnnnnnnnnnnnnnnnnnnnnnnnnnnnnnnnnnnnnnnnnnnnnnnnnnnnnnnnnnnnnnnnnnnnnnnnnnnnnnnnnnnnnnnnnnnnnnnnnnnnnnnnnnnnnnnnnnnnnnnnnnnnnnnnnnnnnnnnnnnnnnnnnnnnnnnnnnnnnnnnnnnnnnnnnnnnnnnnnnnnnnnnnnnnnnnnnnnnnnnnnnnnnnnaaaagaaaaaagaatgacttgaattgtgagaaagaagaaaaatataaatttatttctgttgtccatggtatttggtttgcaatgtagaaaagtaaaattttgcannnnnnnnnnnnnnnnnnnnnnntttatgtctTAATAATTAAAGATAGTTGACTTGTTGACATTACTGTTTATAAGATTCCAGTTCCAAGTTTGTTTCAAAAATCTACCTCACATGGTGG

>Cs1g21350

TAAAAATATACAttttatgcttttttttTTTTAACTTTTGCATAATTTAAGAAATCARAATGAACTAAAAATAACATATTATATATTTTTAGAAAGAAGGATGTTATACAGAAAAGTTGAAAAGAgaaaatgggaggagcataAATAGGTATAGATATTTGAAAATAAAATTTTGATTCGTTTCAGATTAAYGAAGTGTTTATGAAAACTTCAcatgctttttttataacaAAAAGAATGATAGATAATCAATAAAAGCTAAATCACATTATTATTACTAGAATCCGTAAACACTAGGGAGCAAtaactaaaaaatagaaaATTATACAAACGCAAAAAGATTTTGAAATTAGTATTTTTATCGTAAAACATCATCAACTACTCATATTATATCCTTAAAGATGATAAAATTTCAAAATAAAATTTTATAtttttccccagtattAGAAATGAAGACCTCTGTTATTAATACACATTAGAAGAGAAYACTAAATAAATTAYATTTATTATTTATGATTRAGATTAGTAAGTACTATTAAGTATTATTACAAATCAAATCACACTAAAACTGACAATATATGTTTTCCACCTTTGAAATTTCAATGAATAAACTTAAAAACTCTCTAATGATGTTATAGTAATCATTTACATTGTCAATTGAAACGTGGAGCGATGTGCCCACATCAAGATTACGAATATAATTATATGTCCATGTATGTTTAGCCTAAAATAGTTGTGCATCACTTATCCGTTGCCTTAAATTTTGGTCATTTTCATTAACTGAGGGGGAWGTTTTTAGTAAACTAAAATCTTAAGCACTCCCTCAMAAAGTGAaaaagtgaaaaaGACGAAAGAAGAAAATCATAGACCCaatttgggggagattaTTATAAGTTGAACTTATCCAGAAAATGACTAAAATTTTGTTTTTAAtttttaaaaagaaaatAAAGGGATAAATATAAACTAGCATGGCAAGGCTTTTGAGTTGAGTTGTGAAG

>Cs1g25520

nnnnnnnnnnnnnnnnnnnnnnnnnnnnnnnnnnnnnnnnnnnnnnnnnnnnnnnnnnnnnnnnnnnnnnnnnnnnnnnnnnnnnnnnnnnnnnnnnnnnnnnnnnnnnnnnnnnaagcttcttatttgttaaaaaataaaagcttctactttccttccacatgctatgtgcatattagaatgcacagagatcataattaatcaaatccatgaaacactttcactgatttcacgcgaagcagcaatatcttaggcctttgatatgagaaaatttggagnnnnnnnnacagtcacttttacacaaggttcagtacttcagttcactcatataactggatccaatatataattcattcaaagtacaaaagctaattctcaaattaatgatttcaacacaatgctctttgctgccaacataaaatcatgactgttagcatgtcctcaacttagcatcatacatacgaatttcatcataaaacaaggaggagGGaaaaATGGATAAAGCATTAAAGGCTGCTCAGAAGGAGTGGAGTAGGAGCTGATGGGGTTGTAGCCGAGATCAAAATCACTCATTGAAAGTCTGAAATCAATATGACAAAAACAATGTTATGATTGTTTCAAATGGCATTTTACACCTAATAAAGGGTTAGCACATCTTTAAATTAAAGAGAGAAGAATAGAGCAAAGTACAAGAAGCAGCAAAGAGAAGATGAGGCGTTCAATAAATCCTACTGGAATGGAAACAAAAACCTGTGTAGTGGAAACTGAAAACAGAGGAATTTAGCTAACCTAATGATTGCAGAACTTATTCAAAATATTACCCTAGATATGAGACTACAAGCAAACAGGGCATTCTGGTTACAACCCTAACAGCCCCTAGAGATATTGTCAAACTGCAGCCCTGTATAAAATTACAAGTCCACCTGAGAAAATGTCAAGGTTGTAGACTTGTAGTGATTAGAAACCTTTCCTGACTTAATTTACCTTCACCGGTGAAGATG

>Cs1g14893

nnnnnnnnnnnnnnnnnnnnnnnnnnnnnnnnnnnnnnnnnnnnnnnnnnnnnnnnnnnnnnnnnnnnnnnnnnnnnnnnnnnnnnnnnnnnnnnnnnnnnnnnnnnnnnnnnnnnnnnnnnnnnnnnnnnnnnnnnnnnnnnnnnnnnnnnnnnnnnnnnnnnnnnnnnnnnnnnnnnnnnnnnnnnnnnnnnnnnnnnnnnnnnnnnnnnnnnnnnnnnnnnnnnnnnnnnnnnnnnnnnnnnnnnnnnnnnnnnnnnnnnnnnnnnnnnnnnnnnnnnnnnnnnnnnnnnnnnnnnnnnnnnnnnnnnnnnnnnnnnnnnnnnnnnnnnnnnnnnnnnnnnnnnnnnttgatacccctcttaattagtggaaagcatggtgaatcgacaaaagctattacgcgaacttggagataaatcgtcacttcccactatatttgggaacaggaaaacacaagctaataaagatcgaaccacgaagcgtgacttgaagatatttgatttccaaacaatagctgctgccaccgacaacttctcaactgcaaacagacttggacagggtggttttggtcctgtttataaggttctttttctctttgtggtgcttatctttttttcagtcgatacatttggagtataattacaaattagagacaaagccagtatgatttcaggggaaattacttgatggccaagaaatagcaataaagagactctcaaaaagttcgggacaaggaatagtggagttcaagaacgaagctaaactcattgcaaaacttcagcacactaatcttgtgaggcttttgggatgttctctccagaaaggtgaaaggcttttagtatacgagtacttgcctaacaaaagcttggatttctttatcnnnnnnnnnnnnnnnnnnnnnnnnnnnnnnnnnnnnnnnnnnnnnnnnnnnnnnnnnnnnnnnnnnnnnnnnnnnnnnagaaacattccatttttttttccattgttcgatgagacag

>Cs1g19230

GGCAATGGAACTTTAAAATTTCATTTGTATGTGATTTTGTGTTATTGCTTTCTTGTTTTATTTATTATTGtagatttataatttatagtggatttctaaactattatgtaagtttgtttagtgtcgaactttattaagttgatttatttgactttattatgttaATTTATAGACTCAAGATTGTTATTGTAATAACACTATAtttttattatgttttTTAAAATTAAATATACATGATAAAATTATTTGAATCAAACAAAATAAAACCAATTCAAACTATGGAATTGAACCGAATGGAACTAAATTGATTTGGTTCGGTTTGATCCTATTATAGTTTCTATTACTTGATCCGGTTTTGGGCGGGAACCGAACTAAATGGGAAAATGCCCGGCCCTACGAGCAACGTTCGTTTTACCCCGAGTTTGGtgaactggaaatcCGTCATTTCTCTCTCCTATTGgtgctgttgaaacacgtcACTTTCCCTTGCGAGATTGAGACATAATGCCACGCGTACAAGAGAAGCCGCATCTTAAAACGACATGAGCTGCCGAACCGACGTCAAATGTTTGAAAATTGTAATTAACCTTCGatatttaaaaaacctcgAAGTGTATAGTATTGATAGCCGTTAACCTCCGTTACGACTGAGTAAGGTGAGATATTAATTGAAGCACGACgaagtcgacaagaatggACAGcggccgCAGggctctccttgatttgagtggtcatcccAATTTTTTAATTCGAATGGTCATAAGAACAAATGGATGAGTCGAGGCGAAATGTgatttgtgtctgtgaatgGAGAGGGCGAAGGAAATTTGGCGACTGGCAGATGTTATCTGGTTAACGTCCTTGTAACGTGTCTATCCTGCAATCTAGTAaaaaaatttaataaTAAACAAAGCATATGATATTATAACATATCGATGACTAATCTATCTCctgtaggaagaaagcttGTGTCTTAAAATACTCGAAACTAAATGCCAACG

>Cs1g05750

TTACGGATTCacggatttttgcggatTTATAAAAGTAAATTCATAWTCAATCCATCGACTGCGAATTTTAAAATTTTAAAATTYAATCCGATCCACCAATCAACGGATCRGATTTTTGCGGATCTAATAGATTAAGCGGATCGGATTGGATTCTGAACACCCCTAAAAAAAataacacacttaGGTAGTGCTTGTTCTTTTGTCTAAAATCTAAATGTGTACGGGTTTGAYTGAAATGTAAATGGATGTGAATATAATTATCTTAATGACATGCTTATTTTTTATTTTTTAATATCTTAATATAAGCAATAACTTGTTTGTTTTTATAAATAAAAAAATTTAAACATTATTATTTAACATTTATGTCTTTAAAAGTCAAAATGAATAACRAGATTAAATTTTATAGTCCAAGAAATAAGTATATTTAATACRGATAATTTTAGGTTATAATGTTCGCTTCCCATTCAAACATTTCCCCATTTAGACAAAGATTACRAAAGTTCACttttGAGACGTTTAACAAACATGCCCTACCTGATGAGTTACAATACTTAATGTATCACTTAATACTGCACATCACAATTATTTATATTATCATAATATGCCTATAATATATACATACAACAAATATGTAATTAATATATTTTACTTATGAATGAAGATTGAAATATTACTTTCAATAAACTAAATTTTTTAAAGAAAATTTTTATTATTTGTAAGGGTAAATTTAGATAAATGATAATAGTCATAATAAGTGGAAAAAAAGAAAAAAATGAAGTTCaattaattattattattataaatttAAATTTCAAAATTCGCAATAATGCGTTTTCTCTGACGTTACTTTGGAGGGAAAACGAAAACGAAAATCCACCCGTaagtggcgcgcacgcacCCCCCCTCTCGATCCCACAGCGCAAGTTAGCATAACCCAAAATTCAACAATATTAAATTAAAAATGATTCAAAAATTATAAAATTTAAATAAATaaataaatt

>Cs1g23460

nnnnnnnnnnnnnnnnnnnnnnnnnnnnnnnnnnnnnnnnnnnnnnnnnnnnnnnnnnnnnnnnnnnnnnnnnnnnnnnnnnnnnnnnnnnnnnnnnnnnnnnnnnnnnnnnnnnnnnnnnnnnnnnnnnnnnnnnnnnnnnnnnnnnnnnnnnnnnnnnnnnnnnnnnnnnnnnnnnnnnnnnnnnnnnnnnnnnnnnnnnnnnnnnnnnnnnnnnnnnnnnnnnnnnnnnnnnnnnnnnnnnnnnnnnnnnnnnnnnnnnnnnnnnnnnnnnnnnnnnnnnnnnnnnnnnnnnnnnnnnnnnnnnnnnnnnnnnnnnnnnnnnnnnnnnnnnnnnnnnnnnnnnnnnnnnnnCCCCCCAACTTCGTGATGATTTGAAAATGTTAATTACTTCCAAGTTGTCAATTTTTCAAACATTGAGCTGGAGTTTGAACATGACGGAGAGCTGTTCCCACTCATTAATGATAGTGCTTCCAAAGCGCGACTCATGAAGCtttgtaagttataTGGACCATTAAAAATGACTATTAACTCAAATGCAAATGAGTGAAGGACCCATTCTTCAAAAGTTAGTGCAGATAAAAAGGATTAATTTAAGAAAAATATTAATTGATCTTTCCAGCAAAAGAGACAACTTTTTGGCTTCGAGATGGCCCAAATTCGGCCTACACGTTGTATTCGTTCCAAATGTGCCTGAAATCGTTTCCATTATTGGTACTTGGCRGGTCCATGCAAGAATTAGAATTTTTTAAAATAAATATATATATAaataaagagagagagagagaaaagttAAATACATAATGACGTAGCAAGTGCAGTCAAACAAAAGTGGGAAAAACTGATGTAGTAATAACTAGTAAGTGRGCTGMTGAAAAGACTTCCATAGAAACAATGAGATACATCTGAAAGAAATAGTCTAAAGTGTCCTGTTATTGATGYAAGTTATGGTTCAATTAAACTCCAGCTTTCACTAGGTTTTGAAATTGACGTTTTCTTAT

>Cs1g08660

ttagtggtttaaaatacatcaactaattaatataggaaggaaggttccttcatctttcctcaccaatgccactaaggcctaattttaatgccataatctcatatttaatttacaaccttttatttagtgtcttttggcttcttcaaactctataattattaagtatttaagatttgttatgtttggattttttttattatttaactaacatcctctttagaaatgttcttgtgtttttattttttgacttatttaaattttttgtattgtgtgcttcatggagtattcttttcttcatgagttaaacaatgataaagatagttggatgataagtgttagactttgtaagatgtgagagtctgttaataccaaaaaaaatagagagttgattagtgtgaatataatttttattgatgaaaaggttgtgtacacttaactatacacattaatatatatttttaaagttaacaacaaaataattaactttcataaataacattagttttgtaaggctgcgtcatctttaaaaaaaattaattcatattttttatcaattttatcaagtgtaaagtaataaaaaaaataatactaaatttttttttaaagccgcacgaagtgcggttctatttactAATTGCCAAATAAGGTCAAGGTTTTTATGTGGAGAGCAGCTAAGAACCTTTTGCCAACTGCAATGaacttgtggaagAGAAAGCTATTGTAGAGCCCAATTTGCCAGCTTTGTAGGAGAGGAGTGGAGACCACATGCGATACTCTCATGGAATGTAAGCAGGCTTATAAACTACAGAGACATGCTCCTCTCACTATTGCTAGAGCAAACGTGGGTGAGTTAAGTGTGATTCAAGATTTGTCGAAGGATTTAAACAAGGCAGCATTTGAACCTATGTTATTGGCTAGCTTGGAATGCAAGAAACGGATTTGTTTCGAGAACAATAAGATAGATCCTTTGATATCAGTGGCAAAGCCAGAAGCAGTGGTTG

>Cs1g24110

nnnnnnnnnnnnnnnnnnnnnnnnnnnnnnnnnnnnnnnnnnnnnnnnnnnnnnnnnnnnnnnnnnnnnnnnnnnnnnnnnnnnnnnnnnnnnnnnnnnnnnnnnnnnnnnnnnnnnnnnnnnnnnnnnnnnnnnnnnnnnnnnnnnnnnnnnnnnnnnnnnnnnnnnnnnnnnnnnnnnnnnnnnnnnnnnnnnnnnnnnnnnnnnnnnnnnnnnnnnnnnnnnnnnnnnnnnnnnnnnnnnnnnnnnnnnnnnnnnnnnnnnnnnnnnnnnnnnnnnnnnnnnnnnnnnnnnnnnnnnnnnnnnnnnnnnnnnnnnnnnnnnnnnnnnnnnnnnnnnnnnnnnnnnnnnnnnnnnnnnnnnnnnnnnnnnnnnnnnnnnnnnnnnnnnnnnnnnnnnnnnnnnnnnnnnnnnnnnnnnnnnnnnnnnnnnnnnnnnnnnnnnnnnnnnnnnnnnnataatggtcaAAGAAGAGTGGTGGCAAAACTtaaaataaaaattagttaaagattaaaaagattttcagtacatgcaatcacataaaaattagttgtatttttgagtttttcttccaatgatttaaaaatattgtgttagttacaatttgtgcttatggtgattattcaatggctatattaaactatttcatcgttgatttcatttaagaaaaaattaataaattttacatattttgtagtgttatttaattgtataaaaaaatattttaatataatttataccactttaataaannnnnnnnnnnnnnnnnnnnnnnnnnnnnnnnnnnnnnnnnnnnnnnnnnnnnnnnnnnnnnnnnnnnnnnnnnnnnnnnnnntttaacaaatatgaatggaATAGACTATTCCATGAAACTGGATGGAAATGACTTTTKCCCTTAAATTTGACATGTACAGATATACAAGCCCTAACGACACTAACGAGCCATTTAAGGAAAGCGATATTTACTGGCATTTGCGGCGCATCCCAAGGTCATAAGAAAA

>Cs1g15080

ACCATGTCTATCTTTCAAttttttttttaattttTTACCCTTTAGCATTATCTTAGATTATCAATAYACTTGTGTTTTGTGTATTTTCTTAAAAACGACAYTCAATTTGGTCATGTGGAGTTGAAAATTAGGWGAGGGAGAACTAGTTTATATAATTAGTGAGAGGCGAAGTACAGATAATTGAAGATTATTATTTATGGTGATAGGGTTTATTATAAAAATAAAGAAACAAAACGAAAAGGCAGACGAATAGATATGAGGGGCATTTTTGGAATTTTGAGGATGTTATTAGAATTATTTTAATTTtttgaatttttttaatggAATAACTTGTATAGCGGGGTTCTATAAGAAAATTTGAAGGGTGTGAAAAGCTtaacccttttttttaactcTCCCCAATTCTCCGGTTGAAGATGAGCGGGgcccagaattttctacacgacgtcgtgtcgtgaatttgagctccctttggtcctggatatggactcattctcagtagacccacagctgtccgtcgaacaagattctcacatttaaaaaaattaTTTTTTATAAAACATCTTTAAATATTAAAATAACAATCCCACGTTCTTTTCCGAATGAAAAAAATGTACTCATAAGCTATTGATGTAGGCGTAATACGACAGTCTGATCTGAGTCAATATCAARTACATTTCATACTTGCAAGGGTTAAGAAATTCACATGCATCTATGAACATCTAAGCAGKAATTGTAAATACAAGAAAGAAAAGCTTCAAAAGGATCGTCTTCAATGAGAGTAGAAAATTTTTGACGATGAATTAAACGACCCAATCattttagataaatCTtTAAAATACAGAAATacactgggtgattggccAAACTCAAAGGACTAAAAAGAGAAATTAATTAACTAGCTATAAAAACTTATTGCATTTTttggtttggaGTACATGCTGtTTAGGgtgagaaatatcgtgaccaggaatgctcgatcagaatgagctctcctcgat

>Cs1g03440

AAATAAATTTATATTTTCTCCGGTCCCTTACCTTTTAAAAAAGATtaaataaaaaattagttGTGCTTTACTTTAGACAACAACAAASACTTAACAATGGGCTCATTATCATCTTACTAAATATCATACATCGTACTGAAGTTTAGTTTGCAAAGCAAGAACAAGAATGATGACCAATAATGACGTGGTCATAATTGTAGCAAATCAAACCTAGTCACAACAAATAGATAGAACCAGTGAAAAGCCGAGGCTTCAGAGACCAAATCAGCAACATCATCAGATGAGAAGAGTCGAGGTCTCGTTGAAGATAGGTATAAAGTGCAATAGCAGAACGGAGGccaaaattgtagatgnnnnnnnnnnnnnnnnnnnnnnnnnnnnnnnnnnnnnnnnnnnnnnnnnnnnnnnnnnnnnnnnnnnnnnnnnnnnnnnnnnnnnnnnnnnnnnnnnnnnnnnnnnnnnnnnnnnnnnnnnnnnnnnnnnnnnnnnnnnnnnnnnnnnnnnnnnnnnnnnnnnnnnnnnnnnnnnnnnnnnnnnnnnnnnnnnnnnnnnnnnnnnnnnnnnnnnnnnnnnnnnnnnnncaccggatcggcttataaatagtaaagtgctggtcaaattggtgactctatacgagagcgaggtttgaacCCCTGACTtCTCTTaAGGAGTGAAAGTGCGGAACCACTTACACCAACCAACTTTARTTTTACCGCGATATTTTAAATTTATGATAATGTGAATTTATTGTATTGTACTGGGGCTTTAGGTTATAATATTTGTACAAGACAATTGCATTTTACACATAATCTAAAGTATCCCTACCAACGTGAGTATATCTTTAAGCAAGTAGACATATAGAAAGAAGAAAGAAAGTCTAAATCCTGCACCTGCAGCATTAATGAGCTATCTATAAGCAGCACCAACGGAGGAAATATTTTAAACAAAAGAAAATAAAacccctggcgcagtGAGAAAATGCGAAAGCATCAAGTGATAAA

>Cs1g07060

TCAATTTGATTTATGTAGATTTGGCCGCATTAAGTMTATAAGTTAAAGAAATACAGTTTTAGAACCAATCATGTGCAATTATTAATTTAATGAGAATAATATTTTTATTCTAGATAACGCGGCCTAACAAACGTGCCCTAAATACTAAACAAGGCCTACACCTAATAATAAtggtatttgnnnnnnnnnnnnnnnnnnnnnnnnnnnnnnnnnnnnnnnnnnnnnnnnnnnnnnnnnnnnnnnnnnnnnnnnnnnnnnnnntatttatttattaTTTATTAttttttgctttcAGTACGATCCAATGCAATTCATCCTCCAAAGAAacatctaaaaagagttAAACTCCAAATATGAATTAAAGATAAAAACAGTGATCAGTAATTTTAATGCACCAAGTTAATACAGTACTTAAATAAGGTCTCTTCAAACTGGACATTGATTATGAGGGGTAATTCTTTGCCCTTTAAACATAATTTCTGTGTTGGAACATGATTTTGAACATTATGACAGCTTTAAATTTTATTAATTACCCAGAAGAACAAATAATCATAAATAGAGCTTTCTCAAGTCCCAACTCCTCAATCAAGGTAGCTTATCTTTTGTTCTTTCGTTTCTAACCATGGGTATGACAATAATATTCCAAGCTTTAGTTTCTKTTCTGCTGCTTGCTTTGCCTCAACTACTACTACAACTCCCCCCAACTGCAGCCAAAAGTTTTAGCCTGGAGCTCATCCATAGAGACTCCAAAGAATCCCCTTTCTATCGTGCAAAACTCACTTTCCGCGAAAGAATAGAAAGCTACGTCCGAATTTCTGAATCTCGGATGGCATATCTGAGGCCATTTCTGAGATATAACTACACCACATCCACAAGTCCTGAGATGATGCACCCGAAAGTTTTCCATCATTCAAACTCATACATGGTGCGATTATTCATTGGCACTCCTAGCAAGGGAGTCCTCCTGATTCTTGACACAGGTTCTGCTCTAAtgtgggaca

>Cs1g19660

tttattTATATGAATGGAGGGGCGTTACAAGCAATACAAATGATCAAGAAGGCTGCATGTGATTATGATTAGACGATAATCTATGTTTTAATATATTTAATAATACATAAATGTATAGTACAATTATTGATCGACGGATACACTGCTCCAAGCCCGTACAACAAATTGTACAKAATAATTGATCCCCTATTATTAGAGACGAATGSGTGAATGTATACTAAACAATTTCGCCGAATAAAGGCAAATTTGCCAGACAACTTTAGATTTRYTTTCGATACTCGAATCAATTCCAGGCTGAAAGTAACTGAACCAGTTTCGGTTCTTGCAATCTGTTCGTTTGTTTTTTCGCCCCATGAGGAAAAAGGGCAGGAAAGTTGATACGTTTGTTTTTCTAAGAACTTGTTGGGCTTCTACAaraacctttttttttttcGAGAAATTAATTTTCAGAAAATCAGTACAAATTTTTACTGAGTTCATAGTATTTTTGTCAAAAYAAAAATTCATTTTCaaaatttttttctctttaaTAATAATGATAATAATAAtaactgtcttttGTTTATACTTTAAATATAAATTATAATTTCGTTAATTAAAAGAGATTTCTTAACTCAAAAGATGAAtaaaaaaaattaaaattaCTTAATTAATAATCACTTTGCCAACGATGTTTAAAAGATTTCGGCAAATGTGTATGCATAAAGAAAACTCTTattgctttttgttttaGGATGATGTAAGATACTTCGTGCTCAAATTRTAATATCAAATGCTTCATGTGAAATAAAACTAAATTATAACTACAAATTCTAACATATCTGTTGGAGAAATTTTAGAATATCTTTAATGTATTTCAATTAATTAATACATANATGACATGTATAATTGAATTTAATATAATCATCACTTATAtgataattttttaAAaTAAAcACATACATATTAcAATaacatttGCGTATTGTATGATTGGTATGATATTCTAAAATTGGTAAGGC

>Cs1g14690

CAATAGTTGGGTGTGATCTTGTTGAAGTAGAAAGTATTTTCAAGCCACGAAAAWTTCCAGTCTTCTTTATTCTTATTGACCACTTTCGGTAATTAAMTCAAATACTTCTAATGAAATCTGGACTAATTACGAAACAGAATCTTACAAAACTAAAGAAAAATATTCGATAGAATTACCCCCATCATTTTCTTGATTTGTTTATTTAATACCTCGCGTTTTAGTTTCTAGGGGCGCcaccctataayctcaCGAACATTTTGCTCTTCAATTTGACATATAGATAGACATATTAGGAACAAGTCCAATTAGAAAAAAGTGGCCACCTCATATATTAAGAATCATTAACTTGGTATACTTTATTGGAAAATCATGATTCAGAAAAATGTATAATGTCAATGTCCAAATCATATGAGAAAGGACAAAGAACAACCAACACTAGAGAGAGAAATATTGTAGAATATGCCAAGCAAGTTGTTAATTAGATACTAATAATTTATAAATTTCTGTATCTATGACGTTACAATCGTTATTAAGAAAACTAATAGCGTAGCTATCAAGAAAATTCCGCAATATTACCTGTCCAATTAGGATCCTATCAAGTTTGACTTAATGAAATTATAGAATATTTGTAGTGTTATCAAGACCATACCGCAATAGTACTAGTCAAATCACGATCGTACCTTCTAATTTGAACTCACCCAACTTTTTAGAAAACTTTGATTTATTTTTCGGGTTCTTTCTGGACAGATTTAACTGCTAATCTTTCATACACGGATTTATAAAATAAAATAAGCGCAGTAAGAGCATCAGCAAACTACGTTAGCWAMTAATAGCCACCGTACGAGAATTACGCCGCGTTTCATTCTCATCAACAATTCCCTTACGGAATATTCAATTATTCTACTCGTTCACATTCTTAATAGTTATTTAAAAGGAATAAAATAAAAATAAATCCTCTCTCTCSCCTTCCTTTATATGYTCTYYCTTTCTTTCTCTGCWTG

>Cs1g13670

agtataacttatgatattnnncaatagtaatttagttttataacttacataatcaatattttaaaatgaaatccaataatttacaaaaactgtattttatacgaaataaaaaattcttgaagttaaaattatttatttgcagttattttggcactttataatggtaaaacaatataattaaactaatattaaatcaaccttaatggtaaaagatgtttagtcacagaaaataaataaagaaagaaggatataactgattaattgtctaaactttttctttgtttgaattttaaattaattttattaattatttttagtttagagttggcaaaaattatgggggagggggatatcatccctgagaaattattgggcgaccacctaagtattggacaatattgaaaaaaaaaaagaaaaaaaaacactaaaattttcataaacatggcacaaaaatacttaaaaatttagcaagtgtatccccaaattacttttatgttaaccttcattattgtattccgacgtcacaagggtgaatctgatcaccatccgcagttctaaatttagagtgtttaaacctaaaaattacgtgcatgcaaagcaaaagagtcaaattcggatttacccaccctgctaaatcctgcctctaaaaggacagtaacattagcgtcatttttaggaattcaatacgttgaatccattatctacgataatgtagaataataatttttcattgttatgttgttttgttgctgttgttgtataacggtacgcgttgactttgacaacaatttttggacggccacctatcgctagttataaaacaaaatggctcttaaaatatggtcgtcatactcaaaatttggaaagacggtgcaaacttcagacaaaattttcaatcgctcaaaaagttttgagtgaatgtcttatgcactaatatcgaaattagttgtttgcataaccagttcagaataattgtgggttttgtttggcttcagttgtaa

>Cs1g11940

tatgtttttatttattgtagagacttaaaataatatgaatgaattggaaaccaatcttcacaaagaaagaaaagttagccttcacatggaagtatggaaggaaagactaacctttataaagaaggaaaaagcaaccttcagaaggaaagaaagacaaaaagatgaaaatnnnnnnnnnnnnnnnnnnnnnnnnnnnnnnnnnnnnnnnnnnnnnnnnnnnnnnnnnnnnnnnnnnnnnnnnnacattaaattaaaataaaattgaatagaaacaaatacaatatcaattatcacaatgcaattagtaagtgtctatttgctatgacttgtctaatagttttaagtatttatttaatcccataagctcttaatcaaattttttgttattgtttgattatttgtttagtagaatttttttaatattaaataagatatttggtgtccactatcaaaagcttaaattttggacttttgagagtaaaagttgaaattttttttgtaaaatactaaaatgtttaaaatattatttacttatttgacaactttatttcattatttttcataacctacctttaaaaaacttacatattaaaataatttttataacttttaataaaatattgacaaccaaaaaataaatttttttgcgtaaaatatctatttgtaaaaactctgctaatataaacccatttaaataaactgaatttaaaaagctacaaccaaaatacaactagaattgttttttttaatatttaattgtcgggctgaaggatcggactcagactttcttgattaacccctagaccgagcccgatccgactcgaagcctaaaagtggagccgaagcctgactgggccgggcgctcacttttctgccatccctagcggcaactcaactcaagccttggctggatggtcgtagtttttgcgcgtttcagttgacaggcgttgatacaggctacacggggtatgcgcagagtcttaacctacacccacaacatt

>Cs1g24670

nnnnnnnnnnnnnnnnnnnnnnnnnnnnnnnnnnnnnnnnnnnnnnnnnnnnnnnnnnnnnnnnnnnnnnnnnnnnnnnnnnnnnnnnnnnnnnnnnnnnnnnnnnnnnnnnnnnnnnnnnnnnnnnnnnnnnnnnnnnnnnnnnnnnnnnnnnnnnnnnnnnnnnnnnnnnnnnnnnnnnnnnnnnnnnnnnnnnnnnnnnnnnnnnnnnnnnnnnnnnnnnnnnnnnnnnnnnnnnnnnnnnnnnnnnnnnnnnnnnnnnnnnnnnnnnnnnnnnnnnnnnnnnnnnnnnnnnnnnnnnnnnnnnnnnnnnnnnnnnnnnnnnnnnnnnnnnnnnacttGAGCGCATTACTGGTGGTATGAAGGTCAAAGCTGACAGGGATGAATCCTCCCCTTATGCAGCTATGCTTGCAGCACAAGATGTTTCTCAACGATGCAAGGAGCTTGGCATTACTGCTCTTCATATTAAGCTCCGGGCTACTGGGGGTAACAAAACTAAAACTCCTGGTCCAGGTGCTCAGTCAGCACTTAGAGCTCTTGCTCGTTCTGGAATGAAGATTGGCCGCATAGAGGATGTGACTCCAATTCccaccgycagtaccggcagaaaggGTGGTAGAAGGGGTAGAAGGTTGTAATTCTTTTCCTCACACCCATCCATAACGTTCAAGATGATGCCGTGGTTACAGAGAGTTTCACTCTCTTCTGTTCGATGCAAGACTTATTTTCCAATTTTGTTGTTTGGAATGTCTGTAGTCTCGGATTCCTTTGTGTTAGCCTTTCCGATCAATTAAGTTTATACAGTGGCAAGACTAATCATTTCTCCAGGAGTTTCTGAATTTTATtgtttatctacaagtatTGTGTTTGAAGTGGTAAAGTATGATGGTAGTGGTTATCATTAGATGTTGAAAAAAAAATGGCATATGGATGCACGTTGCAGAACAAGGGACAGGTCCTCTTGTTGTTCTACTTCATGGGTTCCCAGAATTATGGTATTC

>Cs1g23330

aatccactttgatccttaatatatatttaactagatgtagctctaatattgaagggtgttattattattctctaaatatagagacatacttatttagtaatgacaataaagaGGGGAGGGGAGATCACTACGTCActttattggtctttaaaatgttaatagatgaattttttttatctctctaccccaaataacttactatttatgttcaaaacttaatttaattaaactataataagaccccgtttaatgaataatgatttgtttaactaaaaaaaaaaactaAAATACACTATGTTAATAACTAATTAGAAACACCAAAATATCAAAATAAACTAAAGTTATTGATAATTTTTTGTCCAACTAAGATCAATGTCATCYACATATCTTACATACGAGATCGTCCAAAATTTAAAGCAMCAAACATGGTTATTAACACAAAATAACAATGTTTTTAGCACCAATTCATTACATAAACCACCATATTTAACTATTCAACAAAATCATTTAAAGTATCTATGTCAACATTATATCATGAACTAAATGTCCCAAAGTATCTACTATGGACAACTTTAGAATCGAAAGCATAACTAWACTTTTAAGATTCTTCATAAATTTACATGAATAAACTTGTATCTATTAATTATGTTTTTATTAAAAATTTTAAAAATAAATATATTAAATATCAAATGAATAAATTATTTTATGTATAATGTTTATTAATAATATTACATAAGCTTATAAAAATACAGTGTATYGGGGAATAGGGAGGCGATTGGACTTTCTCTAAATAAGAAATGAGTATGAAAATAATAAGTATACCCATCCCCTAGTTAAATTTGTGTTTCATTCATCATCTTGATCACAATCCTAAGTCCTACAGCGAAATGGACGCTTTTGGTATTTCTGCCATCTCCTTTAATTTAAATATTAAataaataaaaaagggaaAAGGTCAGGGATCTARATGATAGAACCCCAAAAGTAAACTAACGTGTGCG

>Cs1g05350

acttaaatgagcttatgtattactcattttgttatcatgcatgcatattgttatgtgatatggatatgccatatattgtgtacacatttggttgcgtatggcattatgtatagaatgaggatacgatggtccgagacattttgcttagtctcaatatggacattaaagactgaagcattatgacttagcttcagtgtgctttctggagacattttgattcagtctcattatgatcataagaaagcacgaatnnnnnnnnnnnnnnnnnnnnnnnnnnnnnnnnnnnnnnnnnnnnnnnnnnnnnnnnnnnnnnnnnnnnnnnnnnnnnnnnnnnnnnnnnnnnnnnnnnnnnnnnnnnnnnnnnnnnnnnnnnnnnnnnnnnnnnnnnnnnnnnnnnnnnnnnnnnnnnnnnnnnnnnnnnnnnnnnnnnnnnnnnnnnnnnnnnnnnnnnnnnnnnnnnnnnnnnnnnnnnnnnnnnnnnnnnnnnnnnnnnnnnnnnnnnnnnnnnnnnnnnnnnnnnnnnnnnnnnnnnnnnnnnnnnnnnnnnttatagtcacatattgattaattcggttgttttcaaaatattattttattttaaatttatcattagagtgatttctgtcgtccaagtgggagattgttagatttatttctcacattatgtggactgacataaatcattctaattattttctatattttaccgaattaatacgtgacnnnnnnnnnnnnnnnnnnnnnnnnnnnnnnnnnnnnnnnnnnnnnnnnnnnnnnnnnnnnnnnnnnnnnnnnnnnnattatggacaagtatatctttaattgtgttattttagcccataatgagatgggcaattttgtactaacacatgaggggtccctgcgctcaaccgctgtatttaagtaacttcagcccattttggaagttaactgttgaaaaatcagttgcatgtgggtgcagtgcacctctctctctccctctccctctccctctcgtctcc

>Cs1g13960

ATTCGGTGTTTSTGACTGTACAGATCACGTGTATACTTGTGGGCRCCTTTWATGATCAATTCGATTTCAATTTTTCTTGCTTttcagatttttttttttcaaaaTAAAATAATTGTTtcttttttcttctataaaagGATtATGGATTAATTATGTGccagcTTAGACtacattttttcaatttcaatAAacaaaccttgcatgatatgataacatggtttaattttttgagtagcgtaatagctacagattttcatataaattatttatgtatnnnnnnnnntgatattatataattagttgaatgaaaatataaattaataacaataaatatatgcactaagttatattttcattcaaccactcatatgataccacaaagagtttgtggttatatcattattgtaatatttttaacataaaattatgaagattataaaaaataatactttaaaaatgacagatttatttatagtaacttacattttcaattctcaatattttttagtttcgtaattgaatttttacccaagttAAATCTGgATAATTTTTTGTGATTTTCattttgaagaaaaGAAAAgAAAAAATTCTTATGATTACAAACAGAAGAAATTAGTGAACATAACGACAGACAGCGGAGAATGAAATCAAATCAAAACCTTTTATTTCTGCRGGGATCTTGGGGTCCACAATaatcaattattgtccacacgcAATAATAACCCATCAACYCAATAAGAAAATGTATATAACAGAATATTAGATTTTCCATTAAATTAAATTTGGGGGYAAAAAAAACCCTCAAAGTCATGAAAACGACCGGCTCAGACCGACCAGCCAATGGGGCCCCGAACTTTACGGCACTCAAAATCACACGCTCACGTACGGTTACCTACATGGACAAATCATAACCGCTCTCTTAAGGACACGTGTTGAATCGGGAGTGGTTACGTAATTTTGCCGGCCACATGCTCATTTTCTTCCCCGCCCTTTATATATAC

>Cs1g08670

aagctttttgtcagaatccgattttgatggaacagggaaaaaagaggaaacagctatagtagacttttggaaatggttctctattctagtcagctattttcctatagtatttaagttagtattacagaaattattctgttggataatactacttaaattcgcatcagtatcatttggctttgggattttataaggtgaagctctaatttggtcaggaggttcaccgtgatcaacaattaaactccgaagaggtggatgctcagattctatggttctatcacttagggcagtaaagccatacctgtaaccattcagtcaagaccatagatagaccataagttctttcttcatggatcctctgctctgtagagtatcgtccttttctagctattcttctgggaagactaatgattcaaagcatgttgtgagttcagaagaattcgttattgagaactttgataaagcaattgattgttgggaacttccaaagatttctaaagaaaagatttataaaacaaaaaactttgattttctaaaaaatgattatgtaataaagactgaagaacgtgaaataattctttcaaagccatttgaaacaattcaattgttttaagagcattcattaaagaaattaaaagaaaagaattttaattatgtccatataagattaatccaagttggcataaaaccattaaccaaagaaggcttcgatacttctatcctcgctgtccttagaaatggctgatttatctcttttgatgattctttactaagtagtattgaatcgagtctctgtaaaggccctatatcttttgattgttatccaaacataacaatttctcttaaagacaaaaatattttaaagagcatgatcttacaaatcaaaacccacaattatcatatgattgagggatctgtcccagttgcattaatttttaagatttcttataaagccatgatttctgcatttagcacacaacataaattccagtcaaaaagag

>Cs1g20530

CTGTGGCTTCTTATCTCTTCATGTCRCCCATTATCTTTTTTGAATTAAGATCCAGTGATCCACTATCTTTGACTTTTagataatggtaacccatTATAGGATAACCTAAAGCCTAGGTTAAAATGGCCTGCCCACATTYTCATCTtcttcgttttgtaaaTTTTAACCTTATAGTTCCATTTCACAATAGATTTATAATTTTTTTTTTAAAAaaaaaatttaccttTTGGGATAGAACATTTATTATGTYTTTTCTAATAAGACATTTTGTCCGAAAAAGCATTTGAAGCCCgtgtttaaataaaataaaataaaataaaggaatGAAAGAGCGAACATTTTCAAATATTTTGACAAAATAAATACTTGATTAATGGGGAAGTGTAATTTACATGATGTTCAttgagatgttgatgatGAGAAAGTTCCCGTAACGCAGTCAGAATCATCCCATTTCAATATTCTTTTGTTTTTCTGAAATTATTattatttttttttaAAAAAAAAAAGTCTTCCAACCTTGTATGACCAAATATAAATACACtacacctatgaCCTACCATCATGTTTGTCCTTATATGGACATTAGTAGGACATCAACTAAGACATGCTCTTATTAACGATTTATTTTTATAATTACccannnnnnnnnnnnnnnnnnatatatatatataaataCTATAAATAAACTAAACAGGGAAAACACGACACGAAATCCATACCCAcccaatcggcaatcggcaatCGGCAATCGGCAATTGGGGAATTTCCTTGATAACAGAAATTATCGATATTTCAGCGGCAAAATTGGTTCATTTTAGATCATAATAGACAACAAAATAAAATAAAATGATTAATTGATTTAATCAATAATTTGGTTCTGCTGCCTACTCGAAAAATGAAATGGATATCGTGGGAAATTGGCAGCCCGCCAGTCCGCGGTCCCCACTCCCCATCGCGCTTCTCTTACTATAAAAATACGAGTCTAGTTATTGCACAAC

>Cs1g03260

TATTAAGTATCAGTGTTTGGGTTATATATCTCTTTCTTGAGTAAMTTATGTCTAAACTTTATAATATTAAGCATCAGTATTTGGGTTATATCTCTCTCTCTTGAGTAAGTTATGTCTAGACTTTATAATTTTTATTGACCACTTTCCTATTTAAAACCTCTTTATGGCATTCTTCAATCATTTCTTGGGTTAAGTTAATATTGACAATCTCTCTAAAGGAAATTCAAGTCTATCTTCTTGTAGTGCTATTTATAGACGTCCTCATGGAACTTTTTTGGGSTGCTTTGCTATGCTAATTGAGAATCAAACTTCCTTTTATTTAGAACTTTATGCTACCATTTGTGCTATTCAATTTGCCTGCTATTCATTGTGGTTAGAATGTGATTTGATAACTGCTTATTTTTGCCTAAAAATTTACATTCTTTCTCCTCCTTGGCAACTCTGCATTAAATTACTCAATTGTRTAATTACGATAAAGTCTATGGGATTTCATTATTCTCATATCTACCGAAAAGGTAACGGTGTTGCAGACACTTAGCTAATATTAGTTTGAGTTTTTCCCACTTGACATGGTGAGATTTTCCTCCATCAAAAACTAAGAAGTCTCTAATTGATAACTCTTGGGGAATGCCTAAGTAACAATTTTCTTAATCCCATTATTGTTAGTCCATTAGGATTTGATTTGGTTTAATCATTTTTAACTGAACTCCTGAACCTTTTTTTCTTCATTTAATATATTATACCTCGCTCAAggtttattttaaaagATAAATAAATAAAGGAGAAAAAAATTACATATCCCACGCATACGAAACACGTGATTAAGTTGAGATAAATGAAATTTCAGGGAAGCACCGAGAGAAATAATAAAATAAAACCCTAATGGCATGTGCACTATAAATAACAAGAACCAATTCTCCAATCTCCARAACAATTTTGGTGATCAATAGAAATTAAACAAGAAGCGCTTCTAAAATCTTCCCAGCTTGAACTGAAAAAAA

>Cs1g12990

gggaagccactttatacagatcggagaataagatttttctctctattgttgagagaaaaattttctcgtgctagttgctttggtagtgataaaggcgcccacacgtcaagtgcagatcgaacctgagtcataatctggtagattattggtgacagttcgtgatctaacaagcgtggtggtgacggatcgtgatctaacaggagtggtggtggcggatcgtgatctaggagcctgaatcacttcagcggaaaaagccaactcggattttcaaggtacgatttctagaatacgaattcttatatattatatgagagcgatcttcaaaagattttataaaaatttaaaaacctgatttttccccaacatgtatgtcGTCGCccatgagcaatgggtgccatcgcctgagcgacgacatcaatcattcgagtgatggcacctatcgcccataatctatgccactagagtctgggcaagacttgtgactttgcactgctaagggtttaaagactataaattgagctcattagcattaaggtttaattaaaagcgtttgatactttataattcattttgaggaatgtacgtttaggtaagaatgcacataaagattaagatattcgacaaatgtcaagcacgtgcgggtctaccgcactaaatagtgtaacttacaataagggttagccaagggcgtttctatctaaaatggcgtatttttttgctataaaaattaaaagaagtcaaaaactagctaaaattatggatagctcctaaattttggctattttcatgatattttcaacaaaatttctccactaattactgaaaaaaaaaatttcctaatttaagagagaaaactgatgtttgtatgtaacggacacttgttagttactatttaatcaatcaatcaatcaagatggccacatgaactaacttgcatattttactgtttcaactttcaattccctccatcacaacattttacagtttcaagccgtttttta

>Cs1g15270

RAGAARGYAGAAAGAAAAGAAAATGCCATTGATGAACAACAAGTTCTCACTTCTAAGCGTCYGGWGTCAAGGAGCACGTCGAACATGATGATATCGCTTTCACTTCTCATGGTGAATTGGGAAATCCAAGTCGAATGAAGGGGGACGAGTCAAGTGCTCTRACGTCTGGWTTTCGACGGTTGATCTTGAGACAGGCRGGTTGAACATGACGTTATAGCATGGCTGATCGGGAAACCTGTTGAAGATGTTCCATTTCAAAATTAGTAATTTTAAATAAGAAAATCAGTTAAATTCKGTTATGCTKACGTGGcttgaccctttagcAGGACAGTTAAGTTAGTTTAGATTTCCGATtccagaaacggcgtTTTGGGGCGGATTTTATGAGTCATTTCTAATtaaattgagaaaGAAAGTGCTTTGAGAGTCTCTGTAAAATTAATCTTTGATCCGtaatctttctttttTTTttttttttttttgaAtaacaaagctttcatgcctcttcctttgagtgaatataggccgcgaagtgtGAaGCTTCCAAAtcatggcgtgcgtgatagtgCATTCCGTAATGCCATAAGAGCTGGGCTGCAACATTGTTGGGCTAATAATTTTTCTATGGGCTTTTTCTAAGGGAATGTCCGGCTTTTATTATTTTGGGCTTACATTAAAAAGAAATcctaattttttactttgTTTACATCGGTTTGCAAATTACAATWTTAATAAAATATTAATAGCTTCTTTTTCTAAAAATTCTTTATTTTCTAGAAACTTTTGCTGTAAAAAATTAATCGATATGATATTTATTTCtCATTTAattttttttttttttaaaaataaatttaataacaagcctgaaatatataaaatttcagatttaaggcctttaataaacaaacctcctactgtagtcattaaattctgtgcaaacaaaaaTAATAACTGTTGGAGGTAACTTTTGGATACACATAACGTTAAATAACGAACTTTCG

>Cs1g12610

GAGAGCAAGAAAaaagaagaagaagaagagaagccaccgcggcaaattcgggagcaataatttggagaagtcaatgcagagattggagagagacagtttcttgagtttttctttctcacatctcttttgtcttgcttgaatgttcctaattaaattcatggattctctttatattcccatgaactaatttattttactagggctacgatgtagcctaactatgaagatttaattccataaatctgtgttatttttaatatattattccatgattgagtgttcattattgtgtttaatgcttttaaatatctggccaatatttaaatgatttgaggatacatagtgagaccgagaggagatttatgtatttttgctctgtgtaatgaatgccatgagttgaacgaaagacagagatgtgccaacatgattcatgcaatctttctaagattttccatatagcttaatgaatctttgcatatttAAATTCACAtagagatatagtgggttaatatgtgaaaatattttttatattactcgagagaggaTAttgaAtAGATTagAAAaatttactgtcaacatggataataaactttaatagcatagatggaggattaaattggattggttatggtgaaatcggatgtcctagtgttttaatctcttggtattttttattattgcctgcatctttatttaattttnnnnnnnnnnntttaatttaattttaatctaaattcgcttattcgattgttcaaataaattagggttagaatcatttcggtagctaataaaatatacaatctctgtgggacgatattcTACTCTTCATTATATTACTTGTGCCGACTAGTACACTTGCTAATTTACGCATCAACAAGAGATTGAATATGTTTTGATTCATCTGTGGGCTTCTTGGTCATACTGAAAAACAGTGCTCGAAGTTATATGAATGTGCAGGCGATGTGGTGAAACCTTATGGGCAATGGATGAGGGCGTCGAAC

>Cs1g16710

AAATTAAAACCCTTTTGAATATGTCACTAAAACGATATGATTCTTCCTACCTCCTACGCCTTTCTTGAGAAAATTAAGGAAAAAATAATATAAAGCCAAWCACAGACTTWAATATTATATGMAAACTTWCTMTCAATCRGCTTTTAAATTCAACCATACAGTTGTATATAATTACCAAATGAAATKTATAYGCCGCTATAAAAATTACTTTTTCTGCCTTGCAGCTTTCACATTTTGAATCAGCTTGCAGTTCTTCGGATTCCAGAACTTTCAGCCGAAATTTTTAAATTACTTTTCTTATCTGTGTatatactctttttttctttttAGCCATATATTGACAAATGAAAATCTTCCAGAAAATCCGTCTATATCTGGCAACTTCATGTGATTGGACAAATTTTTGCATGTCTCAATAATAAACTATARTTTTGATAATTTGCCAGCACATTCCAGGGCCCTTCATCTCTTGGTCAGACTTAATTTTAACACCTTGTTTAAATATGAATAAAATACTTAGGTCCTGTAATTTTAACACCTTATTTAAATCtaaataaaaaaaatacctaCATTCTGTTTGGTATTGAGATTAGTTRATTGTAACTTAAAAATTTTTKGTCAATACGAATTGTTGTGACTTGAAAATTAAGTTGATTTAATCCACYACTTGTATAATGAAaaaaaatttataatatctttattatnnnngttaaaattattttttgaaagccatatttattaaacaccattaacttttattttataattataacttctttttttctcaacTACTTTATCTTGAAAGTTACAGTATATCAATATCAAATAAGATCTTAATGTTGTCATACAAAAGCTTAATTAGTTATATATGTAGTTCAGTCAAATTCAAAAAGGAAAGAGAGACAGAGTTAGCACTTAATATGGGGCCATTTGCGCATTGCACGTTGTGTTGTGTGTAATGTGAACATATTGCCTGAAGAGATTAATTGACCCAGCAGTGA

>Cs1g05500

TACTAATGTAGAAAGCGCTTTTGTTGTTTAAGGAGGTAGAAGGGAAAAGTGCTTTTGCGATAAGGGAGCTTAACTATTTTTACTGGCTGAAATagcagtcagctagtcagctttgcttGTGATGGGGGGGATTTCCYGTTTTTTACAACATTTTAATTAAGAGGAATATTTGTTTCTTGCCTCGTGGTAAGTGGRGATGGGACAGTTAATCAATTTTTGCTTCTAGAAATTTATCAATATCCATCATCAACCAACCCAATTGTTTTCATTAACCTAATCTATCAACTACTGTTATAATTAATTACGTGGGAGAAAATGAGATGAAAAAGGGAATAAAAGGTAGAGATaatcagtttttttttttttttttgacattTCATGATAAATGTGTAATCTAATGCCCCCATCAAAAGTGAAGTTATAGTTCACCAGCAAGTGAGTTGAGAAAATAAAATAAGTAATGAAAGTCAAAACTTTAATAACTCAATTAAGAAAATAATTTATGGGTAATTATAAATTTATGACTTATCTTTAGTAAATATTATCAGTTTTGAGTACAATGAAAACCCAATAATTAATAACTTTttttttatgatgaAGTTGTGGCTTTTGGCTTGTGGGTATAGAAAATTATCAAGCTTacagtaaaaaaaggaactTTTTTTTTTTAAAATTTGAGCTAATCAATTCTTTTTAATTGGATATGGAAACacaacatacttTTTTAGCTATCATTATGATTCACTTTGGATGATTAGTAAGATACGTCAATCAATGGATCTATAGTCTGACCTATAAATCCCTATAAGTAGGAATGATTTtatttatttatttttttaaaataaagtatgaaaatgacaACAAGAAAATTGAAACTTGAAAAATAAAATAAAGGTTGTTGGTAAAGAATTACTAAAGTGAAACTATCAAATTGAGAATTGAAATAGAAGTAACTCTGTCTATATTGAGTCTTGCTCTCTGTCTCTTTACTGTCTTGCCTC

>Cs1g08630

gtaagacccaactgtatcccagtgtTGTGCATTCTCTGCTATATAGTCAAGATAATCTAATGCATCTTCAGGACTTTTATCCCTAAACTCACCATCACACATTATTTCAACGACTTGTCTGCATTGGGATGTTAATCCCTCgtagaaataagacactaatctctatgtttcaaaaccgtAGTGTGGGCAAATGTTAAGTAGTTCTTTAAACCCATCCCAATATTTGTAGAGGGTTTCTCCTGGTTTTTTAGTGTGATTTGTCTTTTGAAAGAATTTGTTCTAAGAGATGGGAAGAATTTTTTTAAAAATTGTGCTTTCATTTCATCAAAAGTTCTGATAGAtcctgacctaagattttgtagccaAgttttaactttatcatttaGtaagAAAGGAAAAActtaAGTCtGATTAtaTTCATGCTACAATTTTGATCAGCACATATGTtacagactttctcaaattcccttaaatgcaaatatgaatTTTCAGACTCAAAACTATGAAAAGTAGGAAGAAGTTGAATGATACTAGGTTTGAAATTAAAACGTGATGCATCAGGAGGAAACACCATGCATAATGGTGACCCTAATCTAGTAGGATGCATGAAACTATATAGGGCTAGGTAGAGGGCAAGAATAGACATTAATGGAGATCATGRGAGGGGATATGAAGATTTATTTCAATATACTGCGGTGATTCACAAATATGATCATGGTGCAATTTGTAAAGTGCTTTGTGACGTTGTGACTAGGCTTGAGAAAGTGCTCTTTTAGAGATTCTTTATGGCATTTCcagctcaaaaaatgcccTtaacaatggctataggccatatatGGTGgagTTCTATTAACCAATGTGGGTATGGATGCTAATAATGGAATGGTCCTATTAGCATTAGCAGTGTGTGGATAGAGAATACTGAAACCTAGACGTGGNTTTTAGAAATTCTGCATTCATATTTTGGTAATGGATTAGACCAGATTATATTTTGCATGAAT

>Cs1g05980

TTTTATTGAGGTCGGCCATTACCTTGTTGAGGTCGATCATAAGCTTAACATTTAAGAAGATTGGAAAAAACAAGAGAATCCAAAATAGATCGGAAAATGATAATGTATAAAGTCAAAAAAaawttttccaacCATTTAACCTTCGACTCTGTGTCTTTTATACCCATTCGGAGTATTTGGCCCTCTCTCCACGATCGTTACTGGGTTAGTTGCACCATCATGGGTGAGTGGGGGTTGCAACCGCCTTTGAGTGGATCTCATGCCCGGACTCTCAGGAGAAAACCCATGTCCTGATAACTACTGTTAGCCAAGACTTTAATCCAACTCGACCAGAGTCTCTCGTCAGATACCCCCAACCAAGTGAAGTACTTGGGCGACCAAAAGATGGCTCCTCGTGAATGAGCTCCCATGATGAGTTGCTTTTGGTCGATGAAAGCACCTGTTTGCTCATAGGTTCTTGGTCATCACACTTTTCACCTCTAACACCAGCCGCCTAGTTTCTAGAGTTGAGAGTGAAACGAACCAGGACTAGAAGCTCAATAGGTATCTCCTCGACCAAGCTGCTTTTTTTACTGCTCGACATTTTGGCGAAAGGCCGCTAATATTTGCTCTTTACTTCACAAGCTTGCTGGTATCTATCCATTCAAAGACTGTTGTCGTTTTTAATTCTAGTAACGTGGCATGTTTTAGACCAATCATTTGAATTCAAAACAAGTGGTTTGCATTTTTGGTTACTAGAAACAATTCACTCTCTTTCAACAATTAAATCCCTAAATCTCATCTTTTTCTCATTCTTCTAAATTTATCACTCATTTTCTCTTAAAAGCGAGAAGCGTTTCAGGCAAGGCTTTTCTTTACTATTGTTATTACTGCCATAGACATTTTACTAATTCTGAGTTGCTCCAGGTATAATTTTCTAAACTCATCCTTGATTGTTTGTTGATTAGGATTGTGTCGCTCTTTCTTGCTGGTTCTAGGGATTTTGTTTTCGATTTCTGCAA

>Cs1g12800

GATTTTTGTTTTAAGATTCTAAGAAGAGAAAAGAGATAAAGCTATAATGAGTGCATTGCAGGGGAGAGTTAAAGCCACTAATCAATAAGTTGAATTCTTRATTGACACATTAATTAAAAGATCCGGCTACGGTGGATATGCTTGACCATAAAACACTCTTTCATAATCACACGATTAAAATtacataaattttttnnnnnnttatcattttgactgtataattataaaagggtactccatGGTACAGAATCTCCAACATAGTTATTGCCTTAAATTATCAAATTGTGATACTGTCTGCACGAGTGGAAAAAGAAGAATCATTCAAACATGCATGCCTATGGATTTTAATAGTAGTGATCATTATTTATCTAATCAAATAATATTCAAAAGGTTAATGCATATCCTCTTTGATATACATTATAATCATCGAATTTGTATAAGGTAGTGGGCGTTAATTGCAACTCAATCGTGGGACAAGAATCAGCTAAAGTAATgcaaagtattatttccctTGCCCAACTTGAGAAAGTACTCACAAGTAATCTAATTAATTAAGGAACAAGTTGATGTATTTGGGACAAGAATTTAACATAGGAAAAATCATTTTAtcagaaannnnnnnnnnnnnnnnnnnnnnnnnnnnnnnnnnnnnnnnnnnggtgaaaacaTTGTGTTTGAAGTGCAATTTGAGTGAATGGAAAACGCAtcatgctttaacaaTCCTCTATCTAACTCATTTCATTCTATATCTTAACAATCTCCAAACTTAGTTTgaaaattcaatgtgaggacgtgagttcaacgtaacatattttatttcatatcttaaaaatattggattgagcataaatatattttggataaaactgtaagtttatgtatctggggactcctaattttaagttgtaattatattgataggttgtgtAATACAaatacaatcaatttaatagaatctcataaccaggaatttgccttactcagtatataaacacaaAGCA

>Cs1g20960

attagcAGCCCGCTTGTTCTTGCTGNGAAGTGGCCTGATATTAAGATGAGCAGTGTCACTTGATAGAAAAACAATGTTATAAATTGTTGATATAAAAGCAATTTTTGATTTATTTTTATCCTCGTTTGCAGAATTAAATATCTCACGGATCcagccactnnnnnnnnnnnnnnnnnnnnnnnnnnnnnnnnnnnnnnnnnnnnnnnnnnnnnnnnnnnnnnnnnnnnnnnnnnnnnnnnnnnnnnnnnnnnnnnnnnnnnnnnnnnnnnnnnnnnnnnnnnnnnnnnnnnntcaaaatgtgagtggagtgaatctctctcgaatattaaaaagggtaaaaatctgagctgtgctatataatatttgtaaattagtctaagtaattgtccgattgtaaatatannnnnnnnnnnnnnnnnnnnnnnnnnnnnnnnnnnnnnnnnnnnnnnnnnnnnnnnnnnnnnnnnnnnnnnnnnnnnnnnnnnnccgtgattccgtcagtcctgggATTGGTTTTAATTCTTTTAATAATTTATAAAYGGACCTTGCTACCTTAGTGTGTGTGAGTACAAGGTTGYAATACTCCTGTAATTTTCTTTTTCATAGTAAATTACATGTTACCTAATAGAAATCACAACGTGAAATAAGTGATTAGTGCTTAACAAGAAGTCATATAAATTGAGGCACTTCTTAAATTAAAAATGAAAATCATAATCCGCTGTCGGCGAGGGTGAGCACATTTTTAGATTGATTTGTGTCATACACATTGACACTACTAAATGAACCGTGCTATGTGCATAATAAAAGGCCTTGATTTCTTAAATAATCATCATGTACGTTACTCACAATTATAAAGTTACACAAAACAAATTAAGTTTAATTTATTTAATATAACTTTGTGCTTATATGATGTATTATGTATGAGATCACTTGCCTTAAGTggaaaagacatcagacatgacagaATATCCCGTGTGGGTGACATTGGAGGAATGAGGGGG

>Cs1g22930

TCTTCTACAATTATATTATATTTATGAGCGCACGTATTTAGaaaaggtgaaaaAgaagaagaAGTATTTtgATTTCATAGTCCACATTGCACGGAAGAGCAAAAATCTCGTTAAATTAAACTAATCATAAAATGTtgagtcaaaaaaaaaaaaaaatcctaTAATGTTACTTGTTGCTCCCAAAATTGTCTTGTATATGAGAAAAACTTCCAAAATAGGAGTTGAGACGAATTTAGTTTAACTATCTGTTAGAGAAATTATAAAATGCATTATGTAAATGgTAGTTATATTAAATTTAAATACACATGTCACGCATGTGATATTTAGTTTTATTATCTCTAATAGTTTTTTTTTTCACTCACACTTTAAATTCCTCATAAAACTCTTTTTTAATTGAAATTACAATTACCGAAATAAATTCCTTTCTTGCTTTTGAATGGAAATTTAATTAAACATTTAAATAAATTATTTTTTaacaaatttttttacatcCACCCTAATTCGTAGTTTATATTTATATTTTAGATTCTAATTagaaaatatttatttatttatttttgaaACTTATAAAGGTCTAGAATTATGGAGATATAAACTAAATAATAAGAGGAAACTAATTTAGAAGTCAAGATTTGGCTTTAGTGGCAAGAAACAACCTTCCTAGGGCTCAAGTCCTAGTTTCAATGAATGGGTGAGTTAATAATTGAAAAAAAAAAAAaaaGAGAcaccaagttacagtaaaatagttgattttaatatatggatttatattttataaattaaagtgatgctttttaggaaataaaatccagaagagggttctacaagaattttagaggggtgataaaagctctagtattgcccaaaagatctgatcgaaacttcagcaacagtggccCAAGTTTAAtttatgtgcctACCGAGCCTTAGCCCAACTTGACAAATAAAAATCTGCATCCGCCTTCCGGGTTTGAGTCAAACATGTCGACTCCGTTTACAGA

>Cs1g02610

GACATGCATATTTTATTAATTAGTTTCGTTATTAATAATGAACAAGTGCTCAATTAATTGTTGACATAAGAGATGACACATAAGTAAGTATTCCAATTGAGTGTTATAAGTATGTATAAGGACATTACTCTTTGTTATCAAGAACGTTATAGATATGGACTAATGCTATATGTATCGAAAATAGAATAAAATTTAGTACAAAGTCACATAAAGTACTTATCTTAACTTTGTTGATATTATTTTTATAAATACTATGCTATTTATTATTTATTCCCATATTGCTACAATTTAAAACTTAAATGTCCTTAATAATTATAGAAAAAATAAAATAAAAATAAATTAAATAAATTGGCTCTTAAAATTTAAATTTTACTCTACTAACGTACTTAAGTACCCAATATGATCTTACTAAACAGAATTATTATTAAAACAGCACTTTTATATCGGTGACTCATCAAAATCTAATATCAAATTAATTCCATTACATAATTTTCagccccttttttttttttgttttCTTCCACTTTTCGTCACCCACCCCCCGCGGCGCGGCGGGGGGCGGAAATCTCASACATAAATTAATCATCAAACATAGAGAGATTAATGCAAGCTTTTAACCAATATGCAACTGCAGATGGTAATAACAACATACAAGAGATTGTATGAAAagtttgaaaaaaagaagcACAGAAACTAGTTATTTTTTTGTCTGTCTTTTTCTTACATGAAGCTTGTCTATCAAAAAATATGAAAAAGAGAGTTTCACAAGCAGCAGAATTTCGAGTAGCCAGTGATTATTAGATAAAAATGTTTAGACATGACTTTAACGTTCAAGAAGGGAAGTAGAGAATAAAACTCTTTATCATTTAATTTCTTTTAATATGTCCATCATAATAATTTGCMTGATAAGTATTTTAGTAACAAAAATATTTTATTAAAAAGTGGATTTCTGAGTATTTCCGCTAATCCATTTCCACTCACACACACAATKCTGAACTCAC

>Cs1g18370

TAATGATATCCAATCTTTGAATAAATTAAATTTATCCCAAAAGAAGAGAAATTAAAGTGAATTTAACTAATGGGTTAACTGTAAAAACAATTAGTTTATTTGAATTTTTTAAAAACAACTAGAACTTTATTATAATTTATCCTTTTGAGTAGGACTTTCGCACTCCTAAGTTATTAATATCAATATTTAACTCCACTTCATCATTTATAGTCAACagaaattttttttctttttCTTTTTCTTTTTGCCCTTTTCTGAAAGAACTCAACTGGCAATTTGAATTGCAGGGTAATAGGGTATTATATTAATCTTCTAAGACGTTTGCCGCCATTGGAATCAAGTAGGCAACAAGTAATGTTTAACAYTTTCATSATTTATTTAAWTAAAAGAATTTTCTTTACCTTTCGAATACTTGAGTTTGAGATCATAARTWAAAAGCAAATTGCAAGAATATRTATGAACTAAAAGTAGCATCAAAGCAAAATTTTGCTGGAGGTAAAGATAAGGAATATATCGACCAGAGACTCGAGGGAAAGTAAATGCCATCCATACCTTGAATAGCCTGGCTTCTTGGACTCGAACAATAATTTTGAAGAGGAAACATCCCAAAACAAAAAGAATATATACYATGCGTAACAGCAAAGAAGATGTTAATTTGCMGAAASAAAAGATYGGATCAGCGAAAATGWGATYTGGAAAGAAAAAGTACATGGAATTGCTTGAAGGAAACTACAGATATCTTCATTAGAARCAGAATTTTGCACNAAGTAATAATGRATCTKGGAATCAGTCRGAGGAAAGCAGATACCATGACTAGCATCAAAGCAATATATTGCTTCCATCAAGCSCTTCAAGTAATTAAAGGATTYCTTAAYTACTGTCCTRTCTATAAATAGCCAAATGGAAGATCGTGGGCAGAATTATCACAACCAAAGCAAACCAAAACAAATTCTTGAAATTACCAACTTCCTTGGATCAAATTCGGTAACCATCTTTGACCA

>Cs1g21650

TGCCCGTTATCACAGATATCTATAAGTACTTGCTTATGCCATCCGATTTCCCTCATTTTGGGTGCCGGATAAACCTTTTACCTCTCGTTCACAGCATGAATTTAAATAATATCAACTGTTATTGGACTGATTGATTCATGCATACAGCAGAATTACAATAGCTTTCTCTGCAACATTTGGGAAATGATTTAGGTGTGCAAAAGGCACACAAAAGGTAAGGATTAGTAGCAGAGAATATTAACAAGTAAATGCACGTTATATCCTTGACGTATTCTTAATGATAAATCCAGGGAACATTCAAGGTATGGACGAGGTGCAATAGGAGCTGCTAGATTATGATTATGATGCGAAGAGCATTTTAAGATACCATCTACTGATCTACATCTCACCAACAGAGGTTACCAAGTTCTAACATACAGGTTTTAGCCACTATTTAGCAAAAAGAAAAACGAAATAGTTCTTGCTTAGCTCAATTTTCTAATTGGCAAATCAGATATAATCTGCAGTGGTGATCATCACATGGTTGTTCCATACCTCGGGACTCAATGATGGATCGGGGATTTGAACTTTACTATTGCTGATACTTGAATTAAATTTCTGGCCAAAATCTCCAGCTGCTGGCTTTTGTAGCAGATAAATGACGGAATACYCATACCTTTTCACATTTGCAACTATGAAGGCAAGAAATATATCCCCAATTAAAATTCAAGTTTCAATTACAGTTTCTGATGAGTTGGAATTTAGTRTGCAGTCATCTGGATTGTGCAAGAAGGAGGTCATACGGRTCcagagtaataagcCGAAGGAATGCTTAGCTATGTTCAAAAGATGGGACTTTGAAAGAACCTCTGTGAATTGTGATCATTTCCTGGGTCCAGCCCAAGTTCCAAAATCTGCGAMTTGCAAGATGAAAATCTTGTGTGTTTTGGTTTGCACATCGAGAGCCTGCTATATTTTCATGTCGCAATTAATATCATCTAATATTTGGACATGGCTAAGGT

>Cs1g15760

TTTTGCCAAGTCACATTATTCATAACTTGTATCAATTCAAaatcatttttttttgtatgaCTTAATTATTACCTGTCGAACAATTAAGGGATCATGAAAGACTACTTTTAAGATAACTataagttattacattatCAATTGCCAAATAGTAGTTTCTAAAATTTTAGTACTTTATATATTTTCTTCCCTACTTATACTTTATGCCAGTTATAAATTTTATCCCCACCCTTTTGATCTTTTAGCTATATTACTGAATACTAAATATCCTCTATAATGAACGTTTGTTGTTTCTATSGTTTTTATCTTAAACGAGTTGTTAACGGTGCAAAATGATCACATCTAATTAATAGCAGTAATTGTTTATAATCTGATTAAAGTGTCATTTCCAGTGAGGAGATAAMTAGAAACAATTCCCAATGGGGGAGAAAAAATGACGAAATTttggggaaaaaaaATCATASTAAGATTCACATATATGCTAATGAACCTTTTGATAATTTTAAATACTTATTAATTACTAATTAATTAACCTATCAGGTGATTGAGTACATAAAACGCTTACTAGGAATCTGCATTTTTCATCATTTTAGTTGAAGACATGATATAAATTCAGAAACAAATCTGCTTTTCTTTTTAACCCAAATACCGAATTAGAATCAGAATGCCAACCTAAGAAACTACTCAAAATGGAAAaaaagaaaaaaagaaaaaAAAAAGCAGCTTTATAATATGATATTGAAATCYCCCTTCTATCCTCCACAAAGTTGTCCTCATGTATTTGCATTTTAATCTTTCATCTACCTTTTGTCaataaatatatatatatatttagatACTTCTTCATTAATAATATTATTTATATATTCTTTCACTTCAGTTTCGGCTTTATAAATCCCTAATTGCTGCTGCATAATTTCACTCATCACATTAAATACATTTTCCTCTTAMAATTTGCAATTCCTTATCGCTTGCTCTCTGCAACATTTTAATTTTCTTAAAAA

>Cs1g25230

nnnnnnnnnnnnnnnnnnnnnnnnnnnnnnnnnnnnnnnnnnnnnnnnnnnnnnnnnnnnnnnnnnnnnnnnnnnnnnnnnnnnnnnnnnnnnnnnnnnnnnnnnnnnnnnnnnnnnnnnnnnnncaagattctctccttatctgatcatatcctgaacaaataactcaattgctgattggtagttaataaaaaataaataaaagaagttaaatcttactcatacactactactaaaagatacatgacagaatatcttgaaaagctcagaagagatcagatcaggagagaatcattttcctatnnnnnnnnnnnnnnnnnnnnnnnnnnnnnnnnnnnnnnnnnnnnnnnnnnnnnnnnnnnnnnnnnnnnnnnnnnnnnnnnnnnnnnnnnnnnnnnnnnnnnnnnnnnnnnnnnnnnnnnnnnnnnnnnnnnnnnnnnnnnnnnnnnnnnnnnnnnnnnnnnnnnnnnnnnnnnnnnnnnnnnnnnnnnnnnnnnnnnnnnnnnnnnnnnnnnnnnnnnnnnnnnnnnnnnnnnnnnnnnnnnnnnnnnnnnnnnnnnnnnnnnnnnnnnnnnnnnnnnnnnnnnnnnnnnnnnnnnnnnnnnnnnnnnnnnnnnnnnnnnnnnnnnnnnnnnnnnnnnnnnnnnnnnnnnnnnnnnnnnnnnnnnnnnnnnnnnnnnnnnnnnnnnnnnnnnnnnnnnnnnnnnnnnnnnnnnnnnnnnnnnnnnnnnnnnnnnnnnnnnnnnnnnnnnnnnnnnnnnnnnnnnnnnnnnnnnnnnnnnnnnnnnnnnnnnnnnnnnnnnnnnnnnnnnnnnnnnnnnnnnnnnnnnnnnnnnnnnnnnnnnnnnnnnnnnnnnnnnnnnnnnnnnnnnnnnnnnnnnnnnnnnnnnnnttatatATATCATAACTATTAAATTATTATTATACGATTAATGGATGAATGTGTTAGATACAAATTGGCATCTTTAGCTTCAATCGGAATGTTTTGGTAACTTTGAAGA

>Cs1g08460

tgggaaaacttatttacttgtctcatactagaccagacatcgcttttgcagtcagtatagtaagtcgcttcatgcatcatcttcgggaagaacatcttgaagctgtttacagaattctgagatatctaaaaagtactccaggaaagggacttttgtttagaaaaacagagaatagagggattgaggtatacactgatgctgattgggcaggttcagttatagacaggaagtctactactggctattgcacttttgtatggggtaatttagtgacttggaggagtaaaaaacaaaatgtggttgcacgaagtactgctgaagctgaatttagagctatggcacatgggatttgtgaagtattgtggctgaaacaagttctagaggatttgagnnnnnnnnnnnnnnnnnnnnnnnnnnnnnnnnnnnnnnnnnnnnnnnnnnnnnnnnnnnnnnnnnnnnnnnnnnnnnnnnnnnnnnnnnnnnnnnnnnnnnnnnnnnnnnnnnnnnnnnnnnnnnnnnnnnnnnnnnnnnntatctgcctattgtttgttccaacaaaacaacaaacagcagatatcctaaccaagggactcctcaaaactaactttggacatcttgtcagcaagttgggaatgattgacatctacgcaccaacttgagggggagtgtcaagaagttaggatttttcagttagtttgaaagtcttatcagaataaagttttagataattagtttagttttaaagtcttatcggaataaagttttagatannnnnnnnnnnnnnnnnnnnnnnnnnnnnnnnnnnnnnnnnnnnnnnnnnnnnnnnnnnnnnnnnnnnnnnnnnnnnnnnnnnnnnnnnnnnnnnnnnnnnnnnnnnnnnnnnnnnnnnnnnnnnnnnnnnnnnnnnnnnnnnnnnngcacaattggtgatccaaaggatagaaacaatacaagAAGATAActctctAACAACAGGACCAAACTTCAAGTTTACTGGGGAG

>Cs1g08350

ATTTTTTAGAGAAGTCAGTCCACATgcctgaaacaaCCAACcaaggcgcaaccCGAGAAAGCAGAGRTTCAACTTCTTAAATTATATTTAGATATTAGGTAAATTTCATCCTACCCTCAGTTAAAATGGCAATCTTCAattcacttcactaAAAGTATGAAAATTTTAATTTATCTCCAAAATATTATTATTTTTAATAGTTAACCGACGTTGACTTAAAAATAKAAAAATGTCCCTGGCCCTAACATRAGTCAAATTGAACAGTAARAAAGTTAGTTGGTTCGGATTAGGGYCATTTTCATatttttataagtCAATGTCGGTCAACTATTAAAAATAARGGTATTTGGGGGGTAAATTAAAYTTTTCATACTTTTTGTYGATAAATTYAAGTATGTCAATTTAATAAGGGGTTAGGAAGAAATTTACCCTTAGATGTATCATAATTATACTATTCGGTAAGCTAATTRAGTTTAGTTACGTGCTTCAGTATGAGGAGAAATTCTAGAATACATTAGAAATATCGTGTTAATCATATAATAAGTAAATCATACTATGAGCTTATGATATGTGTGCATTTARTTTGAAAAGCTATCACACAAGTGGGTTTATATCAAATCTAAATACGCATGTCATACATGGGTTAATTAGTTGTWTTYCATTTAATGTATCTAGTATTTCTCCAATATAAAAAACTAAAGGGAYGCTTTTTttaattatttttTTTTTGCCGACCTCGACACTACAGGGGAATTTAATAATTAAAAATAAAATGGATACCTAGTCATGTGTCATAATAGTCATGTGACATAATGAATCTTATCCCTCATGAAATGCATAACACTACAAGAATGCAAGCAACTagccaaacaaaaaaTTCTTCCCACTTCCCAAATTTTAGTTAAACAGTCTTTGAATATTCATTGATCACGACACTTTGCAAACCCTTTAAATGACTCTCGGATCATCTTTCTTCTTTCCCTGCAAAAGTGCTTTCTATT

>Cs1g08920

aaatgggtggttggattttgtggtgaaggtgacttagattatcctaaaattggaattgaaatggcaagaataaattgaagagaaattctattaaattgacgcacttgggtatctggatccgtatcaacatgaatcatgggctaaataatccacattaatgccaattaaatcatgaggggggaatccacacctcatgaaccacgctttaatcaatatggtgctaagggcttatcgtgccaaataataataaccttgtactagggagccggtgaaaccaaggcggtactagacaagattaatattatttgtcaacgagaagtcaaaacatccacaaaaactagaggggaagagaaaataaatttaccaaataaagcccatgacacatgttgagacttcaccttcaacccaagcttgaaagaaaattagccactcataattgaactaggggcaaaatgagaatttattaaaatacgaagaaaatacaagatgaaaagacgcggtttggttgaaaataatcctgtgtgatgcatgtatcgtacgcgagatttttcgtccactgatatgctgccacgtcaccatcaacagtacataagaattttagcccaacaggatcgtgacacctcatcagtcaatgccacatcatcggtcaatgccacgtcatcgtccgtctatatgaacagtgtcatgaacagtaacgtatacagtactgtacacgtgaatagtaccgtacatgtgaacaatgccatttttccttttatgctcctcctaaggttttcgaccgtcttgagttcaaaagtgatgtccattttggcatctgatctctcgtttattgtgaaatgactatgatgcccctaaaatacataaaatacttaattaaaataaaacaaaagnnnnnnnnnnnnnnnnnnnnnnnnnnnnnnnnnnnnnnnnnnnnnnnnnnnnnnnnnnnnnnnnnnnnnnnnnnnnnnnnnnnnnnnnnnnnnnnnnnnnnnnnnnnnnnn

>Cs1g23740

tgcctattaaatcttggatagaaatggttgaagaacatggtgcccattacaaaaccacttcttctgatgaccaagtaaaacaatggatgagttccattacaaagtcccctgagcttatgctcgccttacaaaacctttcccaaagccaaattttctcaaaagaagaaaaagaaaaccctnnnnnnnnnnnnnnnnnnnnnnnnnnnnnnnnnnnnnnnnnnnnnnnnnnnnnnnnnnnnnnnnnccaaattgtgctttcccaaccatcaccttcaaagaaaacctccgattggtttgataaaacccattttcaaaatattctttctttagaagatggattttaccatgctgatcctttccaagcaatttcaaagtttttccctaagggctggttttttaaaccatgggatttaacaaaannnnnnnnnnnnnnnnnnnnnnnnnnnnnnnnnnnnnnnnnnnnnnnnnnnnnnnnnnnnnnnnnnnnnnnnnnnnnnnnnnnnnnnnnnnnnnnnnnnnnnnnnnnnnnnnnnnnnnnnnnnnnnnnnnnnnnnnnnnnnnnnnnnnnnnnnnnnnnnnnnnnnnnnnnnnnnnnnnnnnnnnnnnnnnnnnnnnnnnnnnnnnnnnnnnnnnnnnnnnnnnnnnnnnnnnnnnnnnnnnnnnnnnnnnnnnnnnnnnnnnnnnnnnnnnnnnnnnnnnnnnnnnnnnnnnnnnnnnnnnnnnnnnnnnnnnnnnnnnnnnnnnnnnnnnnnnnnnnnnnnnnnnnnnnnnnnnnnnnnnnnnnnnnnnnnnnnnnnnnnnnnnnnnnnnnnnnnnnnnnnnnnnnnnnnnnnnnnnnnnnnnnnnnnnnnnnnnnnnnnnnnnnnnnnnnnnnnnnnntatgtacaaatttcttccttccatgggtatggatgtggaaccttcggcttcatacccaagattcccaactcatccttcaaagaacctttaaagtaaaatggtggtctaaatttgnnnnnnnn

>Cs1g07510

TGTTATAGTTCTTGGATTCCTTTACTCGAGAGTTGAAGATTGTTTGACCATATTCATTCGTTTCCTGCAGATTACACGTTTTTTCCTTTTCCACATTTTTGTTGTTGAATTTTCAAAATTAGCggacaatttatataraCACACACACACACACATGCATGCATGTTATGACTTCAGTAGATATAGATGGGTACATGAATGTCATAGCGCACccgttgtcctaGACTCTACTAAACAGTAGATGAGAGTAGGTACCATTCGTCTACTCCTGTGTTCTATGTAAGAGAACCACAGCCACTTGATTCAGGATGAAAGTCATAAAAACTTGGAGGGTCACCCTTCATTTTAACCCAAAGGCTATGAGACTGAGTAAACTTTCCCTACACCAAAGCATGGTMTAATTATTTATGGTGAAAGGACAAATGGTGGCCATGAACAGACTACCTGTCTACTCCTGGGGCCAAGCACTGGATATATGAGCCCTTCGACTTGAGCTGGTCCAATCTTGCATAACTGTGAAGTCAACACCAACGGACAAAGAGGCATTAATAGTTCAACGGGAAGAACAAGTCTTATAATCAATTTTGGCCGACTAAAATGTATTGATGATCATAAAGATTAACATTTTAGCCTCCAAATTTAGCTCGGCTAATTGTGAGTGCAATCACATGAGTTTRAGTGCTAATAGGAGAGGAGTTGTGATAAGTGTGTGCTTAGTGAGTGAAGTTTTTCATCTCATCCAAGCCCTTAAATATTAAGAGTTAATAGCTCGAGTGAAATACGTACCGTATGATTATGAACAAATTTAAGATACAATTGAAACGTGTATCACacggttgtgcgAACAGACACATGGGAGGCTCCAATCTACCCCACAATTGACACTACATTAATTGCAATTGCATGATCAACGTTCACGTTCTCTGATTTTGCCACCTGTAAAAAGAAACAGAAGACCAAATACTAGGCGTAGACTAATAAGCAGACAAACAATAACTTCA

>Cs1g25770

gggaggAAATAATCATGGGATGTTTATACTTTGTTGAAACGTTGGTTAGATTAAATCATATGARGGGTTAAGGCYACCCACTAACGCAGTAGTGGATTTTGATAATTGTGCGGTCATGTCACGGAGTTAATGAAGCAATTTTGTTAATTAATGCKTTTTCAATGAGTTCACAGTCCAATCCAATATATTtttgggttataaAACATCTACGTACTTGTATTAATTTATTTATATTATGATAAGCTTTTATAATTGCCTTAACTATGGTAATGATatgtataaataataCACACAGATCAACAGATAACGGATTATACAAAAGATAGCCCCTCGTTAATTATTCTTTTTCGCCAAACAGAAGTTTTTCTTTGTATGATCAACGTAGTTTGTTTGTTTGAGATTAGGCCAACAATTGTCAACCAAATAGCTTGGTCTCTTTCATGATCATGAATAACACCAACTAGAATTCGTGAATTGTTTTATGAGCAAAACAAATCTTATACAAGATTCATACAAATATCTCACGAGAGACTGTATTATTAATTTTTTTTATATCTGATTTACGTTATTAATTTTGTATCCGTAAATAAAGGAAATTCTGATGCCACGTGTTAATACACTACAAATACAAATCGTTACCCGCCAATATCGAGTTTCTGTTAGATGYTTGGACTTGGTCAGCATCCAAATGAAGCACCTTGAACGAGCAGTGCTGACATGAAAGAAGAAACCTGAACCAAACGATGAAATCAATTTAAAAGCWAARAMTTTATTGCACTCAACGCAAACYATTTCTCCGAAATATACTCTCAAGCTGAAAAGATTTTCACTCCACTGTCTCTGACACTGTCACTATGATTCAGCACATCGTAGCTCCATGAATGCTGCAAACCAACTTCATTGATTCGGATTCTGACTGAAACCCACATCCCCAGTTTTGATTAAACACCACAGACTGGTCTGCTCctgttaagtagtagtaacacaCTGACAGTACCACT

>Cs1g10430

tgaaattacctcaacatttatgcgtgagggtaccactttctttatcttttgtttacttttcttacaaatagttgactttttcctcttgcaaannnnnnnaccttATTTCGagttgctactggatcatgTATGTTTGCAGAATTTTCGATATTGACAATACCACTTTTGTGCCAAATTATAGTTGGCTACTTGGCTCTAGAacttacttttttttCTGGTAATGTCTTCAATgtgTtcttgtccaatacaaaactatattacaactttctgcacatcctaaaccaatgcttgcaaccttaataaaaagtagtcaacattttttgtatagcttctattcttttgtggaaatcctttcaaacatctttccgccatctttgtaggatgtacttttttaaaagtacgaaaatattatttcgagtcaagactacaattgaatgtctgcaaacaattcctctaaatttaagcattgannnnnnnnnnnnnnnnnnnnnnnnnnnnnnnnnnnnnnnnnnnnnnnnaacatgaaccgttggattccttaatttgcagatccaacgggtgtggtgaaacataacgtaaaacaaggttcaggtagtggttggatattcatttttaaaaacaaaagaatggctacctgctaaaacggttgttgaaaaaaggaagcaaaggggttagccggtaaagaaaatttgagatggaaatgatgagtgacgagtggtcgctggaaaagccagaaaattgaagaaagaaagtacagtatctaggttttgaaagaggaaagcattagaagcagcaaacgagataaccaaattcgtatttcattatttcatatcacaaattagggaaaccaaaaaaattgcagccccataaaatcgaagctacaggcgctggaatgtggcagaatttggtaaaatgtgaagcatcatcttgtgaagttgtcttcaccatataattttcgtaacatcattccttgttatgggtgggtcgtgattattggaaca

>Cs1g19940

CCTTAAAAGATTTAATTTTTATGACTTATTTCACCTAgattAttaagagcaaAAAGGTAGGAGAAAACATGTTCGTTAATTTAACTCCATATTGTGGCCTAATTTATATTTGAGCAGGTGATTTGTGGCATATTATTTCTCTACATTTACATGCCAAAGCTAAATGCTTGCTTAATATTTTCGAAAGCCTGTTGGCAGAATATAAAAGGCATTAAGGAGGTATTTGAGGGCAGAAACGGAACTTAATTATATTATCTCTTTTMAGCTGGACTATTGGCACCCCTCTAAAAACTCTTTATCACTAGTATTCCACTTAATGCCTAAATTACCCCTTGTTTCTAATTTATTAATTTTTCGATTTCCTAATATTTTCTCACTACTAAAATtattttagagtcttaTTATATTAATCTCCACACAAAATTTATGAGGGtgataaacaaattAGTGAATTAGATTCAATATCTTTAGAATTATGATGCAAAAAGTTTTAGTATGATTATGAAGCAAATCCGAATGAAAAATTGTGGAAAAGATCAAAAAGAAAACTCCAAGATTCAAACAATTCATTAAAAAAATACCAAGAtATATatTTTATAATTTGTTTGATGATGAGTtacaaatttttaagaaGTTGGAAAGGGGATAACAGAAGATACTGGAACTAATGAGGTGGAATCAGAGRATACTAAAGGAAGTCATTAGAATTMTGCAGGATGCTGTGttttaagggaattttggagggttgctaatagtctctctctctctctctctctctttatttatgaataccgatcaaaattggacctcgatttgcggcatattaaacctataacagggattagagttggttcCCTTAGACCATGAAAACAcacagacatttggcatttggggaagGACTTGGCGGGACCCTGTTAATTTGATTGGTGTCCTCTGTCCTCACTCACTAATACTTCAAGAGAAATAATGatatccctgctgcctttgCCAGCGTGTTAATC

>Cs1g25780

AGTGCAATAAAMTRTTWGCTTTTAAATTGATTTCATCGTTTGGTTCAGGTTTCTTCTTTCATGTCAGCACTGCTCGTTCAAGGTGCTTCATTTGGATGCTGACCAAGTCCAAYCATCTAACAGAAACTCGATATTGGCGGGTAACGATTTGTATTTGTAGTGTATTAACACGTGGCATCAGAATTTCCTTTATTTACGGATACAAAATTAATAACGTAAATCAGATATAAAAAAAATTAATAATACAGTCTCTCGTGAGATATTTGTATGAATCTTGTATAAGATTTGTTTTGCTCATAAAACAATTCACGAATTCTAGTTGGTGTTATTCATGATCATGAAAGAGACCAAGCTATTTGGTTGACAATTGTTGGCCTAATCTCAAACAAACAAACTACGTTGATCATACAAAGAAAAACTTCTGTTTGGCGAAAAAGAATAATTAACGAGGGGCTATCTTTTGTATAATCCGTTATCTGTTGATCTGTGTGtattatttatacatATCATTACCATAGTTAAGGCAATTATAAAAGCTTATCATAATATAAATAAATTAATACAAGTACGTAGATGTTttataacccaaaAATATATTGGATTGGACTGTGAACTCATTGAAAAKGCATTAATTAACAAAATTGCTTCATTAACTCCGTGACATGACCGCACAATTATCAAAATCCACTACTGCGTTAGTGGGTYGCCTTAACCCRTCATATGATTTAATCTAACCAACGTTTCAACAAAGTATAAACATCCCATGATTATTTcctccccTCTTATTTAAATGGATAATTRCCTAATGGTCCCCATAATTTACAACAATAGTAAATATGTCCACAAATTTAATKTTGATTTCGAGTTCGTTCCTAACCCAGTAAGCGcaagcaaaaattaaacaaaaaaaattaaACAAAAGAAAAACTTAGGAGTATTTTGTTATTTWWWWAAAAAAATTATRGGGACATTAYTATGTAATMCRGACAATGACTGTTTAC

>Cs1g25170

AAGTTGGTTTtgTTTTTGTAATGGTGTTATACAGACAAATTTATAATTAAAATTCTAAACTTGTAATTAAAATTAGAAGATGAACAACAATCTATTTTATATGATATATCAAGTTGGTTTGTAGTGAAAATGAGAAAACAGATGTTTAaatcttgtgctcgaacgtaATAGACAAACAAAATTAcCTTCTATTAatttaaaatacagctacagaaataatggcacgtggatggtttttgtacaaattgattactattattactattatnnnnnnnnnnnnnnnnnnnnnnnnnnnnnnnnnnnnnnnnnnnnnnnnnnnnnnnnnnnnnnnnnnnnnnnnnnnnnnnnnnnnnnnnnnnnnnnnnnnnnnnnnnnnnnnggatccttattattattttgataagaCAAATTGAATATTAAAATATACAAATTTATTTTTTTAATGCAAAAGTTTGTCCTCATTTTGTCAGTTCAGTCCACTGTGGTTACATTAAGATATCCTGGTTACTTTTCGGTTGAAGAGACGTATCGTAATGGATTTTAAGCAAATTTAGAAATTAATAAATGATGGAAGAGCTTACAGGGCTGTACACGTTTCGACCTAAGCTACGTGCCAAATCAGTGTCCGCTTTTACTAGCTTCATCTTCACCGTTAATTGGCATCACGGAAACGCAAGACAAAAACATCGACCGTTGGTGATCAGCGATTAACGTCCACATCAAAATTTTATGACGTCAGCGAAAATAGGAGTAAAAAaaatttgannnnnnnnnnnnnnnnnnnagtcatcaaaatttgatgacgtcagcgaaaataggagtaaaaaaaaaaaagctcgaTGGGTTGGCTGGAGTTGGTGCCCTGGTGGTGTCTCTATTCATTGGCTTACCATTTATCATTGGAGCATCTTTTYGTATTTCATTTCTCGTGGTTCATTTTTTATGAGCGAAAAGTGACCATTCGCTTTATTGTTCCCTKAaatccttttatatctTTTCAC

>Cs1g02850

AAAACTCATCTTGTTCGTCTGGCTTCTTCCTCTATTAAATCATGGTTGATTAATCGGTgaaggaaaaaaaagaataaAATTGGAAACAAATTGATTTGTAGGAAAGTAAATTCCTGGGCTATTCTTTTTATGAGTAGAGAGATGGGATTCACCCTCGGGCAAGAAAATGGATAGAACCAAATTTGGTCCATGGACGTGAGCTTAGTCCATACAGTCAGAATTTCAAAGGCAAAACTAAAAGTAAAAAATAAAAAATGGTTatagataattagannnnnnnnnnnnnnnnnnnnnnnnnnnnnnnnnnnnnnnnnnnnnnnnnnnnnnnnnnnnnnnnnnnnnnnnnnnnnnnnnnnnnnnnnnntaaaacttatgcttaaaatagtactaccacttaacacataatttatttgtttattatttcTAATTATGTATGCCAAATGCATATTACTGTTATTATTTTTTAGATACATAAAACATGAAATGATATCAAATGASTACTTAGGACTTGAATTAAAAAGACAACAACTCCAAAATACTAATTTTCTTTCACATTTTTGTTTTAAGTAAAYACCAATTCAAAAACAATATGTCGAGTTTCAAATAGTGTTTTGGATATGGGTAAATTTCGGATTATCTCATCCTAAAATGATAATTTTCAAAATATCCTCKAAATTATTTTAAACTTCAATTTGctctattttttttcaaaggATAAGAAGAAAATTAGGGGTTAATTAATRAAAWWWWTTTCATCTTCTCTATAAAAATGAGGATAAGTTGAGGTTTACAATCATTTAGAGGGTATTTTTAATATTATTATTTCATGAGGGGTTATTCCAAAAATTAACCCTTtatatatTTTTTTTAATATTGTTTATaaaaAATATTAATAATAAATCTAtttatttaaaaaaaatggtttcgaaaattgagttacagaaacagttactgagcactaaagccttaTTATAAAGAATATACTTACCGGGCATTATTAt

>Cs1g10800

CAATTGGGTTAAAAGAATAAATATATTGTTACTGTACTTGAAAATCATAATGTTGGTTTTAGAAAACAATTTTTttagtatgatatAAAGCTGAAAATCGTTCATCTTTAATATTAATAATATTCTTCTTTTGTTAATTTATTAATTCtttataaaaaaaaaaattgtttGCTAATATAACTGAAAATACATAATGCTCATCTGCACTGCATCGTGATTATGGATTGGCTGGAGCAAAGTTGCTGCAGCTTGAGAGAGATGAACGTTGATGATGACCATGCTCTTGACAAAATTCAGCTTAGTTCTGCGAATAGCTCAAGTTTCCATCAAAAGATTAATCAAAGAAACTTCCCCTGGTAAATTAAAGCTTATTAATTTCTTTTAATCCTTTCATTAAATTTGATCATGTTATGGATTTTAGCCATAGAACATAAACTACAATATACATGGtatgattttattcccTATATATAAATGCAATTAGTAATTGGGTGCATTGAGTTATAAGCGGCTGATTGCTGAAATTGCACTCATCGATTAATTTGACGGGCTTCATTTTGCTTTGAAGAAACAACATAACAAAGATGAACTCACAAGTCACGTCAAGTGTAAAACTATATAGTTAACTTATTTGATGTGGCCGGGTCGCCTAATATTTATGTGGATTTAATGATAAGGATTGAAAATGTCTCAACTCTTAGTAAATGGAGTTGAAGAGTGCAGTCGCGTCCCTaaTCCTTTATAATTATTAAAGGATTATTTTGTGGTAGATTTTGTAGGTCTATCCAATATTATTTtgtgatgatatgtctatacaatagtaccttgggnnnnnnnnnnnnnnnnnnnnnnnnnnnnnnnnnnnnnnnnnatattaaaaattgaaggtcttgctctTTTGCAAGTTTCGGTAGTCTTGCTCTTTTGCAAGTTGGATTGGTTTCATTTAAAATTCGGTCTCGGCTGGACTGGTATTTTATTTCTGCTCACCATAAGAATTA

>Cs1g23380

nnnnnnnnnnnnnnnnnnnnnnnnnnnnnnnnnnnnnnnnnnnnnnnnnnnnnnnnnnnnnnnnnnnnnnnnnnnnnnnnnnnnnnnnnnnnnnnnnnnnnnnnnnnnnnnnnnnnnnnnnnnnnnnnnnnnnnnnnnnnnnnnnnnnnnnnnnnnnnnnnnnnnnnnnnnnnnnnnnnnnnnnnnnnnnnnnnnnnnnnnnngatgtgccagtctatttgtttcagcacatgcccgtcttcggtcataaGATTTAGTGATTAAATACTTGACAAAAGATGGGCGTACGTTACCCGTAGTCTTGGCATATGATTATGACATAATATAATTATAAATATAATATATTATAATCCGACATTAttgtATAATAAaatataacttatattatattatattataatacaatataatataaatttttaatttaattataaagtaactttcatttattgatgtcgannnnnnnnnnnnnnnnnnnnnnnnnnatcttggtaaataataaatgattaaataattaaaatactttatacatgttttgagtaaaataattaaaacaactttatacagtaaaagtaaaataannnnnnnnnnnnnnnnnnnnnnnnnnnnnnnnnnnnnnnnnnnnnnnnnnnnnnnnnnnnnnnnnnnnnnnnnnnnnnnnnnnnnnaaatttattataagttattgaataacaaaagtaaaatgattaaagttaaaatatgaacaacTTTAATACAATATAGTTTAAATCATCCCCACATGGATTTATTTTYTGTTCRTTTCATTATATTAACTACTTTCTTAATGCAATACAATGCAACTAAAAATTATAATAAAGTATGAATTTATATAAAAATAATGTTTTAACGCAATACAATATAGTATAAGGCAAGCTAGCAATGGCACCATGAGTTTATTATATGATTTGTTTTATAAAAAATCAGGGTTTAATGAATAAAAGCCATCGTGGGCCTTAAATTGAGTTAGTTGAGATGYATGGG

>Cs1g13840

TTTTTTCTTTttctTTTTTTCATAGATGCTTTTCCTTCTTAAGTACTTTCTATTGTTTGTAGAGGACAAGATGGTCCTTTCAGTTTTTGTTCTTTCCCTTGTCCAATGCAAACACTCACTCTCCTTTCATTTCTTGTCTCCTTCCATCCACACTTCTCCCAACACCACTCAACATTTAAAAGAAACCCACCACTCTCTTCTTAGTCTCACCCACATTGTCCCCATCTTCTCCATCATACTCTCCTCTTTAGTCCCCCTTCTTTCTTGTATGCACATACTTGTCCTTGATTTGGTTGTTCTCTTTTCTATTTTTCTTGTATATTCTAGATTtaatttatattttgtagcaactagGATAATGATAAATAAATTATATCTTCCTTAATGAATACCTATGACGTATGTATTATCCTATCACTCAGCATCTCATCTTTCCACATTATTTCCAATCTTTTGATTCCCTAAATTTGAAACCATTTTTTAATGATCAATattattttgttatgtaTTATATGAATATAAGTCAAACTCCAGGTCCCTTTAAACATCTCAATTTTTTTAATTAGTTTAAGCAACTAACATTCTCTCATSCATGTATGTGTGTGTATGTATATATATATATATATAGAAATTATTCAATCTAAAATTATTATACGTGTAACAATTTTTTTGAACTTCAACAAAGTATATGATGTGATTCTGGACCAACACTTATATATATATACATAATTCTCACCCATTTTttttttttttttgaGGCGCCCCTTCCTCTTTCTCTCTCTCATGATCGTCTCTATAAATACTCTACAAAAACCATCATGTCTCTCATCTCATATTCTCCCTctctactcatagtcatccaacTTTTTGCTTACTACTATTGCTATTTCCTAGCTAGTACTACATACTAAGCTTTTATTGCTAGCTCCTATTCCGTAAAACATCTAAAATCCAAGACACACACTTTTTTCTTTTTCCTCTTCAAACTTATTAATTTCATC

>Cs1g08790

aacaagctacttcttttttatttcttttgtttctttttttagtagactgtgctaagaaaataatttgacaattaaaagtaaactttcagaactagctagnnnnnnnnnnnnnnnnnnnnnnnnnnnnnnnnnnnnnnnnnnnnnnnnnnnnnnnnnnnnnnnnnnnnnnnnnnnnnnnnnnnnnnnnnnnnnnnnnnnnntaaatgagaaatatagcagacatattgttagaaaaagattgatgatgttgTAAAACACTTGAAAGCTTTTTTGAATGGGAAATAATGCAGTACCGACAAAGAAAATTAAGAAGCCAAATCactattacaatnnnnnnnnnnnnnnnnnnnnnnnnnnnnnnnnnnnnnnnnnnnnnnnnnnnnnnnnnnnnnnnnnnnnnnnnnnnnnnnnnnnnnnnnnnnnnnnnnnnnnnnnnnnnnnnnnnnnnnnnnnnnnnnnnnnnnnnnnnnnnnnnnnnnnnnnnnnnnnnnnnnnnnnnnnnnnnnnnnnnnnnnnnnnnnnnnnnnnnnnnnnnnnnnnnnnnnnnnnnnnnnnnnnnnnnnnnnnnnnnnnnnnnnnnnnnnnnnnnnnnnnnnnnnnnnnnnnnnnnnnnnnnnnnnnnnnnnnnnnnnnnnnnnnnnnnnnnnnnnnnnnnnnnnnnnnnnnnnnnnnnnnnnnnnnnnnnnnnnnnnnnnnnnnnnnnnnnnnnnnnnnnnnnnnnnnnnnnnnnnnnnnnnnnnnnnnnnnnnnnnnnnnnnnnnnnnnnnnnnnnnnnnnnnnnnnnnnnnnnnnnnnnnnnnnnnnnnnnnnnnnnnnnnnnnnnnnnnnnnnnnnnnnnnnnnnnnnnnnnnnnnnnnnnnnnnnnnnnnnnnnnnnnnnnnnnnnnnnnnnnnnnnnnnnnnnnnnnnnnnnnnnnnnnnnnnnnnnnnnnnnnnnnnnnnnnnnnnnnnnnnnnnnnnnnnnnnnnnnnnnnnnnnnnnnnnnnnnnnnnnnnnnnnnnn

>Cs1g02550

tcagaacaaaaaggagagtaatatccataggctgaaatagaaattgggaagaaagaaaacggcaccagtgagttccattaagattgatcctctctatctaagttgttgaacacttttgattttctctgttgttcttccttatctttcaattgtatttgaaattcgaactaatttgggtaacaaaaaaaaaaaaaaatgaaagtaaatcgagccctnnnnnnnnnnnnnnnnnnnnnnnnnnnnnnnnnnnnnnnnnnnnnnnnnnnnnnnnnnnnnnnnnnnnnnnnnnnnnnnnnnnnnnnnnnnnnnnnnnnnnnnnnnnnnnnnnnnnnnnnnnnnnnnnnnnnnnnnnnnnnnnnnnnnnnnnnnnnnnnnnnnnnnnnnnnnnnnnnnnnnnnnnnnnnnnnnnnnnnnnnnnnnnnnnnnnnnnnnnnnnntgatttgtcccacgttactttttctctgccaagtttattccatgatgattaaaatctctctggattcttcgtcttgataatcaatttctaacttatcttatctgactattgcatcaggttattagtttacttgtcctcgtactccgggcatagaacgggcaatccaagggactaattgttgcatctattttcctgaacacttttgttgggttcaaaaacctaatatgatcataattgaaatatcattcttcgactttgattatacatgtgtcgtatcctcaaagtgttccaacacgacatggatttagagaaatctccctataagaggtcatcctgcttatgaggtaaggtcattaatcaataatcannnnnnnnnnnnnnnnnnnnnnnnnnnnnnnnnnnnnnnnnnnnnnnnnnnnnnnnnnnnnnnnnnnnnnnnnnnnnnnnnnnnnnnnnnnnnnnnnnnnnnnnnnnnnnnnnnnnnnnnnnnnnnnnnnnnnnnnnnnnnnagctttcgcatcaataaatatagatatgtgattgatagtaaataacggaatagtt

>Cs1g15960

tcattattatgttcaaaacacattatatatatagtacataccctgatttttgttgcgggctgtcacgcactcgcacgatgggccaaggaattttcaatataacctcactacagtctggatgtgtttcgtgatatgctatcttctgaagtactagaggtatcatgtctgcaacagnnnnnaattatctcaccaaaattgaatcctttgttaaatctatcatggagtcataaatggaaatcttgcccccaagtatatcgaccacaccgagaatccaatgatcattcatgaatattggcaaatacacctatgaaatatagacaatattcttaacaagtaacggcaaatatatctacgaaatgcggtaactgtttttatctacttgcagtgcatatgatgtaaaaaactaagcatacaatgtgaaacttacagtggaaacatttttccaaggagtggcacagtccaattttgcaccattagcataatcaatgcagcaacagagnnnnnnnnnnnnnnnnnnnnnnnnnnnnnnnnnnnnnnnnnnnnnnnnnnnnnnnnnnnnnnnnnnnnnnnnnnnnnnnnnnnnnnnnnnnnnnnnnnnnnnnnnnnnnnnnnnnnnnnnnnnnnnnnnnnnnnnnnnnnnnnnnnnnnnnnnnnnnnnnnnnnnnnnnnnnnnnnnnnnnnnnnnnnnnnnnnnnnnnnnnnnnnnnnnnnnnnnnnagagagagagagctttgtatctttgaagaagaagagacagtaatcaattggaagaagaacgaacggtaatcaattggaagaagaagagctttgtattcacctctagcttcaactnnnnnnnnnnnnnnnnnnnnnnnnactgattgtgcaggataatttggatgatttaagtttttaattagggtaatttcgtaactgcacataaaaaaaggcatatatgagaaattttagagtnnnnnnnnnnnnnnnnnnnnnnnnnnnnnnnnnnnnnnnnnnnnnnnnnn

>Cs1g09800

tgaaataaatgaaaacaaaataaatgaacacaaataagtgaaagaccactattaaattttttacagcgtgatcaattgtcaacagttataaaataaaaataatagtaaaaataaatttaaattaaataaatattgttgtaaataacataaattttttttcttatttataaaaataattcctccaacattgaaggatcatcatagaaannnnnnnnnnnnnnnnnnnnnnnnnnnnnnnnnnnnnnnnnnnnnnnnnnnnnnnnnnnnnnnncaagttttcttataataatttttttatcatttgactaataattttcttatatatttattataagaaaattttcacaaaataaataagtataaaaaaattttattattaatttttttgttcatatcaaaacatatatttcttatttttaatgctttattttttannnnnnnnnnnntcatgtgatttatttaatttagaataaatataaataaaagagtagttgaaaataagggtaatattgtaaatttatactattatgggggtttttggccattataaaagcagnnnnnnnnnnnnnnnnnnnnnnnnnnnnnnnnnnnnnnnnnnnnnnnnnnnnnnnnnnnnnnnnnnnnnnnnnnnnnnnnnnnnnnnnnnnnnnnnnnnnnnnnnnnnnnnnnnnnnnnnnnnnnnnnnnnnnnnnnnnnnnnnnnnnnnnnnntaacaattaataattattataattttgatataattaaattttattaaaataaaaattattttgataataaaattcaaaccaaacccaaatggtttggtttgtttgggttaaaaatatttgggttggtttgggttggatccaaatttttatggtttggtttgagttgacttaaaatccaacccaaaccaacccatgcccacccctaGTCAGAACCATTTGGAATAGGGGATATCACACAACAAGCTTTTACACATGCATCATTTTAGGTACAAATTCTCAACGTACCTATAA

>Cs1g15780

CCACTCYTAAAGGATTTCTCGACATGCTAGAAGAGGCTTCTTGTGATGAAGAAAATTCGGTGCTTGATGGTTTGAGATGACAAATGTTTCTCTTTTCGATGTTCTGTAAATGCYGGTTTTTGTTGTATGATTTTGAAGTCTCYGAAAATGTCTCTTGTCATTCTACCACKTTGCAATAGRAACTCACTAAAACATACACATTGTGGAGCTTTAAATTGAAATGTCCAATCTTAATAATCTAGCTTGCTGCTTTTGCCTCTGCCCTTTTCTTCCTTTTTATTTCTTCACTAATCTGTTTTTATTATGAATTAAAATTCTGAAAGAGATCTTTTGCTGTTGATGCTGTTGTGGTAGTTATGATCTTTGATTCAGAATGGTTGATAAACTTTCTAAATAAACATTGGTCAAACTCTTTGCAAAACTCTCTTTAATGAACAAATCAATTGTGTTCGACGAAATACTTGGGTACTTTTtttttcaattgtgTTCGACGAAATACTTGGGTACTTtttttttttttACTCTAATATTTTCTTTTTAATCTTTTTCACGGGATTACTAAAGCATRAAATTAATTTAATTTTCTAAATGAATATTTATCATTTTGTTTTAAGTTTTAACCAGGTATTGGAAACCAAAATTCATAACCAAACCGCAGAAAATTTGAAACACAAAAATCAAAGAAAATATGAGAAACTAAGCACAGaaataaagaaaactatgagaaattataactttcttctcaacattttttaagatTAAGTCTAACATACACTCAGTTTGAATCTTAGATGTCTCACCTCTTTTTATATTTTTATTTTATttttgattagattAAATATCGCAATTCTAATATTTCAACCACCYCTAAAAACCAAAACCCAAAATCATTAATCAGTAATCAAGGTGCAAGAAGAACCAATGACAAGAATTATGTGGCGTATGAGAAATAACAAATATGCTTGGGGTCKATTCACAATTTATATAACTTCCTTCCCAAA

>Cs1g11510

AAACAAATATAAATTAATAaaactatttaattatttaattaatttttataagatattttagatatattttattttatcaaaaagttagcaaataatgtatatataaaatcaattatttacttgtattattaattttcatgattatttttaatcttacattggtgattattaaattcaatacaatcannnnnnnnnnnnnnnnnnnnnnnnnnnnnnnnnnnnnnnnnnnnnnnnnnnnnnnnnnnnnnnnnnnnnnnnnnnnnnnnnnnnnnnnnnaagtaaATCATGAATAAAATAATTAATTAAATTTTAGGTTACAAATtaaaaatagtgttaTttATGAAAGAAATATGAATAAATTTTTTTCTCTTATTCTAATCCCATCTAAACCCACTTATAATTTGGGATTGTTAATCCCATGATTTGAGGACTAAATTTAAATTTAGTCCTCCCTAATTCAATCCAAACAAAGTAGCCAAACATGGGATAAAATGTTTAATCCCAAAATTAATCTAATCCCATACTTAATCCTAACTCCCAAATATGCACCAAGTTTGACAAGCTCTTAAAATAAACTTTTAGAGAAAGCAGTAGAAAGSAATATTATTAATTAATAGAAAGCAACCACAAAATGATGCATATTTGTTGAATGTATTTTTCCAAAAAAAAAAAAGGTCTATACATAGTATATATCCTCATGACAGTTATAAATTACACACTCTATGAGGAAAATCTATACGACTGGAGAAGTATTCTCACATAAATATGATCAATAACGTTTTTTTCTTTAAACGAGAAAGATTTATATAGGACAGTCATACCACATTAGCAGCATGCACATTTAGCACAATTGGAATAATGACAGTGTCTAGTTAACGTTTAATTACTTACGCCGCTCGTTAATAATCAAATAATAATAAGCAGCCATTTTTAGAATTTAAGAATTAACAGTTGCTTAACAGAATAAACGTGGCTTCTTTATCTCTCAAATTAATCAAACA

>Cs1g19090

CTACGTAATTGTATGATTCCTAACTTTACCTAGCATACAAACAAGATCAGTAGGAGAATAAATACTTGACCCAAATGGTGAAAATTTTCATYAGAAAGTTAAATAAGAAAAAAtataaattaatattGAAGTTCAACAATGGATGATAGGAATGATAAGAgtaccaaatgcaaaTCTGTGTTCATAATTGTCTCTTACTTGGATTCTAGTAATACCGACCAACTAATTTCATGTTTTTAAAAACCTTGACATGATCTATATTGTAGTACAAGTTTAAACTCATGGGATAGTATTAAAACCAAATAACGACGAGCGTTACTAGATAATATGTATCAAAGTCAACTAATGCTCAAGTGAGGCATACACACCCTTTTTCACATGATAAATTTTAGATAAAATTGTCGACCTACGCATGATGGAGGTTTTAAAAAAGTATTTTATAGTCTCTTCAAGTATTTAAGCTTGGCACAATCTTTAACTATATATGAGCAAACTTTTGAGTAAAAGTTGAGTACATAAATTTCTATATAATATTAGAACTAATTCATGAGCTAATTAAAATATAAACCATCCATAACCATGTAATTAGTGATAAATTAAGCTGCATGAATGATGAAACCACATTGGAGATTTTGTTAAGGCCGAGTAAATTAGATAACGAGGTGACGCATTCAAACTATACAGTTCATCCAACGTAAATTTTATTGTTAGTAAAATTACCAATAAAAGAAAATTTTTACACGAGCCATTGGATGAAAATAAGTTTAGAATGTCTACATACAATTACAGTGAGGATCCATTATTTTGTTAGTGTTGGTTTCCTTTTTAGAAAAtgatacaacgacggatatccaagcataattagtaagttaagcacttccaattattcttaacttatctataaagaacaaacaaaaccgaTTGGTGAATAAATAGTAACGCCCAATGGCCCAATTTTATCACAATAAAATTAAGTTCGCAAAAAAAATTAAAGTTCAACA

>Cs1g16800

atagtcagtaatgtccccgacaactacatttttatgcaatttaactataattaaataatctttttatgtttagccataaaatggagtcataacttaactagccgtgaatacgtttaccatgcatgtGcaatCACTCGAATTTAAATAATTagtaagaaaaaagggtgGATGAAGAAGCTTCTCGTCGCGTCTAAAACTMTTATAAGAAAATACTTRCCAGTTCATCTCTCACTGAAAAATTTATATTTCTATAATTCTTTCTTATATCATGCTATTAAAATAAGTTTGAGAATTtttttatattaaAAATCTACATCTAATAATAAGAAGAAAGATTGACCCGATACCTAttaacttataaatacTACAAAGGAGGAACAAATTATCATGGGCTCTCTTGCCCGGGGAATGGTCCAAGCGCCTATGACTTGGTCTTTGAtgcgtgaataaaaaccTCAGATAATGCACAAAATATGGKGTTCTGTCAACCATTTATTAATGTTTTTCCTTTTTCTTTTTTTATAGAAACCATCTAATACTGCCGTTTACACTATACTGATAGAGATCGGAAATATAATAACTATATTCCAATAATTGCATTGCATCAGAGGCTAAATTTCCATACTCAGTYCAAAAAAGGCATCATCACAGTACAAAATATGTACACTTTGGTCAATCATACCGTTATAAAAAATATCGAGAGTGTGAACAAATGAAAcaaccatgtagaattgtagATCCTTCACCTAATGGTTAATATATAGAAAGTRTGTCATTTTYTAATAWAGATTAATGGAAGATGATTAACATAATCRGACTYCATTAATCAACTTAGCGGTCAAGGCATCCTACCCTGAAGAATTTTATATATGAACACATCACTATCTTGAACCTTGGCAAAAAAAAATCCTTAATCAAACTCTAATTGGAGAATTACGCGTCCGTATACTATCAATTAACGCCCTTAAATTGAGATTAGATGCTGCTTYGAGAGGCCAATA

>Cs1g06850

CRAAAAGAaaatgaaaaaaaatgacagGAGCCTCAGTGTTGGGGGTGAATATGATTTACAGGTCGATTGCCTTCCGTTTCCCCTCATGGTTAAATGTGTACAGAGAGATATTGGGTGAGATTTGTAATATTAGTGAGTTCCATTTCWTCTTTCTTTGTGCTTGTGCTCATCGGAATGGAACACATGGATAGCCATTCTGATTGAGCAttgagaaaaaaaaaatagattCGTTAAAAAaaatgaaaaaaaagaagaaATCTACACCGAGAATTTAGAAATCAATGTATTTCSGGTATCGGGAATTGTAGAGTTATGAGCATGGCAAAAGYTTATTGGMTTTAAATGAAATTCTTTTGTGAttcatyttgccAATCTTGTTTGTTACGCTCATCAGCAGGCTGCTGGGAATGGAATTAGGTGTTGATATAAAAAAAATATGGTTACTGGCACAGCGGCACTTCAAGTTtaagcctctgtgtgtGCGAGCAAGAGTTTTTAGGATCAGCTGTAGCACTTAATGCctaaaccttgtgcGCAAGTATTGGCACCAAAATTATGTAGAATGGGTTGAAACTGAACCATACTATGTCRTTGTAACATTAGTAGTCATATTGTTTGGGACACCTTGTACAAAGTAATTTAAAAATTTTGCTTTGAGATCAGYTAGTTACAATCAGTGTTCCGTTTACAAGTTTAAATGTTCGGTGTTCCATTTACAAGTTCAAATGTTTGAATCTTGATTTCACCAATGCATCAatttgtccttgamGCCAAGTTATGCCAACGCAAGCTCAAAGCCTAaacgtggaatgaaATTTTAACCCATCACTACTATCCGTTTCAACTTATTCAGGCTCTCAGCCCATAACTATTAGGAGTGGAACCTTTCCGAGATTGGACCAAAATACCCTCATTGGCCCACCAAGAAGGTTAAATAAAATATGGCATACAATATCGGGAAATCCAAACCTTGAATTCTCATTGGTGGCTGAGAATTGATG

>Cs1g08205

ttagtatTAAAAATTGTATTCTtTTCTtcaaactttcgttattatttaattttaaaaaaaaatatcttaaaaaaataagaaaaaccttaaaaaataataaatattttatgggtttaatatgattttatcgtgtctataaactttgtgtgcaacaattagttgagaatttttaaaaaattacttgtagtgagatgaattttttTTAAAtTTATTTTCTTACTATGTTGTATCCTAATTTTGTTACTATGTATATTACAAGAATATTAttttgaCCTAGAGGATTACTTCTTAAATTTGGATTATTTTAATGataataaaaaaaattatattgtgcaagtaaaataaaattgaagagagaaagaaaatataaatggggttaatcctatttatatttgctcttaaatgtaannnnnnnnnnnnnnnnnnnnnnnnnnnnnnnnnnnnnnnnnctaatagtccagatcttttttttttttttttatatgtttgttatacttaatatcctcattaaatttcttgtctttttggacccattgatagtttgagattttttttttGAaattcttccacttttattccttcttttccttttaattaacattattttgtttttattttttaaatattagggagatactaaccggaccatagtacagttgaaaacattaattaAAAAAAatgatgttactccATTAAAAAAATTAATGTATTTATTTTGACATTTTAAATTTTAGTTTATTAKTTTTTAGATGTCAAAATTTTGAATAAATATGAATTTGAAAAAATAAAAAGAGATTTTAACAGCATAGAAATTTATGGAAAATATCTGTTAtcacaattttcaataAATTTGAYAGAAGTTTCAAAATTGTTCAAATTCGTCCAGACTTGAGGCTCCTATAGCGCAATCCACGTGCGGAGGAGATGCCACGTGTGGATCGGACCGTTCAAAATGTAGTGAATATTAAGCGCGGCAAATCGCTTTTATGTAGCGGACGTTG

>Cs1g23040

ATTAGATagaatatttatttaATATAATTTTTAAGAAAAATATTTACTTAATTTTCAACCACAAGCCAATAACCTATTGGTTTAAtsagcacctccttTCTTTAACTCTAAAATAGAAGGTTTGATTCTCCTTAATGATTAAGCTTCAATAGGCTTATGTTATGGTGCTTCGGCTGAATTATATACAAGATTGAAAAAAAATAATAATCACAAACTTTGGTTAAAAAAATAAATACATTTGCTGAAGACGTGACTTTTGTTAAATAAAACATGCAATKTGGCAAGAAATTTTCTTTTTTAGataattaaaacatctTAacagagatttttACAGTAAAAAAATAAAAAGTAAAAATTCAACCACAAACTAACGTCACATATCAGACCACAATTACGATCAGTTAAATGAgttaacaaaatgagatTGCCGACAACCAAATTTTTGGTAACCATTCAAGTTGAAGACGAGGACAGAAGCCACCATTAAAAATTAAAAATTGTACGAAAAATTCCAAACTCACCGKCCCTTTGCTCTTCCACCATCCAAGTTCATCAAGTTGACCCAATTATGCGACGCCTCGAAAAGtgagaaagagagagagagagagagagagtacggaaAATTGGCGTATTTGCAGTGAATCTTATAACCMTCCCTTTCAAGCATGAATACATGTACACATACATACATACATAAACATAAACTAGTTTTTATATGCGTCATCTGTCGCTCTTATTTATCTTGGATGTtCAttaattaattcagacacaacaatagaaagaaaaaaaaaaaatgggactgaacttggatttgaacatggcttacgttccaaaaacaaccaaggtgtcaaagctccatgagtgtattaaaagattggaggaagagaggagnnnnnnnnnnnnnnnnnnnnnnnnnnnnnnacgtaaactagtttttatgtgcgtcaTCTGTCGCTCTTATTTATCTTGGATGTTCATTAATTAATTCAGACACAACAATA

>Cs1g03840

ACTGGAGAGTGTATGTTTATAAATGAAAAAAATCATCATTTTACATTTCAAGTCTCACTAACATCTCTACTTTTTATGTAATTAAGTCCTTTAAACTTTGATCGTGCGTGTTCTGTTATAATTTGAGTATTTTATATAttttgggatatTTTGATCATTTCAAGAGTAACAAGAAATCGAACACCGACACCGACATCATGAATGGATTTAGAAGCCCTAAAAACCAAAGGAGCGACATAAACCAAGGTTAAAATGGATTCTAATTATATAATGTATCGAACCGATGCACACTCCTAGCTTTATTTGCAATTTGGAGAGACATGTGTAAAAGAAATATTAGAAAAGCGCATTGAATACTAAGGTTTAAGGGATGGACAAAGAAAAAAGAGATTTGAATAGGTGTAGAGAATAAATTATTTTATTTAATTACATAATTTTATTCTTAATAGTAAATTCATTCGTGGACATAACAGTAATAAATATTTTTATAAAAGTTTGGGTTTGTAAAGTATTCCGGATAGTTCAAATATCCTTACCCCATCCTAAGGTGCCGCTTCACTTGCTTTTATATCCACGTGATTTTTTAACTTAAGTTATATGGCAATTTTGTTTTAACCCCAACTTATTTTCTTTAATTACTTAAACATCCAAACTATTGGAAGCTTTGGGCTTGGGCCACCTAAATGAAGTGAATCCAACACTTTTCATGAGTAAAATAGGAAAATAAAATATAAATGGAATAAAAATTTTAATTTTTTAAACAAAACCATATTTTTCATCAAATAATGTAGCTTGTTTATGGTATGATAAACGCTTTTTGTTTGATACGAATCTATCTGAGTTAAAATTTCAATGTCAAACGATGCCATTCTTATCAATAATGCagtacttttattatgCCGCACACCACACATTTAAAATTAATTTATCAGAGTAGTTGGTATGATTGTATACTGATACATAAGGGTTTAAtctcttttgcaatttgcacatcagCAAAA

>Cs1g04990

TTTACTttgtcactattattattaatattaTTAACTTTTCTGGCATTGATCTTAAAAATGGGCGCCTTCTTCGGGCWGCCATTTGCTTTGTCTACATTCCTGTKGTGCCCATTAAGGAGTGGGGTTCCTTAATCGATTTTAATTAGTTGAAAGAAAATATATCAGAAGAAAGGACTCGCCTGTGTTGCAACCgttgcaacataccactACTTTTGTCATTTTGTAGTTTGATTACACCACACCACCTTATATTCTCTGTAAAATTAAATAGGTACTAAAAGTTACAAATTTTTACATATAAATCCTTTTTTRATAAAATAGCATTTGAAAAGAATTTTAATGATTGTTAttatttatcattaatatcattattattatctaatattattgctatnnnnnnnnnnnnnnnnnnnnnnnnnnnnnnnnnnnnnnnnnnnnnnnnnnnnnnnnnnnnnnnnnnnnnnnnnnnnnnnnnnnnnnnnnnnnnttattataattaattaatttTTTGTCTTTaTTGTTATTATTATTTTAATTATAATATATACAAATAATATTCGGGACATAATTGACAAAGGACATTTTCGTCTATTCTGATTCTAAAATAAAGGGAATGTTATTCATTGTGATTTCAATTCCTACAGTTCAAGTAAAATAATTTATTCTGATTCAAATTCCTTATTACGATTCCAGTTCATTTCTATTTATGTTTAGTAAATGCACCATTAAARTGTGTTTGGAACATAGTATTGTCTAGTATTATACTATTagagtcagcttttannnnnnnnnnnnnnnnnnnnnnnnnnngagaggaaagaggggtttctttgtttacttataattggaaacattgtttatttagaaaagagtattttcggtattataattgaaaaaggggagcccctatgttacannnnnnnnnnnnnnnnnnnnnnnnnnnnnnnnnnnnnnnnnnnnnnnnnnnnnnnnnnnnnnnnnnnnnnnnnnnnnnnnnnnnn

>Cs1g16030

CCTCTTTAAATTGGGACCTTTTATTTCCACTTTAAAGATRTTACGAATTAAATATCAAATGGGTATATAGGCGCAACCTCAAGTCGAAGRATAATTTGCACGTTTTGTAAATACAAAGTCAGCGTAGAGAGCGAATTTAGTTTTGTTTCTTAAAAATAAATATAACAMTATAAATTTATAGATatttaraaaaaaaaatcaatTGATTCTTTTGGATCAATTTTGTGATGCCGAATCACAATTGTAAAAAAATAAAATGAATGAAAAGCCCCGTCAATCCAAGAGTCAAAGCTATAAGTTTTTTCACCCTgtctgtggggaaaaaaaAAATCAGTTGAATCTTTTGGATCAATTTTGTGATAGCACTATTCAAATATTCAAGCTTAAATTAAGTCAACTTTACTTTCAAATACTAGATAATAAAAGTAACTCGAACCCCCTTCTAGCATTTGGGTTCAAGTTCCATCATAAAAAAAAAAATTATAAAAACCACATGAATATGACTCAGAAGAAAATAATATAATAATAATAATaTTACAGTTACAAATAATTGTAAGTTAGGAGATGAAATGATTAGTTGAAAAGGAAAAGAAGTTAACAAAATTATCATTTGGACCTATGAATTTTTTTTTTATTATGCTAATATCTTCGTGATATTTAAATCGAAGAATCTGATACGATGGCCGCATAATATATGGCATTATAGTATTAAGTAAAAAATTATTACTTTTatagttacttttannnnnnntatgtcaatatttagactgttattttcctcttttcgtatatattacgttttattgtcaactcaatccgtacaatttttgtgatactatcataattgaaaataaaatttctaaaagtagctttttttttttttttttCAGAGTCGTGTAGTTGCGACTTGCATGGCATTCGTCRTCCTTAAATATCCTTGCTTTTTATTCTATTTTGTGTGATCAAATAATACAAGTAAAGATAAATATggaagtatcaat

>Cs1g21210

ttttTTTTTGCTTCAAACGGTAGGGTTCCATTTGGTTTGTCAAGATCGAATTAACATAAATGGATGACTTAAGCCGTATGGCTTTAGCCAATTTAATTTACAAATATACATTCTGGTGCAAACTCTTGTGCGGCAAAATCACATCTTTTAATTTATTTCCAACTTGAGATAAACGTTTGACTTCAAAATTCAAATCGAAAGCTTGCTCGCCAGCTTGCTTTCTAGGGTCAATCAACACAAACAAACATTAAGTGAAAAAAAATAAATAAAAACAATTGCAAGAGAGAGAAAATGGCCGACATGGGCTAGATGGGCCACGTAGCACGGGGAGCATCTGGACCAGGGTGAATAGTCGAGGGTCCAAATTAACATGGAATTTGTGGCCTTGTGGGTCACGCTAGCCACTTTTGATGAGATTTGCTGTCGGTTTGGCTTGGTAAACACGACTACAATATCTTTTtctgccaaaacattaAAAAaatttaaaaaagaagtTAATTTTTCTCCGTTTTTCACTTACCACAACACTCGAAAACGTTATGGCACATGAGTGATGCACAATTACTGTAATCATTCCACGCGACTTTGAAGCTAGTGAAGTGTACTTATTAATTGGATAAAAACTTGGAGTATAGAAGTGTGTGATTGTTTGGAGGGACGGTGGATAAAGCGCGTATAATTATCAATTTccctaataatgtAAATTTTTAGCAAGGTAAAGAAATCCTGTCTGGATGGTCAAAAGTTTCTTTGTCAAGCTGTCGTGGCATTCTTTACTTACAAAACAGCTATATATATGCCCACATTGTAATGGACTTCTCCAATACAATCCTCCAAATCAAGAGTCTTCAAACTTCAAATTAAATTGAGACTTCATAATCTTTTCTCTCTTTCAATTCCCTCTCAAAAGTTCTTCCGGCACGTTCTCGGTCTCTGTCTTTTGCTTTGGCACTAGATTCTGTATTGTACATCTTGAAACACTTGTGGACACACTGATTAAA

>Cs1g25340

nnnnnnnnnnnnnnnnnnnnnnnnnnnnnnnnnnnnnnnnnnnnnnnnnnnnnnnnnnnnnnnnnnnnnnnnnnnnnnnnnnnnnnnnnnnnnnnnnnnnnnnnnnnnnnnnnnnnnnnnnnnnnnnnnnnnnnnnnnnnnnnnnnnnnnnnnnnnnnnnnnnnnnnnnnnnnnnnnnnnnnnnnnnnnnnnnnnnnnnnnnnnnnnnnnnnnnnnnnnnnnnnnnnnnnnnnnnnnnnnnnnnnnnnnnnnnnnnnnnnnnnnnnnnnnnnnnnnnnnnnnnnnnnnnnnnnnnnnnnnnnnnnnnnnnnnnnnnnnnnnnnnnnnnnnnnnnnnnnnnnnnnnnnnnnnnnnnnnnnnnnnnnnnnnnnnnnnnnnnnnnnnnnnnnnnnnnnnnnnnnnnnnnnnnnnnnnnnnnnnnnnnnnnnnnnnnnnnnnnnnnnnnnnnnnnnnnnnnnnnnnnnnnnnnnnnnnnnnnnnnnnnnnnnnnnnnnnnnnnnnnnnnnnnnnnnnnnnnnnnnnnnnnnnnnnnnnnnnnnnnnnnnnnnnnnnnnnnnnnnnnnnnnnnnnnnnnnnnnnnnnnnnnnnnnnnnnnnnnnnnnnnnnnnnnnnnnnnnnnnnnnnnnnnnnnnnnnnnnnnnnnnnnnnnnnnnnnnnnnnnnnnnnnnnnnnnnnnnnnnnnnnnnnnnnnnnnnnnnnnnnnnnnnnnnnnnnnnnnnnnnnnnnnnnnnnnnnnnnnnnnnnnnnnnnnnnnnnnnnnnnnnnnnnnnnnnnnnnnnnnnnnnnnnnnnnnnnnnnnnnnnnnnnnnnnnnnnnnnnnnnnnnnnnnnnnnnnnnnnnnnnnnnnnnnnnnnnnnnnnnnnnnnnnnnnnnnnnnnnnnnnnnnnnnnnnnnnnnnnnnnnnnnnnnnnnnnnnnnnnnnnnnnnnnnnnnnnnnnnnnnnnnnnnnnnnnnnnnnnnnnnnnnnnnnnnnnnnnnnnnnnnnnnnnnnnnnnnnnnnn

>Cs1g18880

ATAAATCACCTACAgtaacgaaaaatatagTGTCACTAATTTTATTTTTAAACATCTAACTTGACCATTTTAAATTACATTTATGATGTGTTTAATCTAAATTTAAGACAAATAGTTTACTCAAAAAAACATACAATTTTTCGTGATGATAATAATTTTGACCGTTAGAACCCAATATGACACATTTCCTATGATTAACGTATGACGATTTATTTTGTGGCATGAGAAATATAATGCGAGCAATTTTTTGTCTTCAATTATTAAAATTTTGGTGTAAAatatcattttttataatATATAATTATATTGGGACGACCCAGCTCACTTGAAAGTGGGAGGACTATCATGGGGCTCCGATCAGAGGCTCATCGACTTTGTTCATTCTTTTTAGGGACCGCTSTGTCAAGGAACATTCATTGAGGACCGTTCGTCGTATCCATTCAAGTAAGGGCGAAAGTCATTTGGAGAGACAAATTATTGAGTGTTTATCTGAACGACTTTTTTGGTATAAGCAGGAAGTGGGTGTTGGTGTTTACTATTTTGATTTAatgggtcatctccatctccatccaaatGTTAGaaggagaaaaaaacaaatAATAAACACTACTAGACTTGTTGGCAATCTAACTATCAGAATGCTGACTTTAAAACGTTAAATTGAATGCTTGTATTGATGACTAAAATAGGCAGCCAAACCCCAATGAGTGGAATTGTAGAAATGAACCTCACCATACATGATGATTTGGAAAAATACTCTATCAGCTAAAGAATATAATTAAAGTTATCTTACTTACTCGGAATATATACCACTCTAATGTACACAATTATAGAGCTtttaacccaagtacttcaagcnnnnnnnnnnnnnnnnnnnnnnnnnngggtcaaatccctcctttgtttggagaagtatattatgaggaagacaagtgttgcacaaaggcttaagatggagaataggtaatggaaaccgggtcttagtgtccagcaggaattgg

>Cs1g09830

tgaaatttttctgtcatttagttcagtttagattgaaaagaaccgaaaatttttgaaatttttttctccttttcttttttttttttttaaattctaaaatagaatagaattgatttggactaacctaaaatttttgttgatgatggcagtggatcaaaatgttggatttattatttcgtattgaacttttgtctctcatttattttgagtttagatcgaaaagaactaaaagaaaaaaattgtctcaattttttttaatcaaatagatttgaatcatactatattaaaattttcaaTTCTTTGTTATCATTCAATTTTATTAGATTTTCTTAgggTATAGGCATTCGGTTTGGTTTTCCCAAAACTAAATCGAAATATGTTGGTTGGTTCAATTCGGGCAATAAGAATTGAAttgttcaaaaaaatatgaACCAATTGATTTTTTGGTTTAAAACGAATGGAACCAAAAAATTAATCAGATCAATATCAGATTAAAAAATAATTTATTTTTATATTAATATTTACATGAAACATAAATAATTAGTGATTACAATTGAATTAGAAAAAAAAATGATTTAAGTAAAAAGTCTAAAAATTTTTCCAACATAAAAAAAATGTTGAATTTCTTGAACCGAATCATATAAACCGTACCAAATCCAACTAAACTTAAGGAACCAAACCAAATMATTTAGGTTKAATTCCTAAGTTCGGTTCGCATTCGATCATAAAATTTTCAAACTGATTAAGTTAATGCCAACCcctaaaktttttctttttctttttctttttaaaatAGAGAAAGAGACACTCGTATTGAAATAGATGATGTCAACACGGAAAAAGAATTATACTGTCAAAACAACTTCTTTTCTAAAACATTATTAATTCGTAACGTatcaagtatcaacgTCACTCTTCAAACAAAATAAGTACTAATAATAACTTAATGAAATTTGACCAAACAAAAGGCCRCTYCATGAAATTTGCCCATTAATACGAGT

>Cs1g04235

atgcataTATGGATTTTTAGACTCAAAATTGTGAAAAGTATGTAGAAGTTGAATGACACCATGTTTGAAATTAAAACATGATGGATTaggagaaaaaaactatgcaTGATGTTGCTTCTGTTCTAGTAGGATTCATGAGATCTCTAAGTGTCCTAGGTTTATCATGAATATTTTGTAGTGCTTGGTTATCCCCTTGCACTGAAAAGTTATCAACTTCGTTGTCTGTCATGTCTAAGGGTAGTGAAGGTGATCTTCTAATTAATCTGTCACTATTAGTATGAGACCAACATCTCATACAATGCTAATGATGTGTGAGTGTGTGTCAAATTAAGTGAAGAGAATGATGTACAATCAAATGAGTAATGCATAAAATATGAAATAAAAATTGCACAAAATAAAAaaatnnnnnnnnnnnnnnnnnnnnnnnnnnnnnnnnnnnnnnnnnnnnnnnnnnnnnnnnnnnnnnnnnnnnnnnnnnnnnnnnnnnnnnnnnnnnnnntattgcaaaactaagaatttaaattaattaactaaacataaaataataacacatccacaaaataacaatgtaggcggtattataaaatcagnnnnnnnnnnnnnnnnattttttaaaaaaagaaattataacgagaaatagaaggatacacttacctcaagatgtaaaattgacttttaaaaattatcaaccaattaagcaatcaatttaaatttaatccttaattatatgtttcaaagtggaaaatccaagtccatttagtccacaatctctaaaaagttcaagttcatttagcccacaatgtaatatacgcacttggactttcttaataaagtgccgatttttattttcctcttctctattaatttttaatttcgtcactatagtttaattgggataaattgagctcgattacacgcgtcgatggccggattttatttttatcacgggtccgaattgnnnnnnnnnnnnnnnnnnnnnnnnnnnnnnnnnnnnnnnnnnn

>Cs1g06350

ATAAGTTCATGTTTACATGAAATGGGTTCTTGTTTTAGTTCATTTTTTGTCTGGATATATGGGAATTTTTATTTtctttcctgttatatagtttggcgacaaataaattagctttgttctgtgcatttgaacttttttcgatctGCATCAAGCCTTATTGAACTGCCGTTCTGTAGTGCCCAATGTCTGTTCCAAGCCAAGTTGATTGWTTATACARCATTAGACCAAttacytcaatgctgAAAATTATTCGTGCCTTATTGAGAATATGTTTTAATAACCGAAAAACGAGAACTNGCTGAAAATTTCTTCATCCGTAATGTTTATGGAACTTTCAGATTCTTTTAATCATCcCAcgAAGAGTCgaactTTCAGATTCTTTTAATCATCCTTCCAAGAGTAGAGCTTCAAGTCACATGCAATATATGTTATCAtcagattttttGGTCCCACGAGTTTGTTTTGGTTGTTGTAGCTGAAGAGCTCTGGTAGGTGTAGGTGCACTGATGATCTATGTGCTatttatttatttttgattaatttaagtgtaaaCAAGATGAAAATTTTCTAGGCAGCAAGCAATTGATTCGCCTGGTTAAATCACAGTGTACTTTTTTTtttttttttccaaatTTCTTAAAATAAAAAATTACTKTTAGCTTAAATGCTGTTAAGTTTCTCCCAATCACAGACATAKTAGATATAAATTCTTAGTTTTTCAAGTTAAAATTATCCCTTTTGCAAGTTAAATATTTTTACTTYGAATTGCTTTGTCCAATTAGTTtactgttttgtctcaTGGGAACTTTGTCTYAATTTTATAGTTCATCAGCCATCTTTTAGTCTTCAYTTTGAACATAAGAACTATGTCTGTGTCKARTAGTACACTGTCAATTGCTAATATAGTCTTTGAATATGCAGAGAGAKAWAGCTAACAGAGAGGGATATCAACTGCTTACAAACATYTTCAATTAGCTATTAGCTACTCAGTACTGACACTA

>Cs1g23580

TTTCAAACTCACAGCAGCCGCCATAGCATAGGTTACCAAACACCAAGCTACTTTTTTAATCAGCAGCTTATTTCATCTGCACAACACAACCAGCTTATTTCATCAACCTRTGCAATCTCAAACTGYCCCTAAKTATTYAAYAGAYAGAAGCACTYTCTCATTTTAAAAATCTTATTTAGAGAGTTTTTTAAGAACTCTTTTTATTAatataatttttaaataATTAYTAAAATCTCATTTYAAGAGTTTTTAATAATAGKTTCGGGTGTAAATTTATATTTAGCTTTGTATAATACTATAATATGACTCGACTAATTTCTCGAATAATATGATATTTGGGAATCTGTTTCAACGTAACTACGGAACGTCACTGCATGCATCATGGTCCaaagtcaaacttttTGGCTGCAGTTGAGTTTTGAGGACATTTTCCACCTATATGGTAGAGGTAGACAGCAATAGCAGCGGTACCAATTATGAAAGATATATATGGGCAATTTACCTATTTGGGTTGGAAATTGTCTTATTGTCATCAATATCCAATTTATATATGTATATACAATTTACCAGTGTAGAAGCACTTGCTGTACTGTTTTTtttttTTTTTTTTACATGCATTTCAAATTCCGTATCTAATTATTTATTTATCGATTGAATGTTAAGGCTGTATCTGCACGTCCGATTTCTCTATTTCGTTTATTTTCGGAATATTCTTTCATAACTATATATAAAGAGaaaaataaaaaaatcattTTCTGAATATGGACTtaatcaaacacatagataacacatagatttatgTGCTCGTTCAAACATGTTATATCATGATGGGAGAGGGTCAACGGTGCTGTCCCCAACTCCCCGTCATCCTACTCTGTGAGAATGATTCTTCTTTGAAATAGAATTCCATGACCATGACTGGCCGCTAAATGATGCCCTGGATATTTCCTTTTAAGCAACGTTTTGGCGTACCCCCGTTTGATCTTTCTTCTAATTTCATC

>Cs1g07690

gacaaagccttctcatattgcccaccccagaggagagaaacaacagaagtggttccctccccctgaaaatattttcaaaattaatgtggatgttgccatcaatactaagaatcagattgcaggcgtgggagcagtgattagagattctaatggaaagataattgcagccggtattaatcaaattcatcttaagggaccagtcagtttagctgaggcagagactgtgcaatgggggcttcagttggcaaaagaagcagatttaacctccttgatcattaagtcagattgcttagaggtggttcaacttgtaaacaacaccaagagcagcaaaacagaaattttttggacaattctggagatccgaaatcagttgaaagtttttcaaaaagttgttcatcacataacaagtcaatgtaatgcttatgcccactctctagctaagttagctttgggaagaaATTCTTCTTCTATGTGGCTAGGAACTATTCCAACCGAGATTCAAATTGTATTTGAGGTGTTGTGATTTATGAAAGATTTACTTTCTTTTCaaaaaaataaaagtATTGGATATGCCTATTTATCCTCTAATAATTACTTTAAATACTTTATTGTTCTACACACTCATCTATTAAATTTAAAAAATTAAGTTATACTTTGTATGGTCTTTAAAATACTACAAATCCTTAAGCATGTTAAATTAATTACATTTATTTACTCAAAACAATACTCAAAGAAAATTCTTTAAAACAATTAAGCATATAATACTGAAGTTTTTATTGAKAAATCGACAAATCCTAATATAATTTATTTCTATCCCCTACKTCTTCSAAATTTAAACTTGGAGTGATTAAGAGAATAATAATAATAATAAATTGTTTAGAAAACCACTAACCAAGGCSTATATCATCATATCATATCATATSAATTTTGTTACAAATTAGTTATTAATATTTTGATAATTTAGTGACCCTCATTCKACCGAAATTGGATAYCTTTTTGTA

>Cs1g18000

TAATWTTCTTAACATGGTACTAAATTAGGTTTTGTGTATGTTTTTTCACTTACAYAATCGTTTCAACTTTTAACTTACTGTTTTGATCTRSGGGATSTAATAGACTTTAGAATTAGATTTGTTTCGCATTATAAGAGGGATAGTGCTATATatttcatttttttttatcccAAATAATTAAGGCATTAATTAATTAATTTATTTCTTGGGATAATACAATTAATTCATTAGATTTTTGTAAAGAGTTATCAACTAATTAATGAATARCACATCATTTGAGATAAAAAATTTAAGATGCCTATCATTACTCTTATAAGGGAGAGTTGTTTTTGTTGGCACTCTGYTTTGATCTCTCTAGARCCACACGAGTTATTCGAAATCAAATYTTCTATTATTTATATAAAAggagagaatatttACCTATTAACTTAAGTTTTGAGATTAAAGATTTCtttaataaaatttgaTATCAGTATATGCACGCTCTGTAAGAAAAAAGATAATTTTAAATGAATTWTACTTGGGACATAGAAAAACTGTTTCATAAATGATTATTGCAACACAATAGTATATAAAGAACCAAGAGGGAAATAGGGATTTAGATTTTAGAATCCAAGTAAAAAccctttcaatggaaaagaaaaAGAAAGAATCCAAGTAAAATCTGTAAAGAAMGACAGTAACTACTTCTGTTGTATGAGTACACTTGTCGAAATCTCAAGCATCCYAATATAATCCTATTTTTGGCAAAAGtgaaaaagagaCAGAACATGAGCGGGTAAATATGAGTTAAGGTTTGAAGAACTTGCCACGTACTTATTGTTATTTTTATTTTTTCAATCACATTGGTTCTCATTTCCTCCTAATAACGAAAATATTTAATTGAGTTTCTGATATTCCAGGTAATTAATTAATTTAGTGGAAAAAAAATCCAATGAGAAAAGTGAGTGTAAAAATAAAATAAATAATAAATAAATGAAGAGAAGGACCCCCGGAAATTC

>Cs1g13460

ctaaaaataataattnnnnnnnnnnnnnnnnnnnnnnnnnnnnnnnnnnnnnnnnnnnnnnnnnnnnnnnnnnnnnnnnnnnnnnnnnnnnnnnnnnnnnnnnnnnnnnnnnnnnnnnnnnnnnnnnnnnnnnnnnnnnnnnnnnnnnnnnnnnnnnnnnnnnnnnnnnnnnnnnnnnnnnnnnnnnnnnnnnnnnnnnnnnnnnnnnnnnnnnnnnnnnnnnnnnnnnnnnnnnnnnnnnnnnnnnnnnnnnnnnnnnnnnnnnnnnnnnnnnnnnnnnnnnnnnnnnnnnnnnnnnnnnnnnnnnnnnnnnnnnnnnnnnnnnnnnnnnnnatcaattgtaaaaccaagttaattaactaacattgtagtgctcatacattctcatcacatttcttcatttcgtaaatagttgaatattgaattctatacaaactatgggaaacgtttggtagccatttgaannnnnnnnnnnnnnnnnnnnnnnnnnnnnnnnnnnnnnnnnnnnnnnnnnnnnnnnnnncaattgtaaaaccaagttaattaactaacattgtagtgctcatacattctcatcacatttcttcatttcgtaaatagttgaatattgaattctatacaaactatgggaaacgtttggtagccnnnnnnnnnnnnnnnnnnnnnnnnnnnnnnnnnnnnnnnnnnnnnnnnnnnnnnnnnnnnnnnnnnnnnnnnnnnnnnnnnnnnnnnnnnnnnnnnnnnnnnnnnnnnnnnnnnnnnnnnnnnnnnnnnnnnnnnnnnnnnnnnnnnnnnnnnnnnnnnnnnnnnnnnnnnnnnnnnnnnnnnnnnnnnnnnnnnnnnnnnnnnnnnnnnnnnnnnnnnnnnnnnnnnnnnnnnnnnnnnnnnnnnnnnnncatcaattgtaaaaccaagttaattaactaacattgtagtgctcatacattctcatcacatttcttcatctgtttaatagctgaatattgaattctatacaaact

>Cs1g08380

attattttaaataaataattgaataatttTttatgtttaatattacatactagtttagataAATTATGGAATTAAAATTTTGATATTCATTcttaattatatataagttAAAATAAATTATAAAATTAAAATTTTCATATTTAATTTTGAAAGAAATACAAATTAAAATAAATTATTGAATTAAATTTTAATTTTTACTTTAAGATTAAATACAAGTGAAAGTAATTATTGAACAAAATTTTAATATTTGTAGCAGATTTATATTATAATAAATTATTTAATCCTACTTAAATTCYTGTAGAAATGAGCACTATTAGGYCATCTCCAAAAGTTTTTATTAATTTTACTTTTTAAATATTTATTTGCTTATTTATGTCGTAAAAAGGAGAGTAAAAAAATATGTTATtCtCCaaaatactctttaaataaaaatatattaatattattttaattaaataaatattttttaaaagaaaaataaatagtaattaaaacacttctttctttctccaataaaaagtaataaaatataaattaaaaagggagaaaataactctataaaattgaagaaagataattatttcttatttgaaaaattttaattgaagtattttttggatnnnnnnnnnnnnnnnnnnnnnnnnnnnnnnnnnnnnnnnnnnnnnnnnnnnnnnnnnnnnnnnnnnnnnnnnnnnnnnnnnnnnnnnnnnnnnnnnnnnnnnnnnnnnnnnnnnnnnnnnnnnnnnnnnnnnnnnnnnnnnnnnnnnnnnnnnnnnnnnnnnnnnnnnnnnnnnnnnnnnnnnnnnnnnnnnnnnnnnnnnnnnnnnnnnnnnnnnnnnnnatcgttacagaGACTGACGTCTCATGTCTGTATCCCATAATTTACGCAATGCTGACTTCAACACTTCATCTACTCGTTTTCTTTAGTTTCTATAAATAGCACAgtacagtgaagccaaCAAGAGCTGTACTGCACTTCACTCACTAAAACTCTGTAAAAG

>Cs1g22230

ATAATTACCGATCTAGTTCAATATAGGCAACTAATCTCCTGGATCCGTAAAGCATCTTTTGCAATCTCCCTCAACCTCCTCGCCTCTTCCTCATCCTCCACAGGGTCGCCGTAGTACCGCTTCCCTGCAACCATTCTCAACATAATATTAGAAGTAAACTCCAAAAACAATGTTTTCAGCTMCATCTTCGGAAACTCTTGGCGTGAATAAGTCGACAGTTTTTTCAATAACCGATTGATTTCATCTCTTCTAATTGATATAAACACGTTAAGACGATTTGATGAGAATACCCTCSATAAAAAGACTATTATTTTAATATCCATYATTAATCATCTTAACAGTAGGCAGAATAATTTAATTATACTTACTATTAGTATTTTAAATTCCAGGTAAATCAGTCCTTTTATTTATTAATATTTTAATTAGTCTTTTACAGGTCAACGTATGCAATGAGcctagaaattttTTTTTAAAAAAAAGCAAAGGTGTAATTAATTTTTTAAAGGTCAACTAATAGTCTTTAACGAAAAAATAATTAAATCTAAGAATTTGTAATTATTTAGTAGTAAAATGGTAAGGTACTGTAGTTGCAATTAAATGTTATTCCGCAATAATTGTAGTAGTAAAAAGAAAATAACTTTAATAATTTATCATATGTAGAAATGTTATTATTTTATTGATTTGTGTCCCAATTATGTTCCTGCTTTGGTACAGCTATAATATATCATTTTTGAGTTTTTATTTTACAGTATTAAATTYTTTGTGGTCATTGCAAGTGTTTAGAAATARGAAAATGATAACTGAACTAAARGACGAYAAAATGAAGACAGAAACGGCAGCATACTGCTATCGTTTAGTGATTTATAATTTGCATCTCCTTGAGCCCACGGAGAAGAYAAGAGAGMGAGGAACCGTTTCTGTGCATTATTTTGTCGTACAGCGACTGCCACGTGCATTATTTTTTATTGTATATTTTCCTACGGATAAGTGTTTGACGAGAG

>Cs1g23990

nnnnnnnnnnnnnnnnnnnnnnnnnnnnnnnnnnaaaaaataaaaaattacaagtgtaaaataaaaactcatTATAATAATGGTTACCTTATAAATTTcctgatttttttctaaatTAAATAATAATAGTTTTAGGTTTACATAATACATGTGATAATATATGTCAAattttattaaaaaaaattaatctcnnnnnnnnnnnnnnnnnnnnnnnnnnnnnnnnnnnnnnnnnnnnnnnnnnnnnnnngattttgtatccttctaaacttcttcgacaacaatctcaaatgatatttaagtataagtaAAAAGTAAAGTTTCAAATTACATAAAGTACGTAAAAAAGCAAAAAATAAATATAAAATCCTATATGTCTAGTAATATAAGAATTTCTttttctttttctttttttggtcttgatCYACATCAAGAAAAATAATTTATTTGATTAACATGTGTAGTAAATAAACACTATGATAGAAAATGaAAAAAAAAAAGTTAtaaatatcctataatgaacatagaattTATGGTTAAACCAATATAATAAAGTTATTCTAATTGCTTCAATACAATTTTTtgttttcaattgatgagttttctttgttttattaaattaaatgnnnnnnnnnnnnnnnnnnnnnnnnnnnnnnnnnnnnnnnnnnnnnnnnnnnnnnnnnnnnnnnnnnnnnnnnnnnnnnnnnnnnnnnnnnnnnnnnnnnnnnnnnnnnnnnnntaatctatttattttaccttatatttactagACTTATATATAATTTATAAAATTTATATTTATCACAATATTAATACCATATTTTTAATATAGGCATCGTAGAATTTTCCTACATGGTTATAGAGTTCTTTTTTCCTATTTCTCAAGGTTAACCAAAAAGAGAAAAACAAATATAAAAAAGAAACAGTAGAAAAAAGTAAACAAAGATTTCGAGCCCCCTTTAATTTGAAAATTAGCGATACATATCATAGATAGAGAAAGAGAA

>Cs1g06740

GCCTGAtgggcattccaATTTCAGACAATGAATCATTGACGCGATTTTTTTAATTATTTTTTTTATTTTCTTTCATACTTTCAACCATAAATAAATAAATGGTCTAAATTATATATTTGGGTAAATTTTACATTAATAATTTAATTCATTAGACATAATTGYAASTATATCTAGCGAGtaacaaataTTTACTATTTTTAATTATTTTAAGAATTTCAAATTTATATTTAAATAAATTAATCTCATTTTTTTAATCATATTAGTTCTTTAGAATAAAACCAATTGAGCTACCAATAATTCGGTTGATAATAAGCATAATGTGTGCTTAAATATTTTGTTATATAATATGAAATGTATTCGCATATTTGCACCAATGATAGTTTTTCTTCTTACACACAAAATTATTGTCTTTTTAAAAGTAGTTCTTAAACATGCAATTAAGTTATAATTGATTAAGTTGTTTACCTTTTCGTCTGTATTAAAAATTAAATAAAAAATGAATATAAACTTTTTAAATCTTTTACAGTCATTCAAGRATATATATGATTATTAATATAAACAGTATTATTAGTAGGAAAATTTGAAATATAAAAATCACAATTTGTAATATTCCGAATAAATTTCTCTCACTTCTTTTCTTTTAATTATATAAAATTAAATGTAATATTAAAAAATTTGTATAAAATCAAATAAATAAAACTTRAGAAAGTTACTGAACCGGCGTCGTGTCCCCCGGCACGTGGTTTAATGAAAGAATTTTGTTTTATGAAGCAAGYCGTTGGCTTTCCTTATGTGGACTGCATTTGTTATCTGAARTCRTCATCGAATACCACAGAGACRATGATCTCATGTTTCATACGCAAAATAATAATCCACAACCAATATACGGTAATCATTCAACATCTCAGTCAACGTCAAATGAGAGAATCTAAAACATCATCTTAACCGTCCATTTCGTGATTTCGATCAAAAAACAAAACTCTATCATACACTATAAAATA

>Cs1g18190

CTGCCTTTATTTTGGGTAGATGATGCGGCAACRYAGTAATTTATAGGACAATGATGCATCATCTACCTAGTTGAATTCCACATGATTTTAATYAAAGAGATCGTTTGATTTTAACAATTAAATAAAATAATAAATGAATTTAATTATTCTTATATTTTTACATGTTTGTTTTCTCCAGCACGAAAAATAAGTTATTTGTATATTTGTATCAAAAttaagaaaaaaattttaatttaaaaatcacactaaagtgcgcatctttgttgaaaatatatattatatactatagtccgggctttatatagaaatataaaaatatgatctcttgttattTATTTCTACTTTATAgggcaaCGCATCATCttttCTTTAATTTCACATGATTTTATTAAAAATaagagatttttttttctgttTTAGCAATTACATGAAATGATAATCTATTTTAGTTGTTCATATAATtttacatgttttttTTTTCCTGGCGCTGAACGTGTGTTATTTGTATATCTGGTAAAATTAAGATAAAAACTTTAATTTAAAGATTATGTCTATCCTTTGTCTCGTTGGCTGAATACAACAACCAGCATTCAATATTGTCAAAAAAAAAAAAAAAAAACAACAACAACCAGCATTGAACCAATAAAAAATTCATATAACTGTTGCTTTATCTAAGCCGCCAtttttatttttattttttcgttaTTGAAAACAATTCGGTTTCAGTTTTAAGTTGTttcatnnnnnnnnnnnnnnnnnnnaaatgtaaaaTTATCTTAATAAAATCAATACTTTTTTTTTACTATTCACTGAATTTGGATGTATTTAATATTTACTTAACTTGCACGATAAACCATATTATTTTACCTTAATAAAATCTTACTTGTCGATTGGCTGACAGAGTGATGACAAAATATTGAGCCCAATttactattttcaaatAATAAGAGGGATATATGAGATTTTGTGTACCATAATTTCTATCAGKGCCTCCTTTTATT

>Cs1g16430

aatatctgctataataattgttgtaaatatttatataatacagtaatatgacGTGTAATCCGTATTGAAACAATAAAAaataaaagctgtccGCTCACGGGTCACGTCGCTATCCAAAATTGTAATCGCAAAAAGTGACCTCATGACAGCTTAACTTGGCGAAATAAACGTGATAACATGTAGCCTCTATCGTGCACGTATTTTACGCGCACRATCACTCACGTGTATTTGTTTCAGAAAAAGTTtgagttaaaaaaaaaaaaaaaagcagcATGGCTCTACTGAACTTTCAGAAGTTTTTGTGCCATGTATCACGTGTGATTTACATGTTTATAATTGATAATGTATGATTAAAAATAAATTATTTTTATATTTTTATTTATTAATCATTAATTACTCATGTGAATTTTTAATTACTAATAAaaaaaattaaaaaacatatGCTTGAATTTGGCAGGCCAAAGCTAAAACTCAATGTTTTGAGRTTGATCTATTAGAGCATAATTCGAGCWTATATTCTTTCAAGCAaagtagttttttttggtgaATTTTTCTATTTAAAAAAAATGCTGATATTAGTCTGTTAATTGGGAGAATGASGCTCTAGTATCCATRTGATATCSTAATTTTACCACTTTAGTTCATCATAGATATTTTAATACTCCATTTCATCCATTGTGTTCAAATTATATATAATAAAATAGTTCATTGTTGAAAAAGGTGTTACTTTTTAATAGATAAATGAACTAATTCTAATTTATGAGATCTTAGCTAATCAATTCAAAAGTGAAAATGTCTCGTAATATATATTTTATCAGTGTGTTTTTTATGTTGTAGTTATAACCCCAAGATGAAATATTGTATTAAAAATCTATCAGGGACTAAAATGATAAAAAATAAAAGGACTAAATTGTCATTCTTCCCCATTAATTTAATTAAACATTGCCATGCCAGCCAACCAGCTCCAGCTATATATAAGTGTGTGCTGGTAGCCTCAGTTT

>Cs1g17050

nnnnnnnnnnnnnnnnnnnnnnnnnnnnnnnnnnnnnnnnnnnnnnnnnnnnnnnnnnnnnnnnnnnnnnnnnnnnnnnnnnnnnnnnnnnnnnnnnnnnnnnnnnnnnnnnnnnnnnnnnnnnnnnnnnnnnnnnnnnnnnnnnntttacgcatgttataatttaataatttttaggtgcgaacatccacatattatacacttaatgctttaaaatattgtaatagattttgataatataacattataaaatctttttacaacaccgacgttaatattcatattttgataagcaagtttacactaaagaatgactctnnnnnnnnnnnnnnnnnnnnnnnnnnnnnnnnnnnnnnnnnnnnnnnnnnnnnnnnnnnnnnnnnnnnnnnnnnnnnnnnnnnnnnnnnnnnnnnnnnnnnnnnnnnnnnnnnnnnnnnnnnnnnnnnnnnnnnnnnnnnnnnnnnnnnnnnnnnnnnnnnnnnnnnnnnnnnnnnnnnnnnnnnnnnnnnnnnnnnnnnnnnnnnnnnnnnnnnnnnnnnnnnnnnnnnnnnnnnnnnnnnnnnnnnnnnnnnnnnnnnnnnnnnnnnnnnnnnnnnnnnnnnnnnnattagataataataaaaagggagctgctaccttacatgcacgtaaggtacagcctctcacaaaactcagatgtggatttcacttgggcccaccagcgagannnnnnnnnnnnnnnnnnnnnnnnnnnnnnnnnnnnnnnnnnnnnnnnnnnnnnnnnnnnnnnnnnnnnnnnnnnnnnnnnnntatataattgatccatttctgcacaagcttcaaattttcaaatcctcaacggccatatttacttttgtttttggtcgaaaatttcctttcattctggcaattcgatttcaaaaagcggaagcaggttgttacggtttttaccttaaaaggtttctgcggaagaaaaataagaataaataaaaccagaagaaagcaaaagagtagcggatcaa

>Cs1g15900

tcactctctaAGTAATTATATTTGTATCCTTTTTTGTAAGTTTCCTTGGTCTGGCAAATAATTTGACCCAACTTAATTCGATCCGATGTAAATAAACTCAGGTTTGGATCGGATTGAATTACTTATTCAATTCAATCGGATGATATTTTATGCATCCTGGACAAWTTATGATCAGATCAAGTTAAATCCTATCGGGTTTGGctgacattaaaaaAAAAAAAAATTTAAATCCAACGATCAAAGGAGTCACACTCACACGACACAGCACAACACTCACACCACASACACACACCCAATCTAAACCTAGTCCATCGTGACTCACTCACGCGCAGCACTCACCAAACAGGCAAACGCACACAGAGATCGCAGATGGCGGGACGCCAAACGCAGACGGCACGGCAGCACACACATTAGCCAGCAATCACAGACAGCACAACCGCGTTAAGCCAAGCCATCGGGAGTCCYCGACATGCCGACGCAATCTGCTGCAGCTCGACCGCTGCTGTCGCTTGCTCCACTGACCACCGCATTCATCTGAACGCGATTATTTTTTCATGGTATATTACTATTGAATTTTTTATCCGTGAATTATAAGTTCGTGTCGAGTTCCGatgatgtttttccaaaCCCGATTAATCCGAAAGTCGATTGCATTTATCTGAACGCAATTTTGCCCGCACCTAATTGAGTGTATAAGAACATACATCTGCATGATTTTAAggttggaaaattgtcYTGGTTCGATGTAACAGAATGGAGGCTCAAAAATTAATGGCAATGGACGATTWCAAATAACTCCATCGTCCAGGCAAGGTCTTTACTCTTAGACATTAAGCATGCGATGTAACAGAACTGAGTAATCCATTGTCATGGGTACTGAATTTACCAGTGTTAACATTGTACGCTGGTCAGTTGTCCTATAAACCCCTACACGGTTTGTCGTCTGCGACTCCAAATGCCAAATGTCTTGCTGTTTAAATAGGGTGGGTAAATCATATAAA

>Cs1g21430

GCATTTACCGCGAACAATACTTTACTCGTTTCAACTTTTTTCCTGGTAATTTCGGAATCTCTTTAATTTGCATGTATAAAATAAATCTAGTGGTTTCTTTTAATTAGTACGTACGTAGCTTGAAGAAATAAGAAACAGTTWYAAGTGGGGAATCATGGAGAGCTATTTGCAAGAGAACTTTGGGGTGAAACCAAAACACTCTTCACCTGAAGCGTTGGAGAAATGGAGAAATTTATGCGGAGTTGTCAAGAATCCGAAACGCAGGTTTCGGTTCACTGCCAACCTCTCCAAACGTTACGAGGCTGCCGCTATGCGCAAAACCAATCAGGTTCCTTCATACTTCACGATCGAAGTATTAATTGTCAATTTCTTATCATGCATGCGACTTCCCAATATTTTCATTCATATTTATGAACTTATTAAAGATCATTTGCTAATCATGATCGTTCATCATATTGGTCGTGTCATCTCATCTGGTGAGGCGTGAGGGCTGTAATAATATTTCTTTTAGTAAAACTAAATTGTCATTCTGATATAGTCAAATCATGCACGATACCAAAAAACAAAAAAGAAAATAAATATATTCTCCTTCATCTMTGCCACTTTTTTAAAACTGGATTCTCTACCCTGCTAGCAAAAGATTTGTTTATGTATACAGTGTATATATATATCCGGAGATGAATTATTTATTTATTCGTTTATGACTGGTGCTTAATTACAAATTCTATATTTCACAAATTCATCTCGCTTGATTATTTATAATTCCCCTGAACTTGAAATAATTAAGCAGCAAACCCTGCTTGAGATAAATGCCGCATGCATGAACAATCGTCAGTGGTAGAATGGGCATTCTGAGGGCTGACATGATCTCGATCCTTGCAAGTGACTCAACATATCATATCACACTTCTTGTAGAATCTAGATTCTTCTATTTAGCAACAATTCCCTGATGATGATGTAACTCATTTTTCAGAGCAGAGCAGTGTAGGGATCATTGCTTT

>Cs1g08480

TAACATATAAAATTCATACTTTTTCATGAAACTAACTTAACCATATACACGAATCCAACACAAAAACAAGCTKTAATATCAATGAAGGAAAATAAAAAGATTCAAGAGCTTACCTTTCTTCACAAATCAAGCAGCGAAATAATCAAATGTTAGATTACGTCAATGGCCGGAAGGAAATCATAGGTGGAAATCCTCCTCTTGATCACAGTATAAATGCCTTTTCAAAATGGGTTATTCAAATGTTCTTGGTTCAGTATTAAGAAGAAGAGAAGAAGTTGAAGTCGAAGAAAAATCTGCATTCGATTGGAAAGAACGTATTCYACTATTGGGGAAGTTGATTCAAAATCAACTTRCCTTTGAYCAACTCCCAAAAATCGTACGTAGTTACCATGTACGGGTCGGGTTAGGTGATGGGTTGACCCACTTGTCCAACATTTCTAAACATATAGTAATATTAATGGACAAGGACACTCTCCTTARGTTTTTTTGCTCTAAGTCTCTCCTGGTGAAATAAATTAGGGCAGATCAYTTCATGACCAATGAGAGCACAAGTTTGGCCAAAGAATATAAATTTTAAGAATTAAAGAGAAAATTGCTTGAGTTTCCAACTAATCCCCTTTAAATCTATTAAAATTACCTCAATTAAATCTACAAAAGTAATTCCACAAacccataaattttaATTTTTTACAACTTATTGTAATGGAATTAAACTCTTCATAAATCYTTTTTTGTTAATTTGAAAATAAGTAGTCAAGATGGAGTCTTAAATTTGATGCTGATAATTAAGTCAAAATATTTCACTTTATTTATAATTAAATAATTAAACCTATTTTTTCAACTGGATATAAGATATTGTGAGGAAATACATaaaaacaaacaaacaaacaaacaaacAAACAAGGCAATCCCTAACATACATTTAatgtacgttactcagttactccactcACATYAACAGTGAGTYGAGGAATCCAAATGAGAAATGACTTGTTGGTATT

>Cs1g22910

AATGTCATTGTCGAGCGTTTCAGGCATCTTTGACAAATAAGTTGTTAAGATTAATTTGTTAGCTATGAACAAAAWAAAATYTGCACATACGAGGGCGTATATCAATTTGTATATGCAATCAAAGCTTAATTTGYATTAAGGGGRAAATGCATAAACATGGAAATATGGYAGAaagttaagcgaagAGGGAGCAATTAATATGCACATGAGTATATTGAACACCCCCCTCATCATGTTGATATTAAGGCTCCTTTTTATATTACGGTGTTGAGGCTAAAAAGTtaccctttcttgygAAATCATTGAAATGATAACTTTAACAATGAAATAAAAACTTTAATtgcggaaaaaaaaagttaatAGTAGTGGATAAATGTGATTCTTAAAGTTGACAGTTTAAAAAAATTAAAATATTAAAAATCTTTATCAAACAATTATATGAAAATATAATTTAATTTTTAAACCATAATTAGAAGTATTTACTAAACTGAACACCAGTAATTGTTCTAACCCAAAGTCTAAGAAAAATAAATAAACTCAAATTAGGAAAAacttTGGTAAGAGAGAGTTGGGCAATAATGAATATGTGTGTGTATAATCATTCTTTAGTAAGGGAATTCtcatttttattgaggattctaataaaataaaataaattttcatatttcaatatgctnnnnnnnnnnnnnnnnnnnnnnnnnnnnnnnnaattatatattttttttatattttagactcgtgtatgtatatgtatacatacatacatatacacacacaaatacgtatataatcatttttaaataagaaaATTCTCACTTTAACTGAGAATAttaataaaaaataaaaaATCACATTTTAATATGCTAACAAATAAAAAATCTATTTTTTAATGTATAAAATTTTATATTTTTTTTAATATTTTAGTGATATAATTATTTTAACAAAATAAAAATATACTCATTAATGAATTACTCTCTCTATATATACATGTATAGGGAGGC

>Cs1g18220

cgaaaagactcgTTGACTAATACAATCCATCTCCTAAATATCTCACGCGATCGTCTTCTAATGAGGCCAAAAAGAAAATTCTCACGaacacagcaccaaCCCGACATGGTCAGAattatttatttatatttcaataaccctaattttgattgannnnnnnnnnnnnnnnnnnnnnnnnnnnnnnnnnnnnnnnnnnnnnnnnnnnnnnnnnnnnnnnnnnnnnnnnnnnnnnnnnnnnnnnnnnnnnnnnnnnnnnnnnnnnnnnnnnnnnnnnnnnnnnnnnnnnnnnnnnnnnnnnnnnnnnnnnnnnnnnnnnnnnnnaatgtTTAATTATAAATTTCACCGCCAATAATCAGCCGGCTTACCTAGTTCTAAACTTAACAAGCATTGTTAATAAAAATTATTTAACATGTATTATTTTGGGATCAATCTGGCCTAGCTAGCTAGTTGCTTTTCAAAGTTAGCAAGAAATTAATATGGCGCTGAAAAGGGATGGAAGCACATGCTGACTTAAAAAAAaaaaattttaattgtTTTTGTTTAAATACTTTATTTAGCATTAATTACCCTAAAAGTCTTTTTCCAAAATATATGAAATTTTTAAAATATGAAGATAGGCACTTCTTTACTCTTTTCTATTCTAAAAAGTTGAGATTTataaattgtgtcaAAATATTTATTTTTTCTTTTAAGGTAAGATAATTTTTTTCCAAATCAAAATTTTAAAACGCAGACTTGTGTACTTATAAAAGTTTTTGCACCTCCATGTTATTTATTTATATACatatatacgaaaattCTCTCCTACGTACCTTggcgcacaccaaaaaaaaAAAAAAAAAAGAGATTCAACGTTTTTATGATGTAGTCAAATAATTTTCATCATTATAAAATGATTAAGGATAGTGTAAAACACTAAATTAGATTTATTTCTCTCGGWCATCGCTAGATTGTATATACACGCCGAAGAAATTAATAAAAATGCATATTGACTTTCTGAC

>Cs1g19170

CAAGAGAAATTCTATGGTTTGGTTRCGGCAATACTACTTGGAATATATGATCCATATAACAAGAGAAACGTGTGTATAAATTGAGATgatatatatataTATATWAAATGGGTTCGGAGTTCCATTTTTCAAAGTCTGTTGTTAAGAAAAGGTAATTGCTATACCCTAATTTAAGTTATCTGGTCGTTTTCGGCTTCATAACATTGCAACAAACCTAGAAAGCAATTTGGGTGCGTAGCAtaaccattttttttttaagcaaTCTTTAAGAAAACCAAACTCTCTCTACCTTAGAGAGATACATTATCATGGGACCCCTTRAAAAGCCAAAAWttttgtttaagtgtGAGGATTGTTCAATTTTATGTTATTAGTCCAGCAATTAATTATTTTCAGTCAAWATGAAATTCTCTTTTTACCACTCTCGycatgtattgtaaayCAGTCATTTAGGCAAGTTTCTGTCACATRAAAAGGTACAGTAGGCTCRGGCATTGGTCAGCTTGATTCCCATACCATATAAAAGGCTATAAGCTTAACTCATACTCTGAGCTAGTATTTTGAGTATGAATTtgacctatgagtttttataagtaTTAACTAGTAAAAAGTTAAAATTTTTAATGTGAAAGTGAATATTCATWTTAGTAAAAAGTATTGAAAATTGCTAAACATCTAAAACAACCCAAACACAAAAAATGGCACATATAATGTAGTTAATCTAACAAAAtccgnnnnnnnnGAAATAATGAAAAAAGTTACAAATTGTCAATGGATCACACCTAACTATAATAGAAACATCAAACCAACTTTATTCGACCAAAATTAAACGTCATTGATTAGTAAACTAGCCAGTTAGCCCACCAACCTCTATCTCTCACTCTCACATAATTCAAGTACTCCAATGACACCATTATCGGCCTTGCCTTTACGGACACTCAACCTGTCCATATACCTTATGTGATGTTTGCAATATTGTTCTCAAGTACAATCATGACCAC

>Cs1g18480

ggatgtacttcaacgagaccagctgggtttagcagacatctggtagtagggttccacgaacaggtatatcttgtaagagtctagtagcaggggaacaggagcaggaatattccgctcgtccacgaatacgccatccgcgagggcggtcttctgttagaagtataaagccattcctgctaagggttgaggcgaggagacccggtcaacgagagttggcgagacgtgctctccaatccgagaatcaaggcacgaaggtcatgcaaccacccacgattacagaaacggagcattcactttcgagaggtgggaggataacgggcgtgaatggagggaaggtctgaattcaggagaagcctataaaaaggtagtcaacgaaggtaaaagggttagaatttttgacattgaaactgagataaagagagcattgagtaatcgataccaaaaataagccataaaattagtagctaacgttcccaggaagacaaaaccttcatttttgacttgggcgtcagagggtttaggccgagaaaacaatcggcgtgctctgacttatttctttgtacgcatgaatttttggagaagaagtcatacgaggagagatcctgatcgtggtcgagttgattgagcagagatcctaatcgtggtcgagagggtaaccagaatctcgcatcaataaagaacatatctttaattaaaatttaaagaataatacaaattatgaaataagatagtgttaaattttttttcaaggaataataatatttaaatatttacttgaatttctttgggataaaatttttaaaatatttatcattattcataaattacctcgattatttaagcagttcggaaatttaaagggggtgtcaagagtcgagactcaaaagcctatttgatgacaaaagattgcgtcatccgaagttgtcaggtgttagatttgcgtactttgtaacaaactagttcttcaactaacnnnnnnnnnnnnnnaaaatggatggaaa

>Cs1g07290

nnnnnnnnnnnnnnnnnnnnnnnnnnnnnnnnnnnnnnnnnnnnnnnnnnnnnnnnnnnnnnnnnnnnnnnnnnnnnnnnnnnnnnnnnnnnnnnnnnnnnnnnnnnnnnnnnnnnnnnnnnnnnnnnnnnnnnnnnnnnnnnnnnnnnnnnnnnnnnnnnnnnnnnnnnnnnnnnnnnnnnnnnnnnnnnnnnnnnnnnnnnnnnnnnnnnnnnnnnnnnnnnnnnnnnnnnnnnnnnnnnnnnnnnnnnnnnnnnnnnnnnnnnnnnnnnnnnnnnnnnnnnattaatataatattagtgacattaataaataacaataacgaTGATCATGATGATTATGATGATGATAATAATAATATTACCCTTTCTTAATTAAAATAAGTAAAATTTATTAATGGGAAGGGGTTAtatgtcttttttttaaataTTAGGAAATTTTATATTCCTTTCCCCTAACGTTGGGGTCCTAATAGTTATTTTTTTAGGTATTTGTTATTGTAAATGTACAATATTAACCTTTTTTATTAAAATAAACGGAATTATTAACCGAAAAAGGACTTACAtttttaaaaaaaaaaaTTAGGGCTTTTCATGTTCTTTTTTCCAAACATTGGGGACCCAACAGTCCTTTTCCCTATAATATATAGAAACACAATATTAAATTAAATATATAAAAATTTAAATTATTTTTCATATTACATAAAATAATTTTAATATtATTaaatcatgagatatattttattaactgtaaaatctattnnnnnnnnnnnnnnnnnnnnnnnnnnnnnnnnnnnnnnnnnnnnnnnnnnnnnnnnnnnnnnnnnnnnacattttaaaaattattttatgtataaaaaaaattctaaaattgttagtctactagacaaattaaacaatttaaattatatttatatttattaaaaaattnnnnnnnnnnnnnnnnnnnnnnnnnnnnnnnnnnnnnnnnnnnnnnnnnnnnnnnnnnnnaataaatagtaa

>Cs1g08260

ATTAAAAATTTYCAAAAGCCCTAAATTTTATACGTATACCACAGGCAGATACATGAGTAGGCAGGGGATAAACATRCATTCAACTATAGTAGCACAATCTTTCATGCATTTGCATCTCAATAGGTGAAAACAACATATTACTCCCTAATTTATCATTACCCCCAACAAATTTGTTACAAATCGATCTAATCTATATTCTAGGTTTCTAGTAACAGCGATTAACAACAATCAACagaCATTGTTTTAAAAACATTGTTTCATGTAAAAAATTWAATGAAAATAAATAAATAAAACRAAACTTACTTGATTGTARCTTTAAACACCCCAAAAACCAAAACGTCGTGACTTCTTAACAACTAACCACTTTCGTCAATGCCTCTGAATCTTCGAATTAGCAAAAGATTTGAAACTTAAGTGTGGTGCATTGATGAAAATRCATGGAAACTACAAGAAAAATGGTGGGATTTTGTTRCTTCTTCATGCATGGTGGTGGGGAATAAACTATGGCTGTTTTCGTTGCTTAYTGTGCTGGTGGACCACTATGccttctttttttttttttccttgtTACAGATTCTARACGAYGTTGTTTTCGAAGCAYATGTGAAATTATYATTTTATCCTTATGACGTCAGCAAAGTCTTAACGATTTGAAAACGGAGGTGGAGAGGTGTAAAACAATATAAGYTCAAGTGSAGCGCAGCAAAGAAAATAAAGAATtatatatttttaAAAATGTGAGAAAAAAAAAGGTTGACGGTAGATAATTTTCCTTATTAAaatatgcttagtaatggtaaCCYTGAAAGGTAAATYCAATAGTATAATTTTAAAAACTAGAATGGTTAATTTGTTATTTCAAAAGTTTAGGGAGTRTAATGAAATTTAACTTGTAYATATTGTATTAAATTTGAGACGTTTTGATAAAAAGGAAAATGTTTAAAAGTGCTAAAATTTGAACCAAGTGATGAGGGTGGTGAGGTAAGGTGAGGTTTGTATAA

>Cs1g22990

CACCCCTCAAATATTAGATTAATGAGTTAATGTATGGCGGTAGTCCTAGTTATTTAGTATGATTGTAAATATTATAATTGTAATGTCTGATGTAATATTAGTAACAATCTGTATATAACAGAATTCAAAAAAAAAAATTGTCACGAAAGTAGCTATGCTAATTAGCTTTGATCTTTAGGATGACTTGGTTTATTAAGCAATTGCAACAAGAGGTGACTAAGCGCTAGCcaaactaagtaaggggtaacggtggatatagacactaaTAccacaACAATATATATATATAAACGAAAAAGtGGGTGTttatataattattctcatgtgcgggtatactctttttttttttaatgaaaacacatattattaaactgtataatttaattaagtgaattttcaatacactataaaattatgaaattaaaaaatatatccttataaaaaaaattaggacggtactagaaaaaggtgacactaagtcactaacacgcgcacacacatcagatatggtacaaggaataggtgGAAGGTATCTGGCGTACCAATCacgtagatattatattatattttaggGGAGAAGATATAGAGGGGCCTCGATTCGATTGAATTTGCTTGTCCTCCTTCCCTAACGGAATTCACACGCGCCAGACGCTAATGMAACAGAAAAGAAAATATATAACGGCAAAACGACGCTCTGCAGAAAGAAAAGAGAGGAGGAGGAGGGAAGAAACGAAGACCAAAAAAGAAAAGAGGGAGAAAGGAGAGGCTTGACTTTAGTTATATTATTATTTGCCCCCTCAAAACGGAGAGAATAGACCTTGGTAAGACCCCGCTTGGGCGGGTAACCGAACGGAGTAGGTTGTAAACCGTGTTCTCGAATCAAACCACCTCCTCCGCATCCAATTCCAGTCGTTACAAATTCAAAAATACAATGCGTATCATTTGCTCCCATTTTCTTCCTCCTCCCTCGTAATTGAAAGAAAAATAATGATAGTGTCTGG

>Cs1g08900

nnnnnnnnnnnnnnnnnnnnnnnnnnnnnnnnnnnnnnnnnnnnnnnnnnnnnnnnnnnnnnnnnnnnnnnnnnnnnnnnnnnnnnnnnnnnnnnnnnnnnnnnnnnnnnnnnnnnnnnnnnnnnnnnnnnnnnnnnnnnnnnnnnnnnnnnnnaaagtaattaattaatggaatttgcagtcaatcaaatggttaatttaggacacaacagaaaaaattatgaattaaaaaattttttaactttaatagatgccacgtcaacaacaccaacaccaatcaccAATCAATAAGCACCGCTTAGAGAGTTAACGGCAGCGCAGCGCAACCGTGCAGTGGAGGCAAGGCGACCTTCGTGCGGACATGCGTTGAGGATCTTAGTGCAGCGACCAACAACAAAGTGGTGAGGCCTAGCTTCCAGCCGCAGCGAGCTTCAAACAGCAGTGACGACGATGAGTTCAAAATTTTAGAGAGACGAAGAAGAAGAGTGCCGAGCTTTAAGGGCTGTTTTCCCTAACTGAACTGAAAGGGTTTTAGTTTTGAAGGAGGGGCGATTTGTAAATTGCAATTTGTTGACTTCTTGTGGATCtatgtagaatgtgttnnnnnnnnnnnnnnnnnnnnnnnnnnnnnnnnnnnnnnnnnnnnnnnnnnnnnnnnnnnnnnnnnnnnnntttcggaacttgcttcatacaaattaacaaattaaatGTTTATTTTCAaACTTAttaaagttcagggatctatatccgaTCTAAAATGTCATGCTTTTATTTCTTCTTTTAAGTTAAGTTTAACAATATTGATATTTGATTCGAAATTCTATCTATCATTATTATTTAAATTAAAAATTTCTAGAAGTCAAACCAAAATTAGGCATCCAGATAGAAAAATATTTGGTAAATTTGGTTTAACGAAATTTCGGATTAAATATCATTTTTCTAAATTTAAAATTATAGTGTATTTATTATAAGAATATACAAGAAAAAGAAAATAACGGTAC

>Cs1g24210

GCCACTAACCACTTCCCAAATAAAGAGAGGAGGAGGCAAGGAGCTAGCTAGCGTTTCAAACTCTATTTtGATTTGtctggtgatatgattctggaatttagaggctgctcggaagacacagggattctctcaaggttgccacttgtcaccagtcaaccacccaaaaataacgtcatagtctcaaattttctttttttttttttgccctttttcactgcaagaatgaaacctATTCTTAGCTCCTTGctgAacttggcacgaCGATGAAGATTGAAGAGTGGTTGAATTTGCTTTGTTGTCTGAatGGGCTGTGTGGCCCATCTGATTTGGTTTCTCTTTAGATGGGTGCTAATGTATGTTGCTGCCATCCTTTTCATATCCTCTTAATACCCCCCAACCCACTGCCCTTTCTTTTTTCAGAATTAGAAGCCAACCCCCTTCTTTGTTTAAATAATTTTAAAAAATAAAAATCACCAGATACTTTAATTTTATAACCTAATTAAATATACATCtttgtaataaattttaatttccaaatactgtcaatacaatagaaaCTGCAAACATAGCAATAATACTATCGTATAGCTTTTTACGATTTAAATTTAGTTCCCATAAAATTTGTGTAATAGTGAGTAGTTAAAGCCAATTGATAAGGTAGATAACATTAGAAATCGTTCGAATAACAATTTAGTCAACGTGATTAGCTTACGCTCTAYTTAAAATGAAATTTGTTCTGATCTAAACGACTCGTGAGGAGATTTTGATATTAATATTTTGCAATGATATTAGATGATGCTCTMATTCAAACGACTTTATAAAAGAGATTTTGATATATAATCctaAAAGAggagtttttattagtcaaagactttagaaaagcaaaaacaattatgtatatatttttaataattattttaaaaattataatattatcaaaggaaattataggtttatttttaatcaactagaaacctgcannnnnnnnnnnnnnnnnnnnn

>Cs1g26060

TTTATGAGATCAGAATTGATTTTCAGGTTCARAgaatgcgctttaATCATTTAAGCACTTTACTTGYACGCCAYAACTTTTTRCGGAAGTAGATACCAATTATGCCCccacaattttttttttttaattgGAGCACTGTTATCAAATTTGACAATTAAATCATTAAGTGCACATCTAGATACTTAATTATAAATTAGAATAAATGAATGAGTTTGATTTCTTGGCATTACAAGTTAATAGAATTCTCGAATTACCACGGAAGTGAATATATGCTGGAAAGGAAACAAATGCAAAGATAAAACAGAAAGGGaaaaacaaaaaaaaaaaacagatCGAAATGGATTATTRTCATGTTCGCGTAGATGAGTTGATTATTGATAAGACGATTGCTTTTTTAATTTCTTAAAACTACTTATGGGTTCCCGTTCTAGTGTCCTTCTTCTTCACTTGATCTGGACAAAGTCAATATGTGCTTTTTGTGTTTACGAGTCAACAAATTTTAGACCTCGGTCTACTTTAAGAATCCTGCATCTATTCATYGTGATGATTTTGGAGCGAAATTTactttcttttttctttcTTTATAATATGCTAAAACAACAAGTTGCATTTAATTATTAATCCAGTCTGGGAACCAACTAACGCACCCTTGGACTTATCTGGGTCATTATATGAATAAACTTATTTTATTCTTTTGAACTAAGACTCTCTTTTAATTGGATGCTCTATGCGCCAATCCGTCAAGCCTTGTAAAAGTTTATTTAGAGTATCTTTGGCATTGAAAGTAATTGGAGTTAATGAATCAGCGTCTTCAATATGCGCTcttggtacttctTGAAAATTTAGCTTACATATCTGCCAGTCAAAATCTGCTAGGCAAGCTCTTCGCTTGCCAAGAKGCCACCAGGCTCAGCATTCCATAGTTGAAAGGTAGGTTTGATTATTAGCTCTTGAAGGGTTTGATTTATAGTAGTCTCTACATAATATTAATCTTTATTTCT

>Cs1g17170

TCTTTTTAGTGGACACATCAGCATCATTAATGCCGTGGTGTTCTGGAATTAASACATGTAGCAACTCCTTCACTTGTCGTTCCAGACCTAAAACGTCTGTCTCGTCTTCTGCACAAAAATAATCTGAAATTCTTGcccaycgctgctgctgacttcTATGGGTACTGTAGCCTCCTTGGTAAGGGCTKAAACTTGGGCCAGTATCGTTGACCTCGACCAGTTGCTGCTGATgctcataggaggaggaggaggaggaggaggaggaggagcccctGATTTCGAGTTCGCGRTTGAATTTGTTCATCCCGAAGCTGAATAAAGACCGTAAACGTAAATTCTCAAAYCTTGAGAAAGGGAAAGTAGTGACCGTAAGAATGGTTARCCAGACAGGGTCAATTCTCCACATCTGGCACACTTCCTCTCTTATGGCCAAGCRGTTYATGGCATCCTGTGCAGAGTAAAACGCTTGCATAAAACGGGCTTCATCATCAGCGGCCTCTCCTCCTGCGGAGGAATCCAAGCATGGCTTCTTGTTCTTGATCAACAATTGGAGACTTRCAATAGCTTTCAGTACTCGTTTTTGAGTCCACGAACTTATCGTTGCTCCTTCGTCTGCAGCAGGGAARAGAGTTTTAAGTTTCACTATCAAAAGGGACACTGAAGCTTCAGCATCCATGGATTAGAAACCAGGCCAATCAATAAAATAAAARAAAATTACGTGTTTTAAGCMGTATTTCAGAGAAGAAAATTGATGATCTCATGTATGAATTAAWATAGAAGAAGAKGAAGATATAATTTCCCCGTGAAGAgtcttattttcgatgttttcgAATTATTCTTAAAGCAAAGGGACTCTGCAAGTGGCCGACACTTTTTGTTTTGTTTTTACGTGGCGGGGGTGGATAAAAAGTAACAAATTTTCTCTCACAACAGCGACAACGCTGAATAAAATATTTTTCCATGCTCAAAAGCGATGCCGTATGTTATGGAAAAGGAAAAAA

>Cs1g19820

TAAAAGTAGAGTGGATATTCTGGCTTAGTTTTTTACAAGTCRGAGTTCGAGTTGCAACTTGTTTAGTTTTAAGAATTTAACCTAATCAGGTCCAAAAGTTGGTTTGGTACGGTTGGGTTCGATCcagatcgattcaaattcagtttaagttaaggttataatttcaggtcaaaaattcgttgtattaaaaatttttattagatttaattttttttttaaatgtgatccggtcgaATTTGATGTTTggttCGATTTTGACTCGAGCTGAATCTAAATCCGATCGATTTCTAATTATCAACCCAATTGATTTTTAAGATCAAACCAAATTGTAAGGACCGAAGACCGTTATTTMTATTCATGCCGACCCCCAGGTAAAAGCATCTTCATTTTCAAACTGCCTCGTGACTTTATTTATTAACGTGTAATATAATAGCMCAAAAATTTTAGTTCGGCCATATTTCTCAAACATTMGTTTTACATTTATAACTTCCAGTTCAACCATTGTGGCCTAAAATTCTTTTTGCCCATGGAATCTATTAAGTTAATCGTCTTTGTTATTACGTGACAAGCAATCTTTCAATCAACTATTTGTTCSCTCCTTTTAACCAATTTTTGTTCCAATGTTTTCTTTTCTAATAAATTGTAAATACTAGTTCATTCCATGATATACCCTATAGATGAATTATTTAAAATCAGAAAGTTATGATAATATTATGTATCTCAATAATCATAACATATATCTAATTACATGTTTTATTGAATAATAAATTTAAAACGAacaaaattattATTATATCATCTAAWAAATYAAATTGACATAACTTAAGATGCATATTGAGATTCCAACYTTGAGGGATARTATAATTTTATGGAGAGAAAAAAAAAATAGTTTGAACGAAATTACACGTAGGATTTTGAGAAGGTTTTATTTTACGGCGCGCGCGAAGTCCACCCCTTTTATCTTTCTTAGGCGCACAATAACTCAAAACAGCATTGTCAAA

>Cs1g14940

nnnnnnnnnnnnnnnnnnnnnnnnnnnnnnnnnnnnnnnnnnnnnnnnnnnnnnnnnnnnnnnnnnnnnnnnnnnnnnnnnnnnnnnnnnnnnnnnnnnnnnnnnnnnnnnnnnnnnnnnnnnnnnnnnnnnnnnnnnnnnnnnnnnnnnnnnnnnnnnnnnnnnnnnnnnnnnnnnnnnnnnnnnnnnnnnnnnnnnnnnnnnnnnnnnnnnnnnnnnnnnnnnnnnnnnnnnnnnnnnnnnnnnnnnnnnnnnnnnnnnnnnnnnnnnnaacgtttgaaaatatggtcaaccccaatctactcaaaactcattagtattgagctgacctcgtaagtacgacctggagcctatgttggcaatctggtttacagatacaacctgagatttatgtgaacaaccagatttaaaaatacatccttgaacttacatgaacgataggaaaattcaccagtcccacctgaaaatgaagaaatccgacctcaaaatttatttgtccgactcgggaattagcttgacatggaaaattattggtttaaataacgtggcctaatttttagggaagttgtacgccacgatttgttaaaccgtctccaattattaacaactcgcaactgctgaagaaggtgggctaaagttaaggtggtaaccactcacgaccaagggctatttaagctgaagagggcaacagagaaaggggttagcaaaaattaaccaaaaactctacaagtgacctatctaagagccttaacattctctacnnnnnnnnnnnnnnnnnnnnnnnnnnnnnnnnnnnnnnnnnnnnnnnnnnnnnnnnnnnnnnnnnnnnnnnnnnnnnnnnnnnnnnnnnnnnnnnnnnnnnnnnnnnnnnnnnnnnnnnnnnnnnnnnnnnnnnnnnnnnnnnnnnnnnnnnnnnnnnnnnnnnnnnnnnnnnnnnnnnnnnnnnnnnnnnnnnnnnnnnnnnnnnnnnnnnnnnnnnnnnnnnnnnnnnnnnnnnnnnn

>Cs1g15050

CCAAGCGACACATCTTTGTAAGTTTATTATATTcataaggctnnnnnnnnnnnnnnnnnnnnnnnnnnnnnnnnnnnnnnnnnnnnnnnnnnnnnnnnnnnnnnnnnnnnnnnnnnnnnnnnnnnnnnnnnnnnnnnnnnnnnnnnnnnnnnnnnnnnnnnnnnnntgtgtcatgtgtgcataaaataataataagaaaaaaaaatcaacagttacttaattcatttactttcacaattttttttaataatattataaaaatgtacactgaaatcagatttaatatggcacgacttagatacttaagctcatgatacgagttgaatgtgttttaaataacaaattgtaatgggatttcttaccttatTTTTGAtgtggtgtttatttttaatatggtgcaannnnnnnnnnnnnnnnnnnnnnnnnnnnnnnnnnnnnnnnnnnnnnnnnnnnnnnnnnnnnnnnnnnnnnnnnnnnnnnnattaaTTAGATAGGTTAAATACATTATtggtgtcactctTTAATTTGAATTTATTATTTAACAAGTGSATGTAATTTATGCAATGACCAATGRRTTTGATTYATTTTGGATTcaacgaattaattTTATTGGTAACTTTAAATGTTGATGGGTGATCTAATARGTAAAACTTTAGCATTAATTATGTACActataattatggtatgTAGAATACTAGATGGAATCTACCTATTTACAGATAMGATTAGATTGAGATTAGAGAGTCATAAATGTAAATCGCATTTAATTAAGGAAACCCATTACACAAACCCTTAAAACGGAAACAAAAAGATCAAATAATTGAAATATAAAAGATGATATCCAATAAAAGTTTTCCACGTGGAATTCRATACTAGaagaatccgcacGTGGATTCACCGTGTCAATAAGCAGTCAAAAATTCCGCGTATGAACAGAAAATCCTTGTCcattaagagagagaatgygaAAAACGACRTCATTTTGAGTTGTTaaaaaaaaa

>Cs1g26000

TGCTGCATTAATAAACTCAAAATcactcttatgttgTGTTTGATATATTTTTWATATATATTTGTTTTTATTTTCCATATAGTCATTAAATTGAGCAGTGAAGTTTCAACCAAGCTGCCACGTGTGTTGCCATRAGATKTTCTTTCAACASCAMTTCATGAMATTAYTGCCACTTGCTTATTGAATATATGGACACGTATCTTGTATGCTACAAAATGGACACATGGCCACTCATGTCACACTTGATTTTAATTTATTATTTATtattattttcaaaGTAATCCGATCTTTKARATTTGCATTAGATGTTAGCCAAAAGCAGTAAGGATAAAAAACACACGCAAGACGTCCATGTTCTTACTATTTTAAATAAAAAAWTGCCCATCAAATATTATTCTAAATTTGACATTTTCTTTTTCAGAAAAATATTGCTTTCAGATGTCAATAAATCTAAGTGTATAGATTTTAAGGGCCATTTTCACYTTATTTATGAAAGTAGAAAGAAATTAAGATTGAGYACTTGAGTTCAATTTATGCTTTCTATAAATTAACCTGCTTAACTCAATTTATACATAAATTATTTAATATTACAATTCTCTAATTARATATCRTAATTAATTATCAATAAGAATACACAWTATATCTCTGAAAGAAATTTTTTTAAAACAACACATAATTACAAATRAAACGCGTATTTTCTAATTTTAACTATTTCTTCACCATCATTTCGTGCTTGATTACAATAATTTTAACTAATTTCATGTATGcagtttgtttttttCTTGTTATAAATAAATTAATATTATAATGGGGTGCGTCCAAAATCGAAAAACAAAAATGGAAATAGATTTATTTAAGGATMAAAAATAAAATTAAAATATTTTGGGTGTTTATACTTTCTCTTTCTTTGGCTACAGGTGGAGCCCACAGTTGGCCATTTTGCATTGCTGGCTTTTAATCCATGTTCGTTTTCTTTTTTATAGGCCTTTGTTTTGTCTGCT

>Cs1g21580

nnnnnnnnnnnnnnnnnnnnnnnnnnnnnnaatataatatattttttaccatacaaatatacatttttaaactatatcaattaatatttattaaatattttaatattgtaatTTAAAAATTATAGTTGTCCAACTTTAATTTCGATCGGAACTTACATATATCTTGATAAATATTTCAATCTTTCCATGTTTCTGGGTTAGGTAATTATACGTTTGCCAATGSAGAGAATGGYGGTCCGATATRTTCAATATATTTGGTTCCAGCTATTATTAGTTATTTTATATTTTGTGAAACGTTATATCATGAAARAGTGAATCTTTTTGGTTTCTCTTTGCAGTgaatttggtgatccgaacttttcccggGAAACAGACCCCTTTCATTCTGAAACTATAATTTACATTTTCTCAACTTGTAACATAATTGTATTCGGTGGATTTGATTTCCAATTATGATTCTTKAACTCGAKCGTCTAATAAATAATATTRCTTCATCTCATTTTATTAATTATAKAAGTRCATAAAAAACTGACACTRGGTTGGGTTCAATAAATTAAATGTTTTCGAATATGATTTTAGAAAGcattttaaaaaattttaGTTGGTGTTCTAATAATTAAATTGCATTCAAGTATTTCCAAAATCTTTCTGAGCTTCAGGGTTAGATATTTATGCCTTTAATTGAAAGAATTTGGAATACTTATCAGCATYTATGTACTGCCCAGTGAGGGACTCCGAACAACTGGACCATGGCTGGTACTGGTGCTACATCAAAGAGAGCGTCATTGGTCTAAAAATGGTCAACGCATTTTGTCGCCATTATTAATTTTTMATTCCACGTCTSRGAGCTCCGCCACCGTTTAGCCGCGTCTGATATATGATGATAACTTGCGAATCAtccaggccccccctgtcaCTGGCCTGCCTACCACTGTCGCCATCGTTTYTTTATATTTAACAGATTAACTTTTCTCTTTTAGTTTCAGTTTATCATTACATTA

>Cs1g18670

ACTAGGACATGTGTGGGAAAAAATCCTATGAAACGTGTCTACCCAAATGATACGACKTCTTGGTTAGACAGYACAATACATGCAGTTTCACATAATTAAGAAATTAAGAAGTTTGATTATTATCGAATTTCATGCCACGTCATCAGAAATATGACTAGCTGTTYGGATGCCATTATACTATTTAGAAGATGATTGATGTTTGTGTTYGTacatttaattaaTTATTTAGCAAATTTGAGTAAATTCATTGACAGTCATAAATGGGATAAAATACAGAMAAATAATTGGCAATTATTTGcgttgaaaaaataagagATTATTCCAWCAATTATTTGCTTAACAGCATGTGTTTCAGCGTTTACATAACAAGTTTAAATTATTKATCGTTTATAATMAAATTTAAGCATCCACTAAATTACCAAATTTTTAAGAGTGGAATGGTTTTGCAACAATTCTATTCTTATAATATATAGTKYCAATACAAATTTAAATATATCAATTTAATGTCTGAATYGAGGTMTTATATYATCTATGTGTGAAATAAAATTCATTTTGTAAACAACAGAAATATTTAACATTAACGTGTGCCACGCGTCATTAAAGATTCCTAATAAAATCTYGTTATTTATTATGTAAAATATATTGACATTAACGAATATTCAacgaagtaaaaaaTGaATCaCGATCACCAATCAAGAAATTATTGAAAATATTATGTTATCATGTGTCATGTAAAATTTTAATGTATTATTTTTTAAAATATAAATTCAGTACTCTTACATGCATAAAGAAATCCAAAGTTATCAAGTAAATTCAAATTGTTGTTTGGCATGAATAAAAATGTAAAAATAAACTCATGGAAAGAAATTGAATAAAATTTATGAAAATAAATAAAYTTAATAGTTGTATATTAAAAACCGTACWARAGAGTGAGAATGCCTCACTCGCCAGGCTTTACTCTTTTTAATATTTTTCTTCTCTCACCCaaacgcatacgcgcg

>Cs1g03420

AAGTCTAATTGTGTTTGCAGCGACATGCCCAGCTGCTACTTTTGATGCCTCTGAATTGCTATCATCATCCTGTTAGATAACAACACAAAACTATTCATCTAACCTCATCAAGGGCTCGCCTGATAGTAGTAATTCTCACAATCTGCCAACTCTACTTATCTATCTGATACAAAATCAATTTCCCCAATAACAAACATGTGAAACAAATATATAGGTGAAAAGGTGACTACTACAGTAACTGACTTGAAATTTCATAAAATGAATGTTATAGAAGCAAAGTAACCACCTTTAGAATCAAAGCGGCTATTTGTTGAAGCGCATTTTCAGCATCAGAAACCACATTATCATTATCTTTAGAACTTATAATAATAACACGCTCTTCATGTCGCTGCCAAAAGAAAATCATCAAAGCACATCATTTATTCACCAATCAATGATTTAAAAAAAAAAACCCTAACTTTCCAAAAAAAAAATTAGCCCAAAAAAAGAAAGAGAACTTACAGCAATAGCATCAGCGATTTTGATGGTGGCCTTAGTCTCTTCTCGAATCTTCTGGATTCTATGTCCTTCTTTCCCTATGACCTTTCCAATCTGTCTTGAAGGTACTATTATTCTGAACAAGACGTCTTGTGCTTTCGCTCTCCGTTTCGGATCCGATCCTTCGATTTCTCCGTCTTCGCGGCGGCGCTTTCCCGAGACATCGTGCCGCGGTTCAGGTTCGGTGACGACGGACACGGTAGGGTTTACTGTAATTTCGCCTTGTTCGGCCATTAATGCGTAATAACGGCTGAAGTGGAGTGAGAATCAACGAAGAGATTTATCAACTTCGAGTCATTGTTGCTGCTCAAATTACTTCTTTATCCTCCCAATTAAAAAGGCCAAAAAAACAAAGTAAAATTTTTAGCCATATTTTATAATATCACTGAAATAAAGGATTAGCGTGTTATTATCCATTACCGCCAGCCATTCCTATTATTTTGTTTTTAAGTTAGAAAATTAAG

>Cs1g06830

GTGAATGACGAGAAATTTTAGAAtatattannngtattatgtcagtcatataatagataaATGATGTTATAAAATATGTGTATTTATTTTAAAAAATCATCATGTAAGTGSTGGTTATATTAAATTTAATTACAYATATCATACATGCATCAATTGGTTGTAATACATCGAAGGTATTTTAGAATTTCTCATGAATGAGAGTGTTTAAATTGGGTTTGTAAAAACATGTTTTCARAATCRATWTTTTATAATTTCCAATTTCGAAGTATTTTACTTTTAATTTCTTCGAATCCTCTACTCTTTTCTATGTATATTTTGAGAAATTGATAAAGAATTAAGATGGTGATAAAATGACAACTTAATAATCAGTGATTAGTGTGGATTATAATTTTATAAATAAAATGTTTAAACTTACATCTGATTACAGTTATTTTTATCACTATCTTATctaatttttnnnnnnnnnnnnnnnnnnnntatattttAGGTAAATTTATTTTCTTATCTTATCCCTCTCTTTTTTTATTATTTTTTGAAAAAAAAAATAATTATCAAGTGCATAAATGAAATAGTTTTGTATTTAATGTTCATGTCAATTTCAAGTTGATAAGAATAAAAAAGATACATAAGGTACTTGTGCCTCACTAATTTCTTTACTAGTACGTAACAATTACTTGCTCTTATCATATGACCATCATCAAATAATTTCTCATTTATTAAATAGTTAAAACCTCGTAACATTTACATTGATCGCATTAACGTTTTTCAAGACATGAGTTCTTTTGAATTTTGAGAATTCTTCTGCCAAATGTGCTTATTAAGAAAGGCGTATGATAAAAATCTAATTAAATTTTCTTTATTATTTGCAAAACTTttaatcacatttgCCTtttttttttttttttaagaaaaagcacacaattcacgtcccgggttgcgaattcattgataaaatgataAttaaaaGCGGGTGCCTTCTCTTTTTTTGAAGTAAATATTTS

>Cs1g15280

tagacttctttttctgcactttcagatggggaaaagtagcacaacacgttgcatttgaatttggagaaaatacacagatgattgaaatgttttagtgagtaagaatgcaaattgtgtannnnnnaacaTATACACATATGACCGTAAAAAATAAATATAATTTatcaacagagagcaGCTGCATAAGCCCAYAAATAACAGACAAAMAGCAGCTGCAATRGACTCAAATCACAAATCTCTCATGCCCAGAAATAACGAACAAGAAGAGTGCATAAACAGACAACTGGAGATAAAATACACCTTACATTAATTCAAAAAAGAARAGAGAACAGCTGCAATTGAKTCAAATCACAAATCAGAAATCTCTCATTTCTTCAACCTCACAAATCAGAAATCTCTCATCATCATTCATCCTCAACCTCAACATTAGCAAATTCAAAATTGTACCCAAATCAAGTGCGAAAGGGAAATCAATTGCAATTTCCCGAACAAGTTTCGGCTACCAGTGTTTACAGTGACTGTGGTTGTGAGTCCGTCGTGGTGGATCCCGAATGGCTGGACCTCGGTCAGGGAAGGAAAGTAACGACGGTGACCGTGGctgtgagtcggtcgtggtggatcccgaatggctggacctcggtcaaggaaggaaagtaacgattttttcggtaaggaatcccaggtgaaacgaattcttcggtAagGaAAGTAACGAATTCTTCTATCTGTCTCAGGTGAAACGAAGATACGAAGAGGAAGAGATTTACGGtGAGTGAAATATTCTATCAGAAACGGCaGCGtTTTGGagaagtaaaaaagaaacaaaaaagaaacgaaacgacgccgttttatttattttcggggtttgacccggggcaaacatcattttcctttttnnnnnnnnnnnnnnnnnnnnnnnnnnnnnnnnnnnnnnnnnnnnnnnnnnnnnnnnnnnnnnnnnnnnnnnnnnnnnnnnnnnnnnnnnnnnnnnnnnnnnnnnn

>Cs1g21660

CTATGCATTGATACAGRTAACGTTGGATTCGTTTGTCATAAYTTtacaataaaaaaatttttAaaTTAAAATTTTGACTGTAGATCTACAATGAACGAAAAAGTCCTTTGCCAATAACAACTATACAGTCCTAGCTTAAACAAAAAGAATTTTAATGGTACAAAATATAGTACAAATGACACCAAAATATGATAGTGGCCAATAGTGAAAATCATTAATTTTAATTAAAAAAATTCAAATTATTaannnnnnnnnnnnnnnnnnnnnnnnnnnnnnnnnnnnnnnnnnnnnnnnnnnnnnnnnnnnnnnnnnnnnnnnnnnnnnnnnnnnnnnnnnnnnnnnnnnnnnnnnnnnacatattattatattagtacttaatacaatataatacaatgcaactaatacaataaaatataataggttgAAAGCACCCATAATGTGTATGCATATATCATATATGCATGTGTGTGTGTAAAATCAGTTACCTTATTTTTGTGGTATAGGTGTTATTCGGatcatgtttttttttggtcgAATAAAATCAAACCAAATTTACTCATGAATATCTGCTTCTTGGAGTCATTACCTTATTAAACTTCATTGCTGTTAATtcCTTGataattacaaaatttattcggtagttagcttcaggtttttgttgcccagcaTGACGATGAAAATGCTAAAAACGCTATGCAAGTATTTTGGTAATTATACGAACTCATCGAATACTAATCTGTGTTTATCATAATAAAAATAATAGGCTATAGCCTGTATATTAAATTATAAATAACTCACTTAGCAAAGTCATGAAGTCATCGTTTGATCGCAAATAAAGACCATTTCCATTGCTTTGGCCTCGAATATTTTGTATTCTCATTTACGTCTCGTCGTTTATTTTAATTCGCAATCAGGCCGCAGTAACGCCTGACAATTTCCGCTACAGATATATAGAGTCAACAGTTGAGTCCATTTATAATTAACGACTATTTCCCCTTGTA

>Cs1g18600

TGGTGCTCGGTTCTCAAAGAACGTTATGGAAGGACAGGAGASTGGCGGTACAAGGTTTCTCACATCCCTAATATCAGAGATTGAATAATGGCRATGTGCCAAGAAAAAGCCCAGAAATTCtgagactgctgcaagtGCATRTGTCCCRCAGGTAACTTCTTCCTGTCCTCATTTTTTATCAGTTAAAATATCGCTTTTGTTCAATGCAAAATCTTGCACACWGCAGTCAGRGAGTAATTCAAAAGTATCACATTTATTTTAGGAACTGTTTTGTTGAGCYCCAGCTAAGTCTTCCTCATTCRCTKGATTATTGCAAGATTGAAAYTATTTTGGACTTTCCGGYGCTGACWGTCGTTAGATTTGTTGAAAAAATTAAAAACTTAATTTATGTCCTCATATTTWTTCTATTCTACATTTgcacttaaaaaaagtcaaTGGCACAATTATAAAAGTTAAAATAGCTAAGTCCATGTTAGCAATATATATTCTATCAACTACTCGTCCACTACAKGGTTCAGGCYTTGGATTAGGTTAGCTTCAGGCCacataarttwyagtttagcctcaAAATGCGACTTTCTTGTTGGGAACTAGTGGTTTGCTTGAGTATTATTATGATTAAGTCCACTYAAATTACTAATGCGGGGGTGACATGGCATGCATAATCAGCGGCTAATGCCAAATATTGACTTTTTATGTTACAGWACTAATTATTTCTCAGTAACTAATTTTTCGGTTTTTGTCTTGACAAATTTATCGCCACGTTCCATYTATTATGGCTAAATATTTGATTAAAGAAGTGATAATTTCAGTTTATATACAATGCTGTTAATTCMAAGAGGATTGTCTTCAAGTGGGGCCCGCTGGCGTGGGGAAGTAGCCGAAAGGGTCAAAGGAAAGCCYCGTAGTGGGAGGCTAACAAATTCTTCAAGCACTTCAAAAGCACTTCCTATAAAAGCAGTTGGTCAGTCCCTTGTTTGGATCACTCAaaaagccaagaa

>Cs1g25110

TTTTTAGATACATACGTAAAATAAAGACAACACGCAATCTGAGCATAATTTGCCTAAAACTAGAAGAATATGAACAAAATTTGTTTGTATTATTATAAGATAAGATCTAAAAATGTAGACAAAATATCTTCTACACATGATAATGTTCGATACCGTGCTCCAKAACATTCGTTTACACTCGAATGAAGATGAAGGGTGAAGGTTTGAGATAAGGATGATACATAATTTTCATTCAACAGTACATACTGTGTTCCCAAGTCGAAAATTGGGCCTAGTTCTTAGAAaaaaaaatatTTAATCGGCCCAATAATACGTCTACGAACCTGGCCTCTATAACTAAGagcacnacttcATGATCTTTACAAAACCCCACTATAAGGAAAGTTATTTTCAAGAATAAAAATTAATTTAATTACATTCActtagatttttttttcccctCTCRGTCACTTTTTATCRTAACCAAAATTGATTTACAAAAAATTAGAACAACCTATTAATCTTTTCTTATATCTTACAAATTAAACTTTTGTGTCAAATTGTYcatatatttttttaaaacTTGTCAAACTATGATTTAAAATATGTGAGCTATGAGAGATAAAATGGGGGCCAGCTAGCAAATCCATCTAAGCTTCTACTTAAAATCCACTTGCAGGCCCACCttttagaaaaaaaatcagtCAACTTAATTTAAAAAATAGAAAAATAAAAAATACTCTCGAACTAAGAGCTGTTGGCCAATCACATGATTAGACTTGTAATCTGTTAATGTAAAATCTAAAGTACGGAACGTGTCCAATTGAATGATTGATGGAGAATATCACATAATTAGCGTGCATTTATAGGCAATCAAAAAGCTACATGATCTGTAACTAACAGCCATGCATTTTGATTCGGTGGATTTAGAATAATCGGTTTTTCATTAACATAATTAAATgaGGgtTGtccACcagaaacccaacaggttaACGACACAAATTGAAATTAGCAGCCGTTGG

>Cs1g24240

AAAACAACAAAATGTACACGTTGCCATCTGCATtcactcaaactcaaTTCGCTTAACCGGGCCATATGCTGCTGATGCCAGCCCCTACACTCTCGCTGGCTTCAATTGACATCGGAATCGACCCTCCAACACCACTGAACGTGATTTCATTTCATCACGTGGCTCTGCTTTGATTTGATCCTAACACTTGGCTTCTTCTTTCTTAGGTGAAAGTAAATTCAAACAAACAGCATTTGAGATCAGTTTCGATCGATCGTTCGTGTGGTTCCCATTTTGAGATCAGTTCCAACCCACCAACAAAATAAAGCACTAAAAGTGAAAAGACCTGTAAGAACAATTCCAGCAAGTGTGATTTTCACAAAAGAAcccattatctgacttaattgatatatAATTACAATTGCTTATCCGATAATATTTAAGTTAAAATTGTCGCAACGGGTAAAATTATTAGCCAGTTGTTCAGAACTAATAGTACAGCTCAGCTCAGAAGCAATTAATTAATCAAAGAATAGCCAGCTGTGgaatatataatagtcgaTTATATAGCTGTTACATGGTAAAGTCTTGTGCTTCAATCACGCACTCGCTTCACTGTTTATTACAAAAGAACGTGAAAACTGAAAACTTTTGAAACAGCACCTGCGATCTTTTCCCTTAAGAGTCAAAATCATTAATGCACACGCACCCAACTTTATTCGAGCCATCATTTTCATTACTGCAGAAAAAAGAAAGCATTTACCAgtaaattacaataaaatcGTCAGTGTATACTCTATATGgtagtatactagtatatggtaatacagaatagaaacaaaaCATCAAAAGGAAAAAAAAATAGAAAGAAAGAAAGAAAGAAAATGCCATGCCACGTGTGTTTTAATCAACGCGGTCGACCGAAACAGCGACCAGCCTGTCAACTTTATCCATTAGTAGAATTATATATAGCAGcatctcttacaaaatgtTATTTTATTATATTACTTCCTTAACTTACT

>Cs1g08940

TATTTTTTAACAAGTCCTGCGTTCATAAAATATATTCTTCCTTGCAAGTTCATTTTTAGACTTCATCATGGGTGACTAACTTAATATATCATTTTTTGTTTAAAGGCAAAGATAAAGCTGATAAATCAATGAGTTGATTATATGCATTAATTTTGGGACGGCTGAAGTGCACTTAAGAGTTAGGAAAGCAAATCTTTTTGGATTGCAAAACTGATTAATCAAAAAAGGGCTCTGACTCACAAGCAAAGAATGTCTTAATCTCTTTTATCTTCAAAAATCATAAAAGTGCAAGGACTGTATATTTCCAAAGACTCTTTATTTTCCTTTATTTTATTGGAAGTCAAAAAGCTCTCCTGCAGACGTGTATGAAATAACATGGAGTTGCCCTCAAAATTTTAGTTTCCTTATGTCTCTTTGCCCAtaaaaaagaagctgttaAGCAGTGAATGAAATGTGTtTTTGTCTATCCAGTTtctgcacaaaacAAACcaagatgggaaagcactcatcattaaatggccttctttctttctttttttttgtttcttTTGTGAAATAGTTTCTGTGTTATTTGTTCTTTGAACCAAAGCCAAAGCGAAAAATTTGGATTTTATTAAGGAATTTCTTGGTCTGAAAAATGATCTCAAGTGAGATTTACAGATTATTACTTTGTTCGCAGACTGATAAGTTATCATTTGACCCAAGTTTTAAGCTGTTGGAAGGGGTCCAAAAATTACATTTAAGCTCTTAACATCCCTCATGCAGGTAACAGAAACAAGGACAACTTATTGTCATTTAATTAACAGTGAGTTGATTCAaacacgacttcTTGGTCTAGTTGGCCGTGGAAAGTTACTGCTTGACTCAAAAAGGTTAAGCCATCAGATAAGGGTCCAAAGATAATGCTTTAACTCAGACCAGGAATGAGAACTTGGCCTAATGTCATggagagggaaagttAGAACAGTTTTGAATGGTTGTACGGGAGAGAATGTCTTCTA

>Cs1g04300

AGAACTCGATAAACCATGCAATCTATAGACTTCTTTAAAGAATAGACAAGCAACTGTCAAGGCATCTGTAGtcttcttACAGGCAATAAAGTGAACCATTTTCAAGAATTGATCCACAACAACAAATATTGAATCCATACCCCTCTGAGTACGGGGTAATTCTAAAAATAAATCCATACTAATGTCAGTCCAAGATTGTGTTGGAATTGGCAGTGGCATATATAACAAACACACAYCCGCCCATATACACAATCACACTARTCACCTAGCACATCATAWATATATTTATGTCCTTGTATAKAATTCAAATTTYAACAAAAGTATCAAAAGTTAAATTCATATTCCATCCTATATACATGAAACRTCAATTAAATAATTTTATTTCCCAGGGTTGGTGGGAGAGGGAAATGATCATGTGACCTCTTGTTTTCACTATAAGGGGACAAACCAATTCGACTTATTCCGTCAGAGATACAAAAAGTTACTTCTTGCTAATTAATATTGTGGTGACATTAAAATAAAATAGTAACTAGCATATTTATGGACAAATCTMAAATATTTGTGTTAYTTTCATAAAATATTTAAGTGTTGTGCAAATTKTAATAAACAAGATATWGATGCACAGCTTTCGGGTTCATGGAATTTGCCAATGGACTTTATTTTATTTTATTCATTAGTTCCCCTTCTTTCTATATATTRAATATATGTTCTTATATAAGAAGTAACAAGAATTATTAGAAtttgatatatctcaatctgatctattgacccggagagcgacaatttttttaatattgtctattattgaaatcacctccattTGTTGacTTTGTATATTTTCTAAGGGATTCGCTTTGATTTTCTTTATTATTTTGGAGATCCGATCTTTTCAGAAGCAATTATGCAAGACACTTATAATAAATATGATGAATTTGGCTGCACTACACTATATGCCACTCAACACTCACTTTGGTGAAYAAKTTCTCAAAGCCAGCAAAAGA

>Cs1g18360

GCCAGTGGAGTATCCAGCTTAGCCGACATCTTTCTCTTCAATATCGTTAGCTGCTAAGACACTATCCTCGAACGAGGAGGAGGACGAAGAACAAGCAGCAAAGAAAGAATAGGAAAgcccagttggtGGCTTTTCAAGGCGAAAACGAAACGGAGAAGTGGGTGATAATGATGAAGAAGGTGAAAACTTTACATGGGTTAATGGCTATTGCTGGTTTAGAACTATATGAAATTAGGGATTTTTACGTTTGTGGCTTTATGCTTTTATATGTTCTTTTTTCTTTTTTAATAGYACTGGTGTAACTATGATCGGATTATTCWGATCGACCCGAATTTGATCAAATTCGATKAGACRACACAAATCATATCCACTCAAATTTGAAGCAAAATCCAAATCCGATCCTAATTTGTACGGATTGAATTGGATCGGATGATTCGGATCTATTCAAATCCTATCCAATGCCTAGGCCTAATTAAGATTTATTGATAAAAAAATTRTTGTTCATCAAGTGCGTGCAAAATTATACTTGAAAAGTTAAATTAATTTAAGWATCTAACTATTTCATGAAAAATCTACATTATCCTTAATAATtcatttttatacaacttaaaaaatttgaacgctaaacattcatttattgtttattttaccgttAATAGCTTCAAACCACATCGCCTCAATTCCCTTAGAACTTGAAATTGGTGGGTTACAATCGTGTTGTTCTTTATGTATCAAAGCGGCCCATTTTTCGGCTTTAACTAAGTACATTTCTAAAATTACCCTTTTAAACCTAATCATTTTTCGGGCGTAAAGGCAAGGTGAAAAACTTCGTTGCCGTGTAAGCTGTAAGTAACTCAAATAATGGCCATAAAAACCAAGACATTAATTATATAATTTATGCTTTTAATCATGCCACTCCGCCCGTACAATAAATAAACATTCTCTCTTTCTCATTATAAATAACACATAAACTAACTCCAAAACTTTATAAAATCATCTTC

>Cs1g06470

CAACATTGGCAACATTATTTAAACATTTTAGAAATGAACCATTATAACTTTTAAATTATAAAATCTTCACGAATTAACATTAGTCAATTGGTMTTGGGAAATTTTTGCTTTTTAAATGCCACGGACACCAAGTAGCAGACCTAgggatggcaatgggcaccacccgcagcgggtttgccattatcaaacccaaacccgcacaaaatttaaattaccaaacccacccgcaacccgcagcgggttttaattttttaaaaaaaatccacccacagcgggttttaacaattaccaaacccgcaatggtatccggcggattttttgcgggtttccgtatttaaaatcacaaatatttaatatataaatttataataataacctcaagttccatataacatattcaattnnatatttaaacagtgtaaatgaaaaattaaaatttccacatcaattcaacacaaattctcaaatttaagtataaattatacaaatctatttaaaaaannnnnnnnnnnnnnnnnnnnnnnnnnnnnnnnnnnnnnnnnnnnnnnnnnnnnnnnnnnnnnnnnnnnnnnnnnnnnnnnnnnnnnnnnnnnnnnnnnnnnnnnnnnnnnnnnnnnnnnnnnnnnnnnnnnnaatattttcaaatactaattcaaagggaaatgcctttagttttctttaggttatgactttagaataagagatgagccatatacacacatacacaactttgagccttttgctatttggtaatttgattaattacattattttctttatatatttttatatttaaattaaattaaaaatatttgaaaaaaattattgcgggtttggtggatatccatgcgggtatccaccgggtataaggttaataccaaacccacccgcatccatatgcgggttttaatttataatactaaactcgcccgcaacggataaaattacccgcaacgggtgggtttnnnnnnnnnnnnnnnnnnnnnnnnnnnnnnnnnnnn

>Cs1g03530

CTATAGGATaAAGTGATTTTACTAAAAATAAATTTTAAAAAAATTTACTAAACACCAAAATTATCTTTTAACTTTTCAAAATTATTTTTTAACTTTAAAAAATCAAATCCCAAATGAACCTAACTCATTCTTATAGCTCTCATGTTATGTTTCAATTAGCCATTGcattcatttttttcgataATAACTAATAAAAGTAGGTAAATAATCATCTATGATTTATATGTACATATTTGTGTGCGCACACACATATGTGTGTGTAATTAATAAAAATTAAATTAAAAGATAaaaacatgtacaatCTattaataagaaattgacatgtatcctgttacgcgtatccaatcaaaatacaatctatgagtttgtacagagcaaaactcttattattagagtgagactattggcacccctccaaatacctcttaaGaCGActttaataatttTACAATTTTAATCTTTATTAAAATTACATAATATGACAATTTCTAATTAAAACCCCACTCAAAATTCAAATAATTTTTTTCCTTAAAAAATCTAATATTTTAGAAATTAAAAATTTATTTTTTCTTTTAATATTTAGGTGAGGAATTTACCACAGTTAAATGCAATAAAAAGAAGAGAACCCGTTGTTTTTttttttttttgaatgaAAAGTCCTATTGTTGAAATAAGTTTAATAATAAAAATAAACAAAAAGTAGAGAATGGYGTTTACTTTAATAAATTTTAAGTAAGGATATTTTTATCATTAACAGAGGTATTAAAAGAATTTTATAATTTTTTAAAATATTTTAATGATGTTAATAAAGAATATGGGTGCTGAGAGTTATTTGGAGCCACTCTTAGTATTATTATATTCACTTTAAATTTTATGTCATCTTTGACCTCGCCCGSCGTTKCGCGTTCGGCGAGGAAAAAGAAGTARAGTTGGAACTCGGAACCGAGgagtattataaatttAAACTGCAACGAAGTAATTATACAGAAACGACATcgtac

>Cs1g25210

nnnnnnntaataataattgaaaatatttactctTTATTTTGCTAGTGAACAAATAACTAAAGGCATTTTAATAGCTTTAACAATTCATTCTAAATTATAAGATAAAGTAAACATATTAATGAGAATATATATCYTTTCGATTCTAAATCCCTCAGTTRAAATAAATAATTTATTATGAATCTCACTCCTTATTCTGTTTTCTACCCATTCTAATTTCGAAAAAGTAAATGCACCATAAGTGCATTGTTAGACTATGCACTAACAACATCACACAAAATATTACATATACTTGCAATCAGTAGAAAGACGAATAATTATTTGATTTCAAATCACCATTTATCTTTGAGATGTTGGAAGATTCAAGTGAAACTAATACATATTTAAACAAAATTTATGRGTGAAGAAGATGTACAAGTAGAGTAAGAAAGCCCAGGCACGGGCCCGCGGGGAAATTCTAAGCCCATATCGTTCATTCACAGAGTAGGCTTTTTGACCCCCCCGCCCCCCCTCTTATTTCCGTAtaaaagtttttttttaattgAAATTACCTAAATAATTAATGATAAGTTAATTTCCTTTATTTTGAATGAGAATTCAATTAAACACTTAGATAAATTATTTCTTaacaatttctttcacacctttctcATTTCAtaatttatatttATAttttaaattttaattgaaaatattttttTTAGAAGCTTCTAAGAATTTACAATTATATAGATAGAGATTGCGTAAgaagaagaaaataagttaagattttaagatttataaaTAttttATAAATAAAAACTTAAAATAAACtTTTTAGGGTGAAAATTAATGACAAAATGATTAAttttaatttttagcttAATCTTCTGTAAATCAAATTggggtattttagggaATAAAATttaaaaggggtgttcTAAGGATTTTTGAGGGGTGTTAAAAGATCCACTCGCATTCGTAACATTCACATKGTACAGCTCGAAGCCCATTAAGGCAGCAGG

>Cs1g10270

nnnnnnnnnnnnnnnnnnnnnnnnnnnnnnnnnnnnnnnnnnnnnnnnnnnnnnnnnnnnnnnnnnnnnnnnnnnnnnnnnnnnnnnnnnnnnnnnnnnnnnnnnnnnnnnnnnnnnnnnnnnnnnnnnnnnnnnnnnnnnnnctacgaCAAGATCTTATTACTTCATAATTAAAAAATAAtaaaataaaacaataACAAATCAACGCAAACCAGACACGGCATGAGTAATAATGAGTTATACCGAATTGACCTTTTCATAACAacataaatttatgccaaatcaccaaaatctaaatcnnnntaacaaaataaaatttacacatatacttaaatgttacgagtaaaatattatatgatttaatcagctatcctgtttatacattatttacttattaagttttaacgatcattatgtttaaagaaATTATTTGATATTTTAATGTTATTTATTTAATATATAAAAATTGTAATATAAGATAAACTAGAGATTTATAAGACTAATATGATTGTGAATTAAAACTATTTCTTAAATACACCAATATTTAGAATGTTTGAATCAAACATAAAACAACTCCATCTATTTAGTTTAAATTTTAAAGCCTAATTATTWTTTTRTTAGTATGAGATAATTATTTAGAATTAGAATACGATGAATGTAGAATTTTAGGAAATGTATAATAAAATTCTAtgataagttttaaaaagtttctgTATTTAAGAAAATATGGAAAAATATAAGGGGCTTGCGAGAGATTTCYCTTTTTACGAAATTTGGTTGTATCTAATTTTACGTGGTAAAATCAGAACCCCACATCATGACTCAGTCACCACGTGCAAAAGGACACTCAAGCACGGCGTCATGATACTTGCTCCCAACCCATCATAATCAAAACACGTGTCTCCTTTGAGTGACCAAAACCAAGAAAAAGGAYGTCTAAGAGKTAACAATGRATATGCCCGCACCTGATCAGTCCCTCCCTACACCATTAAAAAkwaaaa

>Cs1g03360

TAATATTATGTGTAAAAAATAAGAYTCATAATGTTAAAATCATGCTCACYTGAGTGGAGAGGGAATTAGATTGTTATTATTAAACAATAATtaaaaaaataatAATGTTGAGTTTTTTTTTTTAATGATCGTATTATAATATTGAGTGTTGTTATATCATTAATCACAATttcatatgtataatatatattatgcaatagacattgaaggctaaatgaaataatttttaacatgttcaatttctatgagataattttacatgCATAAGAAGTCGTATGCATATTACGCAAAATATCAGAAGAAAACTCGAAAAGATGGTACTTTATATTTATACATTGTTACTCAAAAAATCAGATTAATACTCCATATGATGATGCTTTATATTATTTTctacaaaattaaTTTTGTtCTATtGATTtTAtTAAAAATTTGATGAGTAATTATAAAATTTGtTATATTAGTTATAAtCGGtttAGGATGAATTGGAtTTATAAAagtataaaaattatgctcattaatattaactttccttattcaaataaaaaaaaaaaagggctaACGGTTCCAATTTTTATTTAACATTGTTCGTGTTACCCCGAGGGGGTTTGATAATTTTCTAATTTAAATAGCTTTATTTTGAATTTAGGTAAAGTACAYGTAGCCATCTGATAATTGCCATTGGCTCRGCCATCTAATCAAATagttcacagcccgcctGTTTCGGTAGCCGGATTTAACTGCAAAATGTAGTGACCGTTGGATTGCCTCATTAGCAATCCAACAGCCAATGAATTTTACATAAAAAATTCAAATGCCGCTGTATCCTGTATGTGACACAGCGCCGCACGGCAAGATACAAAAGATTAATAAATAATTTAACTGTTAATTGATAGTTAATAAATaaataaataattaaTCTTATTTATATATCAccacgaatgaatcaactgaagtaatgatgtggtttcaatgannnnnnnnnnnnnnnnnnnnnnnnnnnn

>Cs1g10660

AASGACCWATTGTGAGATTTGATGTGATATCTCGGTGTTTAGTTTTTTAATTTATCATTAATTAGTGGTCAAATATTTGTTACATTTTAAATGGTAGAAACACTAAAATTTTATctctgagtcaataTCATTTTTTtttttttnnnnnnnnnnnnnnnnnnnnnnnnnnnnnnnnnnnnnnnnnnnnnnnnnnnnnnnnnnnnnnnnnnnngtgtggtcaattggggtccgaccacaggtccGACCACAGGAATACCTTATCCTATCACTTGAGTATGTTAAATAAATTGAGGAGAAAAGGGGGTAAAAAAAAAAAGAAACAAGTAGAAAAATACTTgcgaatgtatttgattgtttaattttaataatttagaaaatttattcaaaaactaaaaaaaaataagaaattagataaatgatgaagggtattattgacatttcagcaaaagcaaaagtcatttcaataacctaaccgagagtgagagagagaatgagAAACTTATCTATACCAAATCATAACACTTTCGGGTGGAGTAGTYGTGTAGTACTTCTAATTATCATCAACAATAAATTATTTCATCTTTTCTATTATCCAACAGGTTTtaagtgaaaagygkgAAAAATTAAATwgtgcatttttagaatATCATATTTAGTCCAAATAGTTTTATttttttgttcatATGAGTAAAATTAGAATGTTAGTAAATTACACTTTTCGATGAatgaaagtattttgtgacctcattttttaagtcaagggttcgtgtatcatataaatattaatttttcttcaaattttaAAAAAATATATTCGTCTGATCtcgcgtTtagGTATTCAAACTATGTCATTATCAAATTATTCTTAAAGTTAAGAATTTAaatcttattttaaagtaaattttcttttaaatatttnnnnnnnnnnnnnnnnnnnnnnnnnnnnnnnnnnnnnnnnnnnnnnnnnnnnnnnnnnnnnnnnnnnnnnnataaaaaagaaa

>Cs1g21790

GATTTGGTTAGATGCTCTCATCACCGTACAGTAAAATgaattttatatatatattatcgCATACGAAAGAGAAACTTCAGTTCTTGCTTCTCACTACTCGCCATTGGCTTTTCTTCCACTTCGCTTCTTCAATAGTTTGGTCTGTTATTAATAAAGCCAATAATTTTAGGTCATTATTAGCGGAACAAATATTGATTATTAATATCAAACGGTTCTTATGTTATGATGGCGGCCCCACTTCCCCGCCTTGTAAATGATAMAATAATTAATTAATTGTCAATAATAAARCCAATTTAATGCGacgtctccccatttcTTTRTTTTAAACCTCAATCATTGTCAGAGCCGTTTGTGGCTCGCAATTAATTAAgcacttccaacttCACACAAGTTAAAAGGGCGAAGAGTCACGTGTTTCCGTGCTCCATCATCAATGGCGCGGATCCGTCATAATTGATAAACAAAATATTTTCTTTATTTCATAACCAACCCAGCCAACCAACAGCAactatgatttttttattttttttttgtAATTTGAATATAAATAAATATGAAAGATTTATTCATAATTAAAAAATTCTAAGTatttccaaatttttTCAAATGTTATCAGGTATAWTCTTTTTATAATttggttaaaagtaatTTTATTAAGAAGAAAAGGAGTGTCACTTTTTGTTCAATCCTTCTTGATTAAGCGGAAATGTGCTTTTTGATCAAGTGGCGTAGAGTCACGTTCCATCACTGATTTCCACATAAAAAGATGATTGAMGACATTTCAAAAACAAAAAGCAGACTACAGGGTAGATTCAAATATTCAATTCAACAGTGCTGTTTCCGTTGCTGTTGTGGTTGCTGCCCACTcataatgggcaggggcagccctgTGCTATCATTGCGTAGCACTCATAGTCTGATATTGTTTCTGACTCTGTTGTATCATAGCATCGTGATGAAGTCACCGAATCTTCATCGAGCTACCCTTGATTGAGGCCATC

>Cs1g14180

nnnnnnnnnnnnnnnnnnnnnnnnnnnnnnnnnnnnntcaaatggagaagaatcaacgatgatgtggcatctcaaacttggccacatgtcagaacaaggcttgaagattttctctgagcgaaaattgcttccggggctcaaattggtaagtttaccattttgcgagcattgtgttacaagtaaacagcatcgattaaaattcagtagatctattgctagaagtaaatgcattctagacttgattcattctgatgtttgggaatcaccggatatatccatgggaggtgcaaagtacatggtgactttcattgatgattactccagaagatgttaggtgtatccaattaagaaaaagtcatatgtattttcagtgtttaaagaatacaaagcacgggtggaacttgaatctggtaaaaagatcaagtgcttgaggacgaataatggtggagagtatacagacggcgagtttcttggtttctgtaagcaagaaggtattcagaggcagttcacggtggcatacactcctcaacaaaatggagtggcagagcggatgaacaaaactcttacagaacggataagagctatgttgaggactgctnnnnnnnnnnnnnnnnnnnnnnnnnnnnnnnnnnnnnnnnnnnnnnnnnnnnttttgatgAATTATTTTTCTGATACTTTATGCAAAAAATATGATGAAAATGAATTAGTTTAGAATATCAATAAATTTTTATTACATTCAAGATAAGGAGAAATTTGTATGAAAGTCGTGAGAATAGAAATTTTGGTAAAACAAAAGTCGCGGGCTTatttagaaaaaagaaaaAATTACATTCTATTTTCCAATTACTAAGGTCAGCCTTAACACATGCCGGTCTTTTTCTCCTCTACTTTCTAGAAATTTTGGTTTCACTATAAAAACTCGGTATATAACAAACTTATGAGTGTTCTTGCATTACCAATCCAATTAAGCACACGCTTACAATCTAAAATACTCTCATCTC

>Cs1g16190

AATTTTGTTAAGGATTGAGATATTTCCACCCCACTATTTGTGTAAATCTGSCCCATTTTTAGGAAATAATTACAATGAGTTGACAATATTTTTAAAAAATATAAATTAATAAAAKTTTTGTACATATAATTAATTAAATCCTTCTATTAATTTTTTTCTTAGATTCTTTGACCATCTTTAATCATAAATTttaatatannnnnnnnnnnnnnnnnnnnnnnnnnnnnnnTAAAAGACAARATTACYATCATAAAACATGAGTTATTATATCTAATAATTTAGGTATTTAATTTTAYGACCTATAATTTTGCAACCTAATAATTTACATGARAATTATGCTATCAAGACACTATGTTAGTTTAATTAAAAATAATAATCATACTAATAAAGTAGTATTAGAAACAATGAGTTTAGTACAAATTGGAAAAATATTGAAGTTAGTGTAAATTCTTGATAATTTTATTTTACACGAGTAAAATAGTTAATTTAAACTTATGTTAAAGCAATCTTTGTAAGCAAATATACTAAATAAATTTAAGTCACAAAAAAATAAATTAAAATAGATATAATTAAtTAATTCTCCaacttaatttgaaaattttaaaaataatgtgtaatttttataattattttttaaaaatagggtggattcataaaaaatatggggtaaagatatcatcactcttttgttaaggattagtatgggttgagttggattaAATTTTTCTCAATCTACACTGTTTAGTCAACGGATTGACCTCAGTTTTTATCTTAACCGTTAATTACTTTCTTCAATCTTTGTATCCCAAGTAAAAAGGGAAAGGTAAATTGCCAATTTCTGATTCCGGAAAGGTTTTAGTTGTAGCTCCTGACCAGTTGCTTAATTTTCCTAATCCKCACGACCAAACTGACATCAATTTTTATCTCCAACAACGCAGTTACAAATTGCTGTCAACTGAACCCTGCTAAAACCTCATATCAAAATCTCTCTCTGCTGAATA

>Cs1g07740

AAAATATTGTCGGGAATCCTAAGACTTTTAGATAGGTGACTTATAAAATTTTTGGTTCATTTTTCTAACCCCTTCCTCAGATGCCCTTTTCAGCTTAAATAGGCCCTTGGTCGTGGGTGGTTACCACCTTTAACTTCTGCCCATTTTCTTCAGTAGTTGCACGTGGTCATCAATTGGAGACAGTTTAAATGGATCGTGGCACACAACTTTCCTAGAAATTAGGCCACGTTATCTCAATCGATAGCTTTCTATGTCAAGCTAATTCTCGGGTCGAACAAATAAATTTCGAGATCAGATTTCTTCACTTTCAGGTCGGACCGGTGAATTTCCTGGTCGCTCATATAGGCTTAAGGTTGTATtCTTAAATCAGGtcatttacataaatctcatatcatacttgtaaaccaggccgccaaaataggctctaggttgtacttacgaggttagctcgacgacaatgagttctgagtagattggggtcgactatatttctaaatattactAgAAAAATCCAACCTGAACAATGTTGACTCTTTCTAGAGGTTATCAGAAAAATCCAACCCGAACACCAGTCTCCTAGTTTTCAACATCRGGAAGCTATTTTYSGTGTTGGAAACTAATTAATGAAAAATAGAAAAATTCTTACAAAYCGAACACCARTCCCCTAGTTTTCAGTACTGAGAAGATATGAACGGTATTGGAAACTGAAACAATGAAGAAAKCGYARTATGGTCTCCAGCAGAAYAGGGGTAATGTGAAGTAGAAMAGKGATANTGTTGTGTTAAAATAGGCTTAAAAGTAAGAATTTGGCAAGAATAATAATAAAAAATAAaaataannaataaataaaGAATAAAGTAAATAAATAAAAAAGTGAGACMRTGGAGAAAAAGCATAAGCACGTGACTTAAAGGMAAATTGAAAACATACATTTTGTCTTTTCTCAATACCTTTTGGCTTCTGTTTCGTTTGGGCTTCCGTTTCRTCCTCATCCTCCACAC

>Cs1g01400

TTTGTKAGATGTTTATTGGTATCTTGCCATTAATTCAAGTCGATTTTATACTTAGAGAGGAGGTGTACAAATATATAATATTCAGGAAAATTCTATTTCCAGATGCGGATAATTAAGAGAACCAAATAACAAAACTCGTGTAAGAAGTCTGCTCTTGAGCTAAATGATTTTCTTTTTTTTGGGGTTTTATTTTTTGGATTTGATATGAAAGTGAAGCATATACTAGCAGTTGGATCAAGATGTATGATAGTTGTCTATTCATGCTTAAGAGGATAAAAATTGACACGGATCGAGAAGAAATTGAACTTTGAACACTTCCATGATGTTGAAACGAAGGCATACTAGACAAGTCTATGCCCAATTTTACCTAACGGCCTCAAGTTTTTGCTGAAATTTCAGCTTTTTAATATTTCAATCCATTTTTGAAAACACGTTGTGCAAGCTTTTGCGACATGTTGGGCTTAAGGATCACGGATGACAGATTCACAGCCAGGAGGCTGCTGCTAGGGTTGAATTTTCAAATGGGATTATTATTATTATTTGAGTTTTGGGATCAAACATTTTTAAAAGTCAAGTGGTCCTGGTGACAATGTTAAATAATTTTGTGGCAGAATTTGACTATTGACTTGTCTTCCAAAATTTTCGTTATCCAAGGATCCAAGGCACTGACCAACTGACAACCATTCAGCAAAATGTTTGACAAAATCGGAGCCAGCTTTTACCTGTGTTTTATCTTAATTTAGTTCTAGATTTTAAATCGGGACAGCTTGAGGTAATTAGCAACCAGGCCAGTTTGGGAATTGGGATGAGCTTAAGTTTGGGTCCAAAGCAGGCCCAAAAATTCAAATGAGCCCAAAAGCCGAATAATCACTTAGCTTGATGAATCAGTACATCAGTGCTTGTATTACCCAAACACGTTTGCACTCATGCAACCACGTGTCACTTGCCATGCAGAAGCACTAAAAATTATAAGCAAACTCCAAGCTTTCGATGATTCAATC

>Cs1g21390

AATGCTTTCAATTGAGACAGTTATTAATGTCAGACAGAGAACATGTCAAGGTGACTTAATTAATCACSGTTCATTCTTCAACACTGCATTGAAAAGAAAACACAAGACGAATTCTAGAATCTAGATCGCGGCTAATATTTGGGAAATTTATTTCTATGAAATGTCTCGAGATAATGATTCRAGTCTAAAGATAATTTGTTTTTCTTCCTCTTAGAAAAAGATGCTTTATTTAAATACAAACTAAAWTTGGTAAATACGGTAAGTCTTAATCGGACATTATTAATTTATTGGTGATAAAATTAGAGTAAAAYAAGTTTATAAAATTTGTATTCTAAATGATATTACACTTAACGTGTCATTCATCTATTAAAATTATAATGTTATATCATTAATTATAATAAAAAAACTCAATAATTACTAAATACATGACAGTAAatAatTagTGacGTATTAtTTGAGtcaATTTTATAAAATTATCATTATCCAYGATAATATTATAAAAGSTTGTTAAAAACTCATTAAGAGAGAATSTAATAAACTCTCCACGTAAATAATGATTAATTAGTTTGTAGRGMAGCCAAAGTCAATTACTAGATAATTAATTATTAAGTATATAATAAAGATTAATATTTGATTTAACTAAACTCTTTCGCCCACCAGTTAGCGTACTAAATCCGAAACAACGTCCGGCCCGCTAATAGTATTTCATCTTTTCATTTATgatgcatatatagccataATCACTGCTATGATTAATTGATTGTAATTACCTACTAACAAATTCTCACCCTCTTTCCGTACTCCAAATTAAGCCGGCTATTTCTAAAAATCAAAGGCACCCAAAATAAGCCCTTTTTAGAATTTCTTGGGTTTTATTCTTATCTTTTCTTGYTCCTAATTCCTAATTATATTCAAAACGCTAAAATTGCCGTCTATAGTTAGGAATTGGGAACAAGAAAACAAATAACATATTAGAAAAACAAAGGACTCCCCCAAACTTC

>Cs1g22965

caacaacgaacataaaatatgagtccctccgtaacagtgaacatgaaacataaaacatgaaacataaaacatgaaacnnnnnnnnnnnnnnnnnnnnnnnnnnnnnnnnnnnnnnnnnnnnnnnnnnnnnnnnnnnnnnnnnnnnnnnngaaaaatcagtacatgaaacattgcacaaccaacatgaaacatatcacttaaaatatgcaacaacgaacataaaatatgagtccctccgtaacagtgaacatgaaacataaaacatgaaacataaaacatgaaacatagaacatcaaacatgaaacatagaacgtaaacatgaaacgggtgcaaaaaaaaaaaattccggggtatttgaaaaatcagtacatgaaacataaaacataaaatatgaaacttaaaatgttaaacatgagataaatttcatagaaaatgaataaaatttttgtctttttgttaacacacattttattttattttttaaaatatttataataaaaaatattttttttcannnnnnnnnnnnnnnnnnnnnnnnnnnnnttattattattattttttaaatattattcgttttaaatattttttattttaatttatttaaataaaaaaaacatattaatggcagcaaccggctgtaaaaccggttgctgccagtcaaaaaagttgtttggctgccagatggcagccaaacaacaaaggggtcaataaattgaccccctctatgatacagggactttgagtccctgtatcatagccgtgtcctagtgtgagtgTCCTTCTGGAAtctgacttattagtttttataTCGATGGCTTTGTGAGAAGACATCAGACGGCTCCGAATGATGAAAAATATGGAGACAGTGAGCTGAACCCAAYTCGACGTCATAGAATCAAAGCTCATCACTTTTAGGTCAAATCAGGCTGTATCTCATACAGGCCTGTACRATAAGAAGACACCTTTAATTTAAGGATCACATTGATTATGGTCAAATCAGA

>Cs1g09360

aagttaattttggtgtttggtaaaaaaaatcaaaatcacttttgccaaaatcaactcctcctacagctaattttgaaaagcagggaaggagtaacttttagattctgattttgagaatcaatttgatcatttaatataatttcataaatatccttacaattattacttaaacccgaaattatccttatttaaattgaaaacaaaatcaatattttaataattttttattgtaattaacccacgcctttcgtttaccctatttcttcatattttgtgttctatcaaaacttatctagtgcaacaaagcaagatcaagtacgtgttccatcaccatattcaataacagtaagnnnnnnnnnnnnnnnnnnnnnnnnnnnnnnnnnnnnnnnnnnnnnnnnnnnnttgtacgtgtacatttttatatgtataagtaaatttcatcttacattatgtatattttagtcatttattttttttacagcagtttaacaataagatttaccaaacacccataactgctttaaaaatttacagtacttttgaaaataaaatttaccaaacacttaactgattttcttcacaactaattatttttacagcacaactaacattaattattttaaaaactgcagcattaccaaactGATATTTAGTTAAAATTGGGTAACTATGTGATAATTACTCTTGATAGTTGCCAGTTCCCACGTCCAATTCTTTCGTCCGGTGGCCGGTTTCGCCTAAAATTATAGAAGCCTTGGATTGCTTWCGCAATAATTTTTTtttttaaaaaaaacggctaTGTGACATGCAACGGCTGAATTATAAGTGGACCCGAGTTGAATATTTTAGAAAAGAAAACTTTTAGAAAAGTAACATTCTTTTGTTTGTTTGTGATTATCAYTTTCTGAAGTTGTTCAAatcaagacagcaggtcTGTATGACTCGGTTCTTGTGAGCCATGTGACTCCACGTAGTAACGTTCGTTGGAATGATTGAATTGG

>Cs1g26330

ATGAATATGCTTTGATTATTCGCTCATGATAGCTCAGRTGTTTTACATTCTGTAACTCTTTGATTTTAGGTGTCATGTATGTAGRACGCTARCTAAATTTTTATTATATTATTATTTATTGATAAATTTTTATTAATCACATTATCGTTGCAAGGTTTTGTGTTTTAAAAATGATATATGATGATTTCATAGTCAATGCAATAATATACAATATTTGCATGTATataactaagaagaagaagaagaagaagaacaggaggGAAATCATTTATAGTTTCATGTTTTTTAATCATTCCCTTGTTTTGTTAAATTTCTTTTGTCTTCATTTAGGTGTGACTaattcaTGTTTCTAGATTCAAAATAATTAATaattttttaaaaaaaaaaaaatGAACGAGGTTTAAATTTAAAACTCTTCAAAATTATccggacaattttaatactgaaTTGGAGCGCCTATCACAAATTTAAAACAATTATAAATCGATAAAAATTACTCTTCAAAATTTTCTAAAGCCAGCTGGTCCAATACTGACCCATCGTTGGTGTCAACAACAATTTATGTTTCGCAAATAAAGAGATTGYTTATTGATAAATTGATAATCAACAGCgtactgcatgtgatTCCCCGCAATCTTctcggcatatatattaacaaagattttgatatctAATGTAATAATaTaAAAAATTTCCTTGGAGGGTAATCATTAAMTGTTTCTCATTAGACTTTTCTTGATGCTCTCACCGATCTTATCAACATCCAAGAATAATTCAAAGTAGctgatcatatatatttcctTTGGACAGAAGTCTAGCCGTGTAGCGGACGGACAACGTTTATGATAGTCCTTTCCTCTCTGCAAAAATCTTGCCYTGTAGCCGACAGACAYCGTGTATCACCATCTAATGTTGAATAGTCAAATTAATAAAGAGTTAAGATAATGGGATTGAGAGCATGCACATGTTGGTACACGGTACGTCCAATATGTAGCTAGCGG

>Cs1g18320

TTGTGGCATCTGTATAGATGTCATGTTTGTTTGGTAACCTGAATATTAATGCCATTACAAGCACAGATGTTAACTCAATACAAGCGTTTGGTTAATTGCAAAAACCATATTTGAACCATGCTCATATGTTTTATGTAACAATAACTTCTGCTGTTAAAACTTTATGCAGGAGGCATTATACAATATTGCCCGTGCATATCATCATGTTGGACTTGTATCTCTTGCTGCATCATATTATGAGAAGGTGCTTGCTACGTATCAGAAGGATTGCATCATCCCTGGATTTCCAGACCACATGGAGGATTGGAAACCAGGTCACTCTGACCTTCGCAGGGAAGCAGCTTATAATTTGCACTTAATTTACAAAAAATCAGGGGCTGTTGATCTTGCTAGGCAGGTCTTGAGAGACCATTGCACTTTCTGATTAATTGTTTTCACAATGACATCAATAGGTTGTATATGATGATAGTGTATTTTATTTTATTTATATCAATTTTAAATTCATTAACCGGCTTTCATAATGCATAATGATCAAGGTTTTTCTCTTTTCGTTTTTTGATAAATTTATATATTTTGGCTTACTTGGAGAGAATTATATAATTGAAACGTAGAAAAATGTGCGTGTGGAGAATGTtaataagtgAAAAAatactcggtacctgacactggcttatttgtcaaggatggctccctaacaaacgaactaattgtggctttatatgtannnnnnnnnnnnnnnnnnnnnnnnnnnnnnnnnnnnnnnnnnnnnnnnnnnnnnnnnnnnnnnnnnnnnnnnnnnnnnnnnnnnnnnnnnnnnnnnnnnnnnnnnnnnnnnnnnnnnnnnnnnnnnnnnnnnnnnnnnnnnnnnnnnnnnnnnnnnnnnnnnnnnnnnnnnnnnnnnnnnnnnnnnnnnnnnnnnnnnnnnnnnnnntacattatacacattagtagctgtttttatgtcccaaacaaaaataaatgagGAAAAAAAAAAAACACAT

>Cs1g14780

nnnnnnnnnnnnnnnnnnnnnnnnnnnnnnnnnnnnnnnnnnnnnnnnnnnnnnnnnnnnnnnnnnnnnnnnnnnnnnnnnnnnnnnnnnnnnnnnnnnnnnnnnnnnnnnnnnnnnnnnnnnnnnnnnnnnnnnnnnnnnnnnnnnnnnnnnnnnnnnnnnnnnaaccGAACAACAATTGAAGGATACGAGACAAGCAAAATCCCCTAAAAAATACTAAATTTTGAAGCGAATCCTCGACAATTGAAACCCTAGAGAGGTTAAATTGAGATtttgaaggattgggatctaggtttagcgattcttgagaaaccctagattgtcagggaacgaggagaattttttataatttccccttttaaataaagactaaggatatgtcgttatcagacatcagttgaatggaggacagtatttatatgagtagagtgaaggtaattatcctgacttggtgagggtttctttggaaatatgaagaaagttcggggattggtcagagagtgacacgtggaatgggaacattgtttagcttctggcgtaagataataagatatgatacggtcactaatcactatgaggtagtaCATTTGCTTGGCCACAGCCAGTCAGCCACAGGCTTATTAACGCTTATTATATTTGTTTTGTAGTTGTGTTTGCttcaaattgaatcttgaatcttgaatcttgaagttgaatctaaggcgtgaagaaaaatggaccaatgagattatggcatagaagcatctaaaaaaatattataatgtgtttacttctcgaattggaaatcgaaattattaacatcattttacttcataaataaatgttcagttatggtgcaactgtggtaccataccacaattactttcaatcattatatcaagtcatgatgataactgtagctggatccaatggtactacagttgcaccataattttgtctcacaaataaatataagaattagaatccaaataataagacccacatcaaattagaaatagaan

>Cs1g05450

TGGACTTKAAACATTCTTAACAAGAAAATRAACTCTACCCTTGCTCATYTCATAGCTGTAAAGAGAATAATCAATACAAGGAAAAAAAAAAAAGTWACATCTTTAGTCATCAGCAAGAACTAACCCGTGATTTCTGATTGTGTTATTCGATCCTCCGTTGGCAGTAAAGTGTGCTTCCTTGAGCTTCACATTACTTGAGGAGAGAAAAAGAAAATTAGAAGAACTATCTTTTTGTTGCATTCATTTAAGCTTTACGCATTGCATAGAGTTTAAAATCAGATGCCATATCACGTTAAAGTTAAaaaaaagaaaaaaaAAAAAAGAGAAAATGATAACACACTGATCTTTTCCACTATTCAATAACATTTTCCGACTAATCTTAAAGATCATCAATCTCCTTTTACTTTTTCTCCTTCtccccccccccccccccnnnnnnnnnnnnnnnnnnnnnnnnnncaTATTTTTAGACCGATAATTTTCGTCCATTTatctaatttcccaaCAAAAAGAGTGACAAAAATACACCGAAAACAATGTCATtttgagtgtgtgttttaaaattaaATATAAAAAAAAAAAACACCTCCAAACAATGTCGTTTTCGATGTAAtttaattttcacccttttgtcaaaGTTTTATTTAAATAGTAGTGCTTGAAATTGGTAAGTGAACCCTCCAAAATCACTCTCAATATTTTGGTTCACATAAGATACAGCCCAAAGTTGCCCCAAGCAGCTGGGAACTTAATTATATGTAAGTTTAATTGCTTTTGTTAATTTGCCTTCTGGTTTCTTCAGCAGATATTTCTTGCTTCACTtataatttttttttttttggccccCTCCTAGAGGTTCTCATATTTACCTTCTTTAAATAAAAGAACTCTAGGAAAATGCTAGCTTCTTGTTATATTTCCTCTTTTGTTAGTACATAATATATATTTGTATTCACAATTATTACTGAGAAATTTTTATGCTGTTTCTCATAAGAAAACAA

>Cs1g12290

cccacacatgccgtgtcgtgggccgtgccgtgcctaggaaaaatagcccattaagcttttttccttttttatttttttaagagcgcatgccaagttgtcttatttttgtcaatcttatgtacttttttttttgaatttatgtgcctttatttaaatttaatagaacaaaaaatttaaaacttaagtccatattcaataataataaattaataataataagagaatgtaatattagttgctaagtttgattgtatggtattagaattctttttttgtacttaacaacaacaacaataataattttttttaagggttaacttaattgttaacttagaattacgtagagtcatatatnnnnnnnnnnnnnnnnnnnnnnnnnnnnnnnnnnnnnnntaatagttataattttatttattaaaatcatgatatcaattatttatttaataaattataaacataaatcaattataaattttttgaaactaagaattaaataaatttaaaaaacttgagccaataaaatatattaaaagattcaatttcaagttatttataaaatcataaaattttaagtttactttcattaaaaaataaaacgtgcttattgcgtgctttttcgcgtgccgtgctttggcccatctctaatcgtgccgtgcttttaaggtccatgtgcctaaaatcttagttcggcacagcccacatgcgtgccgtgccgtgccgnnnnnnnnnnnnnnnnnnnnnnnnnnnnnnnnnnnnnnnnnnnnnnnnnnnnnnnnnnnnnnnnnnnnnnnnnnnnnnnnnnnnnnnnnnnnnnnnnnnnnnnnnnnnnnnnnnnnnnnnnnnnnnnnnntcaatttgtacaaTATTATTTATACGCTTGTGGCTTTAATTATTCTAATGAAAATGAACAAGCTACCATCTTCACCTACGAAAAAATAAAAATAAAGATTTTTCTTTTAGACCGTTGATGTTTAAAAAACAAACCCTAAAGATTTTTGTTC

>Cs1g21360

cagactaattcaggtctattcagatttcagacaaaaaaacaaacgccacctaagtTAATTAAGTCATTAAATAAAAAAACAAACGGCCCCTTAATTGATTGAAATTAAGTCAACTAAGTCCTCAACTTAAAAAAATAAACGCTGCTTAGTTGAAGTCGTGCCTCCAACAAACGGTAGACTTCGGCGGGTAAAGAAGTTGAAAATGATCATGCTTGGAACATAAAGAATCATTCGCTTTTCGTAATGATCATTAGCTTTTTGATTCCAAGCACTTAGAATTTTTTCTAGACTATACCTACCGGGAATGAGGACATTTTAAGTTACTTTatcagtctattttattTTATTTGTTTCAAGGGGTCATCTGAGACTTCTCTGACTAATTTCTATTCTTCGTAAGCATTCATGCAGGCTTTAGCTTATAATAACAAATGGATCTCCGCAACAAATTAATTTACTAATTTATGAAAAATCTAAAATTTTTAGGTCAATGGTTTCTTGTTCTGATGACTGATGGTCAAGAGGTGAATGATCTGTGTAAGGAaactgtttttttttttttgcccttTAACCCCTTGTTTAGTTATGTACAATATACATACCTCAACGAATCTATCATTACAAAAATGTAGAcagtctatcattCGAGGTTTTAGTTCATGCTATAATTGCATGCCTGTAATCCATCCAATATATTAATAATGTATCTTCTGAATGTAATATGTATATAATTACCTTATCTTATGACATTTTACCTAAAAGTAACTTAGTCAATGGAGCTAGTGAATCCAATGAAATTTAATTAAGGCTTTGGATACACGCCTTATCAACTTACTTGCCAATTATGGGCATTAGCAGACCTAACGAATTGAACCTGCCCTTAGTTTTCTTCTCTTTAATCATCAATTTTCCTTAAATTCATTTAAAAGCAAAACTATCTTCTTCTTCACGTTTCACTTGCTTCCCTTGCCGTTAGGGAATGTGAAGAGCATGAAATTAAA

>Cs1g05470

AATGCAATTCAATGCTTCACACACAAGCTTGGGTTGACTCTTGGCCATACAttagtgaagaaagtccaacaAagcGgTGGTGTtCAAAAACAAAACTTCATCAGTTGAcTCAATTCCCAATTGTGAATGCACAGAAAAATGTTGAGTATAAATTGACTTATGTGAAGtCTTGGtCTGTGAtTCATGCTCTAATTTATAAGtCAAAAAACGACttgcactgcggtgcctcgcaagtctgtgttgattttaaaattctcttaacaagaggTAAAATTATAAGAGATTAcccctcgagaaaatgactatcttaaaaatacttttaaaTTATTATAAATCTCATTTTATCTtTaTTTTTTAAAAgAaGAtaaaaaaaaaaaaatttcATTACCGCACcctcattttttctttatTTTTTAAATAAAAATAGAGTAAATTAAAATTTTACAATAATTTAAAATATTTTGAAGATTACCATTTCAAAAAGGGGGTAATtcgacctttttttttttttttTTTCCTTTACAAGAgtccattcyatgcCCGTTTAGAAAAGAATTTCAATGTCGGAGtgtccaacgttgtTAAAAAAATAAGCTCAATACACGATTCTAACATTTTAAATTCCCATGAGCTCTAGTTCTTAAGGGGATTTTTGCTGTCGAAAACCATACTATTAAGAATTGGCCATATATAGAAACCACATAATCAACATTTGTAGTCGTTGACTAATTAACAATAACAAATAAACAACTCAACAGATTTTATTATCTCAATTTTTtttttgttatttATTGTAATAAATTTTATCGCTGAACTCTGATTAATTTTTTATCAAGTGTATGtgtgatttatatttTTTTTAATTGAGTTGTAAATTGCTAATTTCAATCAAACGTCACATGAAAGGCATGCAATAGAAACTTTACGGAGGTTATTTTCGTCGTTTTactcgttttcccgaaACGGCTAGTACAAAAATCAGCTGAAAGGAATGCCTCAAC

>Cs1g10425

aaaagacatatttttttggtgcaaaaatttccctccaaaagtgaatattttgtgacaatatttcactatttatgacaaaataaatggtcacgaaatataattataaaagacaactattttataactaaaagagaagtgtaaaattggccaaacaatctgcaacacaagatATTGGTCTTCAAAACTATTTTGGCTTTGGCTAATTTTAAGaccatattttttgtcatATATAATTACTTATGATGACAAAACTAAGAAATAGTAGTTGAAAGTTTTGTCTTGATTAATTTAATATTTTATAACTAATTTTTTTCTTTTTGAGACACATTACTTGGTAGCTAAAAAATAGGATAAAATATTTTTTCTTTTAATAGTTGCaaaaagattttttttggggcaTAAAAAAGGGTCTAAAATTAGTTATTTTGGGACAACATATAATCTTTTATGACAAAATATATAGTGGCAAAATATAATTATTTTGACGAAACTATTTAGTAGCTAAAAAAGAAGTGTAAatTCTATCAAACTATTTGTGACATAAAATATTAATTTAAAAAACtATTATACTTTTAGTTaattttaagacccgttttttgttgttatgtataacaatcaatctaaacaaaattaagaaatagtagtcaaaagttttgtctcaattgataatatattttgtggccaaaatattctatctttgtgacactttacttggtctctaattaaacttttttgtgatGATCAATAGAATTACATAATAATTACTAAGTTTTTTAGTTAAATTATATATATACTATTAATtaagttgttggcaataatgactaaatcaatactaattcggaataccatcaacaaccataatactaatgaaacaaaaaattttaatatgtagtgaaaaaattttaacaactgtccctaggatttagaattattttgactaattattctaacaattttatttagggataaatttaataatataattgctccaattttaattg

>Cs1g11770

ATAAAGAAAAACAATTAAAgcttatgcatagTTATAGATGAGGAGGGGAGAAATCCATCCTAATTGTTATACAAATATTTACAATAAAAAATATATTCAATCTTCTTAGACCAATTTTATAATGATGTACTGTGAATGTTTGAGTTAGAAAATGTTGATATATACTTCTGAATATTTAAAAGAAAATTAATTTTTGTTAATCTTCTACATACATGAAGACAAAAATAATTTACTACTTGAATGATATAGTAAAAATATTTACTTATATATATGAAAACAAATGCTTGAAATTGGTCTAGAGTAAATCAAACTCTTGCATGATTATCACCATGCAAGAGGTTCCTTCGAGTAGGAATGGCAAAAGTTCGGGATCAAAAAAATTTGATGCCCGACcctaaataaatccgaatattttatagttagggtcaggttagcttcaacccgaatcgagccaacattgggtcgggtccaaccccactttaccaaaatagttttcatattattttaatgttaaattattttttaatgttttgttttatattcatgttaTTTTAATATTTTCTTCTATACATGCACCTTAATTAACAAGACAAAGGCAAAATATACAATATAATTAATCACAAAATAATTCACAAAATAACCAATTaAtaggaataattttttaaattcaattccataatcaaactttaaaaacacaatgtgattaaattaaaaaaacacgattcaATTCCACAACCATttCaacCCCACACAGTCACACACAAATCAATTCCAAAATCTAATATGAAAAACACACAATGCAATTAAATTAAAAACTTACAATGTAATTCaaagagggtcgggtcaaagtttgttcgggcctaaaaattttaactcgaccctaacccaagactcaaaaattcggatcaaacctctaaaaattagagtcgggtcaatcgagcaaaacacaaggtcggataaaattgtcaaGCCTACCTTGGAGTCCCTCTAAGGGTTTGATG

>Cs1g10030

ataaaaatttattTAAAAAGATATACAGARCCAACTTAAGGCTTTGTTTGGTATGATTTTTCAAATAGAGCTTTTATTAGTTTTACCAAAACAATTTAGTTATTTGGTTGTTAATATAAGATTttattaaaaaaattatgaAATTACTTTAATAGGYAAGTTTTTTTaaaaaaTTaTATTATTAAAAAAATAAATTAATATTGTAAAATAAGTAGATGACATTTTCAATATTTTAACATTTAAAAAGAATTTCAACATCCTCTCCCAAAAGCRTAAAACTTTAATTTTTACAAATAGAAGTAAAATAATTTATTTAAGCTTAAAAAACTCTATTAGAAAAATAACTAAataataacaaaaattatgataagagcttatgggattaaggattcgttgagaattgaggttgtgcatttgttatttggtaaacactagttgatgtaccttaannnnnnnnnnnnnnnnnnnnnnnnnnnnnnnnnnnnnnnnnnnnnnnnnnnnnnnnnnnnnnnnnnnnnnnnnnnnnnnnnnnnnnnnnnnnnnnnnnnnnnnnnnnnnnnnnnnnnnnnnnnnnnnnnnnnnnnnnnnnnnnnnnnnnnnnnnnnnnnnnnnnnnnnnnnnnnnnnnnnnnnnnnnnnnnnnnnnnnnnnnnnnnnnnnnnnnnnnnnnnnnnnnnnnnnnnnnnnnnnnnnnnnnnnnnnnnnnnnnnnnnnnnnnnnnnnnnnnnnnnnnnnnnnnnnnnnnnnnnnnnnnnnnnnnnnnnnnnnnnnnnnnnnnnnnnnnnnnnnnnnnnnnnnnnnnnnnnnnnnnnnnnnnnnnnnnnnnnnnnnnnnnnnnnnnnnnnnnnnnnnnnnnnnnnnnnnnnnnnnnnnnnnnnnnnnnnnnnnnnnnnnnnnnnnnnnnnnnnnnnnnnnnnnnnnnnnnntttAAATAAAATTTAAATAAATATTTATAACCATTAAATAAGTTKCACTAAATTTATTTAAATAAAAATCATTTTA

>Cs1g26180

gtatttgtcataaagaaattaattaaaatgtatatataaatggtgaCGTCGGTTGGATAAGTAAACATGGAAAAGGCTATAAATGGATCACTTGCATGAACAAAATCATATCTTAAATGAGGAGTCTTGATTGATTTGGATGTTTCACAAACCTAAAATCAATTACATGCCAAATAATATTTAATCATTGTACTTGAATTTTATATCGCCAGGGAGGGTTTCAAGTTCGGCGAGCTAAAAAaaaaaaaatggaattgaaatgatgctgccacttctttgcttcttccttttgattatattgtttgttggtaacttgttattctctaacacaaggaacagagagaggggcaaaaaaaaaaaaaaAAgttgtcaaccaacgaaagaatcataatatatatgcgtaattttttaatgtacgtatcataattctattggcttattaaacatatttccagagtacaaacttttttttttttttttgggggggggtatgttttaaagtggtgctatttagacgcctctgttaaatttataaatctnnnnnnnnnnnnnnnnnnnnnnnnnnnnnnnnnnnnnnnnnnnnnnnnnnnnnnnnnnnnnnnnnnnnnnnnnnnnnnnnnnnnnnnnnnnnnnnnnnnnnnnnnnnnnnnnnnnnnnnnnnnnnnnnnnnnnnnnnnnnnnnnnnnnnnnnnnnnnnnnnnnnaaaatatgaatttgtatttttatgtCAATCATCCAATATACATACGGTACACAAAGTTTAAACAATTATTAGAAAAATAGATTGTTCCTAAAGATTAAGCTAATTAATTGATAAGCACGAAATTTTTGMTGGCATGGTGGCCAATTTGTTTgtatgtatggtaGCATGTCACCTTCCTCATATATaatatgaagggggtggtagtaatttgcagttgcaagaaacagtggggtattaAAAGAATAATGAAGGGGTGGTAGTaaaaccattagaaatcttAGAATGAAAAttgggcttg

>Cs1g05900

GCTAAATTGTTTTTTGCATGATTGTGTGATTGAAGTGTTAATGTTGTTGTCATTACTTGAGGACAAGTAATGGTTTAAGTTTGGGAGTGTGATAACTCTATGAAATGAGAGTTATCATGACATTTTTACARTTAAATCAATATTACTTAGAGAGTAAATGATGTGTTTTTATTACATTTTAGTTASATTTAGCATAagcatccttttaGTTTATTTTTAATAAATTGAGTTGTTTTAGATCATTTTYTGCTTAGATTATGTGCATGATTGTAGGTGACCAAAAAGCTCAAGTTTTAGCGATGGAATGCTACTGCAACTGACCTACAGAGATAATATAATAACCTGGATACAAAGGAGTTAAAACAAGTCTGGAATAATGACGATGTTGTAACCGTTGCTGRAGTAATRCTAGAGTAATGAAaaaaaatagatttTGTGTYCAATAACTAAAAGATTGCAATTTGAAGTTTCTAAAAAATAGAGGATACGCGCCACACACTTTCGTTTACCATAATTGTAAGTTATAAAAAGAGTACGCATGAAAAGAAAAAGAGAGAGCTGTCACCCAAAGAGAATAATAGAGAAAAACAACAAGGAAATAAAAAATTAAGATAGAGATTTGAGAGAAATAACCCAACAAGTTTTTGGTGCTGTTGCCGGGGAATGATTGTTCATTGTGATTTGTGAAATTAATCTTGATCGATGCTAATTTTTAATTTTATTTTAATTTGTTTATTTTGTTGACAGAAGTGCTCTAATAACCTTTAATGTGGAAATATAAACCTGTTGAATTCCAGTTtaatctagaaatttaaagaactacaaagagaatgagaaaagagtaaagaaattcaaaacctgttatagcaatggatgatttgcaaggcacgaggaatttgaatcctcgaggagaaatATAaccagTAAATGCACAAGGAAGTCAAGAAGGTCAGAATGGGCAGATTATTTATGGGCAAATAGGGAATAACAATATTATTCAT

>Cs1g08500

ATTTCAGTTTGCGCTATTGTTTCACaacaagagctggcaaaacgggttatcgggtcgtgttcgtgtcgtgtcaacttcgtgtcgtgtcaaatttaggtcgACCCAAACCCGACCCATTTAATAATCATGTCAAAATATTGAGACTCAAACCCGACACAGAAAAATAATCGGGTTACCCAATAACCCGCTTAATATCTATATATTAAACAAAAGTTACATTaaatattaacaatttatcatacatatgtatnnnnnnnnnnnnnnnnnnnnnnnnnnnnnnnnnnnnnnnnnnataatcgggttattgatcgggtaaatgggtcaagaattttaaccctaacccgacacggaaaaaaattgtgttgacccgaacccgacccatttaataatcgtgttaaatttgacgacccatactcatttatttcnnnnnnnnnnnttgccagctctattCACAACAAGCGTGTTAGCGTGAGATGAGATATGTCATGTTTAATATAGCAAAAATAATAACTCTACAACGTTCAAGTTATAACAAATGTAAGATAACTAATAACTATTCAAAATAAATACTAaatcaagttaaataaatactataaacttgataatattttctataaacaaggaacttcttcctaaattaataattggttaatttatcagtatattaatatttactaaattaatagatttttttttatagtttcaatgttattaatatatcaaaattttactaaataattgacaatAGTAAATAAAATATGGCTCTATCATTccttgtTTTTTTTTCTTAAAATTAAAATCTATATTAAATCTCATCTCTTATTTTTTTTATATTGCATCTAAAAGTTCTAAAATTACAATAAAAAATTGTGAACCAATAATTTAAGTGATTCCTTGTGCATGAAAACTATTATGATTTAAATGTATGTTTATTAAATTTTACATAGACAATATACATTAATGCACTGTTACTAACAGATATTAATTTACCGAGGAAACAA

>Cs1g06550

TCAgtttttttaaatTATTATCTTGTTTTTGTTAAATTTCTTGTCTTTATTTATMTATGGCTAATTCATGTTTATAGAATCAAAATGACTAATAAATTTATTTATTTTTAAAAAAAATTTGAAACTCTTCYAAATTATCTAAATAATTTTAATAATTTTAATATTGAACTGAGGCACTATCACAAATTCAAACCAGTTATGAACTAATGTCCTACCTGGTCCTATACTGGCTCTGCAYTGCATGGAAGCTGTTATGATTTTATCAAAGTGAATARTATGACTAAATAYTATTGTAGTTGCTTTGATACGGAAGGTCTATTTTTCTATATAKTAAACGGACATAATCTATCGAGAGGCAAATYCCCTGTAAACAAGTTGGCCAGCCATGGTCctaaaatgcagagAGCAATTTTCCCATTAAtcattaattttgatcacaaaattaattctgtgtatgtcgtcggttgatcttttaagctcttttggaaacatacatacaaatctttgtggaaaaagagaaaattaagatttTTTtcttaaaagccgatgatacacaaaaagagagaaaataagaagaggatacaaaatctgtacatgtgatgaattgttagacattaaaaatataaAATTGTTATCTMTAAAATASAATTTATAAGGAGTAATATTCTAGGATATAACGCTSTAAATTAATAAGGCCTCAATTGACGGATGATTAACCAACAAATAAATMAACCTATTATCTTTCCCTYGCAGCAAGTCCACTTTAYAggtgttgaagacGCATTGACTTGTTGACGTCGCGTTGATTCRAAATTTCTNACTCGTTTGCTCTTGTGTGTAGTTACTATAAATTTGGCTTTCCGTCTGTTTCTTTGTACATCATTCTTAGATGTTTATTTCtaccttgtccagctaatctttaataattcttgttgttctcatctttacttgttctgtgctgtttctacgactatcaagtctcagctgatttcaattacgga

>Cs1g19290

TTTTGTCTCTTGKTTTTTRGAAATTTCAAAATATTKAAAAATATTAAAGGATAATGAGAAGAGGGAAATTTAAAATTCATGTTCTAYYTTTTTTTTCAACATATATCGCASCACGTATCTAACTATAATTATTAATTAGTTGGTGTTTGCACATTAGATTATACATGAAACCTGAGATGCATTGTAYAATTTCTCCATTTGGGGATATGTGGTTTGGTSYTCYACCGCAAGTAACTTCTAGAATATCACAATTTTCTTCTCCTCCAACKAACTTCATAGATGATCAAAAACAAAATATTAGAAGRCAAAACATTATCAAAGATGTAAAATTATTTATGCTCTCCattttctttttttttttttcaaatTTGGACTACWAGAAAATTAAATTACAWTACATATATATATAACTATGTATATAAGAATGTACAGCTGAAGAGCTACTTAATTATAWAATAGAAATAGTACTTTAATCAGCTATTTAATTGAGTCAGTTAATGGAGTTTGACGCTCTGACTATGCTAGCAAGATGAAATGTTGTATACATGTGTTATGGATGTGTTCACACTAACACTAAGGACTCTAtgagataatttatnnnnnnnnnnnnnnnnnnnnnnnnnnnnnnnnnnnnnnnnnnnnnnnnnnnnnnnnnnnnnnnnnnnnnnnnnnnnnnnnnnnnnnnnnnnnnnnnnnnnnnnnnnnnnnnnnnnnnnnnnnnnnnnnnnnnnnngaaaaatttaaaaaaataaattcaaagaaatgtgaaattgagtatttaacttttagaatatgagaatatagtgagagaatacaattacaaagtagaaaaaattaataaaggataatttcagatttttttaatttTTAATATTTAATAATAaatttatttttaaaaatAAATATAGAAAAAATTTTAATAAGCTCAAATACCACCGCCAGAGGAAATGATGTATAAGTCTGCAGACAACATTAAATGCAGCCAAACGTTGACCaactac

>Cs1g03680

tgggaccaactgacatcaatgctaatggagctctgcccagattttaaccctcacaaatattcgagggatgtctactcctcactcatgggtaagggagaagactaccttcactcaaattaattgaggaaggaaatacccccacaatgcttttgtgggggttcgaactgctgccctccaagttggagggcagcagccctggccactcgggctgagccgagtggttaatgctaggagataactnnnnnnnnnnnnnnnnnnnnnnnnnnnnnnnnnnnnnnnnnnnnnnnnnnnnnnnnnnnnnnnnnnnngttatcaacagcctttcctacaaaaagaagaaacttgaagtgggagtggttgaagagtaataactgaaaggaggttgaaattgtttgcggaaaaatgacagataaaggctattttaggaattgaggaggaaataaacagaaaaggggtttgaaaatgatatcggaggggtgccgatagcctcactcnnnnnnnnnnnnnnnnnnnnnnnnnnngtacaacttcgagtgcaaataacatttttcaaataaattaagatacaaatattagcattggaaagttttattcttatttaagctttatttgccactgcttttatttttgtttattttaaacagtaatttagtcaagtgaatgtgagagtaaggttnnnnnnnnnnnnnnnnnnnnnnnnnnnnnnnnnnnnnnnnnnnnnnnnnnnnnnnnnnnnnnnnnnnnnnnnnnnnnnnnnnnnnnnnnnnnnnnnnnnnnnnnnnnnnnnnnnnnnnnnnnnnnnnnnnnnnnnnnnnnnnnnnnnnnnnnnnnnnnnnnnnnnnnnnnnnnnnnnnnnnnnnnnnnnnnnnnnnnnnnnnnnnnnnnnnnnnnnnnnnnnnnnnnnnnnnnnnnnnnnnnnnnnnnnnnnnnnnnnnnnnnnnnnnnnnnnnnnnnnnnnnnnnnnnnnnnnnnnnnnnnnnnnnnnnnnnnnnnnnnnnnnn

>Cs1g19830

CCTAAACAGTAAAAAATAGAATTAAAGCAACYTTTATTAAATTTTGAAACATTTTTTATAACCAAACGGATAAAGATTATCTCTTGATATGAGCTCTTAATTTTTAATGATCTTGTGTCATGTGTATTTAAGTGAtagtgtatggataaaatttaactttttcattttctttatttattaaccaccaatcacccatgtgtatttttactcagaagaagatcagatcagaTCACGAGAGAATCCTAATCCATAACAATAATTTGGTGTTCTTTTGGTAGATATCAACTTGAATTAACAAGTGAGAATCAAGATTSGACAAAATAAATAATTGAACAAACAATTATGACCAAAGAGACTGGAGCAGAATTCTTAACTCATTATATTTATTTTAAAACAAATTTTTATTTTTCGGGTTTCCCCACATTTTCCAACCTACTATAACTCTAGCTGGAGTCAATTAAAAAACAATAATRATAATCAGATTAGGAACAAATTCACATTTATTAACAAGCCCCGAAAAAATTGCTTTTATTTTTTGAAGTACTTAAAAATTATAGCTAAGTTGAAATTTTACGAAGCACTAAACTAATAATTACTAAATGACAaacataaaaaataaaatAAAACAATTGTGACGGAGATTTAGAAGAcaaaaggggggggttgggtTGGCAATGAATTGAAGAATTCTATATAAGTGGCGCCAGAAGTAGATAGGTAGGTGAATGAATAATCAGAAGTGTAACGACGTAAAGGTGGAccaaacgacgggcgactgagctGATATCATAATCGCCACTCATTTGCCAAAATaggggcatctggctatTCCCATGTGCCTATTATAacaTaaccagatcataactCCCAAAATAGcccccaccccatgtgacttgtcCCTCTCATCTCATATCGACGACGACGCATTTTCCAAAAGACCACTCTTACCAAAATACCCTTCAACGAACTTTACCTCTCCACCGAACACAAACTCTCGGTGC

>Cs1g09510

GGATTTAACTCATATATACTCAAGAGTTAACTCAATCGTTGAAAGTTACCTCAAGCTTATATTATTAGTCCAATGTTATTCTCAACTGATGAGAGATCTATATTTTCCAATGTCATAGCTGAATTTTATTTTTGCTTGATGTTATTTAGATACATAGCTTTAAGTTGGAAAATGCTGACTCGCCCAGTAATTTGCACTGAAAGAACATAGAAGGTGCTATGCATCCTAGCCATAGATGGCATGCTTATGATGTTATAAGAGAATTAGTACAATATGTTTATGAATTTATATGCTCCATCTCATTCCTAAGATAAGATCAAATCCCCTATCCTTKGCTCTGTTATAATCATTACAATTACTAAATTAAATCGCTTATAATCATTACAGTCTACTTCTTTCTTCTACCATCGTCCAATATTTTTCACTTATTGACATTCTAATTAATTGCTTATGGTTGGAGATAACTACGAACATAAAACATTTTATGTAAATAAAGATGCTTTAACGTGCTAGTGGAGAGAGTAAGTTTGACATTGTTCTATCTTAAATATCAAAGTAATAATTATATATCAACATGTATTCTCCAAAAATTATTTTCAAATATAAATATTCTTAATTTTTCTATTACATACGGATTTATTTTTTAAACCCAATTAAAATAATTTGATATTCAGTTATCATAGTTATATATAAGTTATATTTGGGAAGGGAGAATACACTYAAGTTTTTAATGTTTACTAGTTTTACCATTTAGTCCCTAATCTATTTTTATTATCCATTTCATCCCTAATTTACTAACTTTTTTATTGGTGCAAATCTAATGTAATCTAATGTGATGTATTAATCAAAAGGTATGATAATTTTCAACCGTACACTCMGAACATTAAAATTAAAACATCAAGAAATAAAACATCAAGGAAGTAATGGATAACTCAAATTTGTTGATGATGTAAGTGATAAAAGCGAAAAACAGTAGGGGCCAAATGTCATTTTGTTTCATA

>Cs1g25570

ATGGGTTATTGATACTGTAATAGCAGCATTCGTAGATTCAAGAACCACTAGATTGCTGATAAATAAAATCCCATCCCAGACATTTGTGGAAGGGAATTGCTTCCGGCAGATTGCCTCTGGACTTTTTAATATGCCCGTAGTGTTCGGGGCTGCAGTAATGCATGCCACCAGCAACTGATCAGTTTTCTAAGTAATTGAAATTAGAAGTATATACCAATCGAATCCTTTGGCTTATTGTTGATATCAATGATTGGTATGATATTTCTAGGCGCAGTcaacagaagaccaattTgggatgaatgccctatttatcagacTAACATTATAGTTTGtcttcctgatataaacctttttttgagtttatgcccattatttcgtcccttacggctctactagacagtagacataagatagaggattggccatagcagccaaaataatcccggggttcactcagttttggaggctgtaacccctgtagctcataggtGCCAACATTTTCTTCATTATCATCTGCTCCTGTGGATTTTGACATCTAAGATTGTTTATTCTTTTCTCTTACAACTTGGATCGCCCACTTGATCTAACATACATATAATGTCCGGTTATSTAGTTAACCTTGGTATTTAAAAAGTAACGTCTGTAACATAACTTGTAACCCGTCCTTARGGATTTGGTTTATGTCGAGATARTTTCAACWWCTTCATGGGGTCCCCGGACGACCCAARTTGGTTTTTTCTTCTTCTTTTTAAATGTGTCTCTAGATATATTATATTCAGATAACTTCCTTGATCCTAGTTTCRGTAGTACAAAGTGAKTGGTATTTTGAAACCATGGTCCGAGCAAARTAttacttaaactttaGAAGCAKCACAATTCATAATCCACYCAATACAAACCTTATAAATAGCTTACAGTTTCTGCACTGACCAAAAARTCAAAGCAAGCAATTCGAATTCTCTGTTGTTCTCTTCCTATCTCCTCCTTYGTTACTTCATAGT

>Cs1g09120

gaaatgatcaaaagttttctgatgcacaggatcctTAGAaaaggcaagtgcaggtacagcannnnnnnnnnnnnnnnnnnnnnnnnnnnnnnnnnnnnnnnnnnnnnnnnnnnnnnnnnnnnnnnnnnnnnnnnnnnnnnnnnnnnnnnnnnnnnnnnnnnnnnnnnnnnnnnnnnnnnnnnnnnnnnnnnnnnnnnnnnnnnnnnnatcaaaaacctttaaaacatttttctcaggttataTaacccaaaacagactttcctttagtctaaaacagggtaccaaaccagataagaaatgatcaaaagttttctgatgcacaggatccttagaaaaggcaagtgcagagcatacttccttaggttttcaaaatgaacaaattcaattctttattctttttctttcttaggacttcatttgcagagagagagaagagttttagnnnnnnnnnnnnnnnnnnnnttttataattaaaaattataattctttaaataaggataaaattgtaattagaaactatttactcccaatctaagataaagtaaacacataaattggattctgatctccattccatgcttccattccagtgtaagtaaacaacctattcccactccacactcccaatcttaggattcccactctcaaagattcccaatccaattcaaaaagtaaacgctaccttattttcatgttgaagcaatcggtgatggaaaaaagttgcaagttgaacactgacagtgcccttagtgccctacctctgtttttccatttggaaacaagccttaaagtggtgctcatccgggttcaaaatccgggtttcagaaacccgaatttttcggattgaacttggacaaacctnnncacggacacggacccggattttgcggataaggaattttgagggttgtactacaaaaaaagttgcacgACATGGGGCTTGTGGAAATTGGGCTTCAATTCTGACCCAACAAAGGCCTAATTTTTTAGAATGGGTAACC

>Cs1g26600

TTTTTCGAGTCTMTCGGCGTCTAATTTCTTTATCATTTCAATAGACGAATAGAGGGTTTAGGTCTTTTAATAGACTTTGAGATTTTATTTCGTTGCTTTTGTGTTGGAGTTTTAATTATGAACTTCTATAATATACTTTAATTGTAAATTTCAATTGGAATCGTACATACTTtttctttttcctTTTTTTTAAACCCATCATTTTTTTTCTTCTTCTATAAATACACGATTGATTCTAAAAATTTCACAaattgaaattcacaTATTTTCTTTCATTCAATAAATTGTATTTAATTTTTTTTTGAAAAGAAATTCATTAAAACAGAAAAACGATTACAAATAATATTTAATTTTTAATCATTTGATTATATATAATATTGTTACATGGTACTCATAAAATTATGATTACACATTTATATTTTGATGGAATGAATTAAAAACGTGCATTgaaataaataaaaaAAAAAAGATTCTTTTATGTGATTCGCTTCCAAATAAAATGCCATAAMAACAAATAAAATGCCATAAAAAAATAAacTACCATATTAAGAGCAAAATGATAGATTAAATAAAATATAGATGAAATAGCCATTCAAAATTGCATCTCAAATTTCATCATAGATAATATTATTAATTTTAATTTACTTAAAACATGTGCAAAATTATAAATCATTTTATAAATAACACATCACATATCWTATCATTTAAAATAATATTACGTAATAAAATTTGAAATGTCTTATTATATTAGAGATCCGGAGGTGCTCTAGAAATAGTTTGAATGCCTATCTCATATATATTTTATTTAATTTATTATAAACCACTttataaaaagttaaaactacataaacatnnnnnnnnnnnnnnnnnnnnnnnnnnnnnnnnnnnnnnnnnnnnnnnnnnnnnnnnnnnnnnaaaaaaaaaaaaaaaaaaaagAATGACGGCGTGTTCGTAAAACGATGCGTCTCGTCGCTGAAATTCAAACGGTGTCGTTCCGGACA

>Cs1g22510

TTTTGCCAAGCGAATTTTTAAAGGATTATtcttctattttttttaattaAAAAGAGTTAAAYATTAATTCAAGCTTAAAAATGAAAAAAAGTAGTCTCACAATACAAAATCATATTtaannnnnnnnnnnnnnnnnnnnnnnnnnnnnnnnnnnnnnnnnnnnnnnnnnnnnnnnnnnnnnnnnnnnnnnnnnnnnnnnnnnnnnnnnnnnnnnnnnnnnnnnnnnnnnnnnnnnnnnnnnnnnnnnnnnnnnnnnnnnnnnnnnnnnnnnnnnnnnnnnnnnnnnnnnnnnnnnnnnnnnnnnnnnnnnnnnnnnnnnnnnnnnnnnnnnnnnnnnnnnnnnnnnnnnnnnnnnnnnnnnnnnnnnnnnnnnnnnnnnnnnnnnnnnnnnnnnnnnnnnnnnnnnnnnnnnnnnnnnnnnnnnnnnnnnnnnnnnnnnnnnnnnnnnnnnnnnnnnnnnnnnnnnnnnnnnnnnnnnnnnnnnnnnnnnnnnaaaaatttgtacGTTTTTTTCAAGACACGACAATGTCAAATAATGGGTTTTCTaTTTTTTTTTCTCTCTCTCTTTCTCTCTTCtctaatcatycACGTCAAAATTGAGTATTAAAATATTTGTTAACCCATCACATTATAAGTTGTTATTTTCCTTTCATTCACTCGAAATTCGAAAATYACTTTTTAAACCAAATCACGAACGACCCACCATCTCAATGCCATATGTATAACTTATATTTTGTTARTTAGGGAAAASCGCCCTCTCAAGTCTCAACAAATGTGTTCGCTTGCTTCCttAATCCTTATTAAATATCATTATGTCTTCCTTTTTTTAATAATATAATATTAGGCTTTTTTGTTGTCGGACACAAATCATTAAAAAaaaaaatacaatTTACGAGAGTTCTAAAACCAGAAAGAAAatnnnnnnnnnnnnnnnnnnnnnnnnnnnnnnntaataaggtaATTTTGTAAGTGYACAAAACCAAAAAACGACAAAAGAAAAA

>Cs1g21770

TCTTTGAAATATTTTTGTACAACGCACAAATTTATTTACCATATGAATGATTTAaaatgaaaaaaaaaagttataTCTTGTCAATTGTTCATAATCCATGTTTAGATATCTTTAGTTCCGTATTAAAAATAGTAATAATATTATTAACATACTAGATTCSTTAGAATGAATCATTTATTAATTGAGTGTTTTTATTTTccTTTTTTATCCGGacacactattaaattatagttaatttnnnnnnnnnnnnnnnnnnnnnnnnnnnnnnnnnnnnnnnntaattaattatattaaattatatgattaaatagtatgttcACACGAAAAAATATATAAATACTTATATGAAAATGATTATATaaagagaggggggtactttAATAAGTAATGGGGATATTTTAACGTAATATTACAGCGGAAATAGAAAATGTATAAATGAGAAAAGAAGGTAGACAAAAGAAAAGTGGCCAAGTTCGAACTGAAAAGCCATCACTAATCTAAGACCATRTGATGATACCCAGAATCCAGCCGAAGGTCCACTAAATATTGTAATTGACTGAGCCGATAATATCGGCAATCTATCATATTATCGCGAGAGAAGAAAAACAGGAGGCAGCAACTAAAGttgatttttgtgggnnnnnnnnnnnnnnnnnnnnnnnnnnnnnnnnnnnnnnnnnnnnnnnnnnnnnnnnnnnnnnnnnnnnnnnnnnnnnnnnnnnnnnnnnnnnnnnnnnnnncgatacaaacattgattaatttaaattaTGATACAAATTTAAAAATATCTTTTATAATTGTGGCCTATTTAAGACCACATCGAGATTAAAAAAAAAAAACAGGAGGCAGCCTTAGTAGCAAGTATCTTTTACCCTTTTAATTTTCAAAATGTTTAATTTAATAAAGAAGAAAAATGAGATGGATGGCATTATTATCAAAGtacgcgcgcggcgagaggaTGGACGTGGATGGGTCGTTTTCTGGTACACGGGGGCTGTTTC

>Cs1g01030

GGGCTACATCTTGAGTTTGTTGCCGAGAACGAGCTGCATTTTGAGTTTGCTGCAGAACCACAAGAGGTACTGACGGAAAGGAGAAGTGASAGCAAGGTTGTAACGTCCCTTGTGGAAATTGAGGATAATAAGATTGCTGGGGGGTTGGCAGACACGCAGGTGATTGATTTAAGTGACGGAGAAGAAAATGATGATCTGAGAGGTGATAATCAGACGCGTGAGACTGACTTACGGCGCCTGGCTTGGCATTATACAGATCCCCAAGGAGATATACAGGGCCCCTTCTCTATTACATCGCTAAAACGTTGGTGGGATGATGATTATTTTCCTTCAGATTTCAAGGTTTGGAAGAGTGATCAGGGCCAAGAGAATGCTGTATTATTATCTGATGTTCTTCAGGGGTCGTTTCCCAGTTAATTCTGGAAGATAAAGATTTTAGTAGTCAAAAAGAGTTGCAAGTTGTTCTGTAACATACAAATTGAAATCTGTGAATAAAATTTTGGAGACTTTTCATCACAGGGAATCAAAATTTGACAACTGAAATAGTTACTATTCTAATTTTGTGTTTGAAACTGGAGATGACGAATCCATTGAGTTTGGAATAATATTGACTTGGGAAAAATAAGATGGTAAAATTAAGAAAGAAAGAAAAATTAAGCTTTTAGTGATGAGATTTGCTCTCTTTTGGGAAGTGTAGCATtaactataccatTCAGAATGAGCAAGCAGTCCTTTTCTCCTAAGCGTGGGGTCTTGTTTGTTTTGTTTTATGTTTTTTTTTTTTCAAATATCATTCGTTAATTATCCAACAGCAATAATTACCCAAGAGAAATAAAGTGAGTTTTTAAAGAGTAAACCAAAAAAACTGGCAAGGCTATATTTTTAAAATAAGAaaannnnnnnnnnnataagagaataaTTAATGGAAATtaaaaaaaaatgaAAAAGGAAACTAATAGGAGCAAGAGAGAGGAAGGTAATTTGATTTGCTGGGAGCTGGG

>Cs1g26080

ATTGTCARAGTAATGGTTACTTTTATATGGGTCRAGTTGGATTRGGTTGGGTTAGTCTATCATGAGTGACTCGTCAAACAACTTGTCCAAGTCTAACCRTTCAAAATAATCATATTATTATTTTGAATATAACATACTACATGTGTTaataaatataaAAAAGGTGGTTAAATCTAAAATGTCCTATATGTTTTTTATTTTAATTTTTGATAGAAAATGTTCTATATGTTTGTGGAAATAATATTGAATAAACAAGAGATCTTATCTTATTCCTAAATGATGCGGGATTCAAGCTAAAGTTGTAATAAATTAACTCAGATTTAATTTAAGAAGCTTTGATGAAGTAAGATACATGCTTTCCTATTTTATTATTATTATTTTTTTAAACCCCCTAAAAGTGTTAAAATGAGTTTCTATAGAGGATTGAAAAATCCATTCCCTGGTCCGTTTCAAAGCAACACCTTCTCCAATCTCCGTCTTCTTTTGGGCTTTTGGCCGCTATCAAGGCAATTGCTAGAGGGTATTTTAGTCAGTCCACTTAAACAAGGTCGGTCTCGTGACCTAGGCGGCTTGTTTCGTCTATAAATATGCCTTTGGGGATAACCCTTTCAYTCTTTCGATCGCAGAGGATTCMGAGAGACCTCTATCGAATCAATATMCCTCGATCTCTCTGTGTCAAATAACAACGGTAACCAACCCAAAWAGTTTTTACGAaatagaaactgagaAAAGAGGAAAAAAACAACAACATAAGTTATTATTAAGTAAAAAATTTMTTTTTAAATAATTAAACTATACTTCCCAATTTTTCAGAAACCCTAGAATAGGGTTTTCTACATTCGATCGCCATGGATACGCGATTTCCATACTCCCCGGCGGAGGTGGCCAAGGTCCGCATGGTCCAGTTCGGCATTCTCAGTCCCGATGAAATTGTAATTTCCTCTCGAATATCTCTTTTGTAATTCCTAGTAGTTTGTTTGGTTGCTGAGGATAAAGGGGAA

>Cs1g02720

TTGACAAGATACCAAATGGAATACAAACGGCAGGGGAGGTCTttgaaattacggtcatactttctatctaagtgtcaatacgccaaatctcaggggagatccctaaaaattaccatttnnnnnnnnnatttttgtaacgttacattccaattccaaagatgttggagacaatacgagttggaatgatataagaagaatgctaagggtagagaaagatgagaaaaataaaaaaagaAAagagtagagagagagggtaaatttATCTCATCTTTCTCTATATTAAGTACAACCCATAATATAATTAGTATCCAGACACACTTTTCATACTTTGTCACCCACTCCTACATCACGCAACTATTGGATTTGAATTATTGACCTAGCTAACTACAAATGCCGTAGACTTGGACAAAACAAAACTGTTtctaagggcattatttGAGGTGTAAAATTTATATTCTACATCTAATATTAATAAATGGATCACACGTCAATTAAGACTCAATTGCTAAAATTCCTGGCATTTACTCTTTTAACTATTTGTAATTCGTTTTCTATTTACTGGTCATGGTGTATAATTATTATTATCACTGGGAGGGGCAAAATTTTCAGCTTCGATGGAGTTGGTTGGAGATATCTGCATTCRACAATGATCCTTTAGGAAGGTAACTTATTTATCATGGAAGAAGATTTTAGATCGTAMCAGAATAATTTTCCAATTACAGgcagcagagtgagtgatatatatTAATCCGAAAAAAAGACAGAgtgagaaagagaaagaaatcaacacaggatgtccactacaaacatgtgaagaaatcactcacaaattaaatagctaaaagtaaggcaacgcgtaacaggagaaacgctatcaataaaaagtgttaccatctatgtaaagatattaagtccagggtttgtaattccagaatttattactaactttatcatgcagttcgctaacccccctactctnnnnnnnnnnnnnnnnnnnnngt

>Cs1g14000

TTACAAACGATTAACTCTGAAGCGTARGAACAATGATTAGTGAGTCACTGATTAGAAATGGAAAAATCTTTAGTTTGGTCATTGTTATTTCTAGAGCTTATTTTATGGAAATAATTGAAGAATAGTGACGTCATTTAATATAGAATTTTGGATTCaaatgttatatgnnnnnnnnnatatAAtATCTTTAGTTTTTGTCATACTAAACATATAAAAAAAAAAAAAAAAAACTAGGGCTTTAGATGTTAGGGCAAACAATTACCCAACGATTTCTACTTTCTATTTGCAAAATAGTAAAAATTATAATAAAAATGTGTCCAATAGCTAGCATCTCAAATTAATGACGTGATAAAAtaaatgaaagcggaaaaaaaagatctTAAAATAGAATAGTTTGCTTGAGAAACCCAAGTCAAGCCAATTACACAAACACCCCTGCACGTAATGTCACATTGAACCAAATCTGCATCCATTTCCTTCAGCTGTTAAAATCAGTTCGTCAAATTATTTCCATTTTTATTATTATTACTTtagtgcannnnnnnnnnnnnnnnnnnnnnnnnnnnnnnnnnnnnnnnnnnnnnnnnnnnnnnnnnnnnnnnnnnnnnnnnnnnnttatttttattatggtatggtacaaaataTTACTTCTATATCTTGAAGAAATTTATTCTTAAATAAAATAAAATACTACACTCATATTAAAGAGAAATTATAATGATTCAAATTCATTTTTTTATTCGTTACGAGCAAGTGAGTCTTTTAACAttgggttatagaaccTAACAGCTTTTAGGGCACACATTGAATTCTTGAATTTATTTTGATAAAATTTAATTAATTAGACTATTTATTTATTGTTTTTAAAATTTTCCAACCAAAAAACAAataaataaataaaaagaaaaagaaAAAGAAAATAACCCCAGGAAATGCCAAGTAATTACAAAAACAAGAGAGAGAGGGGGCTCACTTTAGCAATTAGCTCTGAAGTCTCCAAA

>Cs1g14030

nnnnnnnnnnnnnnnnnnnnnnnnnnnnnnnnnnnnnnnnnnnnnnnnnnnnnnnnnnnnnnnnnnnnnnnnnnnnnnnnnnnnnnnnnnnnnnnnnnnnnnnnnnnnnnnnnnnnnnnnnnnnnnnnnnnnnnnnnnnnnnnnnnnnnnnnnnnnnnnnnnnnnnnnnnnnnnnnnnnnnnnnnnnnnnnnnnnnnnnnnnnnnnnnnnnnnnnnnnnnnnnnnnnnnnnnnnnnnnnnnnnnnnnnnnnnnnnnnnnnnnnnnnnnnnnnnnnnnnnnnnnnnnnnnnnnnnnnnnnnnnnnnnnnnnnnnnnnnnnnnnnnnnnnnnnnnnnnnnnnnnnnnnnnnnnnnnnnnnnnnnnnnnnnnnnnnnnnnnnnnnnnnnnnnnnnnnnnnnnnnnnnnnnnnnnnnnnnnnnnnnnnnnnnnnnnnnnnnnnnnnnnnnnnnnnnnnnnnnnnnnnnnnnnnnnnnnnnnnnnnnnnnnnnnnnnnnnnnnnnnnnnnnnnnnnnnnnnnnnnnnnnnnnnnnnnnnnnnnnnnnnnnnnnnnnnnnnnnnnnnnnnnnnnnnnnnnnnnnnnnnnnnnnnnnnnnnnnnnnnnnnnnnnnnnnnnnnnnnnnnnnnnnnnnnnnnnnnnnnnnnnnnnnnnnnnnnnnnnnnnnnnnnnnnnnnnnnnnnnnnnnnnnnnnnnnnnnnnnnnnnnnnnnnnnnnnnnnnnnnnnnnnnnnnnnnnnnnnnnnnnnnnnnnnnnnnnnnnnnnnnnnnnnnnnnnnnnnnnnnnnnnnnnnnnnnnnnnnnnnnnnnnnnnnnnnnnnnnnnnnnnnnnnnnnnnnnnnnnnnnnnnnnnnnnnnnnnnnnnnnnnnnnnnnnnnnnnnnnnnnnnnnnnnnnnnnnnnnnnnnnnnnnnnnnnnnnnnnnnnnnnnnnnnnnnnnnnnnnnnnnnnnnnnnnnnnnnnnnnnnnnnnnnnnnnnnnnnnnnnnnnnnnnnnnnnnnnnnnnna

>Cs1g01280

tgataatttgatgcattttgtcgtcgaagcgcacgtcatgtgtaacgatgaggtaaacattgtgttatttggtaagtgtattattacttaattttaaatagaaaatcttaaagaatattattataagttttttgttttctgagaaaaaaataactataaatttagtttcaaataactccaagttgtttttcagattatggaactatataaattaaatcaaatttctctctatcctatgaatttaaatcctaaaatctctctaaccattgagagatcttaggaattgtaaatcagctaataatccttccaattaaatactaagaccaactacataacaacatcacattaatactatgatatagaatttatgaattataaatctatgatacgagacaggcttaggactaatttagtaatgttgtagtttttgaaatagctattgttaattttgttgtataaataatcagttgtaaaagcaaattagttaaatatttggtttttaaaagtactaagaatttaaaaattaatcatatatatttaataaatattatcattaannnnnnnnnnnnnnnnnnnnnnnnnnnnnnnnnnnnnnnnnnnnnnnnnnnnnnnnnnnnnnnnnnnnnnnnnnnnnnnnnnnnnnnnnnnnnnnnnnnnnnnnnnnnnnnnnnnnnnnnnnnnnnnnnnnnnnnnnnnnnnnnnnnnnnnnnnnnnnnnnnnnnnnnnnnnnnnnnnnnnnnnnnnnnnnnnnnnnnnnnnnnnnnnnnnnnnnnnnnnnnnnnnnnnnnnnnnnnnnnnnnnnnnnnnnnnnnnnnnnnnattcataaagttaAATTTTTATAAAATATTAAACATTAAAATTAGTTTTAAATTTTTAAAAAATTACTTCATAACGAATGATAATGCTATAATGATTTAGTTGACGATATTGAGTACCATATAACAACGTCGAATTAATTATTAATCTATAAAAATTATCATTCATGTAAATCGACTCGATG

>Cs1g03410

AATACTCTTATTCCTACCCATTAATTCTCCCAACTTACCCTTCTCAAAACGAAAATCATAAAACACGCGAATCTTCCTACAAATATCATACCAAACCTCCACATTTCCCTCAACAGAACTCATCAGACCAAAATCAACAAAAACCGTTTTCAAATCATCAAACAATGTGGCAATCTCTGTACCCCATTCATCTCCATTACCAGCCAACACATGAGGAAAAGCCAAACAAATGCCGACCAAAATAACATTACTAAYCCCATAATAGTTTTTCAACCTAGATAGCTTAGCTGTTAAATCTTGTGAACTCTGACTAAAAATCGAAACTTCCTCTTTGTATAACTTACCCAACTTGTCCCAAGGAAACCCAAAACTGGAAAGCGCACAGGCAGCATTCAACAAGCAGGAATCCTCCGAAAGGAAAAACTTATTAGCAGGCAAAAAGCAACTAACTTCAGCATGATCAATACCAATGCTTTCAAAGAAAAACTCAAATTCGTTAATGGGATGGTACCTGAGGAATCTCTGGAAGGTACTAGAGAAACTTGGSGCCGAATAAGAGACGCCAAGATCGGCAATGAGCTTGGAGAGAGTGTTTAGAGAGTGCTTGCTAATGTGCTCCGCATAAGTGAAAGGTATTGACTTTGTGTAGTGGAGATACTCAGTCACTGCTAGTTGCGCTTCTTTGATCGCTCGTGGCCGGTACTTTGTAGGGATTTTGAGTTTTGCCGCTGAACTCAAGTGGCGGGAAAAGATTGTGCATAGAGATTTAGGGTTTTGCATTTTTGCTTCCTGGGTTTTAGTTAGGGTTTTCGGCTTTGTGATTTTTATTGCATTTggttttggggctttcGGTAAAACTCCTTAATTTTCTAACTTAAAAACAAAATAATAGGAATGGCTGGCGGTAATGGATAATAACACGCTAATCCTTTATTTCAGTGATATTATAAAATATGGCTAAAAATTTTACTTTGTTTTTTTGGCCTTTTTAATTGGGAGGA

>Cs1g20820

cggggaggnnnnnnnnnnnnnnnnnnnnnnnnnnnnnnnnnnnnnnnnnnnnnnnnnnnnnnnnnnnnnnnnnnnnnnnnnnnnnnnnnnnnnnnnnnnnnnnnnnnnnnnnncccattgccatccctacccccacctttgacttttgatttgcgcactcgcttagttttttctacccaagcaaatttctgtattccaacataaatgtaaataaatgagtattttgaccttttcactatttgattattcaaacacaaaagtatttgatggttttttttttattatccacttgaactaaactgaaataatgttaggaatataaaagcatttaaaattaaaaagaataagaaatatagttttaaattaagtattaaaatgtacttattttttaaaagcttttatttatttatttattttttgccaactgatttgtcgttatggaaaataaaaattacaaagaatagtatgagaattgtattgcgcaacataaacattagtaaggcataattttgcatttttgaaactgaaaaggcatctatgcataattacgatgccgtccttgagtcttgagagataaataatgttgtaaataatgacaaatgatgggccgctatcaattccaaagaaaaggcaaaatttagggtagtaagtaagtgaaggataaccactnnnnnnnnnnnnnnnnnnnnnnnnnnnnnnnnnnnnnnnnnnnnnnnnnnnnnnnnnnnnnnnnnnnnnnnnnnnnnnnnnnnnnnnnnnnnnnntcttagagcttgagtgaaggcagtctttctcttagaatgtgagttgggcgtctctcgaatatttgagagggttaaaaatctgaacgatgtcatatcaaaattgatataaatgagtctcaataatttatatggtgtaaatatcatgagtaatagttctctaaaaaaaaaagtannnnnnnnnnnnnnnnnnnagggaataaataactttttagaaaatgttaaataaatgcaattgaa

>Cs1g02295

CATTTARTTAGATTTCTTAAAACCCTAATttcgattttaaaaaaaAAAGAAGAAAATTTGTTGAATTTTTTTCTTYCTCTCTCTCGCTCTGAGGGAGAGAAATTTTCAACGTAAGTGACGGGTTTTTCTGGTGGCAAAAGAGAGCATCAGGATTTGTTTTTTWAAAAAAATYAATCAACAAAACAAACAAAAAGTTCCKACGAGATGGTGGAATTATTGAATTTCCATAACTGCCCATCTGTTTTTCAAATTTTGGCAATTAAATGAACTCTCACACTTACTGAGATTCAAACAMTCTTTTTAATGGGTTAAAAAATAATAGTCATCATTTTATAATTTATTACATTATatatactttttTTCGAATTATACTTTTTTCATCGACAATCACTMTCTACATCMTCAAAATCAAAATATTTATTtCTTTAATTTCAAATTGAGTTAGATTTTAAATTTTATACGTCAAAAAAAATTTACGCATGATATTTTCATTACAGATAAAAATTAAGAACTTTAATTACATTTAAATTATCATTTACGATACTACCCATTTNTATTTARATTTYTATTTACTAATTATGAATTTACACACAATAATTTCATCTAATAGCTAAGGAGCTATTAATTTTAATTCTAGTCTTATTGCTCTTGTAAAGAGATACAAATTTAAGAagagaaatcaaAATTTTTCATAACATAGAAATAAAATAGTAAATGACTCCAATCTTGACATTGACTTCTGCTTCCGCGCGTGGGAAAAATACAAGGTAGTTGAAGATTTGACGTTAACAATTAACAAGGCTGGGGGAAAAAATTAATAAATGAAAAAATAAAWCAAATYAAATTGTCTGAGCCCGGGTTCGAACCGGGGACCTCTAGTGTGTGAGACTAGCGTGATGACCAACTACACCACCCAGACGATGCCAAAGKTTTGGCAAGGTTTTARTTTTCACTTTGTTTTGTATTTTAAAGTTCCTAACccgttaaaagcaacaCAACCC

>Cs1g25190

CTTGGTTGTTCAAGTTGTGAGCAATTGAGCCGGTTGATTGATTGTCTTGTTCATCCACATTTCATTGGTTAAATTCATTTCTGCCTTGTAGTTGTATCAAGTAATATGTTGTTGCTTCTGTTAGAATGATGTACATTGTACTTTAATCTGATGTCTTCCTTTAAACTGAGGTATCTTCCGGATGGCGGTGCTCGATTGAATATCAGGAAATTGGAGACAAAGCAATATTGGCAATGAAAGAATTTTACTTTCACCATGGCTTTCAAGGATGAATATTGCAGCTGTTTTAATGAATTTTCAATTTTTCATTATTCATAATGTCATTGTAGTTATAATGTATCTCATGAGGGTTTAGCAAGTTCACGAACTATGCTTGCTTCAAAAAACACTCGTAAACTCAAACCGCTCTGTTGGTTTACTGGTTGGCTTGTCCTCAACACAAAGGAAAGTCTCTCCATGTTTTTGTGAGAGAGTAGAATATAttctagttsacgacttcacggtgggCCGCTCCATTGTTCATTCTTTATaacataatattttttttaaaaatatatnnnnnnnnnnnnnnnnnnnnnnnnnnnnnnnnnnnnnnnnnnnnnnnnnnnntcaatggacttgaaaaaaaagtgaacacagatattttatatttaatgtaatgcagtgtatattaaattattttttttgtttttaaatttgttctnnnnnnnnnnnnnnnnnnnnnnnnnnnnnnnnnnnnnnnnnnnnnnnnnatctaggtgaTAAATTTTATAGGGTATTATAAATTAGTTTACAATATTCTAAAATTTAATTTATGATTTTGTAAATACATCTTAAAATTTAATTCCAAAARAAAAAATTTTCCCTATACATGACTAATTAAAACTAGTAAATCAGGTTTTCAAATAATAAGATTGTATTACAYGACAAATTATGAGTAAAATAAATATATTAATTTGTCCATAAAACAGAAATGAAAAATCGGAAGATTCGAACGCCAA

>Cs1g14680

TATGAAGACTTAATTTTTAGTTCGTGATCACTGTGATGGGAAAGAATTAATCTTCGTCTGTACATATCTTTGCTTTTCCATTAAGAAAAAGTGAATTTAATTCATGAACTATTCACGTCAAACAATAAGCCAATTATAATGGACATTCAATTTAATTCATTTCTTCTAATCCTCCGTCCTTTACAAGATTGACTTAGGTATATTAAACGACTGAGCTTTGGGATTTGACAGAGAAAAATAAATGAATGAACCTTGACCGTACATTATTTGACTTGTCAGTTACAGACGTGGATTAGGTACTCACATGAGTGTGTAATTTGTAAATGGCAAGTCGATTATCTAATATTTTCCCGTGTATATTAACACATTTCTGGTTATTTTTATGGGTGATGGATTGGATATGCATGACAGCTCAATAATTACGGTTATGGTAAGAAAATAAAAAACGTGTTTAATATTCATCTATAATTAAATCATGAGGAGGAAAAAGAAAGAAATTAAAATATTAAAAAAAATGCACTCGATGTGTTcatctatttcattaACTCTTCCAAAAATAAAAATATATATACTTTCTTCCAATTGTTATAAAACACTTTATTTTTATttttatttttacttaatacCATATAKTACCCATGAAGTAATTTAAATATCAAGGACAAGAATATGAAATTAATTAAAAATAATAATTCTTTTTAATTATCATGAACCTCAGTTTTACTCAATCCCTAATTAATTTATTTAAACTTAAAATTCATGATCATTCCACCCTCAATGAAATATGTCTTAATGAAATAACGAAATTCAAGAACATGCCAAATCAGCTAGACCACTCGAGAAGACTAACAAGTAAACTGTTATGTACTATTCGGAGTATGTTATTAATTATGAAAGATTCGGATATTGATTTCTTGAAGAATTTGTCGTTCGACAGCTTCCTGATTTCTTGTAGAAATAAATGATATTTTCATAGCAATTAATTAATATCGATCTTATCC

>Cs1g09980

GCCTCTCAGAAATGAGTTTTCTGCATTTGTGCATTTCAAAAGATATTTGCCGTCAAGATTAMCCATTTTTCTTTGTAGTCAATGAAGGATTTAAGCATAAAGCATTTGRGTTCATATCTTTTTCAATCCTAATTCTTTGGCGTTAAGGAAAAAAGCACACTTCTGTATTGCTTATTTATTTATTTTTTRGATCCCCTGTACATTTTGTAAAGAGATTTTACTAGTTGAGTCTTTAATATATTATAAactctctagttttttagttttttttttttTGACAAGTGTGAAGTTRTTTATCAATATGTTTTTTTGTTAACACTTTAGCATCAAAGACGAAAAGTTATTTTAAAATTTRAAGAGGGACCTAAAAAGCAAATAAAAGACCTAACTGTCAATATCTTCAAACTTATGATTTACACGTGAAAATGTTACTTAMAAATTTTGTAATTCCTCCATTAGTTGTTAGGTATTTACTCTTATAAAMATAAAATTTTGGAACACATAGCAACACATATATATATGTCTCATCTCTCTCTCCTCGTGGGCGTTAGCTAAGAGCTAGTTTGTTCACAAGCTCCATTTTTCCCGTAAACAACTTTTGAATACGTCCACCCTTCAATCCATGTACCATCGTTCAACATCATCAAAAGCAACAACGTACAGGGCGCGGTCCYTCACATCATCAtcatcatcatcatcatcattcatcatcATGCATACTTTTTGATCTTTCCCTTTTGCCATTGTTACAAACTTGATTAAATTTCTGTCTTTGATCTTCAAACCAATACCCATTATCATCCCCGCCATGATTAGAATGGACCAAATGATCATAGCCCRACAGCAGACTCGGTGTAATCTTAACATTATTGTTATTATTATTTGATGATATTKTCTCTCTCTGGGTTTTTTTTTTttgtATTATTTGATGATATTKTCTCTCTCTGGGTTTTTTTTTTTTTTTTCTGGCCCCTTCAATAATATAAAATTCCAATATCCAA

>Cs1g25350

nnnnnnnnnnnnnnnnnnnnnnnnnnnnnnnnnnnnnnnnnnnnnnnnnnnnnnnnnnnnnnnnnnnnnnnnnnnnnnnnnnnnnnnnnnnnnnnnnnnnnnnnnnnnnnnnnnnnnnnnnnnnnnnnnnnnnnnnnnnnnnnnnnnnnnnnnnnnnnnnnnnnnnnnnnnnnnnnnnnnnnnnnnnnnnnnnnnnnnnnnnnnnnnnnnnnnnnnnnnnnnnnnnnnnnnnnnnnnnnnnnnnnnnnnnnnnnnnnnnnnnnnnnnnnnnnnnnnnnnnnnnnnnnnnnnnnnnnnnnnnnnnnnnnnnnnnnnnnnnnnnnnnnnnnnnnnnnnnnnnnnnnnnnnnnnnnnnnnnnnnnnnnnnnnnnnnnnnnnnnnnnnnnnnnnnnnnnnnnnnnnnnnnnnnnnnnnnnnnnnnnnnnnnnnnnnnnnnnnnnnnnnnnnnnnnnnnnnnnnnnnnnnnnnnnnnnnnnnnnnnnnnnnnnnnnnnnnnnnnnnnnnnnnnnnnnnnnnnnnnnnnnnnnnnnnnnnnnnnnnnnnnnnnnnnnnnnnnnnnnnnnnnnnnnnnnnnnnnnnnnnnnnnnnnnnnnnnnnnnnnnnnnnnnnnnnnnnnnnnnnnnnnnnnnnnnnnnnnnnnnnnnnnnnnnnnnnnnnnnnnnnnnnnnnnnnnnnnnnnnnnnnnnnnnnnnnnnnnnnnnnnnnnnnnnnnnnnnnnnnnnnnnnnnnnnnnnnnnnnnnnnnnnnnnnnnnnnnnnnnnnnnnnnnnnnnnnnnnnnnnnnnnnnnnnnnnnnnnnnnnnnnnnnnnnnnnnnnnnnnnnnnnnnnnnnnnnnnnnnnnnnnnnnnnnnnnnnnnnnnnnnnnnnnnnnnnnnnnnnnnnnnnnnnnnnnnnnnnnnnnnnnnnnnnnnnnnnnnnnnnnnnnnnnnnnnnnnnnnnnnnnnnnnnnnnnnnnnnnnnnnnnnnnnnnnnnnnnnnnnnnnnnnnnnnnnnnnnnnnnn

>Cs1g19510

TAGAGGAAAAGTTTCATTGTAATTTTAGGATTTCTCTTTAGCTGAATTTTTCTTTCTttcttgtttgattgATTAGTTCTTCTTGCTCTCACATAAATAGTAACAGAAAAACCAATGTGMTTTGAAGAGGAGGgttcattgagggttagTGTTGGTKTTAAGAGGGGTGTCAACCGAATTTttttttgtaagaGTTAATTTTCAAATATTTAAAATAARAAAACAGAAAAGGGGTGTTAAGAGATAGTGAAGGGTGGATGTAGCACTACTCTTTTTTTCGAGGGTCCGGGGCCTCCACTGATAAGACTTGGGCCAAGTCCAAGTACATATTCTTGTCTTAATTTTAAATTTTTTCTGATTTTTTTAATAATAATAATTAATCTTCAAGTTCGAAAAATTGTACTGATATACTTTCAGRTCTTTGCTTTTCTTTTTGGTTTCTTCAAAGCTTCATTCGTTTATTAttatatannnnnnnnnnntgcgtgtTGTTATTGTAGAGTCGTAGATTTCATTTTCTGCattgcattttttttttgttttGCTATTGAAAATGTTTGCTAGAAAACTGTATGATAKCttcttcaaaaaaaaaaattgaaTTTATTTTAAAATTATTTTTATCTCTTCGGTGGCCATTAAAGTGTattttttttacTCTACCAGCAACTCAATTCCCCAACGCACGTGTGAGCGAGATAAAACCACAGACATCTTGTTTTTGTATAAAAAATTTTTATTTCAATTATATAAGAAATTTTATTATTTCAGTTAGATGACGCTTGTTTTTATATACCAACTAGTTTTTACTGAGTCAAAAAGGAGAAGAAAAGTATGGCCAAATTAAAAAAAGGCAGAAACTGAAGAACTGTTGCTTTTTGATCAGAAACGTTCGTCACTTTTCTAAAAGAAAAGCATATTCCRCATGCTTTACGGTTACCTCTACGGCATCAACATCCWATAAGCTCCATCTTCCATTTGATAAATTAAagaggggaaga

>Cs1g09070

GAGCTGCAGTTGCAGGCTGAACACTACTAGTAGCAGAAGCAGAATTGGAGTCTTGAGTTTTGGTCTGCTTTGGCTTTTCTTTGTCAGCTTCAGCAGCAGCTTTTATGGGTCTAATGGATGAAAAGCGAAGAGGGTTTTTTCTTGAGGATTGAGTGAATGAAGTGAAGCAAGAGAATGAGCTCTGTGAGACTGTTGCAGACAACGCCATGGAAGAGAAGAAGAAGATGATGATGATGATGAAGAGAGCTCAGTTGAATGTAGAAGAAAGACTGGCTCTTTAGAGATAAGAGATGATTGTAYATTTTCTTTTTTTACTTTTTATTCTCAACGGCCAGGTTTCAAAGACAAAAATCSGTTGGTTCCCGGTAAATCCTTTTATTTTCTATTTTTATGTACTTAATATTTTTTCAACTTTCTTTTAGAAATTTTGGTTMATTTATTTTGTTCAATTTTTTGTTTCAAAAAACTTCATAAAATATATTCAGAATAGATACATAATATTTATCGAAATGTCACATTACtctaaccaaaaaAAAAAAAAAGCGATACCTTCCAAAGAAATTAAATTATTTtagcttactaccTtcTTTATTTTGAAATTTTAGTCAACTTTTATATTTTTTAAAGACGTATTTTGGTCAAATTAAtttttttataaaaatGACATTTTAATTTCTCTTAGAATTTRTTTTTATTCCATACTTATCAAACTTAAAACTAGTGAATTTTATATTAAAATATCATATCTACTCTYCGATTTTCAGTTTATATTTTGATATAAAAAATAAAATAAGGTAACTTTCAAACGGTAATGAGGTAATAAAATTTTAGAAACAAATTCAAATAGTTAAAATAATTAGTGTTATTATCCTAAAATTTATTCACACTTTGTATTAAAAAAATTTGTTTTAATCYCAGGTAATTATTTTAAGTCTATAAGAGTAATATATTAATATTATATAATAATTTAAAAaaATAGTAAAACGAAATACAACATACAA

>Cs1g21400

GAATTGCAGCCATTACCTCAAACAAGTATTATTTCRGCATTGAACTAGTCTATACATATAAAGTTTGGCAACTAAGACAAATGAATGCAAAATGATAACCTCATAGCCTGCACCCATTAGAATGACATCCAAATGGCTAAGTCTCGAATAGATGCTCTAGGAAGAGTATCTGAAAATTTATGTTTTTTGAATTGCCTTAGCAGAATGTAAAAAACTCTATCTACATYCATTATAAAATTGTGATAACTGAAAGCTTTCTAATAGAAAAAATAAGACCTTGCAACCTTAACTAAAATTATTGAAGACCCAAACCCGGCCTAGGCTTATTTAGGCCCGACTCGTTGAAGTTTGGACAAGCTTTAAGAATATTTGGGTTGATTACRAATTACACAGGCTTTGGCCTAGGCTTAGAAAAGCTCCGRCTAACCCCYCAATTTGCCAACCCTAATCGCGAATACATAATGTGTTGAGAGCTTTTTATTTttttcctttaaaaaACTAATAMTTATTATCATAGTACTACTAAGACAAACTCTATTTTTTTATAATGRTGTCTAAAATTATATTATACGATAAATATTTRTATCTTATCATATATTATTTTAAAATGATTTTATCTTATTCTATTTCTTAWAGGGAGGAAAAGAGTCTCACTAGGGGAAAGTGGGGGACTCAAAACACTTTCCTCGAATTCGCTCGTTTCCATGATGGAAATTTCTTTAACATAACATATTATTGACTAGACTAACCACAAAGAAAATTTTAATTTCTTGTGCATTTGCGGTAACATTTCAAAACAAAAAAAGAAATGAGTgagtacaaaaaaaaaaatgcarATGAAGTTAAAGGAATTATTATTATCATCATTATAATTGTTATTATTAAaaaaaaaGAGGTACTATAGATTTTTATaaaaaaaaaaaaaaaaaaaGAAACACGcgttcttttttgtgttTTtAATCTATCTTTTAAGAAAAAGGAAAAGAGGGGGAAAGTGAAGG

>Cs1g04930

ttattatttttaaactaattaatcttactaggaccatcatagtctttgtcttttgctacagagcatctaattccataaccatcttttcaactctaactattccagttttcttttttttttcttttcccctttgaatggatgtacatatctggcaatttaacaatccccacgacccatgacgctgcaagtccactaacttttgctgatcatttgaatttatattaattattaaaaaattaaactatattaaaataaaatgtagattaaaaattgacttgtacagtcaatctgataatggactagctccaaatggattttcattattaggcttgatttaggtgcttaattagcctacatttggcggtattttagttgatgacagaaatgcaatttttcgctggtcaaatgacccggattgggtatttagaattagcttcatgcaaaatattctgcaacttttccatcttggataaagaaatagccatgattgttgtgggtatgagtttctaatttgtgatttttcttaaaaagattaaaaaaataattttagttttcttaatgaagaatgaaggttgatttagttgaaaagaacattaaggtgagagtgaaagctgaaatttaattataatgaattataggcactctacatttctcgatttatttttcatagttatcatatattactcccccattcaacaacaaattaaatttatctccctcgacgtaatcctttttttttttaagtaaacattgtatatcatttaattttcgactttctattagtactaatctaacaatagaaacgatggatgaattgactttataacatacttcttttgactttgaatatttatcgctgcacaacgtgggatcaacggttcaaattttttttataaaaatttaaaaagccggccaccatttgtaactgtatatcctcccccctgctgtaccatatccaGTATCAATTCTCTTCATCTGCTTTCACCTCCCACAATTAATCA

>Cs1g01080

ggggtgttcgcggatcggattttacggattgaacatcaatccatatccgatccatgattttacggatgataatttttcaatccaatctaatctacggattgataaaattcaatccaaatccaattcatacatctgcggatcggatgcggatttgactcaattcatatccaatccatacatctgcaaatcggatgcagatttgactcaatccatatcaaattcaccattttgcgcattagttttcgaattaaaaatttttagttctctccaactaatgaaataaaaaaaatctaatataaaaataattttgctaccaattaaatttaaaattgaataaaatttaaataaattaattatttaaataaagctaaaataatacatttaaagtttcaaaatataacacatcaagactaattattcaaataaagctataataatgcgttcaaagtttcaaaatataacataccaagcttaattgttcaaataaagccacaatgaattgaaatggcgaagttttggaaaccaaacacagtctgatccttcaccagggctgttgcgcctgtcgaaaagtggtgattacggagccgtccacaatgggaaccaggagaagccatacgggtggcgatcttgaaggttgtgatagtgaaattgttcagctagaactagtgaatgtgaagtggctcgatgaacttgggaaagaagatggcttactctcgccggaatcgcgcggtcaatgtccttcaaatctgtgtcgagagtgtatccaaccagtattgagtgttgctagtgtgtgtgacggcgggaaagtttaggattttttgtttgtaaattgttggtgacttggcgttatgttacctataatnnnnnnnnnnnnnnnnnnnnnnnnnnnnnnnnnnnnnnnnnnnnnnnnnnnnnnnnnnnnnnnnnnnnnnnnnnnnnnnnnnnnnnnnnnnnnnnnnnnnnnnnnnnnnnnnnnnnnnnnnnnnnnnnnnnnnnnnnnn

>Cs1g10320

ggtggGATGATRGGAATTATTTTGTAAGGTACTGATGATTTTGTCTCTGAGATTCTTCTTTCACATTGGAATCATGCTTTGTCTCTCAKTTTGTAYTTWACTGGTTTTACAATSTTGTTATGATAAGGAAATCTAAATATAATTTAAAAAATGTAAAGATAAATATTATTATAACAAATTTTTATTTCTTGATGTAAATTTTSTAAGTMGTAAGSAGTATTTTAGTACTCACATTTTTAAGTATTTCAAGCTAGGGGAGTATYGTCTTTTTGAGTTAATAACAGCCTAAGTTAGAATTAATTAATTTTTGAACGGAAATGGGGGCACAGTGCACATGGTCGAAAGATATGGGRATGCGGTGTAAAATATTGAAACAAATGGGGGCTGACACTAAATTGCCCTAAATTTAATTAAAAAAATTATTGATCTGAAAAAACYAACAGAACTGATGGTTCCATTTTTAAAAGCAATCGGTTCAAATGAAAAAAAAAATCAAaaattgaaaaattanaaaaTACAAAGGAAATGAtaatttaaaacaaaaaaaaaaccaaaCGAATTTGGATTCGAAATATTATTTCTAAAaaaatcgatgaTTCAGTTCGATTCGGAACCGAAACGAATTGTTGCMCAKCCCTACTAATCATAACtatgtattttttcttaaTACAAGGTTGGATTATAGCATAAAATCCAAAATTAAGGAAATGGTGTTGTCATTGACGTGGTACTACCGTATCGATTYAGACTGAAATACAAAGACATCAAGCATTACTRTTTTTCATTTTTTGAAGTAATTGACAGCAACACTCTTTCATTTTCACATGTGATTGATGCCAAATCTTRACACACACACACACACTTCTAAGTTTCTTRGGCAATGGCAAGGAAGCGTAAATAGTGGCATCTCTTAGTAATGATTTTGAGCTTGTACTTCAAATGGGTTAATGGAGCAGAGCAAGTGCCACGTTACTTCATTTTTGGAGATTCATTATTTGACA

>Cs1g20980

GTCCTCAATTTGAAATAACTTCCAGCCCAGATTTCTACAGAGAAAAAGTGACGRGTCATTGTGAAACAACCCGAGTTGCTGGGTACAACCCATTGGAGCAGAGCTCTAGCATACATTGCTCGAACCCTATATCTCAAAATACTGAATCCTCAAACTGCCCGAACTTATCTTGCATTCCTTCATTTGCTACCAATGGGTCAATTTTTGTTTCCCTTAGCCCTGAAACCCAGGGCCACTCCCGATTTGTATAGCTGTCCTGYATTCTACTTCCTAGTGTCTTTCCTCCTCTGCCTTGCCATTTatttattttttccctttTTCATTTTTCTCCAACGTAAATATGTGTAATTTCGGYATACTtcctcttttaggtggAATCGGCGGGTATTTATTTATTATTATTATGATTATTAGTATGTCCAATGCAGAGGAAGTAGAGGGAGAGCGGGCGGGATTTTAGTATGTGACTAGTTACATAGAGACATTCTGACACGTCAAATTTCCGATGAAGTGTATTTGTGTTGCCGTGCCTGTATATATTTGCCAAGATTACAGATTAGTTTGGACGGTAAAATCAGTTGCAAGAAACTACCTACTTTGTTGCTGTTTACTGTCTTTTGCAGTTTTCCAAGTATCAAAGGTATTCACAYAATAGAACAGTGCGGATTGCaaaaaatttttaaattgAACCATTCCCGAATATTGGTTTGGTATGTATATGTATTTATGTATCTGTTGTTGCCGAACCAGTTGACATGTGTAAAACTCTCAAAATTGATCTTCAATTTAGTCGTAGGTTTGTGGTAAATTCAGTAACCGATGGTAGCTGACTGTGCTTAGAAAGTATTAAATTTAATTTTATTATTTCTTAGTGATCTGAAATAGTTAATCAATTGTAAAAATAAATGAATGGTATAATGATATGCAGAatatttaataattggtctWTTTTTAggatacatacaagGGTCCARTATAAACCCCCAGAACTTTATACTAGG

>Cs1g11710

TCTTCATTAATTCCCAAGATTATGATTATGTAAATCCTACAATTTATGCCATTTTGTATTTATTCAGTTTCTTAGTTTAGCTTTAGGTAAgcctattttatagtcTCATTCTTTGTGTAAATAATATGAAGAATATATAATAATAATATTTCATGTTCATTTTCAATTATATTTGGTTTAGATCTTTTAAATATCAATAATTATCACTACAATATTAAATGATATTAATACTTTGTAGGAAAAAAAGGAAAAAAAATTATGTTGGATtacacttcatttttttttTTTAACTTCAGTTAAATTTGTATAAGAACAATTATGGTTAATGTGAATATATAATAAGCAAAAACTATTTTACTACCCTTCACTTAATTGTGGAACCCTGAAAATTGTTTGCTAAAAATATTAAAAAGAAACAGTTGATGTCTCGTTAGTCAatagtcattaaTCATGAGAAcAAAAAGTGCATTTTCTACACTTCTTTGTAAttagtaaaaaaacaagtgCAATTGTTTTACAGACTGAAAATTCCACTAGTAAAAACGCCAATCAAATGCATTTTATCATTTTTATTTTTTATAGTCATTGAATGTTTTTTGCCCGCTCATACYCTCAAYCATCTCAATTTTTATGCCttagtaaaaccaactCTTCttttgttttttttccccttGTGTCTTTCCATTTTTGTTTTCGTCGGGCGAACTGTAATTTTCACATTTTGTTTGTAAGCAATAATTTAGGGGTMTCACATCGATCCAACCCCACCTTATTAAAATTATAAAGGAGAAAACTTACGTTTTCATTACATCAATTTTCTTTAGTCCGGAAACCAAATGCAAATGCATCATGCATGTGCGTGTTTCCACTATAATTTTCATTGAACGTCATTGTGGGGACCTAATCACAATTATATCACCGCTTATCCATATACTTTGATTTCCGTCgggttgagtggtTGGTATTCCTCAGCCATTGAAATTGCAAGGGGTGTGATAAAC

>Cs1g18270

GCATTCTGTTGAACAATATAATTATGCCTTAATTCGATGTTGGATTTTTGAATTACTGGGATTGATATAGTCACCCTTGCGTGTGTTGCAGATATGGAGGATGATCGATTTGATTTACCGGCCTGAGGAAGAGGTTTTGGCTGAGCTTGACAAGTTCAAATCTCATATTTTTGGTTGTGATAAAAGTTGTTAGAATACCcagctagtgtcaAGTTTCCAAACTCTTGTACAAATGTGTTTGTTGTGTacctctatcacaATTATCCRTCGGATTTGTTTTCCTCCTTTTGATCTTACGAGGTGCAAAACTTTTAGGAAACTACAGTGTTCTAAAAGAAAATGGCACTCTTGAGGAATTTTGCCAAAGCAATGAATACTATTATTTTATTATCCAGCTAGCTGACTGAGCTTAACTACGGAAACGGAAGGTAGTTAACAAGAAGTGTTGTGTCAAATTTTGTTTTAGTTGAAAAAAAAAAgactgaagtgttgtgtcaannnnnnttttaGTTAACAAGAAGTGTTGTGTCAAATTTTGTTTTAGTTGAAAAAAAAAAAAGGAAAATCATCTGTTTAAGACTTaTTTTTTTAGTGGAAAAAAggAAAATCATCTGTTTAAGACTATGTTTGGCAAACGTTATTTGTTGTATAACATGTATGTAACtcaATAAATATTATATTCGCATCATTTGTTGTATGTAAAACATATTTCTTCATTACAATTTGAACATTACATCGCAAATTTAATTAAAATGTTTTGAAGGTTgttgtggggttCGTTGGTAGTGTTAGCGTCAGACGCCCAAAATATTATTACCCGGTGAGACTTTTACCTTTGCGTGAAAAGACGGTTAAATCATATTCTAATGTRATGTCTATATTCAAATTAaggaagaaaaataataataatAATAATAATAACGAGGAGGAAGGAATTTGGAAATAGCAAAAATAGTAAGTCGTGGCAATAGCAAAACGAAAACCAATGAATGACCATGRGA

>Cs1g02250

AACTAAATTTCAGAATATTTCGGTATGGTTTGAAAATTTGATTTGATTTTGGTTCTATTCTTAAGAACCGAACCTTATGGTACCCACCCCTAGATCCTTCTATATGAATTATAAGATTTGCTTCAAGATTTCTAGCCATTATTCCTTTAATTACTAATAGTAATAATAATTTATACTAGAATAATTTTATATCAATTAAATAAAGACtatattttttcatctcatcgtcaggagaagaaaatgccaaccaactcaatggaggggttatcctccatcccacataaatattaaattttatttgaattaattatttttctcaatagtaatctatcatcatttgtttcaatggttaacaatagggcaaagtttgatggtcattcagtcaaagaatgagcaaaagttatccttcataattcacatttctaacctaacagaagaaaaaaaaaatttgcaagactttggttcctgagtcatgaatttggattggaaaatgaaaaataaaatgacatggtcatcgaaattttatgttagccatggattaaagtagtaaaccgtcattttctaagactttagaagcatcccttgatttcacaaagcaacaaactgattagaaaagtaaAAAGtgTttgAATTTTGTTATATCTTAGACGAGTCtcTCCCTAGGTatggGaattatttGTAAATGTAAGAATTCAGAGACTCAAATTAATGAAATTGGGAAAAGTAAAATATATTTTGTTTGatttataagcttgtccttaatagtacaattactttataagtattttagttatataaaattttattaaaataaaagtgtttaaagcattttaagggtatattgccaattttgtaattatcttataatcattgactagacgaaacttttactgcagaccttacgcgatatgctcttaaattatatctatatatcagacatgatttgaagtaacctcttcattcagatcagagtctcagagccagcaaaaagcaattagaaa

>Cs1g14880

AAATTGTGAGTGGCAAGAAGAACAACAAATCCTATCATACTGAACGCCCACTCAACCTTGTAGGAATTGTAAGTATGATCAGTCAAATTGTGTATGTGAATTTCATTTGAACTTGCATacttgacttcgactgctcttattcggtattgcaggcatggcaactgtggaatgaaggtaaagatttagagctaatagaccttactctggatggatcatgccctcctaatgaagatgcatttttgttggtctcttgtgtgtgcaagagcaagcagcagatagacgtacaatgtccaatgttgtttccatgcttactaatgaatctcttgcccggcctatacctaaaaaatctgcattttttattgatgttaccaccgaagaaccagaagctaccgaaaacaagtcagaagtttgttacgtaaataaagaatcaattacnnnnnnnnnnnnnnnnnnnnnnnnnnnnnnnnnnnnnnnnnnnnnnnnnnnnnnnnnnnnnnnnnnnnnnnnnnnnnnnnnnnnnnnnnnnnnnnnnnnnnnnnnnnnnnnnnnnnnnnnnnnnnnnnnnnnnnnnnnnnnnnnnnnnnnnnnnnnnnnnnnnnnnnnnnnnnnnnnnnnnnnnnnnnnnnnnnnnnnnnnnnnnnnnnnnnnnnnnnnnnnnnnnnnnnnnnnnnnnnnnnnnnntataaacgtagaggggcaagagtatttgttcaaatttaggccattgctatttaaatttttgctatgcgttactgtccttatccaatattgaattggaaacaaagtcataaccaatgtcattaacaaatctagtccgctccagtctggcatgcactgggagacttttgtgacataccacaattgaatgctattcannnnnnnnnnnnnnnnnnnnnnnnnnnnnnnnnnnnnnnnnnnnnnnnnnnnnnnnnnnnnnnnnnnnnnnnnnnnnnnnnnnnnnnnnnnnnnnncactgctactcACAAAAGCCAACAAAACAA

>Cs1g09660

atgttagaggggtaaaattgatatttcatttgaataattaaaattatgttgtattatgctaattttttttaaaaaagaataaaacgacgtcatttaacgtgatatgaaaattaaaaaaaaaattgatgttgtttaactcaattgaaaaagaaaaataaatacaaattttctaaatctaacaagtaagaaatactattttcctctaatttctggtacctacctttgcgcttaattctattgcttgaaaaaatttaaattcagttaatttatctttaatttttatttattttgagttaaattataattttaaaaggtaaaaatatcatttcattaacagtctattaaatcaactaacggagatcacacagaagtgaacgaatacaataaaaaataaattttaagtagtgtgctgaaaatttttctaaaataaaagttttcctgtacgtgactagcatttccggttctttttgggttcgtagttgatcatatgttgaccattagaatctcattatccaatccaagcaaattacttcttggaaaagggcacacctttgtgaggtagcggggaaggtcgtaactgtaaagcagagaaggaatcagaaaagaaaaatggtagtgccatcgtgtggggtctttctttctttctttcttctttttaaattgtattaatgatgctgataaacaactggcagttaaacaaattgtgccattgtacacgttattatacgcgacgagttatatgttatgattttattggtaggtgttctcaatttatcaataaaatatatcgtccagggtaacgtatttcacggtgcgtacttaattactcttaaaacgatataattttaacatctgttgtttattattattttttcaattaatctgtaaaatcatatcgtagcgagagaaaaaaagtcgggccgtgggggatagcatgacgtgtcaatgcttttgcctttataaataataataacataatatgatcgccctctcaccctttg

>Cs1g25330

tttacttactcgaaatatcttctaaaaatacaataggnnnnnnnnnnnnnnnnnnnnnnnnnnnnnnnnnnnnnnnnnnnnnnnnnnnnnnnnnnnnnnnnnnnnnnnnnnnnnnnnnnnnnnnnnnnnnnnnnnnnnnnnnnnnnnnnnnnnnnnnnnnnnnnnnnnnnnnnnnnnnnnnnnnnnnnnnnnnnnnnnnnnnnnnnnnnnnnnnnnnnnnnnnnnnnnnnnnnnnnnnnnnnnnnnnnnnnnnnnnnnnnnnnnnnnnnnnnnnnnnnnnnnnnnnnnnnnnnnnnnnnnnnnnnnnnnnnnnnnnnnnnnnnnnnnnnnnnnnnnnnnnnnnnnnnnnnnnnnnnnnnnnnnnnnnnnnnnnnnnnnnnnnnnnnnnnnnnnnnnnnnnnnnnnnnnnnnnnnnnnnnnnnnnnnnnnnnnnnnnnnnnnnnnnnnnnnnnnnnnnnnnnnnnnnnnnnnnnnnnnnnnnnnnnnnnnnnnnnnnnnnnnnnnnnnnnnnnnnnnnnnnnnnnnnnnnnnnnnnnnnnnnnnnnnnnnnnnnnnnnnnnnnnnnnnnnnnnnnnnnnnnnnnnnnnnnnnnnnnnnnnnnnnnnnnnnnnnnnnnnnnnnnnnnnnnnnnnnnnnnnnnnnnnnnnnnnnnnnnnnnnnnnnnnnnnnnnnnnnnnnnnnnnnnnnnnnnnnnnnnnnnnnnnnnnnnnnnnnnnnnnnnnnnnnnnnnnnnnnnnnnnnnaccctctccgccgaagaacaaattatcaggagtttgaatcctcctaagaaatcatttagacgacccccaaaattcactaacgaaccacctccggatgtccaattgccatcaccgcgaatcaaaccaaaccactctcatacagaccctcnnnnnnnnnnccaaccccatgcaaaccaaccaacaacgtccactgccgctatcctctcgaaacttcagcaaccttcactcacatctaatgccgtaagtgaacaaaaagaaa

>Cs1g14450

gcggtttgcgggtnnnnnnnnnnnnnnnnnnnnnnnnnnnnnnnnnnnnnnnnnnnnnnnnnnnnnnnnnnnnnnnnnnnnnnnnnnnnnnnnnnnnnnnnnnnnnnnnnnnnnnnnnnnnnnnnnnnnnnnnnnnnnnnnnnnnnnnnnnnnnnnnnnnnnnnnnnnnnnnnnnnnnnnnnnnnnnnnnnnnnnnnnnnnnnnnnnnnnnnnnnnnnnnnnnnnnnnnnnnnnnnnnnnnnnnnnnnnnnnnnnnnnnnnnnnnnnnnnnnnnnnnnnntcagactcatgttcactactacaatttttcccttctcattttggttttttggtattaatacatggtactgattacaacttcaaatgtgtctaaaagaacttttctacgtgtgccactcaattaatcctgattgaaactnnnnnnnnnnnnnnnnnnnnnnnnnnnnnnnnnnnnnnnnnnnnnnnnnnnnnnnnnnnnnnnnnnnnnnnnnnnnnnntatcaaaggactacttcacttatgatgataatgatgatTCGTCCACaaaagttacgcaacaGGATGATGTATGGATAGTGAAATGCATGACCATGTGATTCAGATTTCGGTGCAGcattcttaagattTTTCAACCCAATGCACTGAATTAAAGGATGTAATTCAACAGAGCTRGTTCACGTTACACATGGTTTCTGCAATGCCTTGAACATTAATGATCCTAATGTTTTCCTTTTGTTTCGTTTATTCAAATATTAATCGGATGTATTATATTTCTGAACAGGTTCACATCAAAATATTAACGAAcatgttgtagagtcaactgagagaccaaagccacagaagagatcttatatggcgttgccagtgcaaggcacgtgatttctcaaatgatcaagTAACCAAGTTATGTATAGCAAATGATGGACGGTTTAGATTGAATCCTGCAATCTCATTTGTGTATATCTTACAAATCTCACCCCACGTCACGAACCTTATCTTCTT

>Cs1g17850

AACTTTTTGCAAAATGTCCWGTTGGTGTGCTCTATCGTGAGATTAGAAGACTATGAAATCTGATGCRCAACTGACTTCCAAAGAAATCTTGATTAGACTATAATAGGCTCAAACTCTCAAAATGATGTGGCTATCAtgcagaaaaatgaagtAACAAGACATTGCTCAATTCCAGTCCTGTTTTGRGACATATGTTCATCACCAAGCTGCTGCAGTTGATTTGCTGGCTTGTCTTTGTTATTATGTGAATGAARCAGTCGTCATGGAATCATGATGCCCCTCGAATTATTATTTAGTAGTAWGAGACTTGTCGCAGGAGATTCCTGGTGCTGTAACATTACTACTGATTTTTATGAATAAACTTGCTCACTTAYCCTGAAAAAGAAAAGCCAACAAAATGATAAACATGGTCGGYTTATAAATAGTTATTTAATYTCATaattttttttttagaattttTTTTAAGATTAATAAATTATTTTGCCTCTACTAATaACGTTCAAATTTTAgAATTTTATAATTAGAGATTTGaAAATCTTTTTTAAAATGtgaaannnnnnnnnnnnnnnnnnnnnnnnnnnnnnnnnnnnnnnnnnnnnnnnnnnnnnnnnnnnnnnnnnnnnnnnnnnnnnnnnnnnnnnnnnnnnnnnnnnnnnnnnnnnnnnnnnnnnnnnnnnnnnnnnnnnnnnnnnnnaggcTGCCTGCATTCTTCCTGTTGAAAAAGAAAAAGAATGATAATTCCATGAATRGCTCCAAAATATTYGWTTWTGCAACCATCCCCCCTATACAAGAGCAAAAAATCCCATAGCTCTCGAAAGCYAAGTCAACCAGCTACATGGGTCCCTCCATCATGAAAAAATTAGAAGAATACAAAGTAAGTGACAAaaatcagyaagtgACAAAAATCAGCAAGTAACTGTCATTAACGTCCCTCTCTTTCYCCGGCCCKCAAAATAAAGCCTCATGCTCTAAYGAGCTGGCTGGAAGCAGGAGAGTTTTCT

>Cs1g05270

nnnnnnnnnnnnnnnnnnnnnnnnnnnnnnnnnnnnnnagatcgatataaaacagcttttactgttccctttggacaatacgaatggacagtaatgccttttggtttgaaaaatgcaccctcagaatttcaaagaatcatgaacgatatttataatccctattctgagttttgcattgtttacattgatgatgtgttgattttttctcaaagtattgatcaacatttcaaacatttaaagactttnnnnnnnnnnnnnnnnnnnnnnnnnnnnnnnnnnnnnnnnnnnnnnnnnnnnnnnnnnnnnnnnnnnnnnnnnnnnnnnnnnnnnnnnnnnnnnnnnngaacaatcactccaatcgagagatcccttttgtttgctgataaattcccagacaaaattctggacaaaacccaattacaaagatttcttggtagtttaaattatgttcttgatttttgccctaatatcaataggatgtctaaacctttgcatgataggttgaaaaagaatcctgttgcatggtcagaagaacataccaaagttgttagattaataaagcaatctgtgaaaaatattccatgcttatttcttgcaaatcatgctttacctaaaattgttgaaactgatgcatctgatataggttatggaggtatattaaaacaaaaggaaaatgacaaagaacagatagtacaatatgtttctgcacattggaatgattaccagaataattattctactatcaaaaaagaaattctttctattgctttatgcatatctaaattccaaagtgatttactaaatcaaaaatttttacttagaattgattgcaaagctgcaaaacatgttttagaaaaaaatgttcaaaacattgcatcaaaacagatttttgcacgatggcaagctattttaagtgtttttgattttgatattgaatttattaaatgtgacaaaaattctgttcctgattttctaaccagagaatttcttcaaaacagata

>Cs1g16000

GAAAGTCATGACAAATTTGCATATGGAACAACAAGAAGGCACACAAATATTTGTGAACAACCAAGCTGCACTTTCAATTGCTAATAATCCAGTGTTCCATGGCTAAACAAAACACTTCAAGATAAACGCTTTATTTTCTAAGAGAGGTACAAAGAAAAGGAGAATTGCAGCTGATCTACTGCAAAACAGAAAATCAAAGTGCTGACATctcaacaaaaagcactTTCAAAAATGAGATARGAATTCTTGAGGCAAAAACTTGGTGTATMCAGCTTCAGCGTCAAGGAGGAGAATGTTGACCGGAATGACTCATGAAGTTAAATAAGTTTTATTTGTTGTTATTTTTTAGGATTATGACCACTTCTTCATGCTATTTTCTTGCACTTAGTTGGTATGCTCAACATGTAGTAGATTTTTCTGCAATTTAGTTTTCATTATGCTGTATAAATAGAAGACTTTGTTGCTTAATAAAATATAACTTAGTAGTTTCTAAATCAGCCTTCTCTCATTATCTTTAAATCCAACAAAAAGCCTAGGACCACATTGCTAGTAGATTAGGGTAGAAATGTAGATCTATACATAAAAAATTGAACTCCTTtttggtcatatgtTTTCAGTTTCTCGATTTTACCTCTGACCTGCAAGATTCMCAGAAATTGGTGTGGGAATAGAAATTTATAGTTGTAATATATTGAATATCGCATATAAACATGGATGATGGCGCACATTTTAAAAACATTATTCAGTAAAGATATGCACCTAACATATCTGAGCAACTAGTTAGATGTTAGAAGCAATATGGCAACAATATATTAACTGAAGCACAGGCAACTTTCTCATGGCTCCAACTCATAATGCCGCCATCTATTTATTCATCTCAACTACCAAGCCCCCCTCATCACCTACATCCAAGAGAAATCATTAATATGGTTTCTATACATTGTCTGAGCATGAAAAAATATCTGAAAAGGAAAACAACGAAACAAGATACGGAGCACACG

>Cs1g11640

tcatgcatatagtgatgattcgttttgggaaagtcatgctgttaatattattgttaattataattatagaaGAAAATTTCAAGCTATTAAAAGGTCTCATTATACACGCGTATGCATATGCATGATTTATAAACATTTCTATCTCTTagttaacttttcttttcttttctttttttttttaaaaaaatcattatgaaaaacttaagccttggtgagtatttgggttagagatcaggttaaattaaattaaatttaatttaatttagtccttatgaaaaacttaagtcagggtttttgcacacaacttttttgagatattgaagtaaattaaattaaattttaaatgatatttgattaatcagataaacaaaaaagtgggaaatgcaattagtcaaaagctattaaaacttaaaaattaatttttttttatgggaaaataaataaaataagcaagcaaggaaacaatcgtgagaggaactgaaattttctaaggagataggaaagcttctaaaagattagtggtgatatggttggaattttacggattaaaagattaataatgatatggatgaaaatttatggatttaactctcggtgaattgaaattgaattctcttttaatcagagcggttacgctcatagttagatgttgaattctttttcacctacgagatttaatttttttgagtagtggcagttgtgcacatttattaaaataagaatatgatatttatttggaaatttaaccatctaatatgtattaaaaaaatggagatattaatttttttaaaaagtcattcgttatataactaagaagaaacgcagagacaaacaaaagcaaatatatatatatattaaacgcaagcatattaacttgaaattaatttataataaaacatatttatggcattacatgtggcatgacattaactttattaaatctgccttgtatcaaaaacacttttaaacaaacaacgaacacatctactctgcaaaccaca

>Cs1g03590

cttttttttatttttGATGTTCGCTGATACTTCGGGTTCAATCATTAAATAAGAATAATTTCATATAGAAACTGGAGTCGGTGCATTATTTGAAAATAGCTACGTTTTTCTGCTGTGAAATTCAGTATTGTACTTTATGTCTTTGTACTGGTAAATTGAAGATTGTCGCTCTRGGTGGGGGGAGTAAATCAAAATTTACCTTTATTTGCCTCATCTATATATCCATGTCTTCTCTGTTGTTTGCTGACTCACTACTGTGTTACTTCTTCGCAGAACGCACCATGTGATCCATGCCTGGTGCATTGTTGCTTGCACTGGTGTGCTCTGTGTCAAGAGCACAGGGAGATGAAGAACCATCTATCGGAGAATGCTTCTACAGCAATGACCATTGTTAATCCTCCACCAGTTCAAGAGATGAATCCCGGTGAGAATAAGGAACCTGTTCCATCAGAATCAGCTTCTGGAAAAGACGAAAGCCCCAGTTTGGGAATACAGCCTCTGTAGGATTTTATGATTGCARCTCTCTGGGAATCCAAATTGTTACGACTWATGCGGGGATCATCTAATAGCTGCTAGGCAGTGAGGTTTTGTATTTCATCAAAACTTGCTTTTTACGCAAGCASCAAGATGATGAGATTTAAACATCAATTAATTAAGCCACTGCTTAGCTCAGCCCATTGCAGCGATCTKTACTTAACATATGATTGCTTCCAAGGTTTTCTAACTATGTAATtgatatttcaccacTCRTACCMTTGGTTGGTTTGGTGGTAACATGGGGAATGAGCTGTAAGTTTTCAGGGACTCAAAAtttcaatannnnnnnttcatttgtgtagaTTTTCAACTATATTATATAATTCTGATGATTTAGATCTTCAAATAGACCCTGTGAAATGTGTGGGCTATTAATTTAAGCActcacaaatacaacTTAATACGTACAATACGTATTAATTTAAAACATTAATACGTATTGGCATCAAACATAGATGACTGGA

>Cs1g13033

ttttttcaaatttactagtggtactttcatttggtaaactattctnnnnnnnnnnnnnnnnnnnnnnnnnnnnnnnnnnnnnnnnnnnnnnnnnnnnnnnnnnnnnnnnnnnnnnnnnnnnnnnnnnnnnnnnnnnnnnnnnnnnnnnnnnnnnnnnnnnnnnnnnnnnnnnnnnnnnnnnnnnnnnnnnnnnnnnnnnnnnnnnnnnnnnnnnnnnnnnnnnnnnnnnnnnnnnnnnnnnnnnnnnnnnnnnnnnnnnnnnnnnnnnnnnnnnnnnnnnnnnnnnnnnnnnnnnnnnnnnnnnnnnnnnnnnnnnnnnnnnnnnnnnnnnnnnnnnnnnnnnnnnnnnnnnnnnnnnnnnnnnnnnnnnnnnnnnnnnnnnnnnnnnnnnnnnnnnnnnnnnnnnnnnnnnnnnnnnnnnnnnnnnnnnnnnnnnnnnnnnnnnnnnnnnnnnnnnnnnnnnnnnnnnnnnnnnnnnnnnnnnnnnnnnnnnnnnnnnnnnnnnnnnnnnnnnnnnnnnnnnnnnnnnnnnnnnnnnnnnnnnnnnnnnnnnnnnnnnnnnnnnnnnnnnnnnnnnnnnnnnnnnnnnnnnnnnnnnnnnnnnnnnnnnnnnnnnnnnnnnnnnnnnnnnnnnnnnnnnnnnnnnnnnnnnnnnnnnnnnnnnnnnnnnnnnnnnnnnnnnnnnnnnnnnnnnnnnnnnnnnnnnnnnnnnnnnnnnnnnnnnnnnnnnnnnnnnnnnnnnnnnnnnnnnnnnnnnnnnnnnnnnnnaattaataaaaacaaatcatgtgggccaagtgatatttaattcaatcaatcttatcatgccacatcaatttatacaaaataaatttgtacaaaaatttgtggctatatcatcactcttaatttaatagcagttcagcaagcgtaggttccgaaaatttcttagtcctgattagggtttcactaattccacctgaaaattgatctcgcgacagcaattaagacataaaattaga

>Cs1g25990

ctcatcccctccccgaataataacaggggatccccgagggttccagatcctcgaataattaatatattttttttgttttcgattttgagttaatcatattaaaataaaaaattcaaataaaagtaaagttcgaaatatatcttacattaatatccattacaaaagtcacatacattaaattagtaagtaacacaacatgcaatggatacaaactatcatgaacaaattaatagcctaatacaaaattgttacaaataaaatcaaatttagattcaaaattaacttttcaatggtggcataggcactttataaatgtggactattccctttacaacacaatagttaagtcgaagcaaattaagagcaaatnnnnnnnnnnnnnnnnnnnnnnnnnnnnnnnnnnnnnnnnnnnnnnnnnnnnnnnnnnnnnnnnnnnnnnnnnnnnnnnnnnnnnnnnnnnnnnnnnnnnnnnnnnnnnnnnnnnnnnnnnnnnnnnnnaAATCTCCGAAAATACCCGTTCTGTGGGAATTTTTGCCATCCCTAATTTTGACAAGTTGATTATGGTTAGAGATGAATTTYGGCATAAAAACGAAACTACTAGGTCTGGCTGTGAGCCGGTTGGGTGAAAAATCGGAGCCTGGCACACATGAAATTGTGCTACAGTGTACAAGTTCCAAAGTCAGTTGCGTGTGTGCATTTTAATAGTgtacaAGTTCCAAAGTCAGTTGCGTGTGTGCATTTTAATAGttttttttAAAATATTAAAATATTTAAAATATTCTCTAATATTTTAACAATCTCATTTTtTTTTTTACAACATAATTTTAAAAAATTTATTTATTAAAATAATTTTATCATTTTTAATAAAACGTTACGATCTTAGgtccttattctataatTTTAATAAATAAaaaaaatggggtctCATTAGAGRTAACCTCTACTCGGATGTTGGAAGTGGAACACAATTTGTTGATAATTACAGTAGTATATAAyctaac

>Cs1g15540

CATTTCTACCATCTTAAAACCCCAATTAGCATTCTTTTTTTATGTTATGCAGAAGTATCCTTTTAGATAATTGACATAATCTACATTACTAACCCGTTGTGTGTATTTTGGTGTTCCTTCCTATCCCCCCGCTTTGCGTTTTCAACCCCcCTAATATatattgtaatacatacaatagctaatgaaaaatgaaaattttatagggttggtcttttaagcccccttcaagctaggatgatcaatcgacttgtcttggggtaaCgggcttcctccacttttacttttgtttacttattcttaatgtttgtacataggaaatgagacttaatccaTATTTACTGCGAAGAAGGTGTTTTCTTTCACTCACATACAactatctaatttcttttattaacctaaagagtaaataaatgaataaaaaaatgaggatttctattcaagttcttTTTTTTTTCCtagaacgaaaagtttaggttttagcgaatcgcatgttgaaatattgataaaacacataaacttaatggtatttcataattaatcgattgagtaggatcttgcaaaccaCTAAAAAAAggaatatttattatttgatccatatccagacataagaagtccaatagaggcaatacttgaaatggcaatccagaAaAAAATACCGATATTGAGGTCAGCTAAAACAAGGTTATAACTAAAAGGAATTACTGAATAACTTAGTAGAATTGCTATGACTGCTATAGATGGACCAATACTGAATAAACTACTATTTCCTCTAGATGGAAGAAGGTtttctttgaaaaggagttttgtcccatcggctaaagcttgaagaatccccaaagggctggcgtattcaggcccaatacgctgttgtgttcctacaaatatttctctttctaaccacacaattaCACTGATTGTGATTGACAATACATAAATTATTCTTCACATAGAAAATTTTCATCCATATTTTACTAAAATTTATTGGAGAATAAAGTGAAAAA

>Cs1g15350

AAATAATCTTATCWATRGTGAAGGAAATAYTAKTCTTGAACCAGTGAATTTTATCAATCATTTAGGAAATTGTGATTTTGAACCATATTCATTTTACTATTTTATGCTCTATGTGATGAATATCGCATCACCaagacattttttttttaataaAtctttgaaaaaaattattACATGATGTGTTATTAATATTACTTATAATAGTGTATTAGTAGTATTGTTCTTTATTAGCAAAATGTGTTTGAAGCCACTGATTTTGATATGATGTTAAAATTACAAAATTTAAAGTTTCAGTCAAATATTCACTTATTTATAGTCTGTAGTTGAATAATAAGTATTTTTATGTAAGTTATTAAACTGATAATGCCTATAAGTATATTAGATAAAaaaaaatttatttaAGTTAGGGATGAAAAAAACGCCCAAGTTYAATTTTAYCTCGGTTGACTTGARTTAAWTTTTTTAGGCTTTAACTCGAATTTAAYYGTCAAAATTTGAGTTCGGGCTTAAAAATATATGTCCAATTAAGTTRAGGTCAAGATTTGGRTATAGCCAACCCYACCCAACCCAATTACTTAATAATAAAAAtAAAtatatttttatttgaaTtagacattttttttnnnnnnnnnnnnnnnnnnnnnnnnnttttgtggttgggatgttgattagagtaATTTTTATGTTTAATTTTAATATATTAGAATAGGTAAATTTAACTTATTTAAATATTAAAAAATTAAAAATATATTTAGTTGGGCTTGGGTCCTGACCCAATTAGAGTCGGGCttmgtgaaaaattsaaATTTTTATCGGATTRGAGCCCTTTATTTATGCTCAGCCCAAGTTCGACCCAACAAACTACCGTACCRAATTTAAYTATTTCCCAAAGAATTTGGCCGAATAATTTTAAGTAATTATTTTAGTAATGAGAGCAATaatcagtttttttttttttttaattataTCAGAAATAAGTTTWAAAAAAATAGTAATTAATA

>Cs1g09080

CCTTCTTTAATTTAGAGATAAAGAAACAGGGAAAAWTTGTTTTGGTATAAAATGTTAATATGCCCAAACAATACAAATGTGAACTTTGTTAGCCAGTGTGGTGAGAAACGAGAtgaataggacttggaGGTGGTGGGGTGTTGGTGCTGATTTTTCAGATTGTTTGAGGTTAAGATGGGATTTTGGAGGAGTTGGGGGAGTTTTGTGCAAGGGTGGTATGATGTAATTGAATAATAATTTAAGCTTAGATTACAGTAATTATCCTAATAAAWTTTTGTCTGAATTAATATTTGCTGTTCAAACCCCCTAACCTTACATATTCGMTCCGGTCATTAATTATTTATTAAGCAATTATTTATTACGTATGATAGCAAATTTGCATAAAATTAGATGATGATATAATAATCAGTTTCAGTCAAATACATAGGGGAAATAACGATTGGGATCCCTGAATTTTGACAAAATCACAAATATAGTATACATTTTCTTTAAAYGTGCCTACTAATTTTGAYAAAATCACAATCACAARGGTAATACTAATGGCGATTTATGAGACGGCTGGTCAATTTNAAGTCAGCACAATATAAAAATTAATCTTTTSARATTATAATATGTTGAATTCGTTTCATCTATTTAATAATTATGTYGAGTTAACCAATCAACTTTAATRTGAGCTTATTTGTGACAAAATTAATTATTTAAACAATTAATANAATATCTTATCYAAAAGAGTTATATTTTATTTTATTCTTTTTAAAAATGCTTAGTTATATGYGGACGAAAACTTATTAAMGTTTGAATAGATAAAAGTTAAATTAATTAGAGYAGTATCACGTGAACATCGTTTAAGAAAagagagggggggGGGGGGGGGGAGATAAATAACTAATTCACTATGGTGGCGTTTTAGATAATTTTTAAAATATAAGGGATCAATGGRTAATTATTTCAGACTACAYAGGGTGAAGGAATATTTTACCCCAAATTGTCTTTAACGTATG

>Cs1g08810

TTCAGCCGTTGGATCCACGATTGGAAAACTTATTATTGTTATTATTTTTTCTGGATTATCCACCTTATCCTTGAGGCATTCTTTGAAACGACGACCGGGGAAAGCTACTCATTTCTCACCTCTATTGGATATTATAATAATTATGATTATTATTAATTATCCGCTAGGAAAGTCTATAATACAAATCTATACTTTTCAAAGCACACACACGCGTTCCCACTTAACAACCACTAATTTATATGATATTAACTWGTGTCTCGGTCGAATAATGTTGATGGCACCGGTTTGATGGAATTACTTTTCTTCAGTATAAAAATACAGAATATATATAAATTTTTCTTTAAATACTATTAGTAGAACTAAATTTTCAAGTTAATCTCCATATATTTTTAGTATATATGATAAATATATGTGTTTAGTCTCAATAAACGTAAAATTGTAATTTAAAAAATTGCGACATTGGACAAAATTCATGAGTTTTATCAAAATTTAATATAAAATAATTCAGTTTAATCCCAAATTATTTATATATAATCGTTCATTTAATTATCAAAAAATAGGAGTTGAATTGATGGACTCCATATTCTAATTATACATGTTACATCTAAAGCAATTTAAATAGCCAAAGAATRATAATTGGAGAAGATCATGGCAAATTCATGCTGGCATGCTGCTTCCAATTTCCAAAAAGAAAGGAACGAATTATGTGAGTCATAGGAGCTGATATGATCGAATTTAGATCCTAAATTCATTTTTTCTTTGATTTATGTTTTTGATATCCAttaataggtattcGATAAAAAAAaTTAGTAATAAATAACTCACTAAATTACACACAATTATAAATGCAAATGAATGATAAGATTCTTTGGAAGCAAGTAAATATAAAATATAAAACGAGgtcaagtcaatTTCGRAGTCAATCAGCTTTATAAGATCAGCTAACATTAGTTACGTGGCGTTGACATGCAACTAACAAGATAAAgatagttaaattcgaA

>Cs1g05520

acagctggcagaacttgaagaaattcgcaatgacgcgtacgaaaatgccaagatttacaagcaacgaatgaaagtcttccatgacaagcaaattatgagaaaatcatttactccaggtcagaaagtgcttttattcaattctcgcttgcacctattcccaggtaagttacgctctcgttggtctggcccatttattgttcatactgttttttcacatggggcaattgaaattgaggacccaaagaatggtgtcacgtttaaagttaatggtcaaagattaaagccatatctagagtaccaaccacatgaagaagacaccgaaataaatttgagtgacccaccaaatttgaattgatttttttttcttttcgttgatttgattttgttttctttctttatattattCTTCTGCTAATTGAAATTATTTTTGCATAAGTGTGTTTGTTTAACTATTAAGTTTTCTTTTATCATTTTTACTCATGAGTACCTTACTTAGACCGTTCctcctgaacattcttaaaccactttaacatgtcaaaactcatctcaaaaggcagattcaattttgtaaaacacatcgcatttgggatgtacagccaccagagttagtagcctatgttgagggtttagaaagtcaactcagagacattgagagaagtgtttacgacatctagttggagcttgaggtaaattcaataagaggacgattttaattttctttgtgtgttttaatttgtttttctgtttgtgtgctttagtttattaccctggtgaagtggcggataacggtactctgtgacaatcaagtcggttacttcaatttcccataataactgatattctgaagcgaaggtatggacaaaaacaagactttagcgaaacttcacaagcgattcccttcacttcctcagaatgccctccttacgatctataaagctcggtccgaacgcatgcgattactcatgaggaataacatacctgctgaannnnnnnnnnnnnnn

>Cs1g11980

ctaaatnnnnnnnnnnnnnnnnnnnnnnnnnnnnnnnnnnnnnctttggtggaaatcgaccttatttaaagagaatgagccccatgccattgggctacaagcagaagcggcccacaaatagtagagtatctaaaatagtaaaattatatcatctccattgtccagaacactttatatatgcatttccagttttccactatatatgcatccctagtatactttaagcgttgaagaggtctgatggaaagaaaaaaacaaattgattagaatccttcgtttctaggggatcataaaaggctttattatttttctttttcgagtggtgctttttacaaataaaaatgcacaaagctagtaaaagatgaatctattcactttatcaattcaataatgatatagcacaaataattttatacaAATTGGTATGTACCAACTGATGTGGCATAAAGAGATTTATTGAAAAGTGACACATTATTTTTTTAATTATGTGAATCCACGATTGTTTTATAGAACCAACCTTCGATTTTTTAAGATTGTTAACAGCAGACGGGTGATATTCAAAATTATTGATCGAATAGGTATAACTTATTACCTGATTCTTCTTTAATTACAAGAATTCTGATCATCGAAGTTTTTTGAACCTTCCCAAATCAAACTTGGCTGTGGTTGGACTGATAATTTCTTTTAGTATTCTCACAGTAGATCACTTTCACAGTTAATTGGAACTCCACTCGTGAGTCACGGACGCGGTAAGTGTAGACTTGGGAACTAGCTAAAGTCTTAGTATTAGGACCTGTTTGTTTTTATATATGATAATCAACACAAARCAGTCTAATGATTCAACGTTMATGGYTCYGCACGCAAGACTTAATTGGATAAGACTATTTTCAGCGTCAATCYTATTTRATACGTTCTAAAAGAAAAGTCTTTGTTACTTTCTAATTAGTAAATTTCCTTTYAGATGAACTGTATCAACTGGGGCTAGTAATCAGTTGAGCTCA

>Cs1g05340

ccctattgaaggcaaataacgtgggttttattttttgaattatttggagcctagagttttcactaaagggtgatataaaaggcttattttctctaaagaaaagagggaagccactttatacaaatcaaagaataagatttttctctccatgattgagagaaaaattttctcgtgctagttgctttggtagtgataaaggtgcccacacgtcaagtgcagatcgaacctgagtcataacctggaagatcattggtgacagttcgtgatctaacaagcgtggtggtgacagatcgtgatctaacaggagtggtggtggcagatcgtgatctagaagcctaaatcacttcagcggaaaaagccaactcgaattttcaaggtacgatttctagaatacgaattcttatatattatatgagagcgatcttcaaaaggttttataaaaatttaaaaacctgatttttccccaacagTTAAAGCTTGAGATGATAGACGTTGGAGACAATCAGTTAATAGGAAAGTTCCCAGCTTTCATCGCAAATTTTTCAGCTCTCGAGGCAATTGATATTTCCGCGAATATGTTAGGCGGGAGAATTCCTGATAGTCTTCGTCAACTAAGAAGCTTAAACTACTTAAGCATAAGTGAAAATAATTTCTCCGGTAAGCTTCCTCTTTCAATTTGGAATATCTCTACCCTTGAGATAATTTCTCTACATTCAAATAGATTCGAAGGAAGTCTTCCACTTAACATAGGTTTTAATCTTCCAAACGTGAATTTTCTTAGTGTAGGTCAAAACAATTTCACAGGTTCTCTCCCACACTCGTTGTCCAATGCTTCATATCTTCAAGTGCTTGAACTTTATAAAAATCATTTCAGTGGACAAGTGAAAATTGATTTCAACCGTCTCTGGAATCTGTCCAGGCTTTTTTTAAGTAAAAATAATTTAGGAACTGGGTCAACTAGTGATCTTGATTTCCTAACTCTTCTCACAAACTGTAGCCAA

>Cs1g16026

TATTTAGGTTTGATTAGGTCCAACCCATTTCTCAGCACATTGACCAGACATTTgactttaaaaaaagaAAaaAAGCAACCAAGATTCCCAATCTTTGCTTAAATGTCCGTAGACTGCCTTAATTTCAGCTTTATTTYGTATTGAGYTTGGACAATTATAATTTTTAATGTACAACATTAAAGTGTTTAATAAARACTAACTATTAGAACTTAAAAYTTAAATTGATTTGGTCAATTATTTTTATGATGAAAAACTTATAATATTTTTACCATTTTTGTCYCAATTATTATTTAAAAGTTATGTATAATAATATATTATTAGCTTTTATTTAATAARTATAATTTTTTTCARAYCAACTGAAATTTAAAAATTATAACATCTCAATACCAAACATAACTTTAAGGTAGTCRAACAGTCTCTRTTGCATATAAACCTTAGGATTATTGTAATTATCTTTGAAAAAAGTTTATGTGACCATTTTTGAGACTAAAAAGATCTTTGTGTAAGGTAAAGGCCGATCCGAAGATAGTTACAAAATCCAATAGCACAAATAGTataaaacaaaacTTGCCCTAAAATAATTACAAACTGACACCAGCGGGGATAAAATATTGACGATGGCATCGTTTTTCTGAAACAGTGTGCAAGGACACAGGAATTTGTCAACTATGAAACCTCATCAAACTTTCATTTTCGCTGTCGTATCAAGTAAAATTGTTTATGCCAAAGCATTTCCcggaagacacacacacacacacattataCAAGTAATCAAACAAGTGGAAAGCGAGTGGCATGGCAATAAATTCAAYTAACATTAAAATTGCATGGTAAAAGTCCAATTATGCGGAGGTGGCTACTTGAAAATTTCAACTAATTAAGACAATTTGGGCTTCAATTATTTTTATTGGTGGGAATTGAACCTTTTTTATTCACATTGTGATCAGTTCTTACAAGCATAAATATGTCTCCTTTGCATTGACAATCTCAGCAAAAACAGT

>Cs1g15230

AATTCTTTTCYATATTCTTCACACTCTCGCTGAACTTGTTCACRGCCCCAGCAAGATCTGGGAAATTCCCCAAGGACACTTTCCCACTAAACCARGCCATCCCCYAGCTACAACACAGAGCTTAAATCCACTGATCCAAAAGCATAgcatccaaaaatctgcCATTTAAACACACAATTCAGAACTCGACAGACAAATTAACCAATCATTGAGAAAAATCTASGATTCAGAATGTGCTAACTCTAACACGTTAATCATATACGATAATCTTGATTAACCAAAATGAAGCATTCACATTTGTAATTAGCACGTGGAGATAAAACCTATATACTTGGAGATCCAAATTGAATAAAAAGGGAAAAGAATAAAAATTAAATTAAATTATAAATCTTGCACAATGCAAATCCTAAAAAACGATGAAGTCTTTATTCGAGTTCATTTTATTTTCGTACCGTTAAAGAGATTGGAGATGACGATGAATGCTATGAGATTGAAACCGAKAGCCGAATCATCACTCTCTTTTGTTTTCACCTCTTAAGCTGTTGATGAATAATGATATCGYCGTATCAGATTTGAGCGTAATAAACTTTTGTTGAGAGAGAGAAACGAATGAACGATCAACGAWCTTTAAATTAGTATCGGTTTMGAGAAAGGTTCGGGTTGAGGGATCGATCTGATTATTTTCYCTGTGWATTTTCATGGGGCTTGCTGACAAGTGAGACGTCGTCGTCTTGATAACTCTTTttaatattttgttttATTCAATTTACAAATATAATAAATTTTTAAATCAATAAATACCAAATTAATGTAATAAAATTGAAATAAGTGCATTTCGAATAAGAATTCCAACCATTTCTAGGATCRGCCTGCCGTACcacttcttttgtcttTTGTGACAAATTTTCAWATAACCCCTTTCACTCTTTTTTATTTAAACTTTACTTTANAAATATTAAGTTAATTTCAACTGCATCCATGTGAACATTACCGAAATACCC

>Cs1g15020

CCAGAAATAAAGCATACAAAATCCATTCTTACACAGAGCTAAAGATCAATCGAATTGCAGTAAAGTATCTCACTTCAACCAAGAAAtctcacttcaaccaagaannnnnnnnnnnnnnnnnnnnnnnnnnnnnnnnnnnnnnnnnnaacatccaAATGCATAAACAAACACAAACACGAATCTACCGATAAAAAAACTGCTTTGAATGWGCATATAAAGAAGAAAGTTTAAGGAAGCATACACGTTCTCTTGCGAGCAGCGGGGTAAGCACAAGCAGCGCAACGACTCTTCTGAAGATGGAAGCTGCGACGGCCACATCGCACACACAAAGTGTGTGTCTTGTTTCTCCTCTTACCGAAACTCCCTGTTCCCTTACCCTGTCataacccaaaaaaaaaaaTCCACTCAGAAAATCAACAAATTTCAGTTTTCGATCATTTTAACTCACGAAAATGGCACATAAATTGAAATGAATGGTACATGCAGAATCAAAAYAGGAAAATGGGAGACGGGTTGTGATTTGTTTTTACCATCGAAGCGTATGCGAAGcagaggaaagccttCTGATGAGTGAAGAGCGAGCGGCGGAGACGAGAAGGTTTATATTGTGGCTTTGTTGCGTTAAAAAGGAGGGTTTTTTTCTAGGGTTGGCTGATGAGGGTTGAGTGAGGGAGGCTACGTGTCAAGATCTTGTTGGGTTTGATTATAATGGCTCTACTAGTCTGATTGGGTGATTCATTAACTGGGCCTGGATGGGCTTAATTTTTGCCCAACCATACAAGCCGACCCAGcccatcmtttattaatattaatttgTTCAGAGTATTTTTTTTTTTCTTTCGCGGGAAATTAGAAAACATTTAAGAAAAAGGGGCGCCAAACAAACACATCGaTatacaccatttaacttaaagcaaataatcaacaagaaccctttttacttacaaaatcaagcgCCAAAAAGAAAAGAAAAAAATTGAAGCAAGATCAGAAAGAAGAAG

>Cs1g06370

TGAAATGAAGTCAGCCAAGAGTGGAGAAGTGGAGTGGGAAGATAACCTTGGGTATTTCATCAAGAAGGTAATTTTTTTTCTTGCTGTTACAATTGTGCTGTTTATTTTGTCACTCTTAAATTTTTACTTTTnnnnnnnnnnnnnngatttctTGGTTTCTCTTTTTGTTTCTTAGTGGTGGCAATGAATTTTGTMTGTTAAATGAAGTTTTGGAGTTATTACATAGACTTTTTCCACTAGTTAACGTGACTGATTTTATTGGGTAAAATGAAGTTTTTAGAGAGCTTCTGAAAGCTTTTAAAAATTGGGTATTTTACCAAGGGGTAACTGAGAGTAAAACAAAATTTTGTTCGTTTTATATTTTGCTGGCTTTTTGGTTATTTTGTTAATTTTGGTTATGTTAGAGTGGGTTTGAAGTTGTGCTAGTGCATACAGACGGGTTTGAACTCTTGTaattggttttattttCACCTTTCATGTGTGAATTTGAACTTATGAGCTTTAGAAGGCTTTTCAATGCTTGATTTATTTTTTTGATTGATTGAGCATTGTAACTTAATCTTGGGAGGGTTTTCAGCTCTGTAACTTATATTGGACCAAGAGAATTTTTGTTCTATATGTATGCATGTGACCGGTTTTGTTTCWTTTTGTATTAGGAAGTAGAAAATGTGACCTTAAAAAATGGGTAGGACATTGGTAATTGCTGAAAAATTGGATCGTTGCTATTGATTTCTACCACTAGTTTTATTCTGTGGTTGGATATAAGTTAGGAGTTGATAGTTTATGTTTGATATAGTTTGTCACCGTAAGCTGTAAACTTTGTTTTCTAAATGATGATTTTGAATTTTGTTTAGAAACTGCGTGTTTGGTGTCGACTTGAGGATGGAAAGTGGGAATCTGGAATGATACAGTCCACTTCAGGGGATGAAGCATTTGTTTTGCTCTCCAATGGAAATGTAAGTTCATAAGTTTTGAAGGTCTCTAAAAGCCATACAAAATTC

>Cs1g05150

gttcctcaccaactgaaagaaacaaaacttattagctaagattcaagctagctcactgttaggaatattaatctaggcaaaaatacgctacctggacattattcctaccagagaaaggtgggtagccattaagaagctcaaaaagaattgccccaacgctccacatatcaacctacaaagaaagttttaaaactttcagtacattctgatgcattttaatagcacagaatgattatctagggggaaataaagaaattcagcttctcatcatatctttgaaactgaagaacttcaggaaccatgtatattggggatccacaaactttctcagcataattgcctggatgcagggtgctgcttaaaatacgaagtaaacagtcaattttaagctccagacttttcaagaatgaaacttctgcataatgtttacattttaaaactacaatagaaacaaaagaacagaaaaagtcaacattcaaacacacttccaggacattgtcaaaaataagaactatcctcacacaatatatttgtatccttctactaaaagtttaccatgacagaccaaaatctgctatcttgagcatcacatcatcatctaggccgaatagtaaaatgttctgttccaccaaaagcaACAGCAAaGTCAATTTAAaAacttacaatctcgaaaacatacaatgttttaaaatgaaaacatataaattaaaATGAAGAAAATTCATGTGTAGAAACACTGAAAATTTGGCAGTTTAGTGACAACTATACAATCGCTGACGACATTCAAAGTTCCTTTGAACTTCCCAAAATAGGAGTACAAAATAGTAACTCATCAAACTTTAAGCTTGTTCAAGATTGTTCATTTTCTCGATTTCATCACTTTCTTGACAAGCACACTAAAGAGCAAACCATATAAAGCaAccacaatcctctagggaactccATCCTCTATGATTATCACCAACCAAGACTCTCATTTAATTATTCATATTATACTTCTC

>Cs1g08760

acacacacacacacttcttgtcaccatcttcgactgaaaatgagacactagcttgtatcaaaatctctaccattacaaatttgctgcaccctccacaagttctctttacagttccaaaagcttcattccatgtacggacttgtgtcttgtaacccaaaacttggaagggcaatgatcacattgaacattgtaacaaaataataaaaattggacatagtaacaaaatcataaaatttgttatggctataatatacatgatgtaagataaatgaaccatttccctacatgaccacaaagggataagctaattgaccatgggttatttttacatacctcccttgaggtttcacataatatcagtttaatagaaagtatctccatatttttacatccctcccctgcagttagtatttccgtaatatcagttnnnnnnnnnnnnnnnaattgaccatgggttatttttacatacctcccttgaggtttcacataatatcagtttaatagaaagtatctccatatttttacatccctcccctgcagttagtatttccgtcagttgaccgttagtggactaacaagaggacgaaaataccctttcaaggttgacagtcttctttcatccaacaaaaactcaaatcccctgtaccacctcaccaacaaaaccacatccttgttttcttaaaatcaagctttccatttttgatatttttccccaaaaattcaaataaaaaatcaattcgaaaaaactaaacctcataacatgtccaacgtcatctaactttagcttggtatccataacaacagcaccaaatgttgctatgaaagcactgtcgactaaacttcaatttatgtccgcggcacaaccaaaataatttatatcaattaaattagccaattctaaaagcaaataaacacaattcgtccctctgccaacaacaacctgcaccagtgagaaaagaagaaaataagatcggttccacgaagaagaaataaaaagaaaa

>Cs1g09363

tggagatctttaattaatgatttcttttattattattttttataggactctgtcatatcccttgtagctgatgtacgtgttaactactattgacaaaaatatcagtttagttcatnaacctataattaatgggaaatttacaaaaatagccatacaattttagcgtttttcaattttaacccccccaaattttttctatcaacattagccaaatcaccaaattgtggacgaaattgccctcatcattctcacgtttccctcaaccaaattcctccccctctcaccaaaatctcatcaccggaatcccgtatacatcgtcagtggcatagatcggcagcggaggaagttgtcggcggctgaaacaagctaaatcacgttgaaaatctttggagatctccaaaatcaaagacaaaaattggaaaccctaggtgagttgttattgtcgttgttgttgttgttgttgtttgggctgttgttggtgtttaatggcgnnnnnnnnnnnnnnnnnnnnnnnnnnnnnnnnnnnnnnnnnnnnnnnnnnnnnnnnnnnnnnnnnnnnnnnnnnnnnnnnnnnnnnnnnnnnnnnnnnnnnnnnnnnnnnnnnnnnnnnnnnnnnnnnnnnnnnnnnnnnnnnnnnnnnnnnnnnnnnnnnnnnnnnnnnnnnnnnnnnnnnnnnnnnnnnnnnnnnnnnnnnnnnnnnnnnnnnnnnnnnnnnnnnnnnnnnnnnnnnnnnnnnnnnnnnnnnnnnnnnnnnnnnnnnnnnnnnnnnnnnnnnnnnnnnnnnnnnnnnnnnnnnnnnnnnnnnnnnnnnnnnnnnnnnnnnnnnnnnnnnnnnnnnnnnnnnnnnnnnnnnnnnnnnnnnnnnnnnnnnnnnnnnnnnnnnnnnnnnnnnnnnnnnnnnnnnnnnnnnnnnnnnnnnnnnagagaggaagagagtgtttgacttggtttgacccggatccgacccggaaacccgcgaccttgacccggacggatctccc

>Cs1g21680

TATATCCACTAGGAGTTAGGATCTGGTTTAAGTTTTCTTGTAAATCACCGGCATAGAAGATTGTAATCAGCACACTGATTATCAAATTGAATTGGATGGATCCAAGATTACAATCGATTGAATAGACTTGTCCGATATTAGACCAAACTACAAAATGATCTAAATTAGCATTTGCTCTGAAAATGAATAYACTGCACCAACAAGCGCGTAGATCAACTGGCATGTAGTTGTTCTAGATTAATCAATATcCTCGGTtCGaggcttgggaatacagctgcgttaaatacttattgggagagttttgccgtcctattggtcctacccggctcgaagctggattagtcgggacccaatgtggtcttcGGATACCAGATGGTTAAATACACCGAAAAATATACTGCAAGAATTCTTTTTTTTTTTTCAGTTCTGTTTGACTTTGACCCTTCAGATCCAGAAAAATCCTTGAAGTTTTACACCCATCGTAGCTTAATTAAATGAAATGCAAGCAAATAAAAAAGGTTTTGCACCCAATGGAACAAAGAAACAAACGTATTGCACTGCAATGGGAAGCAAATAGAAAATGGTTTTTGTTATCGTAATAATGAAATCTGGCAGCTAATTAACGttcagttgcgttaGCTCGGTCAAAGGGCACACAACGAGTTGCGACAATTATCTTAAATTGCACGAGTTGGATTGTGTCGCTGTTAAGAGGCGGCTATAGCACCTGCAGGCATTACCAAAGCGATGACCGAAGGGAATACGAATTAATAATAAAGCGCACCAGCTGTGGAGACTATAGGGTGATACTACGGATGGGACCCTATATTTTATTGTTCATCAACAAAATTGGTCAAAAATTGAGTACTTTGACTACGCTGTTGTACCATGTCAGCAGTCACATCTGTGGCGGCCGGTCTCAATTTTTAAttaaaaaaaaaaaaaaCTGATATTTAAATTTATGGGTTGCCTTGCCTTCTCAACGGTTGCATTAATATCTC

>Cs1g22850

CCATCAACAAaaataaaaaataaaaTAACTATCATTGCCAAGTGGGTTTTATGATGGTACAGAAGAATCCCTATCTTTTTGGTGCTTTTCGGTGATAGTGGAGGATCCCAACTTTGCTCATTTTTGTATTCTGTTATTATAAACTAACTACGATTAGCTTTAAAAAATAAATAAATAAAACTAACTCTCCTTGGCTTTTCAATCGTACGCTTTATGTAARCATAAAACCGATTAAACAGATTGCCGACCTCGCAACATTCAAACTACTATAAGTTAAGTaacactcagcatAATGAAAGGTCAAGTGGGGAGAATAATCAAAATACCTAAATTGTTTCAGTGAATTAATTTACTTCTCTATTTTTTTGTAAACTTAATAAaaaaaggaaaaaaGAAAAAGAAAAAGGTATTGAGTTGATTTttaaatttttttatctttTAGACTTTAACTATTTTACCTTTGACGTAGTAAAACTATTGATCAATAAAATTTTTTTGGGGGTAAAGTAATAAAAGAAACGGAAAAGTTTTAAATGCTCCTAAAATACTATTTTATTATTTTTGAGCTTGGTGGCAGGGAGGGGGCGTGTGTGTATATATATAAAATAAAATATAAAATCAACAGTTAGTCAGTGTCACTGTTTCAGTCTCCCATTAAAATATTGTTATTAACATATAAAACTTGGGAGATAGATAATGAATGCAAATTGCACTAAGTTAGCATGATTATATGCAAAAGAGGGATAAAGACAGATCTTCAAAATATACAAAGTTATAGAATAGGGAGTTTTCAAATTAAATTAAAGCYTCTGACACATACACCGTCCATTTGATAGTGGTCCGCATTTTTTGTTATGAAATAATTAGCGGTTAGGATACTTAATGTGCATACATTTTAAGATTATAAATACCTGAGGAATCAAGAGCTTATTACCATCGTCTTGTAGCTTTCTATCTTCAAGTATTTCTTAAGCCTTTCATTCGATATCCTTCTCTAACAA

>Cs1g26375

CAASTTAATTTATTAAGAGTCTAAGTTCATATAATTATAATAWGTATAATTAATATTGTGAGTCCGAACGACTTGAAAATTTGTATTTAATAAAACATGCTCRATCAYATACTTATAACTATTTATATAGGGATGTGAATAAMAACTAGAACTGCGTTTTTCTAKKATTGGAGGGGAGGGGTTTATGTTGTCGATATTCCAAAGACTCTATTATAAATTACAATTAAGAGATTTTGGAAAAAAAAATAATTTTGMTTTAatagttcaaattTGAAATTgaagaatattcatgtCGATGGATTATATTGAAATAGAAGTATATAAATCTAACCTACATAGTCTGGCACATCCATTCTCACTACACAAGAAAATTAAGGAGTGTCTTAAacTTAaAATTTATTTTTATTCATGTAAAAACTTATATGTATTTTCTTAAAATACATCAATTATTTAAGTTCTTAACTCTACGAACTTAACTAAAATGCATGTCCCCATAAGTTAAAATGATTAAATGTATATCCCTTTGAACTTAATTAAAATGCCACATGAATTAAATTTGTAATTACTCATTTCAAGTTCATAGTTAAGTTGCACCTATAAACTAAATTTCAAGTTTGGTCCCTGTCTGAAATGAACTCTtctaaattttttaaaatTCAATACCTCGTTCGAAGATATAAAAACGTCCACGAGGATACCACATTGTAAATGTTACACAGGAATGATAAAAAAGAAGAAAGTGTTTAAATAAAAAGTAAAATGAAAAAGGAAAGAAAGAAAGGAGATCGAAGAGAGTAGTTGAAGACTTGCAGAGTAGCCTGTAACCATGCAACTAGCTATGCGAAAAAGCCCAAAGGGTTGAACCAATCACAAGCAAGCAAGTATAATTCCATTAGAATCACTCCAGAGCTAAAAATCAATCTGAACCGTTGGATAAACCTCACCAGCAGTGGTAGGGTAAATGATAATGCCCCCATAACGACATCCATTCTTCCACACAC

>Cs1g09030

TATCATCATTTTCTTGGACCCCAAACTCATAAGTAAATTATAGCAACTAAAAATTAATAAAGCACATACACCCATGTAGATTCTCTCTTTATCCCTTTTCCTCTTGCCAATTttttctttttttttttaaaaaaATATTTAAAACATCACCAACTCCAACTACATGTGCTCGCACGCaCACGTAAACAACACCCACTCtCTTTCTCGGAttTAtttttatttttattttttaacaaattttctccctcacaaaaagaagaaagaaaacggaaaAAGAAAataaaaaaaAATAAAGTGACACGTCAtcctcttctctttATTAAAAAGTGMCACGTCATCAGCCTCCCATTATCCAACCACCGCTTGCCGTCCCCTTTCTCACACAGCCTCCCTTAGCTGTTCCTCACTGTAGCCACTGGGCCCCACTTCACTCTCTCTCTTTCTCTGTCCGAAGCCATTTCTCCATTTGGGATAATAACATGCTAATCCCTGTTCTTTTTTCGCTCTTTTCTCACACTTGCATCAAATAaatccaagagaagagaactatctTAATGACCAAATTGGGCCTGATTTGATATTATAGTTAGCCAACTGTACTTTCTGAAACTATCACATAAAAKTATTTAATAACACTTATTATTATAATTTTAAAAATTAAATTAATTTTATCTCACATTTTTATTTTAAAAARTTATAATAATTTTATTATTTTTGTAAAAAATTTAAATAAAAATCATTTTTATCGCATATTATTAAGTTTTATTCCGTACTACAAAGGTGATGTATTGCTAATTACTAACTGGGTACCTTAAAACCATATAAGACGTTACACAATTGAATTTGACTCGACTTATTTGATACAGACATAGAAACTATATCTATCTCTGaaaaaaaaaaaaggAAAAGGGGTCTCAACATTATTAACCCATTTCTCGTTTCCTTCCTCTTTCCTTCCTCATATATAATCTTCTGTCAGAGCCCAAATCAAAACTTTCCTT

>Cs1g02620

TTTCTTTATTTGCAATATATATATAATTGATGACTATGAAACCAATTACATCGTCCAYAAATACTTGTTGAAATGTATAagattgaaaaaaaaaggctaACTTGAGGGCTATAGGATGCATATAAAAGGGCAAACACCATGGTCAATGGAAACTAAATGGTATGGACAATATAAATACATACATACATACATGCACAcacgcgcgtgcgCACATACACACACaCATATACATACATACATACATACATACATACATACATACATATATGAGTTTTCAATCAAATGACCCGAACTGAACGCCCACCCCAACCTATAATAGAAGATGTGGAGGAAAGAATTAAGGAGATGGCATTAAAAATAGATAAATAGAGATACATGGAAAGCAAAACTTAAAAAAGCCAACataattatttggTGGGGCTAATTTTTATTTATTTTTTCATTTAACACTAATATTGGTTATCAATTTAATTCTGATTTAAGGATTGAAATTTTATGTAAAAATTTGTGTTAGAGAAAGGGGCTCCCGATTATGACCGTGTATATGTATGTAtgagtgtctatatatacacacacagatgcatacatacatacattcATACATatacatatacaTATACGTCCAGGCTCAGACTGTTTTAGCCTCCTGGACGTGCAACAAGATCCATCTATAGATTTTGACGCAAAGTTCATACATTGATATTATATTATTATTWWTTTGTCATCATTGATATCATCGGCGCCTCGTTAATTTTGTAGGTATTAGCTAGGAAAACTGAACCGTCTTCCCCGCCAGCTGCACATCACATCATCTTTATCCTTCATGATGCCGTGATCTGTCCGTAGTTATTATCTCTTAGCTAATTAGCAAGCAATAGCCAGTGCTGATTTTTTTTTAAAATTTATTTTCATTTTATTTTATTAAAAGAAAGAAAAGAAAATAAAAGAAAAGAGAACCCGAGTGTGTGTCGGGGACACTTAGTCAGCAAAATTATGCAATT

>Cs1g06520

AATACTGTACTTATGTATTAACATATAATAAAAAATAAACAAAATTAATAAAATATGACTATTTGATGGTATGGATattgtaaaggacaataAATGAAGTatataaaattaTTTGTGAGAAAAGTTTAACATATTCTTACTTTAAAAAGTCCTCTCATGATTTTGATCTTTTAAAAATTTATTGACATGTGACGTTACTAAATTCGATGGCTAATTATCAGGAGAAGAAGAGTTgatgatgagagaaTAAATTGACAGTAGCAAAAATTATGGAGTGAAAGCGGGAAAAATCAGTCATGACGAAGAGTTTTAAGGAATTAAAATGCATCTTTTATAATTAAAGTTAGTTGGTGTAAGTGGTTCGACACTCTCACTCTTTaaaagaggttaagggtttaagttccgctttcgtatggagtcaccactttagccagcactttaccccttacgggttgacccggtgcaaacggggattagtctgggttgtattacggGcTtaaaggtgtcttccacagttggggcccaCTCAGaACCACCTCGTGGTTcagacaaaaaaaaaaatgcatcTTTTAAGAGGTTAGTCAAAATAATTTAGAAACAAATCCTGACCATTGAATTAATAATATCATACGTCAATTGATTTTAAAAGAAATCTCACAGTGGTCGGGTCATAATAAAAAAATCAACAAAAATAAAATAATAGTAAAACTTATTTACCTAATCACTTTWAAAATTTTTCTTTTCTATTTTTTTAGAAAATTATAGAAAGAtttttAAAAAATAAAAAGAAGAGAAATATTTTAGAACTTCTTAAAAAAatagaaaagaagaggataaagagttaactcttttttCCAgatttcaaaaaaaaccctaaTGAGTTCTGAGAAAGTCTCTCCATCGCCATTGGCGAGCGACTGTTTCACCAAAAAGGCAAAGTTTAGGGCTGAAGGGGATGATGGAGACAACCCTAcaccctttttcctaccGTGACATAGCAA

>Cs1g02760

TGTTATACTGAAGATTTTATTTGTAAGCAGATATAAGCCCTTTTTGAATCGTCGAAAAAATTGGAgcaactttttgttgggtgggtatttttgttacggaagaatgaaagtgGTTTGAAGAGTTATTGATGAGTAATGGGTGAGAGTGGATTTGAGAGTGAGGATAATGGCCATTGATGAAATGGCGAAATGGAGTGAGTTTAGTGAGACAAAGAGGGAGAAGGCGTGAGGGCTCGAAATGGATGGAGGAAAATTTGAGGAAGAGAAGAATGAAAAATGGATTGAAGGATAAATTTGTAATTTAAAAATGATATTtttatttnnnnnnnnnnnnnnnnnnnnnnnnnnnnnnnnnnnnnnnnnnnnnnnnnnnnnnnnnnnnnnnnnnnnnnnnnnnnnnnnnnnnnnnnnnnnnnnnnnnnnnnnnnnnnnnnnnnnnnnnnnnnnnnnnnnnnnnnnnnnnnnnnnnnnnnnnnnnnnnnnnnnnnnnnnnnnnnnnnnnnnnnnnnnnnnnnnnnnnnnnnnnnnnnnnnnnnnnnnnnnnnnnnnnnnnnnnnnnnnnnnnnnnnnnnnnnnnnnnnnnnnnnnnnnnnctttattggaaagagtaatgatacagtcacaaactcttgtataaacttattttgtacaaactgatgtggcattaatttattggttgaatgaaaatataaattaataaaaataaatcatgtgggccaagtgatatttaatttaaccaatcttatcatgccacatcagtttgtacaaaataaatttatacaagagtttgtggctgtatcattactctattggaAATGATATttttttataatgaacaaaagtttggagtcttacggaactaagtcgggGGGAAATTTTTAAAATAATAAAATTAACACGTGTCTATTTATAGAgcggcgactgactgagacactCGCGAGTGTCCGTAGCAATCCTCATATTTAATAGGCAGGCGCTGGAGACTAAAGAGCCCGCCCCAC

>Cs1g21840

GATTAGTCTATTTGTGGTGGGGTTGGGATTGGACGGGgagaacaaacaaggAAACGAAAAAAAAAAAAAAAAAAAGTAAATCACGTGTAAGCTGCAGGAAGATCCCATGTGTACAAAAGAGGAATACAAGATATTCCATGCATATACATATGGACCTGTTCCAAGGATTGATTTGCATGTGTCCTTCACATGTAGAAATGTGTATATAAATCCGATAGCTTGTATTGTTGTCCCTCTTTTCAAATATCCAATATATGATTATCCAAAATGATCCTGGATAAGATATATAATACAAATAACTTTGTAGAACTAGAGAACGTTGAGAACTTTATATTCATTTATTAGAGATCAGATTATTGGGAAGATGGAAGCAGAACGAAATTGTGATAACATTTCATGATCAGCAAGCAAACTCATGGTTTCTAGTTTTGAATGAAGCTTAGTATGAAATTTTAAGTAACTGAAAGTAAAAAGTTTGGAGTTCAAATCTTGTTCATATAGATGAAGAAATTTAAGAAAAAAATTGGATAACTTAAaaaaataacaatTAGAAAGCCCTAGCTTCTAGGGTCAGTGTTACAAATAATTATTAATACATGCCGTGATAGAAATGAAGATTTCAACGAATTATTCTTCCATTGTCTTGGATAATGACCGTCCATTTGTCTTATGGAAGCATGAATGTAGAGATTTTGGCTTTCATATATGAGTCATTTGCGGGGCCCTTACTCTCATTTCTTTGTCGCCATGAATTATCATATATATTCTGTAGTGATTTCAATACCCCACCGAGACGGAAAACTTTAGTtataaggtttaaaacATTAGAGAAATGGGCATAAAGATGATCTGTTCATGATATAATAGATGATGGCCCGTGAGTAGAGTGAGCCAAAAAGCTCAACTGCCTTCAAGGTTCAATCCCCACTACTCAATACATGACAAAATTCCAACGCCCACATATRGACAACTCAATGGCCGGCTATTTCACATGCAACCAA

>Cs1g06240

RCAAACTAGAGTTGAATAAATGAAATTTACACGTGATTTGAATTATTCGAGTTGAATTAAATTGTGATACACATGGATAACTAATTCAAATTGAATAAgaaaagtgaatgATTAATTCAAATTGAATGAGAAAAAGGTCTGTATAAGAGAGACCTATAAAATCTTTAGTAATGTCTACTAYGACAATAAAAAGAGAAGAGAACAATTCGATTATTTTAATAAATGCTCTCTACTTYTRTCTTGTATTTTCCTTCATYGATTTTTCTTTTTGTTGTTTAAACCRTCAARTTTGGAGTGCACTTTTTATAGTAATCGTCGTTGAATCTTGAAGANCAGRTCGAAAAATCTTCCTTTGAGGTGTGAAAATTTATCTTAAGAATGTGACTTGACAAATATATAATTaatgtgtattgggtaggataagcaGTAccAATAAACGGTGGTGTAAACATTACCAACACTAACTATTCATGAAATTTTATTCKCCACGTTCTATGTTTGTCTCGGTCTATTATTAATCAATGTTTATTGATTAATGTTGTTGTTTTGGTTGGTTTAGGAGTTTWAAGAAACAAWATTGCcttataaatatgagTCCTCRTCTTTATTCATCTTTCATCGATCCTCATATTYAGAAAGTTAATTTTTATTTGACAAATCACGGAATCCGTGTAAATATATTGAAGYTGGCTTAYGCAAGTGGATTTTAGTGGAGGCTCaactcgattcaCCARGGACcctaaagagagcaaaacCAAGCCCAGCGAGAGCTGGACACTAGAGCAGACATCRGGGCCCTTTTGTAATTTTGTCTCAAAGTTTTTYATTGGGCTAGACCGATTCGGCCCAAMCTTTTGATTTTTMTTTTTAAACTATTTCGGCCCTGCTCACATATTCTTTGACGCGTAAAAGAGAAAAAATAACACATGGACCACTGCTTGCCAACACAAGGAAGCAAAAAGCCTCACGATTTTCAACAAATTAAGTAAATCAGAAGAAAC

>Cs1g09680

gatatggatatgggtattttaaaaaaatatccaaataaaaattggatgggtatggatatccaattaatatcttatttgaaattaaaaaattaaaatcgtaaaactaaaatgaaataagcaaaatcaaatgtctggatagcaaaacctaagattatcacatcgctcgctggccggtcttcgcttaccgacgctcttccccgactccgacgctgctttccgggggttccgtttaggtgcgccggcgctgtaccaatggttgatcagccgtaactgtagcttccagctattcttgagcgcttttctgcctcgttccagctgccatcgtctctgtcgtcgctaccaccgccgtcgnnnnnnnnnnnnnnnnnnnnnnnnnnnnnnnnnnnnnnnnnnnnnnnnnnnnnnnnnnnnnnnnnnnnnnnagtgggagttggcaagagtagtatgtgagcgtttgaaacccttctatactatgactgagatgttttctggcactaaatacccaacagctaacttattttttccaataatttgtgaaattagattatcattgaatgcttggcttaattcttcatgtgatgtgataaaaaaatatggcgaaaagtatgttagaaaagtttggaaaatattgggatgaaatccatggtgtaatagctgttgctgttgtattggatcctagatataaaatggttctagtggattatttctttcctcagatttatggtagtgatgcatcaactcatattgatagaatCCGTACTCTCTGTTCTGACTTATATTCAGAATATAAAAAAAAGAGTGTGGTTGGATCGAATTTGGCTGAAGGATTTGGTGAGTCTAGTGTTGTTTGTAATTCTAATTCTAGTtcagttgTGatgggtatgtgggatgtgcaaaaatttaatgcattcaaagccaacatattggtaaacgagttaagtcggagttagataattacttggaagaagaggataagactactccagacttcgatattttaa

>Cs1g20110

CCCCGCGGGGTACTTATTGCACTCCCGctaattaaacgaaaAATTATGTATAAGAGACCTGTTGGTGGTAAAATATGGAGAAAGAAGTATTGAGCGCATGACACGTTGGGAATATGATTCATGACTTTTGAAGCCCTCCAAAGGATGAAGGGAAAGTTGAATAGGCCACAGTAGAATGGGAAGATCTCGAAAAATTAGTATCAACCACCTAAATCATCTAATAATAATAAAAAATATCTATTATAATATATCTAAGTTGCCACGTGTCAATatgggaccacataacagagacaatctcaccaagagcagggcatgtcattacagcacagtctCCACCTGAATTATCTCTAAAATATCTCCTTGCAAACCACCAAAATCATATTTCACAAAGAGGACCACATATACAACTACATACATAATRTCTAATGAAATTAGGTGCTCCTTAGCCCAATTGGTTCATTTCAAATGAGCCAATTAGAATTAAAGATATATTACCAACAAAAACTATCCCCCTAGAAGACAACTCTCTAACCTAAGCAAGCTCAAAAGCCTCAACAAAAAGGCCAAATTACTCTTAAATCCCTAAGGAAGAAAATAACTAATGGTGGTTTGccatgctatctaAAAGAACATAAAGAGGTCCTCAGGGGAGCATTCCATCTTCAAAAGTCAGGTTGATTTCACAAAAATATTAAGTTTGCTCGTTCACAAAAATACTCAACTTTTCTCCTATTCTAGCGTTTCTTGGAAAACCTCGCCTCTTTCAACCTCCCACTGACTTGACCGTCAGAGTTTCATCATCGGCCACTGACGTCATTGCCTTTAACGGCTATTCCTTATGATTTCAGGTTCATCTTTGTCGCAATGTTCCCCAAAAAGACTGTTTCTAGGCACCTCCATCTCCATTACTGTCAGAAATCACCTTCTGTAGAGAATCTCTCATAATGCTTTGTGCTAACTAGAATTGGACCAAAACAAGACCCAAAAAAAGACGAAGAAGA

>Cs1g05380

TTTAGTaatttatttctcttAGCAGTTTAACAGTAAAATTTACCAAATGTTCATAATTATTTTTAAAACTCACAGTACTTCTGAAAATAAAATTTACCAAATATTCAACTGATTCTCTTCACAGCTGATTATTTATACAGTACAGCTAACAACAATTATTTTAAAAGCTACAATATTCCCAAACTGGCCCTAAGATCGATGTTCAAGTTTTTGTATCTTTGAATATGTGTTGTGGCAATAGATTTTCTTCTTTATAATGTGTATATTTTCAAATTTACCAGCTTCTACTTGGATGCATCATAATAGTGAGATTCAGAGAGATTTATAATGCATCGATGAGTTATATTGCCCAATACTTCTGCATGAACCAATGATATTCTCGCAACTACTTTAGAATCATTTGCTTATTCTATAATTGGAGAAATTCTAGAATATATCAGAGATAACATGTCAGCCATTCAACAAACAAATGATGCTATGACATATATACATTTATTTTGAAAAATCATCATACAAGTGATGATTGTATTAAATTCAATTATACATGTCATGCATGCATCAATTGGTTGTCGTAAATTAGAAGTACTCTATAATTTCTCCTATAACTGATATGGTACTTCAGACGTATTCTAAAATTTCTCAGCAACTTGATTGCCGTCAATAGATGCATTTGCTATTGGCTTCGTTCTACCCCACGCAAACATCTATCAATGAAGAAAAATCTACATTTTTATGCTTTAACAACCCTCGATAGGAAATTGAAACAATCATCCACACGTTTTGAGAGAAGAGCCTCAAAGGTCAAACATGCTAAATTTCATTTCCATTACCTACCTTGCCACTTCAGTTTGGTGCTTCGTCTTGTTTTTGCTTAATTCTCATAGTTGTTTTGGCCTTCATTCAAACGAGACAGACCGCCTTGCTTATCTAGCCATAAAGTCACAGCTTCAGGATCCACTTGGGGTTACAAAGTCATGGAATAATTCTATAAGCTTGTGCCA

>Cs1g06770

TGATTTAAGTCTCGACTCGATCTGTGCCATCCTCYCTTAAAAGAAAAAMTTTTGYGATGCTCCTATAGCAATATTCTTTTTCCYTTCACTTGCCTACCTGAAACTTTATCACCTTAGGGCAACTCTTAGTTCTACCTTTACACTTTTTAGTTCTACTTTAAATTGCCCCATTGCTTTCTTTACATCTTCGCCAACACTAATATGTGTAAAAAAAAAAAAATAcaaactataaaaaaaAAACTATGAAAAAGTTTCACTACATGACAAAATTTCTTTCATTTTTGTGCCTTTTGCATATTTTCAATATATARAACATTATTCCATCTTCACAGGACATTTTGTTCAATCTCRTCTTTYGAAAACTGTCGACTTCCCATCMGTCCAACCATTTCTCCAACCAGTGTGTAATAAAATTGAKCTAATATTTGCCAAGATGCTSTCARAATCCTATATTTAGTTGCATTTATTgtactccttgatcaGATAAATCGTAAATTTATTATGATAGATTTCATCATAGCATGTTTAACATACAYTTTCATAATTGAAAGTAAGAAAAATGTTGAGAGTGATTGGATGCTGTAAAGACAGAAAGAGACCTTTTTCTCTTTTATCTGATCAGCACTCAACTTTGCGTGACTCTCGAAGAGACAAATATAGTAGCCCAGTAACACACCTCTATTAGGAATTTTAGTATTAAAGATAAtatcactaattaatgcatgtaattaaatnnnnnnnnnnnnnnnnnnnnnnnnnnnnnntaaaaatatataataataataatatcatttatttatcgtatgacagacatgATACTTTTTAATGTATTGTAGAATTCTCCTCCATTATTTAGCCTTTTTCCTGCTGTTCTTTTACAAGTGGKGAACGCRAAATSACTYGCTTTCRTCACTAAAGTTATAAAACAACAAAAACATTAAAGTGGCTTTGGAACTGGTACATTCAATAGCACTACAGAGAAAWTTCTTCTGCTGTTTYAC

>Cs1g07500

cattgtctttaataaaatctcatttccaacccgagagagcatcctcgtactccaactctgaagcctttgccagagcctgtcccgaatatgcctgaaaacttctttcttttttctgccaatatatgatggaagaccaaggtaggctccatgattagtggtnnnnnnnnnnnnnnnnnnnnnnnnnnnnnnnnnnnnnnnnnnnnnnnnnnnnnnnnnnnnnnnnnnnnnnnnnnnnnnnnnnnnnnnnnnnnnnnnnnnnnnnnnnnnnnnnnnnnnnnnnnnnnnnnnnnnnnnnnnnnnnnnnnnnnnnnnnnnnnnnnnnnnnnnnnnnnnnctatcatcggcaaagaacaagtgagacaccgatggagcacctctcacaattctaacaccatgaagcaagccattcctttcttgccttcgaattaaagagcttaatccttctgcacaaataatgaataaatacagagataatgggtctccttgacgaagaaccctacaaggaacnnnnnnnnnnnnnnnnnnnnnnnnnnnnnnnnnnnnnnnnnnnnnnnnnnnnnnnnnnnnnnnnnnnnnnnnnnnnnnnnnnnnnnnnnnnnnnnnnnnnnnnnnnnnnnnnnnnnnnnnnnnnnnnnnnnnnnnnnnnnnnnnnnnnnnnnnnnnnnnnnnnnnnnnnnnnnnnnnnnnnnnnnnnnnnnnnnnnnnnnnnnnnnnnnnnnnnnnnnnnnnnnnnnnnnnnnnnnnnnnnnnnnnnnnnnnnnnnnnnnnnnnnnnnnnnnnnnnnnnnnnnnnnnnnnnnnnnnnnnnnnnnnnnnnnnnnnnnnnnnnnnnnnnnnnnnnnnnnnnnnnnnnnnnnnnnnnnnnnnnnnnnnnnnnnnnnnnnnnactcaactGGATATTCTTTTTCGTAGGCATTTTTATCTTTTTAATTTCACCTTCTtgtggtatatgcccatATAAATTACAAAAAGTCCAGTTTACCCTTAAAGACACGTCAAGGA

>Cs1g09770

ggctaaccagttagagcgtaagcaatttttcctctctggattttatcgattaactgtttcaaagaaaacgatagaccgtttatttccagaatcgcttagctagccaaatattgcagggaatcatttttcaatttgagtttaataattatccatttttcatgaatataattggaatgattataaggcttatgtttattaatgaatagcacaacattttaatcaaagtcatttaaagtttcttatcatcaaaatcaatattatgaacatatacaagttaacattctccccctttttaatgatgacaaacttctttcaatattttgctcccccttaatatatgctccccctaaatgtatggccaatttaaaaattgttgaataaacagatttttacttaataaagtgtttaaggagatttatttcaaatttaagttttatacttattattctctacctcccccttctttattttatannnnnnnnnnnnnnnnnnnnnnnnnnnnnnnnnnnnnnnnnnnnnnnnnnnnnnnnnnnnnnnnnnnnatttaaggaagaaattaccctgattaggcgagcaaatttttttggcaaagttccccttaaaatggaacaaatcctcaaatacaaaacaagattaaaacaattccaaatataagaaaaaacaaatttctatatatcaacaactccatcaagcatcagtccacacacatagaatcatccaatatcaaaagcatcacatataaagaattcatcatcaaaatcaaatannnnnnnnnnnnnnnnnnnnnnnnnnnnnnnnnnnnnnnnnnnnnnnnnnnnnnnnnnnnnnnnnnnnnnaagatgaagaatgaaactaagatggaggtggtgaagatgaactgccagggtcatagccaaaataacgaaggagtgtctgttgtccagcaataagctcaagccgctgatcaatgctctgctactgaaaagtctgaaactgnnnnnnnnnnnnnnnnnnnnnnnnnn

>Cs1g09140

attttaacannnnnnnnnnnnnnnnnnnnnnnnnnnnnnnnnnnnnnnnnnnnnnnnnnnnnnnnnnnnnnnnnnnnnnnnnnnnnnnnnnnnnnnnnnnnnnnnnnnnnnnnnnnnnnnnnnnnnnnnnnnnnnnnnnnnnnnnnnnnnnnnnnnnnnnnnnnnnnnnnnnnnnnnnnnnnnnnnnnnnnnnnnnnnnnnnnnnnnnnnnnnnnnnnnnnnnnnnnnnnnnnnnnnnnnnnnnnnnnnnnnnnnnnnnnnnnnnnnnnnnnnnnnnnnnnnnnnnnnnnnnnnnnnnnnnnnnnnnnnnnnnnnnnnnnnnnnnnnnnnnnnnnnnnnnnnnnnnnnnnnnnnnnnnnnnnnnnnnnnnnnnnnnnnnnnnnnnnnnnnnnnnnnnnnnnnnnnnnnccttcttgagaatattaaaaaaaaagaaaataatattaatttatcaaattagcattaaaataacataaaaatttatggaaatttttatgttattattttacttttaggattaattttggtaaaattattttttatacaataactattagtaaaATTGTTTCAAGGATATTAAACATTACTTTAAGTTAGTATATAAATTATTAGCAAGATCGTCATACGGGTATAAAAAATAGTAGTTCTTTCGTATTGATATTTAttggttaatttgctaactaaaatttttgtagataaattaattacggccaagaatattannnnnnnnnnnnnnnnnnnnnnnnnnnnnnnnnnnnnnnnnnnnnnnnnnnnnnnnnnnnnnnnnnnnnnnnnnnnnnnnnnnnnnnnaatagagtgaaaatatagagaataaatacaaattgttgaatgaaattttatgttgttgaatgaaaatttttcttgaaccacgtataagttaatcaagttacgccaaggcacttgtacAAAAATAATTTACAGAAGAATTTGTAGGTAGTATTACACTAAACACANAACAAAAATGTTGAGGCGAGATTACT

>Cs1g05440

AGATATACAAGCCATTTTAAAATAAAACTGTAAAAAATAATTAAATAATATTTGACTttttgcataaatttaaaatttaagaannnnnnnnnnnnnnnnnnnnnnnnnnactGAATAATATATATAATATATAACTAGGACAAAAAGAAACATAGATTTCAGAGGCattagattttttttttttatcttTTAGCTTGTTTGGTCAAGATAAATAATTAAATAAATAATCAATCAGTACTATTGTTTTATCAAAAGTAAARWCAYCCTCTTTCATTTACTTAAAAAMAAACACAACAAAGATATAAGAATACAAATTTAATCTATTACAGAATAAAAAACATTGTATCAAAGRGTGCGTCTTTTTATCTTTTAACTTGTTCCTNACTTTAGTATAGTCAATGATTCTataccaTTTTCATTCTATTTAATCCAAGATCGTTCGGTATTTAAAGTGATGGGAGTCTTTAAAGTTAAAATTYTWATGAAAAAAATAATTGTAAAATAAAAGTTAATTGTGTTTGGTAAATTTAGGTACCATAAAATATGATCACTTACACCACATRgtatgtagtaaGGTTGGCAAAATCYGATTTAGATATGGATGTAAGTGCATTCGGGTCCGGATATGCATTTAcatttctatatccnnnnnnnnnnnnnnnnnnnnnnnnnnnnnnnnnnnnnnnnnnnnnnnnnnnnnnnnnnnnnnnnnnnnnnnnnnnnnnnnnnnnnnnnnnnnnnnnnnnnnnnnnnnnnnnnnnnnnnnnnnnattcgaatgcgggtaatatccacaacggatgcgggcggatattccggatctgtaaaaataaaaatcaaataaattaaaaattcaacaattcaannnnnnnnnnnnnnnnnnnnnnnnnnnnnnnnnnnnnnnnnncaatTAAACATAAATAACATACAAGAACATCATAAATCCACAAATAAAGATYTCACAACATAAATTTGAAAATRTGAAATTGATTTGTTGGAAACT

>Cs1g02110

gaaatatttgcactatgattgtcaaccttaaacagctcattgtgatctcaaacccaacaatgttcttcttgatgctgacttgactgcacatttaggtgacttcggcttagcaagattccttccatccactcataagaaaacgagcactattggtatcaaaggatctattggctatattgctcCAGGTATTAGCTTTTACATTTATCTTTTTTGACACGTAAATTTTCATGATACTTAATATAAATTGATTGAATTGATTGTTTGGATGGCATCAGAGTATGGCTTWGGGAGTGAAGTGTCAGCATATGGAGATGTTTACAGCTATGGGATACTACTGTTAGAAATGGTGACAAGTAAAAGACCCACAAATGTTATGTTTGAGGGAGACTTGAATCTTCATAACTTTGCGAAAACAGCCTTGCCTGATCATGTGATAGATATTGTGGATgcagtaattctaaacgatgtcgaagaattgactgctactaaccaaaatcaaagacaagcaagaatcaacagcagaattgaatgtctCAAATCTATGGTTGGAATTGGAGTTGCATGTTCAATGGAGTTGCCACAAGATCGAATGAACATAACAAATGTTGTCCACGAATTGCAATCAGTCAAAAAAattcttcttggacattgaactaTATATGTTCAATAGGCAAAGAGGTAATTGATAGTTCTTTGTCTAATCATATGGAacaaattcaattgtacattttggcttttgtaataaagaaggtttggtgatcacactgaacagtgccgaaaaatcgtggtggtgagaatatttcgtggatttttttttcaaataatatTaactgtgttCAAGGGgcatgtgaagagaCTAGCATCATCGCCACTATAAACACTCCCAAATGAAAGCAAAAGCTTTATAATATTTTAAGAGAAAGAAAAACATTAGCACATCAACTTTATCAATATTTTATAATTTATGCTGCGATAAATAAGGAAAAGAAAAGAACAAATTA

>Cs1g03810

AACTACAAAATGACATAGGCTTTATTCTTGAGTGGGGCAAATCACATGCATGTAGCCGTATGTTCACCATCTTSGAATTGGGGATATAGTTATTATTTTTGTATTCMCTCCAAGTGTTTGATAAAAAGCCTGAATCAATCTTGGCRCTTTAATTATTGCAGGCCATTTCTACMTATGAGGAGCCAAGGAATATGAAGCTTATTATATTCATTCGGAAAGCAAAGCTTAATGAGGTGTTGTATTTAATCTTGCTTATGCTTAGTCTAGGTGTACAATTCATCTGTCACATATTGTTCAATTTATGTTGACATAATGTTGCTTTGCTTTTTGAAGGACTGGGTTGTGTAAACTTACAGGAGATTCAAGGATTGTGAGAATCTaaatctatattttatattttatatgaatttGGCTTATTGAAATTATATTCCAATTGAATAATCAATGAATAATGAAATTTWCTTTTGTSATTTTAAAAGATAGATGGAGTAAAATTATSTCAAAGGCTTACACATATTTCTTCCAAGATACAATTTGGAACTTTGTGCTTTGAAGATGAATTGTAAACTTTMTTRTAATTTCAAGAATTCAACAGGCTTCattgcttttttatctacAAATGATGCACTTGAGCACTAGTTACAGAATAGTAATTAGCAGTWTATCTTGATCTTTGGCAGTATCTACAAGGCTGATGCTAGTGAAGCTCCAATAATATTACCGCCTTAAAATATTACCATTKTCACKTTTGATTTTTTCAGGATCTGGGCTCTRTGCAGGAGAGTATGTTACTCTAGGATGGAATAAAGTAAGYGGTTGGTTAACTAGTGGGAAAAAATTACRCATCTAGGAATTTTTTTTWAAATTTTAATTTTTAAAGGGAAAAAATCATTTATGAGATCAATTATCTATCTTGTTGACCTTTATTCTTGTAATTAAGGTCCATCTGAAGTTGATTGCTTGTTTTTCAGAAAGATGATCTGAAGGCTGTGATTGATTAC

>Cs1g11750

TAAGCTTCAGAGCAAATATTGTTGGGTGTATTTAGTAATATGATTTCCCAAAATTCACACCTTTCTTGGATCAACAAGTTTGTGGGCCGAACCCAACCATTGTAAACCTTTCATAGTTTTTWAttttattttttttaaagtaAACCTTACGTTGGATTTGTAATATACATTTGTTTTAGTAAAAAAATTATTAAAAGAAAGCACCCTTRTACACAGACACAGAATACTTGAGTCATCCACTTTAAGAACTAAGAGATGAGGTGTGCACCACTTAATTAAAGAACAATTTGTAATTTATTAAACGTATATAACCTTTATTTATATTGTATGAAAGTAAATGCATTTAACAATAAGAAtgaccttaagacctctcTATCTATTTTAATGTTAGACAGTGGGTGTACACTAAAAAAATTAGTTAGAGTAAAATATTATTATTTTTTTAGAAAATTATCAATCCTTTATCCTAAGAATATCCCGTTATAAAAAATACTATAAAAACTTGAATTTATTCGATTTTACATTCATTATTTATATTTTGTATTAATTGTCCACTCTACCATTGGGAATTTATAGTCATTCACTAGATTaagacttttttaaactaTAGATATATCGTTTGCCACCATATATGGTTGATAACGGGATAATTTCAAAGTTGACGCAaataatttcttaaaAAAAGGGAAAATTATTaatcttgtttttttTTCTAAATATTGACAACAAATATAGATTAAAAGAATGTTGGAAAAGAATATATTCTAATAAATAATGGAGAAATCTACTTACATTTCTATTATTAGTGGAGAGGCCACTCTCACATGAAAGACGGGAGAATAAAAAGAGGAGTGTAATTGTTAACTATCTAAATATTTAATTAGTGTAAATTATTGATTAAGTACTTTTTGTTCACGTAATGTTATTTGTTTTGGTTTGCTTAAACTGTTATAATTATTGAAAAATTAATWAGTTATTCAAGAAATCTTAATCCGGTGCAT

>Cs1g07210

AGTCTCCCTTAAAAATTAGAACCAATTCCATACTTCAGATACCGCCACGTGTCCTCTAAAACCCTAAATCCTTCCTATATGAAACCCCTCACAATTGGCTCCCAAAAATATTAACAGAGAACCAAAACGACTAAAACTTCCAATCAATCCACATGGTCAAGTGTCAACTGGGCAACCAGAATATTCCGCAAAAACGGCATGTCGTTTCCGTAGCTTATCCGCAGCCGACTTGTTGTGCATCCACGTAATGAATAAATCCTATTGGTAAATTGCTCTTTATGTATGTGTATGCTTAAATTTAAGTAACCAAATTCTTCAAAATATCTCCGCATAGCAAAAATCAttttttgtttaaATTTCACTCATATATTTCAATTAGCACCCAATACAAGTCAAACAATAAGATATTCTAAAAAATTTGACTAATAAAATTAAGGGTGCATTTAAGAAcaaagtattctaacttttaaaatatagatgttggagagtaaaatgcatgattaagaagtagaattgaagaaaaaaaaaactataaaacttccaaaatcccattataaggnnnnnnnnnnnnnnnnnnnnnnnnnnnnnnnnnnnnnnnnnnnnnnnnnntgacttcCAAATGCACCTTAATAGCACTCAAaatttaaaaaaAAaAGATATGGAATTAATCATAATTCTTAAAATTAATTGCCATGTTTTCTTTTAAGGATACATTTGTTGCCACTTCACTATGATTAATAAACTACAGTTATTTGAATTAGAATCTTCTCTTAAATCGAATTCTCTATTTTAAAAATTCTATATCATGTATATTCTTgtgataatatataaataaAatttagttttttttttattaaCCCTTTGAGCCTTAACAAACACTGTTGACAACTTCCGTAACTCACGTGGCACATACTTACGGAGTCTCTAGAACTAACTCCACATTTCTGGTACAGCCACGTGTCCTCTAAAACCCTAAACCCTTCCTATATCATAACCCCCCC

>Cs1g04750

nnnnnnnnnnnnnnnnnnnnnnnnnnnnnnnnnnnnnnnnnnnnnnnnnnnnnnnnnnnnnnnnnnnnnnnnnnnnnnnnnnnnnnnnnnnnnnnnnnnnnnnnnnnnnnnnnnnnnnnnnnnnnnnnnnnnnnnnnnnnnnnnnnnnnnnnnnnnnnnnnnnnnnnnnnnnnnnnnnnnnnnnnnnnnnnnnnnnnnnnnnnnnnnnnnnnnnnnnnnnnnnnnnnnnnnnnnnnnnnnnnnnnnnnnnnnnnnnnnnnnnnnnnnnnnnnnnnnnnnnnnnnnnnnnnnnnnnnnnnnnnnnnnnnnnnnnnnnnnnnnnnnnnnnnnnnnnnnnnnnnnnnnnnnnnnnnnnnnnnnnnnnnnnnnnnnnnnnnnnnnnnnnnnnnnnnnnnnnnnnnnnnnnnnnnnnnnnnnnnnnnnnnnnnnnnnnnnnnnnnnnnnnnnnnnnnnnnnnnnnnnnnnnnnnnnnnnnnnnnnnnnnnnnnnnnnnnnnnnnnnnnnnnnnnnnnnnnnnnnnnnnnnnnnnnnnnnnnnnnnnnnnnnnnnnnnnnnnnnnnnnnnnnnnnnnnnnnnnnnnnnnnnnnnnnnnnnnnnnnnnnnnnnnnnnnnnnnnnnnnnnnnnnnnnnnnnnnnnnnnnnnnnnnnnnnnnnnnnnnncccttaagatttgggaaacctcgaccacatatacagagacctatcctcttatgcttttaattttaaaaagaaaaaaaaaacgcacacagccaaannnnnnnnnnnnnnnnnnnnnnnnnnnnnnnnnnnnnnntgagaaaataaCAGAAATCACGCTTAATGAGGAACAAAAATAATTATGAATTAAACTATTCCAACCAAACAAATAGGACAAGGCAGATACGTATATAATAATGCAGGCATAATTAAGTTAACAAGACAGATAGAGGAATCAAAATCACAGATCCTGAGCTTTTCAGTATGAGCTTACAACAGAAGCAAAACATTTTTTACATGTTATT

>Cs1g22960

ATCAAGTTTGTTCAGCTGCAACTGGATACACATTTCTTGTTTAATAACGAACCTCTTAAccagttgcacaaGTTTGATCACTGAACCAAGCAGTGCTTATACACGCAGCAATCCAACTTGAGGATGCGACAYCTTAAGTGACAGATTGGAATGTGCATGCCAGCGAATTGTAACCACGGAACATATRGACACATTTACATTAATGCACGTAAATAATACATGTTCAGATGCAAGTAAAAATCCTCTTTTGAGTTGACTGTTGAGCCAAAGTCATTATTGGTCTATAATAATTAATGACGCTAGTAATTCAACGCTGTTATAAAAATATATATAAAAGAAGAAAGTGAAAAATGAACTGCATTAATTTGCTGTGTTGATTGCCTCTTTAATCGTCAATATATCCATCACTTTTCTTGCAGGGCACTGGCTTATTGTATCGAAGTCTTCGAAGGATCAATTTCACAAGGCACTTTCGGCATATAAAGAAATCATCGCTGCCTAGAACTTATCTCAATCTTTGAACTATCTGACAAGAAATGGGCGAGGGAAGCTTCTCTCAAgtgcagtttttctaagATTTAAGTCAAACTTGAGAGTATAATTACATTGTCCATTGTTGTTAATTTCTCAAGAGGAATACATCATCAAGCCACTCACWGCTATTTTAATTTTTCATCAAGTGGGGTTAGCTGAATgcaaaaacaaaaacccaacaaattaacaatagattcaagcttcccattacattacaaaacaattttgcataccaatttcttagaaatggaaacgctaaaacccaaatcanatctaAAAAAAATGGAATTGTACCGAACTCACTGAGAGTGACATCTTTAGTGGCTCTATCACTCTCCAATTTTAGAAAAAAGAAAAAAAAAAGAAATTAATCAAAGTTYGCAGGTAGACTATCTTCATTTCAGTCTATAAATTGCCTTAAAACACAAGTGAAGTGGTGAGCAAGCTAACCTATTTGTTTTAAAGAT

>Cs1g13070

TGCACATTATATTTTCTTGACTAATGCTTAACATTTGATTCTAAACCACAAAATCATTGTGTGAAGTAGTCCACGTTTCGCCCAATCTTAGCCCTTATCATCAGAATTGCTCTCCTTCGGCCCCATACCTCAGGTTCAAATCATGGGCAATTGAGCGACAAACATGATAACGCATTGTAAACTTTTAGTGGCTTGAAGGAACATGGGATGTTTCAAAATGAACAGTAATCTCCACATCTCCGTTTAAAAAGTTTTTCTACATCTCCAATTGTATTTCACACAAAAGAGTGATTTTCACCGCATATGACTTAATGCAATGTAAATGTCATTGATATAATTCAATAGTTTTCATGTCAAAATAAGTGTTAGCAAATGCAAAGACATTTAAAATCACTAATTGCATTAAGAATATTTTGAAAAAAAAAAATCTGTGAAGGTCTAGAAGTTCAGATCAATTTACATAACATGAGAATTCATACTCTAGAGATCTAGATTTTTAGAAATCTGAAAAATGTTCCGAAAACAATCTAGCACCAAACTCAATTATTAAGAATCAACATCATTAATCTCATCTAaacacgacatgttacatgttacatgtacataAAAGTATATTTCATCATATTTTACCTAAAAGTCTGACTAAATCAAATACACTTAACATTTTTTTAATCCATATAAACATATAAAAAGACCTAAACTCATTTATTGAAGACAACAAAATTTAATTTTCAAAAATTATTAGCCTGGAGAACGGAGATGATAATTTGAAAATGGAAAGGCTGGGCTTTTTCTTTGGTTCGGGCCATAACCAGCCTTGGCCGACACCGGACCCGGCCCGACCCATTCCCATCCCATCACACATTAATGGAACAAATGTCCAAGTGTTTTATGAACTTTGTTGAGGTAGTGCTCTTGTCATATCATTTGTWCTGTAGGCTCGgcctgattggaacgacTACCAAACGAGTACCAGCTGGAAGGCTGATTGGAAGGAAATT

>Cs1g10840

ttatttatttcattatatgttattgctcttgtattaacttattaatcnnnnnnnnnnnnnnnnnnnnnnnnnnnnnnnnnnnnnnnnnnnnnnnnnnnnnnnnnnnnnnnnnnnnnnnnnnnnnnnnnnnnnnnnnnnnnnnnnnnnnnnnnnnnnnnnnnnnnnnnnnnnnnnnnnnnnnnnnnnnnnnnnnnnnnnnnnnnnnnnnnnnnnnnnnnttatggtgttccagttggaaagggaagaaatgatcttaggagttacattggagtcattatacgtgaaacaatttcaattttacttgatgattggaggcgtgnnnnnnnnnnnnnnnnnnnnnnnnnnnnnnnnnnnnnnnnnnnnnnnnnnnnnnnnnnnnnnnnntgtttaaaaattttatataacttctagatttgttgaaagctgtttctaatccatgttattagtttacagaaaaaattcaaattgagcttgaaatgcaaaagccaagtgttaaagtggatgaagattgcatcaagaaactttcgaagtgaactggcagctgaattcgttctacctaacaaggacaatcgaaagtcactaaggttgcctcctattgaatatccgagcattaaaaaggaatattggaaacttnnnnnnnnnnnnnnnnnnnntgaacaattttaggtgtgttgtttgttaatattacctgatttatttgttgctagttactaatagtcagtggtgaccttttgttcttcgtcgtactgtttgtgcaggaaaaaagtaagaaggcaaaagtcaaaagagcaaagaatgtctacaaccatcgcttgggtagcacaagatatggtggcatgttgtatagaaaagtatgtgtagtaaataatctaatatgtgtagtaagtaatctaataCATATaatataatagaatcaattatttatgttttatgtcaccttgtaacagaaaaatgagagtggagtttctgagcgggagattgaccgcagtgaagcttggtta

>Cs1g17680

CATACTTTGTTGAACTTTAAACTGGTGCAATAACTTGAATAATCTTCTCGTGTCCTGAAAATCAATGTGTTAAATGGTTTCTTTCTATGGTAATGTAGATGGGTTCCCGGAAACATGCATCTTCAGTGCCTATTCCCATGCCGGTACTGCTTCGAGTGAGAGAGCTTGCGGAAATGCAGTCACAGTTTCCACCAAATCTGAGCTCAAGCAAGCTTACTGGATCTGCTGGAAGAAATGTAAATGAAACAAGATCGGGGTACTTGTAACACTGTAAATAAAATAATACATACCCAGTTGCCTAGAGTACTAAAATAAAGTTTGCTAATTGCTAGTAGCTTTGTTGGTTGAACATTAGTTATTCTGACCTAAATTTAGCCCTTTAAAAAATTATGGTCTTTTAGATTTAATTTTATGGTGCCGCAATCCCCCTGGGATTAAGTAGCTGGCTGCTTAAGTGATATCTTTACCGAAGATCTTTGTTAACCTAGATTTGTATTATTGGAACGAGAATTGTACAATTCATCTTGTTGCAAGTCTCCTGTTGATGTTCCTTTTTTTCTTTTCCTTTCAGCAGTCTGGAGTCTCTCGGTTTCTTGAAGTCTAGAAAGGTTTTTTATATCGTAATTTGTATTTGCGAAGGGTGTGAAAGGACGTTCAAATGGAGTAGAGGCATGCGTACTCCGTAACGTCAATAATCTAATTGTTGGACTCGGAGCAGATCAGAAATTTGTCGTTTTTCAATTAAATATGAAGAAAATAAAACAAATTGATTGTGCGATAATTAATTCACTACTCGAATCCCCTGTAATATAAATTTTCTTATTTCTTTAAAATGGAAAAAAGAAAAAACTGTATACGAGTCTGAGCTGGGCTTGAAATATTTAGAATCTGGGCCCCTAAAACCCAATAAATACGGcctgtaaaattagccCAATCAAAAGGGCTTACAAAATTAGCCCAAAGGCCCTCTTTTTCAGCCCTTTTCATGTCACCCGCTTCGC

>Cs1g07600

TTAAAatcaatttttttaaaattGTTTATTCATTTTCAATTAAATTAAAGGAAAGGAGACTCCAATCTCCGTAGTGACATTTTtttataaacaaaaaaagatatttctcccttttttttctaattaacaaggaacGttcaaccttattTTCATtCCGAATAAGATAAATTATATATATATTTAATAATTTATTTAAAAGAAAACGTGATAAAACTGTTTTTTCTtattttttttcaaaaaAGATGAAGAATAAAAAAAGGTGGGTTTGGTGAGTTCACatgaactaatatTACATTCTCTTAATCTACGCCATTTGGCATTTGCCCGTGACGTCCGCCTACCGCCGCTCRCTCCCACCGGTTATAGCCGGTCATCCCTTTGACCTTTCATGTTCATCGTTTTTCGCAATTTCACTTGCTATCCAAGCGCGTGCTTCCMCCTCCAGCCACGATATACTACYATATTTTCTGCTGCTYCTTGCATTTAGTGTCTCCATGCAAGCAYGAAGGTTACACACATTTTGTTAATGGTTTTTCATAAATTGAATTGAGAGATTTAGTAGCATGAGACATTTATACAATTTTtagtcttttttttttaggattTCACATATACTTAATCTACTCCCACCCCGTTCACAAATAAAAATTAAATAATTTMTCTTTCAAAAATTGATATGAAATGAAATYGRTYCCTTCAAAGAAGAATCTAACACACTCCGCAAAGCAATATGCTAATATGTGAGTTTAAATATTTATTTTTGTTTACGAAGTAATAAATAGAAAAATAAAAACATCCATGaAAGAAaaattaaaaaaattaAATAGTACTTTAGCCACAAAaaaaatactttGGATAGCTTAAAGCCTTAAAGAGAAAAAAGAAATAAACATAGTATTGAGCTAAATACAACAGTTTTTTTTATGTATTAAATTATTTAGTATTCAMAAAYTATATTAGACATAAGARTAATTAATTTACAATAATATATCCWGAAAAGC

>Cs1g04000

GGCCATATTTAATYGCTTGATCGAGAGTAATTTTCATCSCATTATGATCATGATCTTTAGGATCAAACATAGATTYYRTGATCTTCATTTTATTCCTAACATCCCCTCTCAAACAAATCTCAAGTCTCCATTTTGTAGTTAGGTTGTGAAATTAAAGTGGGTAGCTATGAAGAATCTTTGATAAATATCAATTAATAATATTACTATTGACAAAATACTGAACATAATATGAATTTTTaattgaaaaaaaaactgtttACCAAAGCCACTTTTAACTTATAAATTAAGGGTTAAAAATTAAGGAAAAAAACACCTACATCCCGAAAGTATGAAAAATGGTCATAAAAACTTTTAAATTTTCACTAAAGGACAGCAAAATGCCTTTTTGACTGTAATGTCTTTTGAATTTCATAATTAGAAAATTGAATTTAAATTTTCTTAAAAATAATTTAAATTTAAGACAAATAACATAttatatctagtannnnnnnnnnnnnnnnnnnnnnnnnnnnnnnnnnnnnnnnnnnnnnnnnnnnnnnnnnnnnnnnnnnnnnnnnnnnnnnnnnnnnnnnnnnnnnnnnnnnnnnnnnnnnnnnnnnnnnnnnnnnnnaaatttaaatTTAAAATATTTTATAATTGTAACTTTTAAACTATTGCTGCTAATTAGCAATATTAAAAGTAACCTCACAGGAGGTAAGTAATAATTTCCCATTTTAAAAATGGATGACGAAATAATTACGATGGGCAGGTAGAAATTTACAAACATATCTAGTCTCAGATTGAGGCTGAAATTAAAGGAAAGGACAAACAAGTAAGTTCACTTGATAAAGGTATGTAACTTCCACCTATAAATTGCTTGCAATTAATGAGATTCTTATGCAAAGCACATCTCTTTGTTCCAAAAAATAATTTCTctggaaattgcaaggcaatgaaaccaaccacgAATATTTCGTCCTCCTCTCTTTTTCTTGCATTGCTTCCCATAGCATCAATCAGCA

>Cs1g13480

agctttttgaatttgggccccgttttgtttaacccaataaagtcataagtgggctgtgtaagtgacaattgcgacggcttattttgcagtcgcaagagctgtcgcgaaagacactttcttttgtagtgtggattCATTtGGTTTTAAGGTTGGACACGGTTTCTGATCCAATGATCCAATCTACCAAAGGAAATGCTGATTTTCAGGRTCGTTTTCGAGCATTTGCATTTCATCTTCTCAGATATTGAACGGGTTCAATAAATATTTACATRATAATTTTTATTTTGAAACTTACATCATAAGGTTAYTAACTTGATCTTAATGACTCCAACTTATATGSGRCACATGACCAATATTAGATACATAGATATTTGTCAAAAATGTATATTTTCAAAGTTAATAACCGTAAAGATTACGAAGGTTTGTTCGCATTAATTGAAGGACTTGAAAAAACATTACGTTCCTTCAATATATTTGGCTCAAACAATCACATAAAAAGTCcaagtcttattcggggacacctatcacccagtgatggtaccttctaagactaaaaaattgaCTTTCCTAGTAGaaacatataattgaattttttttttttttttgatattaatcacgaggtatcctggggagggccccaactgtgggaggcacctttaagcctataccacaacccagactagaagtccccgctcgaaccgggaggcacaggttctcccaacaaatacgacttccctgcgactcgaactggggaacaaacccagtcaagccacttaaggggactccattgccagtggggccaacactttgttggttataattgaattaataccacnnnnnnnnnnnnnnnnnnnnnnnnnnnnnnnnnnnnnnnnnnnnnnnnnnnnnnnnnnnnnnnnnnnnnnnnnnnnnncatcaattaactaacattgcagtgctcatacattctcatcagatttcttcatttcctaaatagttgaatattgaattctatacaaact

>Cs1g12870

GATATTATGCAGCGAGCAAAATAAGGTGAGCAATGAAAAATGTGATGTCCATACGCAAGATAAGAgagataaaaaatcaactTAGTTTttaagcaataacagctaataTTTATCGAATATTAATGCTGTAGCTTCTCCAAAGTTAGTACTAAAGACAACCTTAATTCATTTAGATGCTGTGATTTGTTTCTGATTTTAATTGTTAATTCGGATTCCACAAGTTCAATTtcctagtgttaTTAAATATGATGGATTATGTTGATCATCTTTGATATAGTTCTCTTCTTTAATTTTCAGAGCTTGATAATCACTTTTCAGAATGCCATCATTGTTGACCCCTCTCAAAATTTCATTCTTTTGCCTGCTGCTCAAATTTTTTTTCTATATTCCTTCGGAAAATCCATTTTAGCTGCACGAAATAATTTGATTCTGGACTGTGATATGAATGAACGAAAAATACAATGTAGAATATTAACAATTTTATGTAAATATATTTTGGTTTTGAGTTGTAATTTCTAGTTTCAAGACTTCGATTATTTCGTtccaaggggttttttTTTAGTAGTTTGTTGATTTTAAGATACCAAGAATGAATTGAATTTTCAAGagaaatttcattacAGAGCTCGAGTTTGGTGCCAAAGTTTGTGAATGAGTTAGTCGCCTTTCCATTTGACACGTTTCTTACACGTGTTAAGTTTGTGGTGTTACAAATGTCTTTAGACGCGTGATGCCACCTGTGATAGCCCCGTTCTCGACACAGCTCTAACGTAAACTTAAATAAGAAGACGACCCATTCATCTCAAAATTAAAATATCGCCTCTcgaataaccaaACGATTGACGTCAAACAAAAAACAACAACCTATCCAGCAAAGAAAataacacaaacacAAACACTGTTACTTAAACGGCACCGTCAcccaaaaaaaatttgagcgccaaatttgaaaagcttcaaaaacttccccgccaaaagcgatgaccagaatttagatcaca

>Cs1g07240

TTTTTAAAAAGATAATGAAAAAAATTGGAATTGTGACCCTAAAAAATAATCAACTTGAAAAATTAAtacatattaatatatttaataaattaTGAATCAAATGTGAAAAGTAAGTAAAAAAAAAAAGGTACATATATTTGCTTTTTGCATTGGGAWTAGTATTCGTTGTTAAGCCCGGCTGGTGTTAAGTATTGAATTAATGGGCAAGAGTTGTGAAATATGTATCCAATRAATTTCTAACCATTGATTTGGTGCTTAGAACAAGTTAAACTGAGCACCGAATTCATTMCTTRTTATAAGTGGGTAGAATGAGACATCTCATTAATTATTATCTCATGGTYGGTAGGCCATCCATTCCCTTGTCTACCCACTTGCGTGAGCAAACTAAATTACCATTTCTAGTTAGTAACATTATGGTAAAGCCYCGTCCCATTGACTAGTTTTGTGCCTTCATCATTTAMAGTAGCACTAGCACATGAAATCCCATTCATTTTTATCATTTATAGTAGCACGAGCTGATYACATCCCATTCCCATCTTCAWGCTCACAGAAGCTACACTTTGTAAAttatataatatccttcGGCTTCATTGTTTCATTTCCACATTGTTTCCTTTGCTTTTCTGGTAGATAGGAAAACTTGAAGATCAAGGGAACAGTACTGGAAGATCTCTGGCACGTTTTCTGTCTGTAGCATCACTTCTCAGGTATGGAATCagtyactttttttttttttactttATTTTTYCTCTTCTGTTATCAGCTAAAGAGAATGATTTGAGTTTTCACTTTTTAAATTTTTATTTTAATCCTTTgaagctctggagATAGCTYCAAAATTTTTAAGTCTCAAGCAGTGCACTTGGATTCATTTTTKTAGTTTTACTAGACTTTGAAAMTTTTAARACTAAWGAAATTTTTCAGARGTCCCTAAAAATTTACAGAAAGAWGAAAAAAATTAAAAAAATTAATTTGTAATTTTYTTAAAGTTAAACAGTCGG

>Cs1g21270

gtatcctgatcagcttccattttcttgccattctcttcatcttccattgaatcgccgcaacatgccatcattctcgtgatatttttcgccccaaagagttaacagacttataaaattgatgttcactgtggttttaactgaatttggaaaattggagccaagaatctggaggggcgtcccctctttttatagagtggcaaagatactcattttgatgtggaaaatggtaacggccttcgagtgaacggtctgccgttagatttagtgggaccagtgttgaaatggatagcagcgattgcgccctggccggggactgattgatagcggctgatagaaagggacgacgtttttcaagtcataagacgactatctcacagaacggcagtggttcggcttgtcgtttatattgaaagttgaagccggtaaatgacgtagtctggcattgccatttgcattacgtacattcttatcgagcaggcaaggaagtcaagagaataattaatggtttgtgcaaaaaaacaaagcgaatatgagaaataaaatgaatgacttagcgttggtgccgtgttaagcagaaaagttctcatgattcagacgtatgcagttctctagaagtagagttattgtgctgccaatcatgacatttagattttatactaaatgagattgatgggcgatgtagacgacaaatacaaaatagagaagacaaaatttgtttcgatgattccatgatcatttatttgattgattcatatatactcgagtttggcgtttgctagcttcaccttggccttgggtgatggaatcaaccgaggggatagcgaatcgaggtagttttatttatttatttattattattattattatttttggtgaaaggtttggaagtagtatttaatttgccaaaagcttttatttttataaacaaaatttatttacgaaaaaaatggaaaaagacagtacccatcacacgcacgcgtataaaacaatagcacactaca

>Cs1g07430

TTTTTTTTGGTTATTGTTGTGAAGGTTTCGACTTGTAATAAGTAAGCTTGGCAAAAAAGCACGCGTCCTGCGGACATAGACTCGATCCTTCACATGAGGGcttgcaaaaaaaaaacttaagGAAAAAGAAAGGCCAAGTCAGGCCGAACCTCTATTTCACTAATATAAATTAAAATaacaaaaatnnattgAAAAAGAAAAAAAAAAGAAAACATAAATCTCTATTCATGAATCACTAATGATTCAAAAAATTTACGGTTATAACATTAAACTCTCCCTTGGCCTTAATTAATTAATAAGATACTTGAATTAGAGAGTTCAGTACTAAAAAATTAAATATATAAATttttattattattTTATTTTAAaATATTTATTTTCTTTATTTATTATTTATTATAACAACTTATTAATTCATTATAAACTTACAATAATTTAAAAAATAGTTTAAAAAAATATGTGAAAGTTCATCTAGACGCTYACACTTTTCTTAAAAAGAGTTTCAGCTTAGGCTTAAAAAAGCTTGGCCCAAGCATGCAACTTGCTATCATATTCATAAGGAGAACTTATTAAATGACATTGATTTTGGTGAAATAATTTATGTTTTTCGCTTACAACTTGCAGATTAATTATTTTCATTTATTGTCAAGGATTTGACAGTAAACTTTCAGGTTGTtctaattttttttctttctGTTATAAGAACAGAAAATTTCTCTTCCAYTTTACTTAAAMTATAATCTATTCAGTTTTTGTTGATATGATTTATAATAATTTTTGTTCGAAAAAATATAAATAWTCTTTTGAAAGAAAAATACAGTTTAAAGAGACCATATAATGGTTTTTATTATTTAATTTTAATAAAAATAAGTTTAACTGGTTTGACATATGATTAATTAAATTTTTAATAATTAAAATTAAAATATTATCATAATTAATTTCTAATTTTATAAGCGGTTATATATTTATCCCAATGTTGTAATTTTAGAAGAGCATTGTAG

>Cs1g11180

ACTTTCGTACGTGAAAATGAAAAGTAAAGTTGATCAAGCGCCATCTTCCCATTTAATAAAAATGTGCTTATGTGCATGCCCTTTTCTCgtatatgtgttgtnnnnnnnnnnnnnnnnnnaatggggtccaaaaTTGAAACCCAATGGGACGTTCCCACGTGTCGTTTTGTCGTCTTGTGGTGACGAAGCACCTGGGTACGGCGACAAGTGGCCGACAAGTAGATGGGGAAGTTGATGTGGAGAGGCCKCTTAGTTGCCTCCCATAGTGCTTGTTCATCACAAACTTTATCATGAATTATTATTCATCGGCATTGTTCGCAACATGGTCTCAATTCTCATCAGGTCTTTTGAGATAAGCATCAAGAGTCGAACTTTAAGTTGATAAAACAGAAGATAAAAAGGGACCGATGTCATTGATTGTGAAAGCACTTACCATGTTTGAAATTATCTGctttgtctattggtgagAGAGAAATTAATAAAGCTAATGAGATTCCTTTGTTGGGTTTGCCTTTGCTTTGTCTAATCAAATTGGACCCCAAATCGTGTTGTTAGATTCCTCTGTCCATCTGTATCATGTTGTCAGTAGATTGGAKCTTGTTTCAAGAGAAAAGCCATAAAGTCTTTTGCATCACTATCTCAATTGATATTCGCTTCCTCCTAAAATTTATCATCAGAASTTGCATATAttcctcttctgtgCACAAGCACAATCATGTTTAGATACCACatgaaagatatggcTGCTTAGAATTAAATGTTTGACTTAGGATTAAATAATTACAAGTATCTATGGGTCAAATAGTTATTGTCAAATATTTATATTATATAAGTATGAARTGACTCAATGTGGGAAACCCCTTGggatagtttttttttttttttttcatttTTTTTCTTTTTCAGTAGAAGATCGGCAAAATCCAATTTAATTTTTTTAACCACTACCCATCTCAAGATTAGATCCCTGATGGACCCTTCTCTTAGTTCAGTAGACGAGG

>Cs1g10630

acacaactgtaaggttgtattctaagcaatgacaatttaaatagcaaaacaaagaaaattgatgataaaatatgagaataaagtagagaggattaattataacnnnnnnnnnnnnnnnnnnnnnnnnntattttgAAATATCAATAAAATTATACCGATATATTAGGACAAtattacttgtgtTGCATAAATTAAAAAAGAGTAYATCAAAGGAAATAWAACCAAATACAACGTCTAACTAAAAWTTTTCTACACTCATTTGCTCCAACRGAATTAGAAAAATGATATCAAATAGGTACAATACACTAATAACTTSTATAGATCTATTTACMaaagctttactgacRTAAGCTTTTGAAAAACTAGTACKYAACATTAGCATGCGTTGGCTCTGCAGATTATAAAATTGACATATGTTATTTTMAGKAGGGAGGTTTTATTGAGGTGTACTAATCAAGTCCAACACTACTTTTAATATCATAAAAATTATATGCAAAAACTTAATTAYGTATATGTTGATCGGTTCTTTTTTTTTAATTAATTAAACATCAAGTTATTatattatacatatatttataactgttatgataacagatcattacactgatatttataatcagatatatatatgagactttattatannnnnnnnnnnnnnnnnnnnnnnnnnnnnnnnnnnnnnnnnnnnnnnnnnnnnnnnnnnnnnnnnnnnnnnnnnnnnnnnnnnnnnnnnnnnnnnnnnnnnnnnnnnnnnnnnnntatatttaattaagcgatgttaaattaatgggaactCGAACTATTGTCCTCATAGTCATGCGATTGGCCCTTGTTATATGGGACTTAaagtgtggcattTTATTGTCAGGGGTATTTTAGATTTTTCATTCTTGAATAATTGTCATATTTGTGAATTAAATGACTGCCGTGCcatatgtttgcaAATTTTAACCACCTRCGCAGCCGGYGCAAACTCCGTCATTACAGTCTTCCTTTTCCTC

>Cs1g24600

TCCTCCGCTGCTGCAACCACCACTCCTAGTGGTTATAGTGTTACCGGGGAGGGGCAGATGCGCCACCACGAGAAGAAAGGAATGATGGAGAAGATCAAAGACAATCTTCCTGGACACCGTCACTAGACTACCTGCCTGTACKTGTCTTTCGTCGACGACACATACAATACTATATATACTAGTGCTTTTTATTATTTGCTATATTTTACACRAGTGCTTTTGTAAAAtgtccataattTTCGTAGTTTnnnnnnnnnnnnnnnnnnnnnnnnnnnnnnnnnnnnnnnnnnnnnnnnnnnnnnnnnnnnnnnnnnnnnnnnnnnnnnnnnnnnnnnnnnnnnnnnnnnnnnnnnnnnnnnnnnnnnnnnnnnnnnnnnnnnnnnnnnnnnnnnnnncatctctttttactagttatgcatcttgggatagtattatgtaacacatacaacaagctgtcaagctaTTTGTACTCGTGGGTGGGCTGTTCCTTTAGTTTTGTGTATTAATACAGCTTTCTATTGTATTTTGTTATATATATATATTGAACTAATTAATAAGAGTTCTTTTGCTAAACGTTTTTTTATCGATTCTCCACACTRCACCGCACCTTAAGCCTGCTTATTCGTATAACAAACTTTCATTTCTGAAACTAGATAGTATCCTGATTCTTACAGCTTATAAGACATGCCAACGGACGGCTTGTCGGTATGCWCTTTCGTTTCAATTYAAATGACGTACTGGAGGAGAAACAGCGGAGGACATCTCTCATWACGGGTATCTGTTAttggggattttGATAATATGGTGAAAAAACAAAAAGAAGTAaAGAACTCAGCATCAAaATGTGAGAAATTGGTTAAACCTCagtAATAGAaatttggttaaacATTCAACCAATTTAAGACAAAACATCTTTTAACAATCCGRCGTTGAGAAATAGAAATTTGCCTTTGGTATCACGGATGAAACAAAAGCCATTAGTATGATACCGGTACCCTTTC

>Cs1g07480

attattttaatttaaatattatcaaaactctaaccatgaacaacaataatgcctcgaagctgagagttttttgtaattttattttatattatttacttcgaattgtaattaaactatcttatcctctacttctctgtttttattttttgcctttaaaaagtaattcgataattgattggaatttggatgtgtgtttatcctggagaaacaggtaagagagattgatcnnnnnnnnnnnnnnnnnnnnnnnnnnnnnnnnnnnnnnnnttataattataattcatttaataattattatatttttttaattaaatagaggttgtttttggaatttaaaattatagggggtgaattagtactctannnnnnnnnnnnnnnnnnnnnnnnnnnnnnnnnnnnnnnnnnnnnnnnnnnnnnnnnnnnnnnnnnnnnnnnnnnnnnnnnnnnnnnnnnnnnnnnnnnnnnnnnnnnnnnnnnnnnnnnnnnnnnnnnnnnnnnnnnnnnnnnnnnnnnnnnnnnnnnnnnnnnnnnnnnnnnnnnnnnnnnnnnnnnnnnnnnnnnnnnnnnnnnnnnnnnnnnnnnnnnnnnnnnnnnnnnnnnnnnnnnnnnnnnnnnntaatttttagtgataaatTTAATTTAAaTCAATTTTTAATATTAAGTTACACAtttggactaaactagatcaaattttaaattagatattagaccttgttttaatggattccaagtcaagcaactaggcttataaattataaatgaaagaaatatgtgtgtgattttaaaattaattttgaacttgaatttatacatttaattaattcaaagaggtttggaatatatcatctgaaatttttggatgggacttggttaatggttgttnnnnnnnnnnnnnnnngacacatatggacagcatttatcataatttggtaaatcataaagattaaggattggacgttgttaccgcaatcagaaaataactttcatcgcaataaatgtgc

>Cs1g10380

gaatgtacccTACTCACCACARGCAACTATCTTACATCATCATGTTTAAGAATGCATCATCAAATGTTGTAGTCCACAAAATTTAAACAATTATTTAAGTAAGACCATGCATAATACTGAAAAAAACTGCTTCATTTTCTTAATTAATTCTCGAATCACTCTATTCTCTTTCTCTTCTAATTATTCCCACGCCCGCCATCCCTTATCTATTTGCAATTGCAGCATGTCGGgggatcaatgcataTAGAAAGTAACTTAACATGTATTGTKACATAGGCTGTTGCTTGGCAATTCATTGRTGAGGGAGTGAGTTGAACCTTTCGAAAGAAATACTTAATTCCGCTTTCATAATTGCATCAGGTGTATATAATATACGGTAAACGCTCTTTATTCACTAGGATTTGAACTAATTATSTAATTAGTTTTATACTTTTAAAAATTATTCAAAAGACTTATGTTGTATATCTATATATGAATAATTAACCCAAATTTTAAAGAGTAAAATTAGGAGATTTTAAGCCATACAAATAATAATWTKWAAGKAGTAATTATTTTTTCTCCCAACTGCACCTAAGCATGACTAAAACAGTCTCCCATATCCCAATATTAATTTCGCCATAACCGTGTGACAAGTATATGCAATTTTATTTAGCAAATTATCGATCATCAAATTAAACTTTTCTCATTAGCATGATTCTCTTAACTCTAATTCTTTGCTTTattgctgcctttTTGTTAAAGCCAACTCATCAATTCATTTATTATAGTTTTAAAATATTTTCAACCAACAACAACAATACCAAGCATATACATCAAGGAAAAATTATTGATATCTCGATTTCAGCRTCCCGACCCATCACCATTGATTGCATGGTCCAATTACTGTTTTACTTTTTATTTGGAGTTATGGATGTCAATTAATGATATCGTACAAAACCATTCTCTGATATTTCAGCTCCATTGCTATAATTCATCAAACTCTCTACTCCATTTGTTCGTAT

>Cs1g06670
[truncated: 29,733,924 more chars]
